# GATA3 Promotes the Neural Progenitor State but Not Neurogenesis in 3D Traumatic Injury Model of Primary Human Cortical Astrocytes

**DOI:** 10.3389/fncel.2019.00023

**Published:** 2019-02-11

**Authors:** Hilal Celikkaya, Mehmet Ilyas Cosacak, Christos Papadimitriou, Stanislava Popova, Prabesh Bhattarai, Srijeeta Nag Biswas, Tohid Siddiqui, Sabrina Wistorf, Isabel Nevado-Alcalde, Lisa Naumann, Violeta Mashkaryan, Kerstin Brandt, Uwe Freudenberg, Carsten Werner, Caghan Kizil

**Affiliations:** ^1^German Center for Neurodegenerative Diseases, Helmholtz Association, Dresden, Germany; ^2^Center for Regenerative Therapies Dresden, TU Dresden, Dresden, Germany; ^3^Max Bergmann Center of Biomaterials Dresden, Leibniz Institute of Polymer Research Dresden, Dresden, Germany

**Keywords:** GATA3, neurogenic potential, neural progenitors, scratch injury, primary human astrocytes, SOX2

## Abstract

Astrocytes are abundant cell types in the vertebrate central nervous system and can act as neural stem cells in specialized niches where they constitutively generate new neurons. Outside the stem cell niches, however, these glial cells are not neurogenic. Although injuries in the mammalian central nervous system lead to profound proliferation of astrocytes, which cluster at the lesion site to form a gliotic scar, neurogenesis does not take place. Therefore, a plausible regenerative therapeutic option is to coax the endogenous reactive astrocytes to a pre-neurogenic progenitor state and use them as an endogenous reservoir for repair. However, little is known on the mechanisms that promote the neural progenitor state after injuries in humans. Gata3 was previously found to be a mechanism that zebrafish brain uses to injury-dependent induction of neural progenitors. However, the effects of GATA3 in human astrocytes after injury are not known. Therefore, in this report, we investigated how overexpression of GATA3 in primary human astrocytes would affect the neurogenic potential before and after injury in 2D and 3D cultures. We found that primary human astrocytes are unable to induce GATA3 after injury. Lentivirus-mediated overexpression of GATA3 significantly increased the number of GFAP/SOX2 double positive astrocytes and expression of pro-neural factor ASCL1, but failed to induce neurogenesis, suggesting that GATA3 is required for enhancing the neurogenic potential of primary human astrocytes and is not sufficient to induce neurogenesis alone.

## HIGHLIGHTS

- Primary human astrocytes do not induce GATA3 after injury.
- GATA3 promotes neural progenitor state but not neurogenesis.
- GATA3 increases the GFAP/SOX2-positive cells and ASCL1 after injury in 3D.
- GATA3 reduces lesion-induced scar-like collagen deposition.

## Introduction

Astrocytes bear multiple vital functions such as maintaining the ion homeostasis, contributing to the blood–brain barrier, restoring synaptic integrity, regulating immune response, and acting as neural stem cells (Kettenmann and Ransom, 2012). Especially the glial nature of the neural stem cells (Doetsch et al., 1999; Laywell et al., 2000; Doetsch, 2003) suggests that these cells in our brains can be used for producing more neurons in case of injuries or diseases where neuronal loss is prominent, and remedy necessitates neurogenesis (Fawcett and Asher, 1999; Silver and Miller, 2004; Buffo et al., 2008). However, in diseases and injuries of the central nervous system, mammalian astrocytes cease to produce neurons but rather undergo reactive gliosis, where they amplify themselves and form a scar tissue (Yiu and He, 2006; Fitch and Silver, 2008; Sofroniew, 2009). Therefore, finding out molecular mechanisms by which reactive astrocytes can be coaxed into neurons will be of utmost importance for regenerative therapies as these astrocytes are the imminent cell types around the lesion site.

Several studies using reprogramming and direct conversion succeeded in converting astrocyte into neuronal subtypes (Heinrich et al., 2010; Cherry and Daley, 2012; Guo et al., 2014), yet an alternative approach can still be to learn these mechanisms from regenerating vertebrate brains that can efficiently convert glial cells into neurons in case of injuries (Rolls et al., 2009; Karl and Reh, 2010; Kizil et al., 2012a; Hong et al., 2014; Cosacak et al., 2015). One such regenerative organism is zebrafish and it can regenerate its brain upon traumatic injuries and neurodegeneration (Zupanc, 2008; Fleisch et al., 2010; Kroehne et al., 2011; Baumgart et al., 2012; Kishimoto et al., 2012; Kizil et al., 2012a; Marz et al., 2012; Cosacak et al., 2015; Alunni and Bally-Cuif, 2016; Kizil, 2018; Kizil and Bhattarai, 2018). This regenerative ability is remarkable and seems to depend on molecular programs that zebrafish utilizes in its neural stem cells that are of glial nature (Grandel et al., 2006; Zupanc, 2008; Kizil et al., 2012a, 2015; Alunni and Bally-Cuif, 2016). One such regenerative program involves the activation of Gata3, a zinc finger transcription factor, after injuries in the adult zebrafish forebrain (Kizil et al., 2012b). Blocking Gata3 activity hampers the proliferative and neurogenic ability of zebrafish neural stem cells in the telencephalon, suggesting that Gata3 is a regeneration-induced program for zebrafish neural stem cells (Kizil et al., 2012b). Therefore, Gata3 can serve as a promising candidate factor that might be used to coax astrocytes to an injury-induced neurogenic fate. However, the effects of Gata3 on the proliferative and neurogenic ability of human astrocytes are not known.

Following traumatic injuries, the astrocytes located in the brain parenchyma react by proliferating and forming a scar tissue around the lesion site (Fawcett and Asher, 1999; Silver and Miller, 2004; Yiu and He, 2006; Rolls et al., 2009). Astrocytes inherently bear neurogenic ability in culture, all astrocytes of the developing cortex can form neurospheres (Laywell et al., 2000; Temple, 2001; Doetsch, 2003; Gage and Temple, 2013) and adult astrocytes can be reprogrammed *in vivo* and *in vitro* to form neurons (Heinrich et al., 2010; Cherry and Daley, 2012; Guo et al., 2014; Magnusson and Frisen, 2016). However, astrocytes are not neurogenic after injury *in vivo* (Costa et al., 2010; Robel et al., 2011). A recent study demonstrated that the scar-forming astrocytes that populate the lesion site after stroke are derived from the subventricular zone astrocytes that act as neural stem cells (Faiz et al., 2015), suggesting that these cells can still manifest their neuronal progenitor characteristics under certain conditions, which cannot be manifested within the injury context. Therefore, parenchymal astrocytes are intriguing cell types that can be targeted for regenerative therapeutic applications provided that we can coax them to form neurons. In our study, we hypothesized that Gata3 might enhance the neurogenic potential of the human astrocytes, and we aimed to investigate the effects of overexpression of Gata3 – a candidate protein that might impose a regenerative neurogenic potential to human astrocytes.

## Materials and Methods

### Ethics Statement and Experimentation Permits

All experiments were performed according to appropriate safety regulations in DZNE Dresden and TU Dresden with the following permits AZ: 54-8451/247/2 and AZ: 54-8452/78/10. In overall, no performed experiment involves ethical concerns, security issues or violation of the rules of Convention on Biological Diversity Cartagena and Nagoya protocols.

### Primary Human Astrocyte (pHA) Cultures

Primary human astrocytes (pHAs) isolated from the cerebral cortex at gestation week 21 of human fetuses were obtained from ScienCell Research Laboratory (SRL, Catalog Number 1800) at passage one and delivered as frozen stocks. The cells are certified to be negative for HIV-1, HBV, HCV, mycoplasma, bacteria, yeast, and fungi. pHAs were seeded on cell culture treated 24-well plates (Nunclon, Delta surface, Thermo Scientific) and cultured with Astrocyte medium (SRL, Catalog Number 1801) supplemented with 5% fetal bovine serum (SRL, Catalog Number 0010), 1% astrocyte growth supplement (SRL, Catalog Number 1852) and 1% penicillin/streptomycin solution (SRL, Catalog Number 0503) in an incubator with a 5% CO_2_/95% air atmosphere at 37°C.

### Preparation of Lentivirus and Transduction

The lentiviral transfer vector containing human GATA3 was pReceiver-Lv53 and was commercially available (GeneCopoeia^1^). This is a third generation lentiviral vector that does not require Tat activity. Lv53 can be classified as a self-inactivation vector due to a deletion in the 3′ LTR (long terminal repeat). When the 3′ LTR is copied at the 5′ end of the vector genome during integration of the lentiviral plasmid into the genomic target cell DNA, the absence of full 3′ LTR prevents the production of full-length lentiviral RNA. The lentiviral particles are incompetent for replication because the two transformation vectors cannot encode the HIV-1 gag, pol, and rev genes. These replication genes were expressed from a separate packaging plasmid lacking a psi packaging signal. We used a second-generation packaging system based on the pCD/NL-BH plasmid (Mochizuki et al., 1998) and a sheath plasmid pczVSV-Gwt (Pietschmann et al., 1999) encoding the HIV-1 gag/pol structural proteins and vesicular stomatitis virus glycoproteins encoded.

For the production of viral particles, we transfected packaging cells (HEK293) with the vectors pczVSV-Gwt and pCD/NL-BH and the transfer vector using standard transfection protocols in T175 cell culture flasks with 25 ml medium. Viral supernatant is collected and concentrated by centrifugation, and the resulting viral particles were used to transduce pHAs. Replication deficiency of the lentiviral particles were performed as follows: HEK293 cells were transduced and 24 h after transduction, cells were washed with culture medium, and infected cells were cultured 48 h more. The medium from transduced cells was collected by aspiration and the presence of infectious virus particles were analyzed using microscopy for GFP fluorescence. None of our experiments resulted in replication competent lentiviral particles.

### Scratch Assay in 2D

pHAs cultured in growth seeding density according to manufacturer’s instructions at 5,000 cells per square centimeter at 24-well plate (Nunclon, Delta surface, Thermo Scientific), from passage 6, until they reach 100% confluency (approximately 8 days after seeding). Afterward, with a 1,000 ml pipette tip scratch lesions were performed diagonally as described (Cory, 2011; Matsubayashi et al., 2011; Gao et al., 2013).

BrdU was administered at 10 mM concentration for 8 h in an incubator with a 5% CO_2_/95% air atmosphere at 37°C followed by two washing steps with cell growth medium for 10 ms each. The control wells without the scratched lesion followed the same culturing protocol as well as BrdU treatment and termination of culture period for fixation and analysis.

### 3D Cultures and Lesion Assay

starPEG-Heparin hydrogels were prepared and 3D cultures of pHAs were performed as described before (Papadimitriou et al., 2018). Lesions were performed at 14 days of culture using a glass capillary by impaling the center of the gel vertically once. The gels were grown 1 more week until 21 days.

We performed the lesions in comparative developmental stages so that the outcomes would be comparable – day 8 in 2D cultures and day 14 in 3D cultures. We believe that these time points best reflect the comparative developmental stages of the primary astrocytes in these two systems.

### Immunocytochemistry

Cells and hydrogels were fixed with ice-cold 4% paraformaldehyde and incubated at room temperature for 20 min followed by three washing steps with PBS for 10 min each in room temperature. The DNA denaturation step for BrdU-treated samples was performed by incubating the samples with 2 M HCl (Fluka) for 10 min at 37°C followed by three washing steps with PBS in room temperature. The samples were permeabilized in blocking solution (0.3% Triton X-100, 10% normal goat serum, 1% BSA in PBS) for 20 min at room temperature. Samples were incubated with the listed primary antibodies in blocking solution overnight at 4°C. Primary antibodies: anti-GFAP (1:1000, Abcam), anti-SOX2 (1:300, Santa Cruz), anti-TUBB3-160 (1:500, Life Technologies), anti-GATA3 (1:300, R&D), anti-BrdU (1:500, Bio-Rad), anti-GFP (1:500, Abcam). Secondary antibodies (Alexa Fluor, Invitrogen) were used 1:500 dilution in blocking solution at room temperature for 4 h. The cells were counterstained with DAPI (1:3000 for 10 min at room temperature, Invitrogen).

### Fluorescent Imaging

The imaging was performed as described (Bhattarai et al., 2016, 2017) in an inverted Carl Zeiss Apotome 2.0 microscope. Images acquired with the Carl Zeiss ZEN blue software. For 2D cultures, 36 images from a single well were taken with automated stage function of the Zeiss microscope using 10× objective. Resulting tile images were stitched together to create a larger field image. A max projection was not needed for 2D cultures. For 3D cultures, the images were obtained using a Leica TSC SP5 MP microscope in confocal mode equipped with an IRAPO L 25.0 × 0.95 water objective. The image acquisition was carried out in X, Y, Z dimensions (620, 620, 200 μm physical length, respectively) with a step size of 1.68 μm. Recorded images were analyzed by Arivis Vision 4D and FIJI software. Maximum projection images were created for representation.

### Whole Transcriptome Sequencing

RNA isolation from 2D and 3D cultures, library preparation and bioinformatics analyses were performed as described (Bhattarai et al., 2016; Papadimitriou et al., 2018). GEO accession numbers: GSE116662 and GSE117906.

### Quantification and Statistical Analysis

The quantification was performed using Vision 4D software (Arivis) on the acquired images at 10× magnification. The statistical analyses for 2D cultures were performed using six samples per experimental conditions. Thirty-six images were taken per culture well (2D) and stitched together for quantifications. Student’s *T*-Test and ANOVA analyses were used (GraphPad Prism) as the samples conform to normal distribution according to Ryan-Joiner test and D’Agostino-Pearson test. The levels of significances are ^∗^*p* ≤ 0.05, ^∗∗^*p* ≤ 0.01, ^∗∗∗^*p* ≤ 0.001. For 3D cultures, three sample images were taken randomly in one gel, and quantifications were performed on 200 micrometer-thick Z-stacks using Arivis software. At least 1,000 cells were counted for every sample in 2D and 100 cells were counted in 3D. Statistical analyses were performed by JMP software and significance was calculated by Wilcoxon/Kruskal–Wallis test using rank sums. Chi-square values were calculated using one-way test as the samples in 3D cultures do not conform to normal distribution. We used two different statistical tests for 2D and 3D samples because 2D samples conform to normal distribution and we could count all cells in a well, while 3D cultures do not conform to normal distribution and three random sample blocks were analyzed per gel. Therefore, to increase the statistical significance and reliability of the methods, we used Wilcoxon/Kruskal–Wallis test using rank sums and chi-square analyses for 3D cultures.

## Results

**FIGURE 1:**
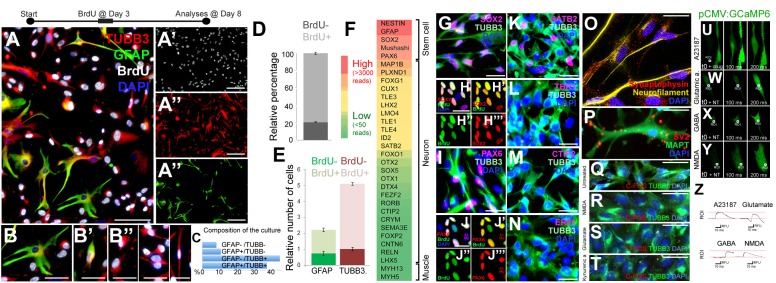
**(A)** Immunocytochemical (ICC) staining for TUBB3, GFAP, and BrdU in 2D cultures of primary human astrocytes (pHA). **(A’–A”’)** Individual images of each fluorescence channel. **(B–B”)** Morphologies of GFAP and TUBB3-positive cells. **(C)** Percent distribution of individual cell types. **(D)** Graph quantifying all of the cells for BrdU incorporation. **(E)** Graph quantifying glia and neurons for BrdU incorporation. Abundance (relative number) of cells are shown in a relative scale. **(F)** Expression heat-map for selected stem cell and cortical neuronal markers. Red: high expression, green: low/no expression. **(G)** ICC for TUBB3 and SOX2. **(H–H”’)** SOX2 and BrdU staining as composite and individual channels. **(I)** ICC for TUBB3 and PAX6. **(J–J”’)** PAX6 and BrdU staining as composite and individual channels. **(K–N)** ICC for TUBB3 with SATB2 **(K)**, TBR1 **(L)**, CTIP2 **(M)**, ER81 **(N)**. **(O)** ICC for synaptophysin and acetylated tubulin. **(P)** ICC for SV2 and MAPT. **(Q–T)** ICC for TUBB3 and C-FOS in untreated cells **(Q)** and in cells treated with NMDA **(R)**, glutamate **(S)** and kynurenic acid **(T)**. **(U–Y)** GCaMP imaging in neurons after addition of the calcium ionophore A23187 **(U)**, glutamic acid **(W)**, GABA **(X)**, and NMDA **(Y)**. All recordings are performed at the end of culture period. **(Z)** Green fluorescence histograms for **(U–Y)**. Scale bars: 50 μm **(A–A”’)** and 20 μm elsewhere. At least 1,000 cells were counted in every well. Percentages under the panels of **(B–B”)** indicate the ratios of depicted cell types among the whole set of cells in 2D.

To assess the proliferative and neurogenic properties of pHAs we cultured primary fetal cortical astrocytes in astrocyte medium supplemented with FGF and EGF (Figure 1). To determine whether the pHAs have proliferative ability *in vitro*, we treated the cells with bromodeoxyuridine (BrdU) at 3 days after the start of the culture with a 3-h pulse. This treatment labeled a significant portion of the cultured cells 8 days of culture (Figure 1A–A”’). The cultured cells included arborized, BrdU-negative, GFAP-positive, quiescent astrocytes (Figure 1B); GFAP-TUBB3 double-positive cells with retracted processes (Figure 1B’), which might represent differentiating astrocytes; and BrdU-positive TUBB3-positive cells (Figure 1B”), which include immature newborn neurons. In the current culture setting, the most abundant cell types were TUBB3+/GFAP- cells (44.3%) and TUBB3+/GFAP+ cells (Figure 1C), suggesting that the cultures predominantly contained proliferating astrocytes and early immature neurons. This composition of cells in the culture setting was useful for our main question that focuses on the proliferative and neurogenic ability of the human astrocytes. Consistent with the abundance of progenitor cells, 81.7 ± 2.3% of the cultured cells were newborn based on BrdU incorporation (Figure 1D). Similarly, the majority of GFAP+ (61.7 ± 4.2%) and TUBB3+ cells (81.2 ± 1.8%) were also BrdU positive (Figure 1E). BrdU was administered for 8 h on the third day of the culture and at 8 days of culture, more than 80% of the cells retain the label indicating that the majority of the cells produced in the culture come from proliferating progenitors. Whole transcriptome sequencing of the pHA cultures showed that neural stem/progenitor cell markers such as NESTIN, GFAP, SOX2, and PAX6 were highly expressed (Figure 1F,G,I), and the majority of SOX2 and PAX6-positive cells were BrdU positive (Figure 1H–H”’,J–J”’). This indicates that the ancestors of these progenitors were proliferative and the progeny of original BrdU-labeled astrocytes retain the label during the 8-h BrdU incubation time.

The cultured cells also expressed the cortical region marker SATB2 (Figure 1K) but did not express TBR1 (Figure 1L), CTIP2 (Figure 1M) or ER81 (Figure 1N), the neuronal markers DCX and NeuN, or the oligodendrocyte markers OLIG2 and O4 (data not shown). This cellular composition supports the observation that cultures contain mostly astrocytes with proliferative and neurogenic ability.

To determine whether the cultures contain mature neurons, we performed immunocytochemical stainings with TUBB3 and synaptic marker synaptophysin and SV2 (Figure 1O,P). TUBB3-positive neurons with an elongated morphology reminiscent of axons expressed synaptic markers synaptophysin (Figure 1O) and SV2 (Figure 1P); however, the synaptophysin- or SV2-positive puncta were scattered throughout the cells, indicating that the neurons formed in pHA cultures are not mature enough to form synaptic connections, which is a desired feature of our system because we were interested in the initial phases of astrocyte proliferation and neural differentiation ability. We further verified the immaturity of the neurons as they failed to express nuclear C-FOS after neurotransmitter treatment (Figure 1Q–T). To determine whether pHA cultures have functional neurotransmitter receptors, we transfected cells with the GCaMP6f calcium sensor expressed with the ubiquitous CMV promoter. As a positive control, calcium ionophore (A23187) induced calcium influx (Figure 1U,Z). Since it is known that astrocytes also have functional neurotransmitter receptors and can respond to neurotransmitters by increasing intracellular Ca+ levels (Gould et al., 2014; Deemyad et al., 2018; Vizuete et al., 2018), we specifically investigated the cells that have processes longer than 50 micrometers. These cells would represent neurons that have axonal processes rather than astrocytes with stellar processes. We found that glutamic acid, GABA or NMDA induced elevations in intracellular calcium levels (Figure 1W–Z), suggesting that neurons derived from pHAs express functional neurotransmitter receptors. Thus, functional neurotransmitter receptors and scattered synaptic stainings show that 2D pHA cultures contain early immature neurons.

**FIGURE 2:**
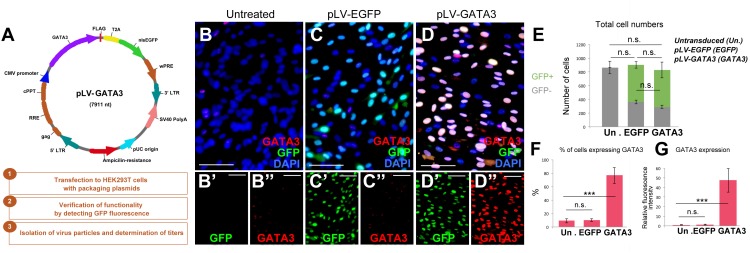
**(A)** Lentiviral vector for GATA3 and steps for generation of virus particles. **(B–D”)** Immunocytochemistry for GATA3 and GFP on untreated **(B)**, pLV-EGFP control virus-transduced **(C)**, and pLV-GATA3 virus-transduced cultures. **(B′–D”)** Individual GFP and GATA3 fluorescence channels. **(E)** Quantification graph for total number of cells broken down to GFP+ transduced and GFP-untransduced cells. **(F)** Quantification graph for percentage of cells expressing GATA3. **(G)** Quantification of fluorescence intensities as a means of GATA3 expression. Scale bars: 50 μm. At least 1,000 cells were counted per condition per sample. ^∗∗∗^*p* < 0.05.

After establishing the culture system, we generated lentiviral expression vectors for full length human GATA3 as experimental group (pLV-GATA3) and nuclear EGFP as a control (pLV-EGFP) to determine the effects of overexpression of GATA3 in pHA cultures (Figure 2A). To determine the effectiveness of lentiviral constructs, we transduced the pHA cultures at passage 2 with the virus particles, and passaged the cells two more times to passage 4. When we cultured the pHA cultures at passage 4 for 8 days, we found that compared to the untreated cultures (Figure 2B) where few cells express GATA3 weakly (Figure 2B), pLV-EGFP and pLV-GATA3 transductions gave strong GFP signal in the nucleus (Figure 2C,D) in approximately 72% of the cells in culture (Figure 2E). Despite strong GFP signal, pLV-EGFP-transduced cultures showed GATA3-positive cells comparable to the untreated controls indicating that virus transduction does not induce GATA3 expression alone (Figure 2C,F,G). pLV-GATA3-transduced cultures expressed GATA3 more abundantly (in approximately 80% of the cells, Figure 2F,G). These results indicate that lentivirus-mediated overexpression of GATA3 is an efficient method for expressing this factor in majority of the cells in culture.

**FIGURE 3:**
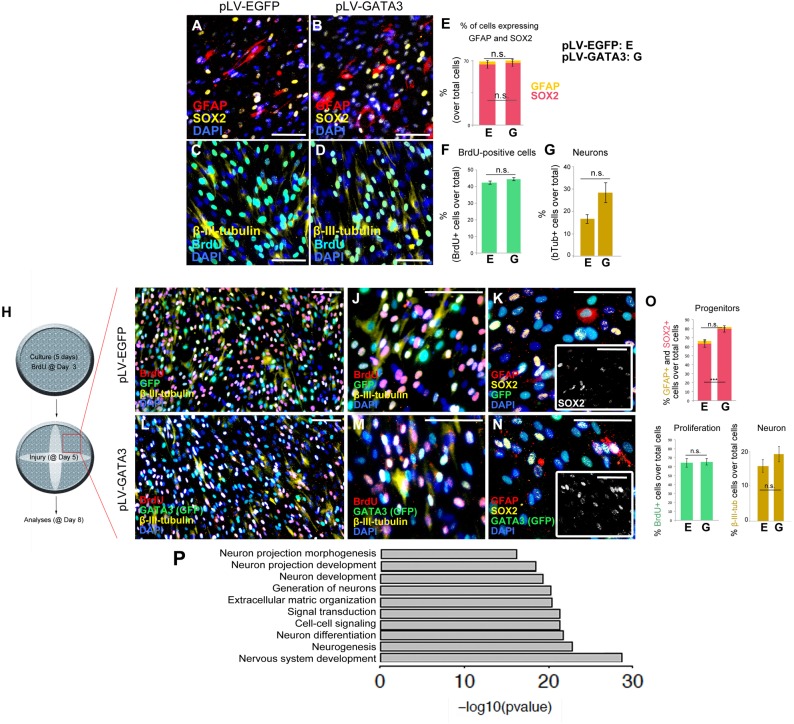
**(A,B)** Immunocytochemistry for GFAP and SOX2 on pLV-EGFP **(A)** and pLV-GATA3-transduced **(B)** cell cultures. **(C,D)** Immunocytochemistry for TUBB3 and BrdU on pLV-EGFP **(C)** and pLV-GATA3-transduced **(D)** cell cultures. **(E)** Quantification of the percentage of cells expressing GFAP and SOX2. **(F)** Quantification of the percentage of BrdU+ cells. **(G)** Quantification of the percentage of neurons. **(H)** Schematic view and experimental setup for the scratch wound injury. **(I)** Immunocytochemical staining for BrdU, GFP, and TUBB3 on pLV-EGFP-transduced cultures. **(J)** High-magnification image from **(I)**. **(K)** Immunocytochemical staining for GFAP, SOX2, and GFP on pLV-EGFP-transduced cultures. Inset: Individual fluorescence channel for SOX2. **(L)** Immunocytochemical staining for BrdU, GFP, and TUBB3 on pLV-GATA3-transduced cultures. **(M)** High-magnification image from **(L)**. **(N)** Immunocytochemical staining for GFAP, SOX2, and GFP on pLV-GATA3-transduced cultures. Inset: Individual fluorescence channel for SOX2. **(O)** Quantification graphs for percentage of GFAP and SOX2-positive neural progenitors, BrdU-positive cells, and neurons. **(P)** GO-term analysis for neurogenesis-related biological process compares lesioned samples of pLV-EGFP and pLV-GATA3. Listed categories are enriched in GATA3-transduced samples. Scale bars: 100 μm.

To determine if GATA3 overexpression would change the composition of the cultured cells expressing glial marker GFAP, neuronal progenitor marker SOX2, and post-mitotic neuronal marker neurofilament and newborn cell marker BrdU, we performed immunocytochemical stainings for GFAP and SOX2 (Figure 3A,B) and neurofilament and BrdU (Figure 3C,D) on pLV-EGFP and pLV-GATA3-transduced cultures. We found that compared to control transduction, GATA3 overexpression does not significantly alter the percentages of SOX2 and GFAP-expressing cells in 2D (GFAP: 64.9 ± 3.7% in pLV-EGFP versus 66.4 ± 2.6% in pLV-GATA3; SOX2: 2.9 ± 0.7% in pLV-EGFP versus 2.6 ± 0.6% in pLV-GATA3; Figure 3E). Similarly, GATA3 expression did not affect the overall percentage of newborn cells after 8-h BrdU treatment at the third day of the cultures (40.1 ± 1.6% in pLV-EGFP versus 44.2 ± 2.9% in pLV-GATA3) (Figure 3F). GATA3 expression failed to increase the total number of post-mitotic neurons (neurofilament: 3.3 ± 0.9% in pLV-EGFP versus 5.7 ± 1.1% in pLV-GATA3) (Figure 3G), suggesting that in pHA cultures, GATA3 overexpression is not sufficient to affect the progenitor state and neurogenic outcome.

Injuries in the central nervous system compromise the homeostasis and induce a set of events that affect the homeostatic functioning of all cells including the astrocytes. For instance, the dying neurons secrete danger signals that activate the stem cells or various cell types can secrete inflammatory molecules that modulate the neurogenic capacity of the astrocytes (Pekny and Nilsson, 2005; Costa et al., 2010; Liddelow et al., 2017). Therefore, to mimic an injury situation and to determine the effects of GATA3 on pHAs after injury, we performed a diagonal scratch in the wells at 5 days of culture as a surrogate injury model and analyzed the unscratched areas of the wells (Figure 3H). After performing immunocytochemical stainings for BrdU, GFP, TUBB3, GFAP and SOX2 in pLV-EGFP (Figure 3I–K) and pLV-GATA3 cultures (Figure 3L–N), we found that in contrast to unscratched cultures (Figure 2A–G), GATA3 increased the percentage of SOX2-positive neural progenitors (62.2 ± 1.9% in pLV-EGFP versus 79.6 ± 1.0% in pLV-GATA3) but did not affect the percentage of GFAP+ glia, BrdU-positive cells, and percentage of post-mitotic neurons (Figure 3O).

**FIGURE 4:**
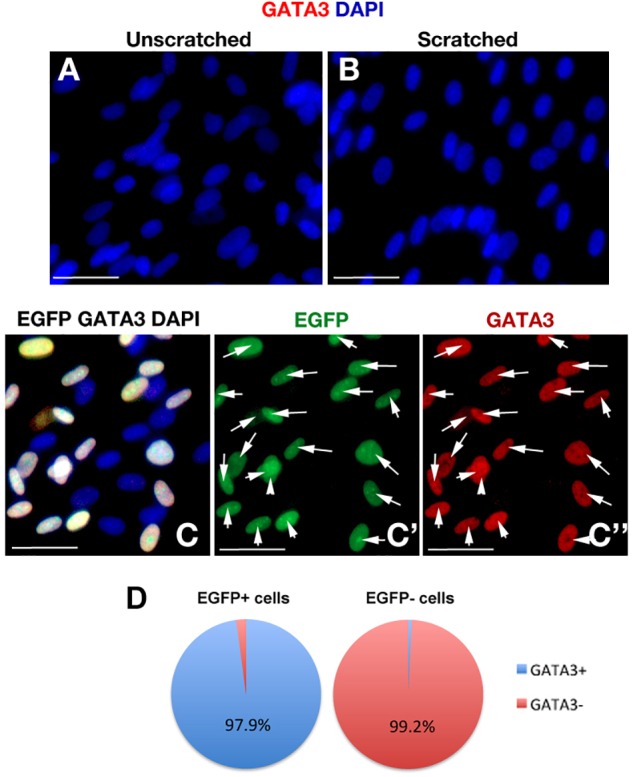
**(A,B)** GATA3 immunocytochemical staining in unscratched **(A)** and scratched **(B)** primary human astrocyte cultures. GATA3 is not induced after scratch. Images were taken at 100 μm distance from the scratch front and its corresponding region in the unscratched sample. **(C)** EGFP and GATA3 immunocytochemical staining in pLV-GATA3-transduced cultures. **(C′)** Individual fluorescent channel for EGFP. **(C”)** Individual fluorescent channel for GATA3. EGFP and GATA3 stainings overlap indicating proportionate stoichiometry of expression from the viral vector. **(D)** Pie chart indicating the percentage of GATA3-positive cells among EGFP-positive and EGFP- cells in pLV-GATA3-transduced cultures. Scale bars 50 μm.

To determine whether the scratch itself would activate GATA3 expression, we performed immunohistochemical staining with GATA3 in scratched and unscratched cultures (Figure 4A,B) and observed that scratch injury does not elicit GATA3 expression. Additionally, to confirm that EGFP and GATA3, which are both expressed by the same viral vector would localize to the same cells, we determined the co-localization of EGFP and GATA3 signals in pLV-GATA3 transduced cultures (Figure 4C–C”). We observed that EGFP and GATA3 expression overlapped in all transduced cells, conforming the stoichiometric expression of both proteins from same lentiviral vector (Figure 4D). This finding also indicated that EGFP expression could be used reliably in substitution for GATA3 expression (i.e., EGFP-positive cells are also GATA3-positive in pLV-GATA3 transduced cultures).

To determine in 2D the effects of GATA3 overexpression in gene expression, we performed whole transcriptome sequencing (Supplementary Figure S1 and Supplementary Data Sheet S1–S4). GATA3 changed the expression of 1,354 genes in unscratched conditions and 1,513 genes in scratched conditions compared to EGFP controls (Supplementary Figure S1 and Supplementary Data Sheet S1, S2). We performed GO-term analyses for the altered genes and pathways (Supplementary Figure S1 and Supplementary Data Sheet S3, S4) and found that pathways related to ECM–cell interaction, neuroactive ligand receptor interaction, cytokine–cytokine receptor interaction and focal adhesion pathways were significantly regulated (Supplementary Figure S1). We also observed that neurogenesis-related pathways such as neuronal projection development, neurogenesis and neuron differentiation were also enriched in our GO-term analyses (Figure 3P), indicating that GATA3 – through regulation of SOX2 expression – would turn on molecular programs that would push astrocytes toward neurogenic fate.

Recently, we have developed a 3D culture system based on starPEG-Heparin hydrogel system using pHAs and found that 3D topology of the cultures dictate a better resemblance of the human neural stem cells and neurons to their *in vivo* counterparts (Papadimitriou et al., 2017a,b, 2018). Given that 3D cultures promote an advanced and more tissue-mimetic physiology of the human brain cells (Murphy et al., 2017), we decided to perform our experiments in our starPEG-Heparin 3D cultures to address whether 3D system would provide better conditions compared to 2D and whether GATA3 has a different effect. By performing gene expression analyses in 3D cultures and comparing to 2D conditions, we found that 3D cultures express a wide variety of genes differently compared to 2D (Supplementary Figure S2 and Supplementary Dataset S5). These genes included neural stem cell markers SOX2, PAX6, MSI1 (Supplementary Figure S2B). Additionally, pathways related to nervous system development, neurogenesis, neuronal maturation, synaptic signaling, axonogenesis or axon guidance are better represented in 3D cultures compared to 2D (Supplementary Figures S2C,D and Supplementary Dataset S5). These results indicate that 3D cultures might be more suitable for studies concerning human neural stem cells.

To determine the gene expression changes exerted by GATA3 in lesion and unlesioned conditions, we cultured pHAs expressing EGFP or GATA3 in 3D starPEG-Heparin hydrogels and performed whole transcriptome analyses (Supplementary Figure S3 and Supplementary Data Sheet S6, S7). We found that increased GATA3 expression in pHAs led to the differential expression of 3,511 genes in unlesioned 3D cultures and 3,472 genes in lesioned 3D cultures (Supplementary Figure S3A). GATA3 expressing samples showed high variation to control samples (average 46% variance) while lesion seems to have minimal effect as the variance between lesioned and unlesioned samples in EGFP and GATA3-expressing cultures did not exceed 5% (Supplementary Figures S3C,D). By generating a heat map of differentially expressed genes in all four conditions, we found that GATA3 exerts lesion-dependent (784 genes differentially expressed in both lesioned and unlesioned case) and lesion-independent (2,689 genes differentially expressed only in lesioned case but not in unlesioned cultures) transcriptional control (Supplementary Figure S3D). By performing GO-term analyses (Supplementary Figure S3E and Supplementary Data Sheet S6, S7), we found that lesion-independent changes were mostly related to nervous system development, cell proliferation and neurogenesis while lesion-dependent changes were mostly related to cell adhesion and synaptic integrity (Supplementary Figure S3F and Supplementary Data Sheet S6, S7). In lesion-independent regulation of GATA3, cell cycle genes, growth factors, chemokines, markers of mature cortical neuronal subtypes and matrix regulating ECM components (e.g., MMPs) were upregulated while connective ECM components (e.g., collagens) were downregulated (Supplementary Figure S3G and Supplementary Data Sheet S6, S7). On the other hand, the genes GATA3 specifically regulated in lesioned 3D conditions included interleukins (Supplementary Figure S3H and Supplementary Data Sheet S6, S7). Interestingly, unlike in 2D cultures (Supplementary Data Sheet S3, S4), GATA3 upregulated the expression of ASCL1 in lesioned 3D hydrogels (Supplementary Figure S3H) suggesting that GATA3 might either promote neurogenesis or would enhance the pro-neurogenic progenitors in 3D cultures.

**FIGURE 5:**
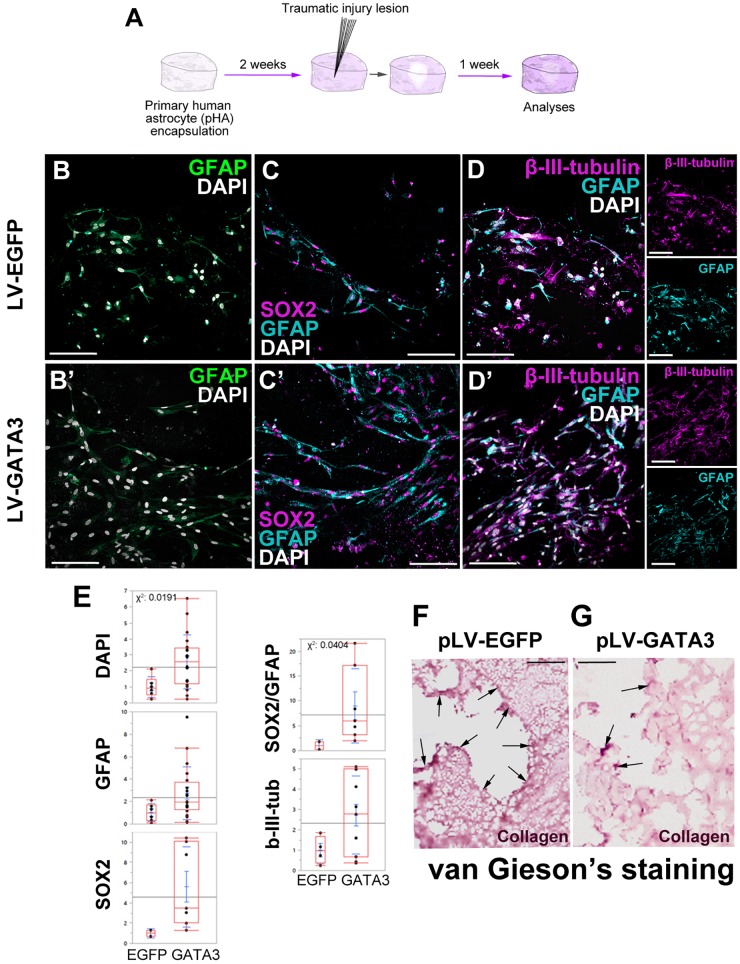
**(A)** Scheme for lesion experiment. **(B,B′)** Immunohistochemistry (IHC) for GFAP in control and GATA3-expressing 3D human astrocyte cultures. **(C,C′)** IHC for SOX2 and GFAP in control and GATA3-expressing 3D human astrocyte cultures. **(D,D′)** IHC for TUBB3 and GFAP in control and GATA3-expressing 3D human astrocyte cultures. Smaller panels to the right show individual fluorescence channels for TUBB3 and GFAP. **(E)** Quantification graphs. **(F,G)** van Gieson’s staining for collagen deposition and connective tissue in pLV-EGFP-transduced lesioned samples **(F)** and pLV-GATA3-transduced lesioned samples. Arrows indicate dense collagen depositions, which are more numerous in EGFP-transduced samples. On average more than 100 cells were counted per stack (upper limit 608 cells). Scale bars: 100 μm.

To investigate whether GATA3 expression would enhance neurogenic progenitor cells and neurogenesis in 3D cultures, we performed immunohistochemical stainings in lesioned control and GATA3-expressing pHAs in 3D cultures (Figure 5A–D). By performing quantifications for total number of nuclei, GFAP-positive, SOX2-positive, GFAP/SOX2-positive, and TUBB3-positive cells around the lesion site (within a distance of 150 um from the lesion front) (Figure 5E), we found that the number of total cells are higher in GATA3-positive cultures and the number of GFAP/SOX2 double positive astrocytes are elevated in lesioned conditions (Figure 5E). These results were consistent when the analyzed region was extended to 300 micrometers from the lesion front (data not shown). We also observed no significant difference in total cell death between control and GATA3-positive cultures (TUNEL-positive nuclei in cultures, data not shown).

Traumatic injuries in mammalian brains lead to formation of a permanent scar tissue, which is rich in collagen deposition and is impermeable to neuronal projections (Fawcett and Asher, 1999; Silver and Miller, 2004; Fitch and Silver, 2008; Rolls et al., 2009; Sofroniew, 2009). To determine if in our 3D culture system injuries would cause a scar-like tissue, we performed van Gieson staining to determine the collagen deposition (Figure 5F,G). We observed that compared to control lesions where collagen deposition is apparent at the lesion site (Figure 5F), GATA3-transduced cultures reduced the collagen deposition significantly (Figure 5G). This observation is consistent with the effects of GATA3 on the expression of factors related to remodeling of the extracellular matrix (Figure 3P), and suggests that GATA3 might have an ameliorating effect on the scar formation. Therefore, these results indicate that GATA3 alleviates the scar-like collagen deposition, promotes the neural progenitor state (GFAP/SOX2 co-expressing astrocytes) while does not affect the neurogenic outcome after traumatic injury paradigms in human cortical astrocytes cultures.

## Discussion

Astrocytes in mammalian brains exist in various forms such as neural stem cells or parenchymal astrocytes (Doetsch et al., 1999; Johansson et al., 1999; Seri et al., 2001; Doetsch, 2003; Pekny and Nilsson, 2005; Costa et al., 2010; Jebelli et al., 2015). These cell types could serve as endogenous reservoir for new neurons as they are known to have neurogenic properties *in vivo* and *in vitro* (Laywell et al., 2000; Doetsch, 2003; Gage and Temple, 2013). However, after injures or neurodegenerative diseases, mostly the parenchymal astrocytes proliferate and form scar-like tissues around the lesion sites, and they are rather non-neurogenic (Doetsch and Scharff, 2001; Kriegstein and Alvarez-Buylla, 2009; Costa et al., 2010; Heinrich et al., 2010; Robel et al., 2011; Codega et al., 2014). Therefore, investigating the molecular programs by which these cells can be enhanced in their neurogenic potential could be important for regenerative medical applications. Given that several seminal studies succeeded in converting non-neurogenic cortical astrocytes in mouse to neurons by reprogramming (Heinrich et al., 2010; Karow et al., 2012; Chen et al., 2015; Masserdotti et al., 2015; Gascon et al., 2016), enhancing neurogenic potential in human brains using endogenous reservoir cells stands as a plausible future goal.

In our study, we investigated the effect of overexpression of human GATA3 in pHAs in 2D and 3D culture conditions with or without injury because Gata3 is associated with regenerative neurogenic potential of glial cells in adult zebrafish brain (Kizil et al., 2012b), and its mechanism of action is specific to the neurogenic potential of neural stem/progenitor cells after the injury (Kizil et al., 2012b; Kyritsis et al., 2012; Rodriguez Viales et al., 2015; Katz et al., 2016). In zebrafish, Gata3 expression is significantly upregulated after injury, and this leads to an induced neurogenesis response (Kizil et al., 2012b); however, in pHAs GATA3 is not induced with injury (Figure 4 and Supplementary Figure S4). This discrepancy could point toward a biological difference in the capacity of mammalian astrocytes and zebrafish glial cells to activate GATA3. At this point, we cannot provide a definitive answer to this hypothesis, as primary cultures of astrocytes cannot mimic the mammalian brain in full because the culture systems lack various essential cell types such as the immune cells or endothelial compartment. However, despite the reductionist nature of culture systems, we also did not observe increased neurogenesis from pHAs after artificially activating GATA3 expression in 2D and 3D cultures. Here, we also emphasize that culture systems cannot manifest a full spectrum of the brain physiology and in our system, essential contributors that might help GATA3-positive cells to turn into neurons might be absent. Therefore, the failure of GATA3 treatment in promoting neurogenesis in the primary astrocyte culture must be further investigated in an *in vivo* brain model. Yet, it is known that rodent and human brains are physiologically different and rodent brains can also recapitulate human brain physiology only to a certain extent (Urban and Guillemot, 2014; Choi et al., 2016; Qiu et al., 2016; Espuny-Camacho et al., 2017). Therefore, since traumatic injury experiments cannot be performed in human brains, our 3D model system can be used as a surrogate to address questions on human astrocytes and their response to injury. Starting with primary human cortical astrocytes, our culture systems can increase the number of neurons, demonstrating that 3D cultures can be used as a reductionist tool for assessing the neurogenic properties of human cortical astrocytes. In our previous model of Alzheimer’s disease (Papadimitriou et al., 2018), reduced neurogenic response could be restored by using interleukin-4 as a stimulant of neurogenic competency and neurogenesis in primary human cortical astrocytes. In our current traumatic lesion model, although GATA3 failed to enhance neurogenesis to a considerable degree, we did find an increase in the extent of the neural progenitors and reduction in the collagen deposition around the lesion site. Future studies with factors that are known to be enhancing neurogenesis could serve as benchmark studies to assess the robustness of our culture conditions and system and its resemblance to human brains. Additionally, co-culture studies with immune cells would be instrumental to assess the role of immune system in neurogenic potential of astrocytes. In overall, given that no culture system can fully recapitulate the *in vivo* conditions, we believe that our 3D culture system provides a reductionist but realistic model to assess the cell intrinsic neurogenic potential of pHAs.

A consideration why GATA3 would not enhance neurogenesis in our culture system could be due to the nature of the astrocytes. If primary astrocytes would lose their neurogenic capacity during the time period of the culture, GATA3 would not be sufficient to activate neurogenesis. We believe that this is not the case because in our previous studies where we block neurogenesis using pathological hallmarks of Alzheimer’s disease and Kynurenic acid, interleukin-4 was sufficient to restore the neurogenic capacity at the later time points of the culture (Papadimitriou et al., 2018). Furthermore, when we determined the growth curve and neurogenesis dynamics of the culture using EdU and BrdU, we found continuous neurogenesis during the culture period in 2D and 3D (Papadimitriou et al., 2018). These results indicate that primary astrocytes still bear neurogenic ability during the entire culture period, however, GATA3 is not sufficient to drive the astrocytes to neurogenesis alone.

Neurogenesis is a lengthy process and depending on the time points after any treatment, the manifestation of neurogenic output may vary in time. In our culture system, we believe that the time points (1 week after lesion in 2D and 3D cultures) is sufficient to manifest neurogenic outcome because based on our neurogenesis assay we used to characterize the 3D cultures (Papadimitriou et al., 2018), 1 week was sufficient to observe significant amount of new neurons. Additionally, in various studies, 1 week was shown to be a reasonable time for neurogenesis to occur in both *in vitro* and *in vivo* (Lim et al., 2000; Heins et al., 2002; Costa et al., 2010; Heinrich et al., 2010; Pozniak et al., 2010; Curto et al., 2014; Kotterman et al., 2015). Therefore, we believe that the inability of GATA3 to promote neurogenesis in our culture systems points toward a biological phenomenon rather than a technical hurdle.

Neurogenic cascade from astrocytes follow a well-defined path where GFAP-positive astrocytes lose this marker expression while differentiating into neurons, which start expressing early neuronal markers such as beta-III-tubulin and later neuronal markers such as neurofilament (Doetsch and Scharff, 2001; Seri et al., 2001; Alvarez-Buylla et al., 2002; Heins et al., 2002; Phillips et al., 2006; Chapouton and Godinho, 2010; Bonfanti and Peretto, 2011). In culture conditions, it is often observed that there is a temporal overlap between GFAP and beta-III-tubulin expression (Draberova et al., 2008). This overlap is hardly seen *in vivo* but frequent *in vitro*. In our culture system, we do see such an overlap in a portion of GFAP cells (Figure 1, 5). In addition to GFAP/beta-III-tubulin double-positive cells, we also see cells expressing only GFAP or beta-III-tubulin (Figure 1, 5), indicating that presence of GFAP and beta-III-tubulin might mark a transient stage where astrocytes are differentiating into neurons or turn on neurogenesis programs. Additionally, GFAP/beta-III-tubulin-double positive cells also display morphologies that are reminiscent of astrocytes, and when these cells lose GFAP expression, they tend to get more elongated and acquire neuronal morphologies (Figure 1, 3) (Papadimitriou et al., 2018). These results suggest that a transient stage of neuronal differentiation might be more pronounced in *in vitro* cultures and in our culture systems, this stage can be quantified using GFAP and beta-III-tubulin immunocytochemical detection methods. We believe that this stage is important because it cannot be determined by using other mature neuronal markers such as neurofilament, which marks only mature neurons that start expressing neurofilament long after the astrocytes lose GFAP immunoreactivity.

Astrocytes are also important players for the scar tissue, which manifests prominently in mammalian brains after injury (Fawcett and Asher, 1999; Silver and Miller, 2004; Sofroniew, 2009; Jones and Bouvier, 2014). The contribution of reactive astrocytes to scar tissue is an important aspect of neural regeneration because if these cells could be coaxed to become neurogenic rather than scar-forming, a restorative neurogenesis could be made possible (Stevens et al., 2007; Aguzzi et al., 2013; Liddelow et al., 2017). Given that reactive astrocytes derive from subventricular zone neural stem cells (Faiz et al., 2015), it is a plausible hypothesis that reactive astrogliosis could be nudged to neurogenesis with appropriate neurogenic factors. We found that GATA3 expression in pHAs after lesion significantly reduced the collagen deposition in the lesioned area (Figure 5F,G). This observation is consistent with our findings with transcriptional profiling (Figure 4 and Supplementary Dataset S6, S7) where extracellular matrix reorganization is enhanced by the presence of GATA3. This finding is interesting as it suggests that GATA3 is sufficient to alleviate the scar-like ECM deposition in lesioned brains and might generate a permissive environment for new neurons to form from reactive astrocytes. Since astrocytes function to contain the lesion site for preventing secondary damage but are unable to resolve the gliotic response later on, the effects of GATA3 in reducing the ECM deposition around the lesion site concomitant to enhancing the potential of neural progenitors might provide a basis for how post-traumatic lesion conditions could be gradually alleviated to allow neural progenitors to survive and form new neurons that integrate into the lesioned areas in the absence of non-permissive scar tissue. Further studies *in vivo* in mammalian brains would shed more light onto this aspect and can provide a new experimental model that is clinically relevant to traumatic injuries.

Several reports successfully demonstrated the conversion of primary astrocytes to neurogenic fate (Heins et al., 2002; Buffo et al., 2005; Brill et al., 2009; Costa et al., 2010; Robel et al., 2011; Karow et al., 2012; Lujan et al., 2012; Niu et al., 2013, 2015; Chen et al., 2015, 2017; Brulet et al., 2017) indicating that human astrocytes can be harnessed for neurogenic outcome. This route offers enormous clinical ramifications and identifying relevant factors that are capable of such neurogenic instructiveness is an important research realm. Based on our findings that lentivirus-mediated overexpression of GATA3 can significantly increase the amount of GATA3+ cells (Figure 2 and Supplementary Figure S4) and this is accompanied by increased neural progenitors and neurogenic potential, we hypothesize that the lack of upregulation of GATA3 expression by pHAs after injury could be an underlying cause why mammalian astrocytes cannot manifest neurogenic capacity under injury context. Experiments such as targeted overexpression of GATA3 in specific cells of mammalian cortical astrocytes *in vivo* and investigation of neurogenic potential after injury could be informative in testing this hypothesis in *in vivo* disease settings such as traumatic injuries or neurodegeneration.

## Author Contributions

HC: collection of data, data analysis, and interpretation. MIC: sequencing, collection of data, analysis and interpretation. SP: generation of lentivirus, viral transduction. CP, PB, VM, TS, SW, SNB, INA, LN, and KB: collection of data. UF and CW: provision of study material. CK: conception and design, supervision of the project, financial support, assembly of data, data analysis and interpretation, writing and editing the manuscript.

## Conflict of Interest Statement

The authors declare that the research was conducted in the absence of any commercial or financial relationships that could be construed as a potential conflict of interest.

## Footnotes

1 http://www.genecopoeia.com

## Supplementary Material

FIGURE S1 **(A)** Experimental scheme for whole transcriptome sequencing in 2D cultures. **(B)** Sample correlation heat map. **(C)** Principal component analyses. **(D)** Differential expression intersection sets. **(E)** MA plots. **(F)** Read number heat maps. **(G)** Differential expression fold change heat maps. **(H)** Log fold change comparison. **(I)** KEGG pathway comparison for top5 hits. **(J)** Biological process analyses for differentially expressed genes. **(K)** GO-stat KEGG pathway analysis pie chart and depiction of selected pathways. Red, upregulation; green, downregulation.

FIGURE S2 **(A)** MA-plot for comparison between 3D and 2D cultures. **(B)** Heat map for a set of genes selected from MA plot. Red, upregulation; green, downregulation. **(C)** KEGG enrichment analyses in 3D versus 2D cultures. **(D)** Exemplary KEGG pathway representation for axon guidance. Red: upregulation, green: downregulation.

FIGURE S3 **(A)** Schematic view of comparisons and MA plots for gene expression changes after GATA3 expression in lesioned (LP) and unlesioned (LN) conditions in 3D. **(B)** Principal component analyses for variance. **(C)** Sample clustering. **(D)** Heat map for gene expression changes. **(E)** GO-term and KEGG charts for top10 hits in lesion-independent regulation by GATA3. **(F)** GO-term and KEGG charts for top10 hits in lesion-dependent regulation by GATA3. **(G)** Heat map for selected genes in lesion-independent regulation by GATA3. **(H)** Heat map for selected genes in lesion-dependent regulation by GATA3.

FIGURE S4 Quantification graphs for GFP/GFAP, GFP/neurofilament, GFP/SOX2, and GFP/BrdU double positive cells. UE, EGFP-expressing unscratched pHAs; UG, GATA3-expressing unscratched pHAs; SE, EGFP-expressing scratched pHAs; SG, GATA3-expressing scratched pHAs. ^∗^*p* < 0.05; ^∗∗^*p* < 0.01, ^∗∗∗^*p* < 0.005.

DATASET S1 List of differential expression genes in primary human astrocytes (pHAs) in 2D cultures. **(A)** GATA3-expressing and scratched pHAs versus GATA3-expressing and unscratched pHAs. **(B)** GATA3-expressing and scratched versus EGFP-expressing and scratched pHAs. **(C)** EGFP-expressing and scratched versus EGFP-expressing and unscratched pHAs. **(D)** GATA3-expressing and unscratched versus EGFP-expressing and unscratched pHAs.

DATASET S2 Heat maps of differential expression in 2D cultures of pHAs. **(A)** Log fold changes. **(B)** Normalized read numbers.

DATASET S3 GO-term analyses of GATA3-expressing and unscratched pHAs versus EGFP-expressing and unscratched pHAs in 2D cultures.

DATASET S4 GO-term analyses for GATA3-expressing and scratched pHAs versus EGFP-expressing and scratched pHAs in 2D cultures.

DATASET S5 GO-term analyses of control cultures (EGFP-expressing and no injury) in 3D versus 2D.

DATASET S6 GO-term analyses of GATA3-expressing versus EGFP-expressing unlesioned pHAs in 3D.

DATASET S7 GO-term analyses of GATA3-expressing versus EGFP-expressing lesioned pHAs in 3D.
