# Supplementary figures and images for: GATA3 Promotes the Neural Progenitor State but Not Neurogenesis in 3D Traumatic Injury Model of Primary Human Cortical Astrocytes (part 1 of 3)
Source: Front Cell Neurosci. 2019 Feb 11;13:23. doi: 10.3389/fncel.2019.00023 (PMC6380212; doi:10.3389/fncel.2019.00023)

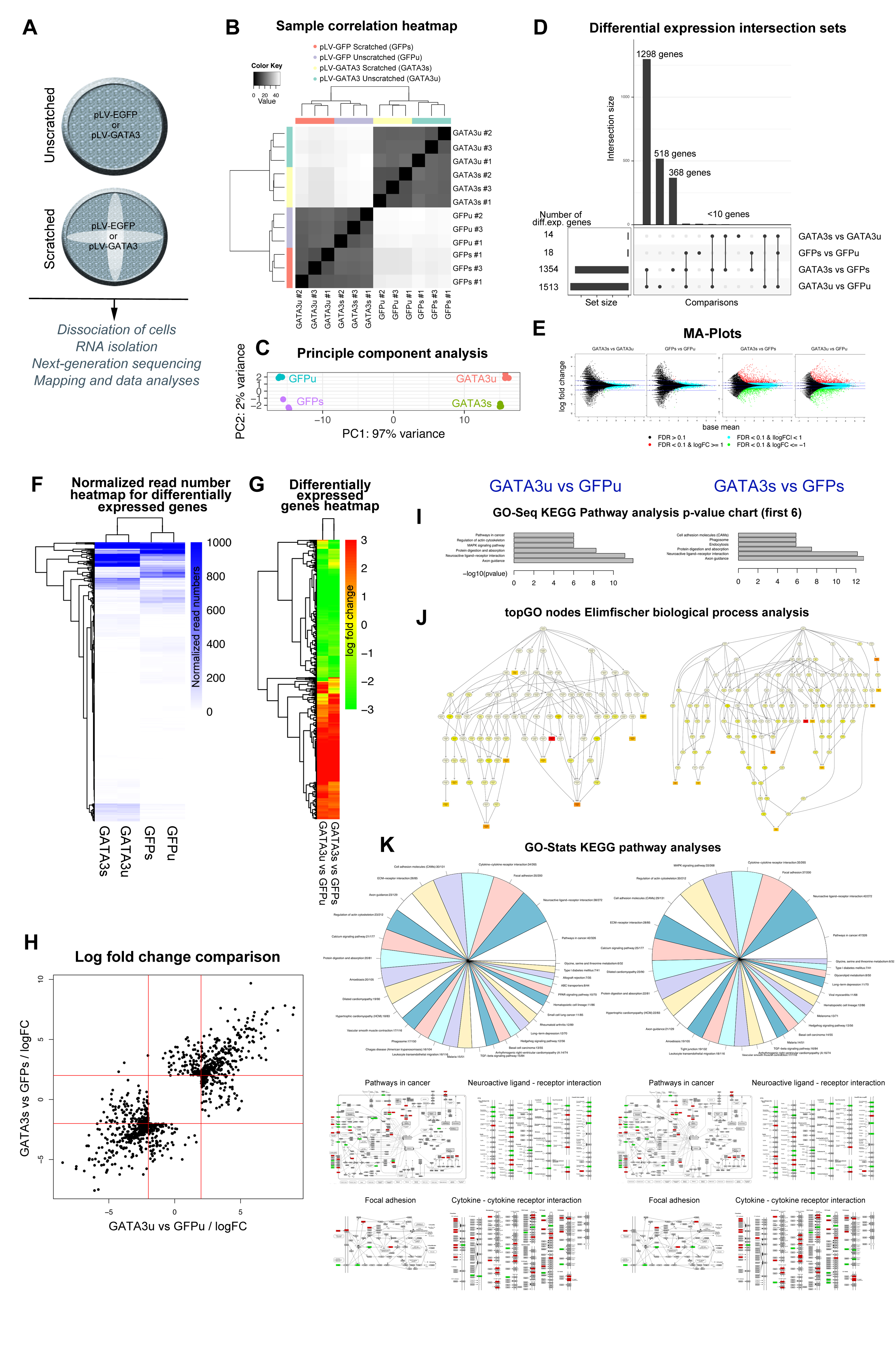

Supplement: FIGURE S1 — (A) Experimental scheme for whole transcriptome sequencing in 2D cultures. (B) Sample correlation heat map. (C) Principal component analyses. (D) Differential expression intersection sets. (E) MA plots. (F) Read number heat maps. (G) Differential expression fold change heat maps. (H) Log fold change comparison. (I) KEGG pathway comparison for top5 hits. (J) Biological process analyses for differentially expressed genes. (K) GO-stat KEGG pathway analysis pie chart and depiction of selected pathways. Red, upregulation; green, downregulation. [file Image_1.JPEG]

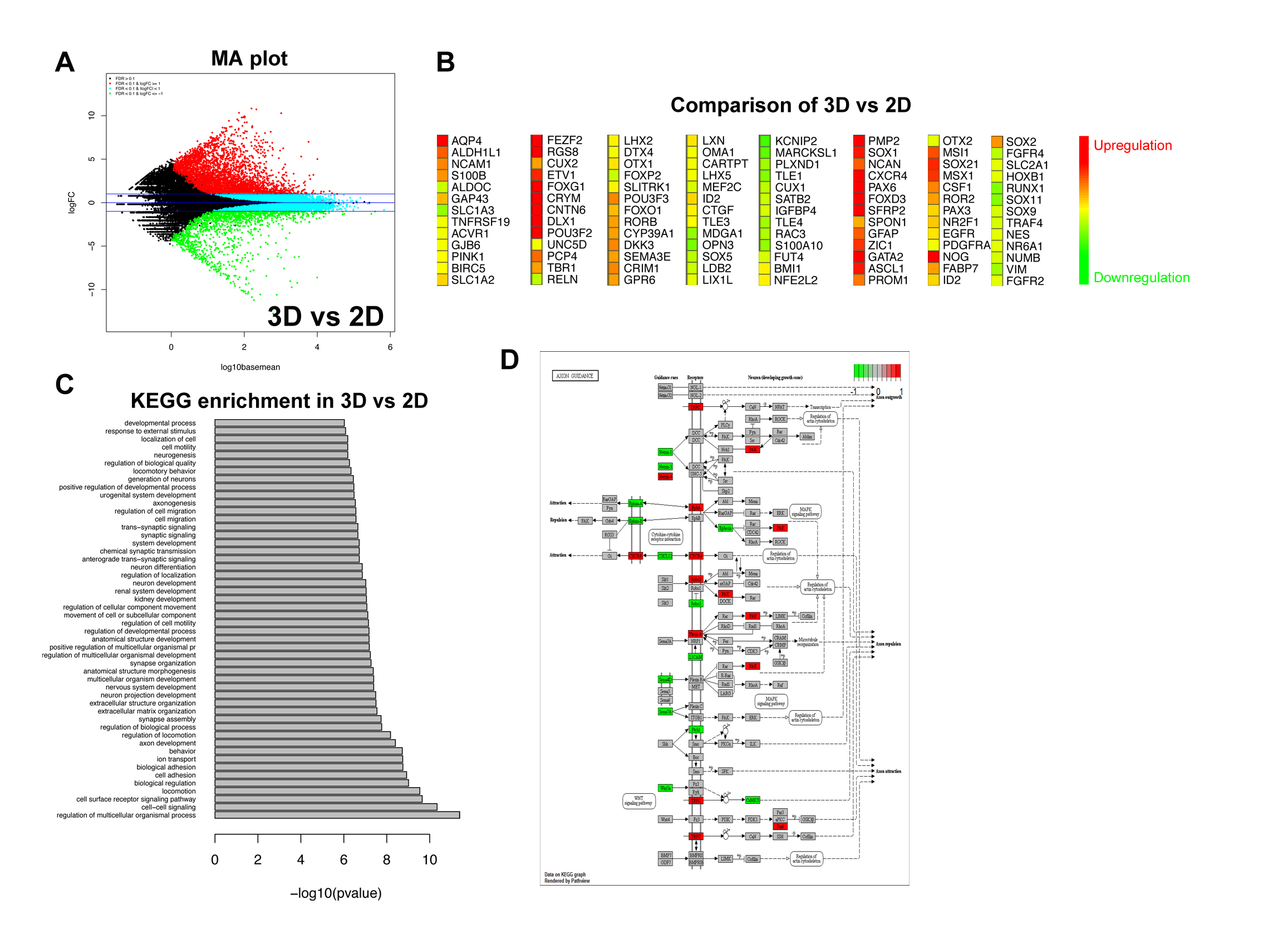

Supplement: FIGURE S2 — (A) MA-plot for comparison between 3D and 2D cultures. (B) Heat map for a set of genes selected from MA plot. Red, upregulation; green, downregulation. (C) KEGG enrichment analyses in 3D versus 2D cultures. (D) Exemplary KEGG pathway representation for axon guidance. Red: upregulation, green: downregulation. [file Image_2.JPEG]

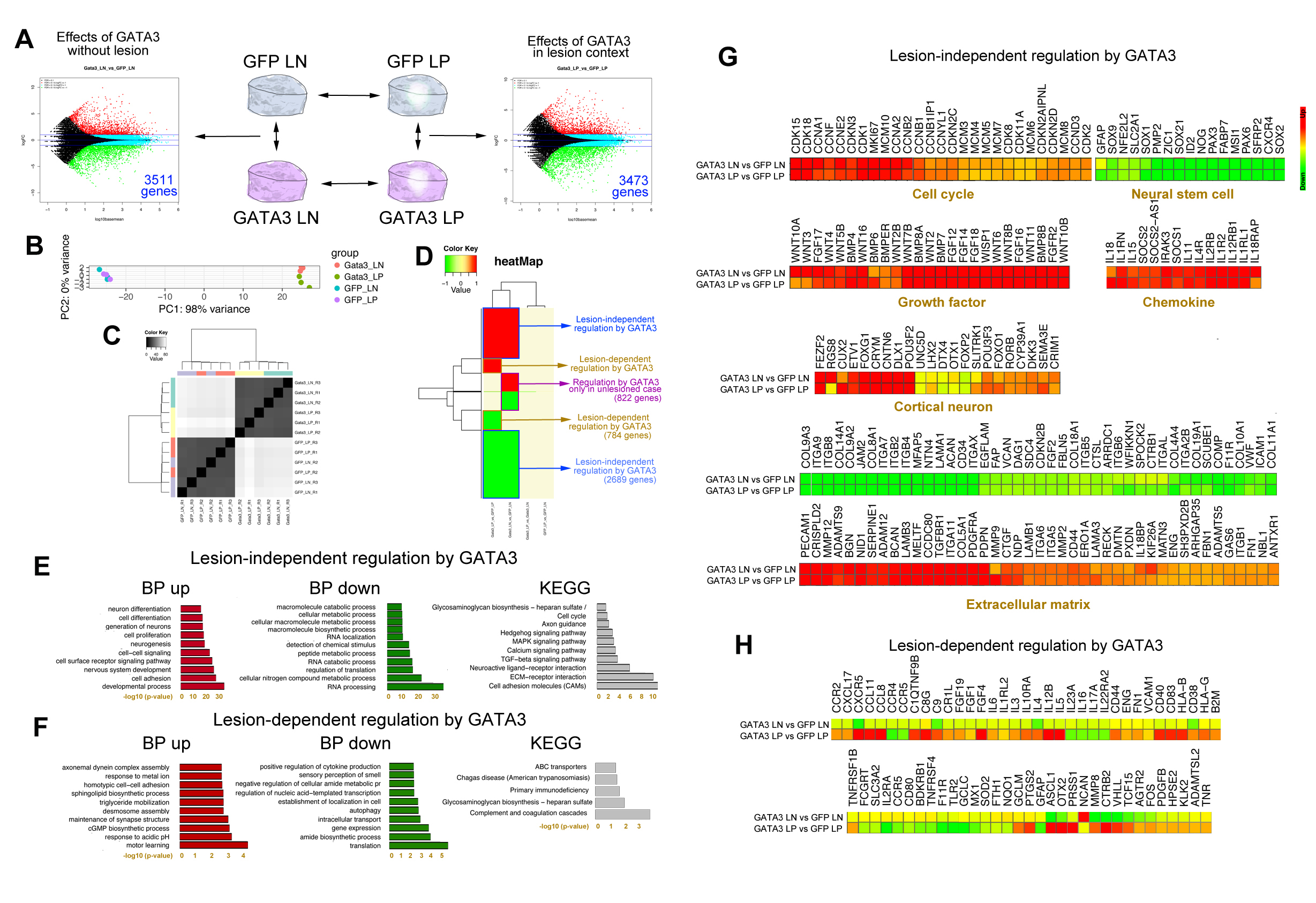

Supplement: FIGURE S3 — (A) Schematic view of comparisons and MA plots for gene expression changes after GATA3 expression in lesioned (LP) and unlesioned (LN) conditions in 3D. (B) Principal component analyses for variance. (C) Sample clustering. (D) Heat map for gene expression changes. (E) GO-term and KEGG charts for top10 hits in lesion-independent regulation by GATA3. (F) GO-term and KEGG charts for top10 hits in lesion-dependent regulation by GATA3. (G) Heat map for selected genes in lesion-independent regulation by GATA3. (H) Heat map for selected genes in lesion-dependent regulation by GATA3. [file Image_3.JPEG]

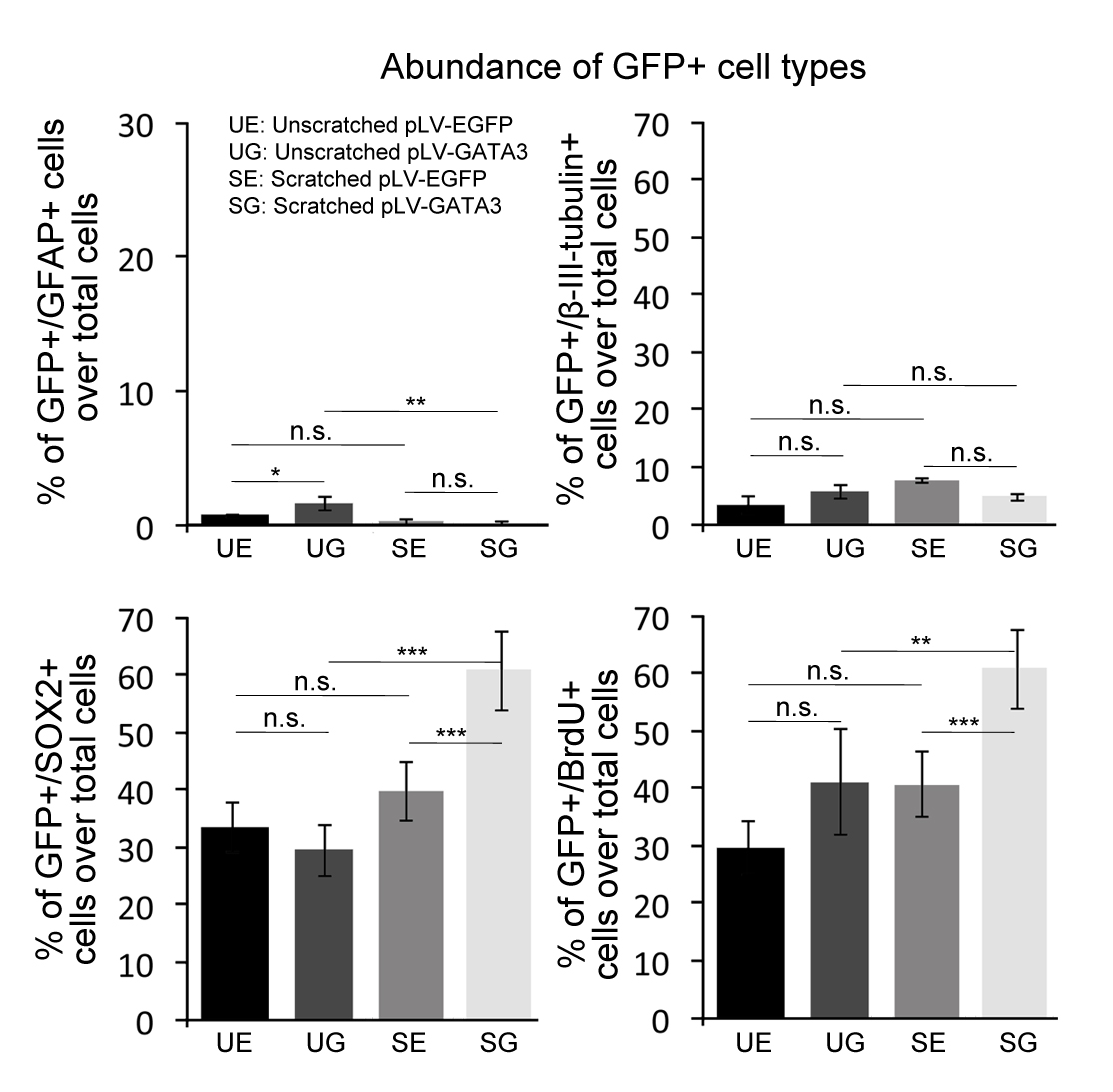

Supplement: FIGURE S4 — Quantification graphs for GFP/GFAP, GFP/neurofilament, GFP/SOX2, and GFP/BrdU double positive cells. UE, EGFP-expressing unscratched pHAs; UG, GATA3-expressing unscratched pHAs; SE, EGFP-expressing scratched pHAs; SG, GATA3-expressing scratched pHAs. ∗p < 0.05; ∗∗p < 0.01, ∗∗∗p < 0.005. [file Image_4.JPEG]

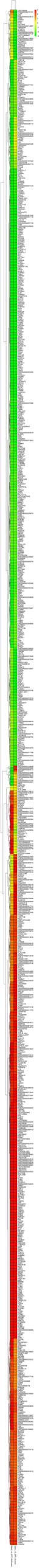

Supplement: DATASET S2 — Heat maps of differential expression in 2D cultures of pHAs. (A) Log fold changes. (B) Normalized read numbers. [file Data_Sheet_2.ZIP › Heatmap_log2FoldChanges_HighResolution.pdf]

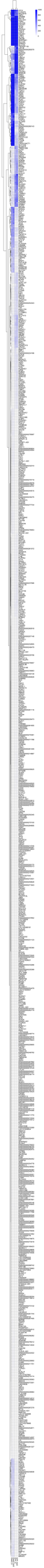

Supplement: DATASET S2 — Heat maps of differential expression in 2D cultures of pHAs. (A) Log fold changes. (B) Normalized read numbers. [file Data_Sheet_2.ZIP › Heatmap_normalized_gene_expression_HighResolution.pdf]

CP\_enrichGO\_BP

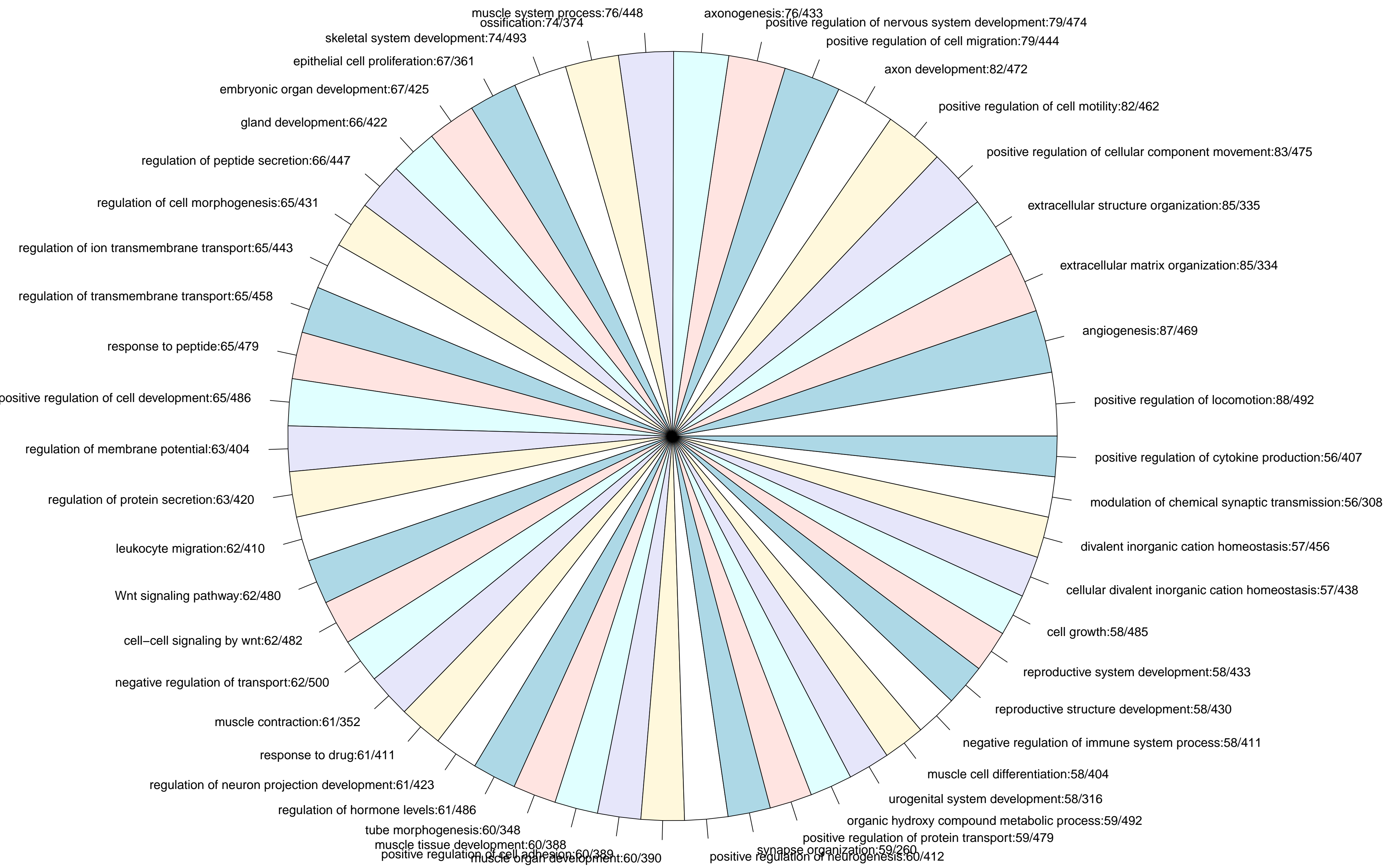

Supplement: DATASET S3 — GO-term analyses of GATA3-expressing and unscratched pHAs versus EGFP-expressing and unscratched pHAs in 2D cultures. [file Data_Sheet_3.ZIP › GO_term_analyses_GATA3u_vs_GFPu/clusterProfiler/CP_enrichGO_BP.pdf]

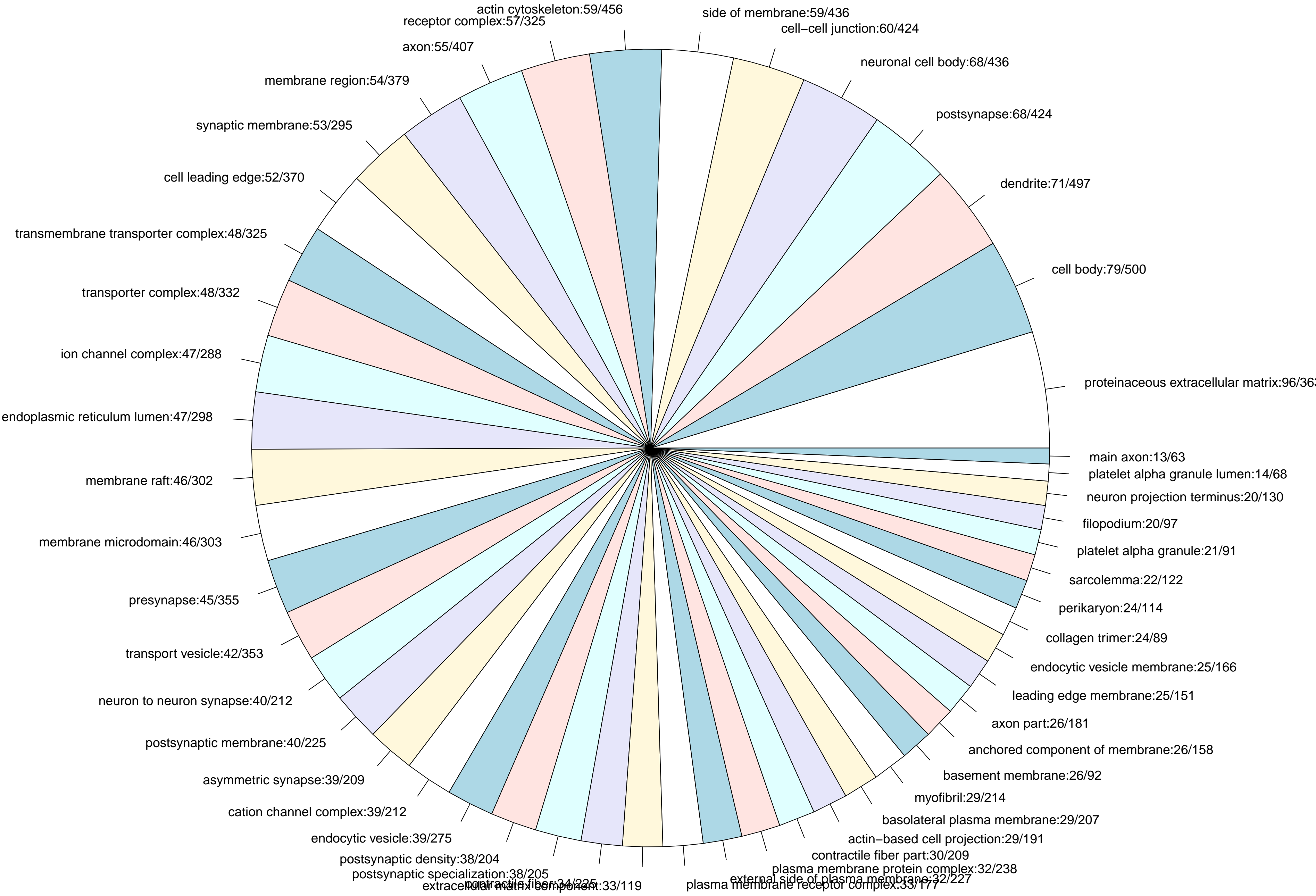

Supplement: DATASET S3 — GO-term analyses of GATA3-expressing and unscratched pHAs versus EGFP-expressing and unscratched pHAs in 2D cultures. [file Data_Sheet_3.ZIP › GO_term_analyses_GATA3u_vs_GFPu/clusterProfiler/CP_enrichGO_CC.pdf]

CP\_enrichGO\_MF

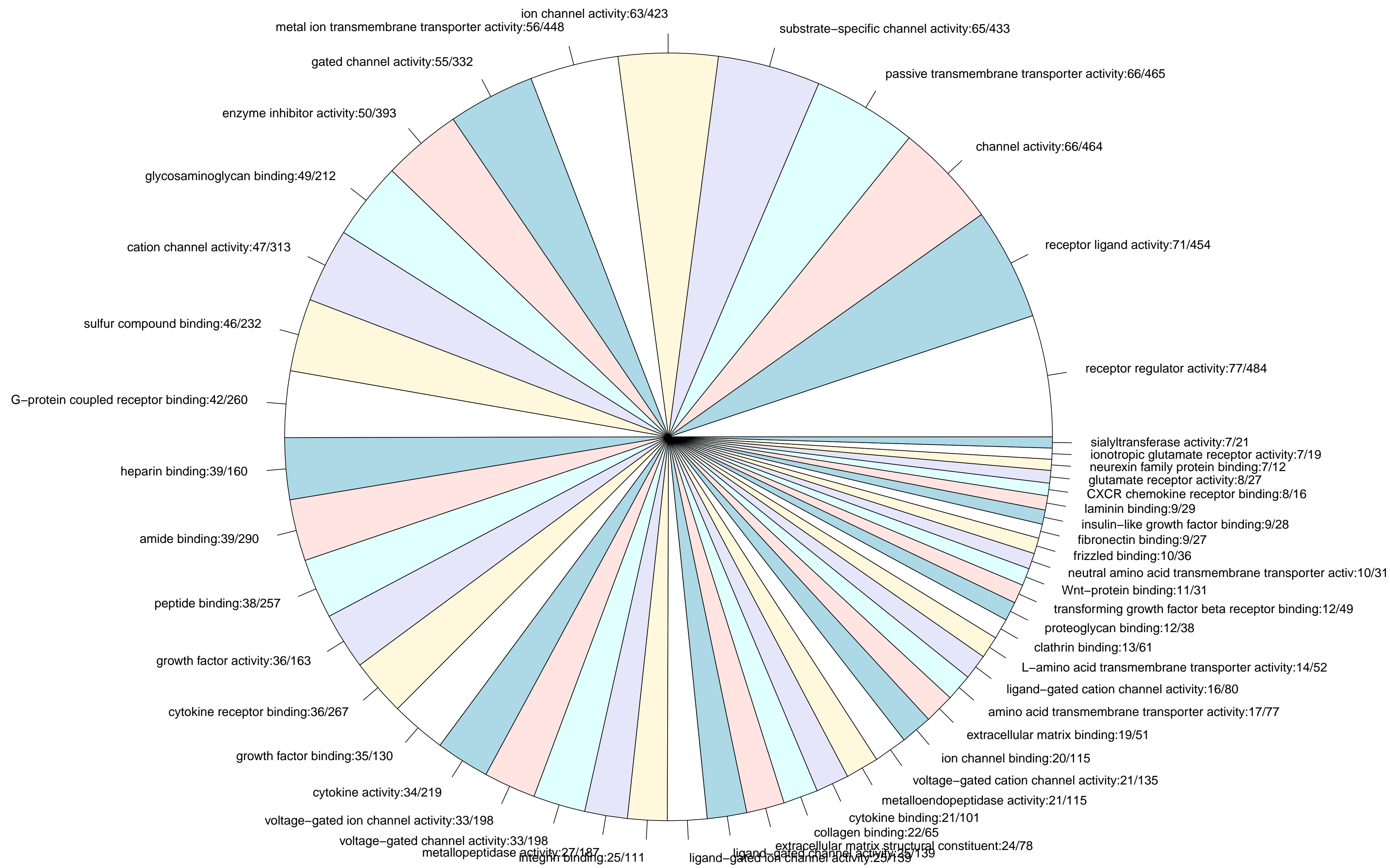

Supplement: DATASET S3 — GO-term analyses of GATA3-expressing and unscratched pHAs versus EGFP-expressing and unscratched pHAs in 2D cultures. [file Data_Sheet_3.ZIP › GO_term_analyses_GATA3u_vs_GFPu/clusterProfiler/CP_enrichGO_MF.pdf]

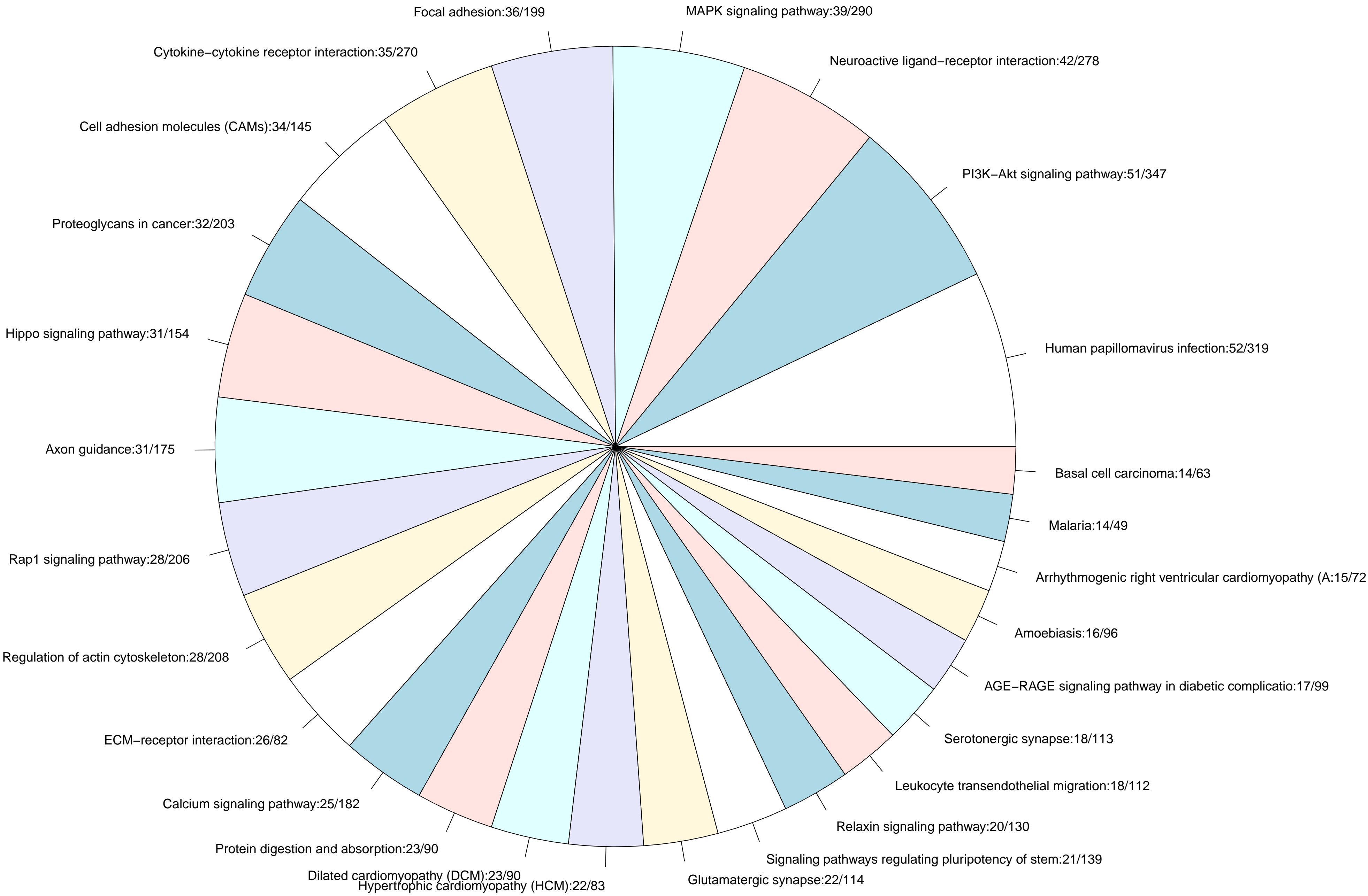

Supplement: DATASET S3 — GO-term analyses of GATA3-expressing and unscratched pHAs versus EGFP-expressing and unscratched pHAs in 2D cultures. [file Data_Sheet_3.ZIP › GO_term_analyses_GATA3u_vs_GFPu/clusterProfiler/CP_enrichKEGGS.pdf]

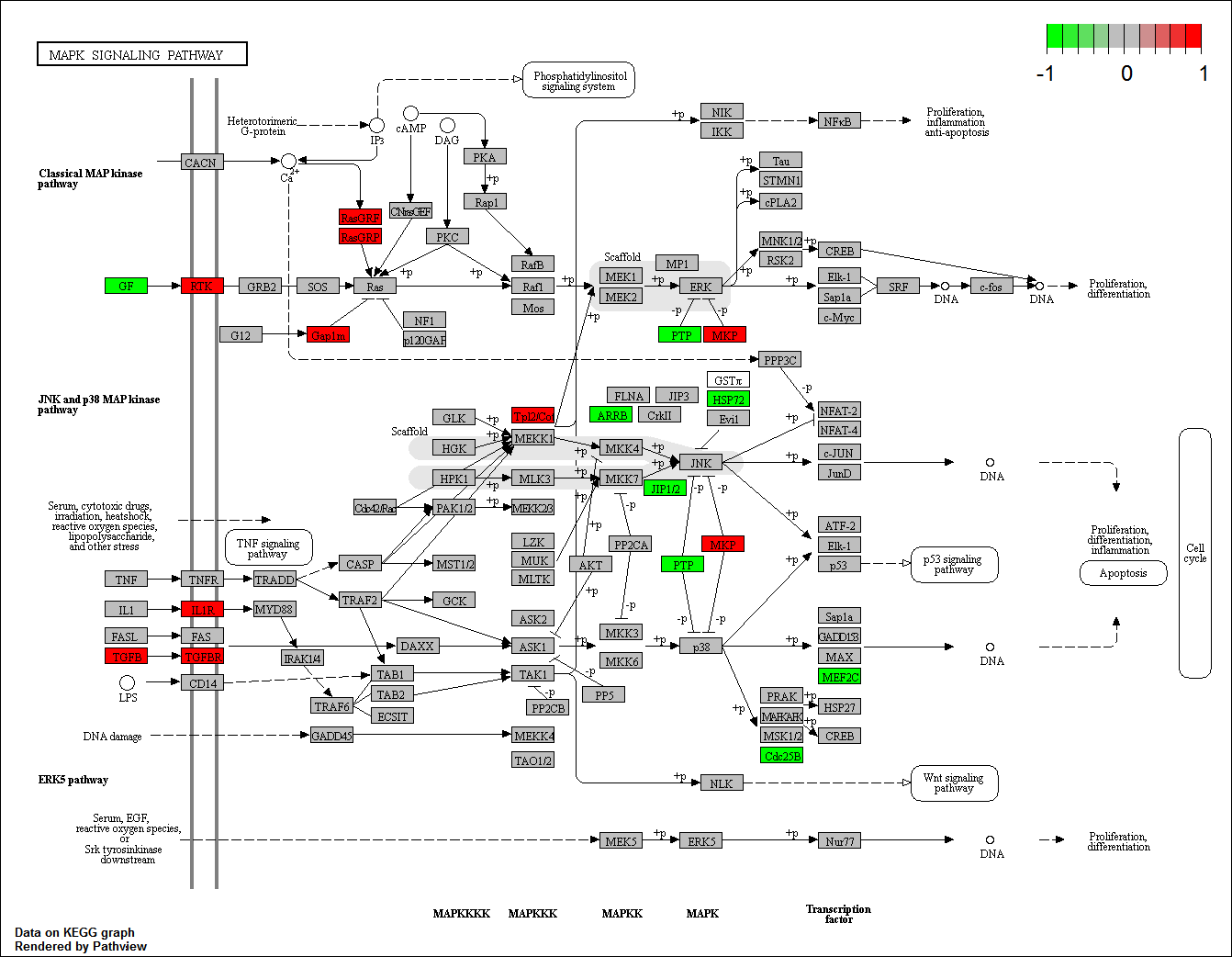

Supplement: DATASET S3 — GO-term analyses of GATA3-expressing and unscratched pHAs versus EGFP-expressing and unscratched pHAs in 2D cultures. [file Data_Sheet_3.ZIP › GO_term_analyses_GATA3u_vs_GFPu/clusterProfiler/hsa04010.MAPKsignalingpathway.png]

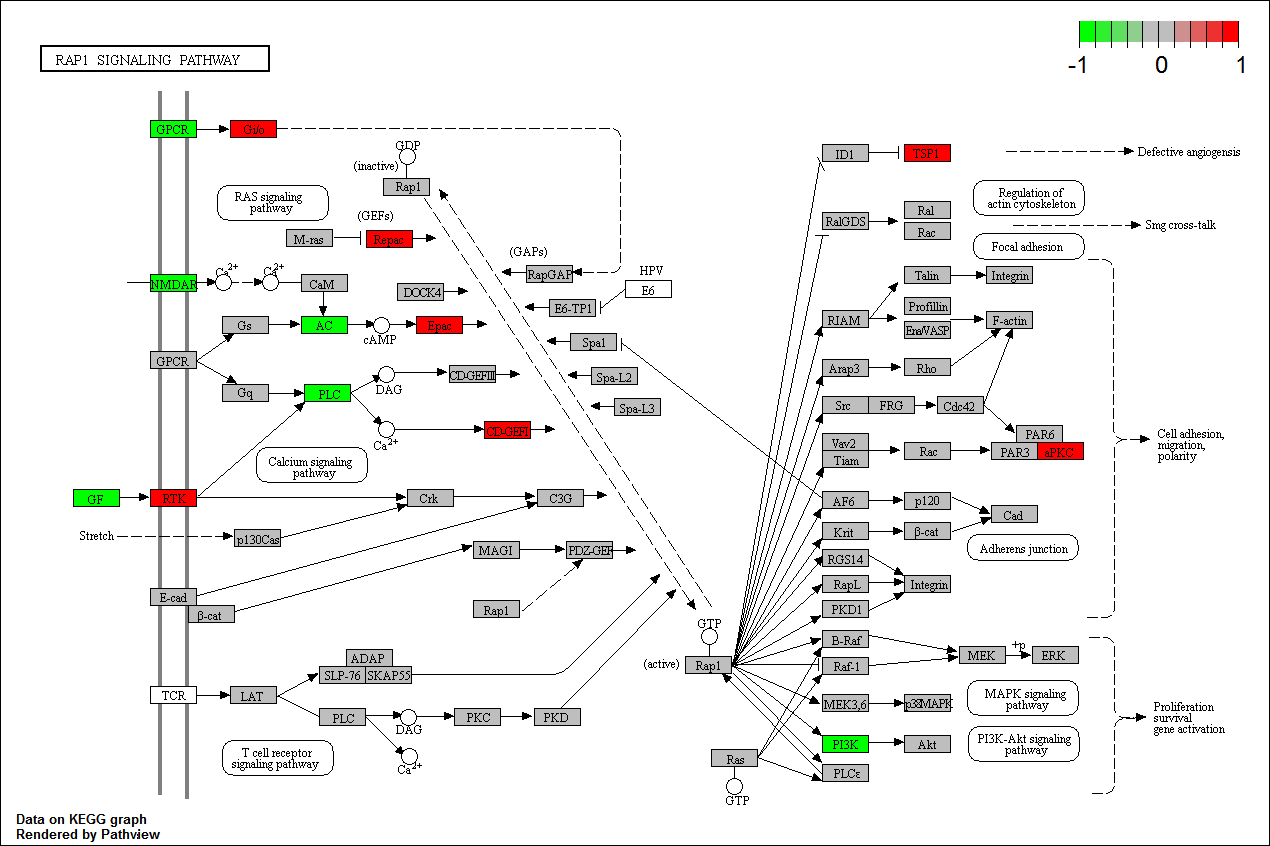

Supplement: DATASET S3 — GO-term analyses of GATA3-expressing and unscratched pHAs versus EGFP-expressing and unscratched pHAs in 2D cultures. [file Data_Sheet_3.ZIP › GO_term_analyses_GATA3u_vs_GFPu/clusterProfiler/hsa04015.Rap1signalingpathway.png]

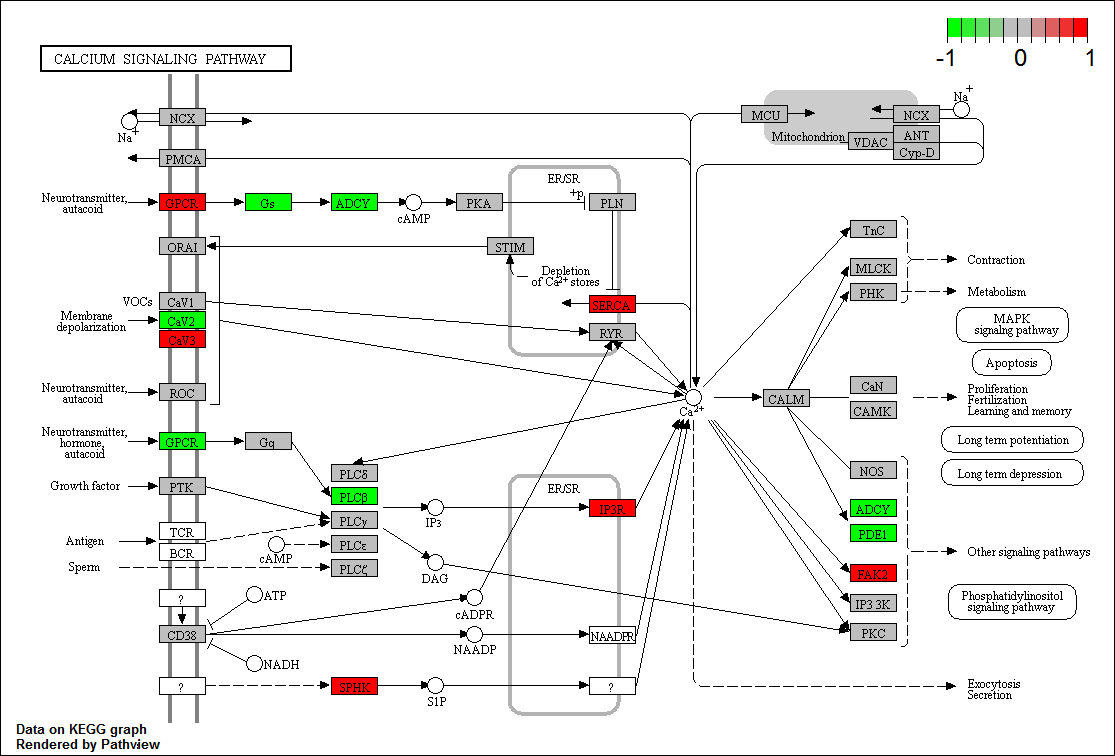

Supplement: DATASET S3 — GO-term analyses of GATA3-expressing and unscratched pHAs versus EGFP-expressing and unscratched pHAs in 2D cultures. [file Data_Sheet_3.ZIP › GO_term_analyses_GATA3u_vs_GFPu/clusterProfiler/hsa04020.Calciumsignalingpathway.png]

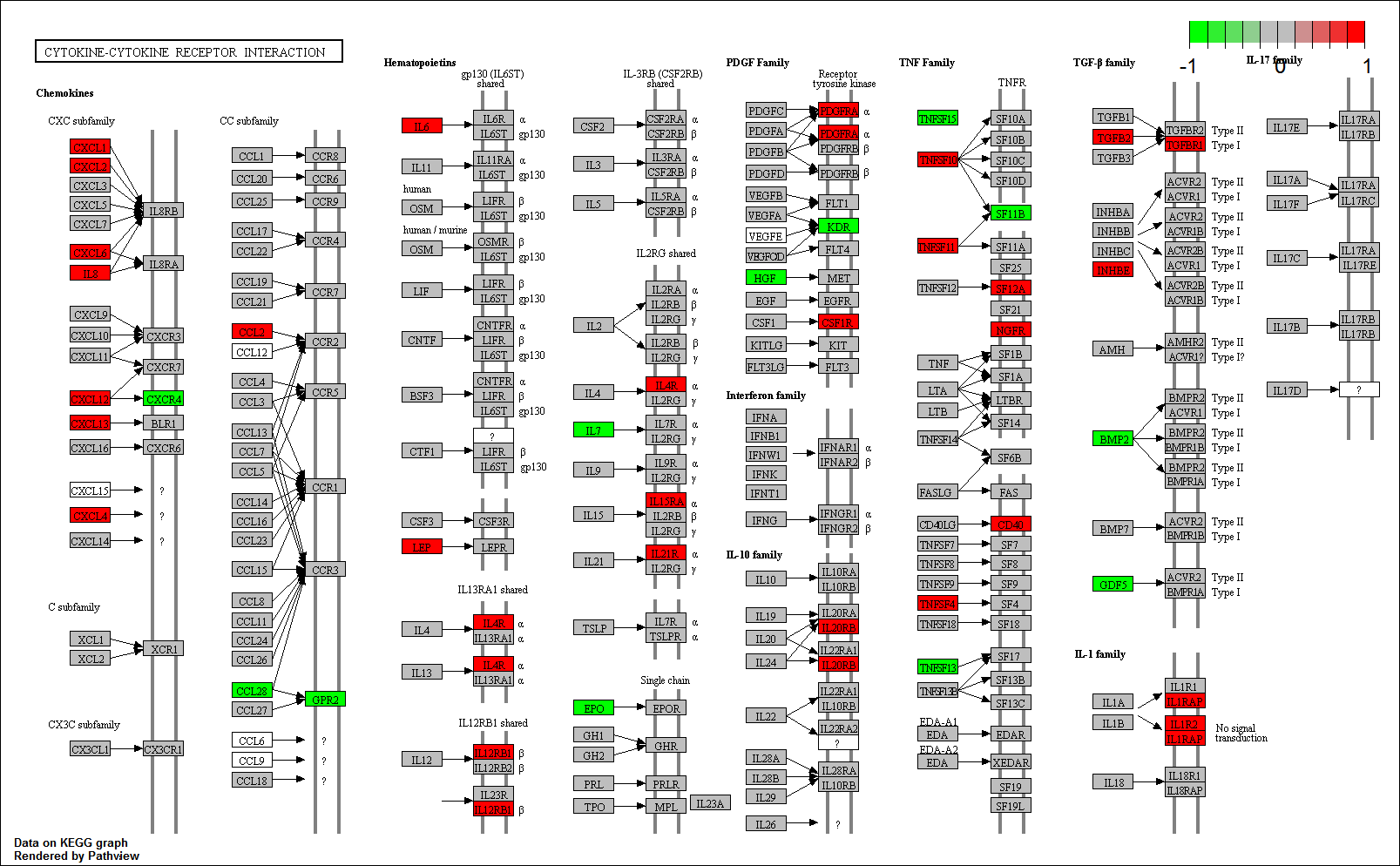

Supplement: DATASET S3 — GO-term analyses of GATA3-expressing and unscratched pHAs versus EGFP-expressing and unscratched pHAs in 2D cultures. [file Data_Sheet_3.ZIP › GO_term_analyses_GATA3u_vs_GFPu/clusterProfiler/hsa04060.Cytokine-cytokinereceptorinteraction.png]

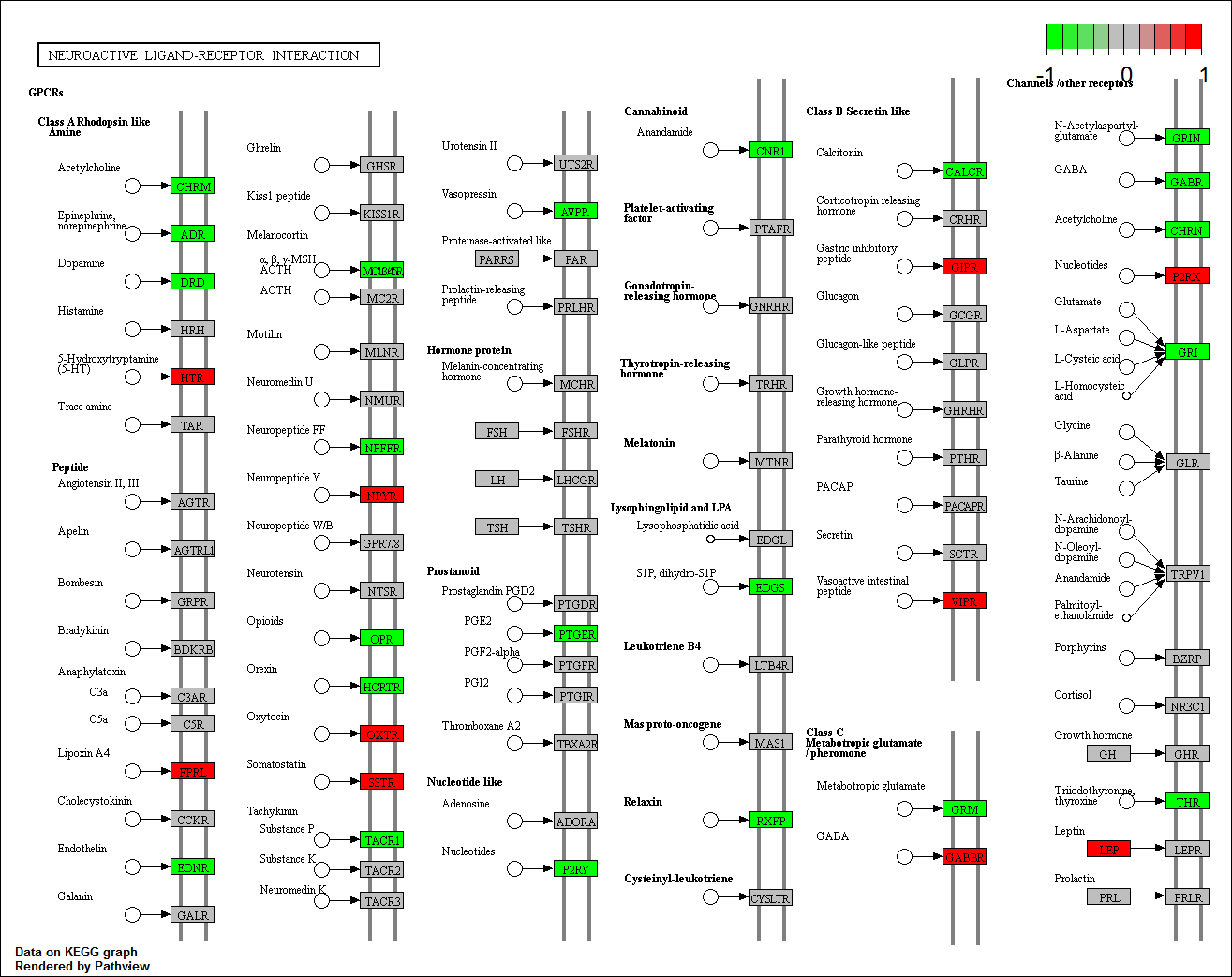

Supplement: DATASET S3 — GO-term analyses of GATA3-expressing and unscratched pHAs versus EGFP-expressing and unscratched pHAs in 2D cultures. [file Data_Sheet_3.ZIP › GO_term_analyses_GATA3u_vs_GFPu/clusterProfiler/hsa04080.Neuroactiveligand-receptorinteraction.png]

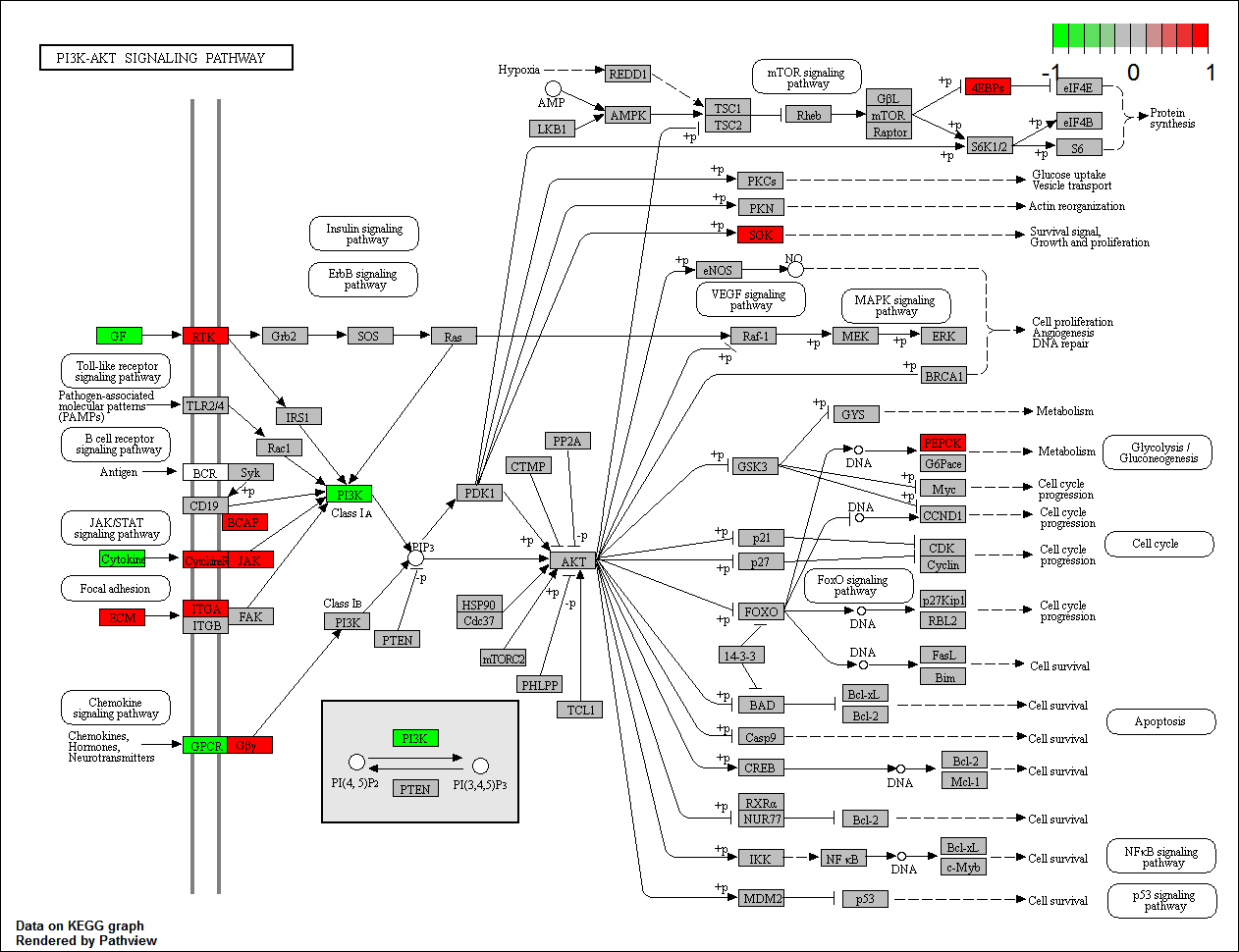

Supplement: DATASET S3 — GO-term analyses of GATA3-expressing and unscratched pHAs versus EGFP-expressing and unscratched pHAs in 2D cultures. [file Data_Sheet_3.ZIP › GO_term_analyses_GATA3u_vs_GFPu/clusterProfiler/hsa04151.PI3K-Aktsignalingpathway.png]

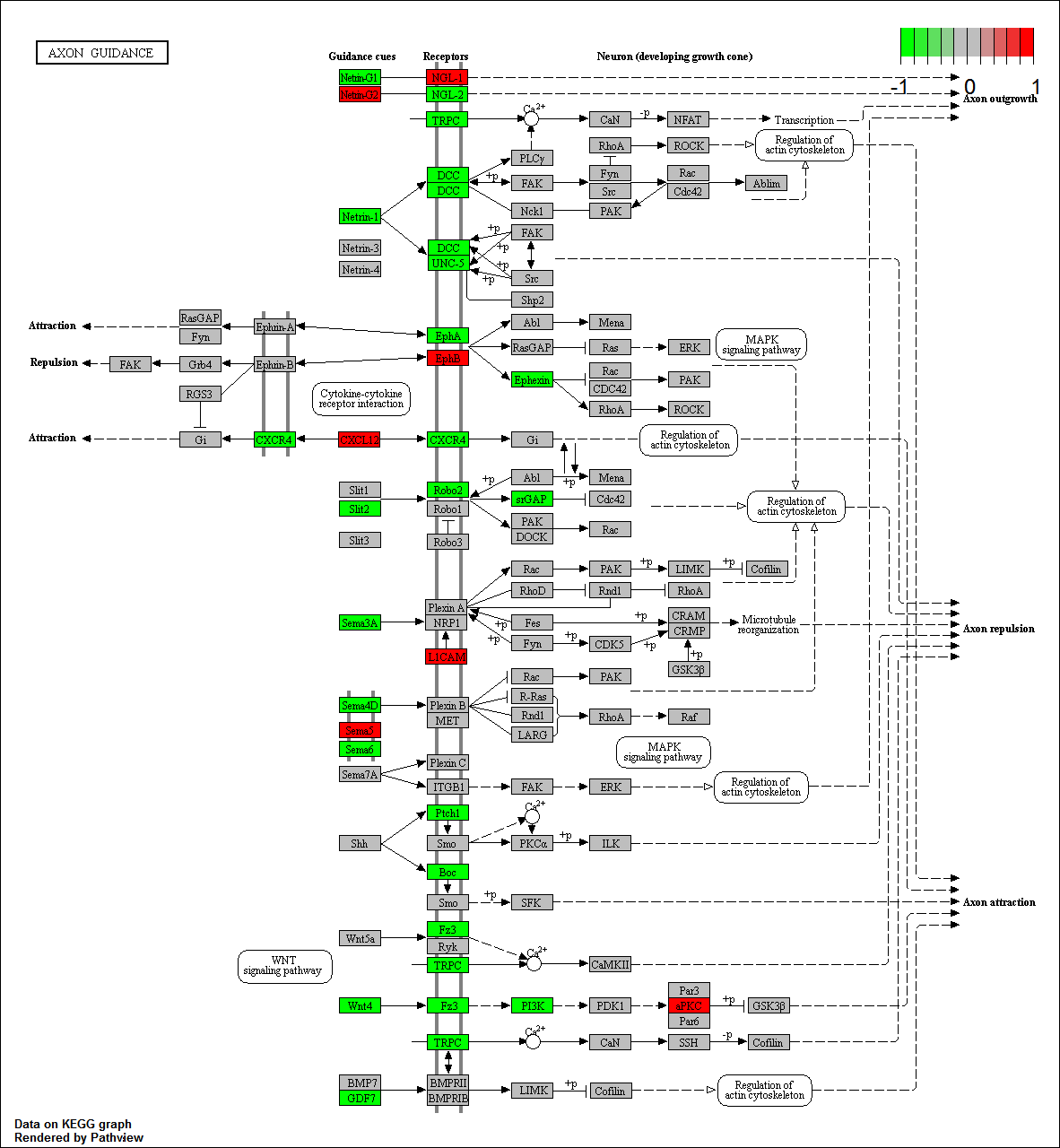

Supplement: DATASET S3 — GO-term analyses of GATA3-expressing and unscratched pHAs versus EGFP-expressing and unscratched pHAs in 2D cultures. [file Data_Sheet_3.ZIP › GO_term_analyses_GATA3u_vs_GFPu/clusterProfiler/hsa04360.Axonguidance.png]

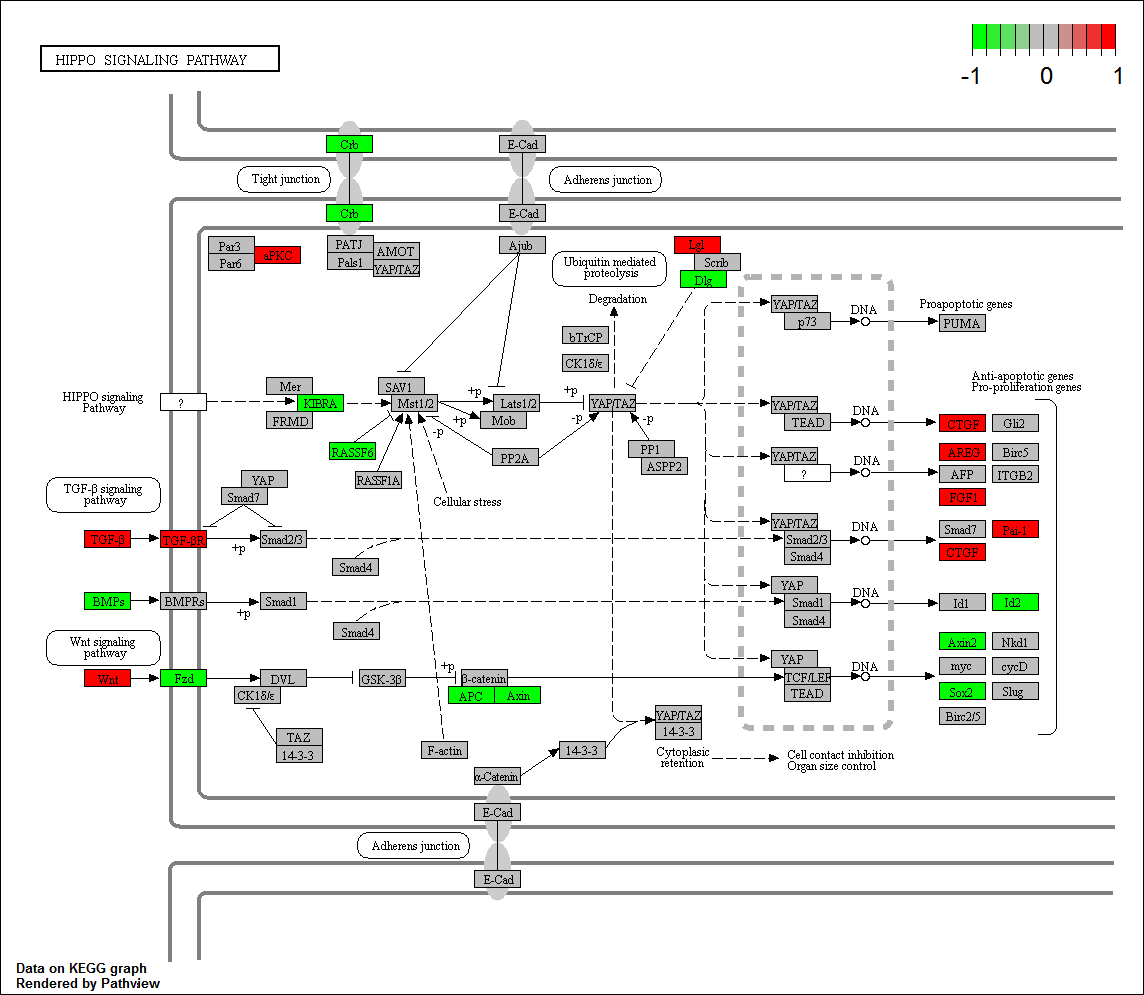

Supplement: DATASET S3 — GO-term analyses of GATA3-expressing and unscratched pHAs versus EGFP-expressing and unscratched pHAs in 2D cultures. [file Data_Sheet_3.ZIP › GO_term_analyses_GATA3u_vs_GFPu/clusterProfiler/hsa04390.Hipposignalingpathway.png]

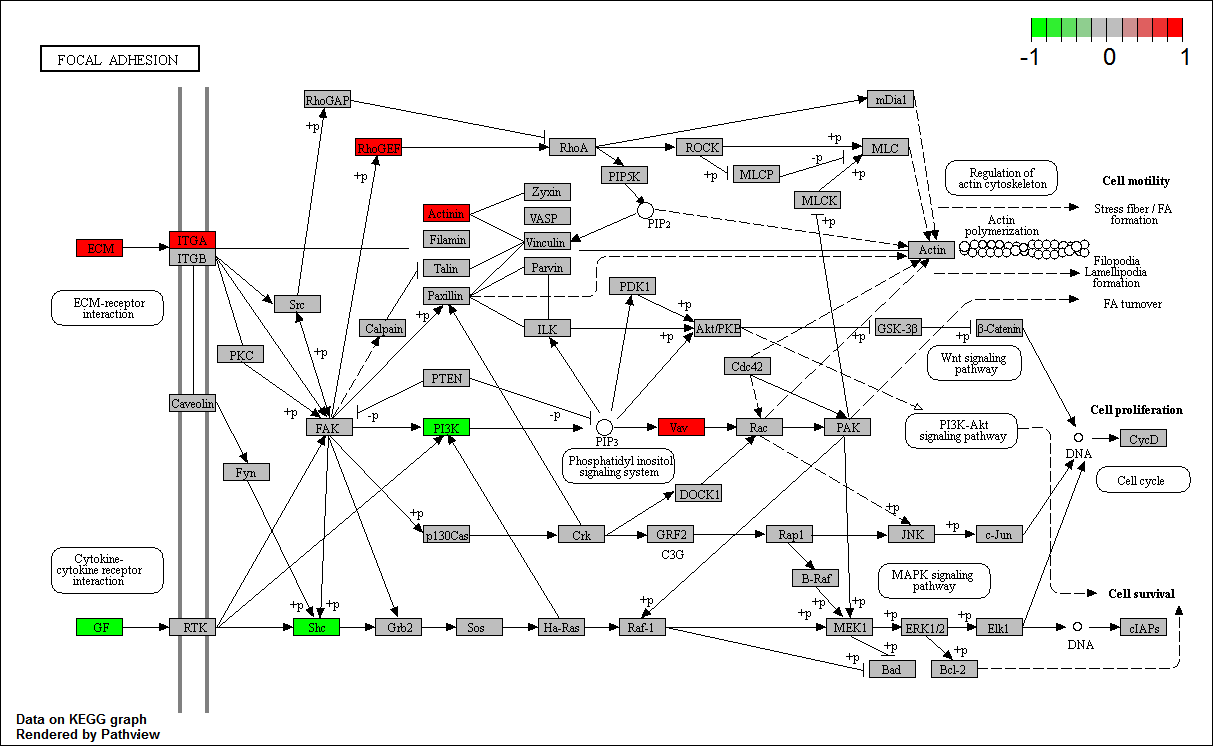

Supplement: DATASET S3 — GO-term analyses of GATA3-expressing and unscratched pHAs versus EGFP-expressing and unscratched pHAs in 2D cultures. [file Data_Sheet_3.ZIP › GO_term_analyses_GATA3u_vs_GFPu/clusterProfiler/hsa04510.Focaladhesion.png]

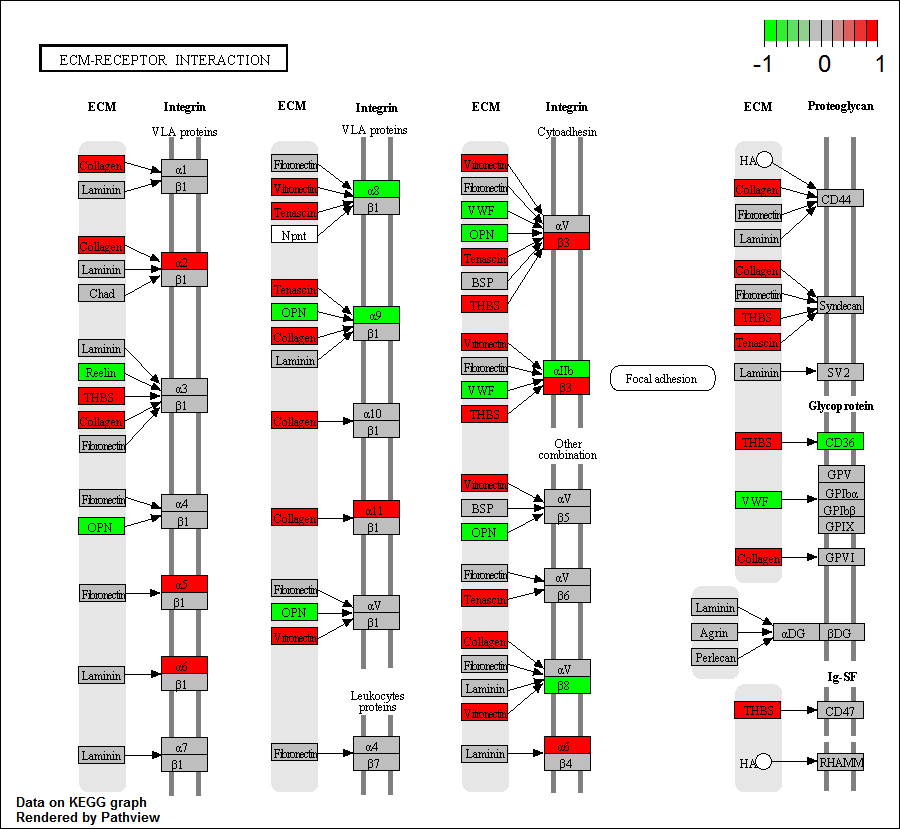

Supplement: DATASET S3 — GO-term analyses of GATA3-expressing and unscratched pHAs versus EGFP-expressing and unscratched pHAs in 2D cultures. [file Data_Sheet_3.ZIP › GO_term_analyses_GATA3u_vs_GFPu/clusterProfiler/hsa04512.ECM-receptorinteraction.png]

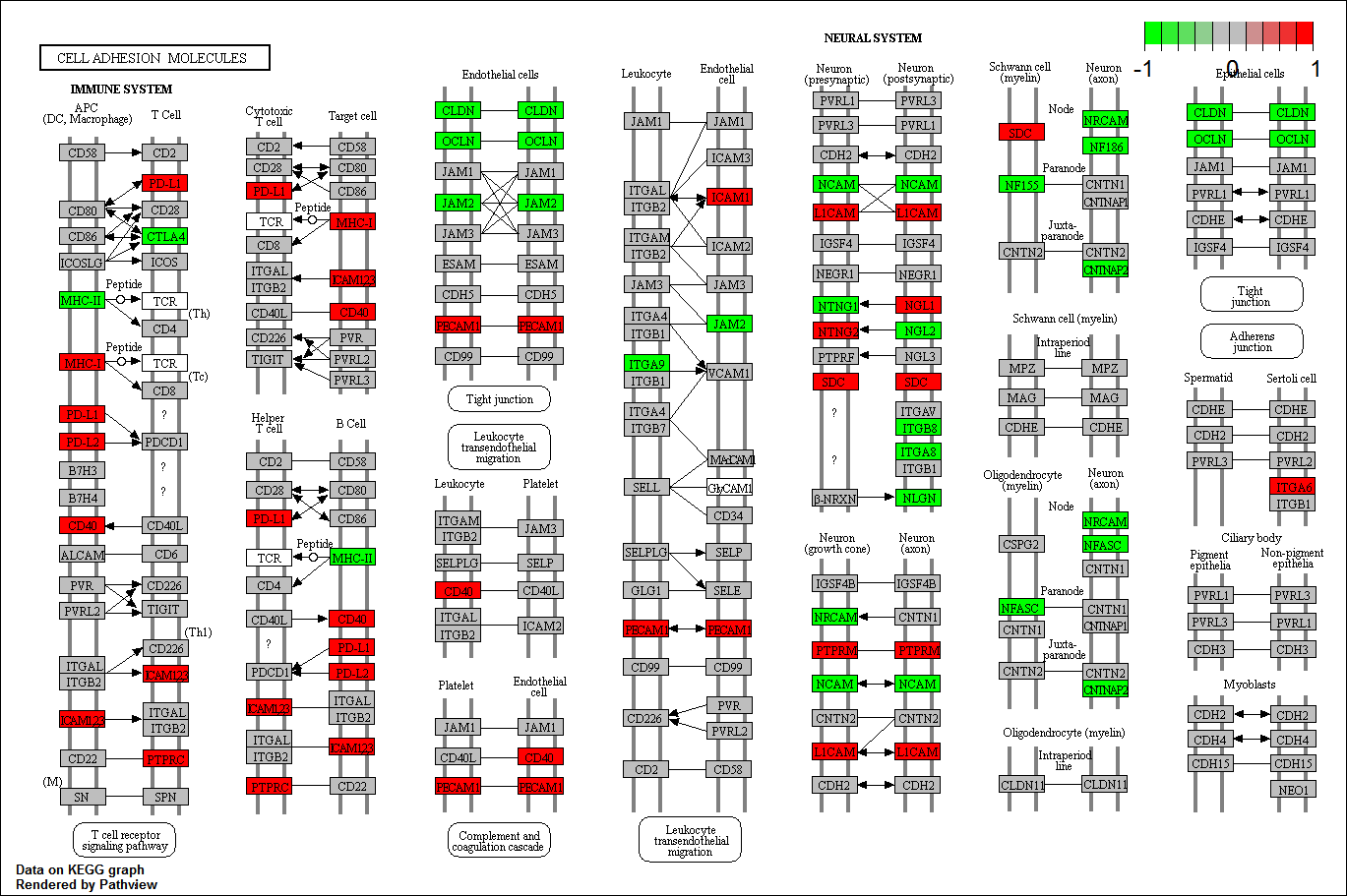

Supplement: DATASET S3 — GO-term analyses of GATA3-expressing and unscratched pHAs versus EGFP-expressing and unscratched pHAs in 2D cultures. [file Data_Sheet_3.ZIP › GO_term_analyses_GATA3u_vs_GFPu/clusterProfiler/hsa04514.Celladhesionmolecules(CAMs).png]

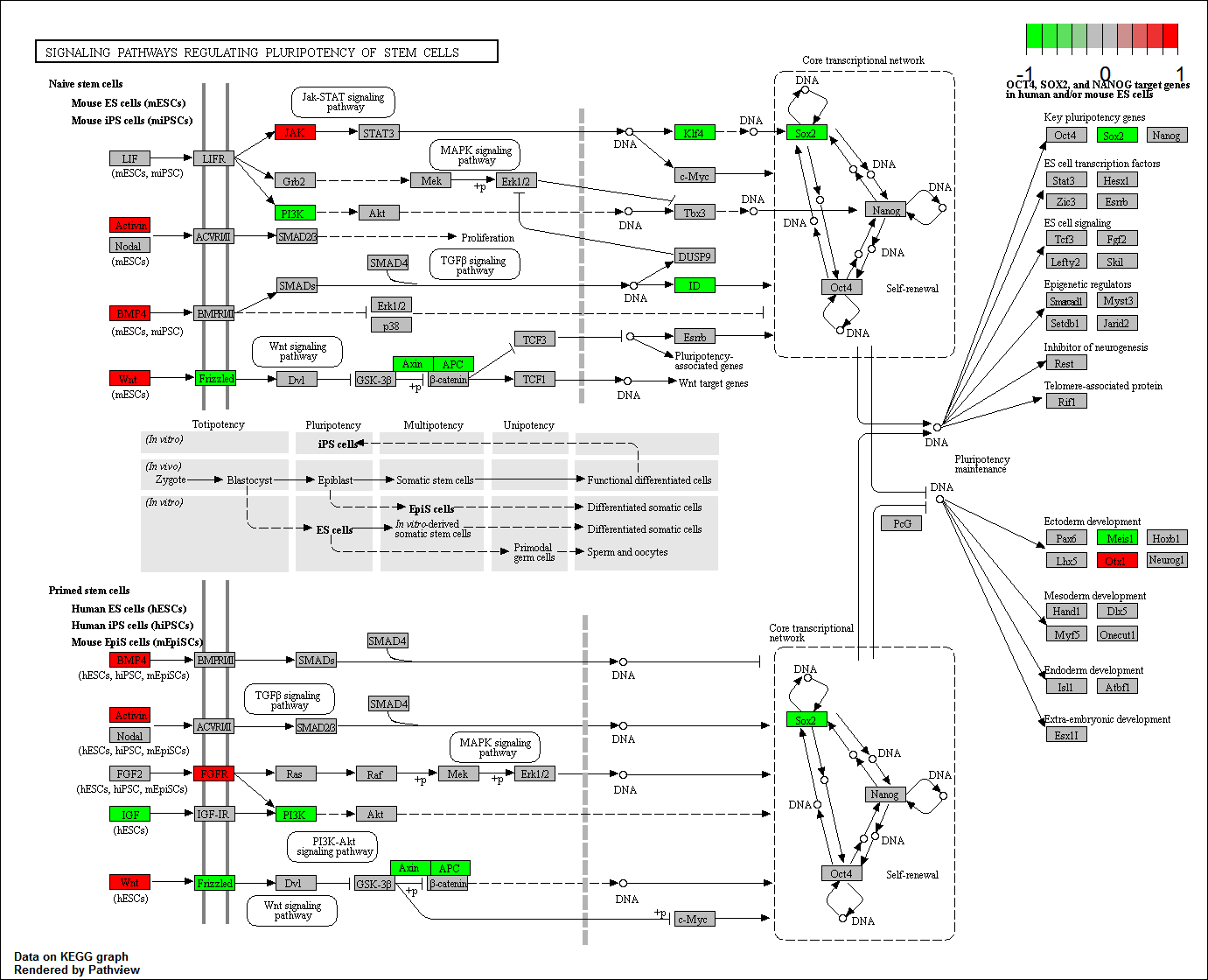

Supplement: DATASET S3 — GO-term analyses of GATA3-expressing and unscratched pHAs versus EGFP-expressing and unscratched pHAs in 2D cultures. [file Data_Sheet_3.ZIP › GO_term_analyses_GATA3u_vs_GFPu/clusterProfiler/hsa04550.Signalingpathwaysregulatingpluripotencyofstemcells.png]

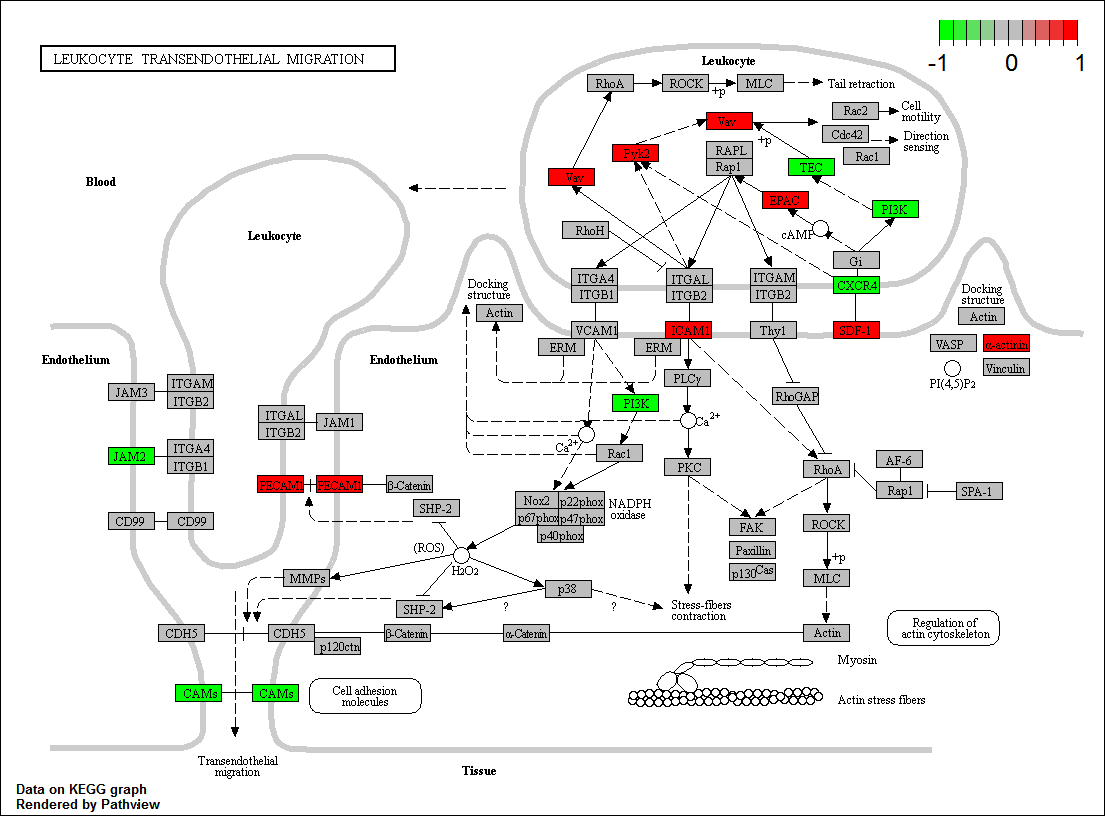

Supplement: DATASET S3 — GO-term analyses of GATA3-expressing and unscratched pHAs versus EGFP-expressing and unscratched pHAs in 2D cultures. [file Data_Sheet_3.ZIP › GO_term_analyses_GATA3u_vs_GFPu/clusterProfiler/hsa04670.Leukocytetransendothelialmigration.png]

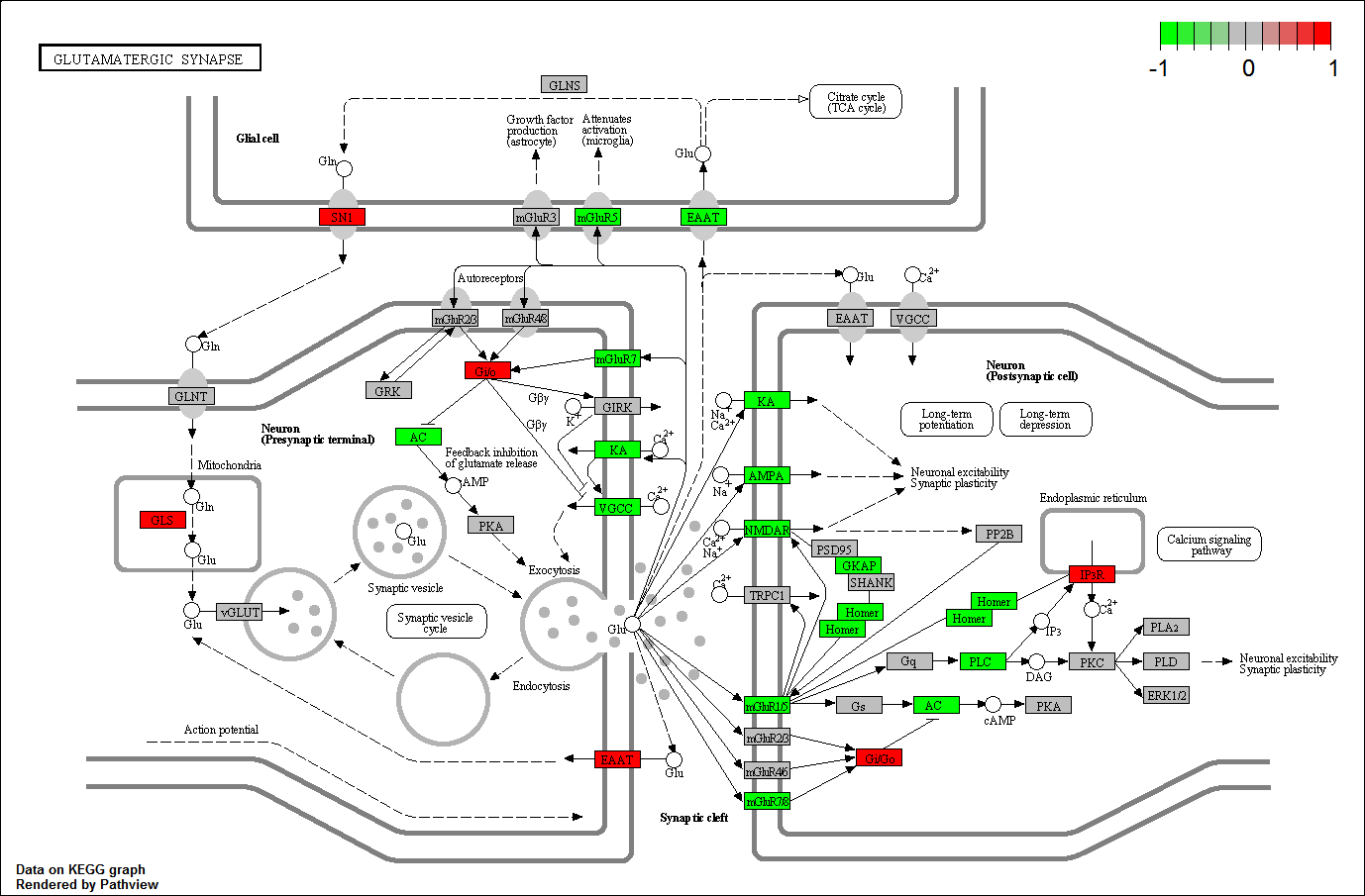

Supplement: DATASET S3 — GO-term analyses of GATA3-expressing and unscratched pHAs versus EGFP-expressing and unscratched pHAs in 2D cultures. [file Data_Sheet_3.ZIP › GO_term_analyses_GATA3u_vs_GFPu/clusterProfiler/hsa04724.Glutamatergicsynapse.png]

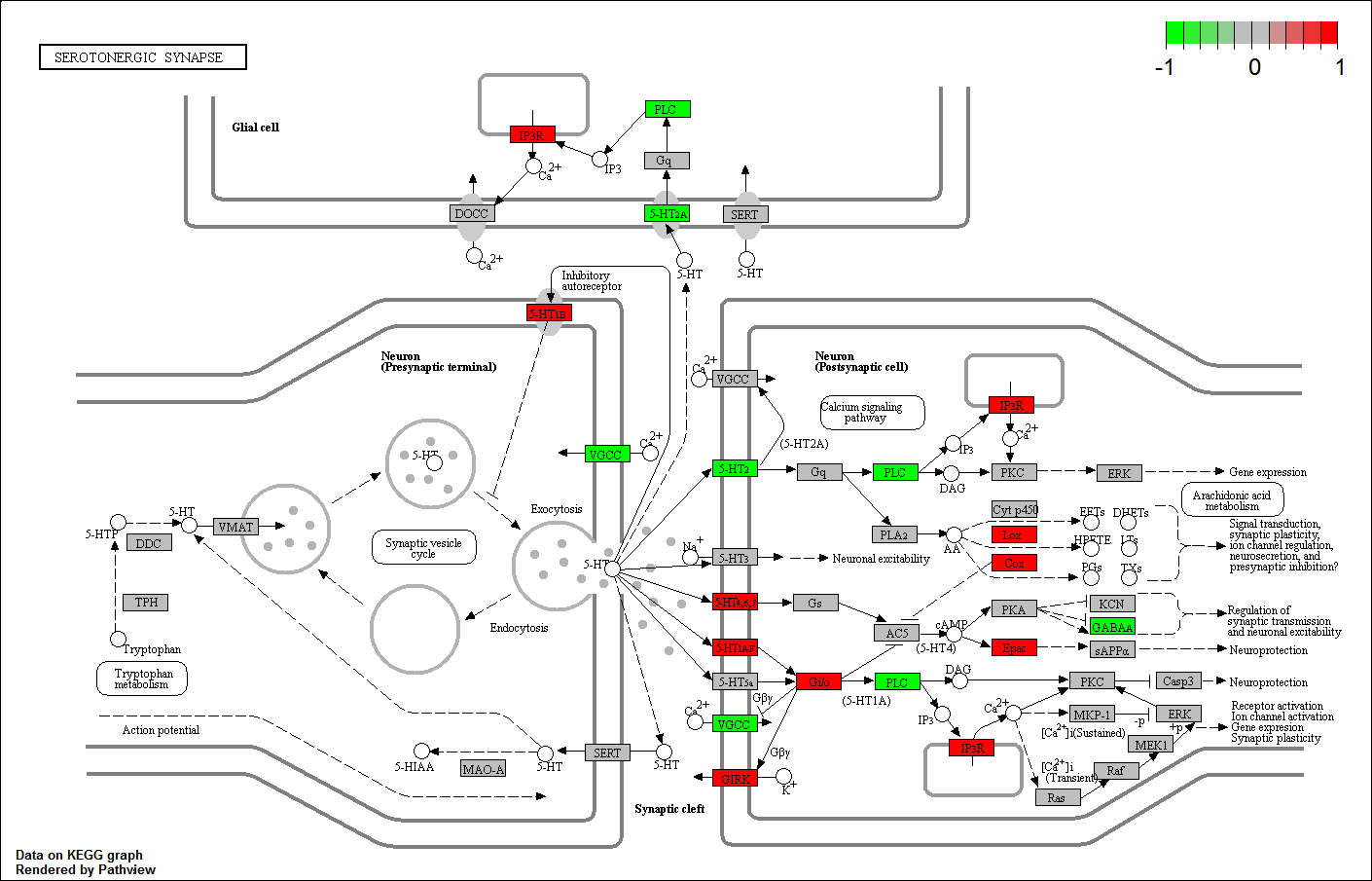

Supplement: DATASET S3 — GO-term analyses of GATA3-expressing and unscratched pHAs versus EGFP-expressing and unscratched pHAs in 2D cultures. [file Data_Sheet_3.ZIP › GO_term_analyses_GATA3u_vs_GFPu/clusterProfiler/hsa04726.Serotonergicsynapse.png]

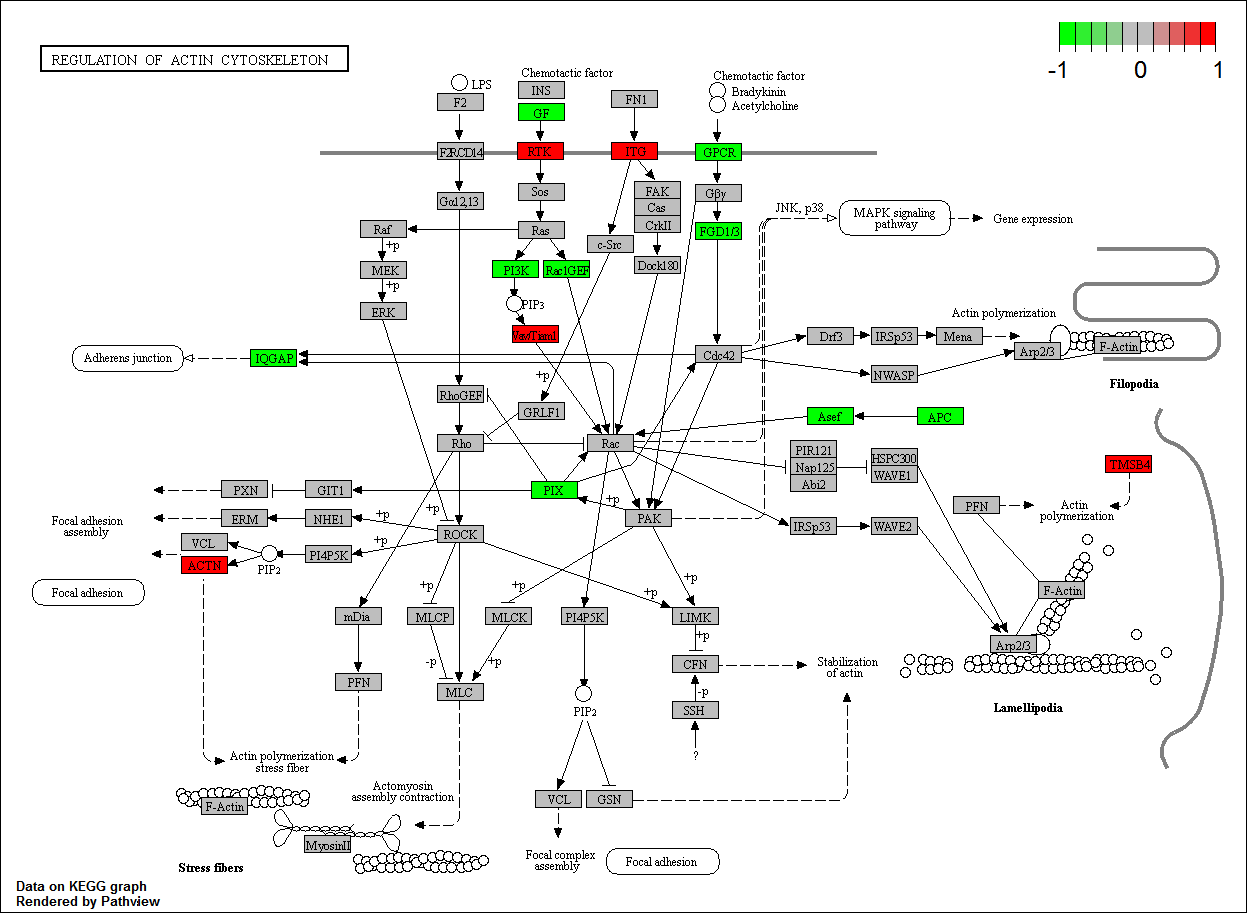

Supplement: DATASET S3 — GO-term analyses of GATA3-expressing and unscratched pHAs versus EGFP-expressing and unscratched pHAs in 2D cultures. [file Data_Sheet_3.ZIP › GO_term_analyses_GATA3u_vs_GFPu/clusterProfiler/hsa04810.Regulationofactincytoskeleton.png]

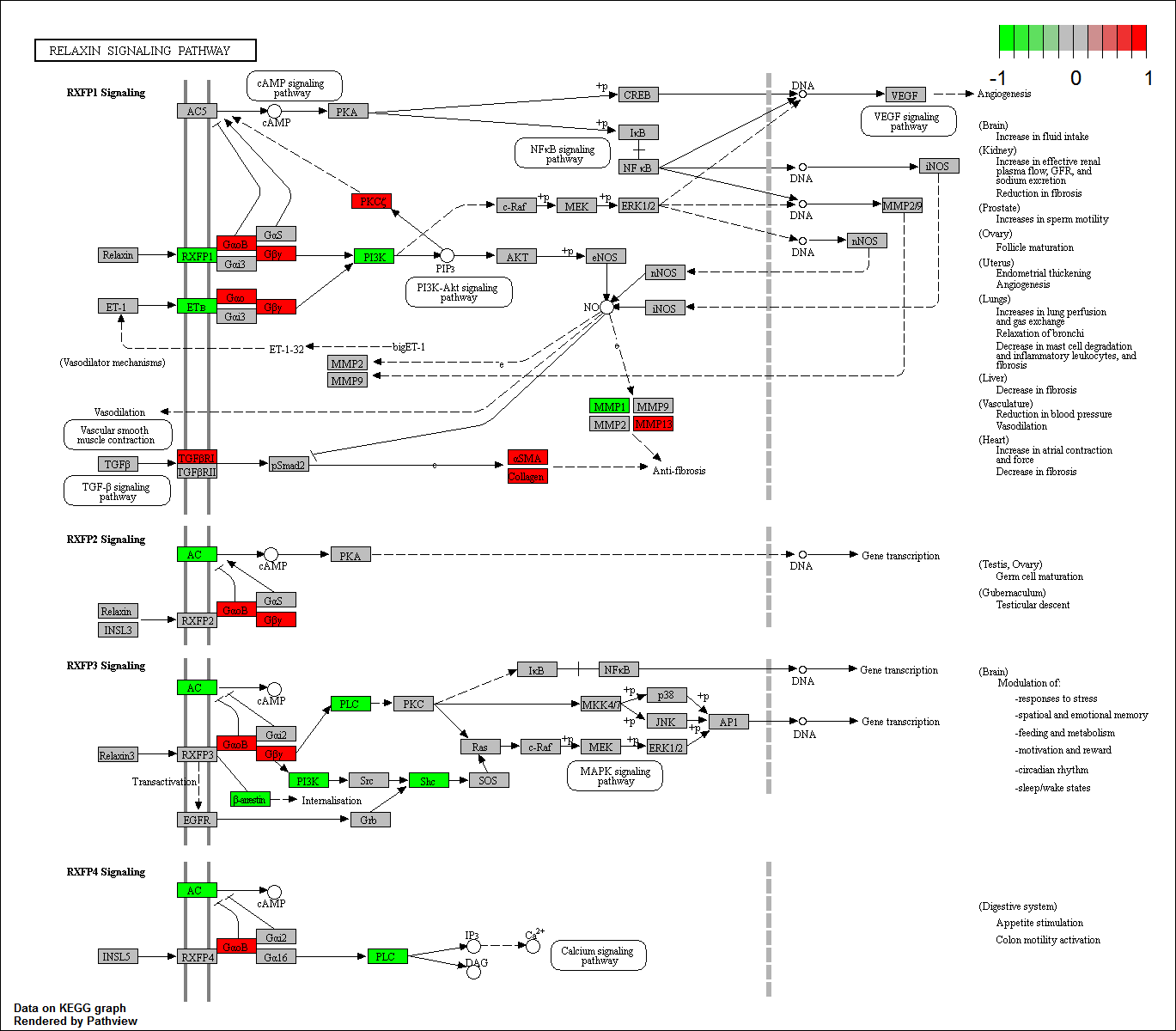

Supplement: DATASET S3 — GO-term analyses of GATA3-expressing and unscratched pHAs versus EGFP-expressing and unscratched pHAs in 2D cultures. [file Data_Sheet_3.ZIP › GO_term_analyses_GATA3u_vs_GFPu/clusterProfiler/hsa04926.Relaxinsignalingpathway.png]

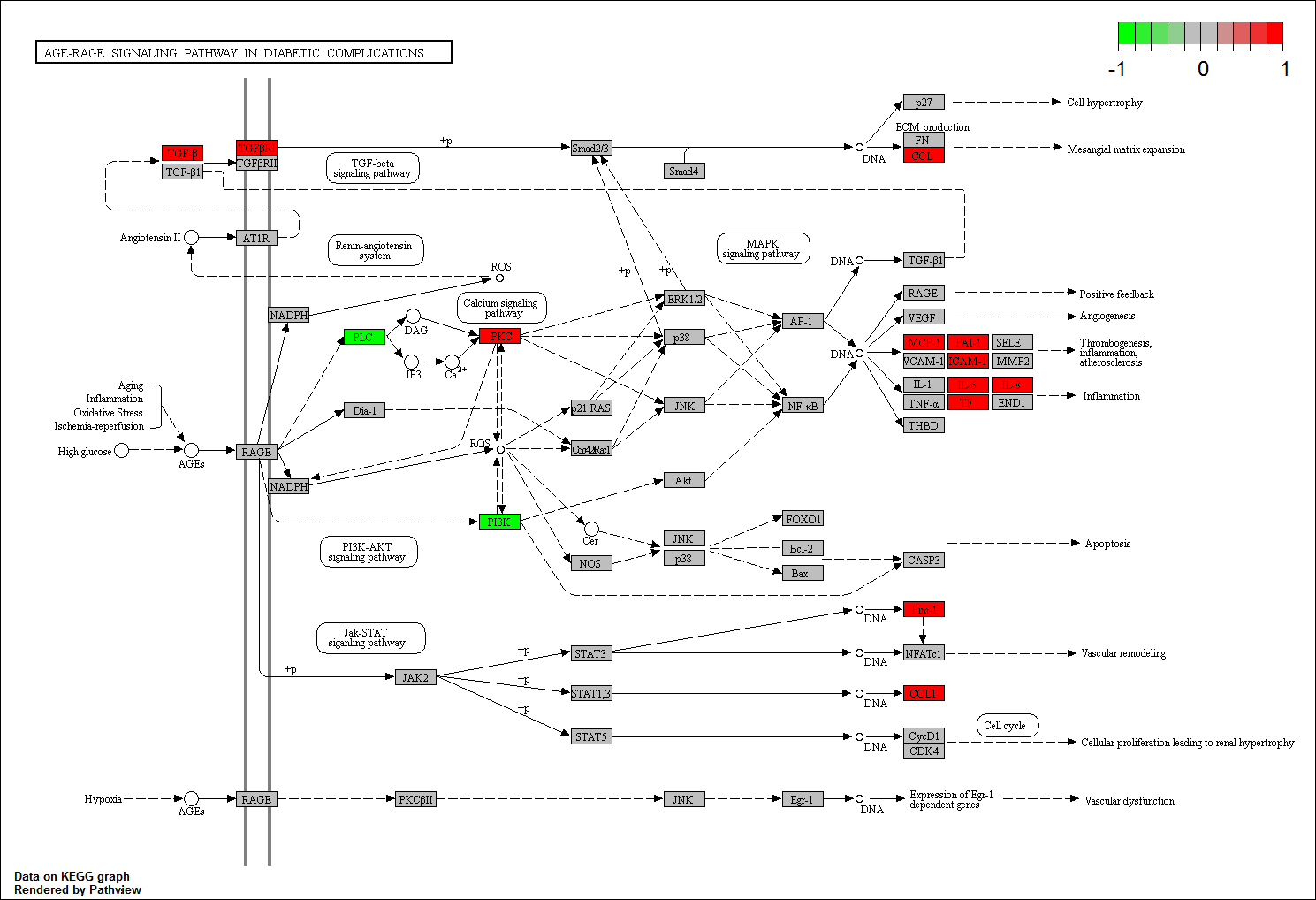

Supplement: DATASET S3 — GO-term analyses of GATA3-expressing and unscratched pHAs versus EGFP-expressing and unscratched pHAs in 2D cultures. [file Data_Sheet_3.ZIP › GO_term_analyses_GATA3u_vs_GFPu/clusterProfiler/hsa04933.AGE-RAGEsignalingpathwayindiabeticcomplications.png]

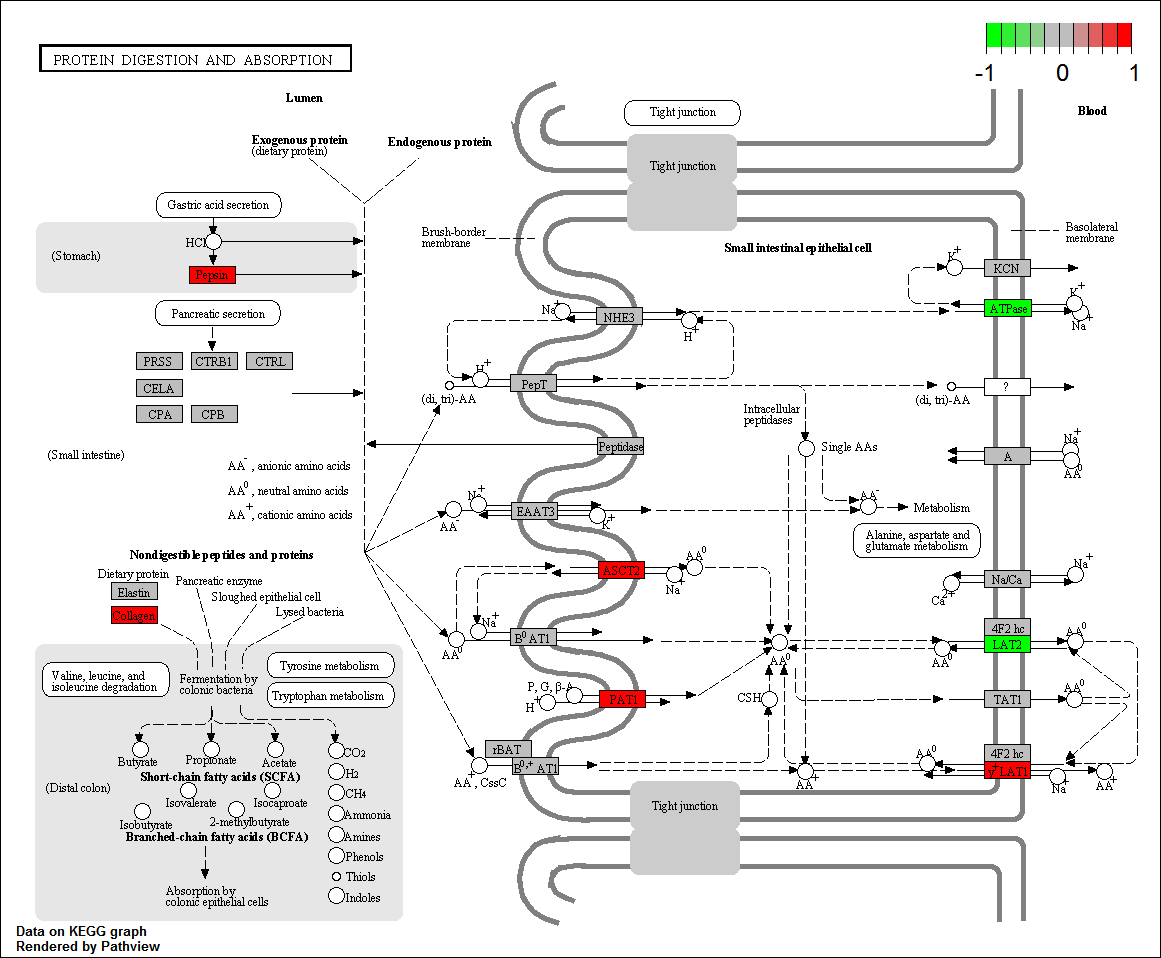

Supplement: DATASET S3 — GO-term analyses of GATA3-expressing and unscratched pHAs versus EGFP-expressing and unscratched pHAs in 2D cultures. [file Data_Sheet_3.ZIP › GO_term_analyses_GATA3u_vs_GFPu/clusterProfiler/hsa04974.Proteindigestionandabsorption.png]

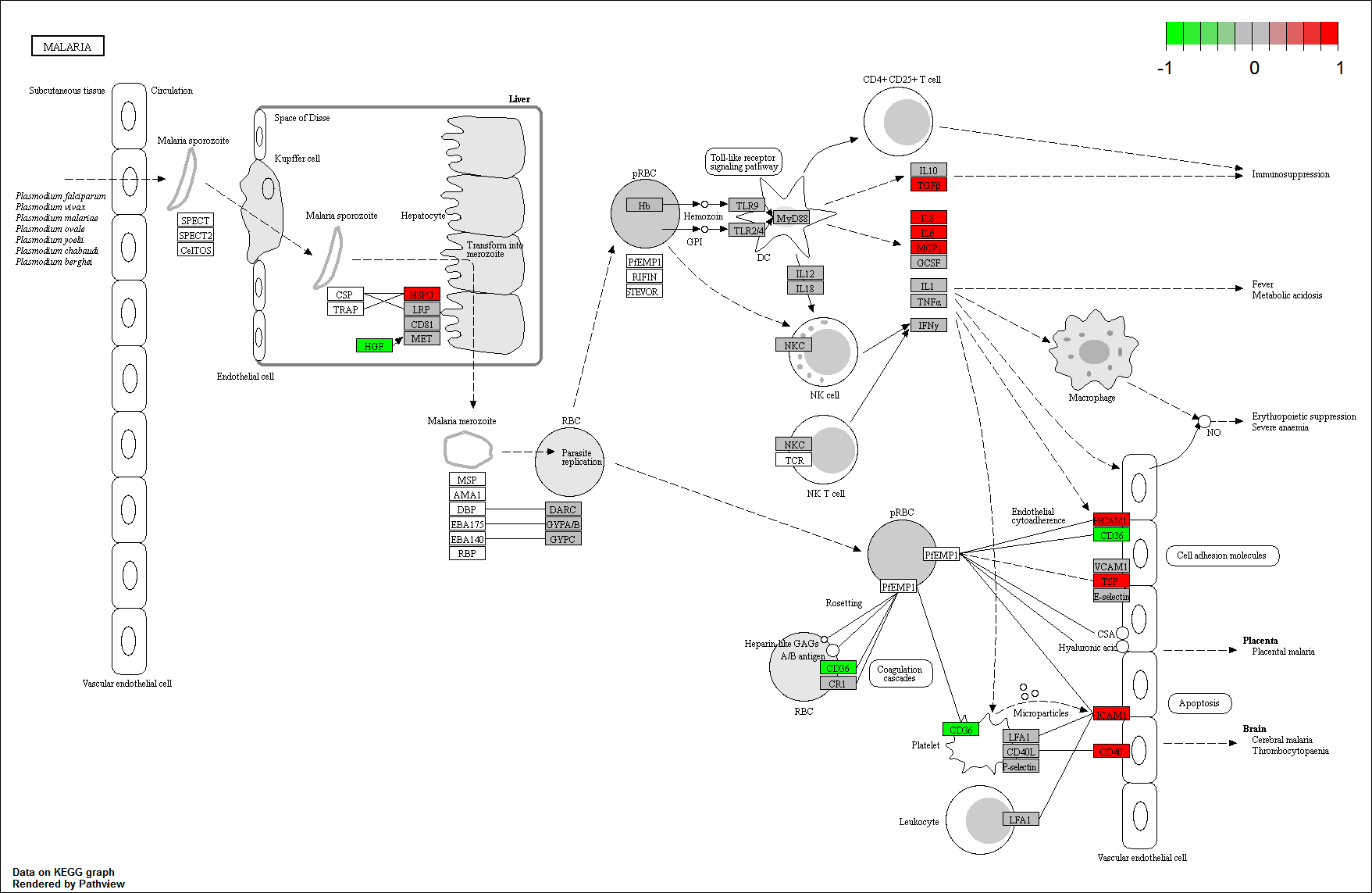

Supplement: DATASET S3 — GO-term analyses of GATA3-expressing and unscratched pHAs versus EGFP-expressing and unscratched pHAs in 2D cultures. [file Data_Sheet_3.ZIP › GO_term_analyses_GATA3u_vs_GFPu/clusterProfiler/hsa05144.Malaria.png]

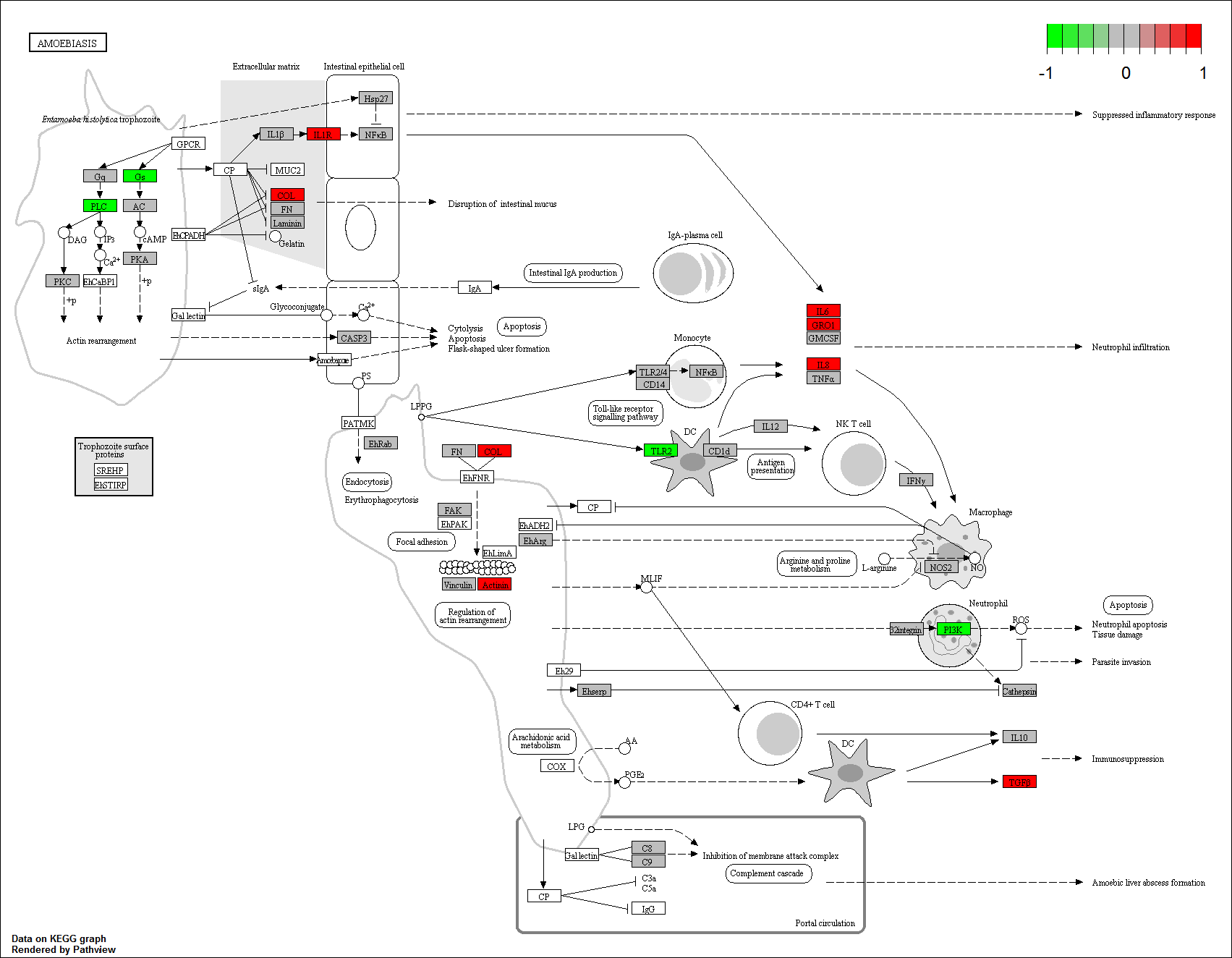

Supplement: DATASET S3 — GO-term analyses of GATA3-expressing and unscratched pHAs versus EGFP-expressing and unscratched pHAs in 2D cultures. [file Data_Sheet_3.ZIP › GO_term_analyses_GATA3u_vs_GFPu/clusterProfiler/hsa05146.Amoebiasis.png]

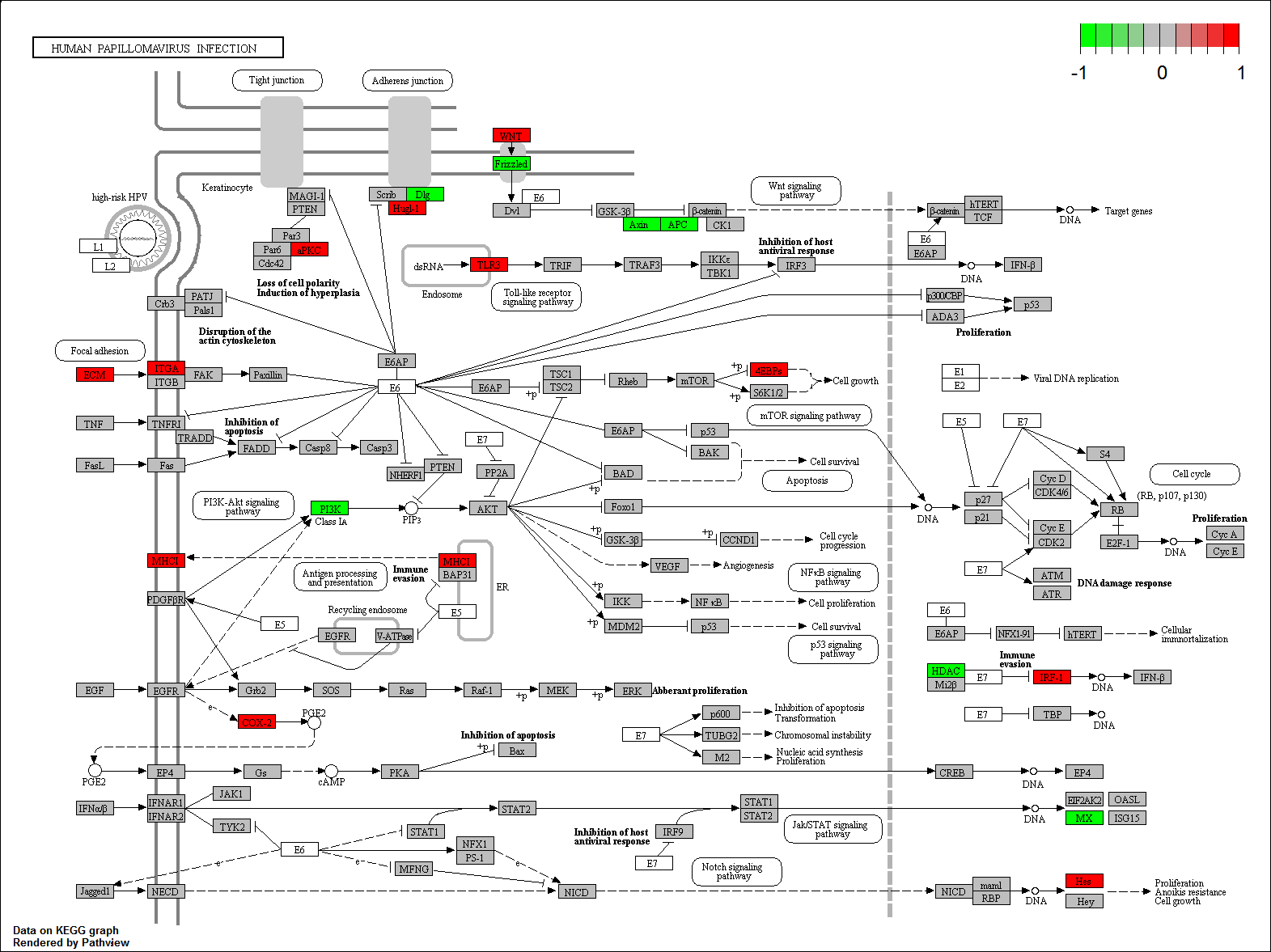

Supplement: DATASET S3 — GO-term analyses of GATA3-expressing and unscratched pHAs versus EGFP-expressing and unscratched pHAs in 2D cultures. [file Data_Sheet_3.ZIP › GO_term_analyses_GATA3u_vs_GFPu/clusterProfiler/hsa05165.Humanpapillomavirusinfection.png]

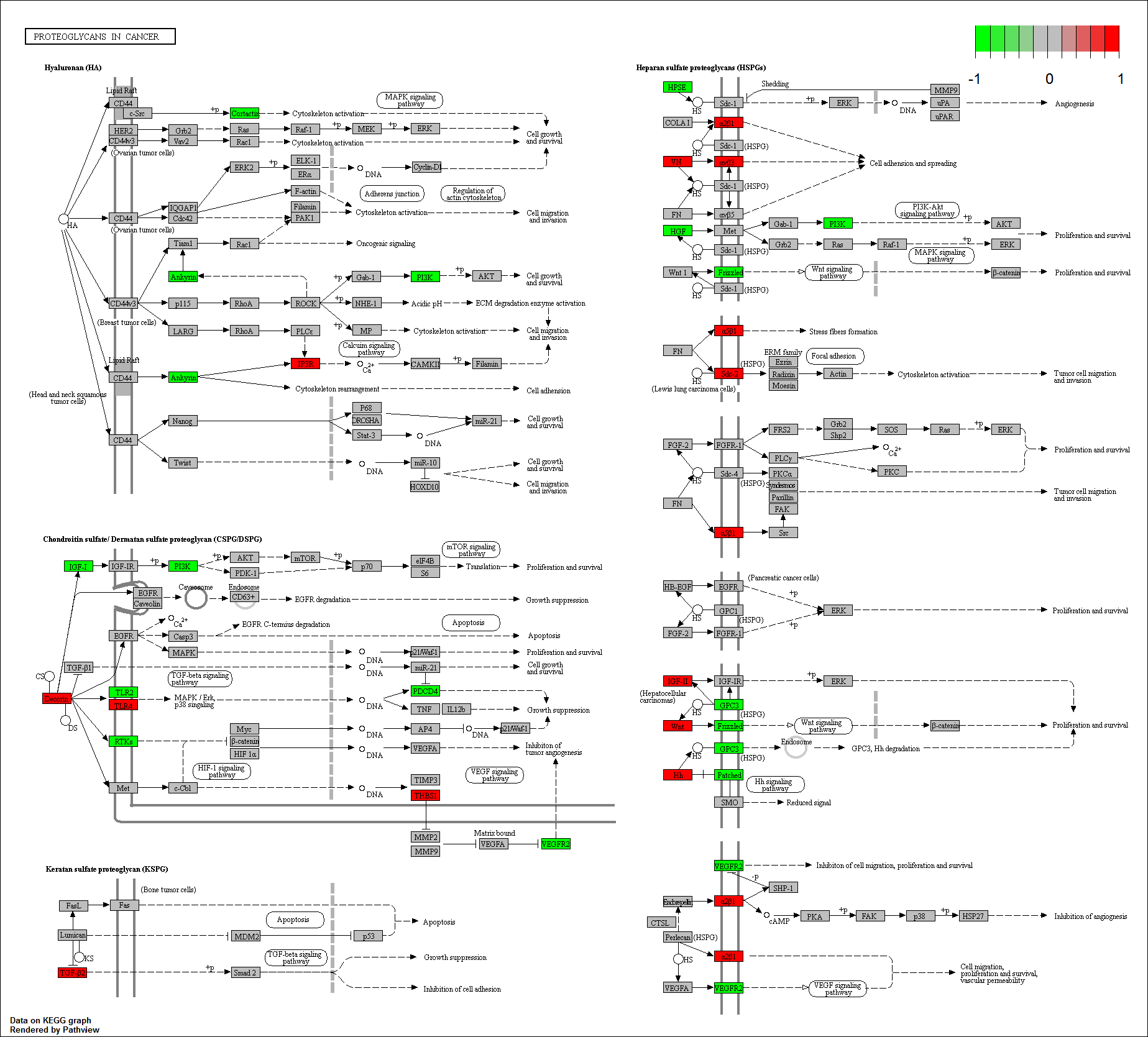

Supplement: DATASET S3 — GO-term analyses of GATA3-expressing and unscratched pHAs versus EGFP-expressing and unscratched pHAs in 2D cultures. [file Data_Sheet_3.ZIP › GO_term_analyses_GATA3u_vs_GFPu/clusterProfiler/hsa05205.Proteoglycansincancer.png]

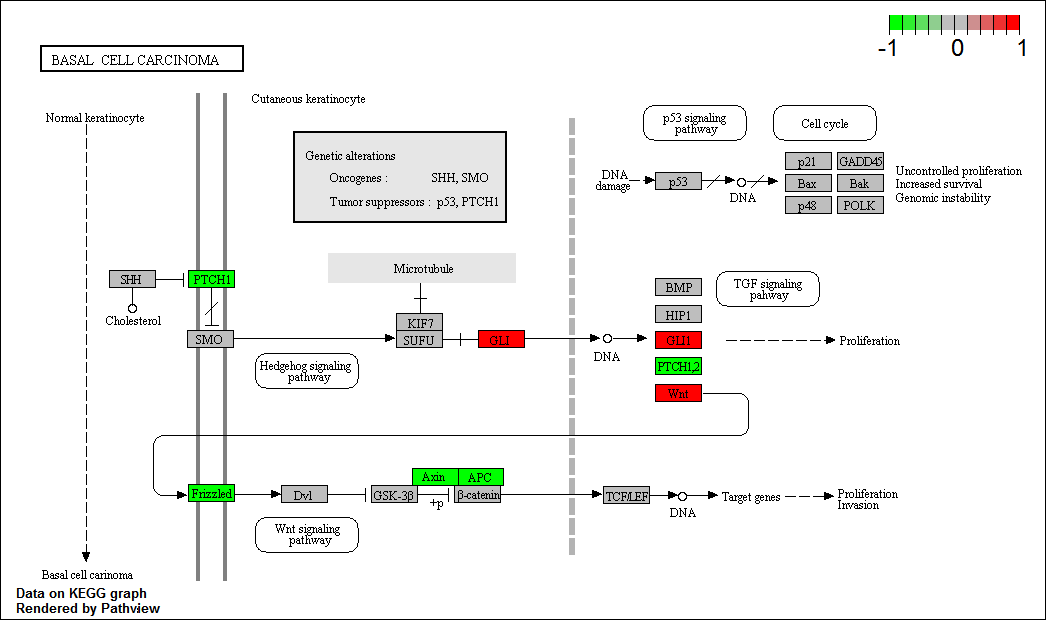

Supplement: DATASET S3 — GO-term analyses of GATA3-expressing and unscratched pHAs versus EGFP-expressing and unscratched pHAs in 2D cultures. [file Data_Sheet_3.ZIP › GO_term_analyses_GATA3u_vs_GFPu/clusterProfiler/hsa05217.Basalcellcarcinoma.png]

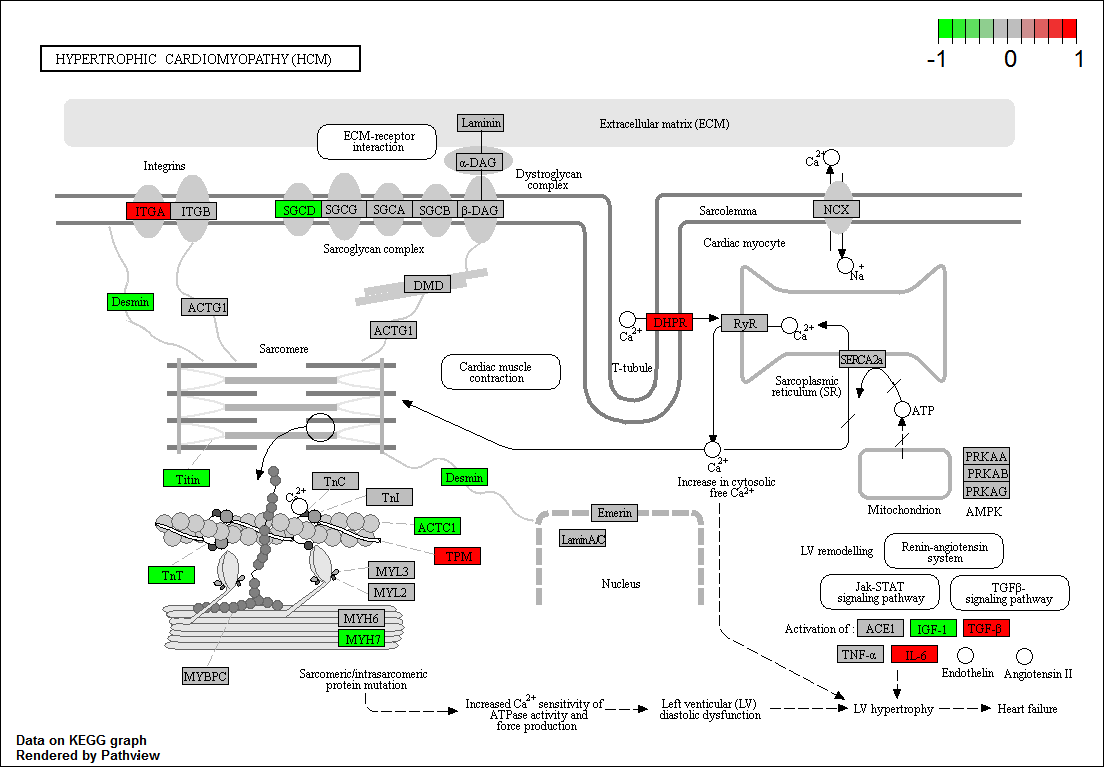

Supplement: DATASET S3 — GO-term analyses of GATA3-expressing and unscratched pHAs versus EGFP-expressing and unscratched pHAs in 2D cultures. [file Data_Sheet_3.ZIP › GO_term_analyses_GATA3u_vs_GFPu/clusterProfiler/hsa05410.Hypertrophiccardiomyopathy(HCM).png]

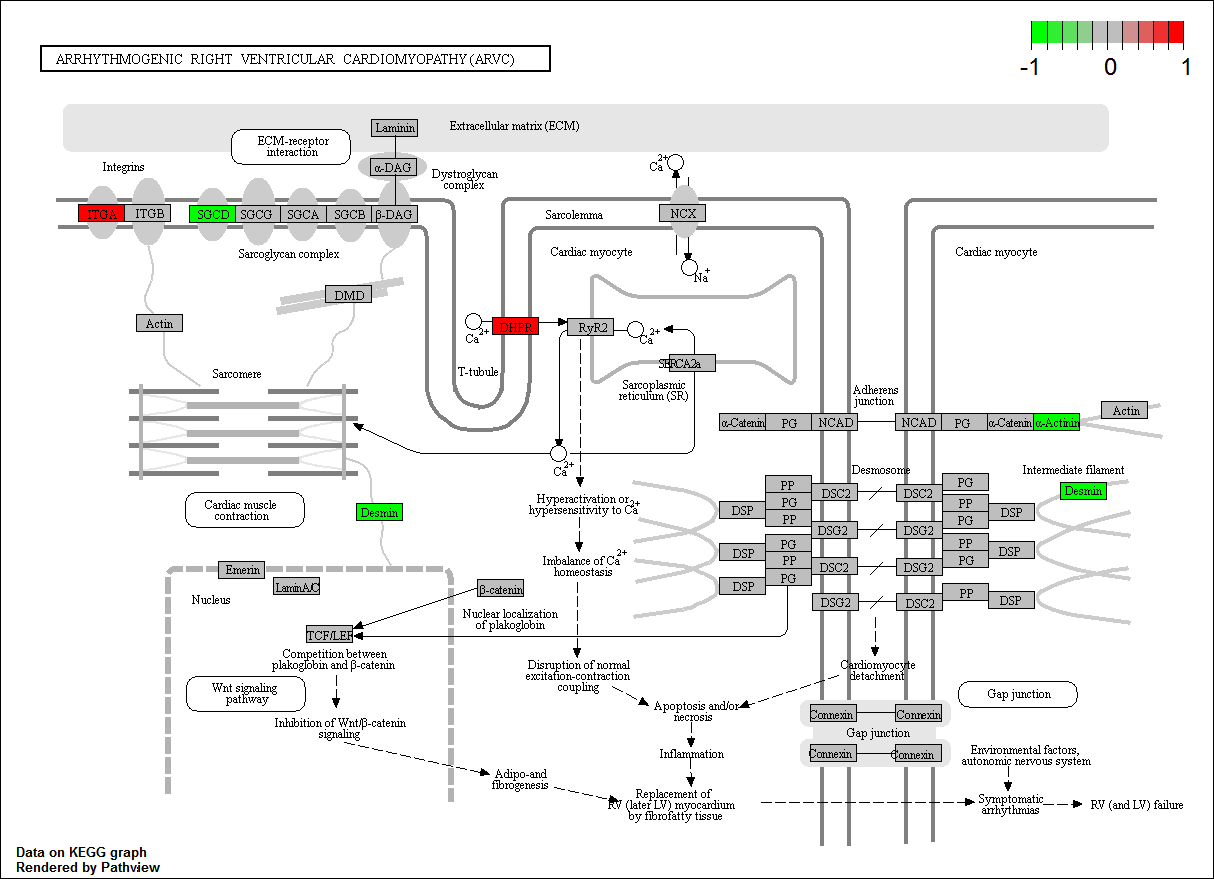

Supplement: DATASET S3 — GO-term analyses of GATA3-expressing and unscratched pHAs versus EGFP-expressing and unscratched pHAs in 2D cultures. [file Data_Sheet_3.ZIP › GO_term_analyses_GATA3u_vs_GFPu/clusterProfiler/hsa05412.Arrhythmogenicrightventricularcardiomyopathy(ARVC).png]

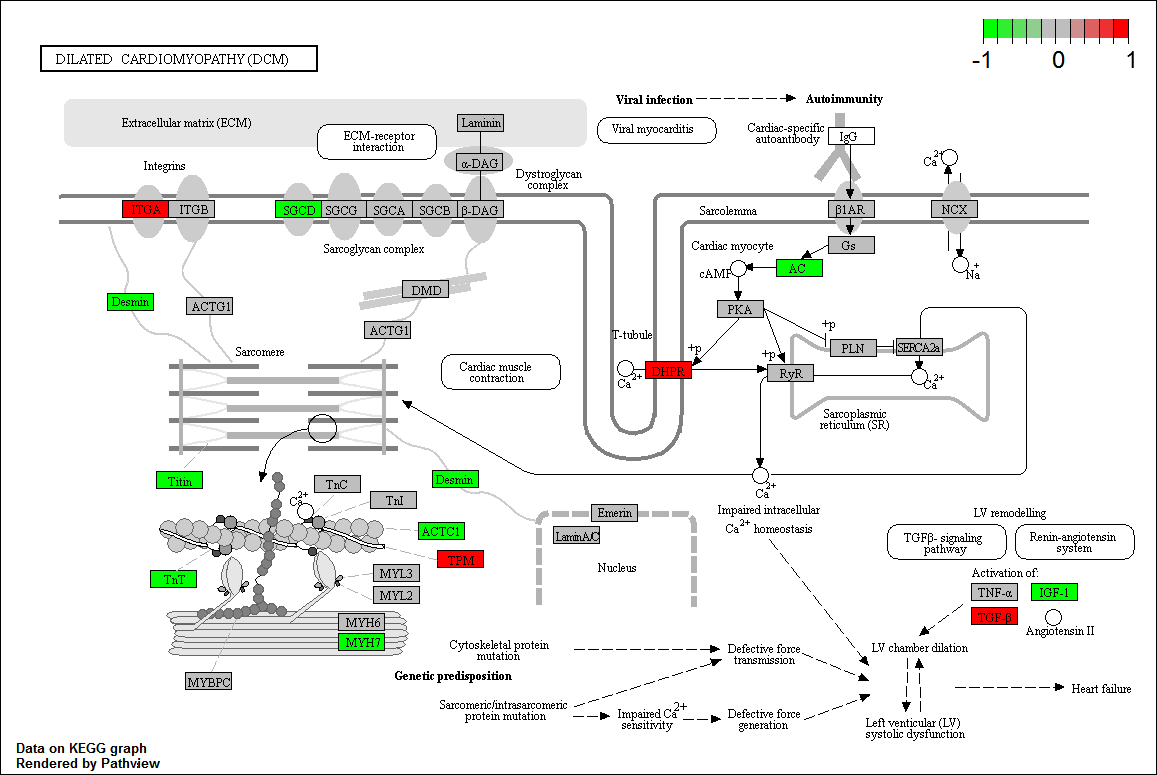

Supplement: DATASET S3 — GO-term analyses of GATA3-expressing and unscratched pHAs versus EGFP-expressing and unscratched pHAs in 2D cultures. [file Data_Sheet_3.ZIP › GO_term_analyses_GATA3u_vs_GFPu/clusterProfiler/hsa05414.Dilatedcardiomyopathy(DCM).png]

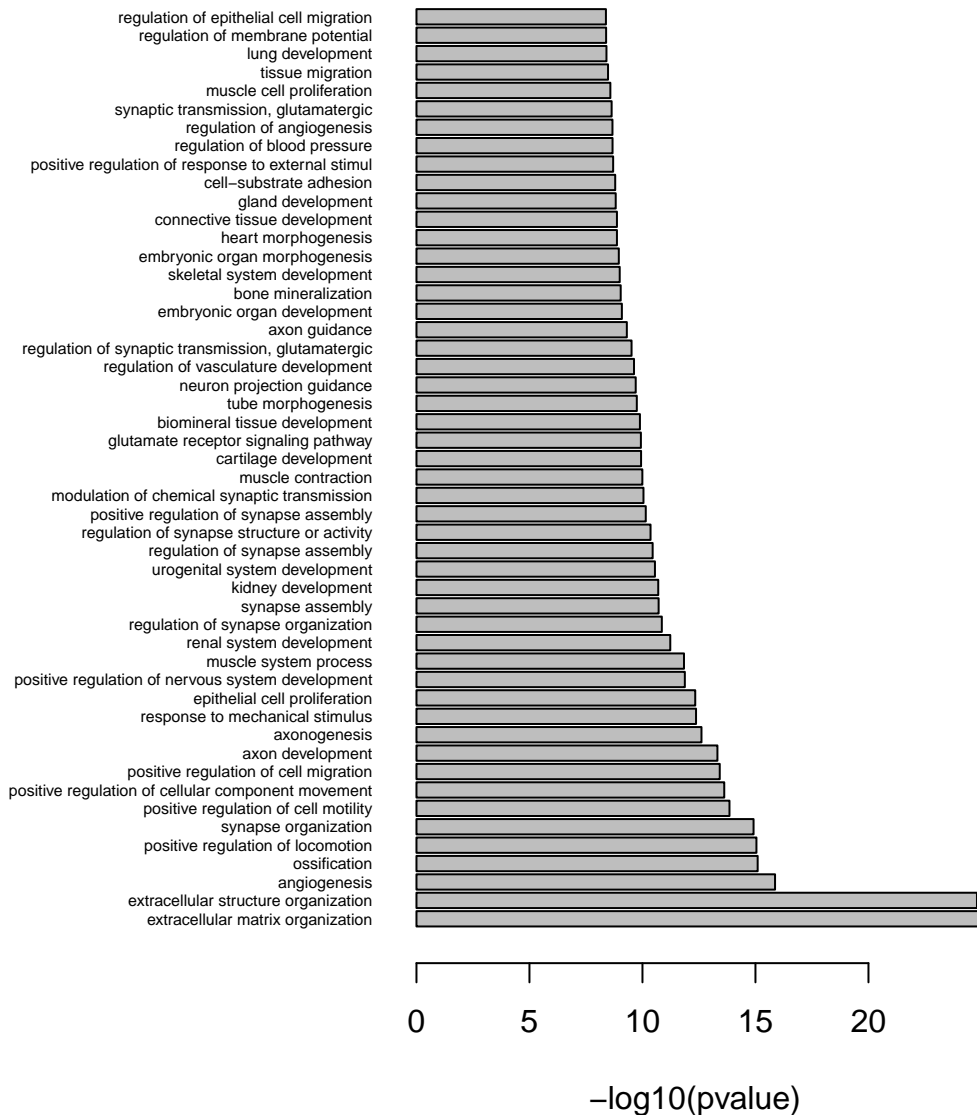

Supplement: DATASET S3 — GO-term analyses of GATA3-expressing and unscratched pHAs versus EGFP-expressing and unscratched pHAs in 2D cultures. [file Data_Sheet_3.ZIP › GO_term_analyses_GATA3u_vs_GFPu/clusterProfiler/pVal_CP_enrichGO_BP.pdf]

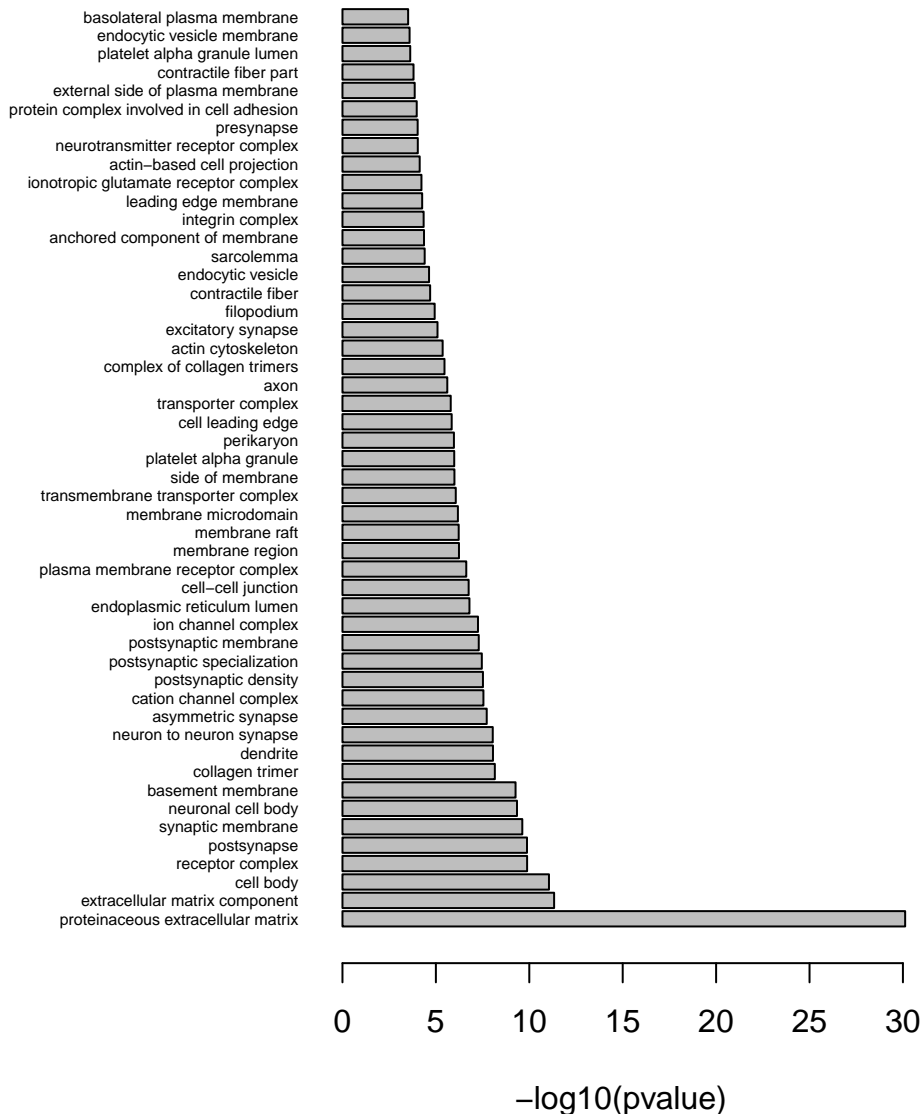

Supplement: DATASET S3 — GO-term analyses of GATA3-expressing and unscratched pHAs versus EGFP-expressing and unscratched pHAs in 2D cultures. [file Data_Sheet_3.ZIP › GO_term_analyses_GATA3u_vs_GFPu/clusterProfiler/pVal_CP_enrichGO_CC.pdf]

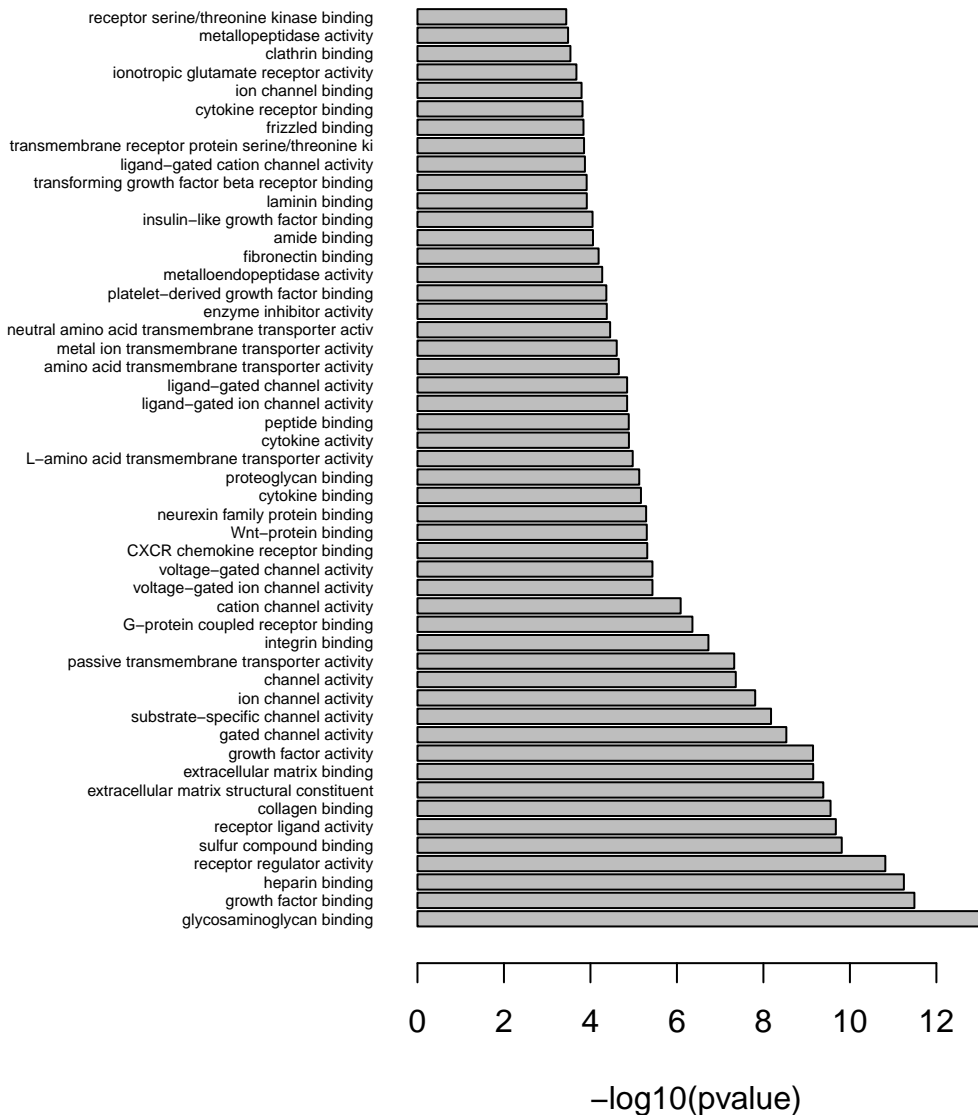

Supplement: DATASET S3 — GO-term analyses of GATA3-expressing and unscratched pHAs versus EGFP-expressing and unscratched pHAs in 2D cultures. [file Data_Sheet_3.ZIP › GO_term_analyses_GATA3u_vs_GFPu/clusterProfiler/pVal_CP_enrichGO_MF.pdf]

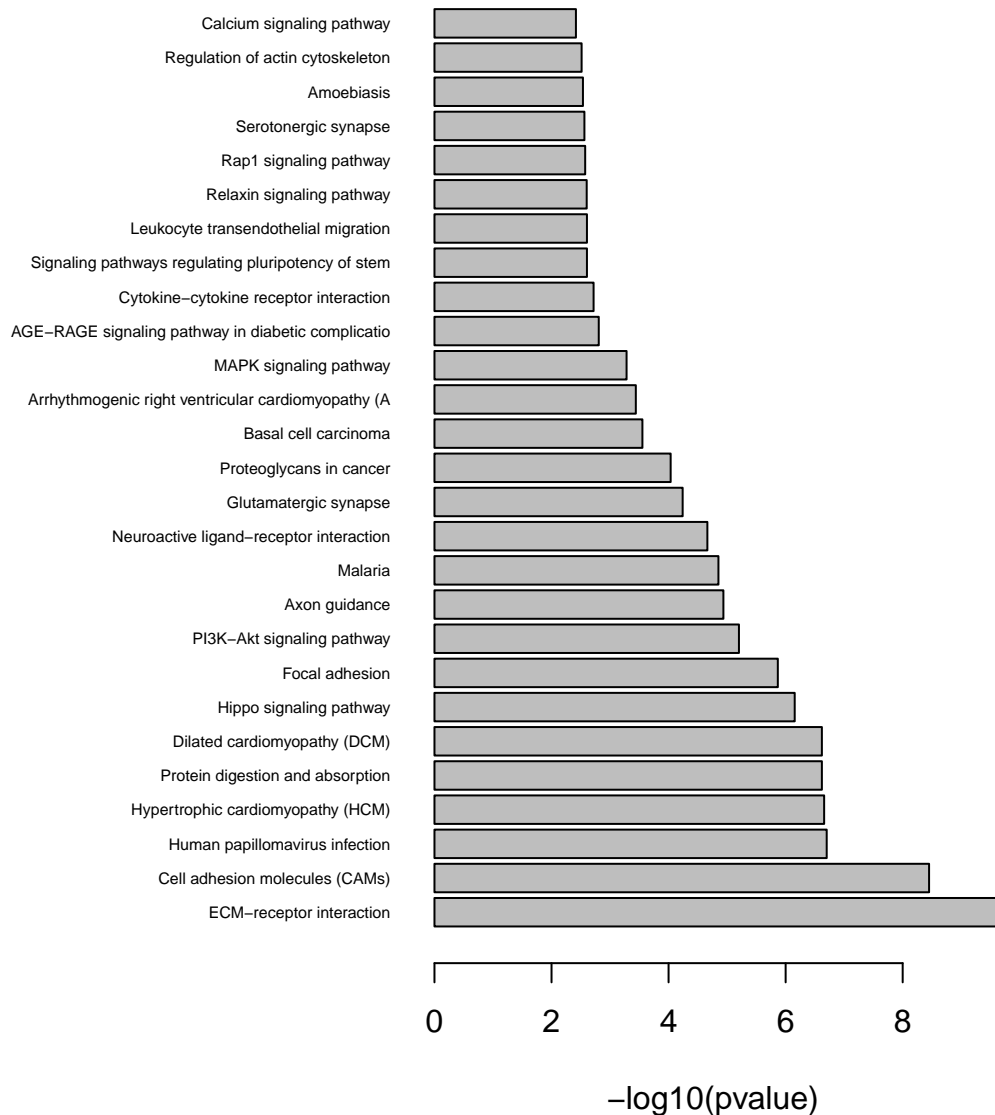

Supplement: DATASET S3 — GO-term analyses of GATA3-expressing and unscratched pHAs versus EGFP-expressing and unscratched pHAs in 2D cultures. [file Data_Sheet_3.ZIP › GO_term_analyses_GATA3u_vs_GFPu/clusterProfiler/pVal_CP_enrichKEGGS.pdf]

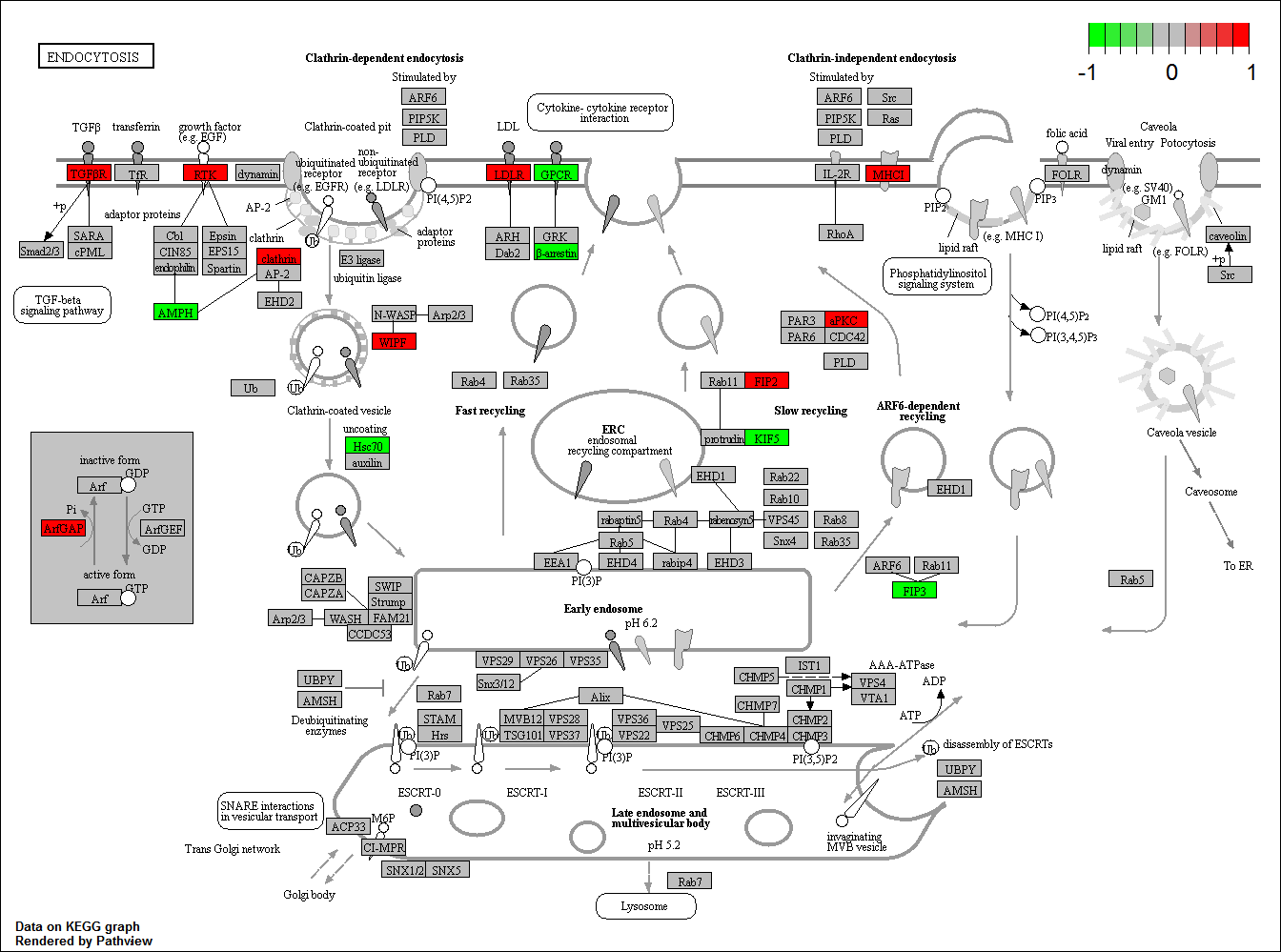

Supplement: DATASET S3 — GO-term analyses of GATA3-expressing and unscratched pHAs versus EGFP-expressing and unscratched pHAs in 2D cultures. [file Data_Sheet_3.ZIP › GO_term_analyses_GATA3u_vs_GFPu/GOSeq/hsa04144.Endocytosis.png]

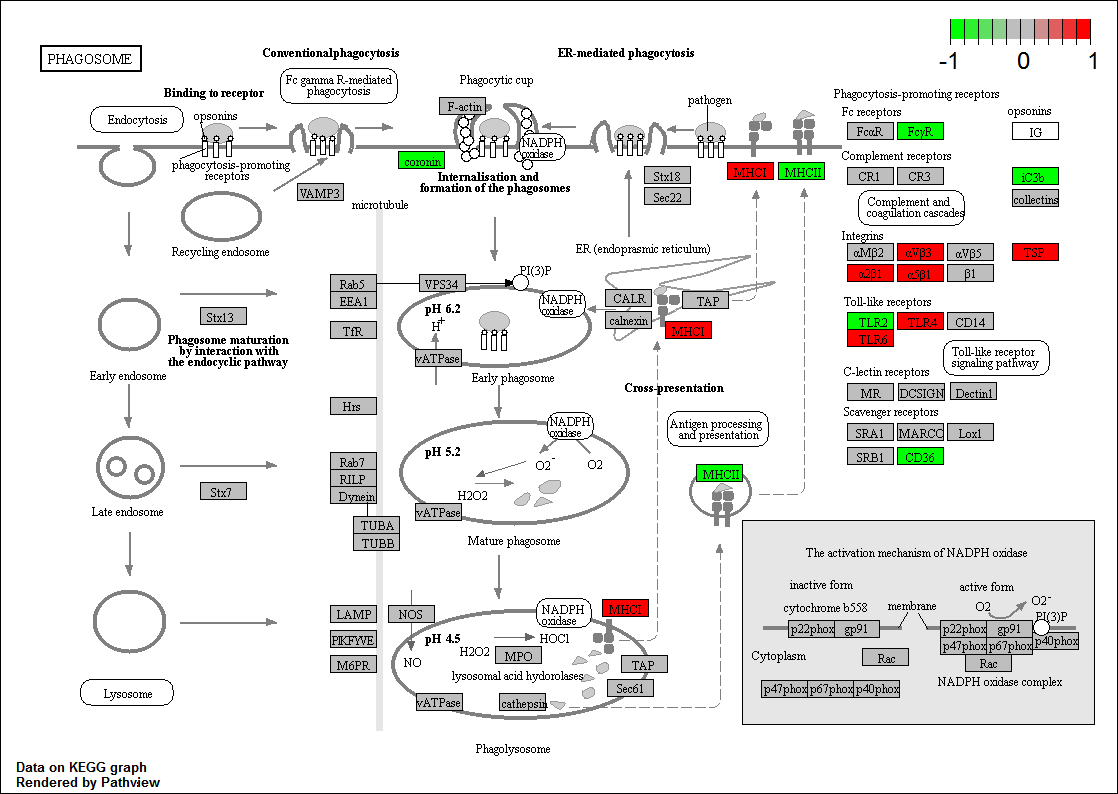

Supplement: DATASET S3 — GO-term analyses of GATA3-expressing and unscratched pHAs versus EGFP-expressing and unscratched pHAs in 2D cultures. [file Data_Sheet_3.ZIP › GO_term_analyses_GATA3u_vs_GFPu/GOSeq/hsa04145.Phagosome.png]

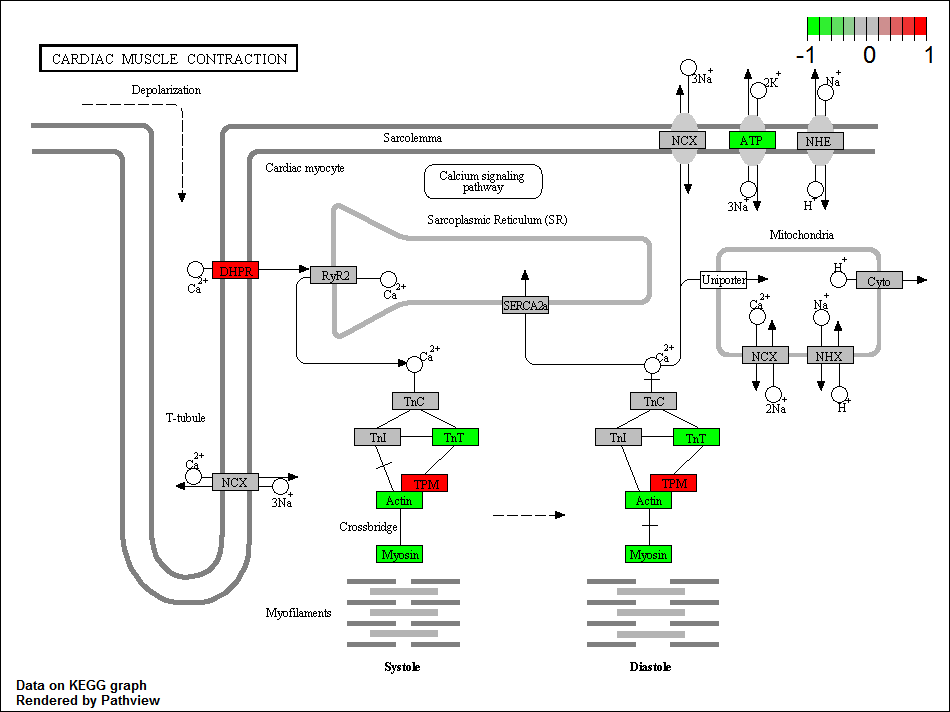

Supplement: DATASET S3 — GO-term analyses of GATA3-expressing and unscratched pHAs versus EGFP-expressing and unscratched pHAs in 2D cultures. [file Data_Sheet_3.ZIP › GO_term_analyses_GATA3u_vs_GFPu/GOSeq/hsa04260.Cardiacmusclecontraction.png]

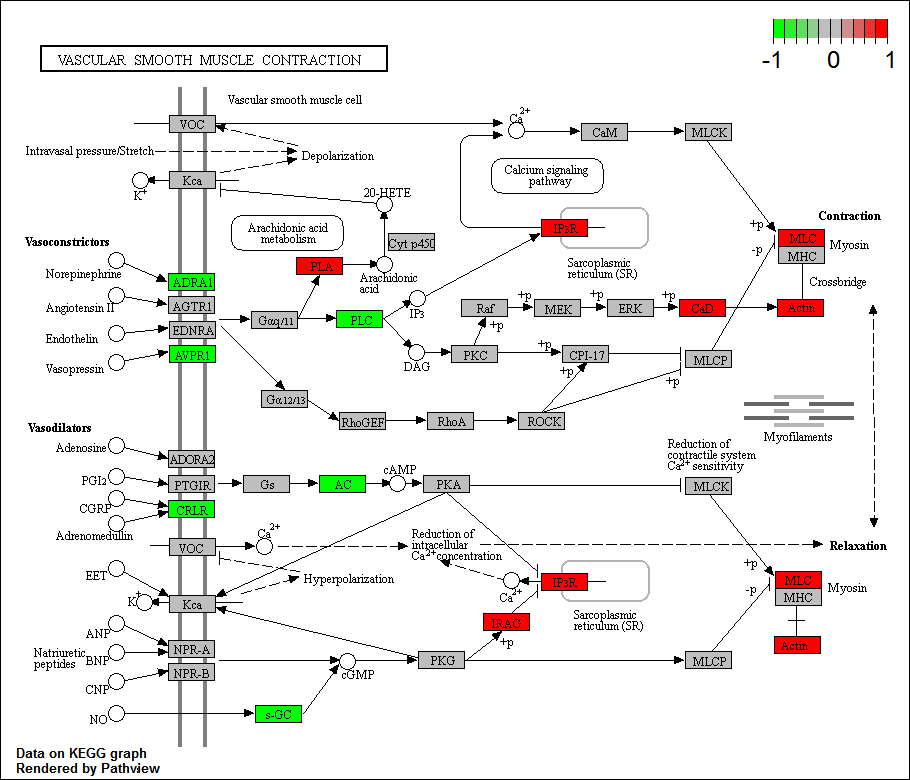

Supplement: DATASET S3 — GO-term analyses of GATA3-expressing and unscratched pHAs versus EGFP-expressing and unscratched pHAs in 2D cultures. [file Data_Sheet_3.ZIP › GO_term_analyses_GATA3u_vs_GFPu/GOSeq/hsa04270.Vascularsmoothmusclecontraction.png]

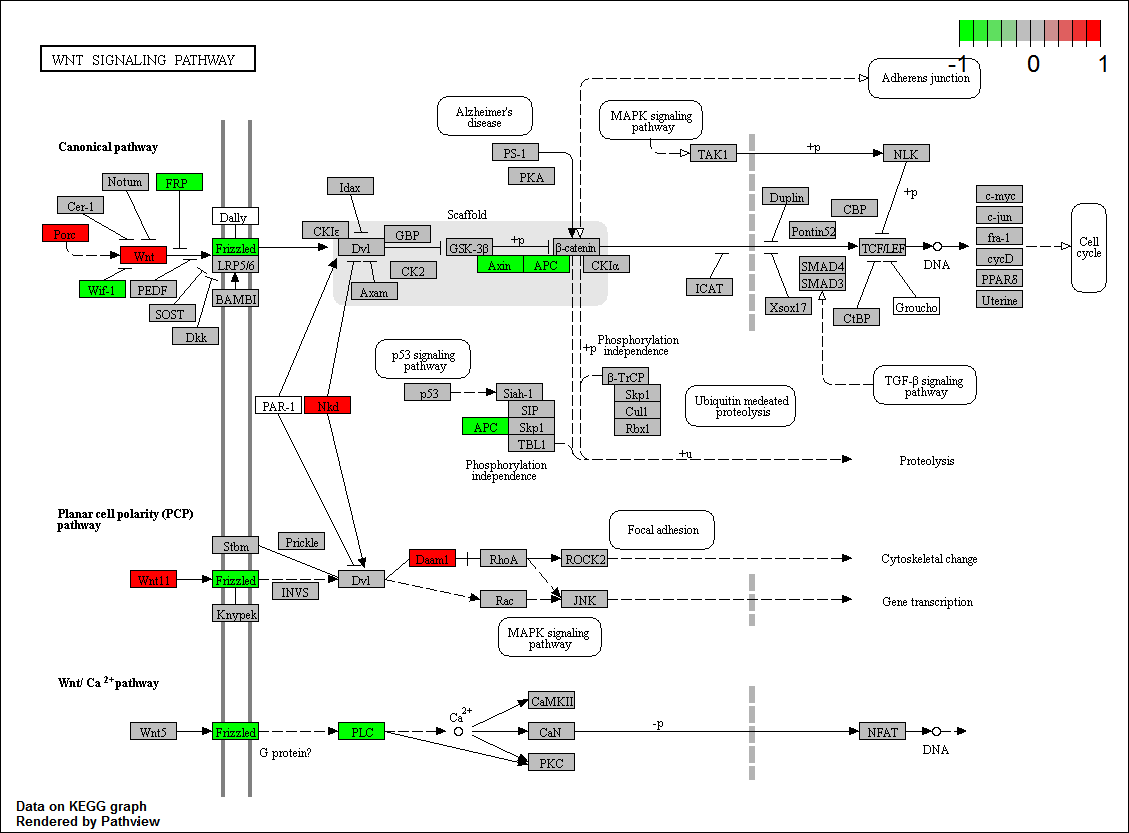

Supplement: DATASET S3 — GO-term analyses of GATA3-expressing and unscratched pHAs versus EGFP-expressing and unscratched pHAs in 2D cultures. [file Data_Sheet_3.ZIP › GO_term_analyses_GATA3u_vs_GFPu/GOSeq/hsa04310.Wntsignalingpathway.png]

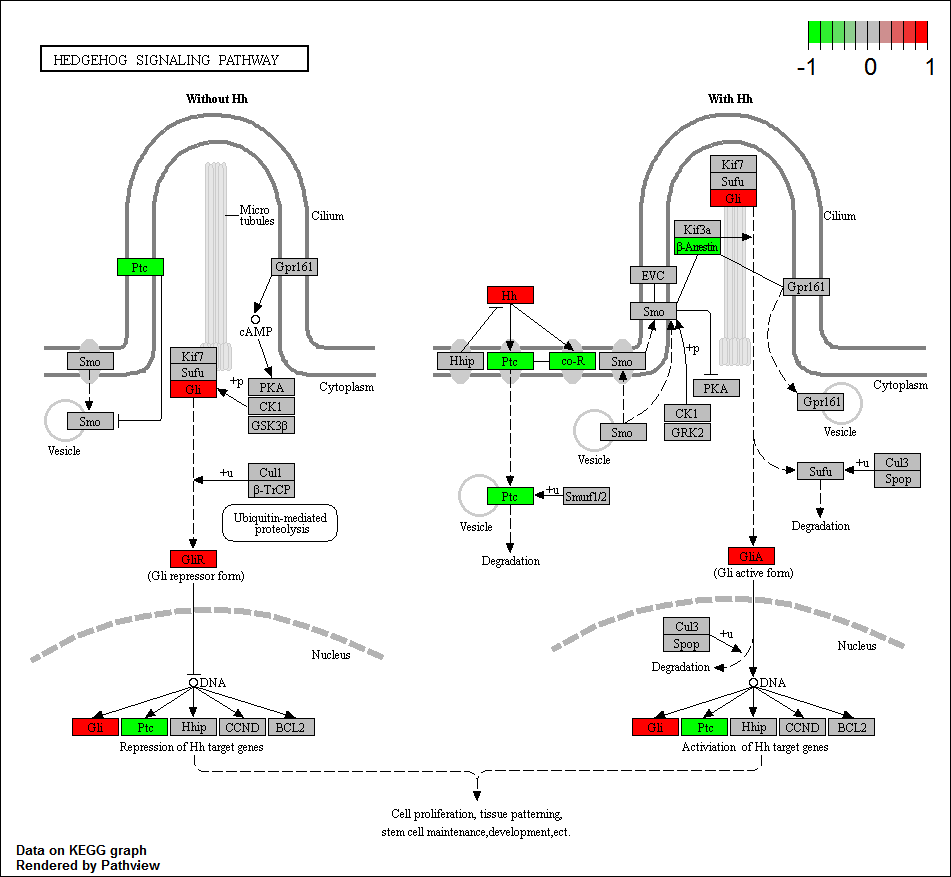

Supplement: DATASET S3 — GO-term analyses of GATA3-expressing and unscratched pHAs versus EGFP-expressing and unscratched pHAs in 2D cultures. [file Data_Sheet_3.ZIP › GO_term_analyses_GATA3u_vs_GFPu/GOSeq/hsa04340.Hedgehogsignalingpathway.png]

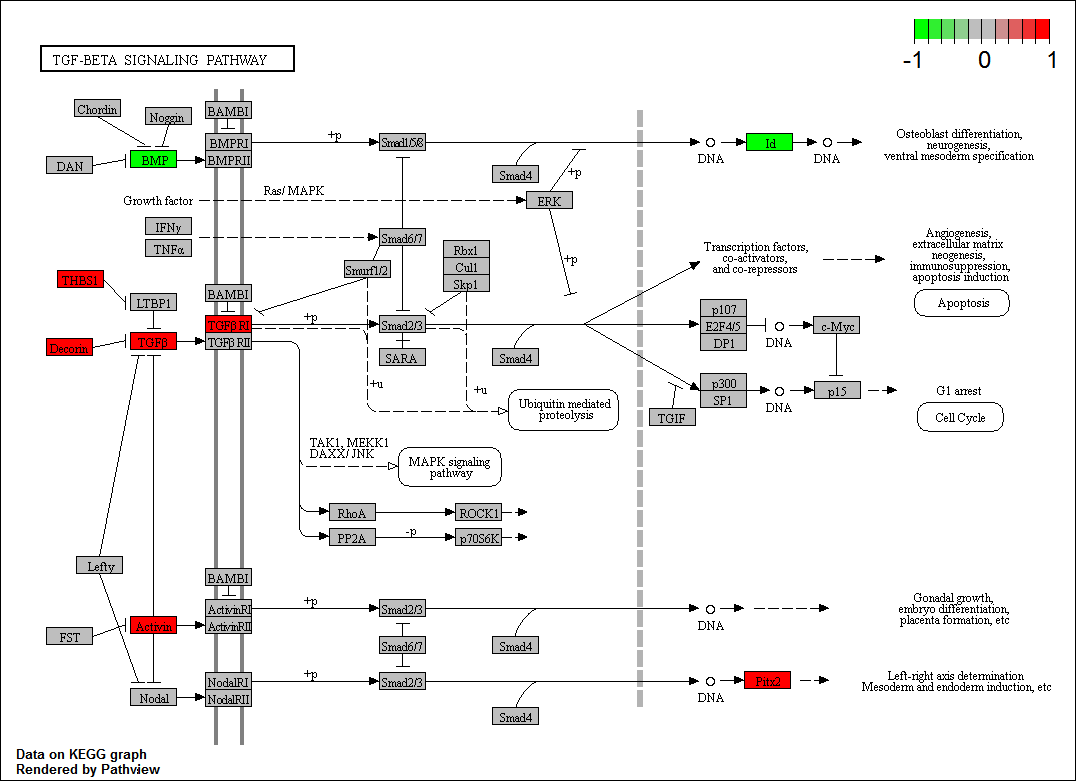

Supplement: DATASET S3 — GO-term analyses of GATA3-expressing and unscratched pHAs versus EGFP-expressing and unscratched pHAs in 2D cultures. [file Data_Sheet_3.ZIP › GO_term_analyses_GATA3u_vs_GFPu/GOSeq/hsa04350.TGF-betasignalingpathway.png]

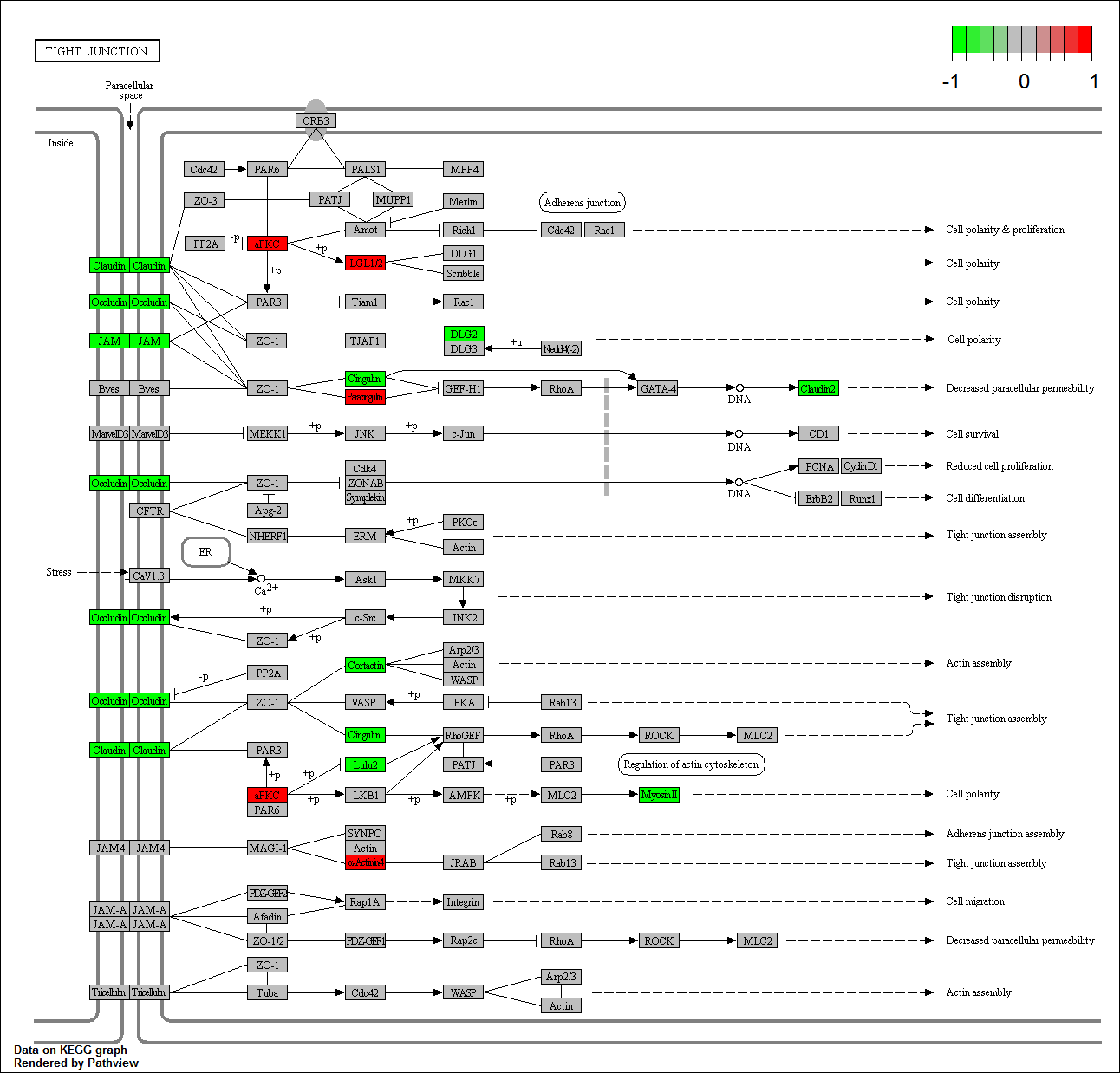

Supplement: DATASET S3 — GO-term analyses of GATA3-expressing and unscratched pHAs versus EGFP-expressing and unscratched pHAs in 2D cultures. [file Data_Sheet_3.ZIP › GO_term_analyses_GATA3u_vs_GFPu/GOSeq/hsa04530.Tightjunction.png]

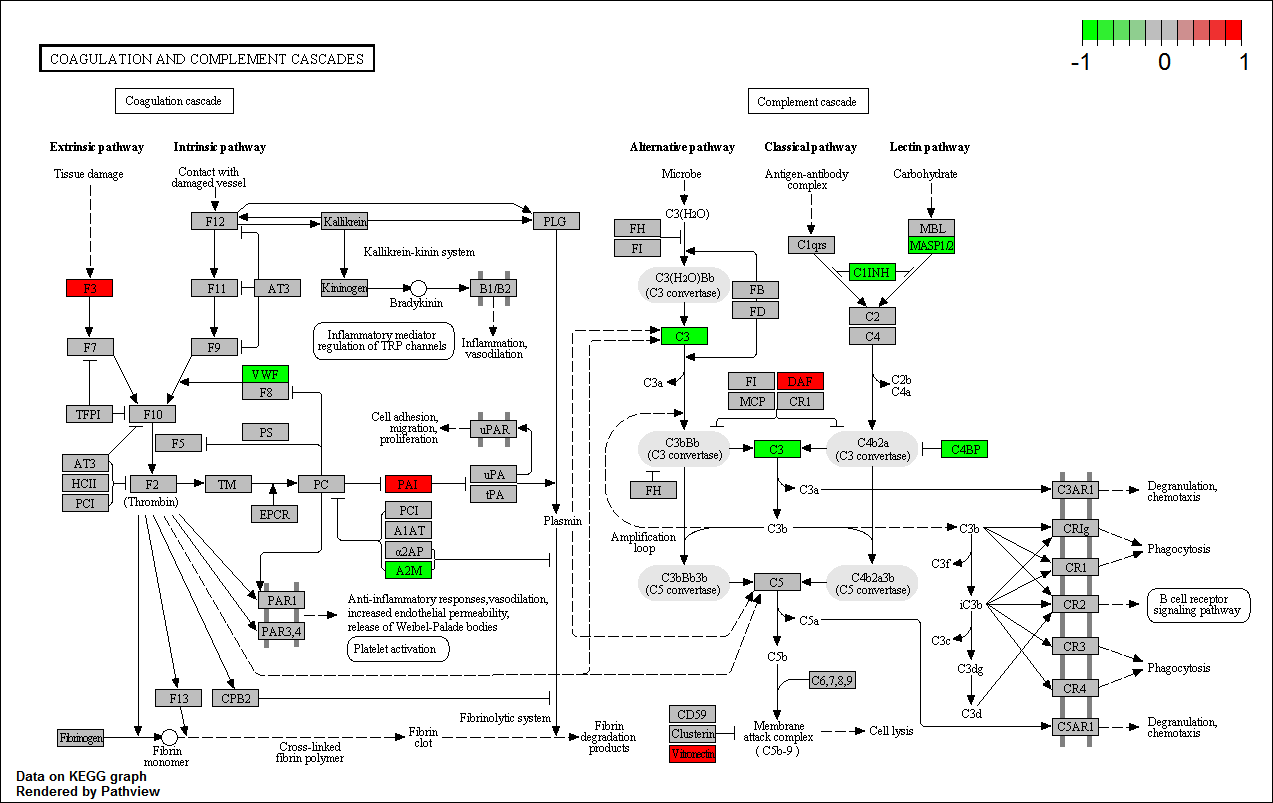

Supplement: DATASET S3 — GO-term analyses of GATA3-expressing and unscratched pHAs versus EGFP-expressing and unscratched pHAs in 2D cultures. [file Data_Sheet_3.ZIP › GO_term_analyses_GATA3u_vs_GFPu/GOSeq/hsa04610.Complementandcoagulationcascades.png]

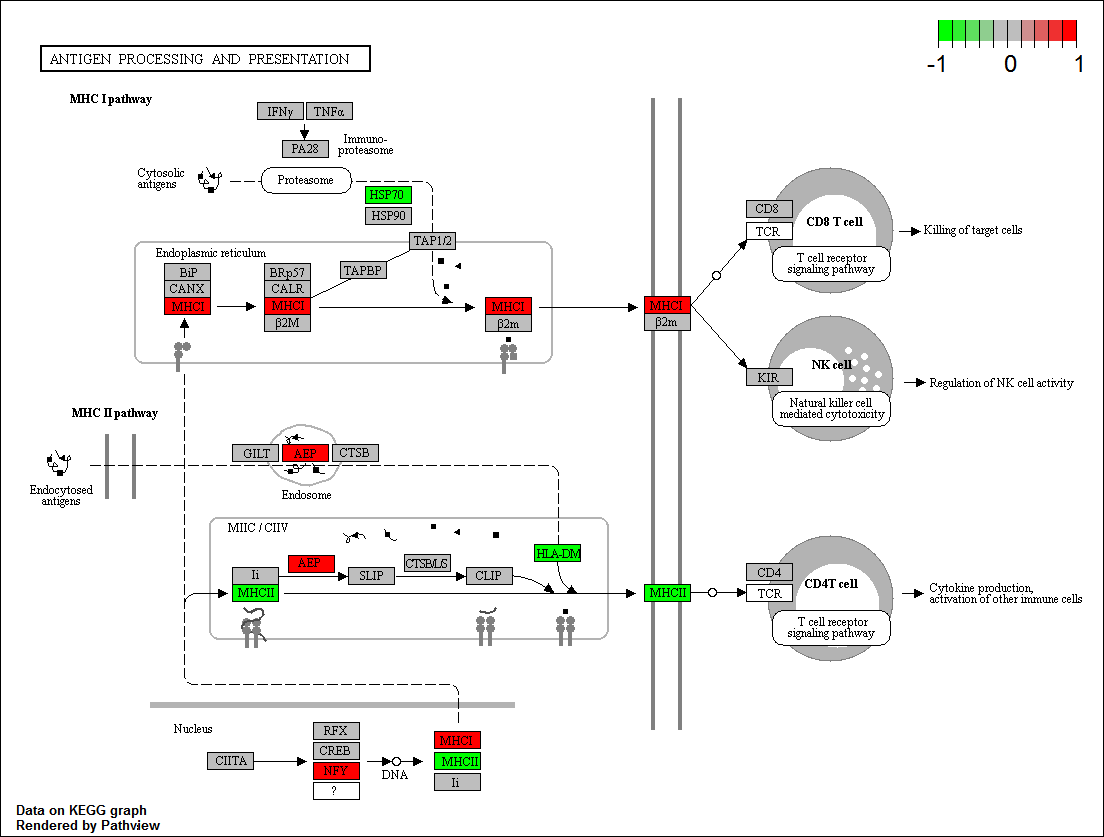

Supplement: DATASET S3 — GO-term analyses of GATA3-expressing and unscratched pHAs versus EGFP-expressing and unscratched pHAs in 2D cultures. [file Data_Sheet_3.ZIP › GO_term_analyses_GATA3u_vs_GFPu/GOSeq/hsa04612.Antigenprocessingandpresentation.png]

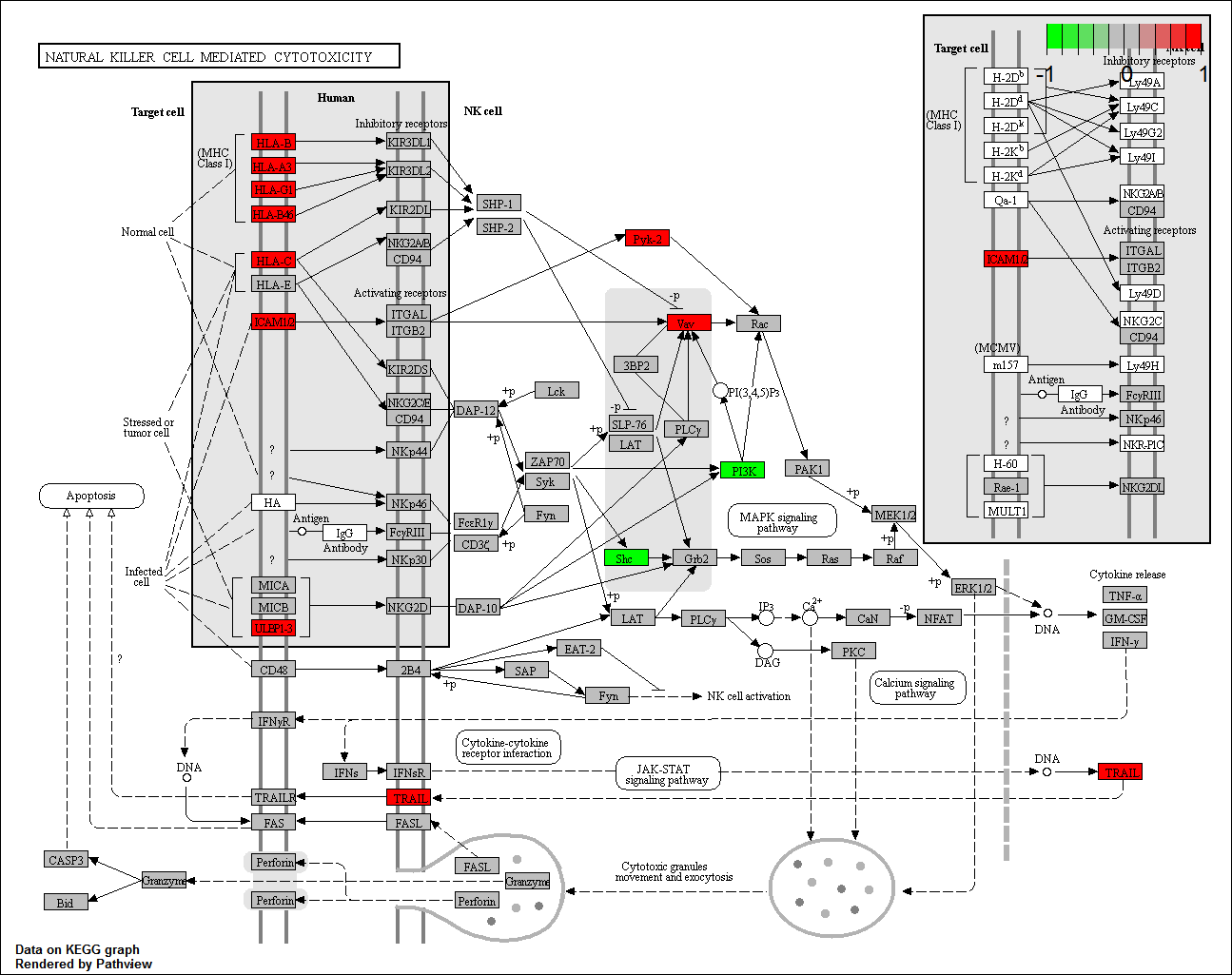

Supplement: DATASET S3 — GO-term analyses of GATA3-expressing and unscratched pHAs versus EGFP-expressing and unscratched pHAs in 2D cultures. [file Data_Sheet_3.ZIP › GO_term_analyses_GATA3u_vs_GFPu/GOSeq/hsa04650.Naturalkillercellmediatedcytotoxicity.png]

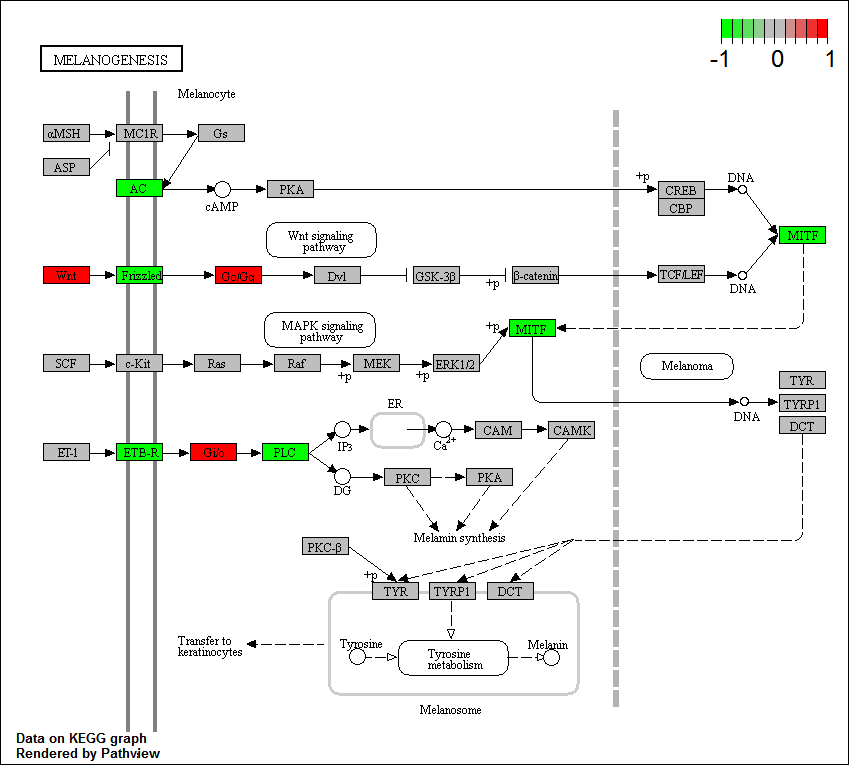

Supplement: DATASET S3 — GO-term analyses of GATA3-expressing and unscratched pHAs versus EGFP-expressing and unscratched pHAs in 2D cultures. [file Data_Sheet_3.ZIP › GO_term_analyses_GATA3u_vs_GFPu/GOSeq/hsa04916.Melanogenesis.png]

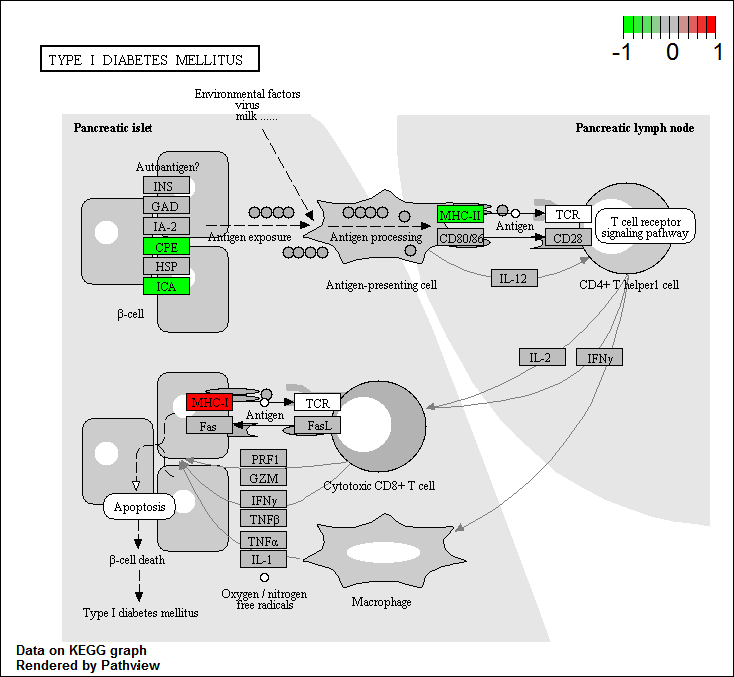

Supplement: DATASET S3 — GO-term analyses of GATA3-expressing and unscratched pHAs versus EGFP-expressing and unscratched pHAs in 2D cultures. [file Data_Sheet_3.ZIP › GO_term_analyses_GATA3u_vs_GFPu/GOSeq/hsa04940.TypeIdiabetesmellitus.png]

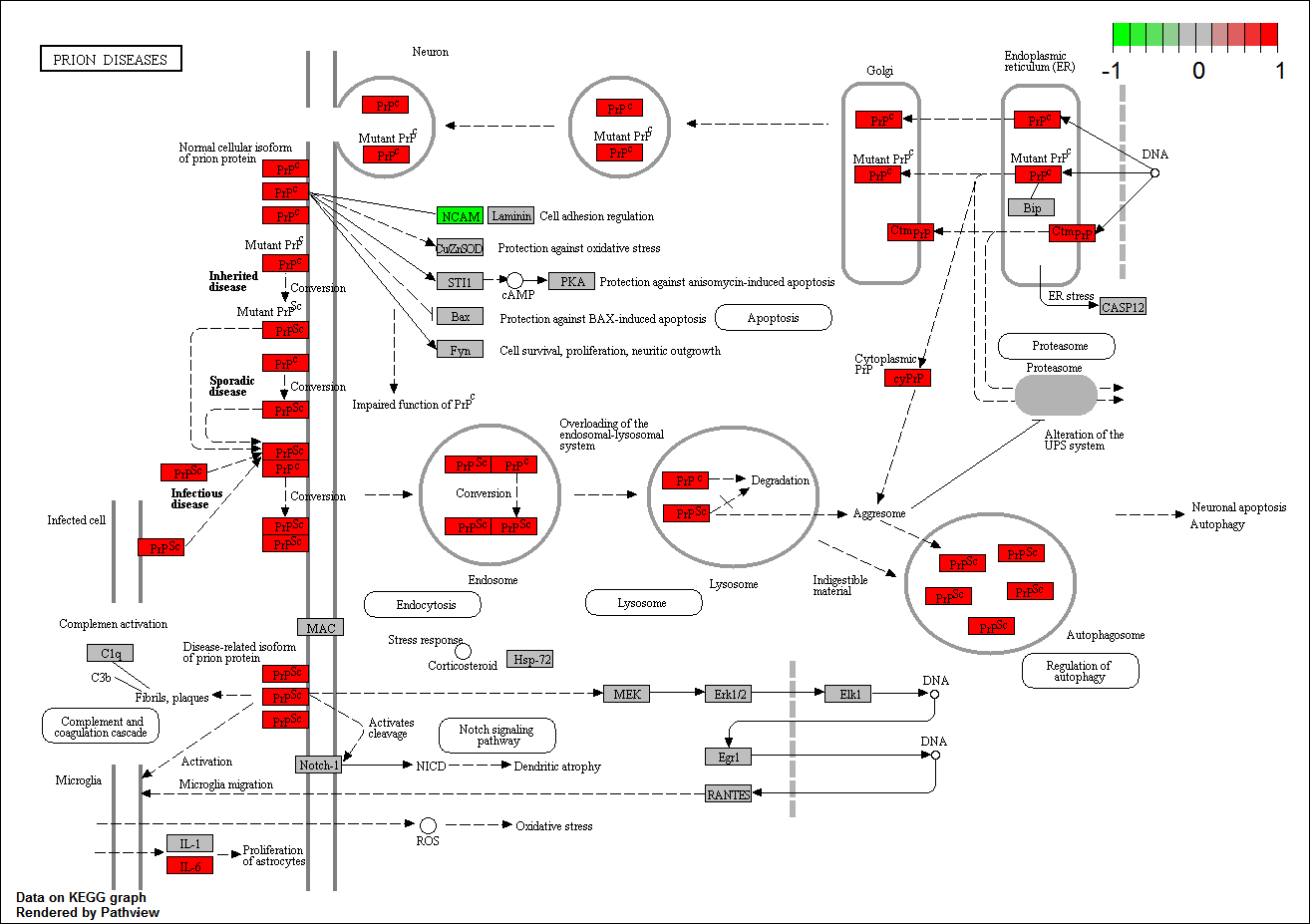

Supplement: DATASET S3 — GO-term analyses of GATA3-expressing and unscratched pHAs versus EGFP-expressing and unscratched pHAs in 2D cultures. [file Data_Sheet_3.ZIP › GO_term_analyses_GATA3u_vs_GFPu/GOSeq/hsa05020.Priondiseases.png]

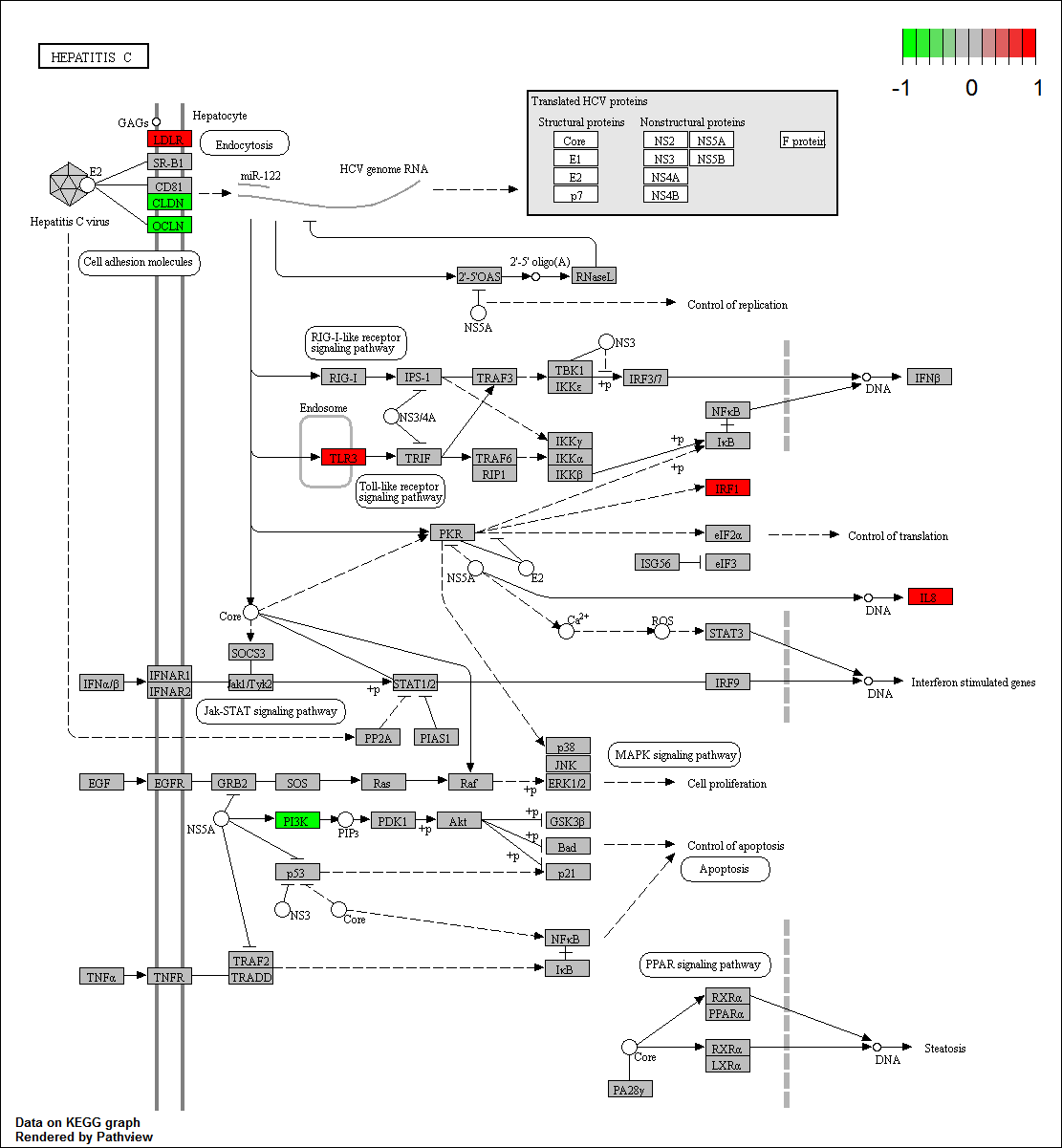

Supplement: DATASET S3 — GO-term analyses of GATA3-expressing and unscratched pHAs versus EGFP-expressing and unscratched pHAs in 2D cultures. [file Data_Sheet_3.ZIP › GO_term_analyses_GATA3u_vs_GFPu/GOSeq/hsa05160.HepatitisC.png]

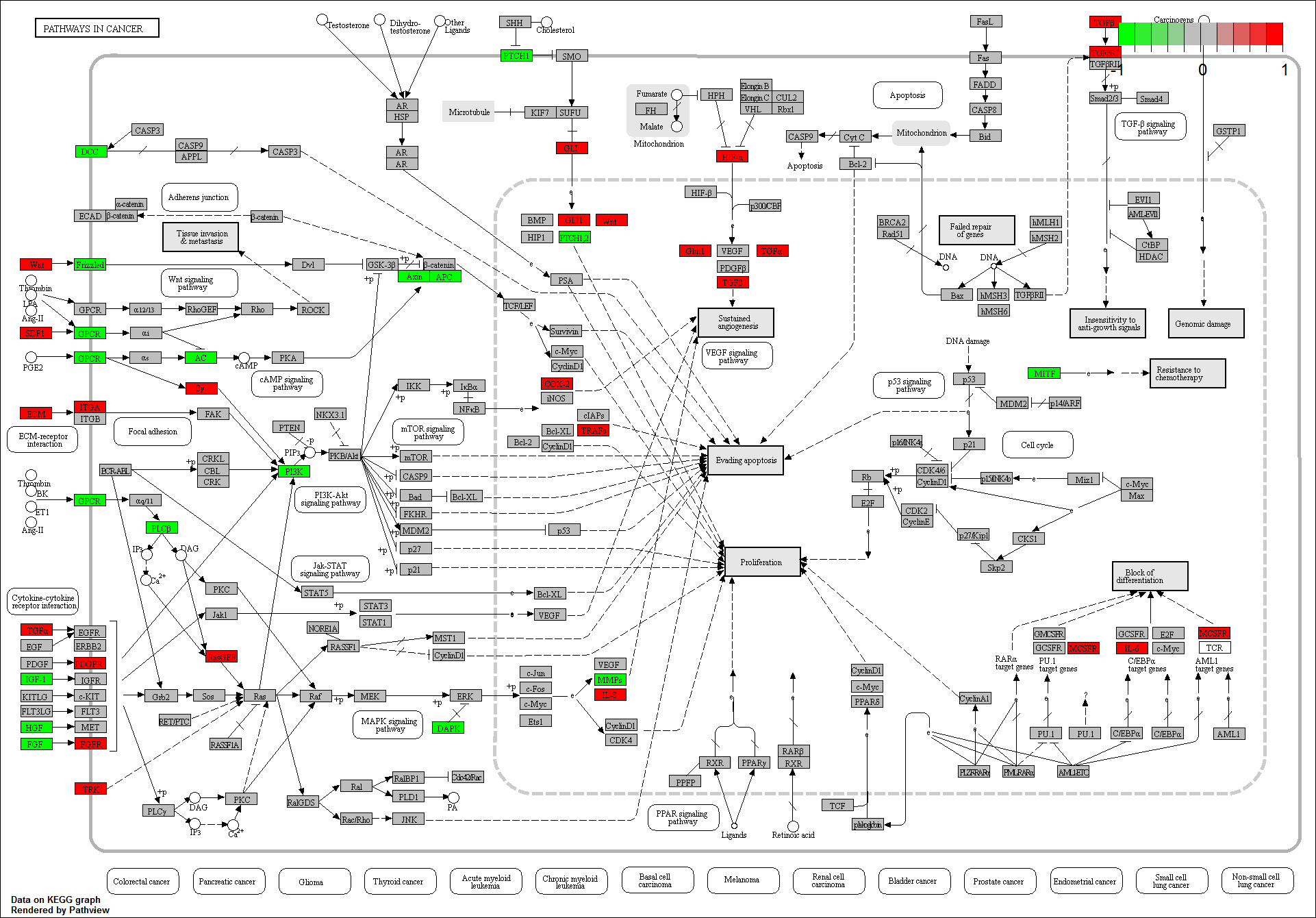

Supplement: DATASET S3 — GO-term analyses of GATA3-expressing and unscratched pHAs versus EGFP-expressing and unscratched pHAs in 2D cultures. [file Data_Sheet_3.ZIP › GO_term_analyses_GATA3u_vs_GFPu/GOSeq/hsa05200.Pathwaysincancer.png]

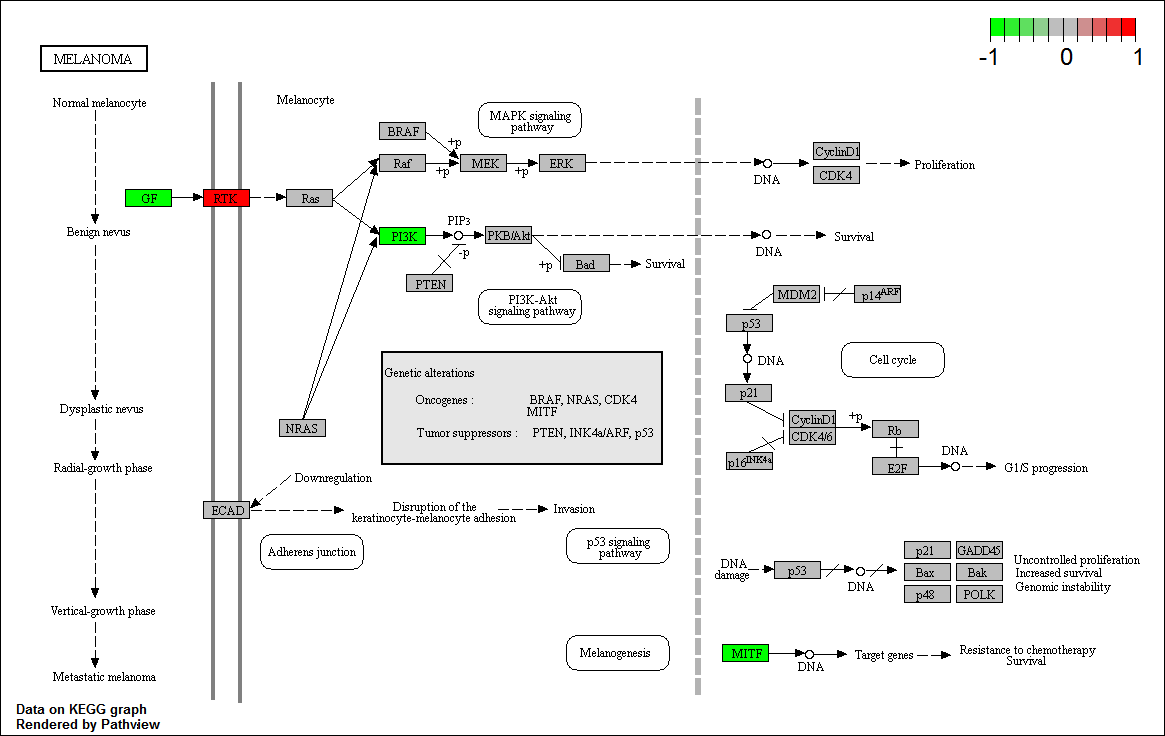

Supplement: DATASET S3 — GO-term analyses of GATA3-expressing and unscratched pHAs versus EGFP-expressing and unscratched pHAs in 2D cultures. [file Data_Sheet_3.ZIP › GO_term_analyses_GATA3u_vs_GFPu/GOSeq/hsa05218.Melanoma.png]

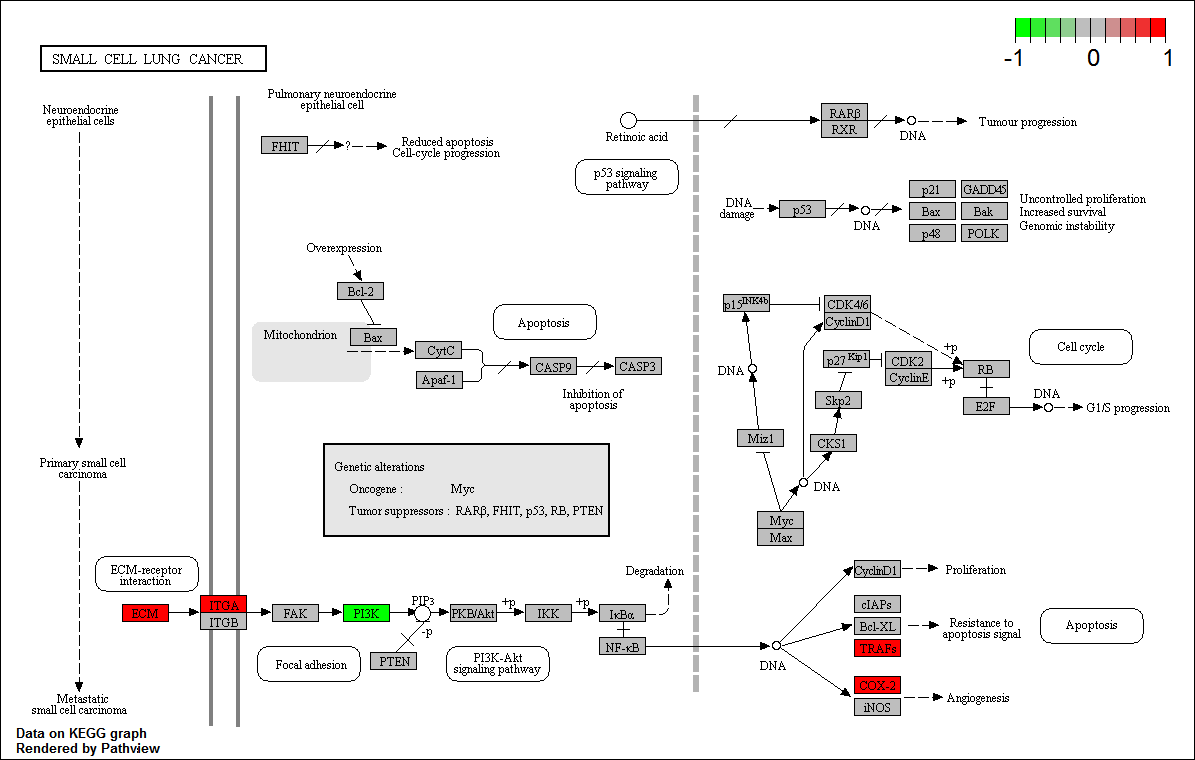

Supplement: DATASET S3 — GO-term analyses of GATA3-expressing and unscratched pHAs versus EGFP-expressing and unscratched pHAs in 2D cultures. [file Data_Sheet_3.ZIP › GO_term_analyses_GATA3u_vs_GFPu/GOSeq/hsa05222.Smallcelllungcancer.png]

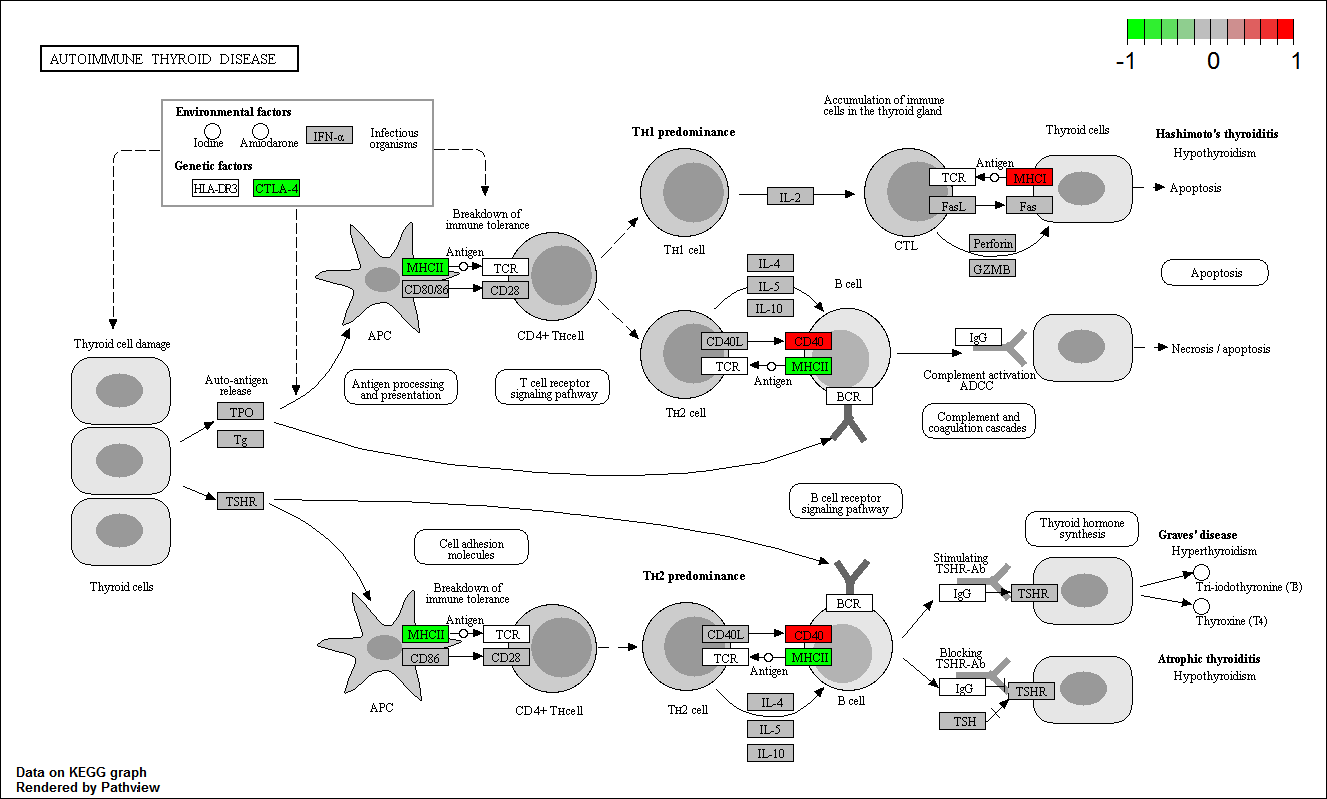

Supplement: DATASET S3 — GO-term analyses of GATA3-expressing and unscratched pHAs versus EGFP-expressing and unscratched pHAs in 2D cultures. [file Data_Sheet_3.ZIP › GO_term_analyses_GATA3u_vs_GFPu/GOSeq/hsa05320.Autoimmunethyroiddisease.png]

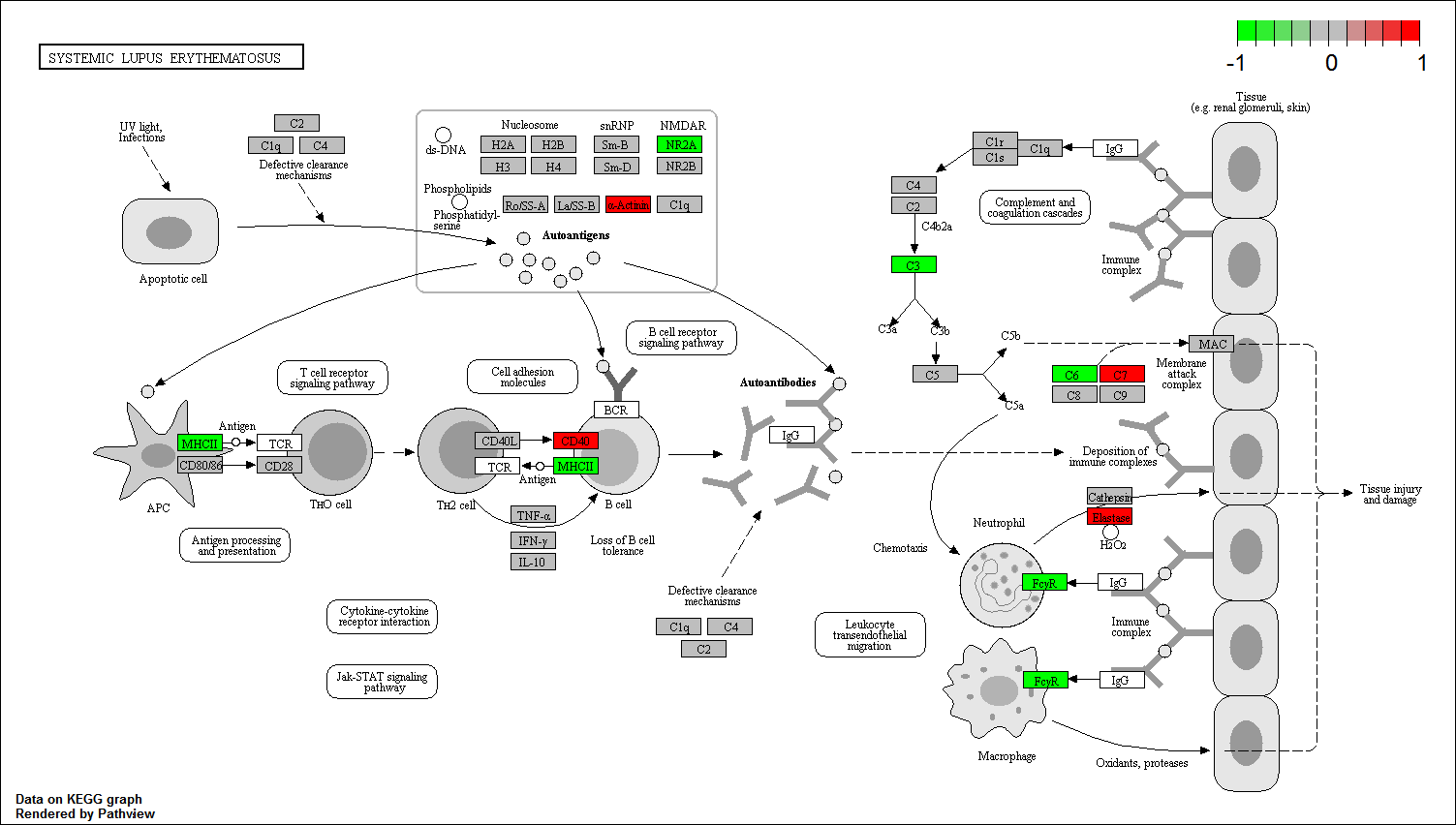

Supplement: DATASET S3 — GO-term analyses of GATA3-expressing and unscratched pHAs versus EGFP-expressing and unscratched pHAs in 2D cultures. [file Data_Sheet_3.ZIP › GO_term_analyses_GATA3u_vs_GFPu/GOSeq/hsa05322.Systemiclupuserythematosus.png]

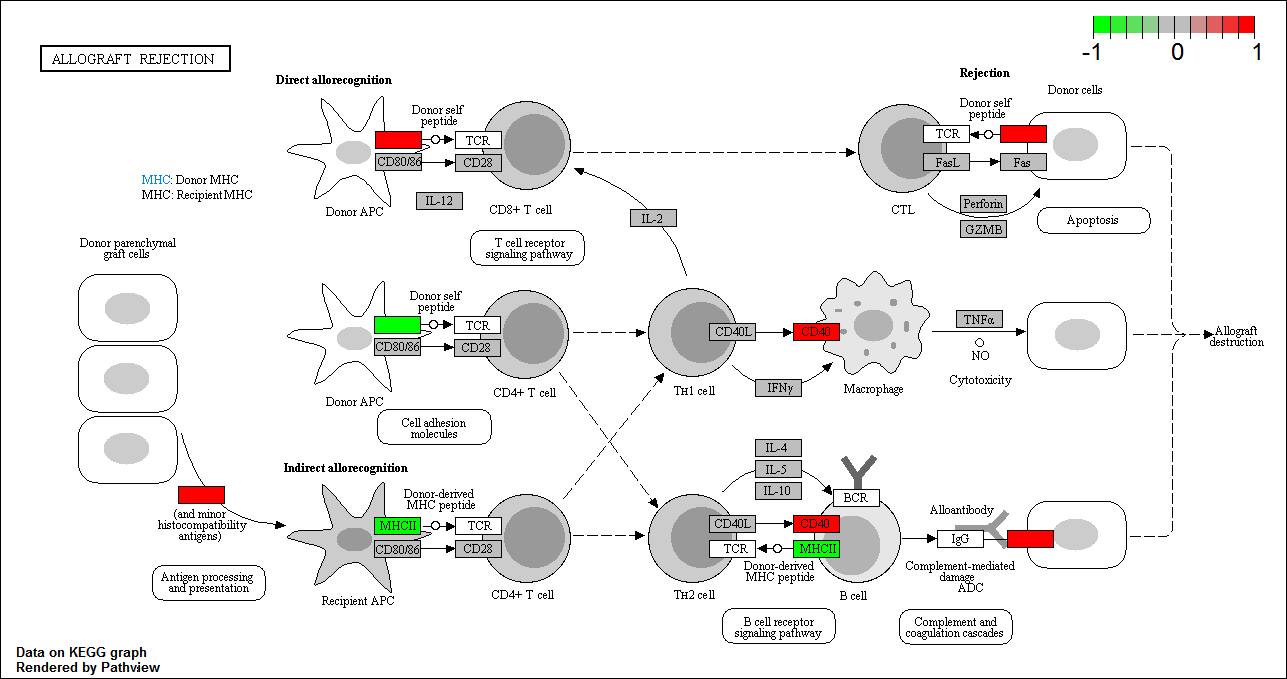

Supplement: DATASET S3 — GO-term analyses of GATA3-expressing and unscratched pHAs versus EGFP-expressing and unscratched pHAs in 2D cultures. [file Data_Sheet_3.ZIP › GO_term_analyses_GATA3u_vs_GFPu/GOSeq/hsa05330.Allograftrejection.png]

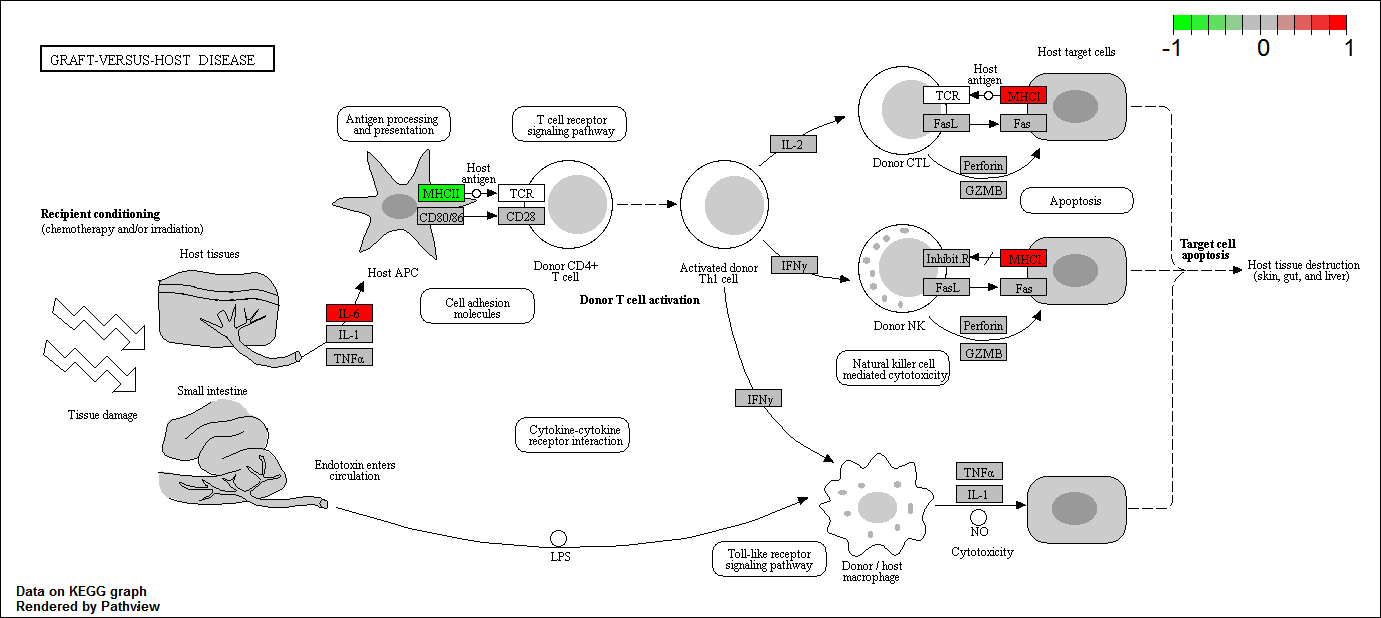

Supplement: DATASET S3 — GO-term analyses of GATA3-expressing and unscratched pHAs versus EGFP-expressing and unscratched pHAs in 2D cultures. [file Data_Sheet_3.ZIP › GO_term_analyses_GATA3u_vs_GFPu/GOSeq/hsa05332.Graft-versus-hostdisease.png]

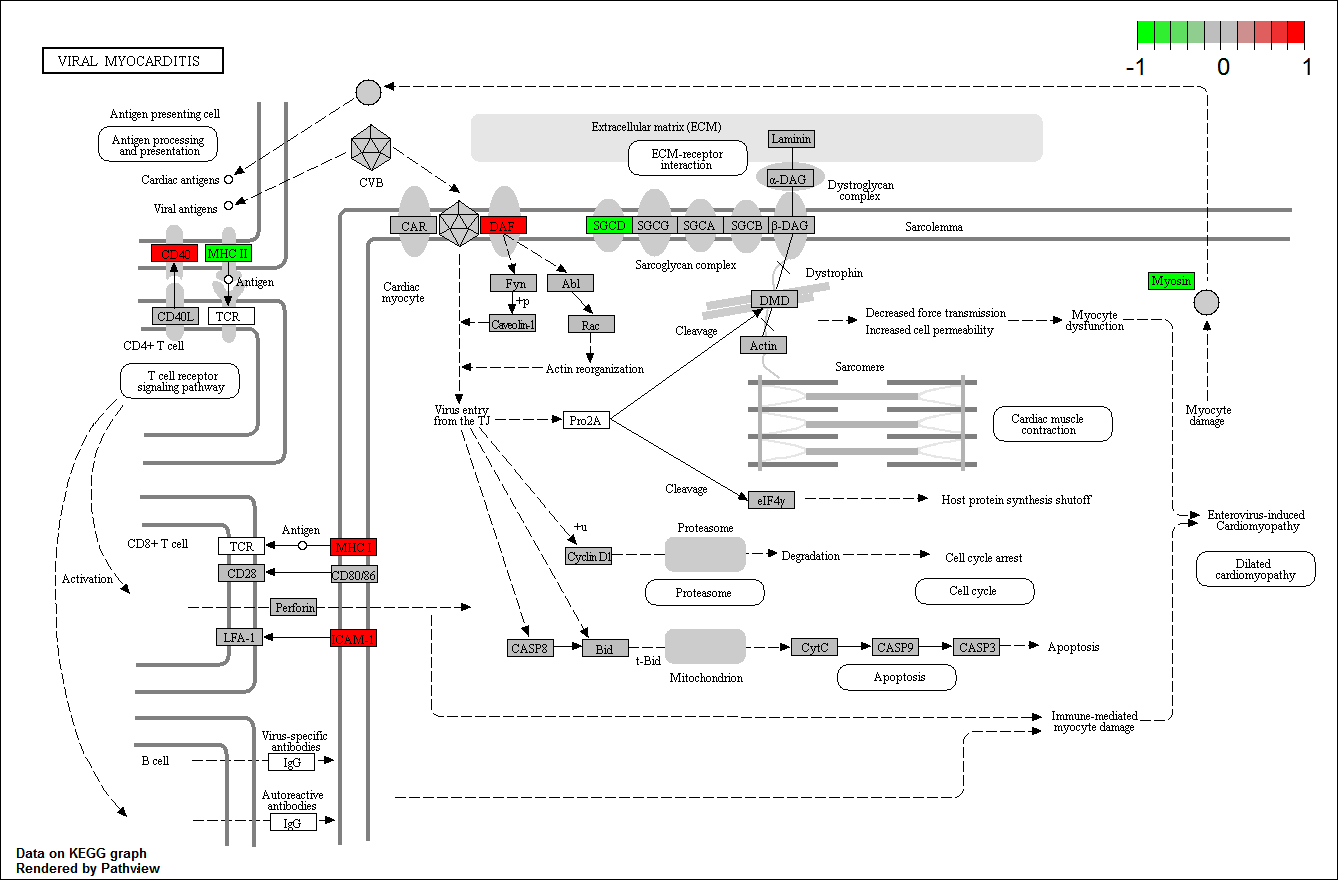

Supplement: DATASET S3 — GO-term analyses of GATA3-expressing and unscratched pHAs versus EGFP-expressing and unscratched pHAs in 2D cultures. [file Data_Sheet_3.ZIP › GO_term_analyses_GATA3u_vs_GFPu/GOSeq/hsa05416.Viralmyocarditis.png]

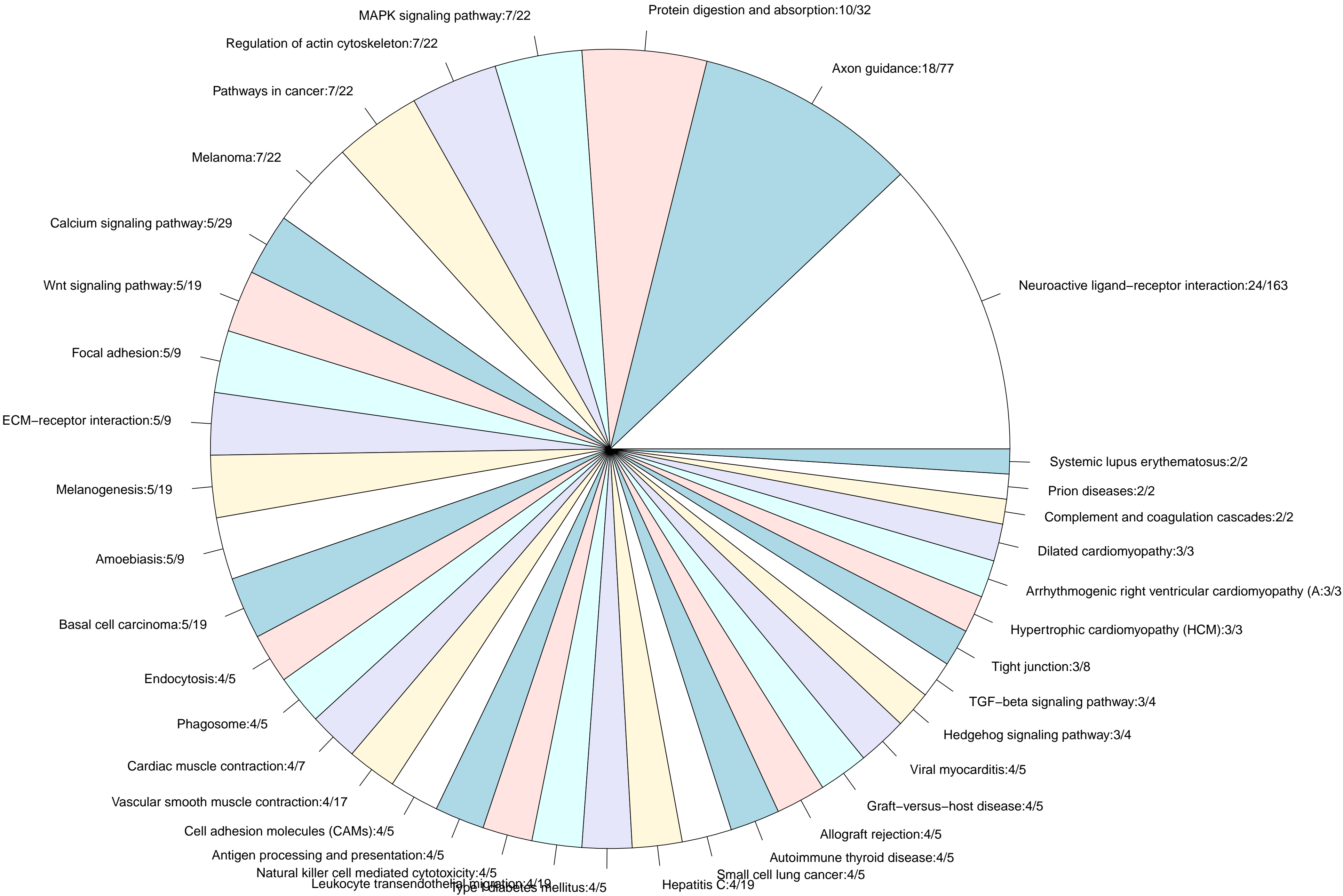

Supplement: DATASET S3 — GO-term analyses of GATA3-expressing and unscratched pHAs versus EGFP-expressing and unscratched pHAs in 2D cultures. [file Data_Sheet_3.ZIP › GO_term_analyses_GATA3u_vs_GFPu/GOSeq/KEGG_adjusted.GO.kegg.Over.BH.pdf]

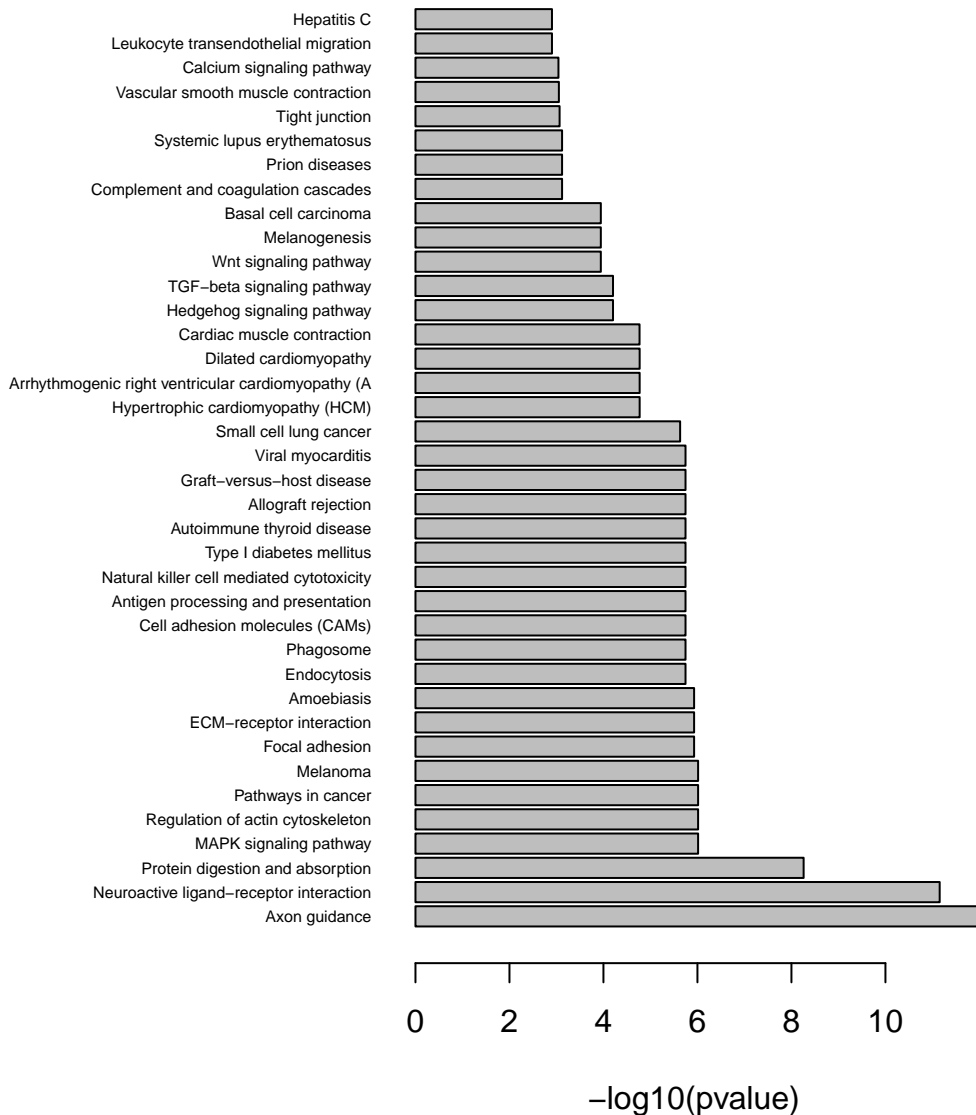

Supplement: DATASET S3 — GO-term analyses of GATA3-expressing and unscratched pHAs versus EGFP-expressing and unscratched pHAs in 2D cultures. [file Data_Sheet_3.ZIP › GO_term_analyses_GATA3u_vs_GFPu/GOSeq/pVal_KEGG_adjusted.GO.kegg.Over.BH.pdf]

GOstats\_BP\_Down\_pieChart

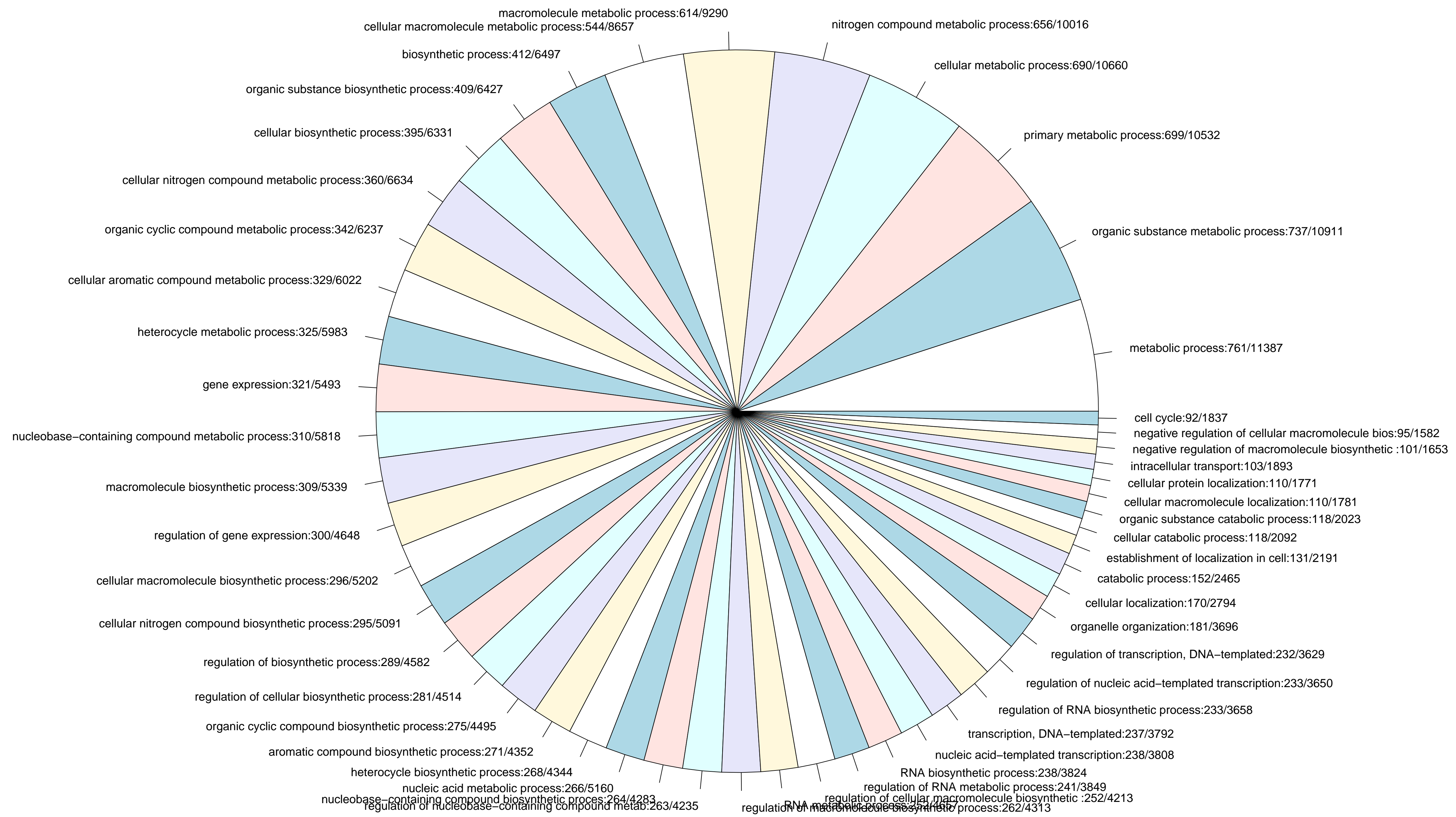

Supplement: DATASET S3 — GO-term analyses of GATA3-expressing and unscratched pHAs versus EGFP-expressing and unscratched pHAs in 2D cultures. [file Data_Sheet_3.ZIP › GO_term_analyses_GATA3u_vs_GFPu/GOstats/GOstats_BP_Down_pieChart.pdf]

GOstats\_BP\_Up\_pieChart

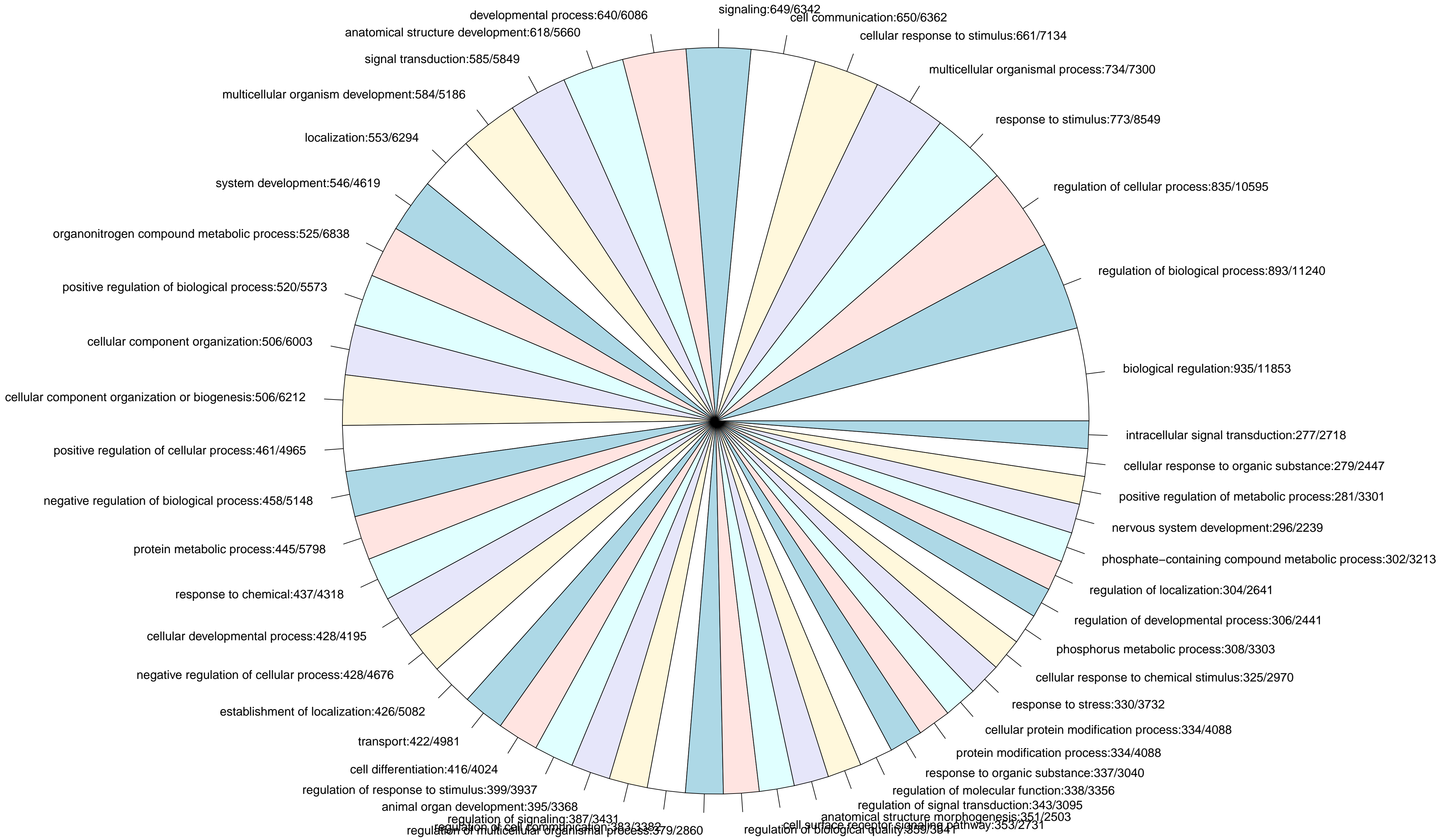

Supplement: DATASET S3 — GO-term analyses of GATA3-expressing and unscratched pHAs versus EGFP-expressing and unscratched pHAs in 2D cultures. [file Data_Sheet_3.ZIP › GO_term_analyses_GATA3u_vs_GFPu/GOstats/GOstats_BP_Up_pieChart.pdf]

### GOstats\_CC\_Down\_pieChart

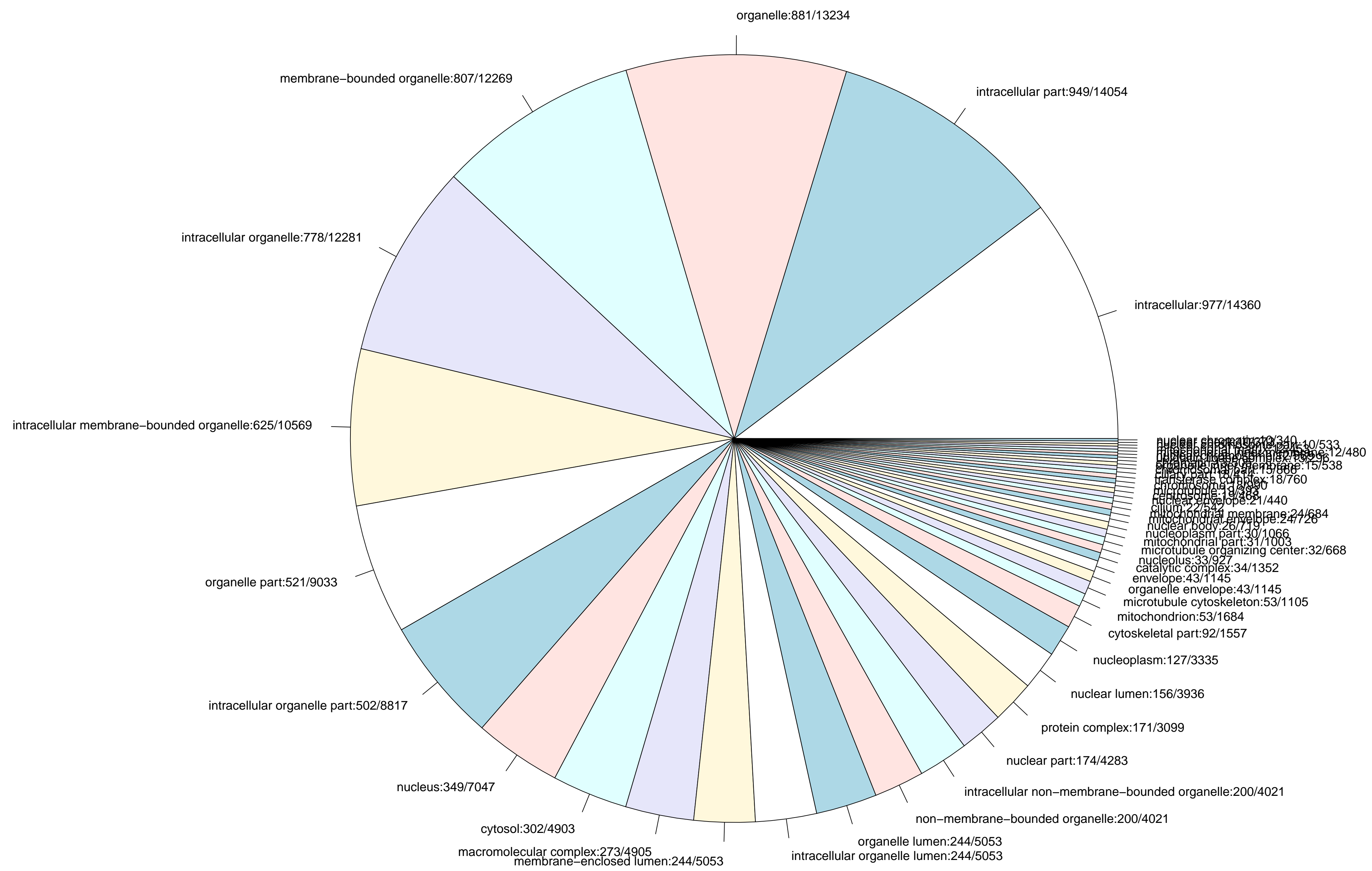

Supplement: DATASET S3 — GO-term analyses of GATA3-expressing and unscratched pHAs versus EGFP-expressing and unscratched pHAs in 2D cultures. [file Data_Sheet_3.ZIP › GO_term_analyses_GATA3u_vs_GFPu/GOstats/GOstats_CC_Down_pieChart.pdf]

GOstats\_CC\_Up\_pieChart

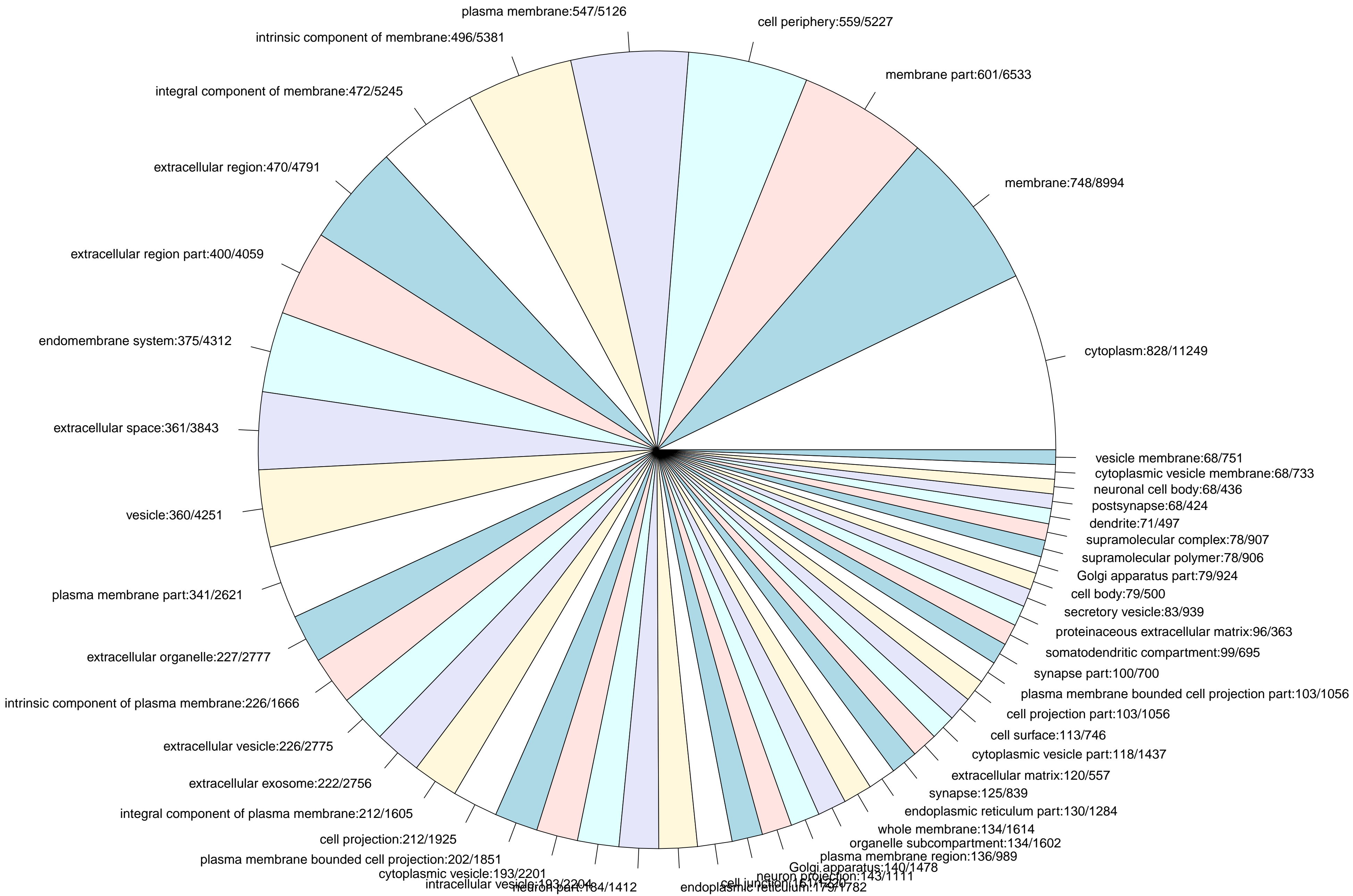

Supplement: DATASET S3 — GO-term analyses of GATA3-expressing and unscratched pHAs versus EGFP-expressing and unscratched pHAs in 2D cultures. [file Data_Sheet_3.ZIP › GO_term_analyses_GATA3u_vs_GFPu/GOstats/GOstats_CC_Up_pieChart.pdf]

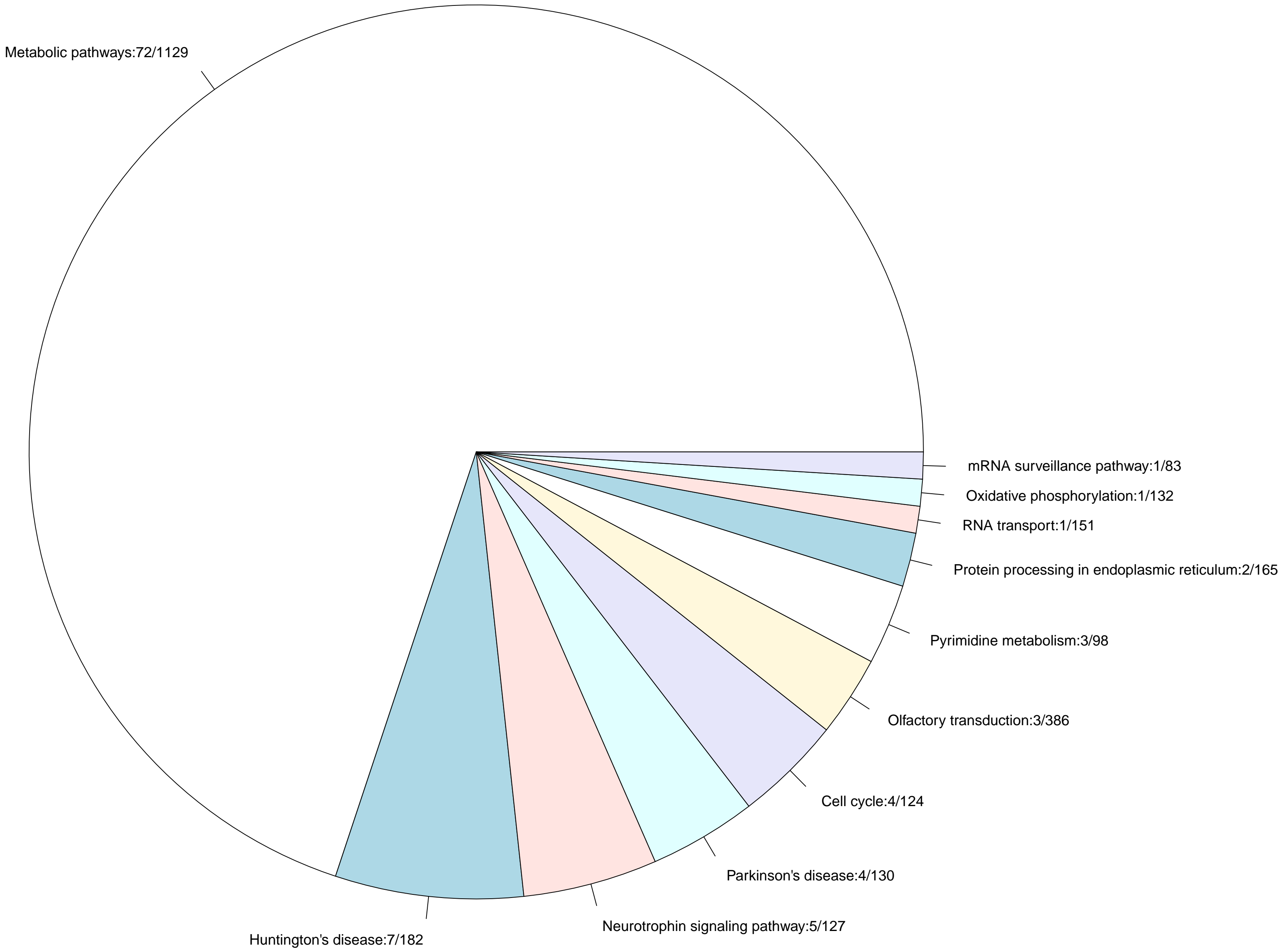

Supplement: DATASET S3 — GO-term analyses of GATA3-expressing and unscratched pHAs versus EGFP-expressing and unscratched pHAs in 2D cultures. [file Data_Sheet_3.ZIP › GO_term_analyses_GATA3u_vs_GFPu/GOstats/GOstats_kegg_Under.pdf]

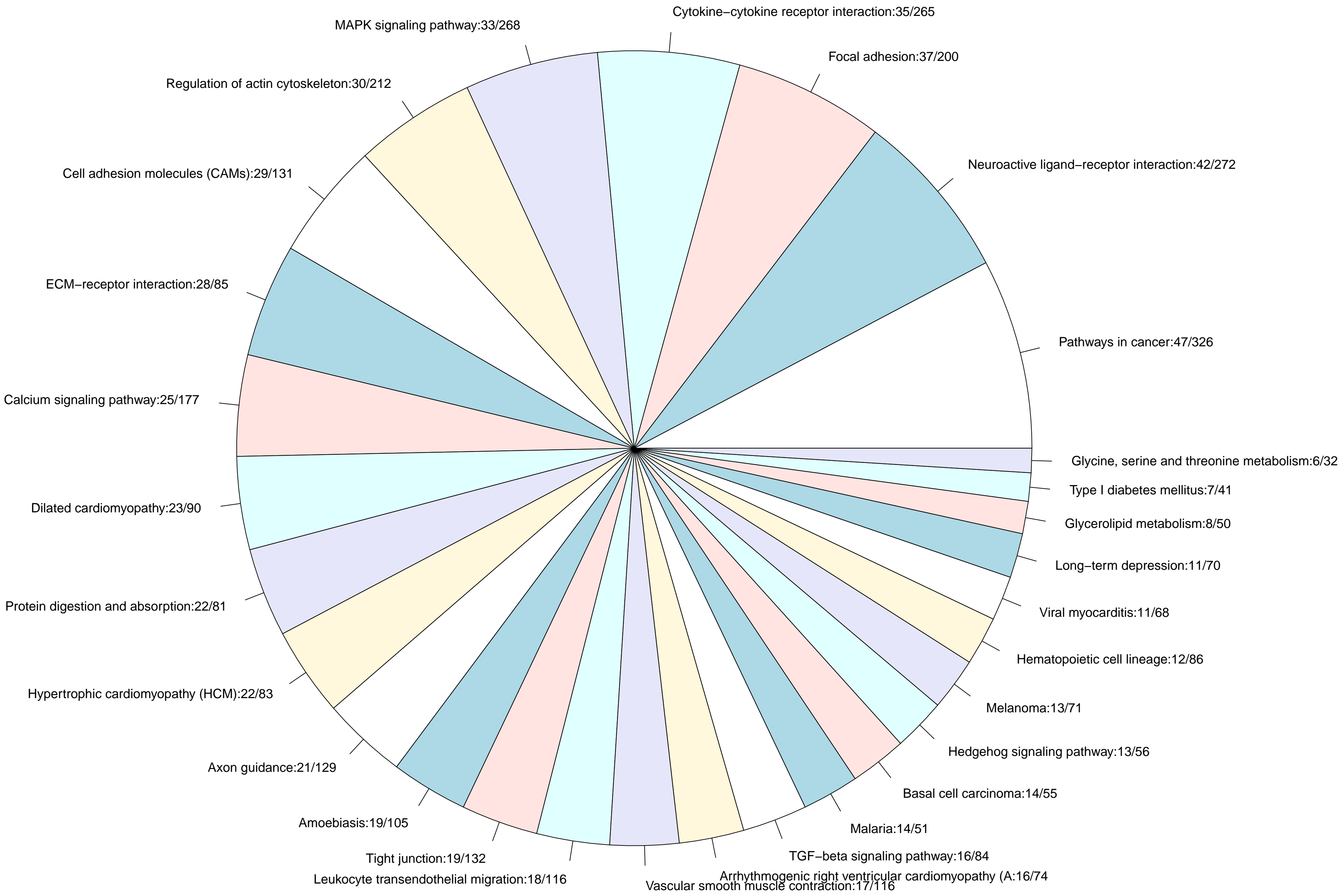

Supplement: DATASET S3 — GO-term analyses of GATA3-expressing and unscratched pHAs versus EGFP-expressing and unscratched pHAs in 2D cultures. [file Data_Sheet_3.ZIP › GO_term_analyses_GATA3u_vs_GFPu/GOstats/GOstats_kegg_Up.pdf]

### GOstats\_MF\_Down\_pieChart

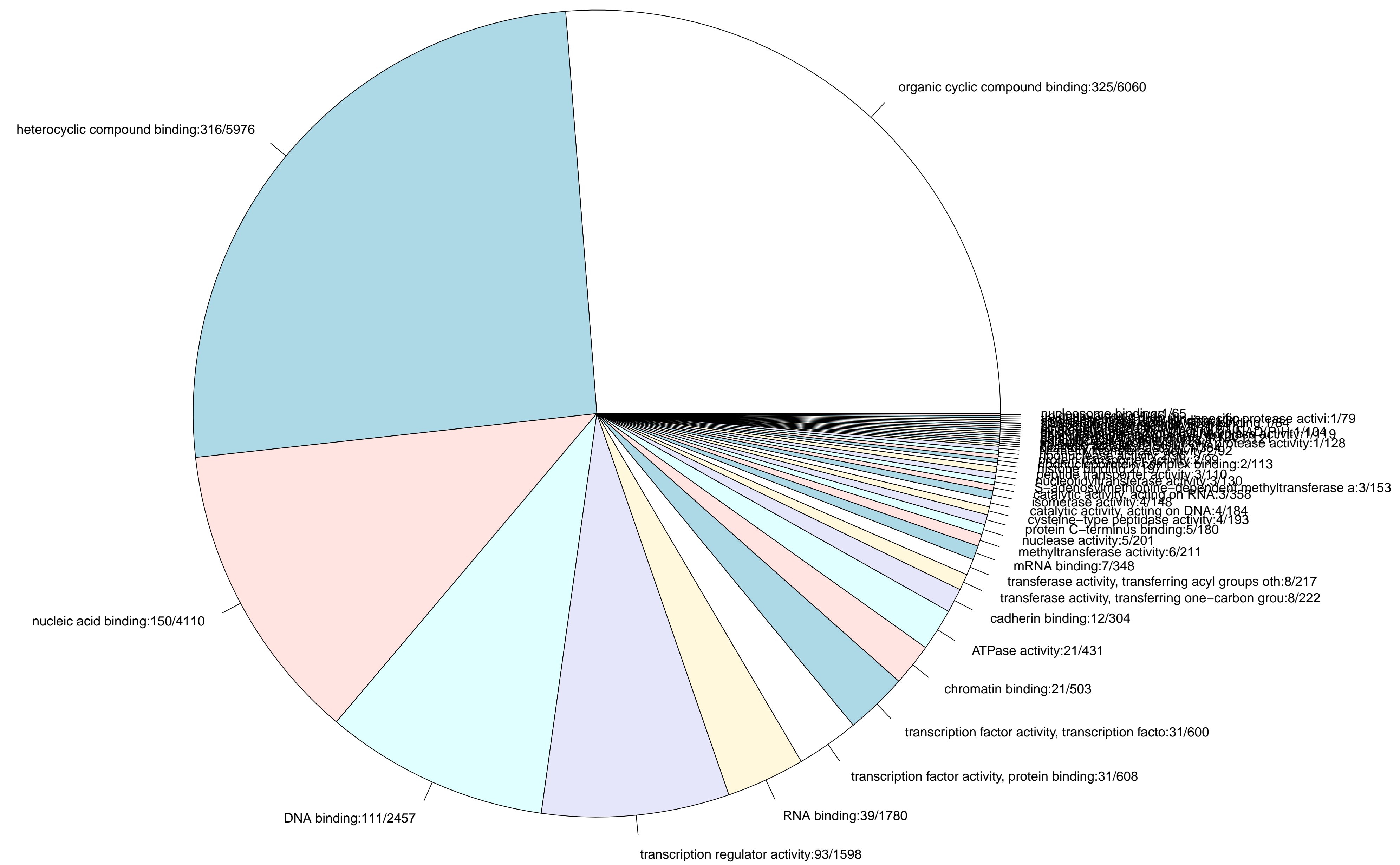

Supplement: DATASET S3 — GO-term analyses of GATA3-expressing and unscratched pHAs versus EGFP-expressing and unscratched pHAs in 2D cultures. [file Data_Sheet_3.ZIP › GO_term_analyses_GATA3u_vs_GFPu/GOstats/GOstats_MF_Down_pieChart.pdf]

GOstats\_MF\_Up\_pieChart

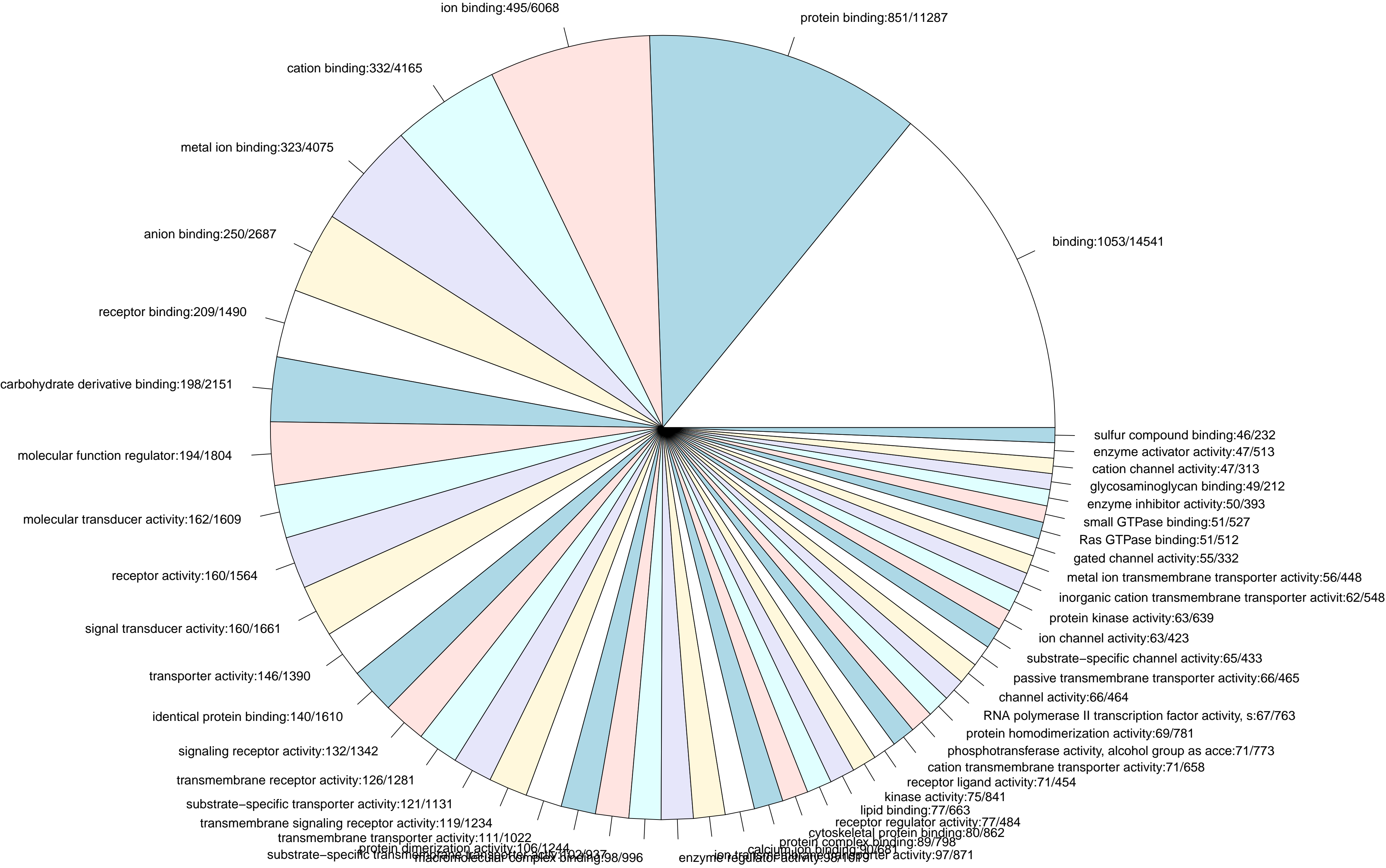

Supplement: DATASET S3 — GO-term analyses of GATA3-expressing and unscratched pHAs versus EGFP-expressing and unscratched pHAs in 2D cultures. [file Data_Sheet_3.ZIP › GO_term_analyses_GATA3u_vs_GFPu/GOstats/GOstats_MF_Up_pieChart.pdf]

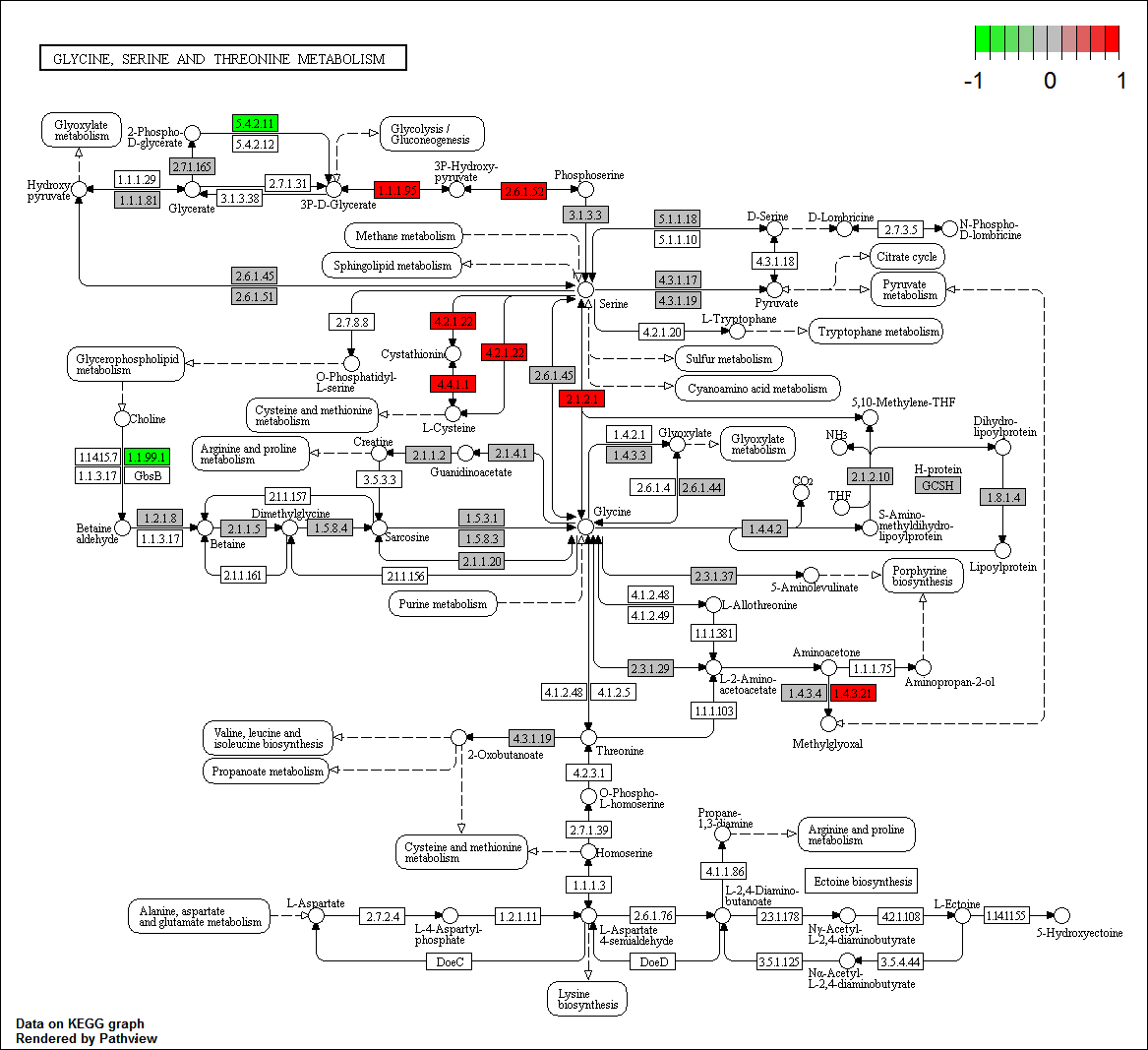

Supplement: DATASET S3 — GO-term analyses of GATA3-expressing and unscratched pHAs versus EGFP-expressing and unscratched pHAs in 2D cultures. [file Data_Sheet_3.ZIP › GO_term_analyses_GATA3u_vs_GFPu/GOstats/hsa00260.Glycine,serineandthreoninemetabolism.png]

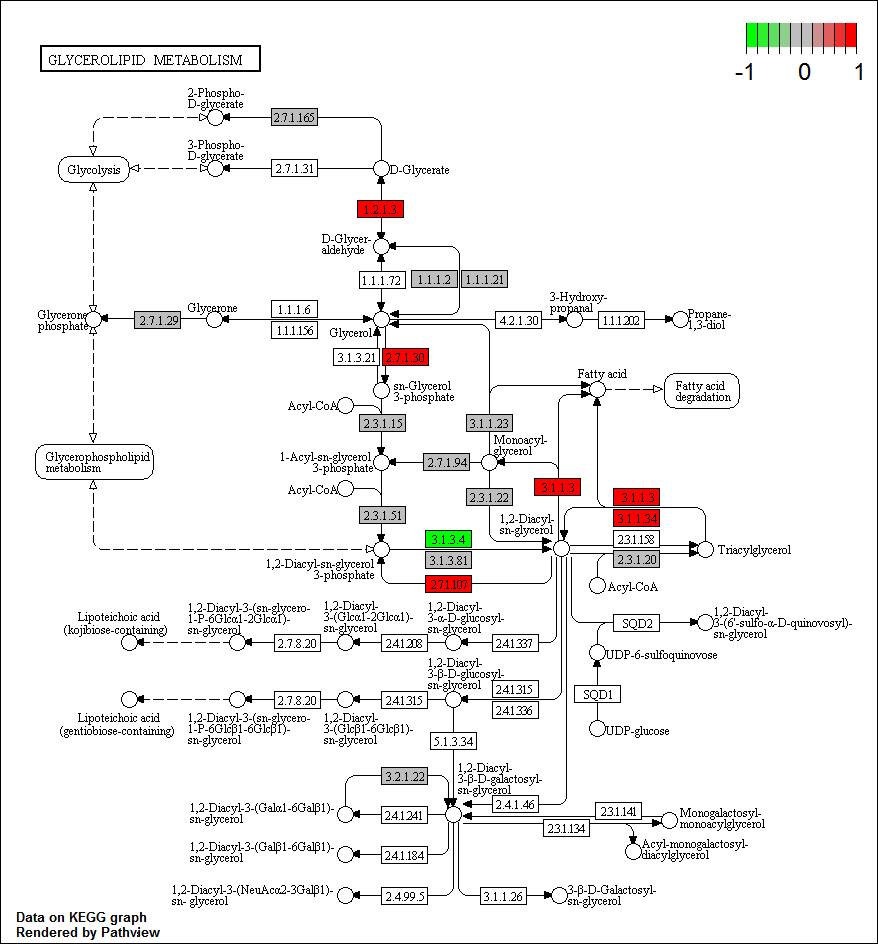

Supplement: DATASET S3 — GO-term analyses of GATA3-expressing and unscratched pHAs versus EGFP-expressing and unscratched pHAs in 2D cultures. [file Data_Sheet_3.ZIP › GO_term_analyses_GATA3u_vs_GFPu/GOstats/hsa00561.Glycerolipidmetabolism.png]

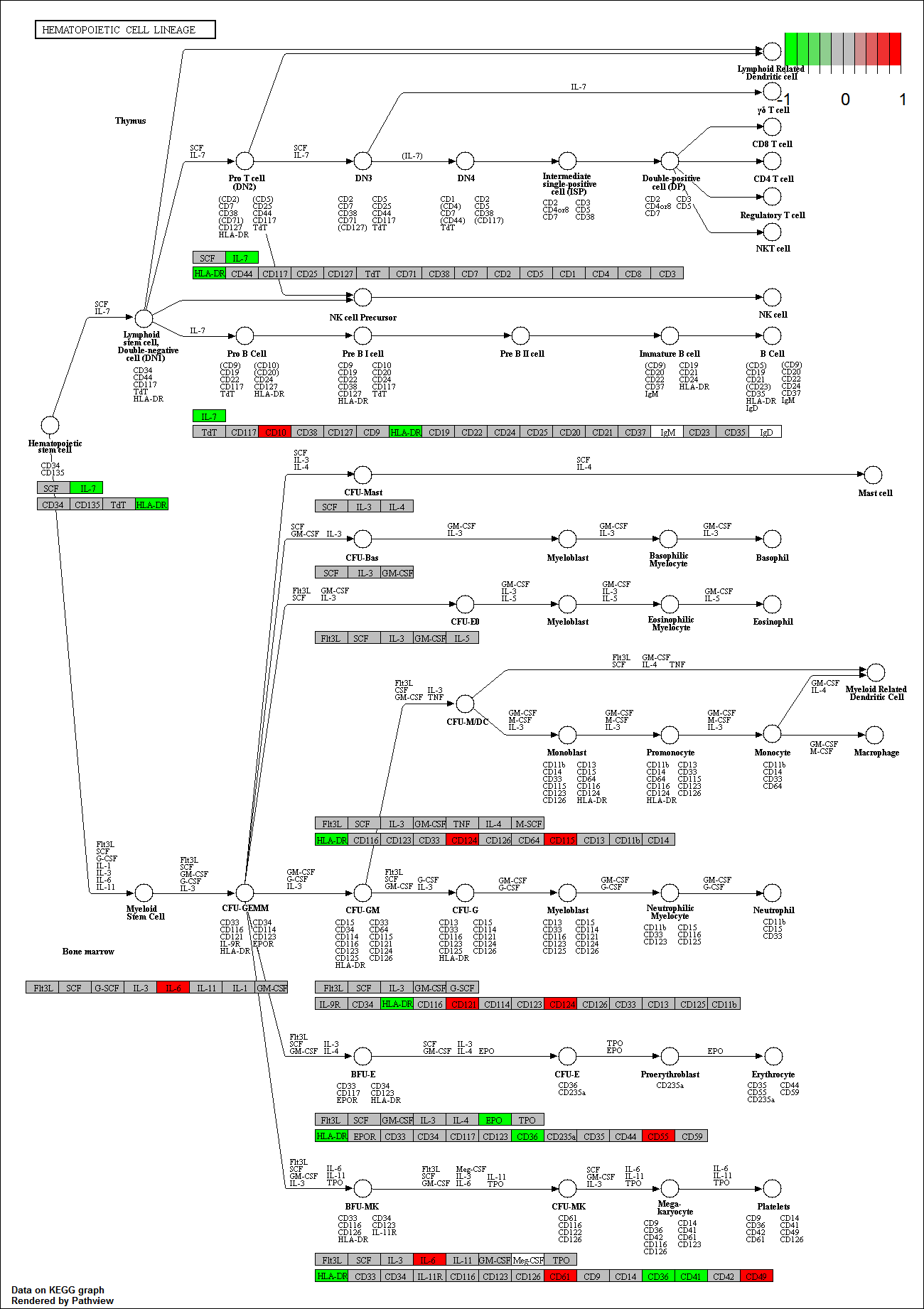

Supplement: DATASET S3 — GO-term analyses of GATA3-expressing and unscratched pHAs versus EGFP-expressing and unscratched pHAs in 2D cultures. [file Data_Sheet_3.ZIP › GO_term_analyses_GATA3u_vs_GFPu/GOstats/hsa04640.Hematopoieticcelllineage.png]

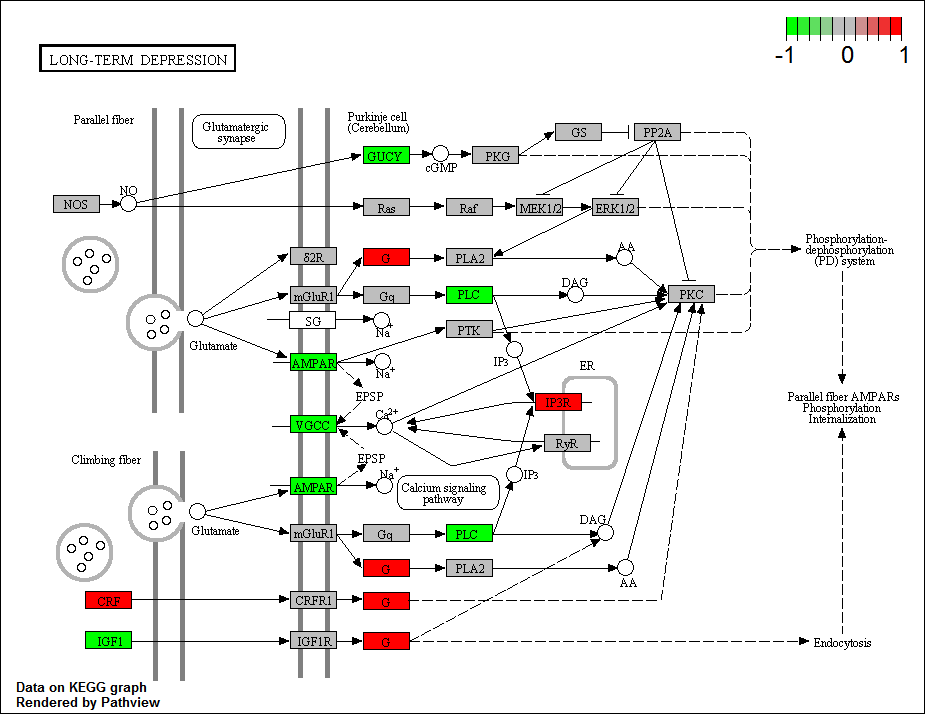

Supplement: DATASET S3 — GO-term analyses of GATA3-expressing and unscratched pHAs versus EGFP-expressing and unscratched pHAs in 2D cultures. [file Data_Sheet_3.ZIP › GO_term_analyses_GATA3u_vs_GFPu/GOstats/hsa04730.Long-termdepression.png]

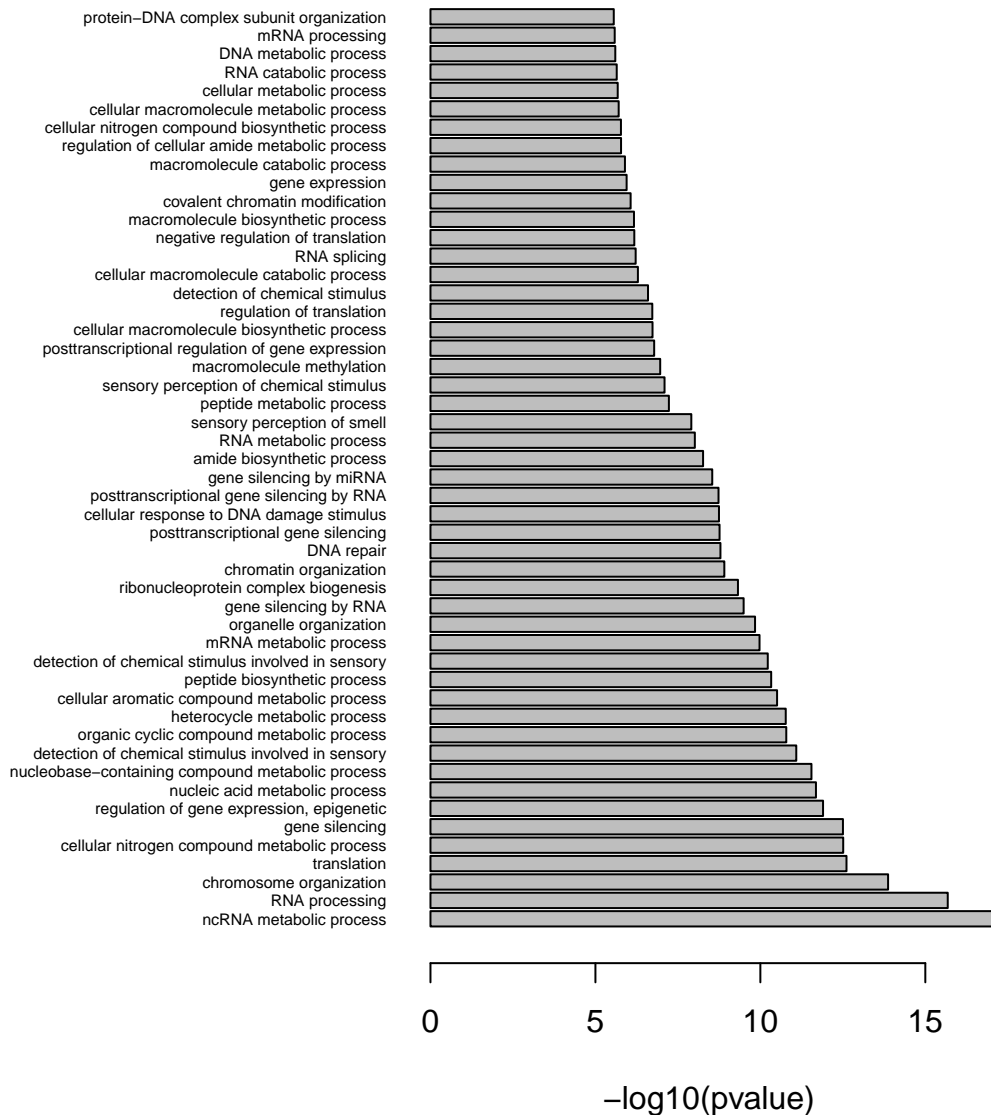

Supplement: DATASET S3 — GO-term analyses of GATA3-expressing and unscratched pHAs versus EGFP-expressing and unscratched pHAs in 2D cultures. [file Data_Sheet_3.ZIP › GO_term_analyses_GATA3u_vs_GFPu/GOstats/pVal_GOstats_BP_Down_pieChart.pdf]

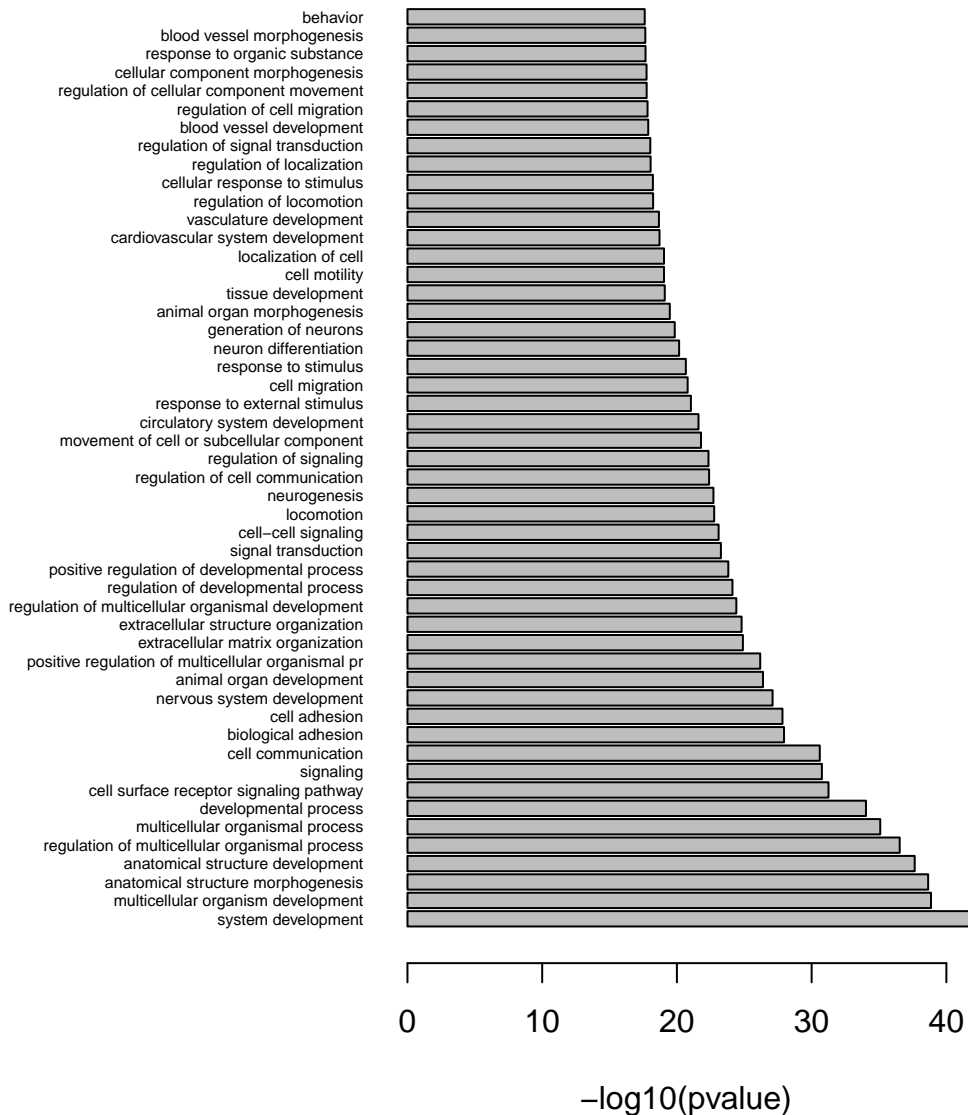

Supplement: DATASET S3 — GO-term analyses of GATA3-expressing and unscratched pHAs versus EGFP-expressing and unscratched pHAs in 2D cultures. [file Data_Sheet_3.ZIP › GO_term_analyses_GATA3u_vs_GFPu/GOstats/pVal_GOstats_BP_Up_pieChart.pdf]

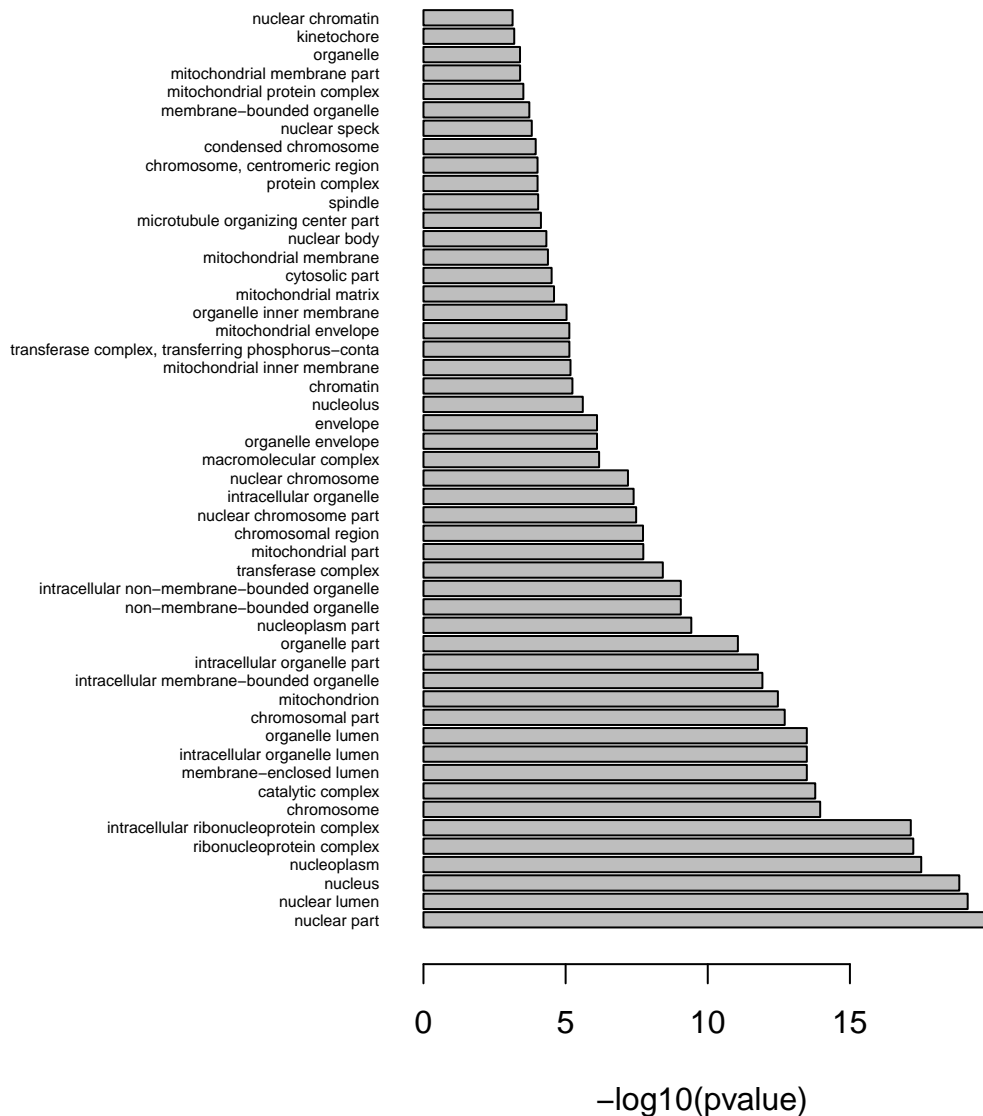

Supplement: DATASET S3 — GO-term analyses of GATA3-expressing and unscratched pHAs versus EGFP-expressing and unscratched pHAs in 2D cultures. [file Data_Sheet_3.ZIP › GO_term_analyses_GATA3u_vs_GFPu/GOstats/pVal_GOstats_CC_Down_pieChart.pdf]

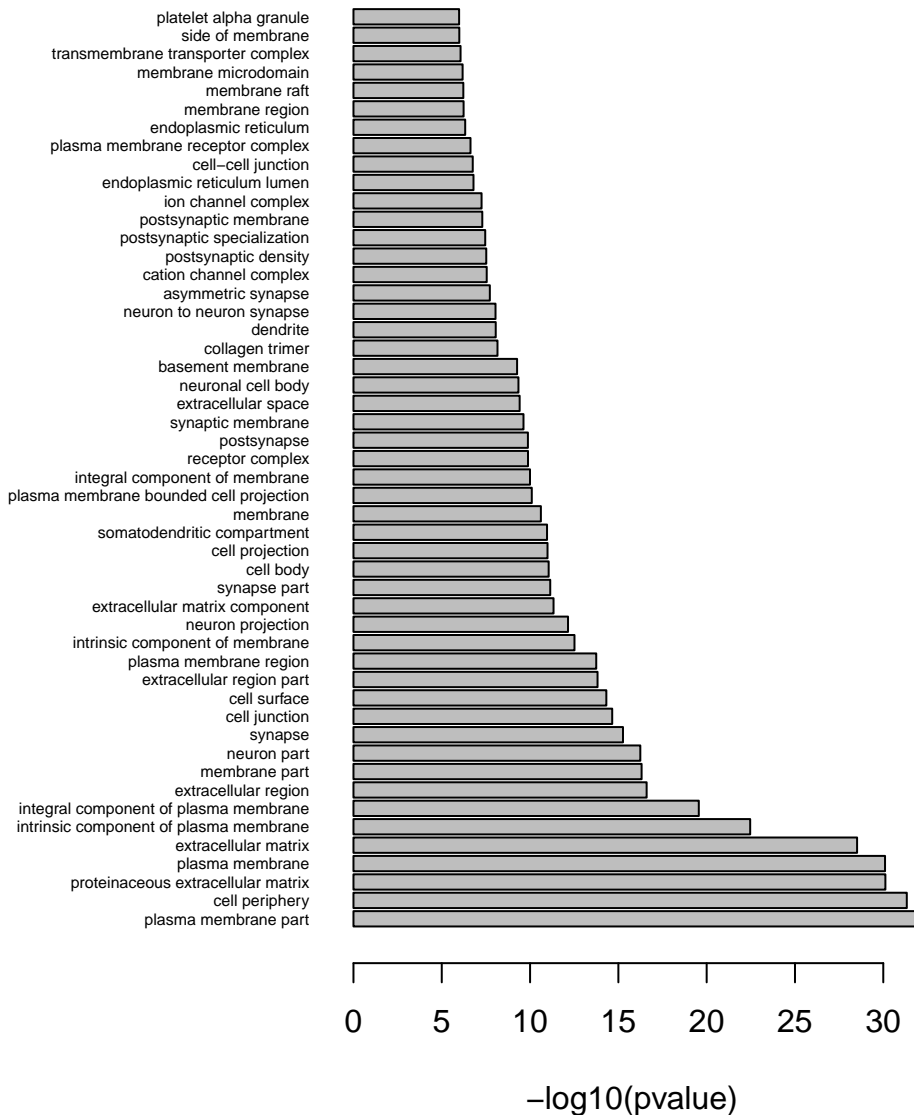

Supplement: DATASET S3 — GO-term analyses of GATA3-expressing and unscratched pHAs versus EGFP-expressing and unscratched pHAs in 2D cultures. [file Data_Sheet_3.ZIP › GO_term_analyses_GATA3u_vs_GFPu/GOstats/pVal_GOstats_CC_Up_pieChart.pdf]

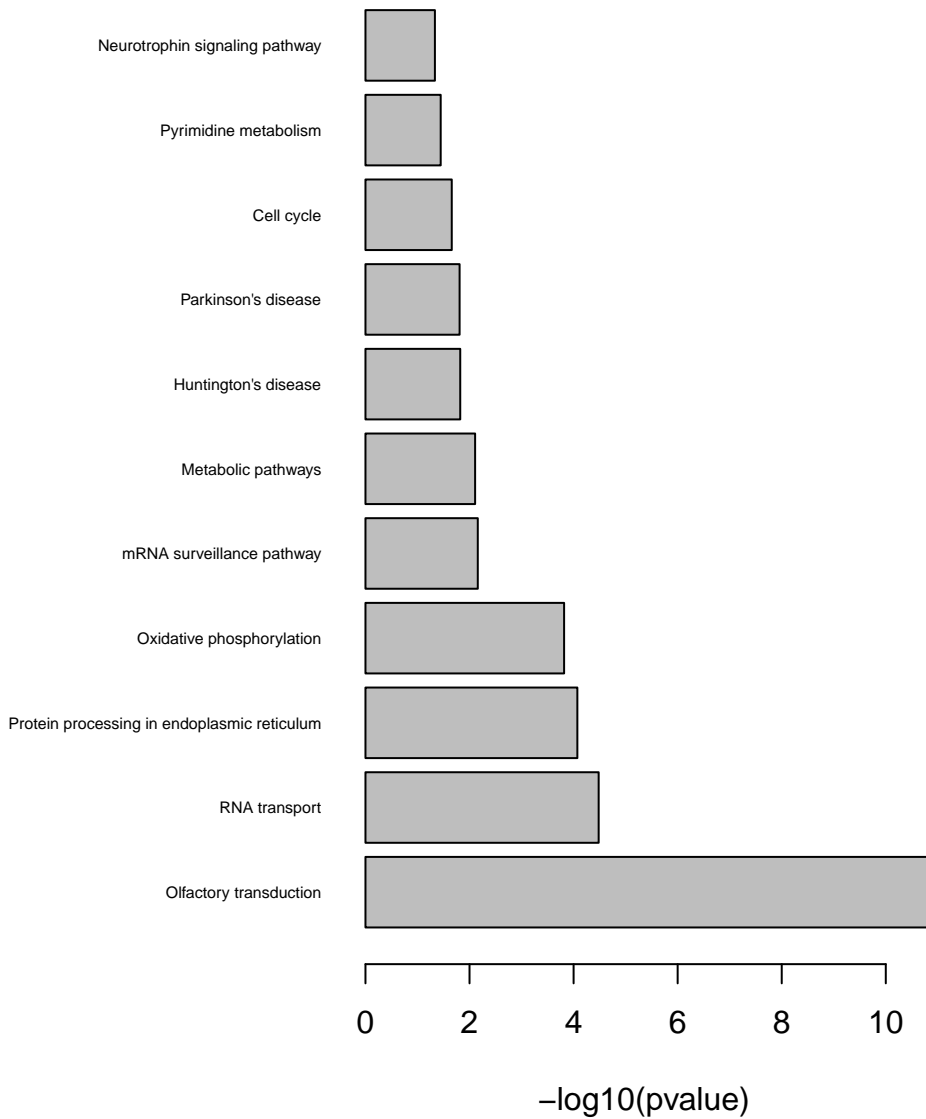

Supplement: DATASET S3 — GO-term analyses of GATA3-expressing and unscratched pHAs versus EGFP-expressing and unscratched pHAs in 2D cultures. [file Data_Sheet_3.ZIP › GO_term_analyses_GATA3u_vs_GFPu/GOstats/pVal_GOstats_kegg_Under.pdf]

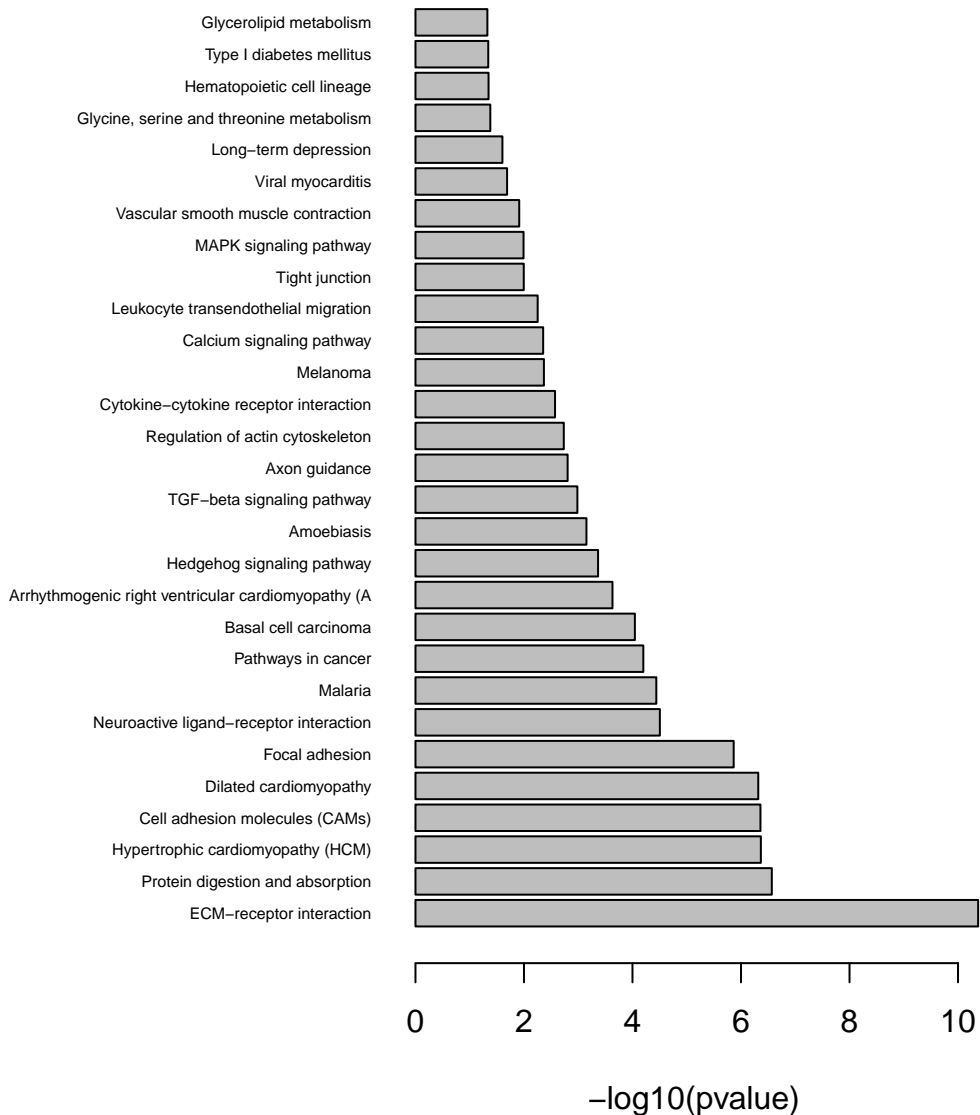

Supplement: DATASET S3 — GO-term analyses of GATA3-expressing and unscratched pHAs versus EGFP-expressing and unscratched pHAs in 2D cultures. [file Data_Sheet_3.ZIP › GO_term_analyses_GATA3u_vs_GFPu/GOstats/pVal_GOstats_kegg_Up.pdf]

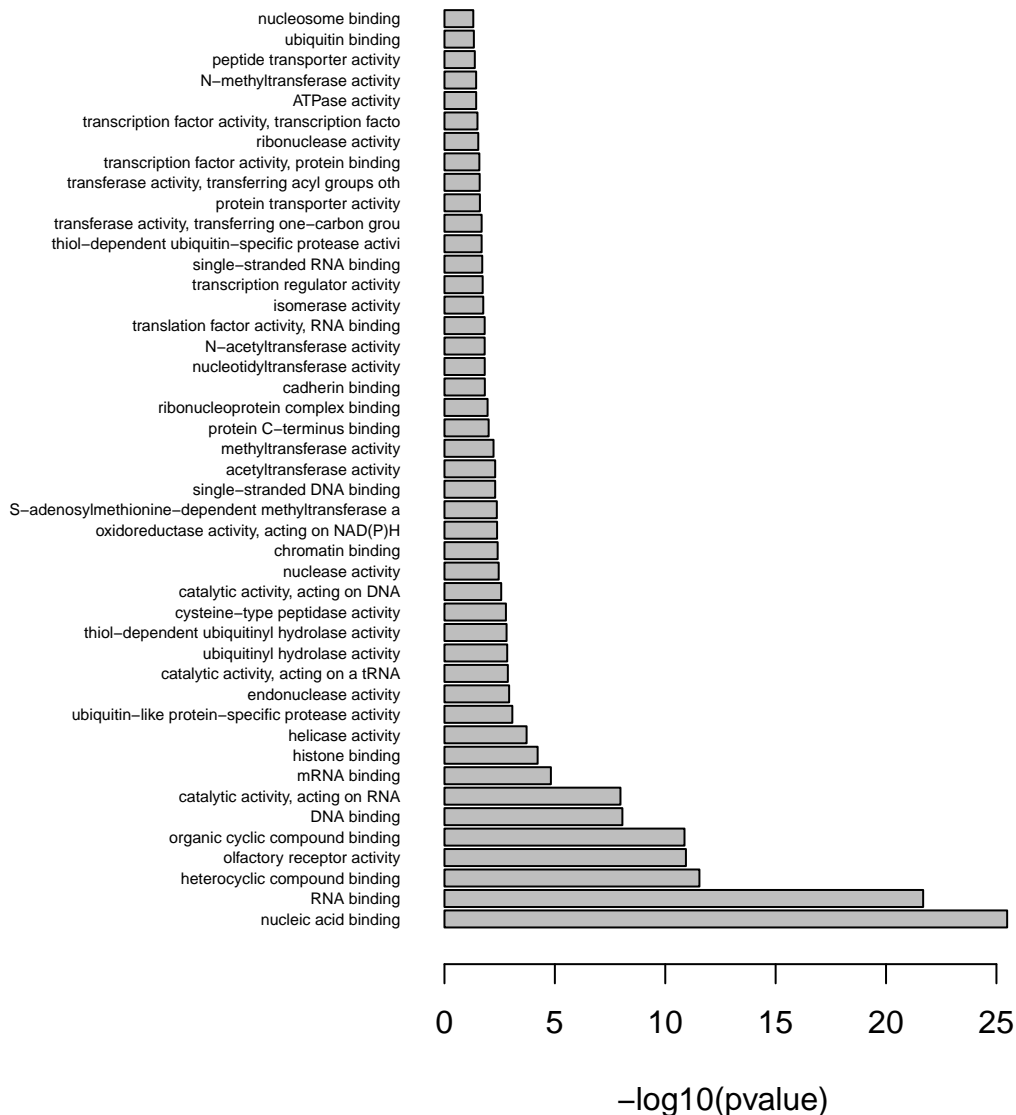

Supplement: DATASET S3 — GO-term analyses of GATA3-expressing and unscratched pHAs versus EGFP-expressing and unscratched pHAs in 2D cultures. [file Data_Sheet_3.ZIP › GO_term_analyses_GATA3u_vs_GFPu/GOstats/pVal_GOstats_MF_Down_pieChart.pdf]

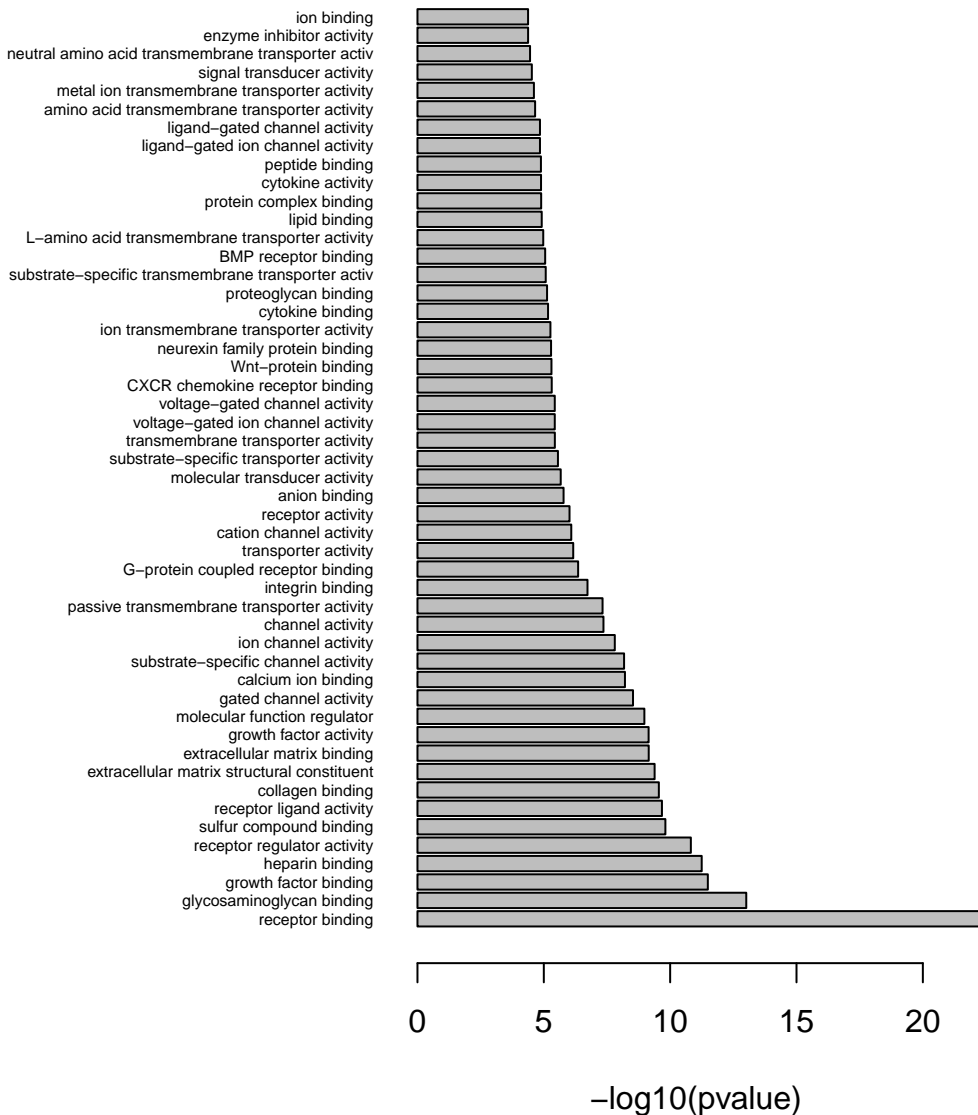

Supplement: DATASET S3 — GO-term analyses of GATA3-expressing and unscratched pHAs versus EGFP-expressing and unscratched pHAs in 2D cultures. [file Data_Sheet_3.ZIP › GO_term_analyses_GATA3u_vs_GFPu/GOstats/pVal_GOstats_MF_Up_pieChart.pdf]

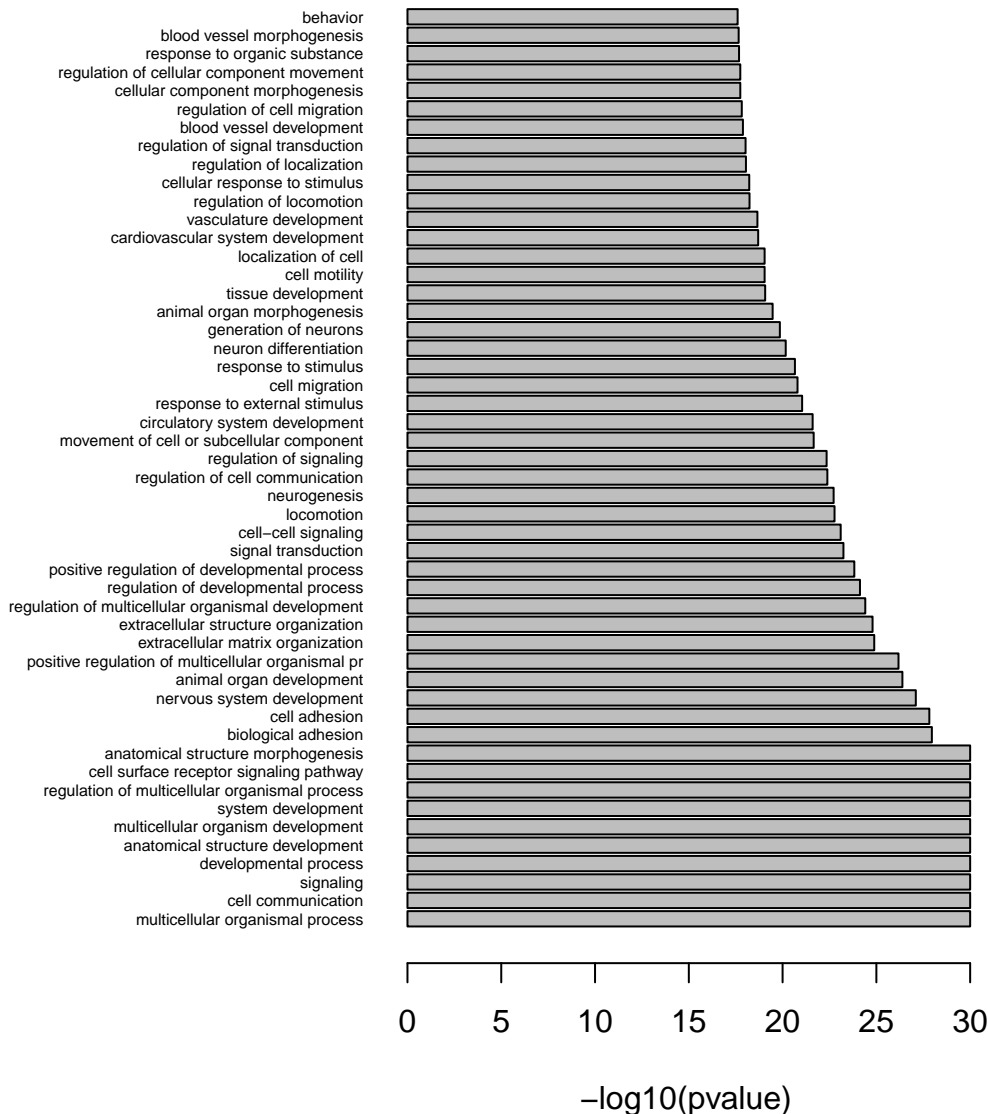

Supplement: DATASET S3 — GO-term analyses of GATA3-expressing and unscratched pHAs versus EGFP-expressing and unscratched pHAs in 2D cultures. [file Data_Sheet_3.ZIP › GO_term_analyses_GATA3u_vs_GFPu/topGO/pVal_topGO_BP_classicfisher_pieChart.pdf]

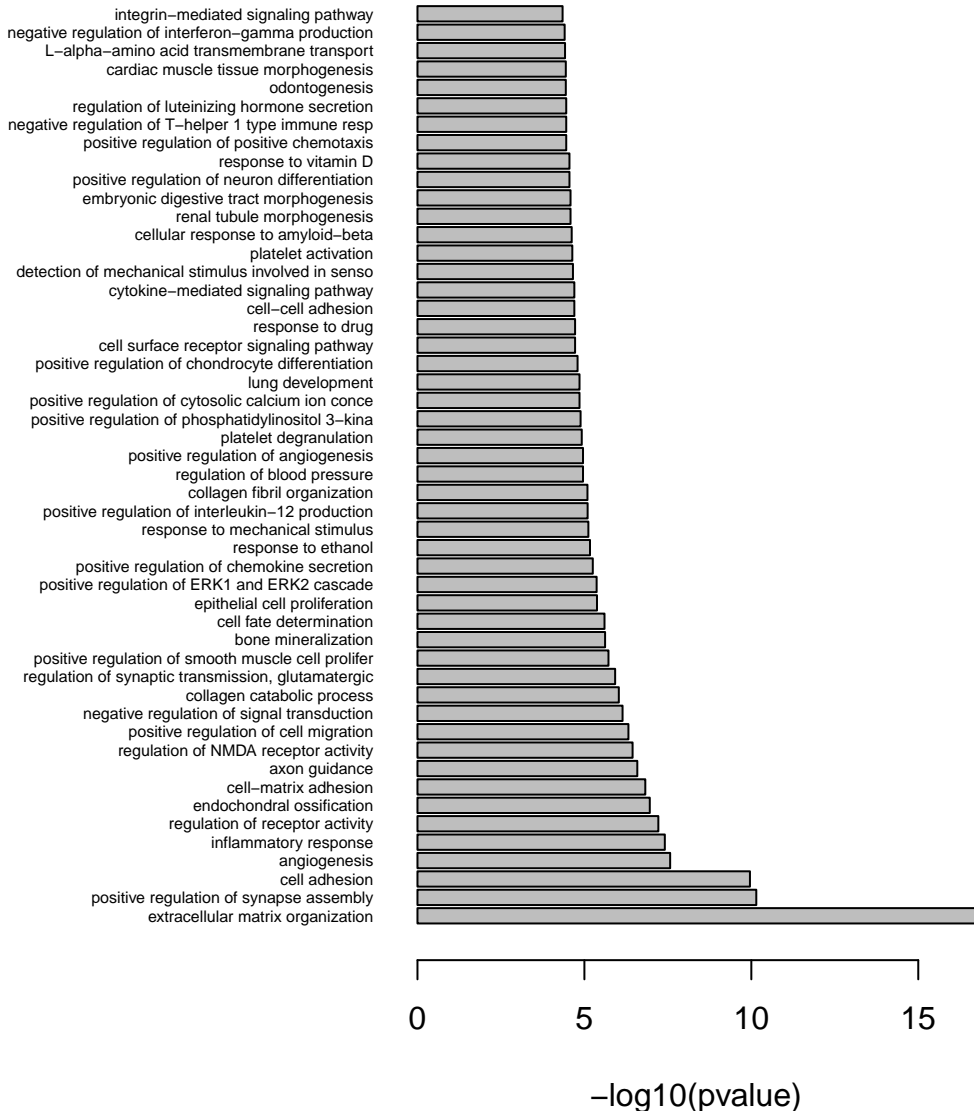

Supplement: DATASET S3 — GO-term analyses of GATA3-expressing and unscratched pHAs versus EGFP-expressing and unscratched pHAs in 2D cultures. [file Data_Sheet_3.ZIP › GO_term_analyses_GATA3u_vs_GFPu/topGO/pVal_topGO_BP_elimfisher.pdf]

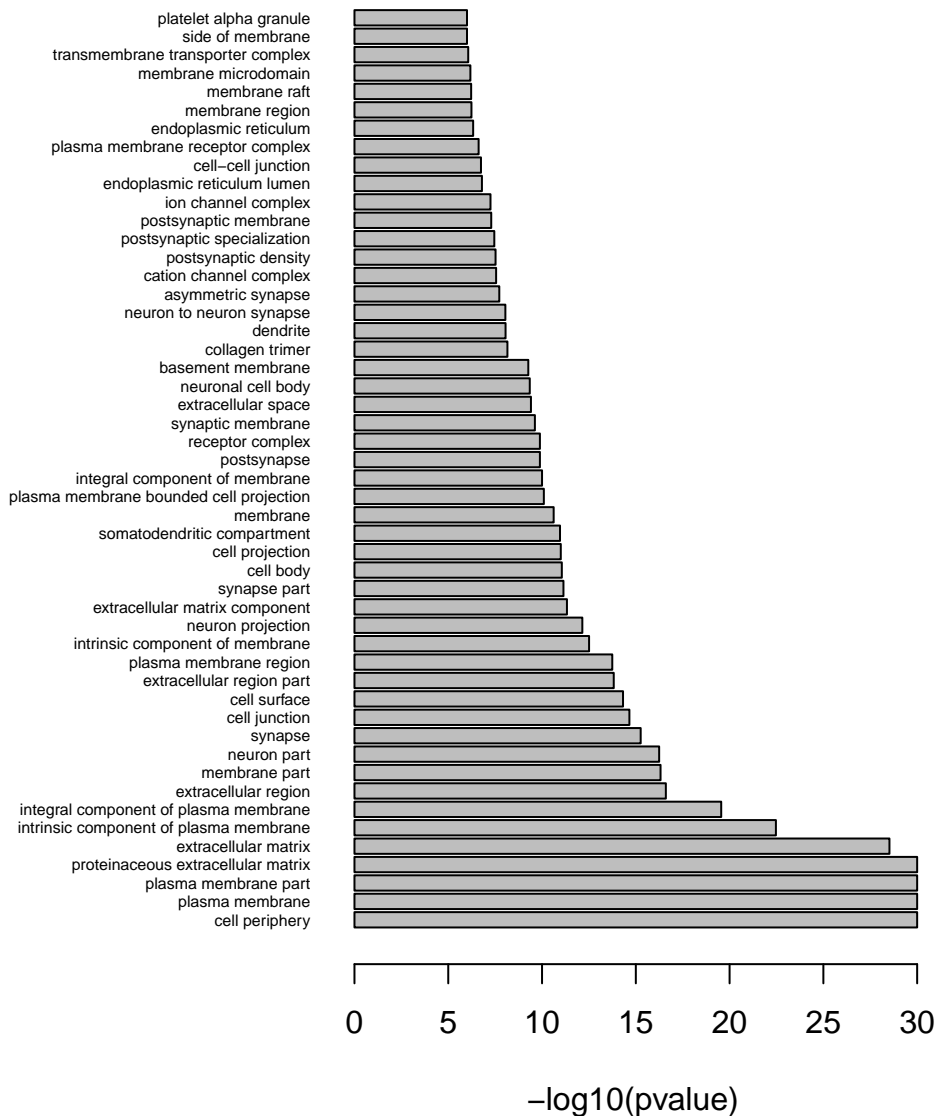

Supplement: DATASET S3 — GO-term analyses of GATA3-expressing and unscratched pHAs versus EGFP-expressing and unscratched pHAs in 2D cultures. [file Data_Sheet_3.ZIP › GO_term_analyses_GATA3u_vs_GFPu/topGO/pVal_topGO_CC_classicfisher_pieChart.pdf]

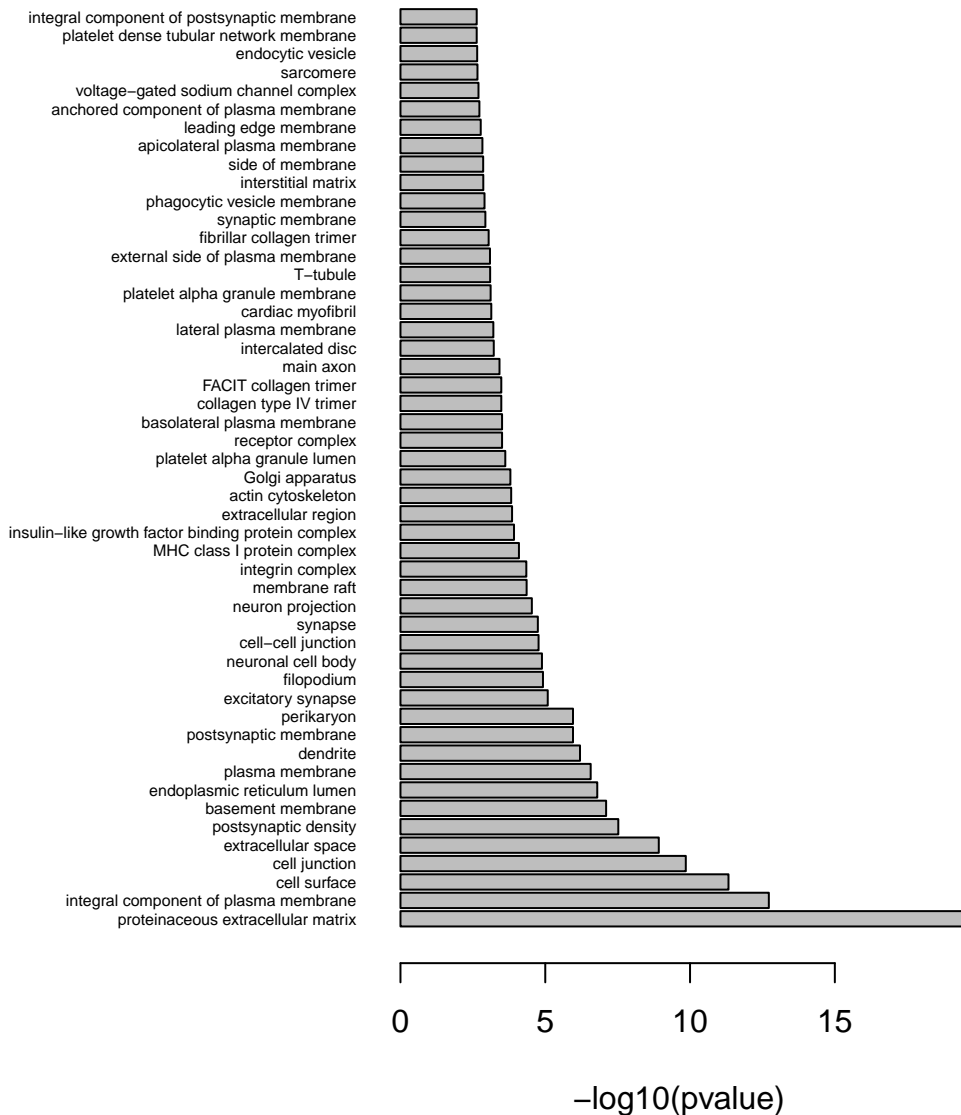

Supplement: DATASET S3 — GO-term analyses of GATA3-expressing and unscratched pHAs versus EGFP-expressing and unscratched pHAs in 2D cultures. [file Data_Sheet_3.ZIP › GO_term_analyses_GATA3u_vs_GFPu/topGO/pVal_topGO_CC_elimfisher_pieChart.pdf]

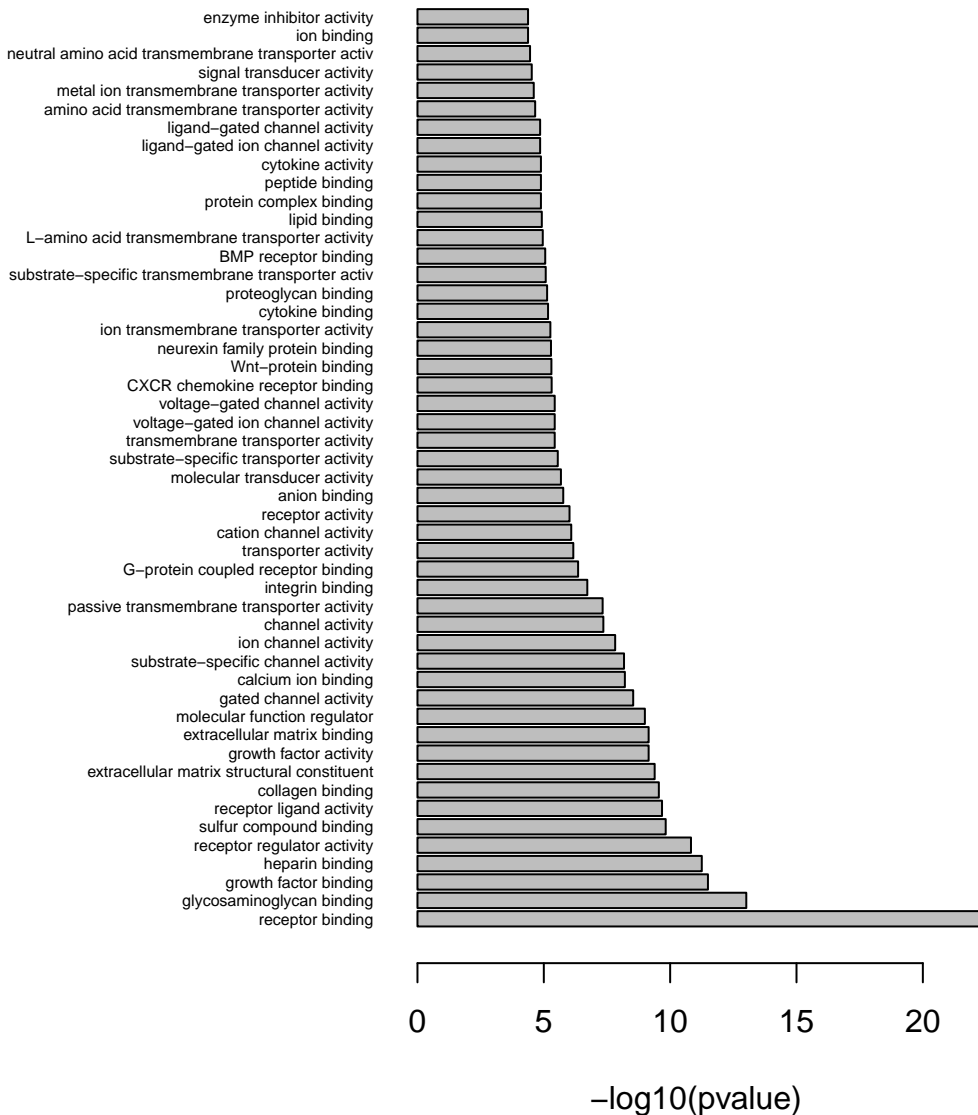

Supplement: DATASET S3 — GO-term analyses of GATA3-expressing and unscratched pHAs versus EGFP-expressing and unscratched pHAs in 2D cultures. [file Data_Sheet_3.ZIP › GO_term_analyses_GATA3u_vs_GFPu/topGO/pVal_topGO_MF_classicfisher_pieChart.pdf]

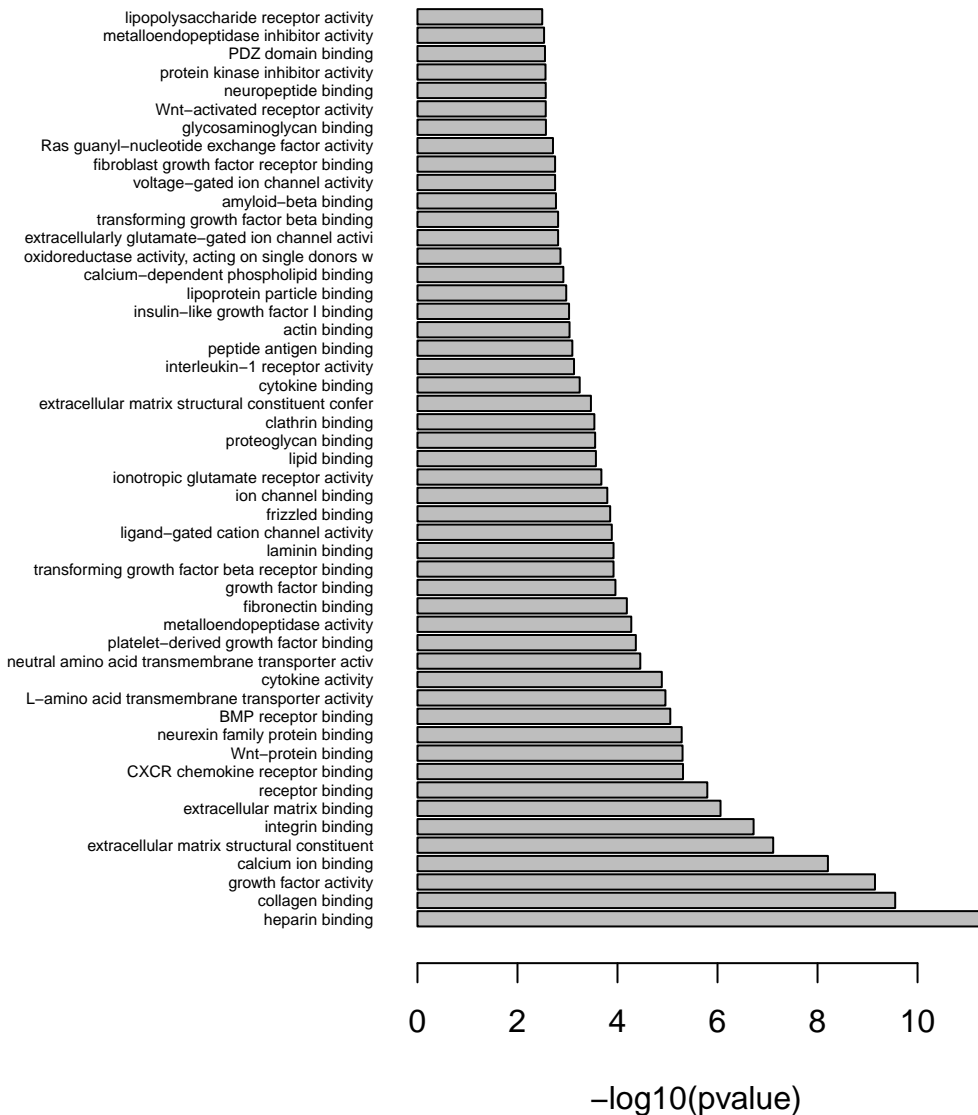

Supplement: DATASET S3 — GO-term analyses of GATA3-expressing and unscratched pHAs versus EGFP-expressing and unscratched pHAs in 2D cultures. [file Data_Sheet_3.ZIP › GO_term_analyses_GATA3u_vs_GFPu/topGO/pVal_topGO_MF_elimfisher_pieChart.pdf]

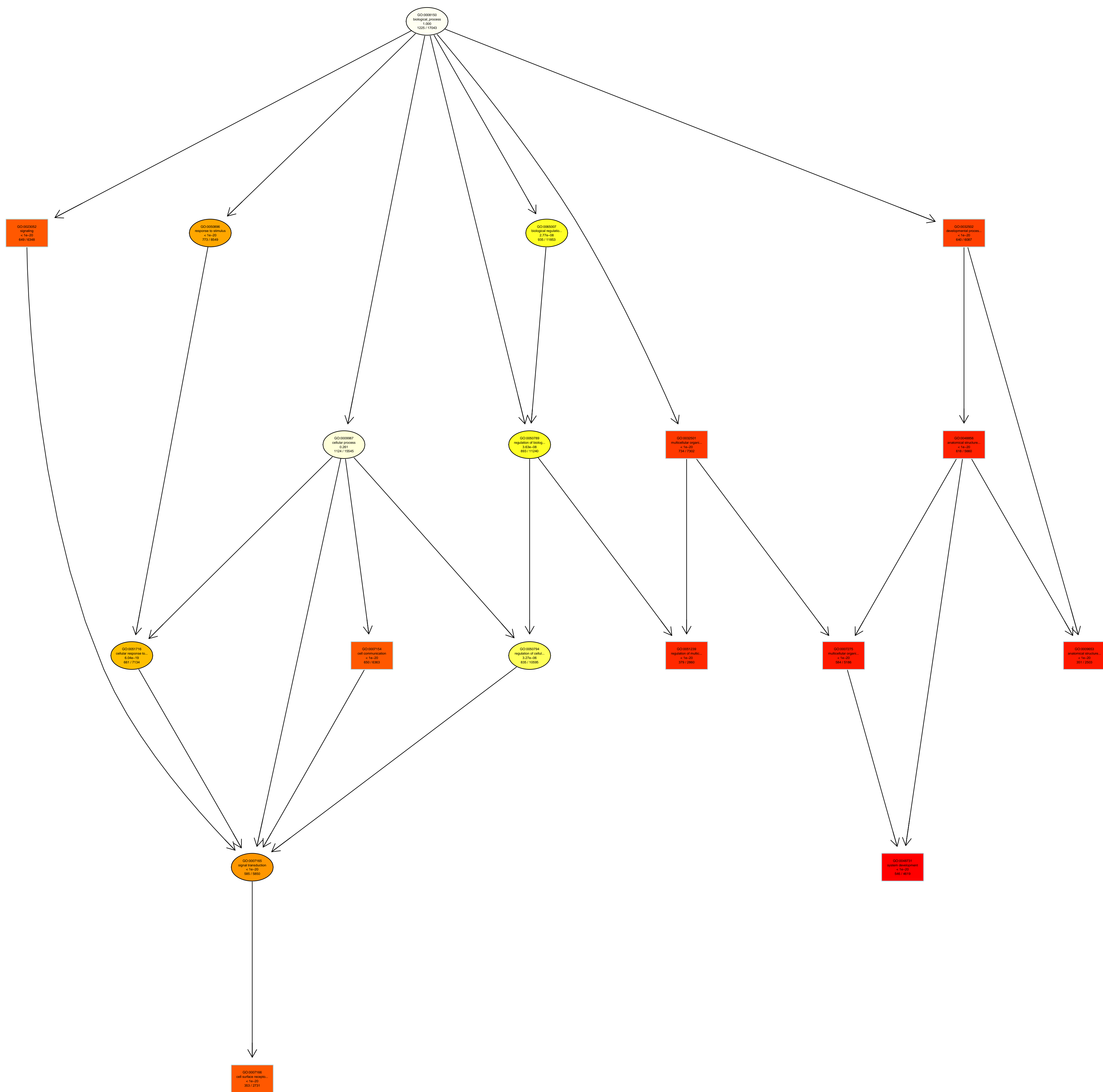

Supplement: DATASET S3 — GO-term analyses of GATA3-expressing and unscratched pHAs versus EGFP-expressing and unscratched pHAs in 2D cultures. [file Data_Sheet_3.ZIP › GO_term_analyses_GATA3u_vs_GFPu/topGO/topGO_BP_classicfisher_nodes.pdf]

topGO\_BP\_classicfisher\_pieChart

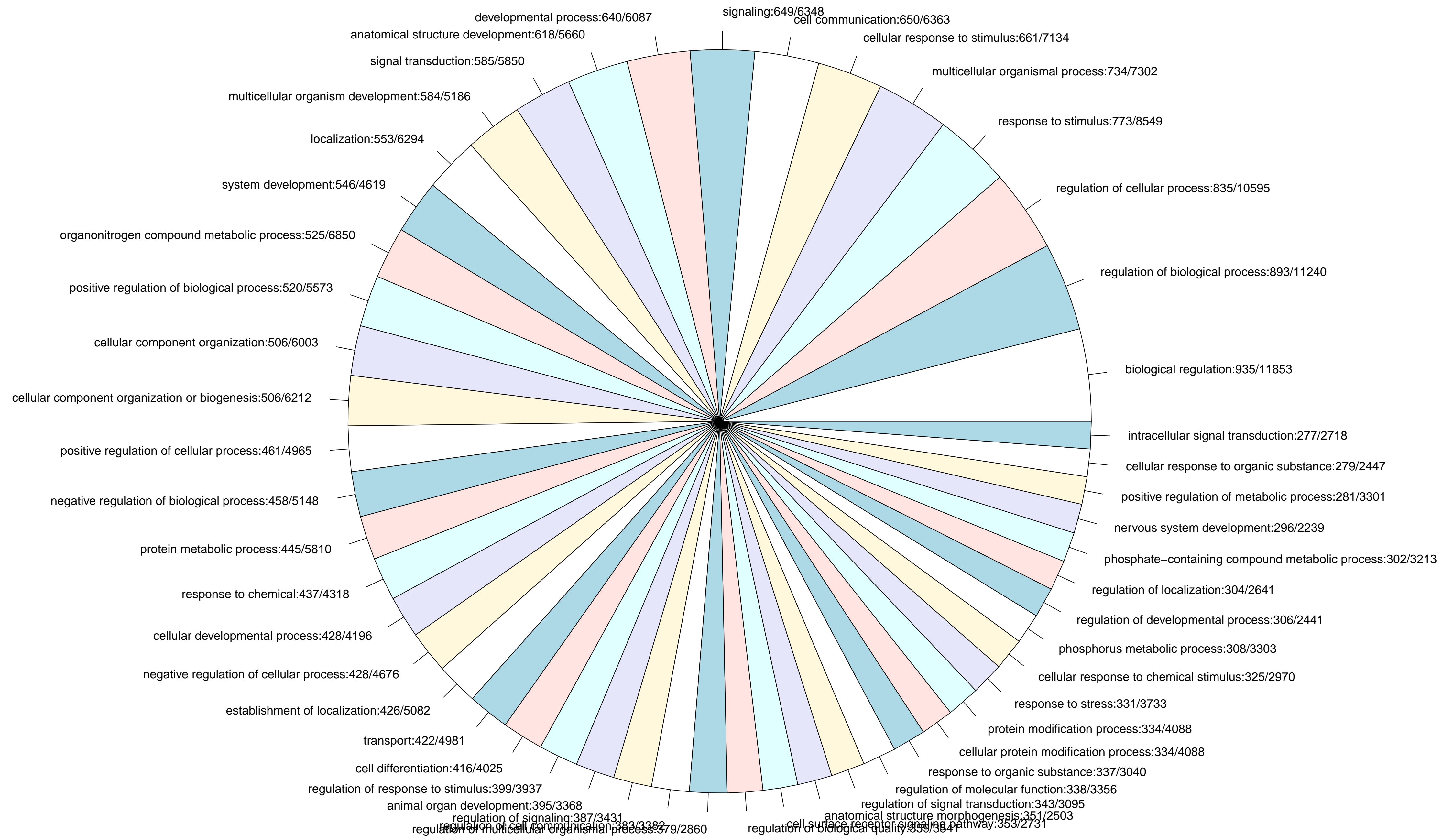

Supplement: DATASET S3 — GO-term analyses of GATA3-expressing and unscratched pHAs versus EGFP-expressing and unscratched pHAs in 2D cultures. [file Data_Sheet_3.ZIP › GO_term_analyses_GATA3u_vs_GFPu/topGO/topGO_BP_classicfisher_pieChart.pdf]

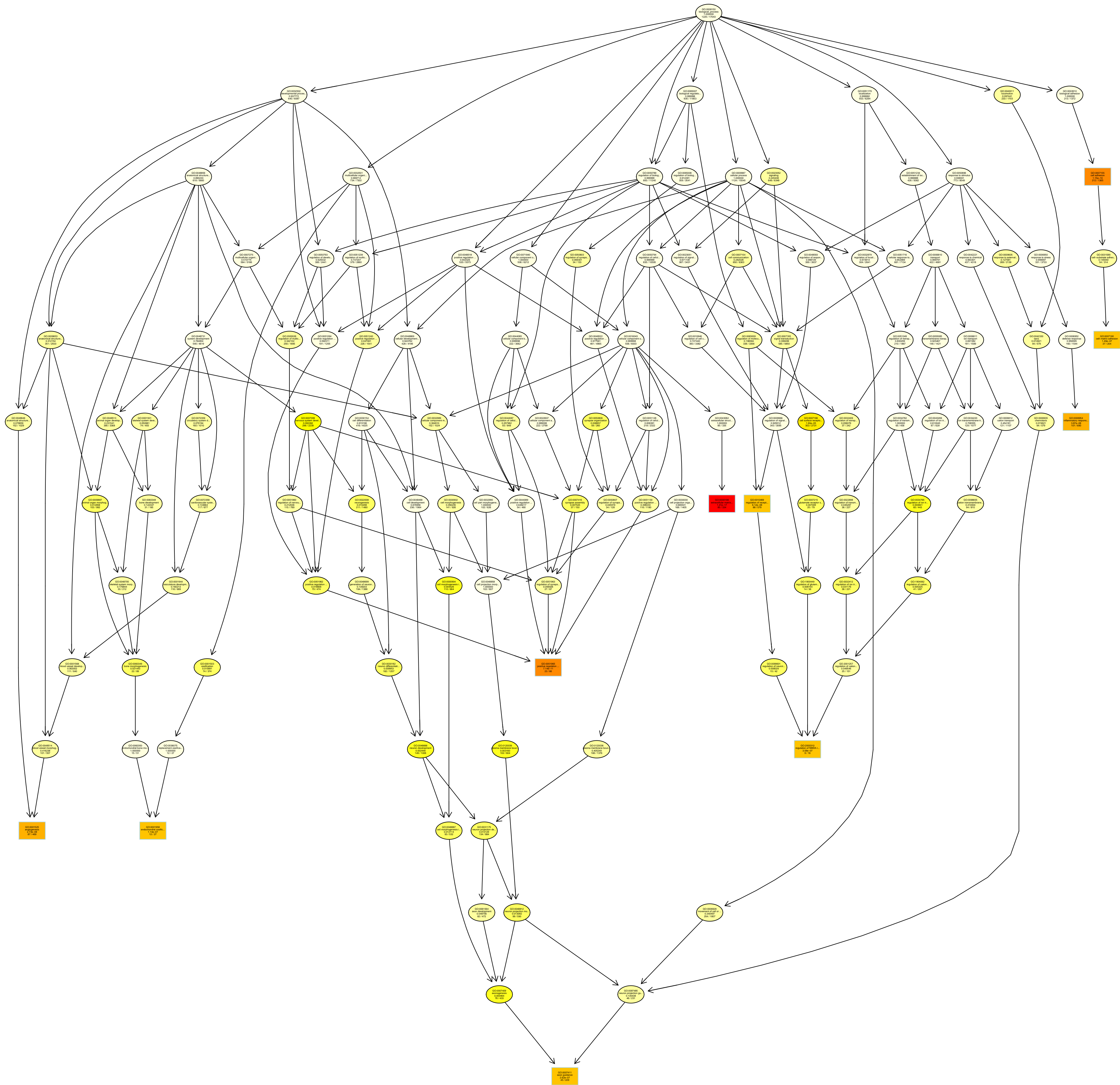

Supplement: DATASET S3 — GO-term analyses of GATA3-expressing and unscratched pHAs versus EGFP-expressing and unscratched pHAs in 2D cultures. [file Data_Sheet_3.ZIP › GO_term_analyses_GATA3u_vs_GFPu/topGO/topGO_BP_elimfisher_nodes.pdf]

topGO\_BP\_elimfisher\_pieChart

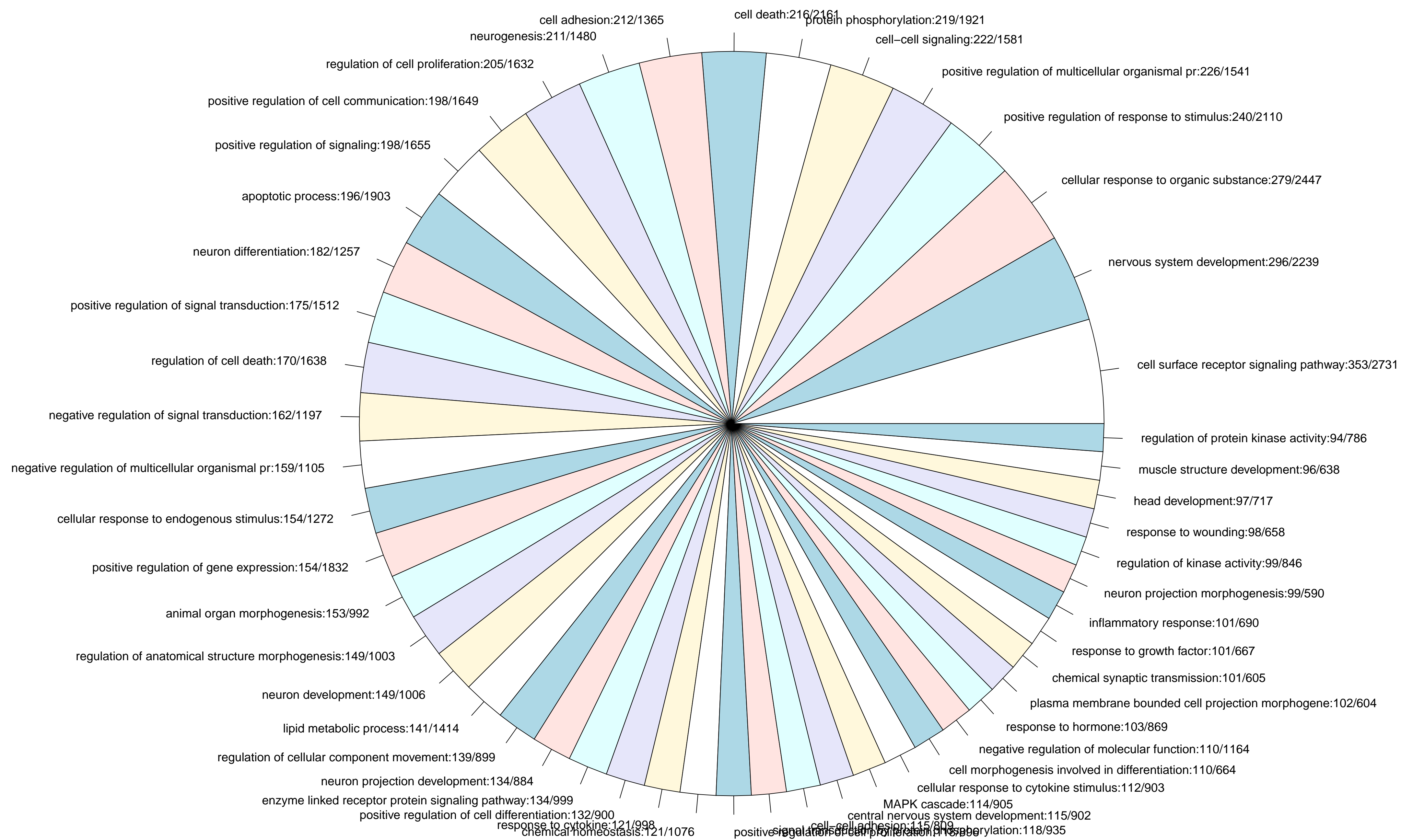

Supplement: DATASET S3 — GO-term analyses of GATA3-expressing and unscratched pHAs versus EGFP-expressing and unscratched pHAs in 2D cultures. [file Data_Sheet_3.ZIP › GO_term_analyses_GATA3u_vs_GFPu/topGO/topGO_BP_elimfisher_pieChart.pdf]

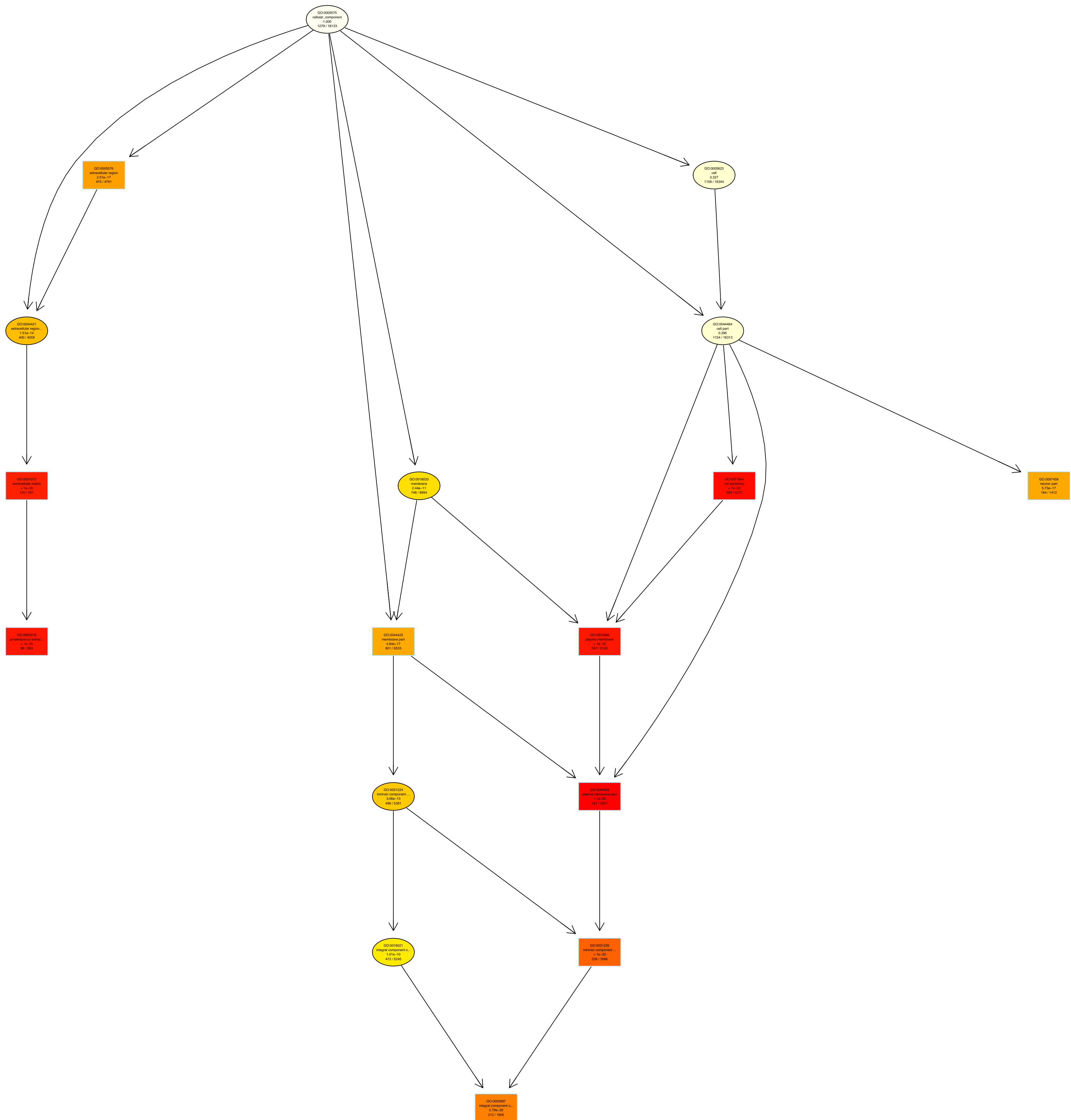

Supplement: DATASET S3 — GO-term analyses of GATA3-expressing and unscratched pHAs versus EGFP-expressing and unscratched pHAs in 2D cultures. [file Data_Sheet_3.ZIP › GO_term_analyses_GATA3u_vs_GFPu/topGO/topGO_CC_classicfisher_nodes.pdf]

topGO\_CC\_classicfisher\_pieChart

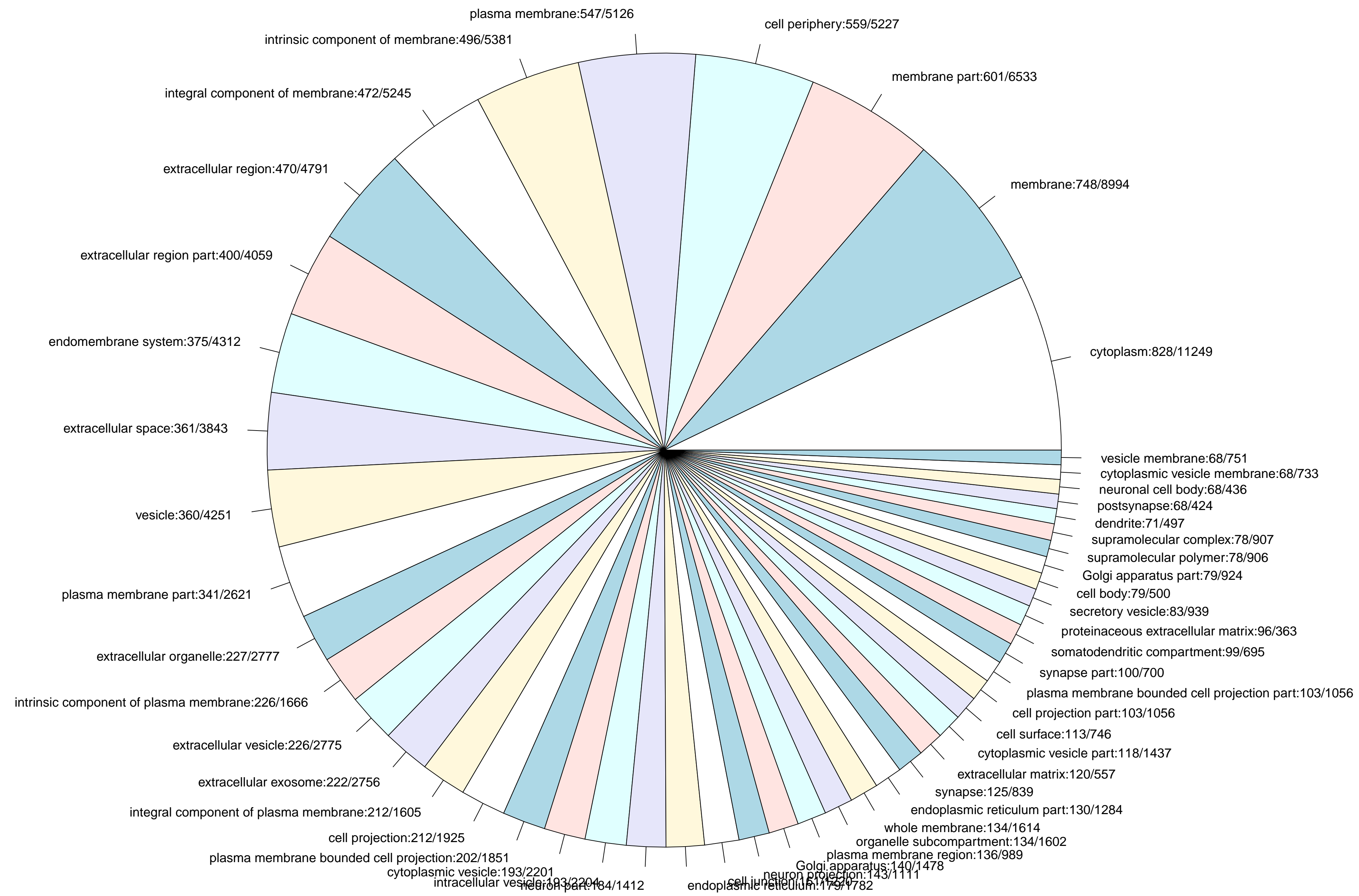

Supplement: DATASET S3 — GO-term analyses of GATA3-expressing and unscratched pHAs versus EGFP-expressing and unscratched pHAs in 2D cultures. [file Data_Sheet_3.ZIP › GO_term_analyses_GATA3u_vs_GFPu/topGO/topGO_CC_classicfisher_pieChart.pdf]

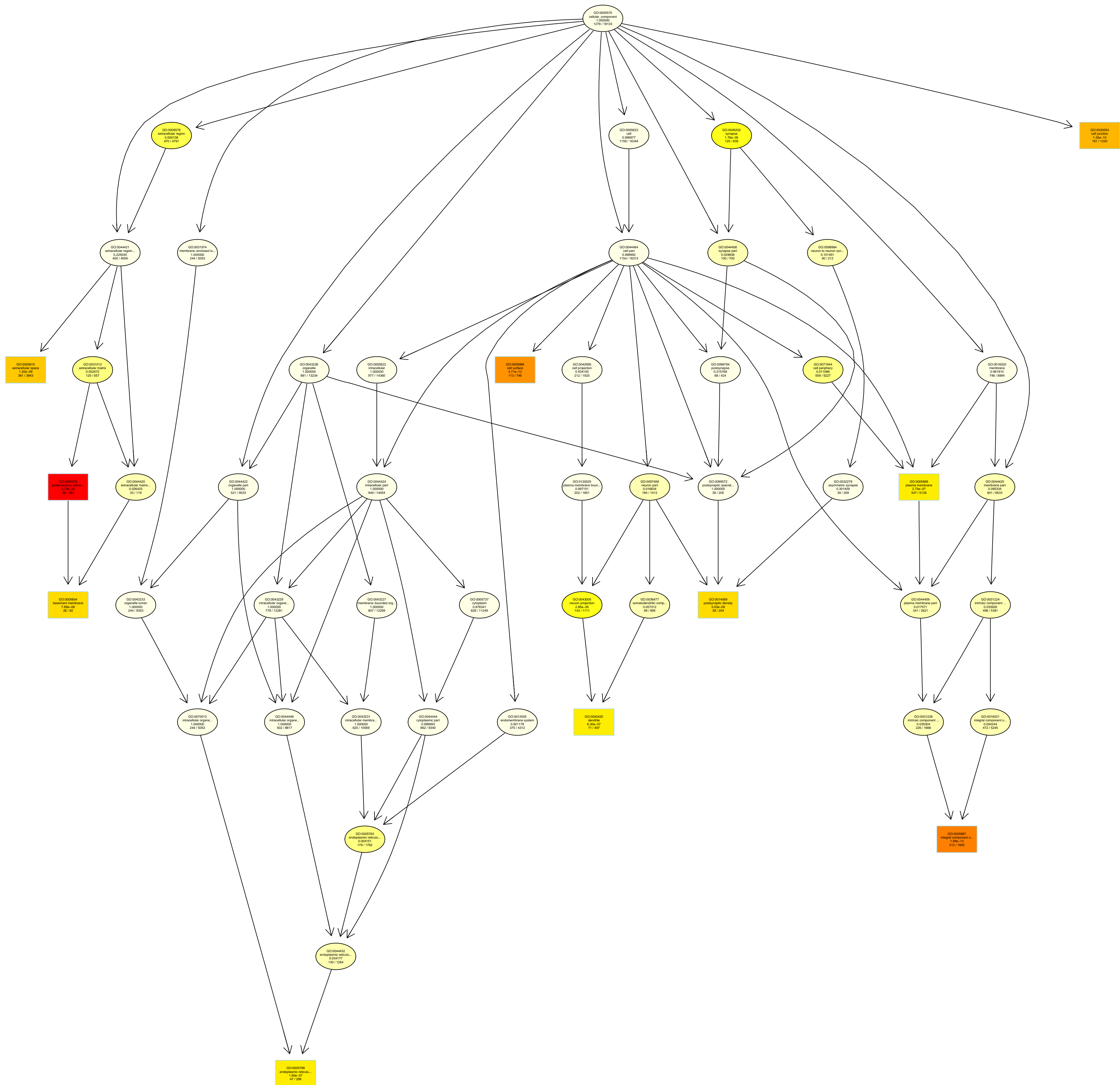

Supplement: DATASET S3 — GO-term analyses of GATA3-expressing and unscratched pHAs versus EGFP-expressing and unscratched pHAs in 2D cultures. [file Data_Sheet_3.ZIP › GO_term_analyses_GATA3u_vs_GFPu/topGO/topGO_CC_elimfisher_nodes.pdf]

topGO\_CC\_elimfisher\_pieChart

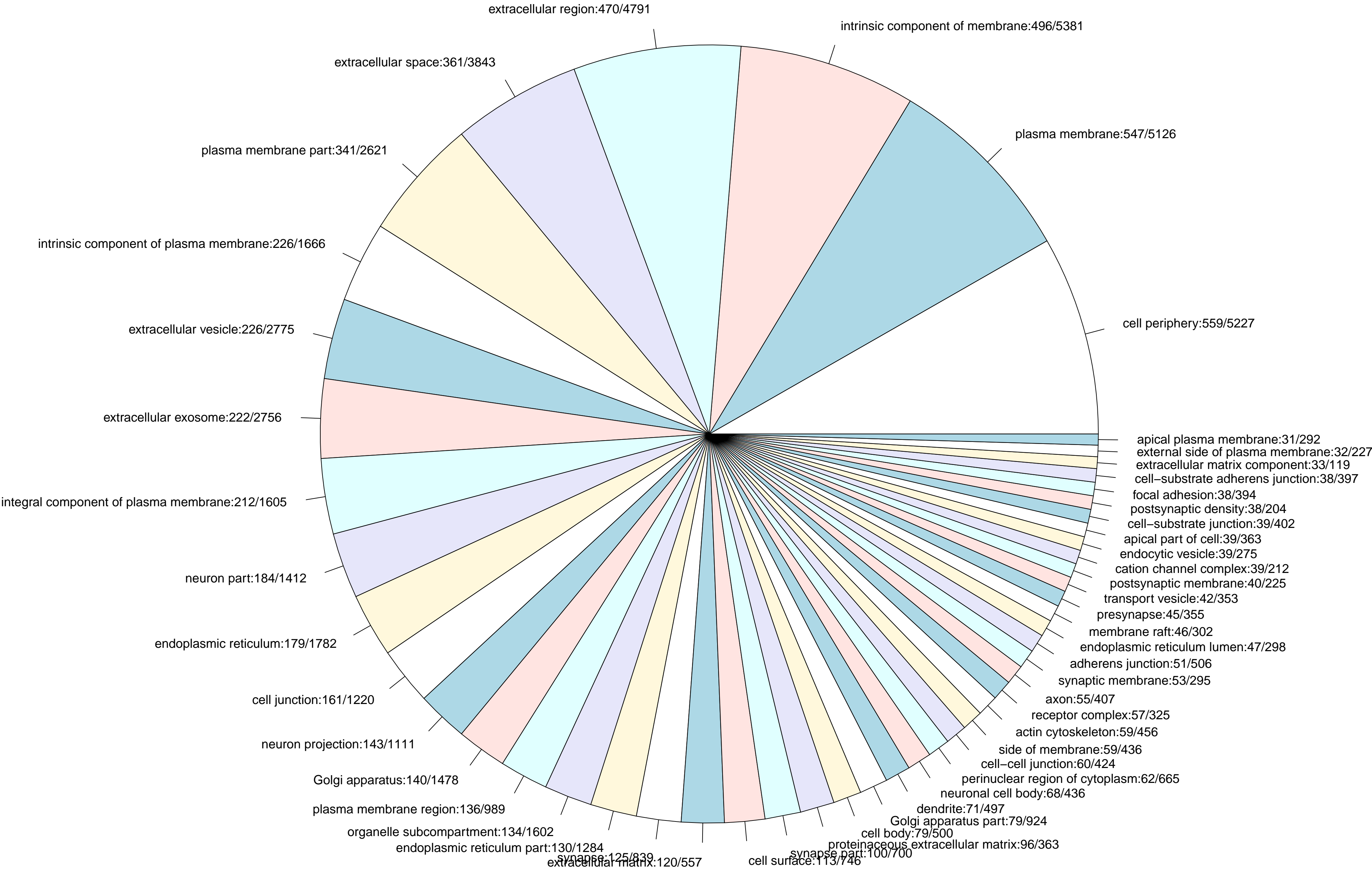

Supplement: DATASET S3 — GO-term analyses of GATA3-expressing and unscratched pHAs versus EGFP-expressing and unscratched pHAs in 2D cultures. [file Data_Sheet_3.ZIP › GO_term_analyses_GATA3u_vs_GFPu/topGO/topGO_CC_elimfisher_pieChart.pdf]
