# Supplementary figures and images for: GATA3 Promotes the Neural Progenitor State but Not Neurogenesis in 3D Traumatic Injury Model of Primary Human Cortical Astrocytes (part 2 of 3)
Source: Front Cell Neurosci. 2019 Feb 11;13:23. doi: 10.3389/fncel.2019.00023 (PMC6380212; doi:10.3389/fncel.2019.00023)

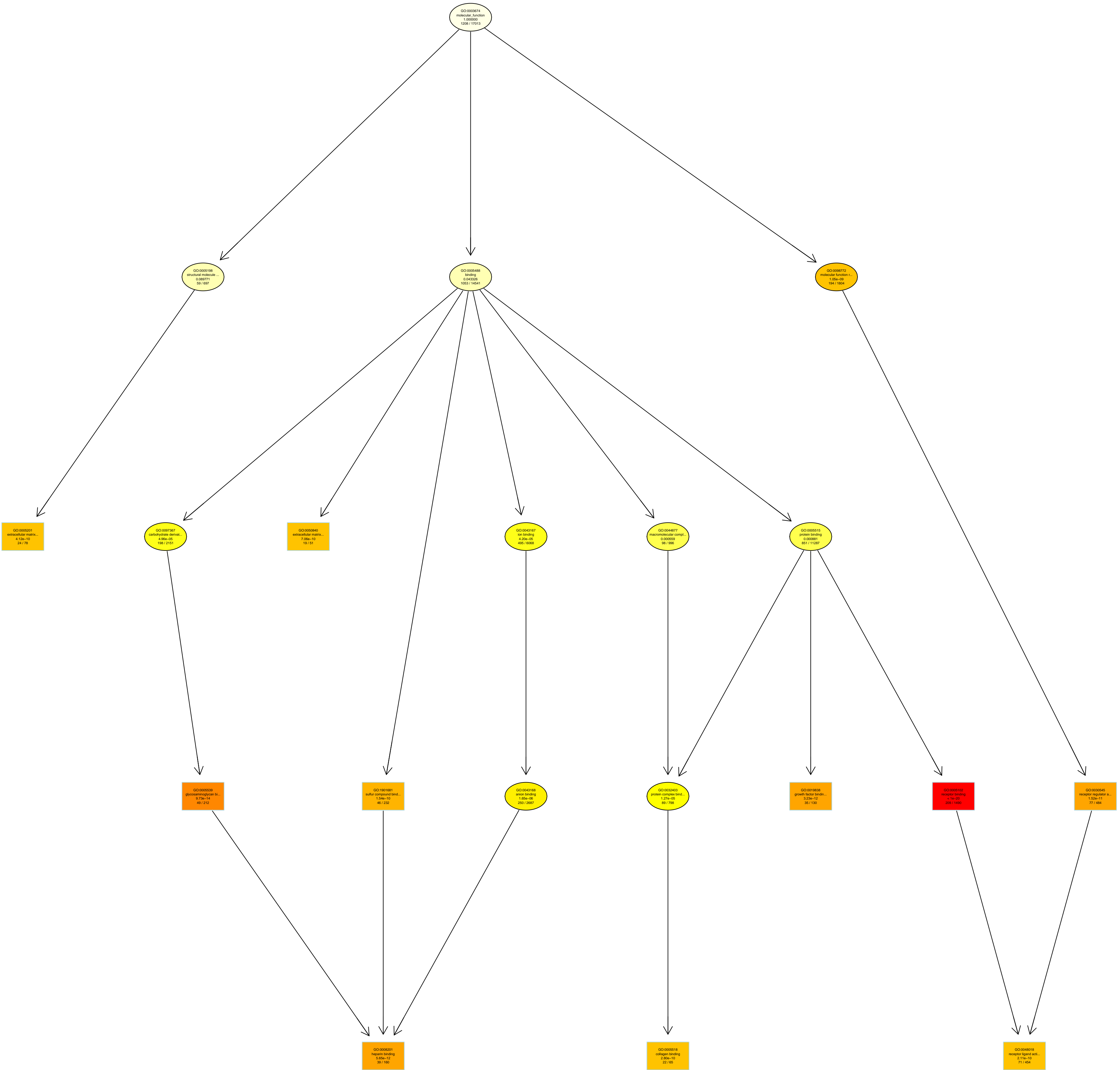

Supplement: DATASET S3 — GO-term analyses of GATA3-expressing and unscratched pHAs versus EGFP-expressing and unscratched pHAs in 2D cultures. [file Data_Sheet_3.ZIP › GO_term_analyses_GATA3u_vs_GFPu/topGO/topGO_MF_classicfisher_nodes.pdf]

topGO\_MF\_classicfisher\_pieChart

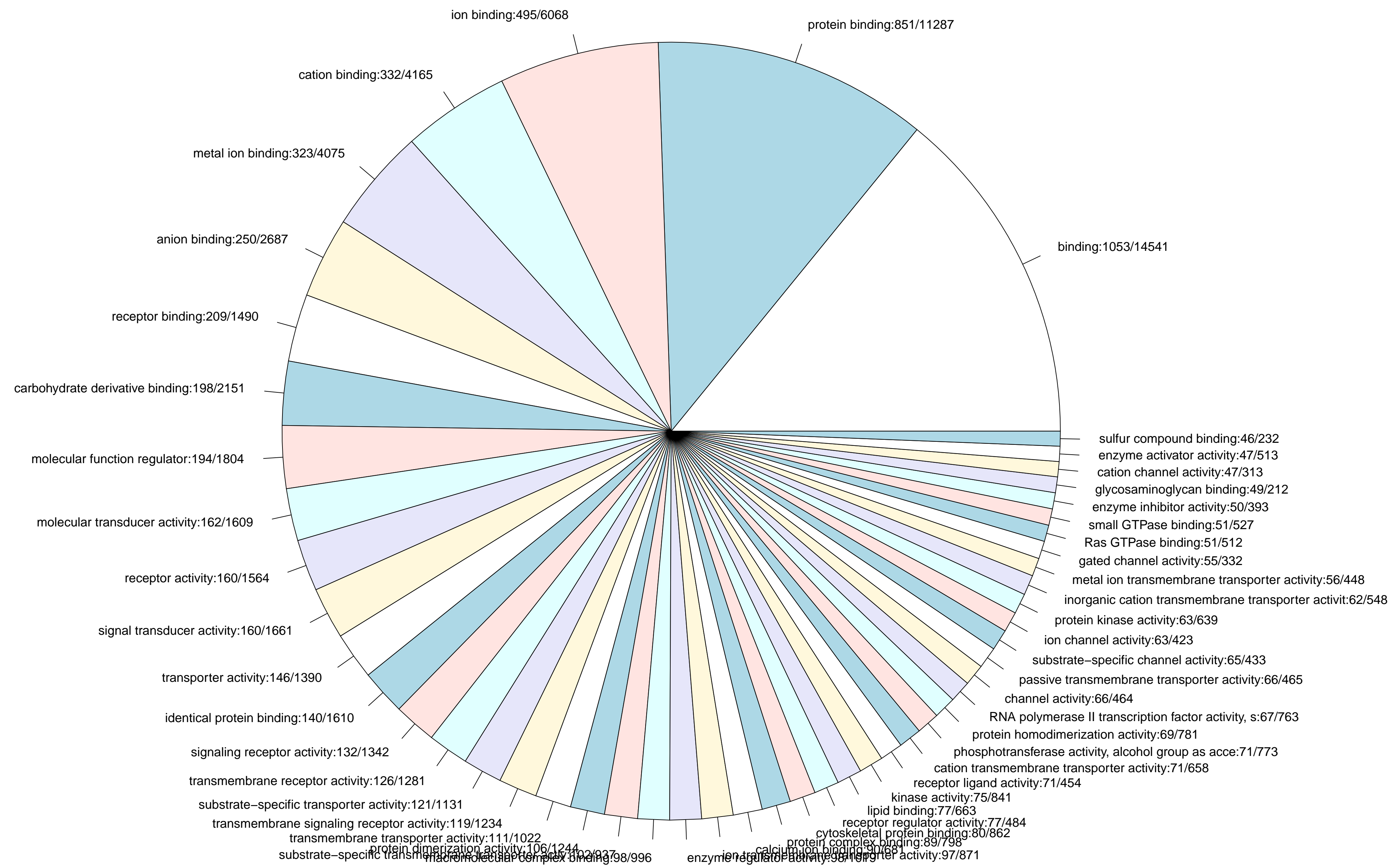

Supplement: DATASET S3 — GO-term analyses of GATA3-expressing and unscratched pHAs versus EGFP-expressing and unscratched pHAs in 2D cultures. [file Data_Sheet_3.ZIP › GO_term_analyses_GATA3u_vs_GFPu/topGO/topGO_MF_classicfisher_pieChart.pdf]

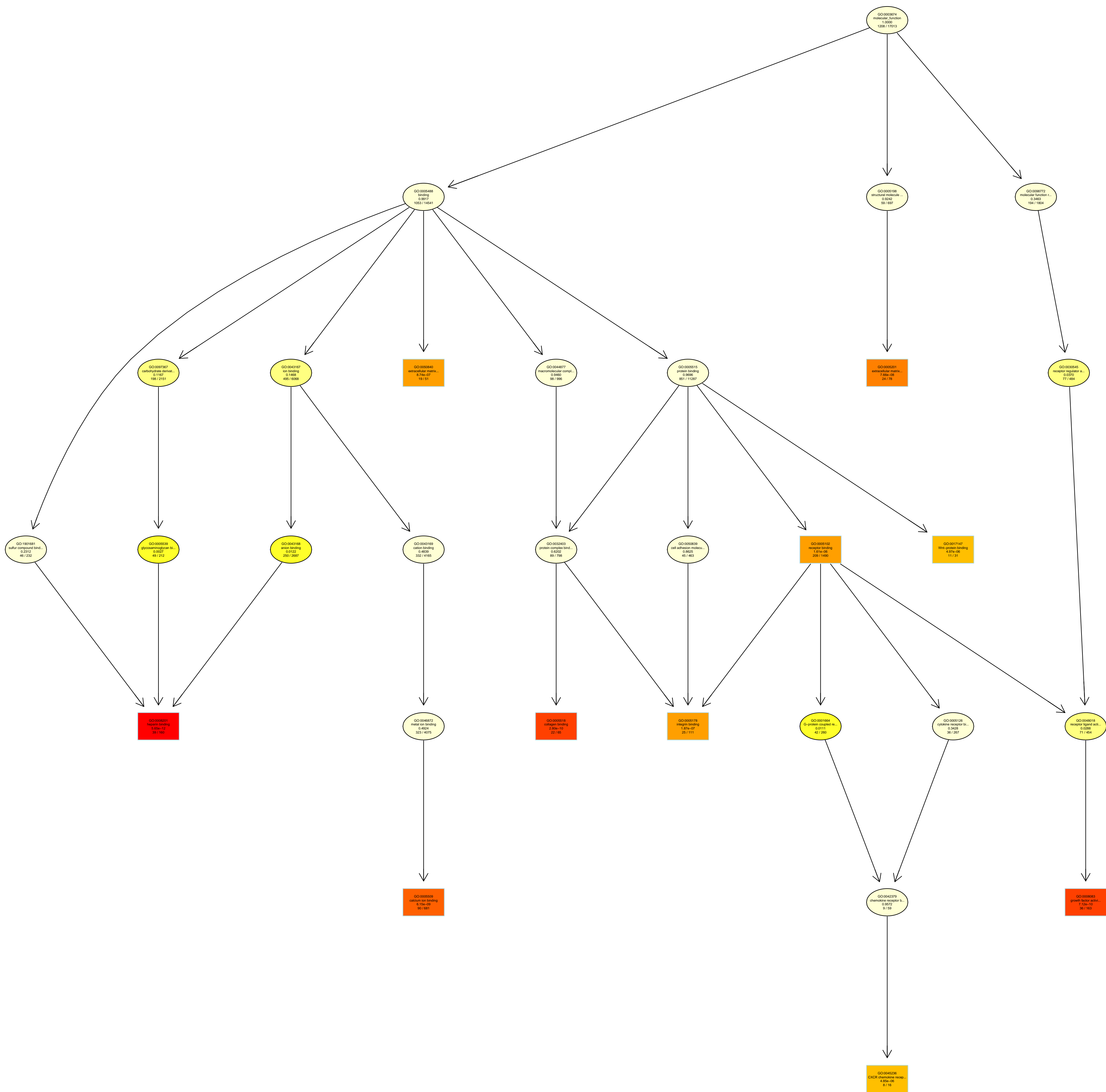

Supplement: DATASET S3 — GO-term analyses of GATA3-expressing and unscratched pHAs versus EGFP-expressing and unscratched pHAs in 2D cultures. [file Data_Sheet_3.ZIP › GO_term_analyses_GATA3u_vs_GFPu/topGO/topGO_MF_elimfisher_nodes.pdf]

topGO\_MF\_elimfisher\_pieChart

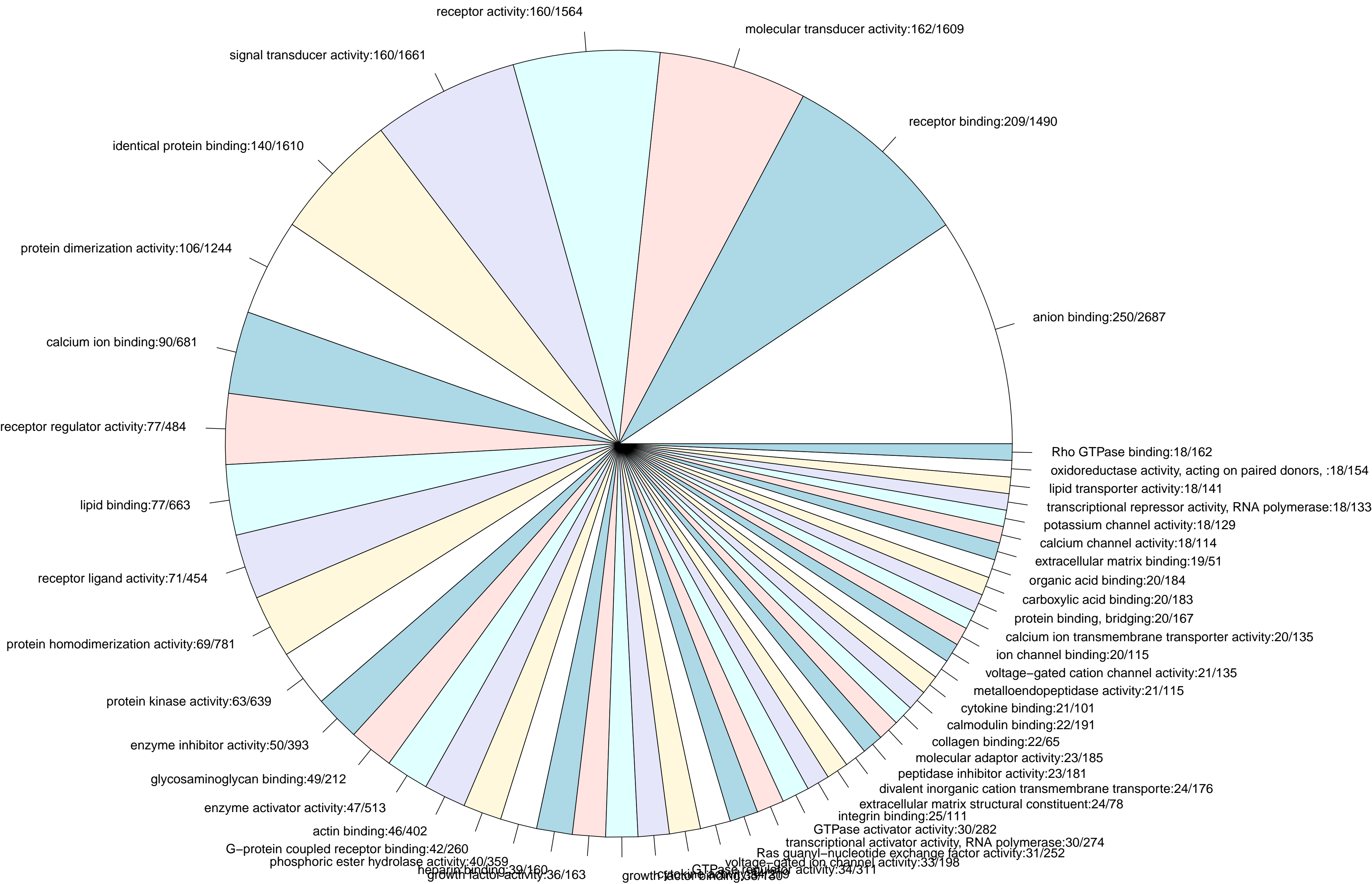

Supplement: DATASET S3 — GO-term analyses of GATA3-expressing and unscratched pHAs versus EGFP-expressing and unscratched pHAs in 2D cultures. [file Data_Sheet_3.ZIP › GO_term_analyses_GATA3u_vs_GFPu/topGO/topGO_MF_elimfisher_pieChart.pdf]

CP\_enrichGO\_BP

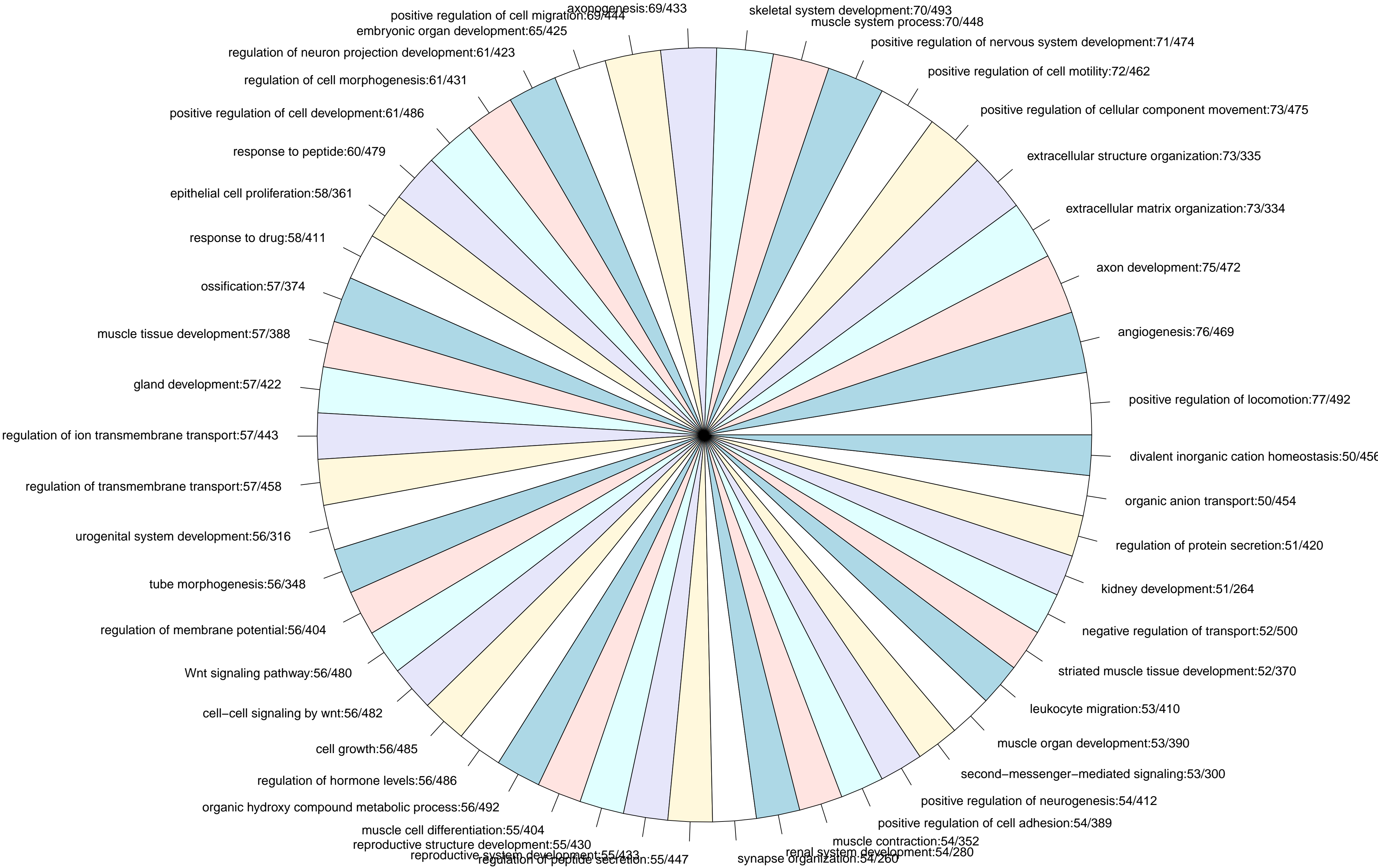

Supplement: DATASET S4 — GO-term analyses for GATA3-expressing and scratched pHAs versus EGFP-expressing and scratched pHAs in 2D cultures. [file Data_Sheet_4.ZIP › GO_term_analyses_GATA3s_vs_GFPs/clusterProfiler/CP_enrichGO_BP.pdf]

CP\_enrichGO\_CC

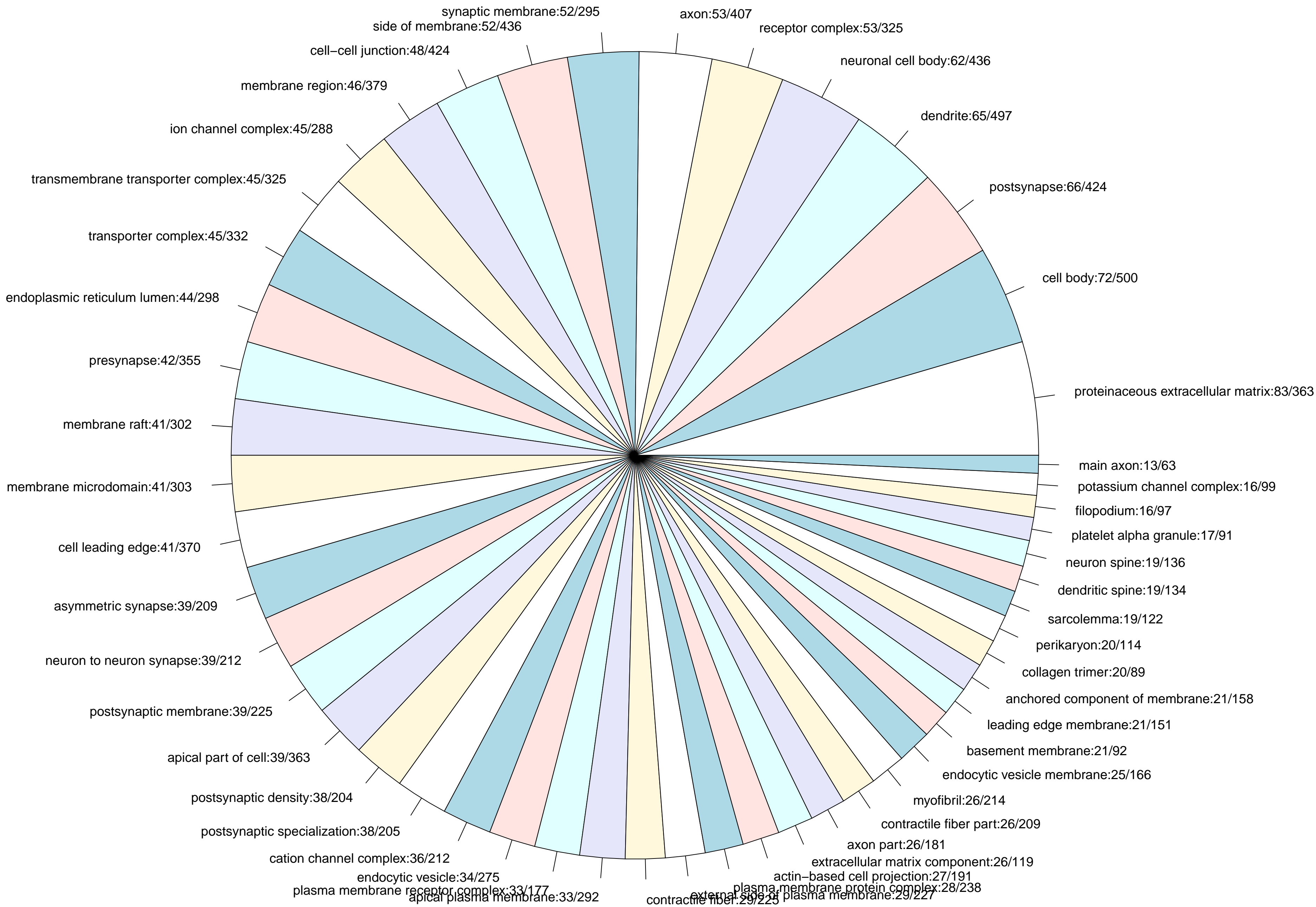

Supplement: DATASET S4 — GO-term analyses for GATA3-expressing and scratched pHAs versus EGFP-expressing and scratched pHAs in 2D cultures. [file Data_Sheet_4.ZIP › GO_term_analyses_GATA3s_vs_GFPs/clusterProfiler/CP_enrichGO_CC.pdf]

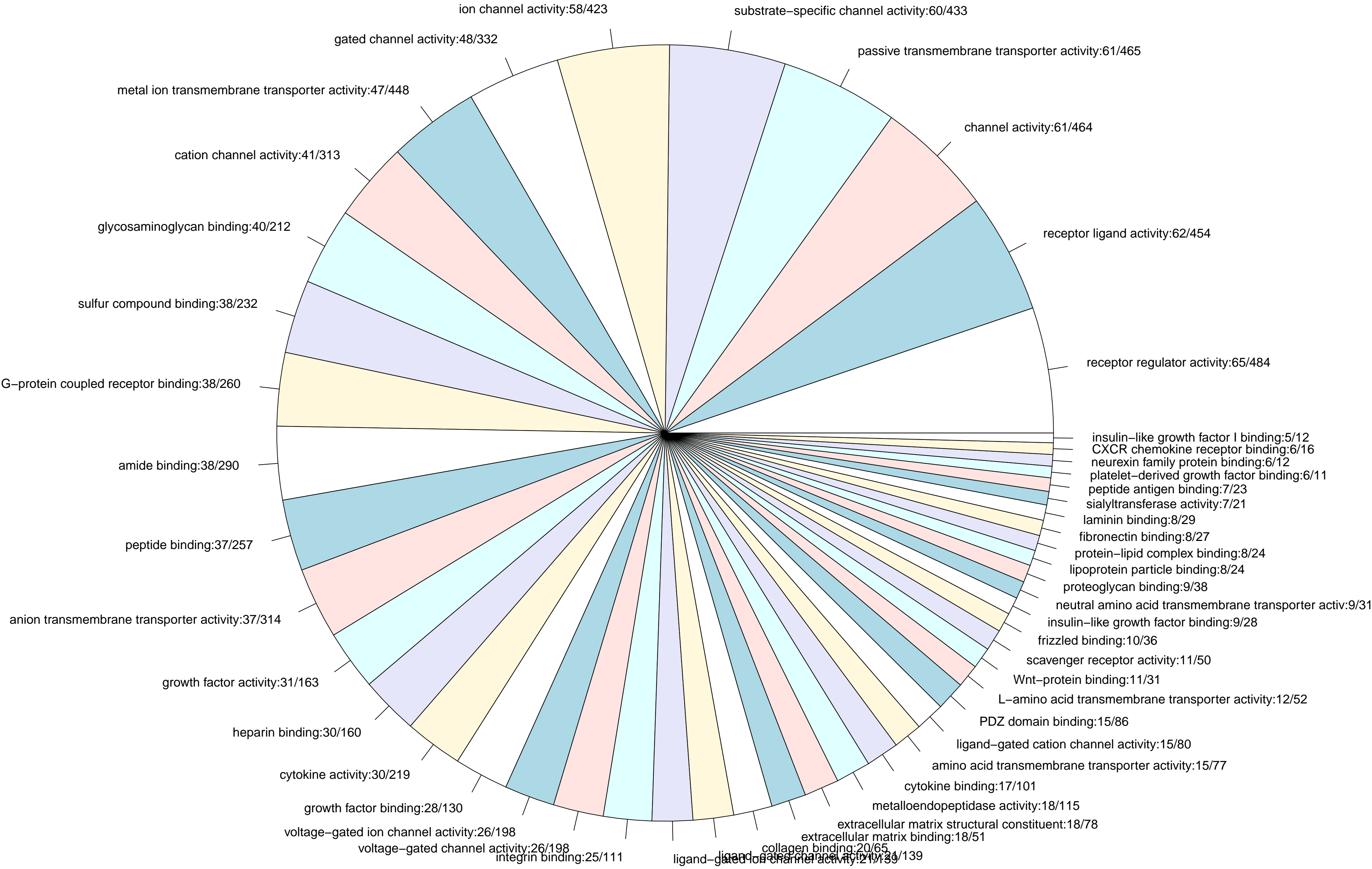

Supplement: DATASET S4 — GO-term analyses for GATA3-expressing and scratched pHAs versus EGFP-expressing and scratched pHAs in 2D cultures. [file Data_Sheet_4.ZIP › GO_term_analyses_GATA3s_vs_GFPs/clusterProfiler/CP_enrichGO_MF.pdf]

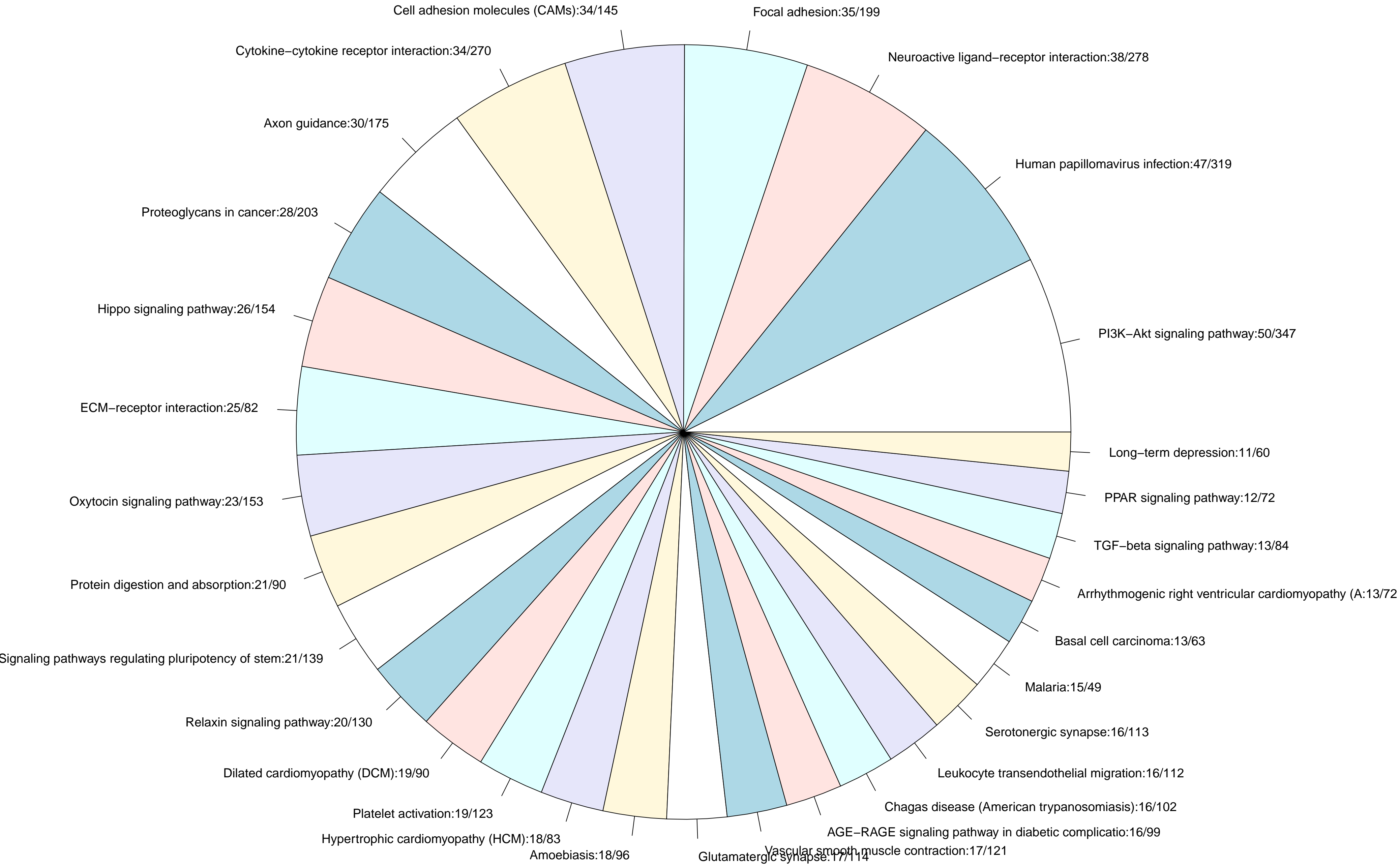

Supplement: DATASET S4 — GO-term analyses for GATA3-expressing and scratched pHAs versus EGFP-expressing and scratched pHAs in 2D cultures. [file Data_Sheet_4.ZIP › GO_term_analyses_GATA3s_vs_GFPs/clusterProfiler/CP_enrichKEGGS.pdf]

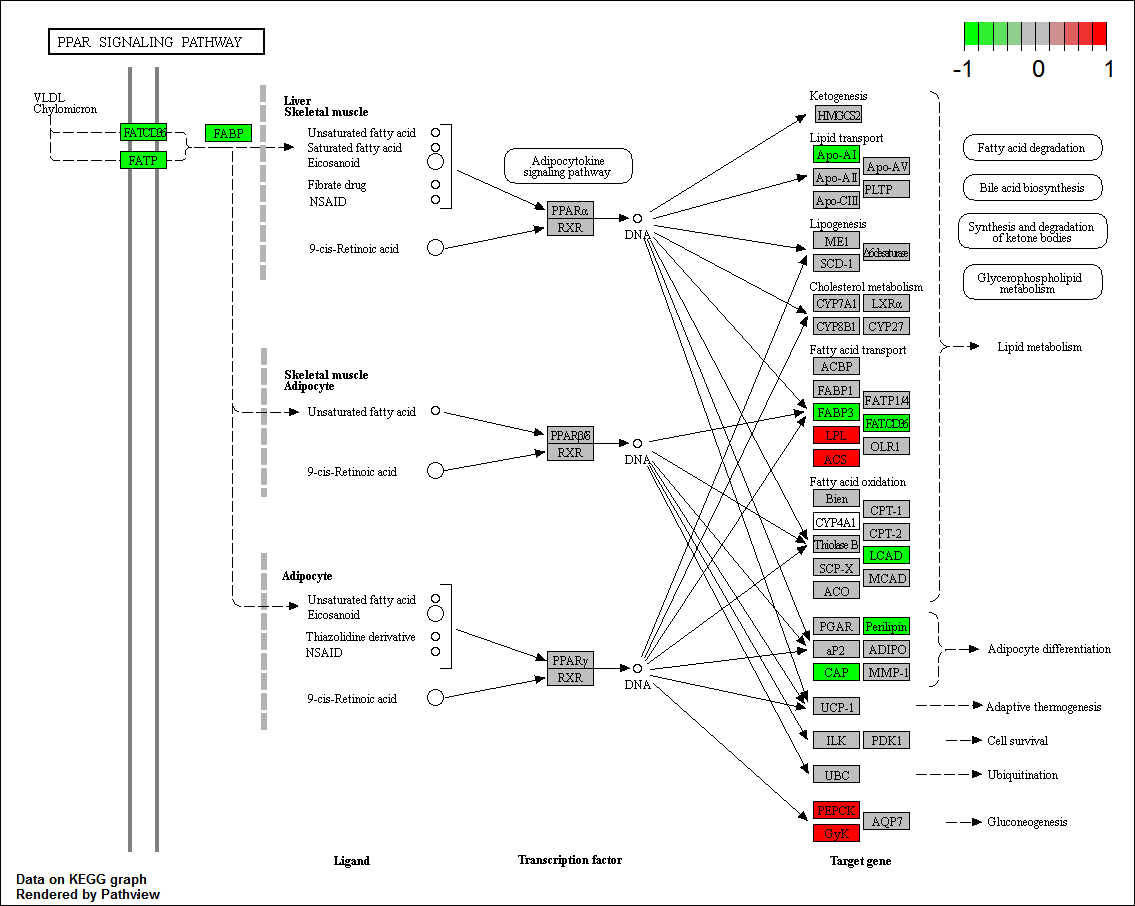

Supplement: DATASET S4 — GO-term analyses for GATA3-expressing and scratched pHAs versus EGFP-expressing and scratched pHAs in 2D cultures. [file Data_Sheet_4.ZIP › GO_term_analyses_GATA3s_vs_GFPs/clusterProfiler/hsa03320.PPARsignalingpathway.png]

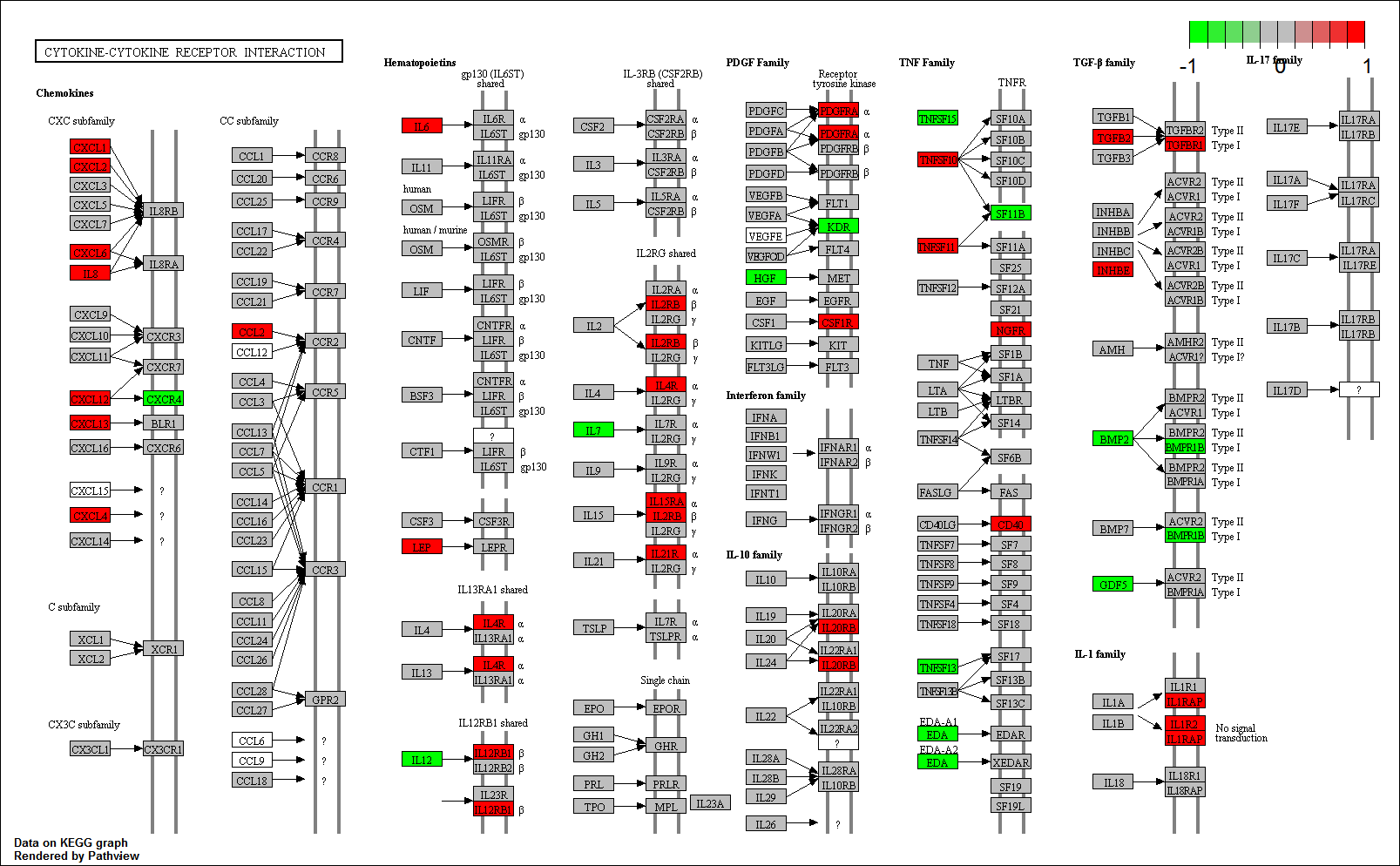

Supplement: DATASET S4 — GO-term analyses for GATA3-expressing and scratched pHAs versus EGFP-expressing and scratched pHAs in 2D cultures. [file Data_Sheet_4.ZIP › GO_term_analyses_GATA3s_vs_GFPs/clusterProfiler/hsa04060.Cytokine-cytokinereceptorinteraction.png]

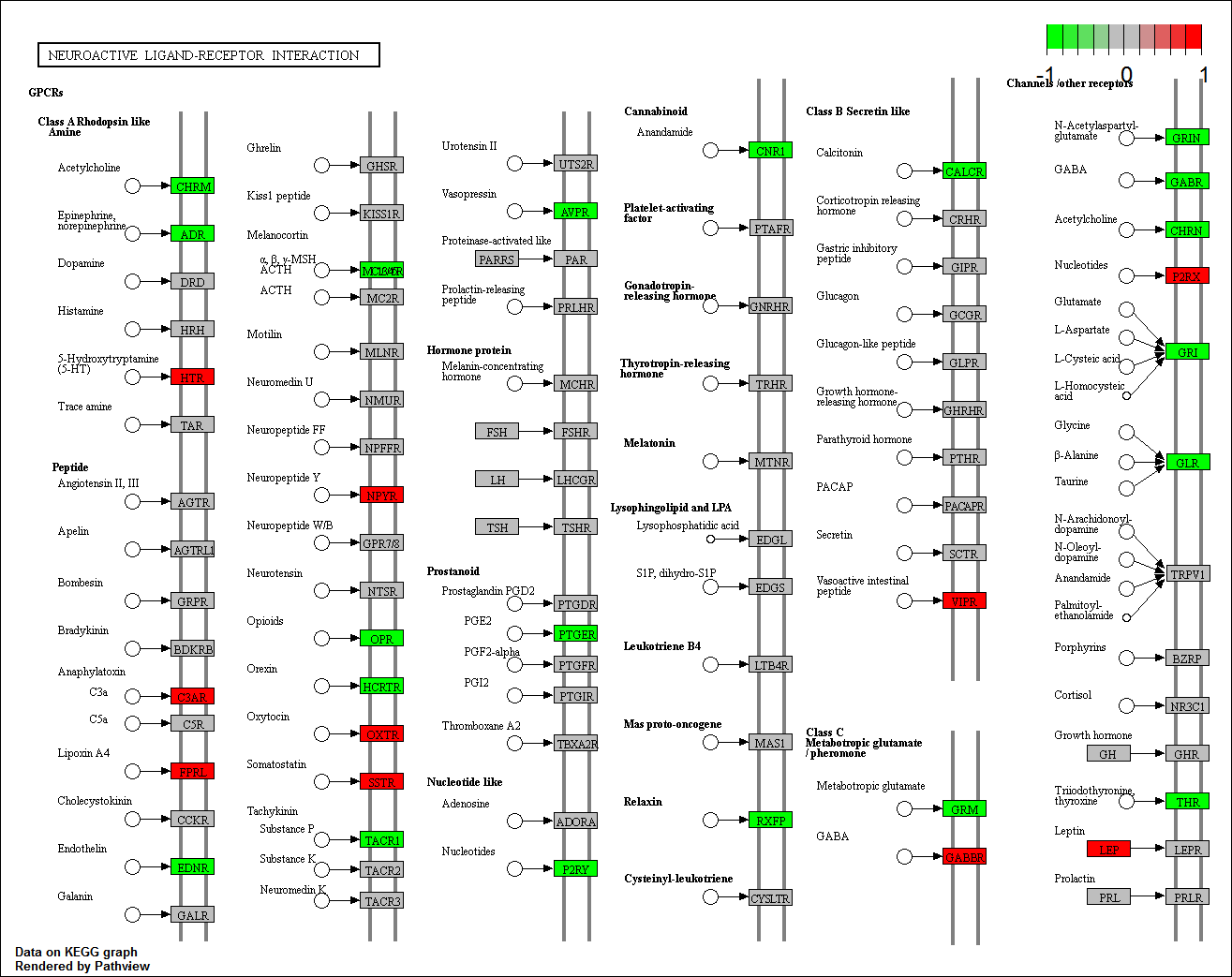

Supplement: DATASET S4 — GO-term analyses for GATA3-expressing and scratched pHAs versus EGFP-expressing and scratched pHAs in 2D cultures. [file Data_Sheet_4.ZIP › GO_term_analyses_GATA3s_vs_GFPs/clusterProfiler/hsa04080.Neuroactiveligand-receptorinteraction.png]

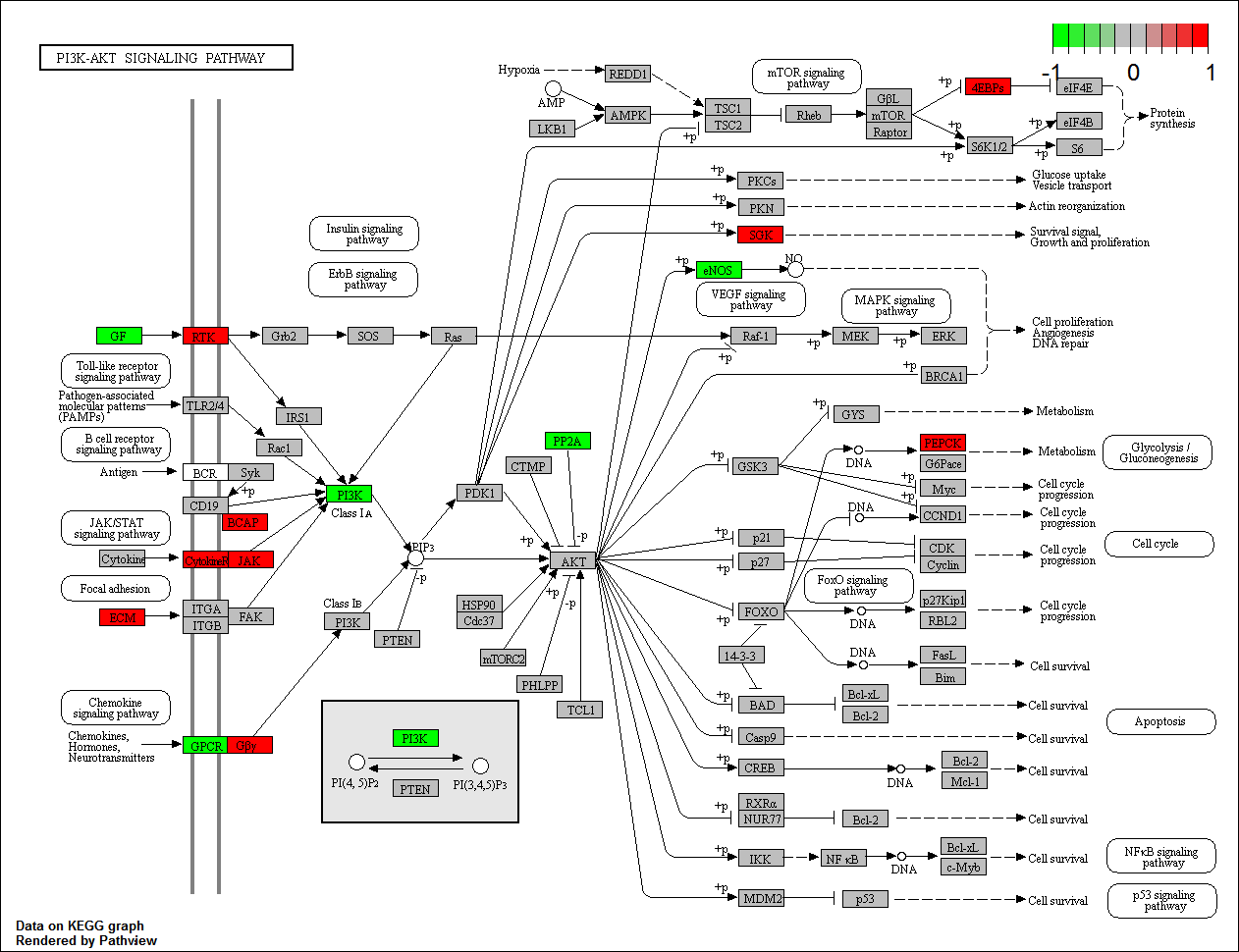

Supplement: DATASET S4 — GO-term analyses for GATA3-expressing and scratched pHAs versus EGFP-expressing and scratched pHAs in 2D cultures. [file Data_Sheet_4.ZIP › GO_term_analyses_GATA3s_vs_GFPs/clusterProfiler/hsa04151.PI3K-Aktsignalingpathway.png]

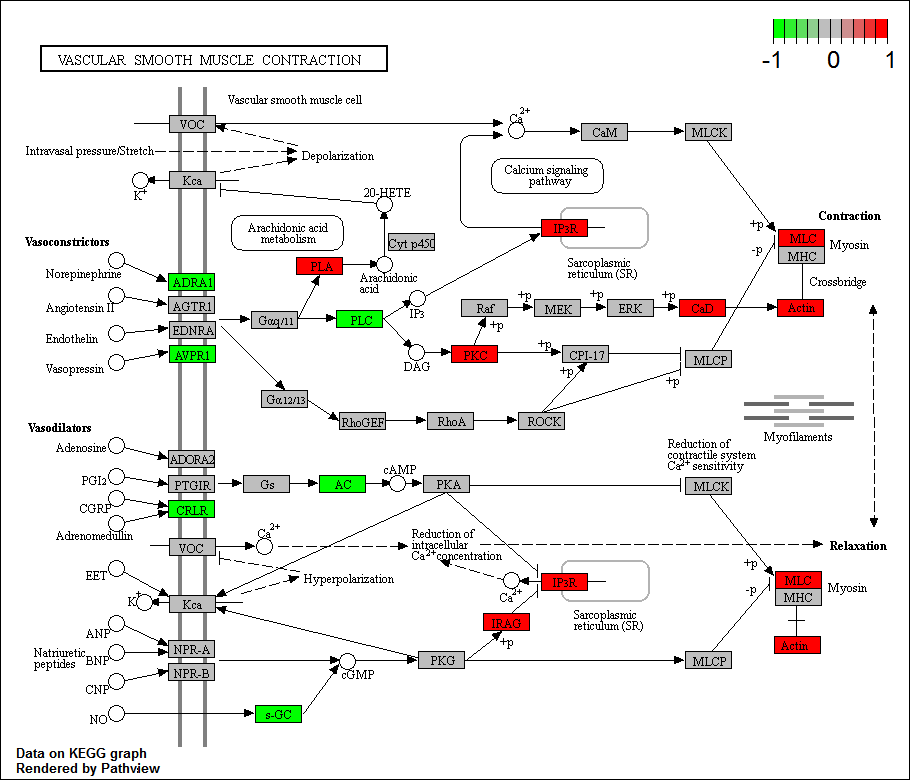

Supplement: DATASET S4 — GO-term analyses for GATA3-expressing and scratched pHAs versus EGFP-expressing and scratched pHAs in 2D cultures. [file Data_Sheet_4.ZIP › GO_term_analyses_GATA3s_vs_GFPs/clusterProfiler/hsa04270.Vascularsmoothmusclecontraction.png]

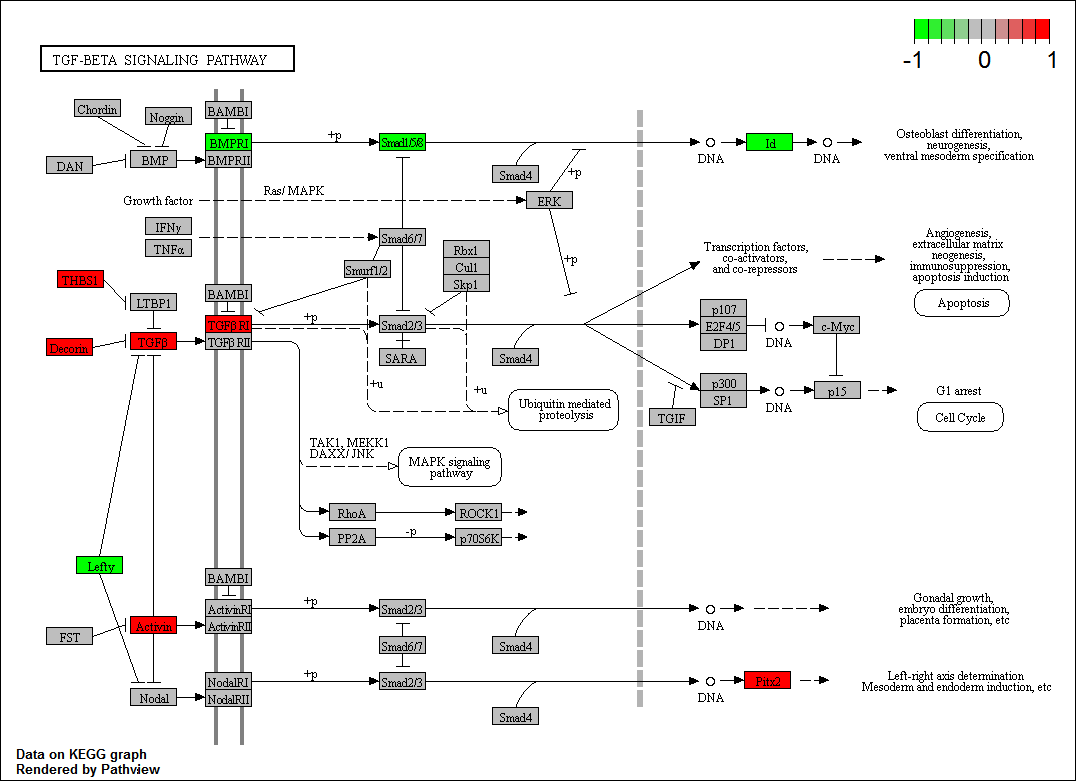

Supplement: DATASET S4 — GO-term analyses for GATA3-expressing and scratched pHAs versus EGFP-expressing and scratched pHAs in 2D cultures. [file Data_Sheet_4.ZIP › GO_term_analyses_GATA3s_vs_GFPs/clusterProfiler/hsa04350.TGF-betasignalingpathway.png]

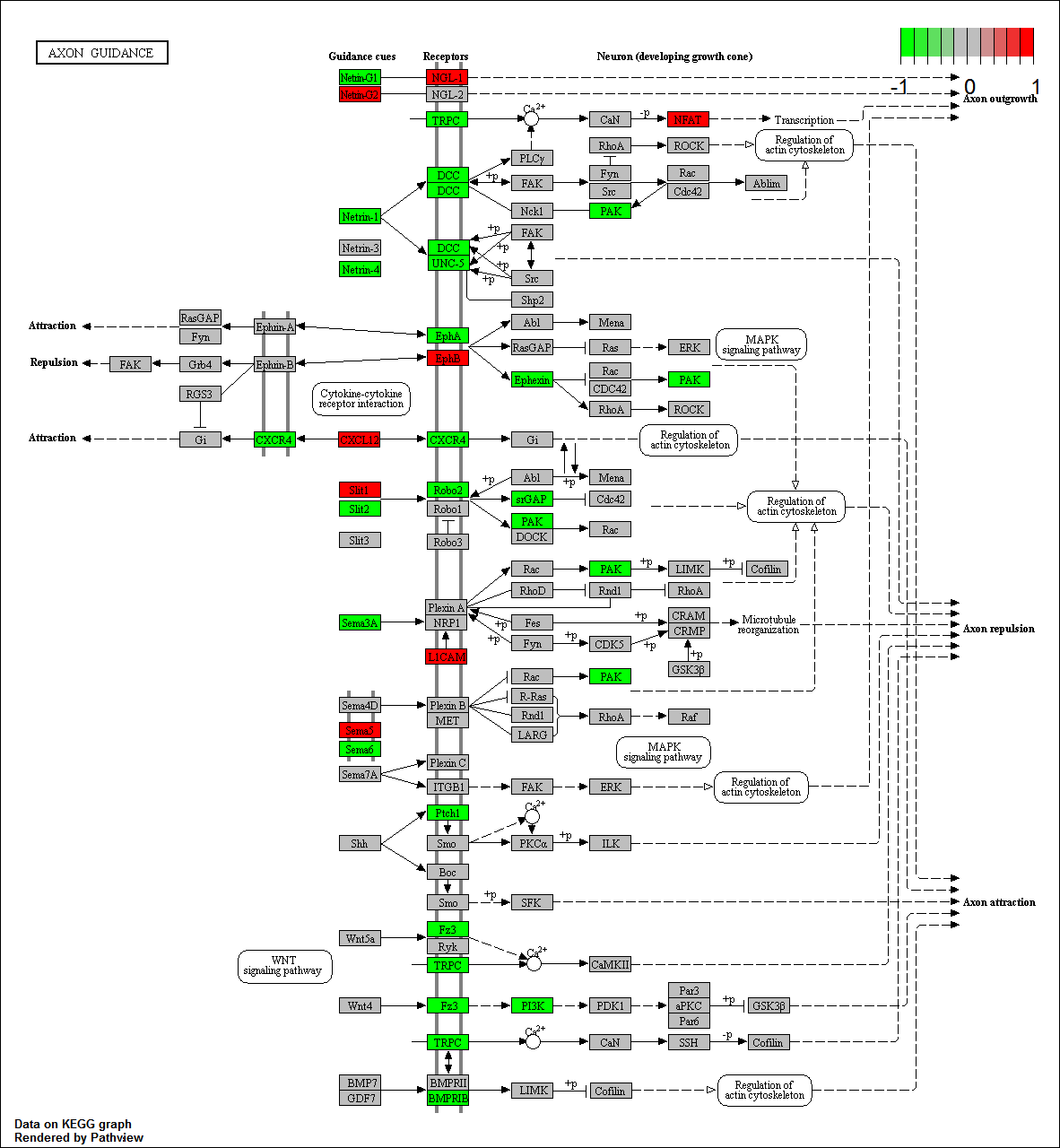

Supplement: DATASET S4 — GO-term analyses for GATA3-expressing and scratched pHAs versus EGFP-expressing and scratched pHAs in 2D cultures. [file Data_Sheet_4.ZIP › GO_term_analyses_GATA3s_vs_GFPs/clusterProfiler/hsa04360.Axonguidance.png]

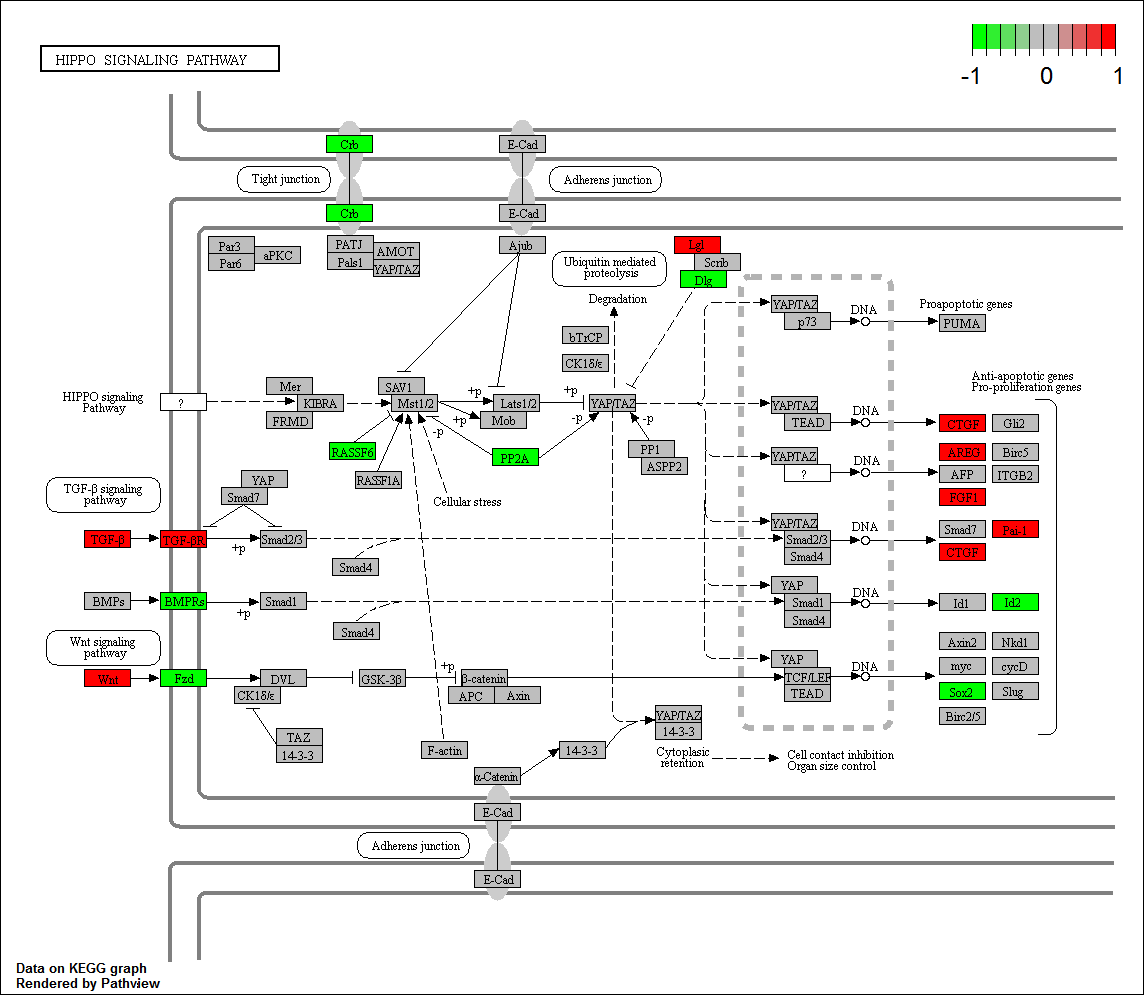

Supplement: DATASET S4 — GO-term analyses for GATA3-expressing and scratched pHAs versus EGFP-expressing and scratched pHAs in 2D cultures. [file Data_Sheet_4.ZIP › GO_term_analyses_GATA3s_vs_GFPs/clusterProfiler/hsa04390.Hipposignalingpathway.png]

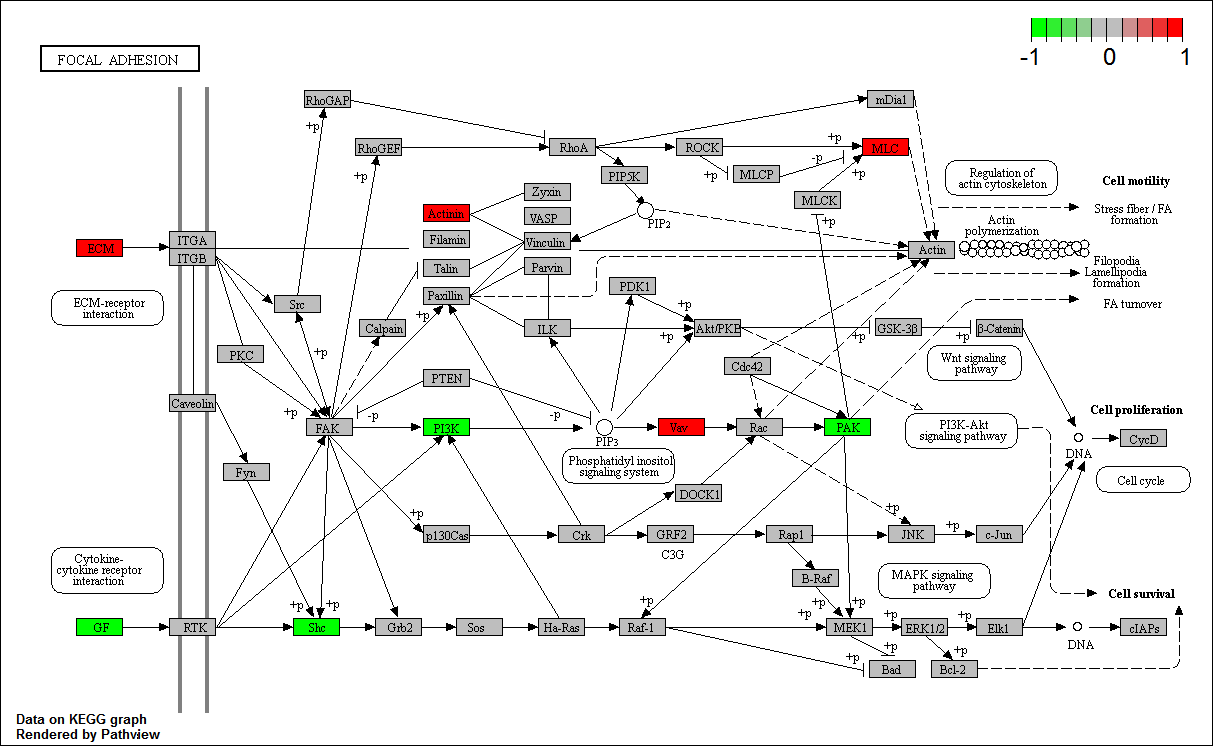

Supplement: DATASET S4 — GO-term analyses for GATA3-expressing and scratched pHAs versus EGFP-expressing and scratched pHAs in 2D cultures. [file Data_Sheet_4.ZIP › GO_term_analyses_GATA3s_vs_GFPs/clusterProfiler/hsa04510.Focaladhesion.png]

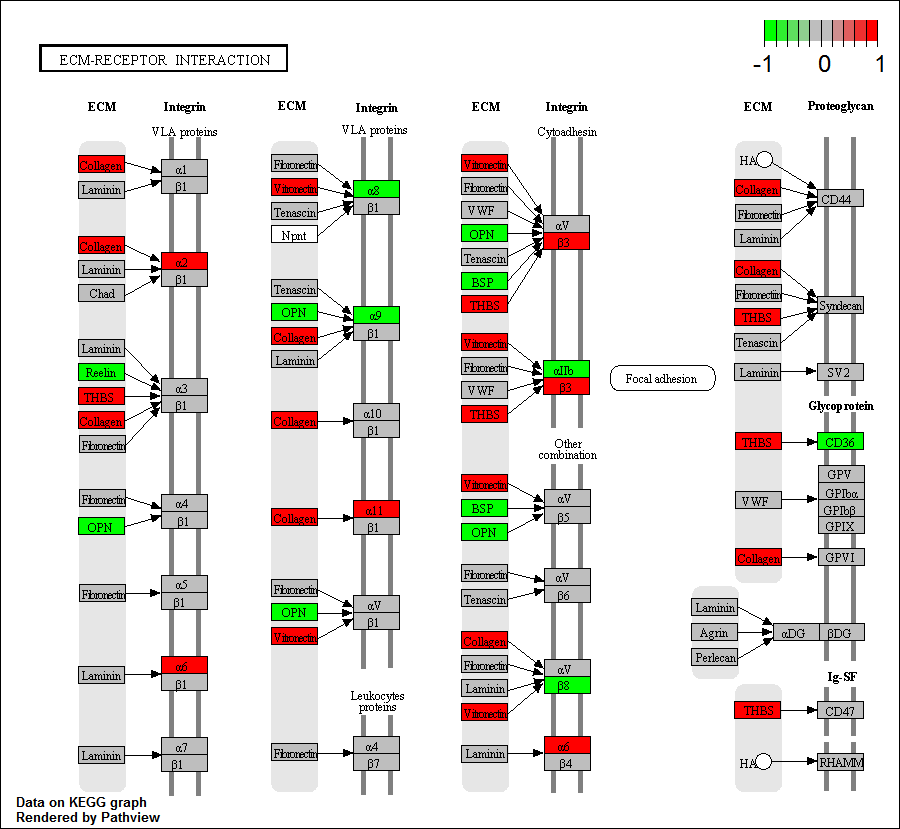

Supplement: DATASET S4 — GO-term analyses for GATA3-expressing and scratched pHAs versus EGFP-expressing and scratched pHAs in 2D cultures. [file Data_Sheet_4.ZIP › GO_term_analyses_GATA3s_vs_GFPs/clusterProfiler/hsa04512.ECM-receptorinteraction.png]

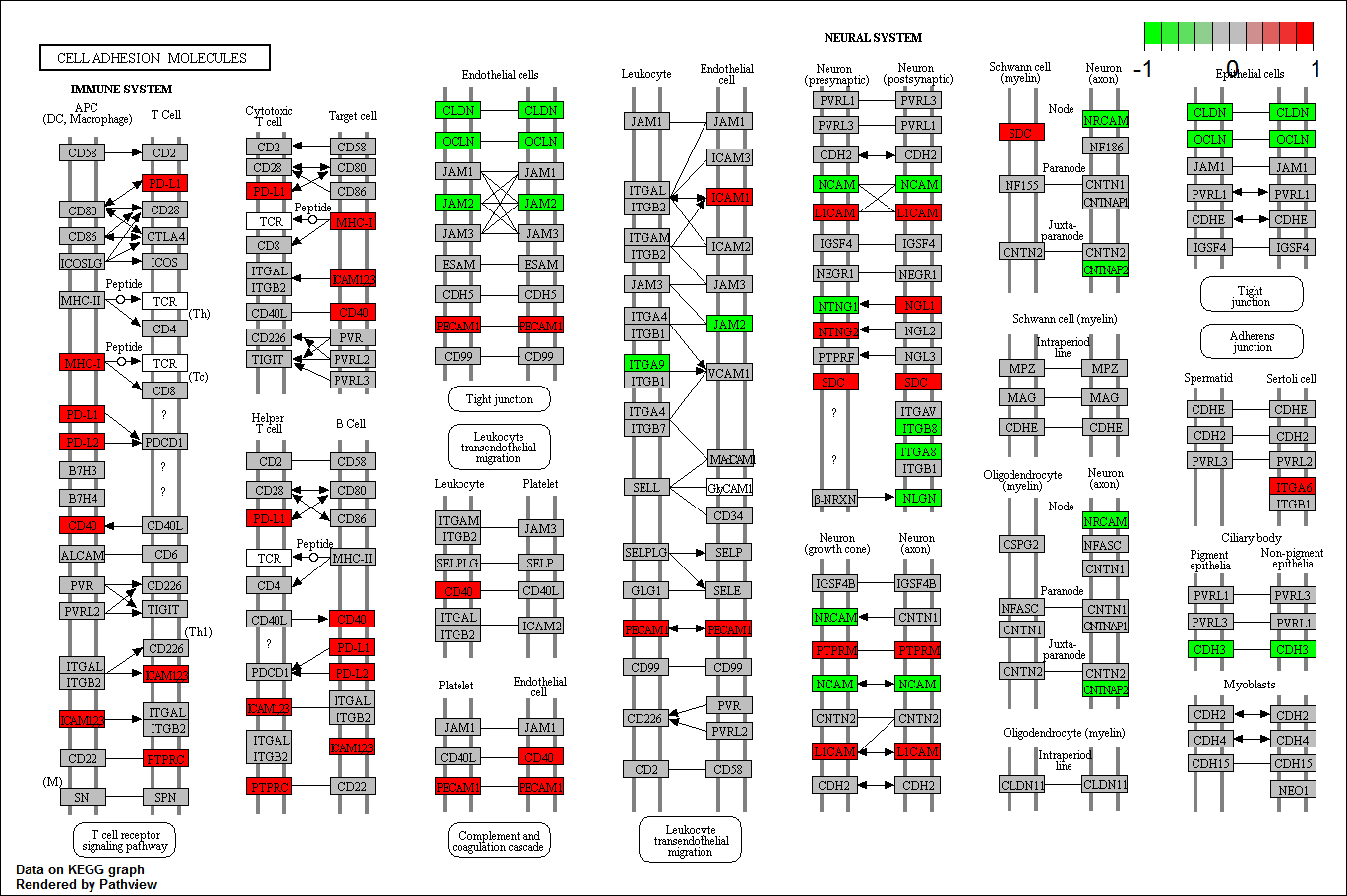

Supplement: DATASET S4 — GO-term analyses for GATA3-expressing and scratched pHAs versus EGFP-expressing and scratched pHAs in 2D cultures. [file Data_Sheet_4.ZIP › GO_term_analyses_GATA3s_vs_GFPs/clusterProfiler/hsa04514.Celladhesionmolecules(CAMs).png]

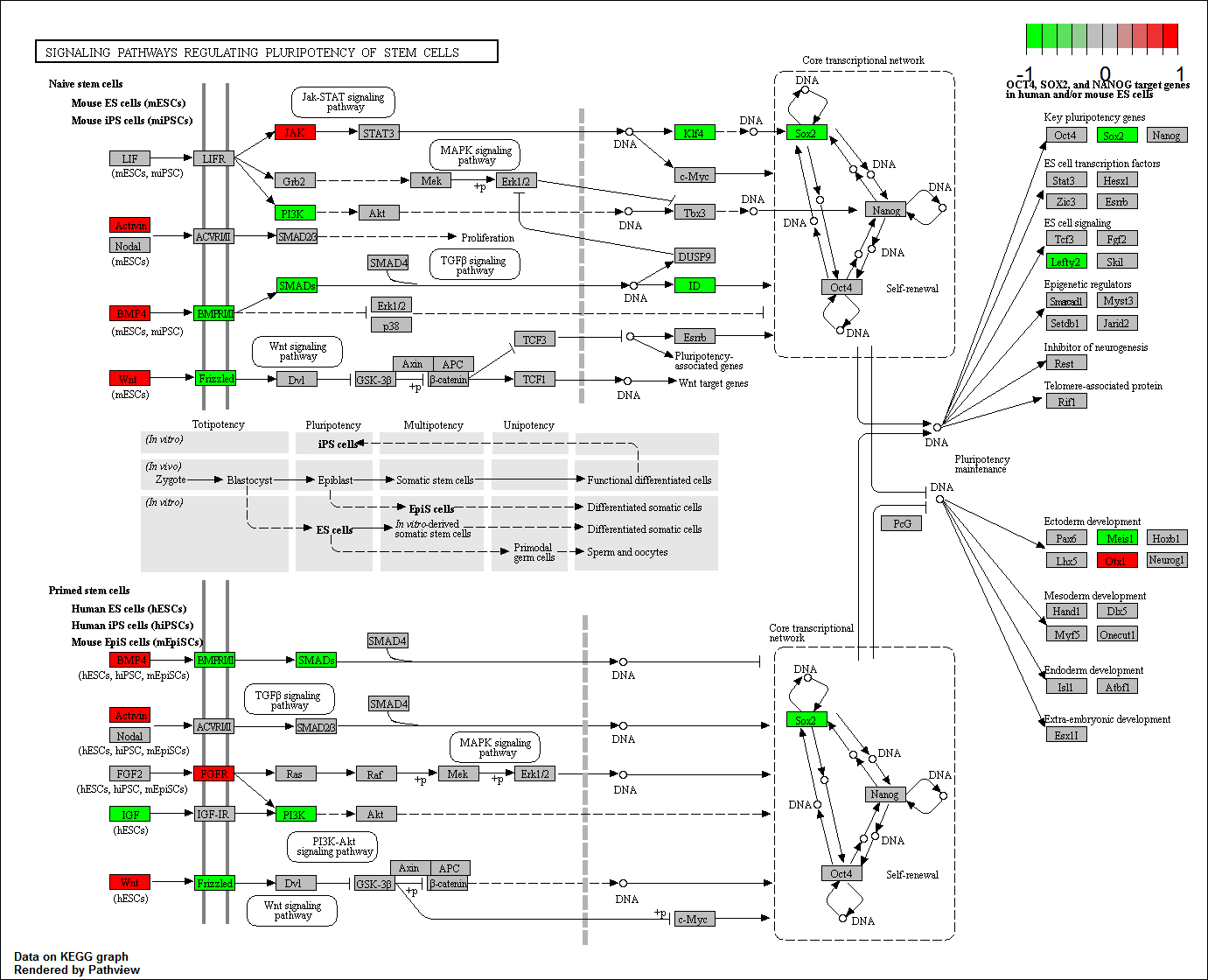

Supplement: DATASET S4 — GO-term analyses for GATA3-expressing and scratched pHAs versus EGFP-expressing and scratched pHAs in 2D cultures. [file Data_Sheet_4.ZIP › GO_term_analyses_GATA3s_vs_GFPs/clusterProfiler/hsa04550.Signalingpathwaysregulatingpluripotencyofstemcells.png]

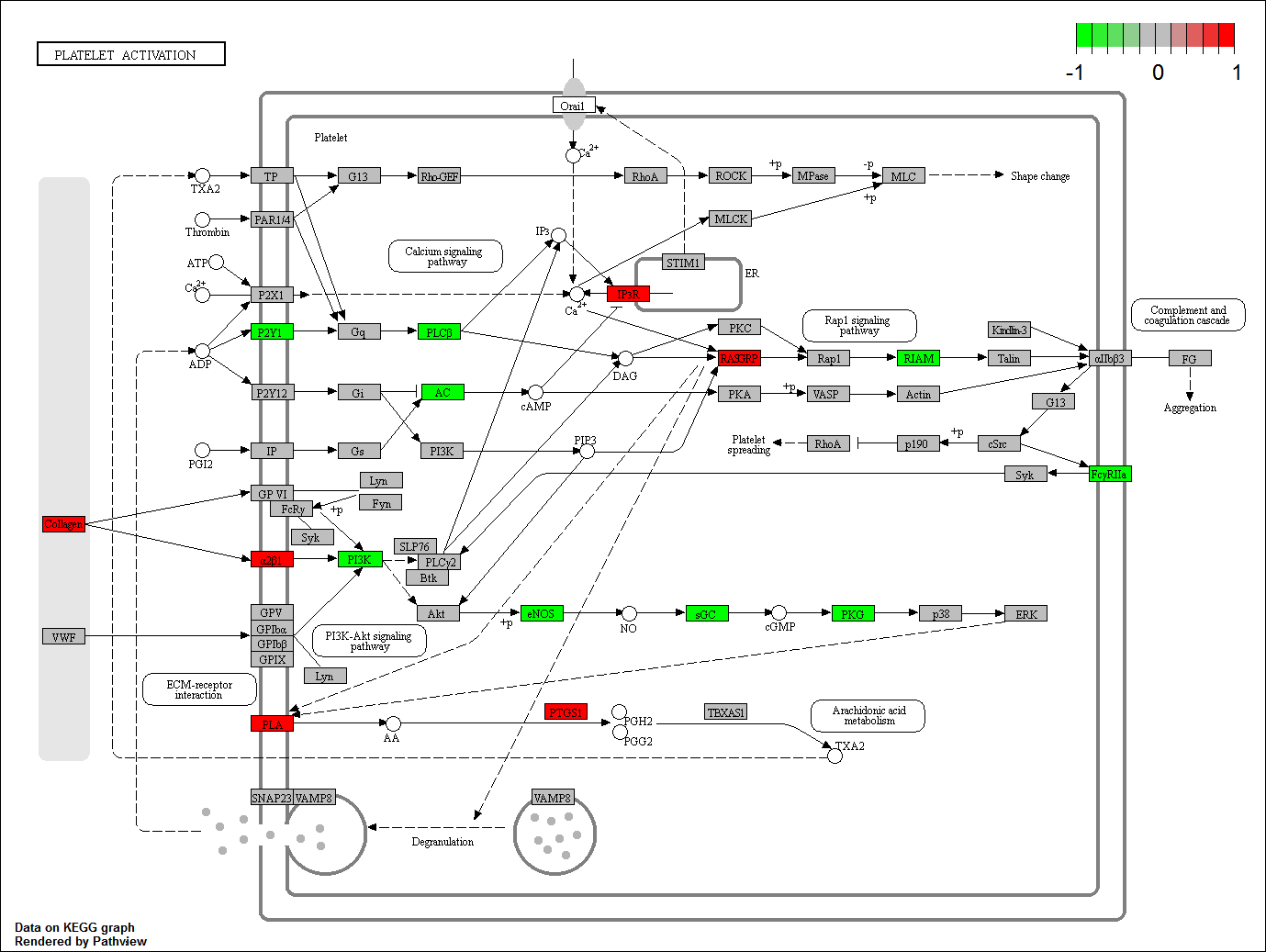

Supplement: DATASET S4 — GO-term analyses for GATA3-expressing and scratched pHAs versus EGFP-expressing and scratched pHAs in 2D cultures. [file Data_Sheet_4.ZIP › GO_term_analyses_GATA3s_vs_GFPs/clusterProfiler/hsa04611.Plateletactivation.png]

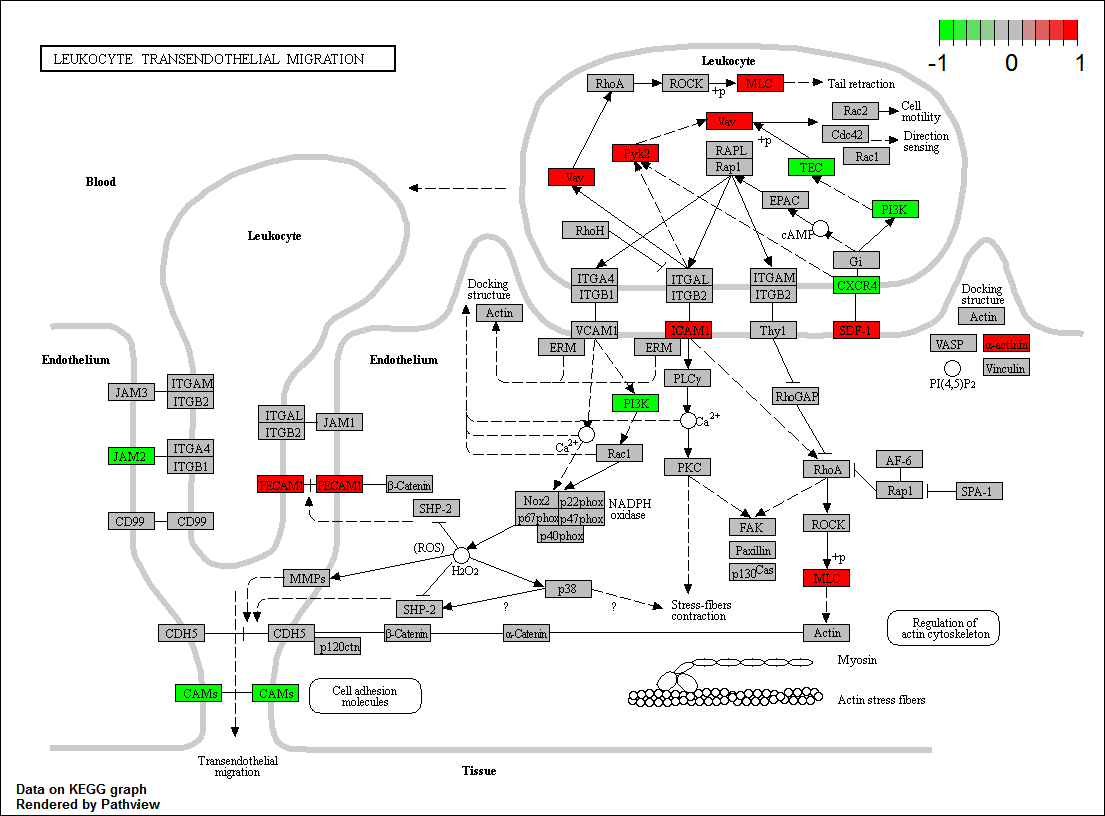

Supplement: DATASET S4 — GO-term analyses for GATA3-expressing and scratched pHAs versus EGFP-expressing and scratched pHAs in 2D cultures. [file Data_Sheet_4.ZIP › GO_term_analyses_GATA3s_vs_GFPs/clusterProfiler/hsa04670.Leukocytetransendothelialmigration.png]

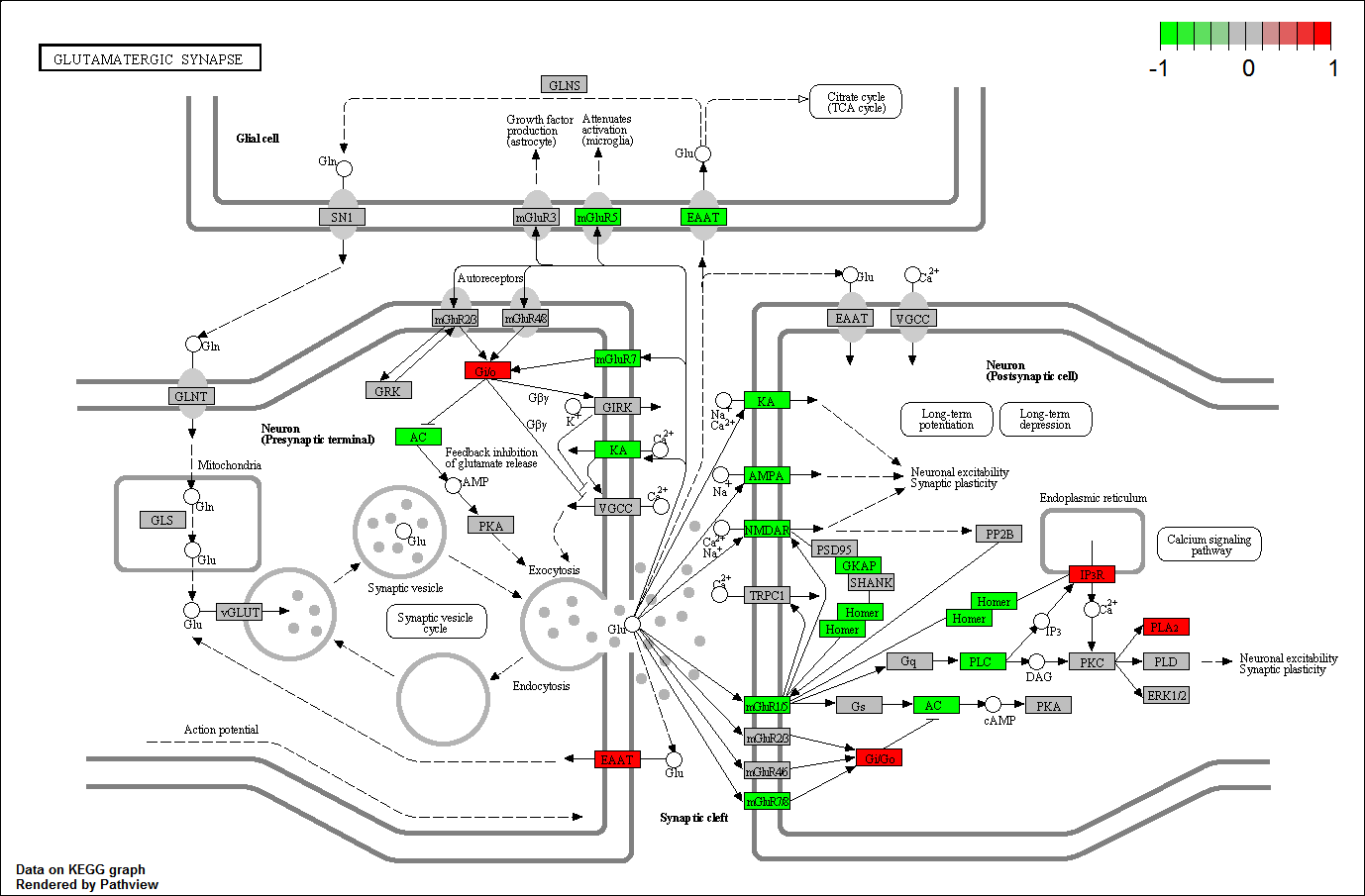

Supplement: DATASET S4 — GO-term analyses for GATA3-expressing and scratched pHAs versus EGFP-expressing and scratched pHAs in 2D cultures. [file Data_Sheet_4.ZIP › GO_term_analyses_GATA3s_vs_GFPs/clusterProfiler/hsa04724.Glutamatergicsynapse.png]

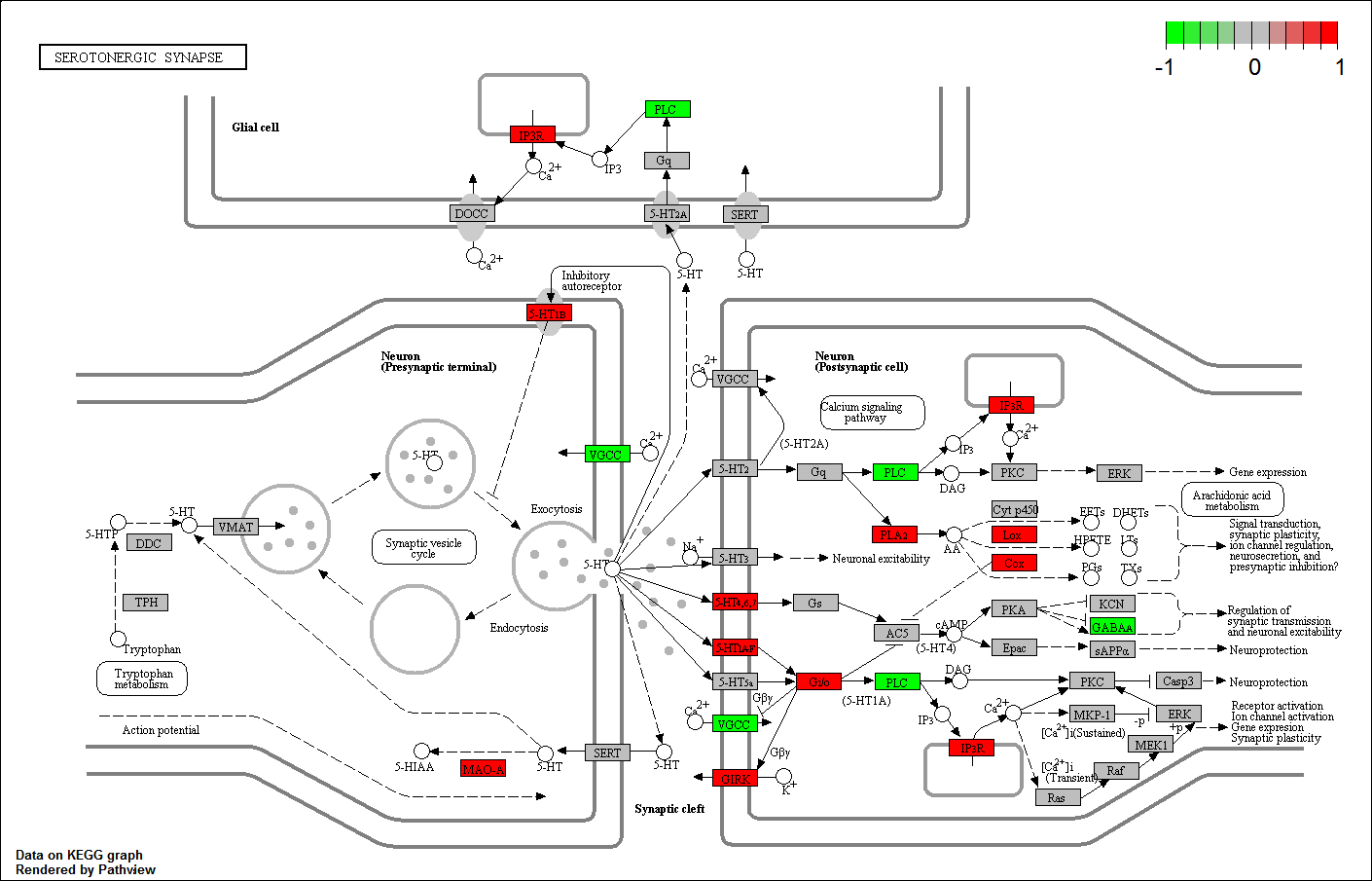

Supplement: DATASET S4 — GO-term analyses for GATA3-expressing and scratched pHAs versus EGFP-expressing and scratched pHAs in 2D cultures. [file Data_Sheet_4.ZIP › GO_term_analyses_GATA3s_vs_GFPs/clusterProfiler/hsa04726.Serotonergicsynapse.png]

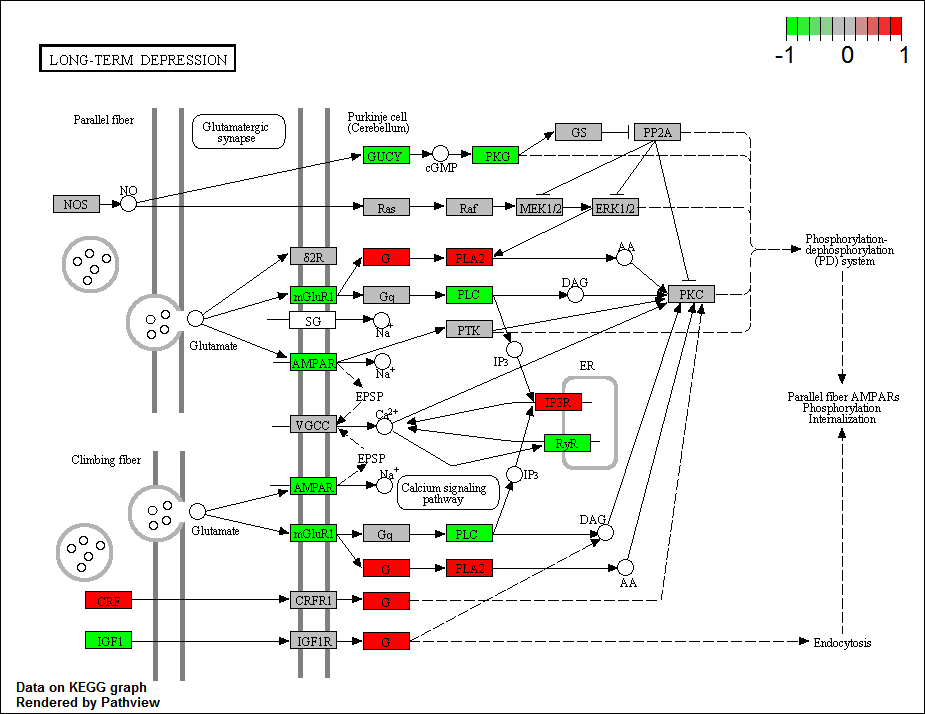

Supplement: DATASET S4 — GO-term analyses for GATA3-expressing and scratched pHAs versus EGFP-expressing and scratched pHAs in 2D cultures. [file Data_Sheet_4.ZIP › GO_term_analyses_GATA3s_vs_GFPs/clusterProfiler/hsa04730.Long-termdepression.png]

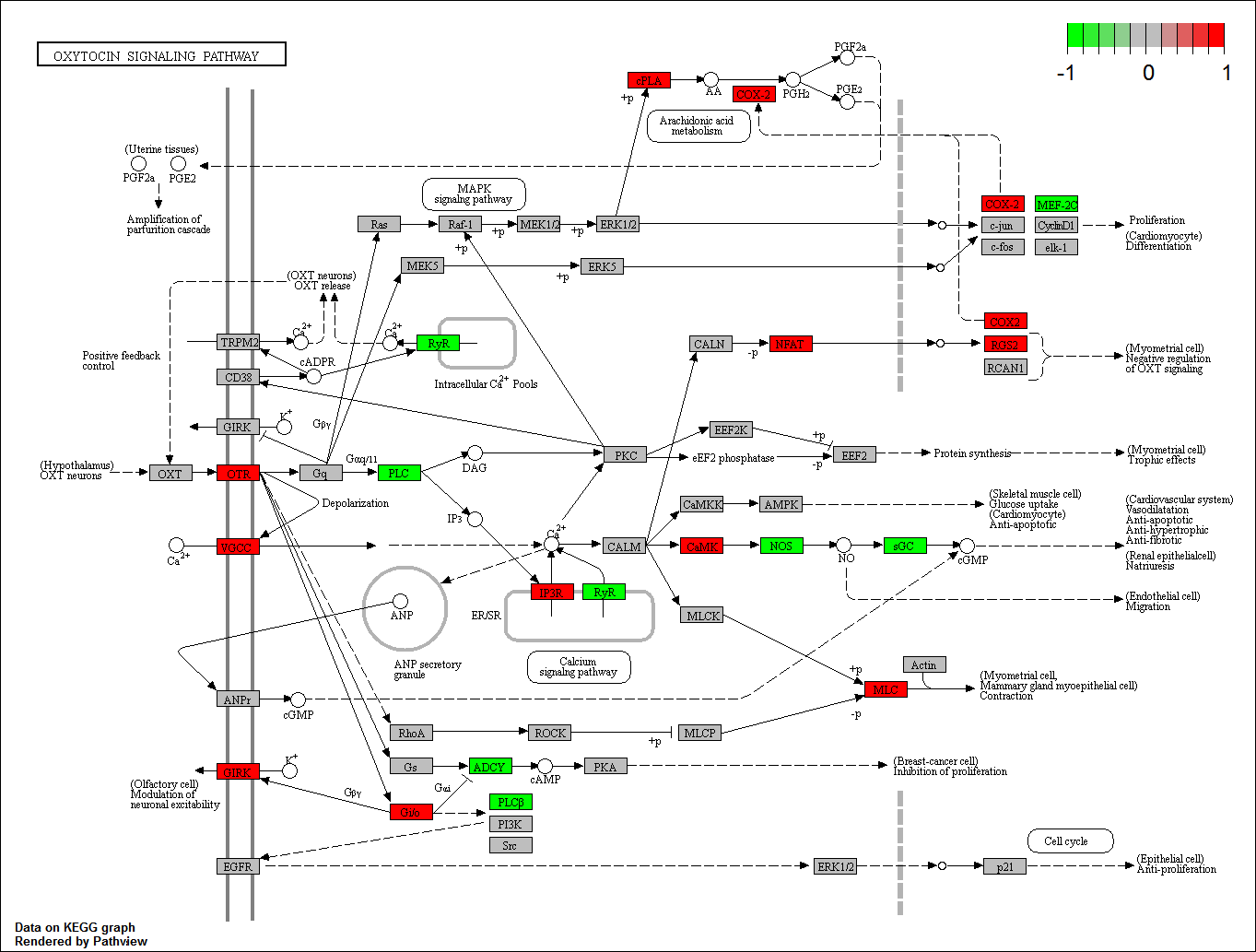

Supplement: DATASET S4 — GO-term analyses for GATA3-expressing and scratched pHAs versus EGFP-expressing and scratched pHAs in 2D cultures. [file Data_Sheet_4.ZIP › GO_term_analyses_GATA3s_vs_GFPs/clusterProfiler/hsa04921.Oxytocinsignalingpathway.png]

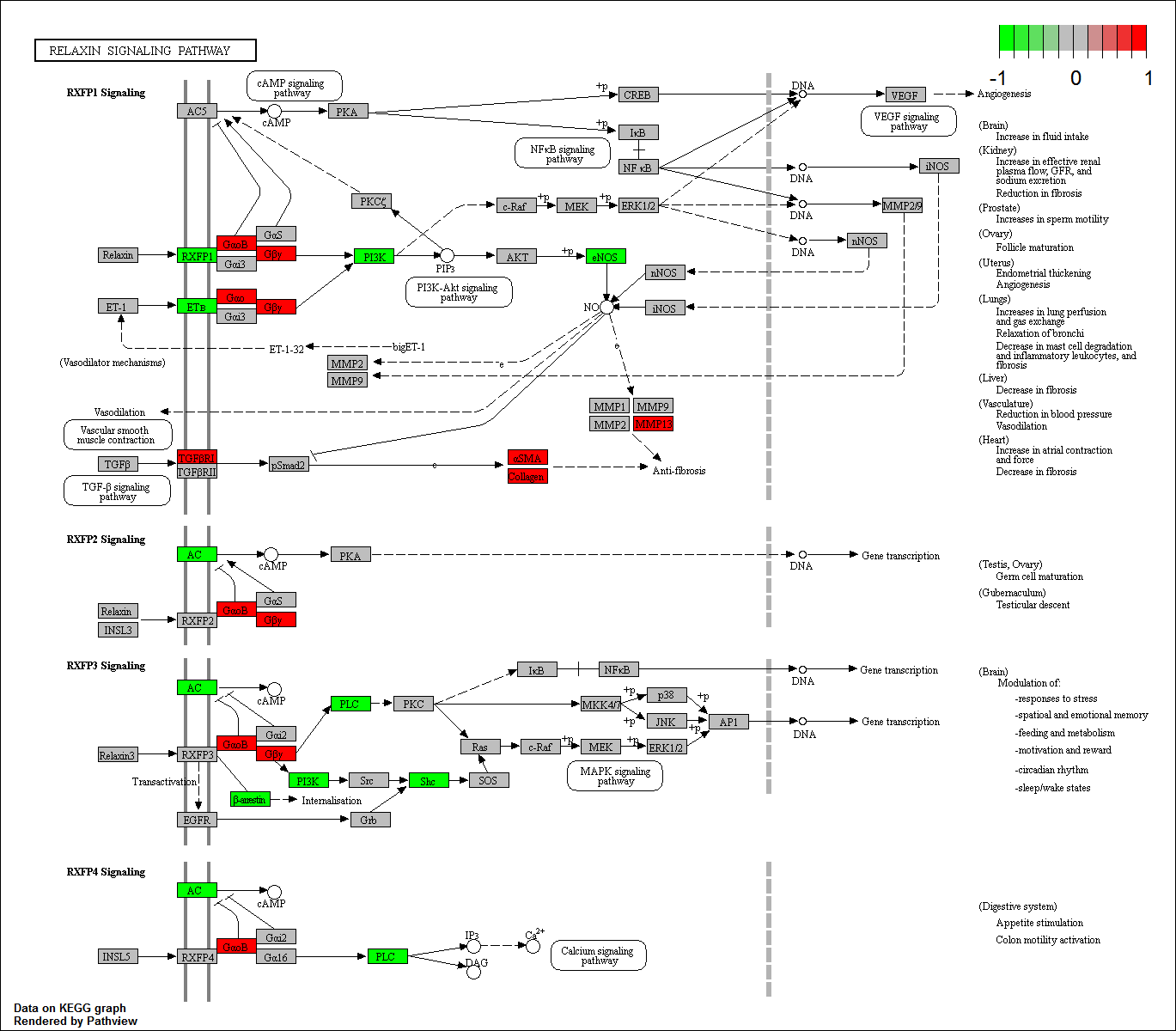

Supplement: DATASET S4 — GO-term analyses for GATA3-expressing and scratched pHAs versus EGFP-expressing and scratched pHAs in 2D cultures. [file Data_Sheet_4.ZIP › GO_term_analyses_GATA3s_vs_GFPs/clusterProfiler/hsa04926.Relaxinsignalingpathway.png]

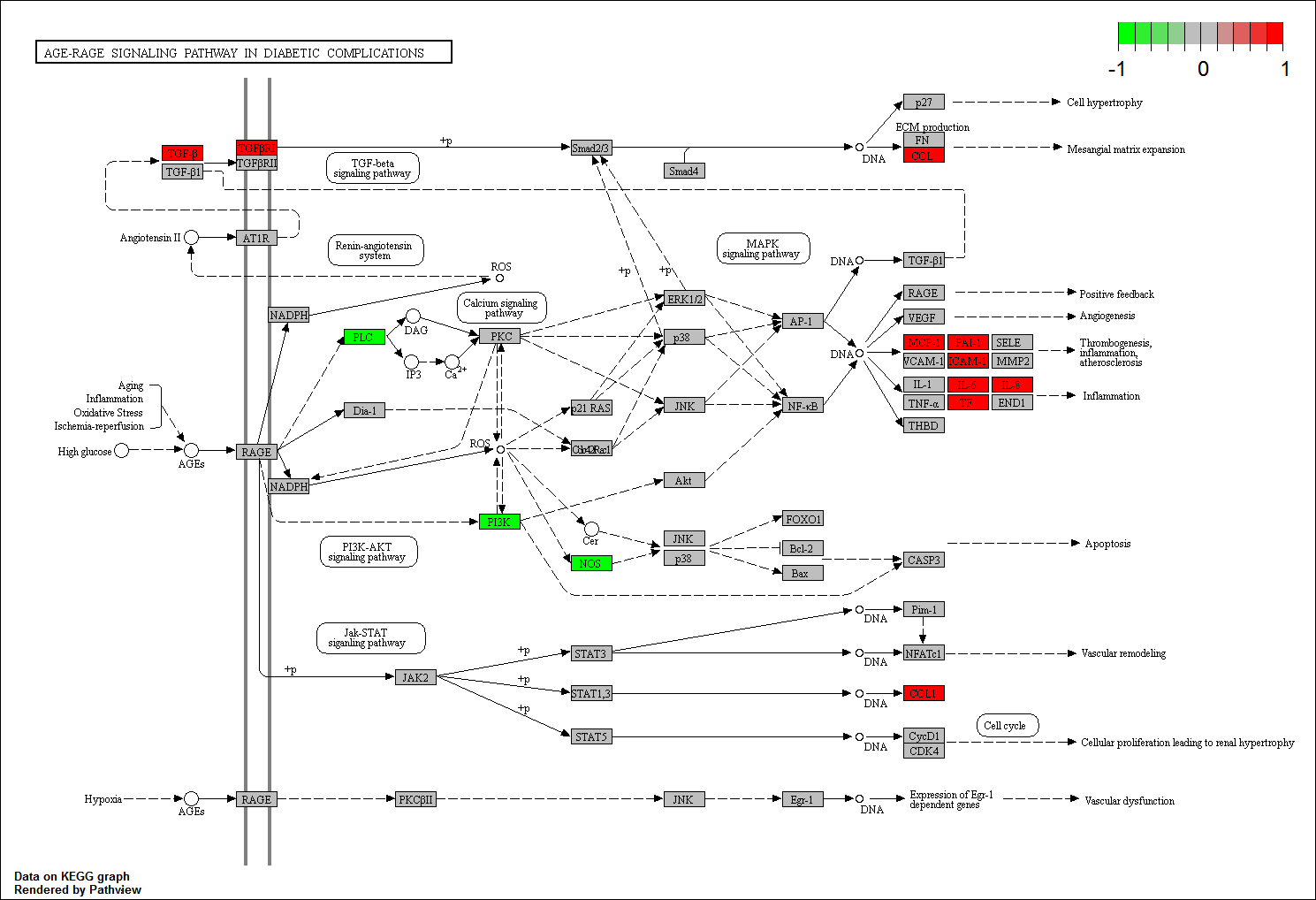

Supplement: DATASET S4 — GO-term analyses for GATA3-expressing and scratched pHAs versus EGFP-expressing and scratched pHAs in 2D cultures. [file Data_Sheet_4.ZIP › GO_term_analyses_GATA3s_vs_GFPs/clusterProfiler/hsa04933.AGE-RAGEsignalingpathwayindiabeticcomplications.png]

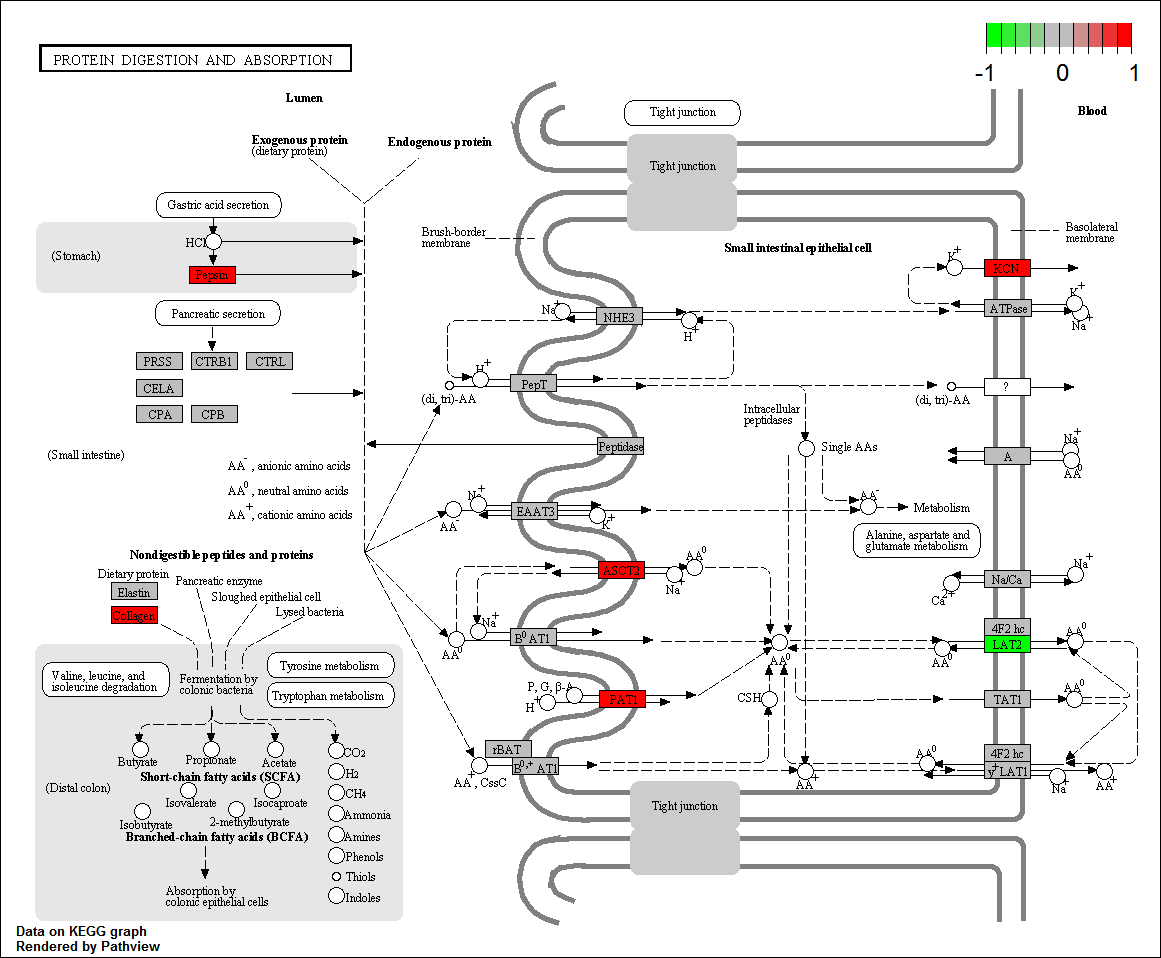

Supplement: DATASET S4 — GO-term analyses for GATA3-expressing and scratched pHAs versus EGFP-expressing and scratched pHAs in 2D cultures. [file Data_Sheet_4.ZIP › GO_term_analyses_GATA3s_vs_GFPs/clusterProfiler/hsa04974.Proteindigestionandabsorption.png]

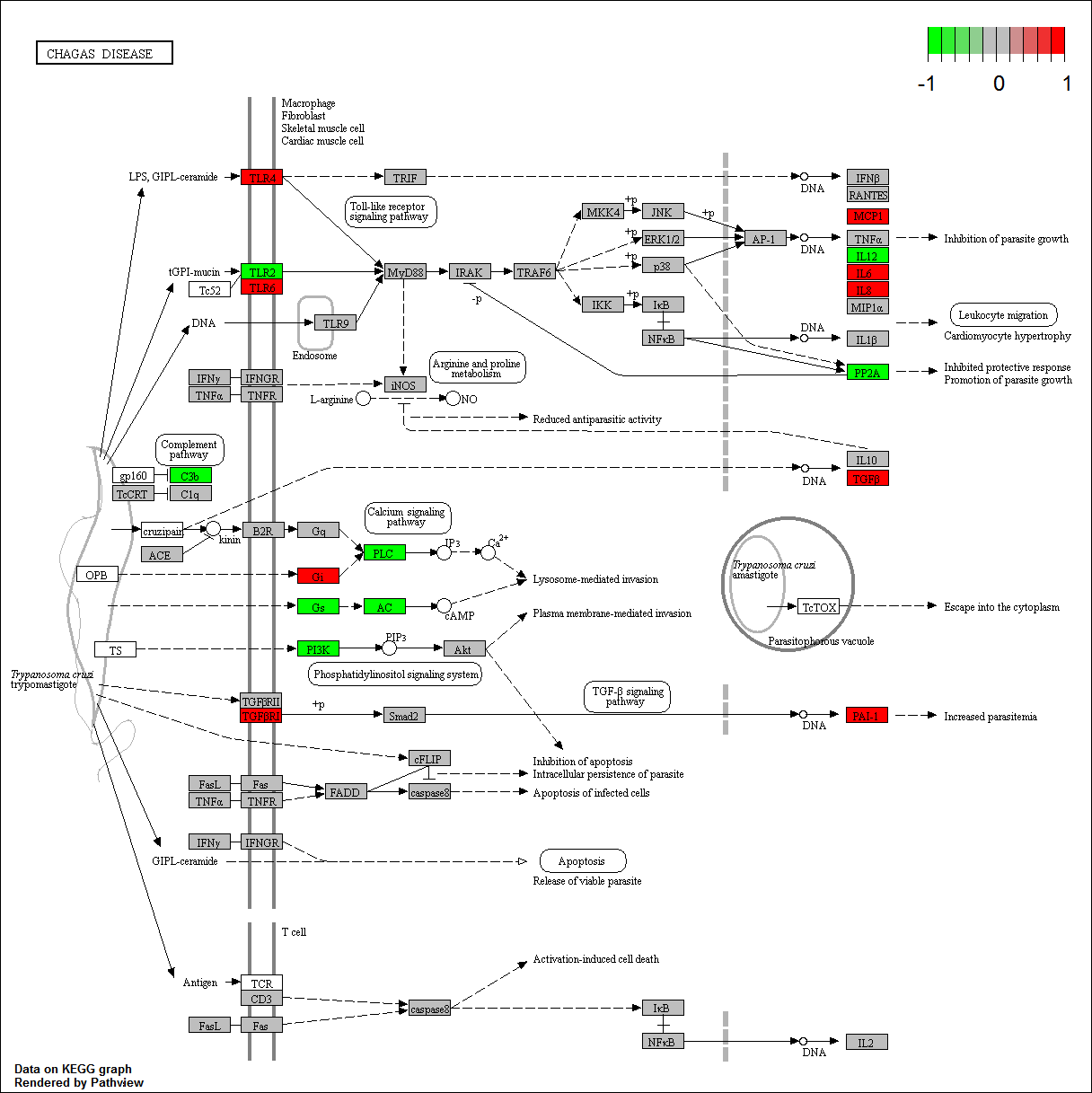

Supplement: DATASET S4 — GO-term analyses for GATA3-expressing and scratched pHAs versus EGFP-expressing and scratched pHAs in 2D cultures. [file Data_Sheet_4.ZIP › GO_term_analyses_GATA3s_vs_GFPs/clusterProfiler/hsa05142.Chagasdisease(Americantrypanosomiasis).png]

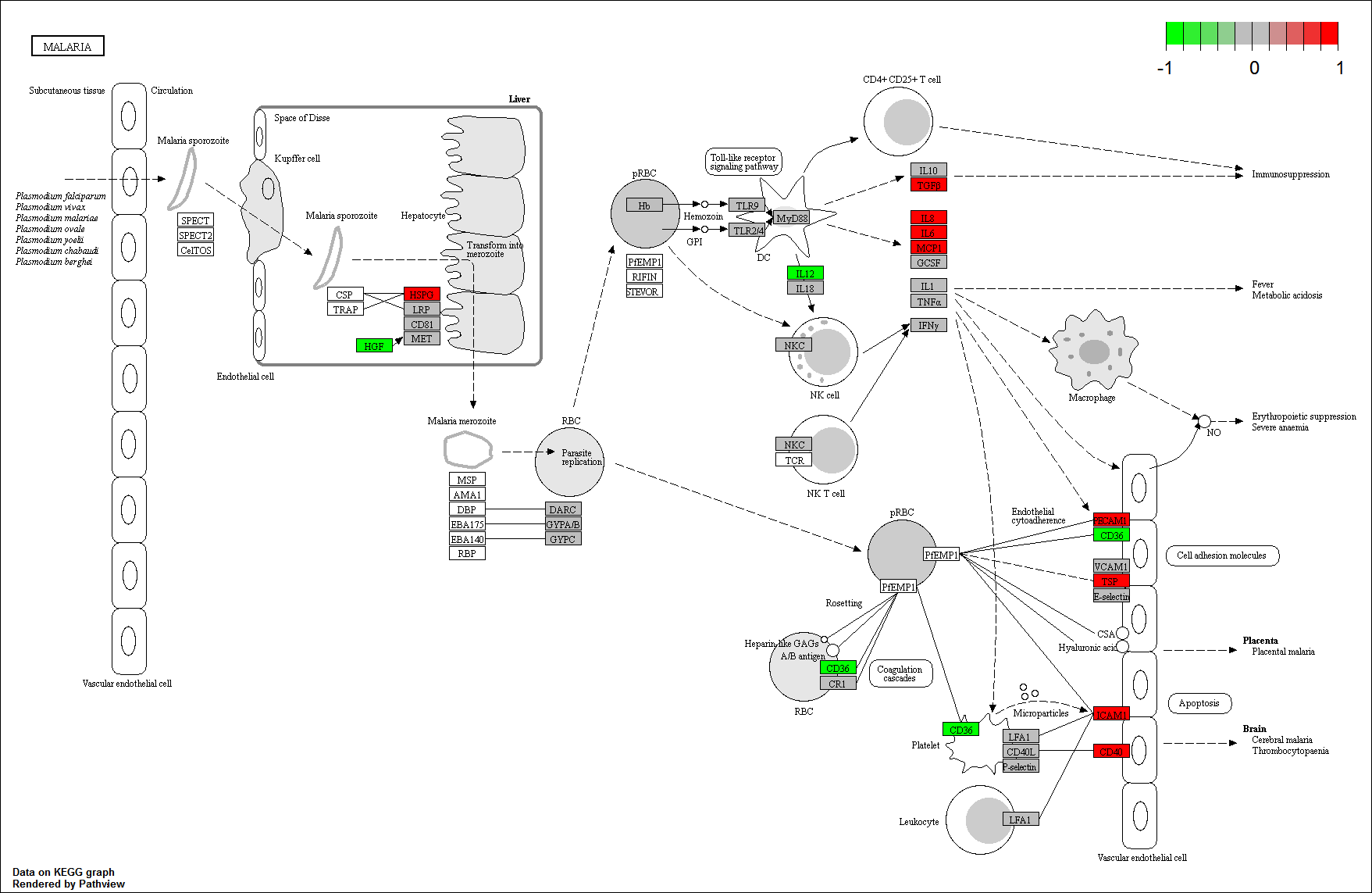

Supplement: DATASET S4 — GO-term analyses for GATA3-expressing and scratched pHAs versus EGFP-expressing and scratched pHAs in 2D cultures. [file Data_Sheet_4.ZIP › GO_term_analyses_GATA3s_vs_GFPs/clusterProfiler/hsa05144.Malaria.png]

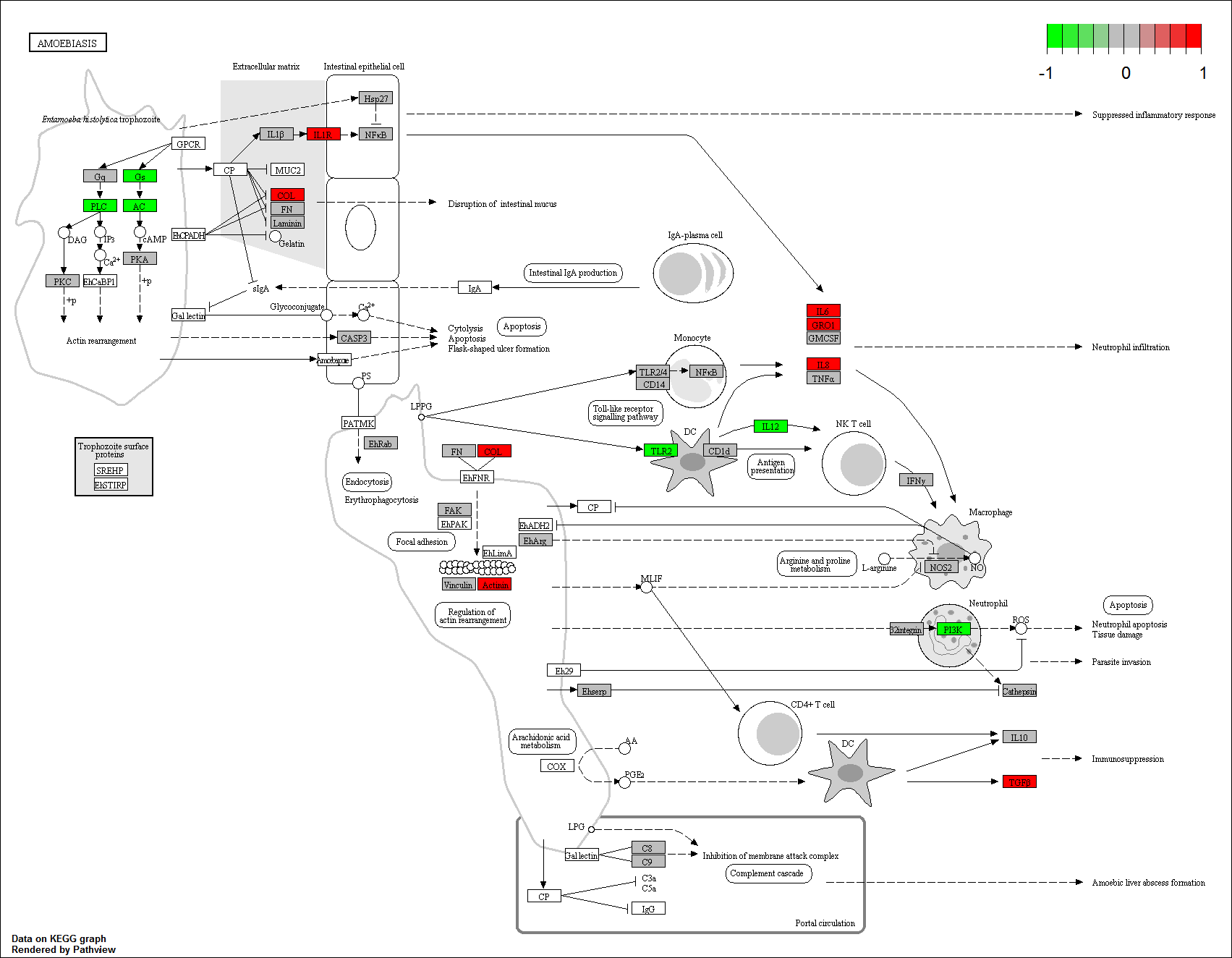

Supplement: DATASET S4 — GO-term analyses for GATA3-expressing and scratched pHAs versus EGFP-expressing and scratched pHAs in 2D cultures. [file Data_Sheet_4.ZIP › GO_term_analyses_GATA3s_vs_GFPs/clusterProfiler/hsa05146.Amoebiasis.png]

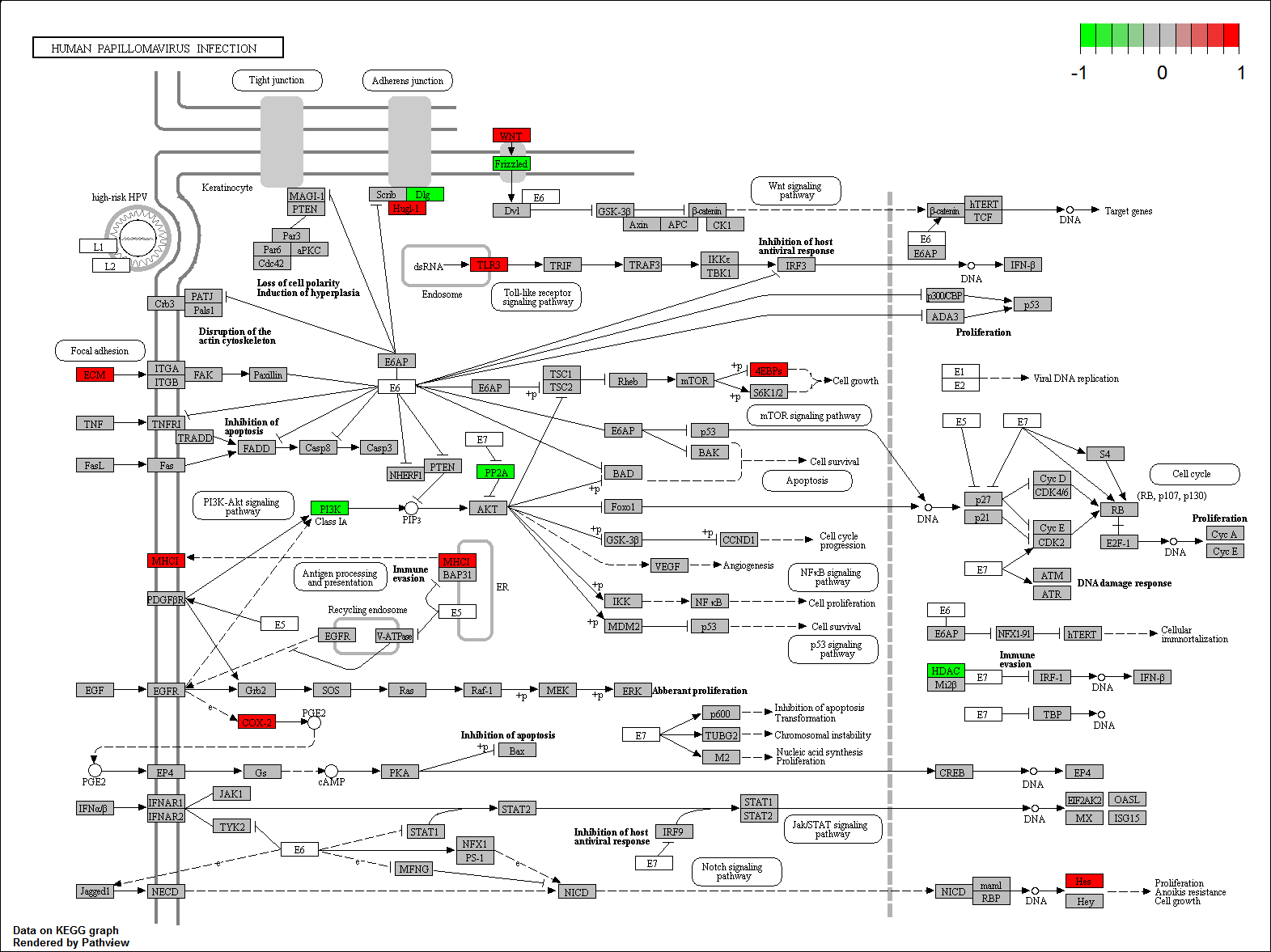

Supplement: DATASET S4 — GO-term analyses for GATA3-expressing and scratched pHAs versus EGFP-expressing and scratched pHAs in 2D cultures. [file Data_Sheet_4.ZIP › GO_term_analyses_GATA3s_vs_GFPs/clusterProfiler/hsa05165.Humanpapillomavirusinfection.png]

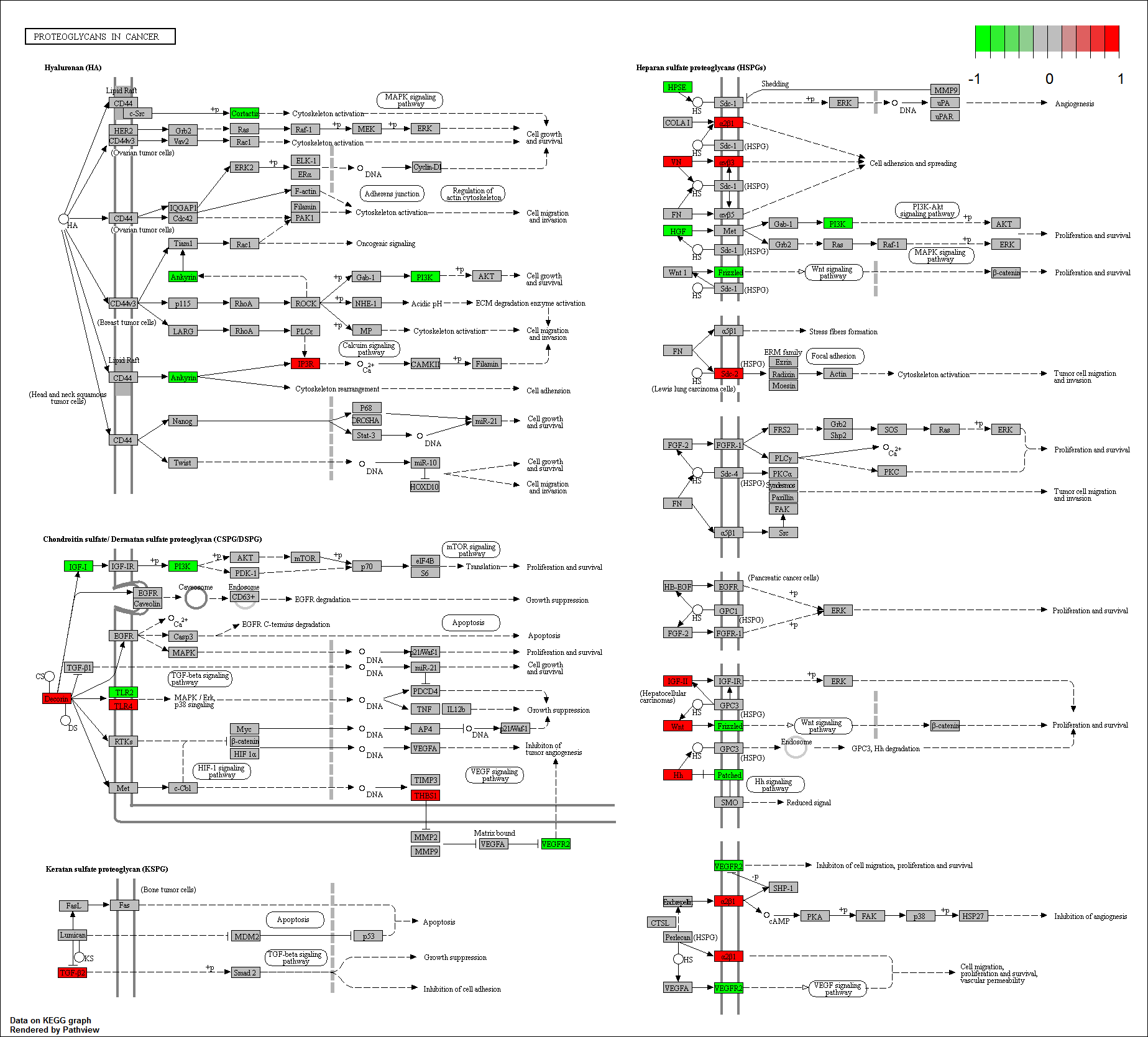

Supplement: DATASET S4 — GO-term analyses for GATA3-expressing and scratched pHAs versus EGFP-expressing and scratched pHAs in 2D cultures. [file Data_Sheet_4.ZIP › GO_term_analyses_GATA3s_vs_GFPs/clusterProfiler/hsa05205.Proteoglycansincancer.png]

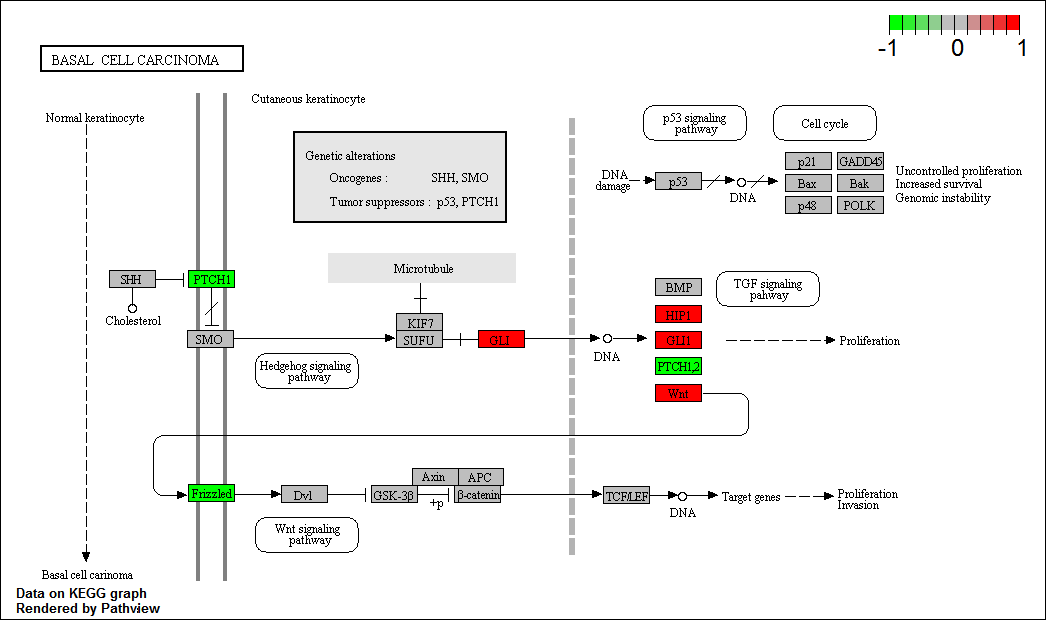

Supplement: DATASET S4 — GO-term analyses for GATA3-expressing and scratched pHAs versus EGFP-expressing and scratched pHAs in 2D cultures. [file Data_Sheet_4.ZIP › GO_term_analyses_GATA3s_vs_GFPs/clusterProfiler/hsa05217.Basalcellcarcinoma.png]

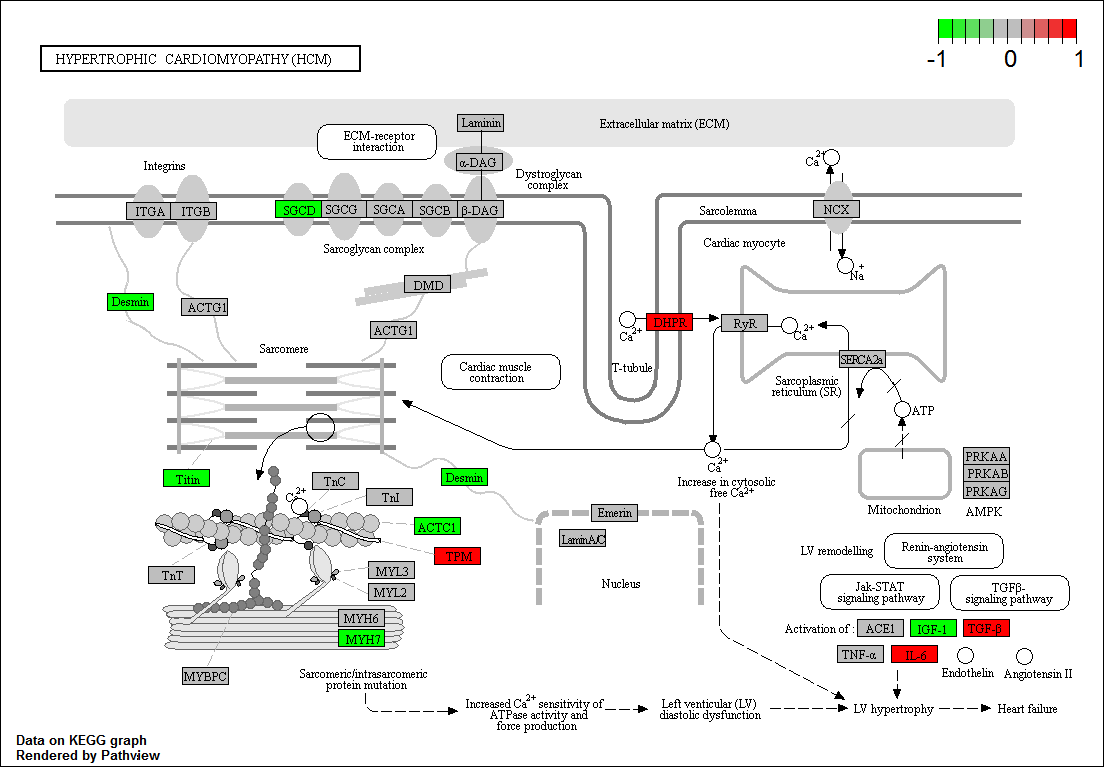

Supplement: DATASET S4 — GO-term analyses for GATA3-expressing and scratched pHAs versus EGFP-expressing and scratched pHAs in 2D cultures. [file Data_Sheet_4.ZIP › GO_term_analyses_GATA3s_vs_GFPs/clusterProfiler/hsa05410.Hypertrophiccardiomyopathy(HCM).png]

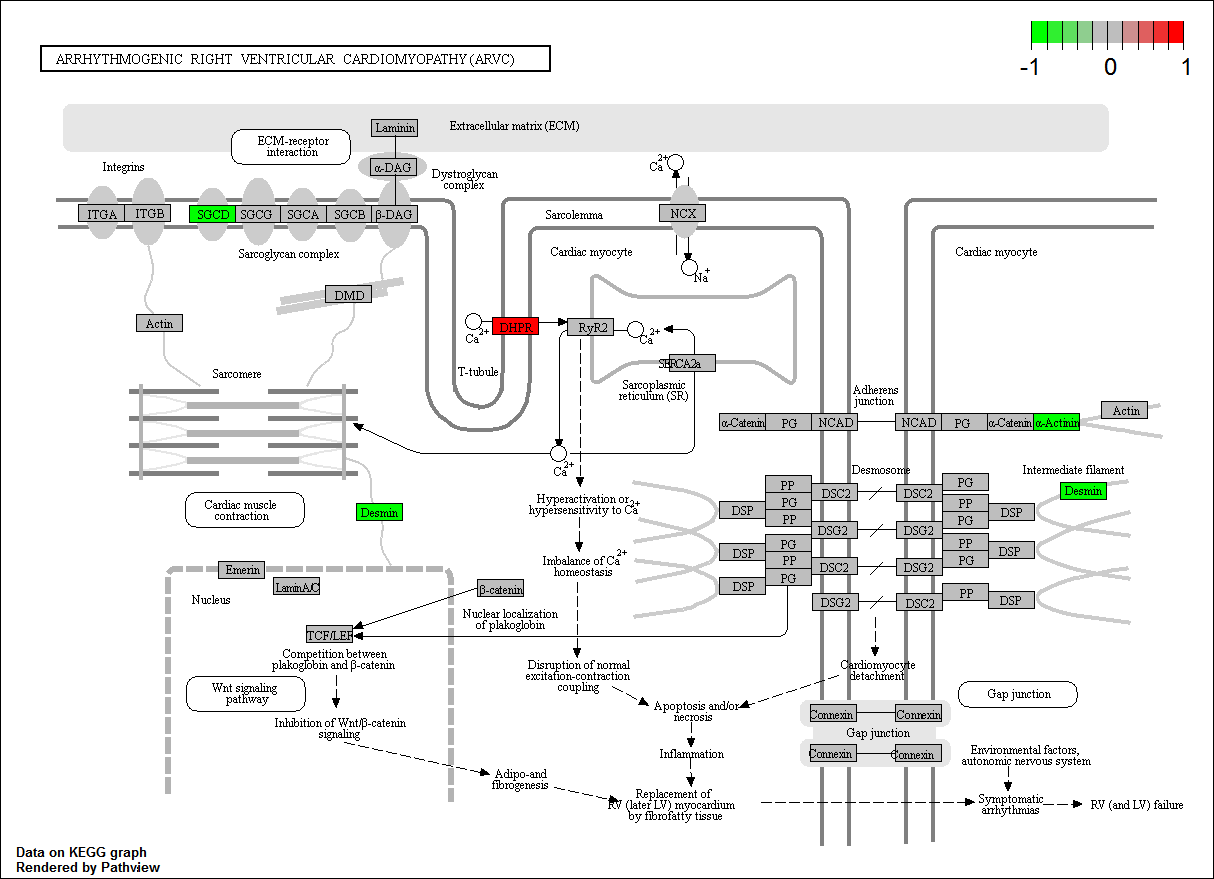

Supplement: DATASET S4 — GO-term analyses for GATA3-expressing and scratched pHAs versus EGFP-expressing and scratched pHAs in 2D cultures. [file Data_Sheet_4.ZIP › GO_term_analyses_GATA3s_vs_GFPs/clusterProfiler/hsa05412.Arrhythmogenicrightventricularcardiomyopathy(ARVC).png]

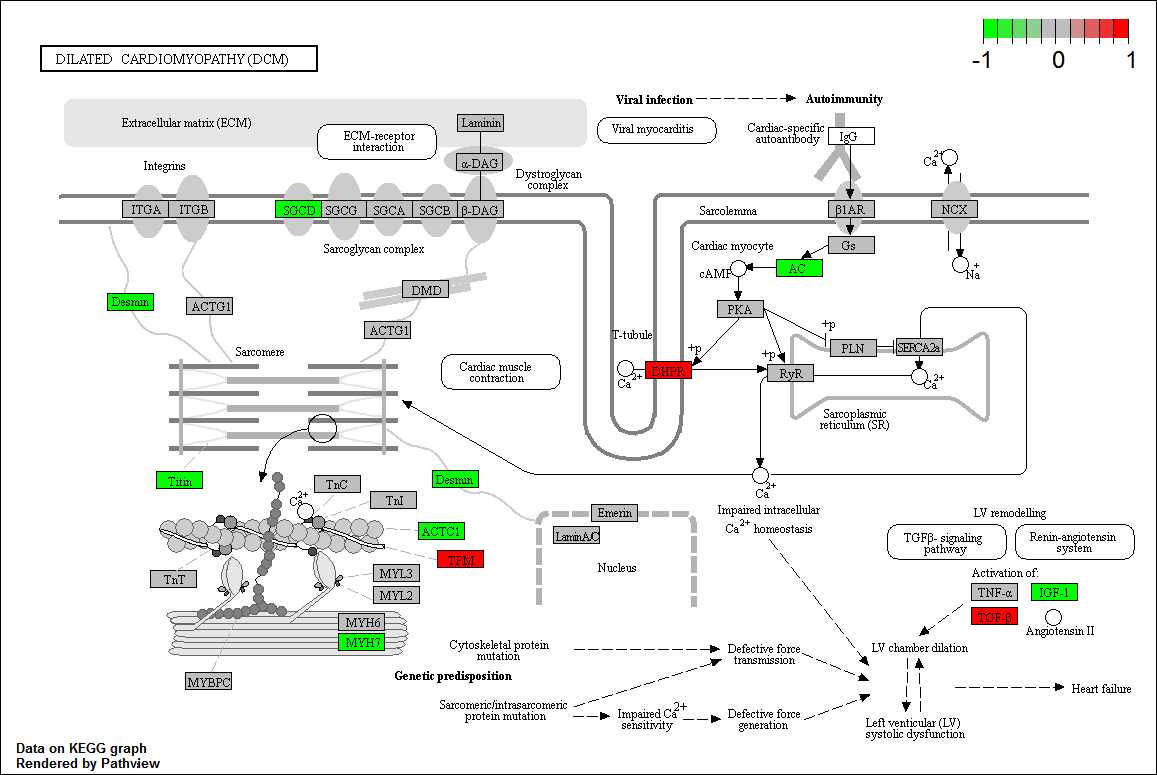

Supplement: DATASET S4 — GO-term analyses for GATA3-expressing and scratched pHAs versus EGFP-expressing and scratched pHAs in 2D cultures. [file Data_Sheet_4.ZIP › GO_term_analyses_GATA3s_vs_GFPs/clusterProfiler/hsa05414.Dilatedcardiomyopathy(DCM).png]

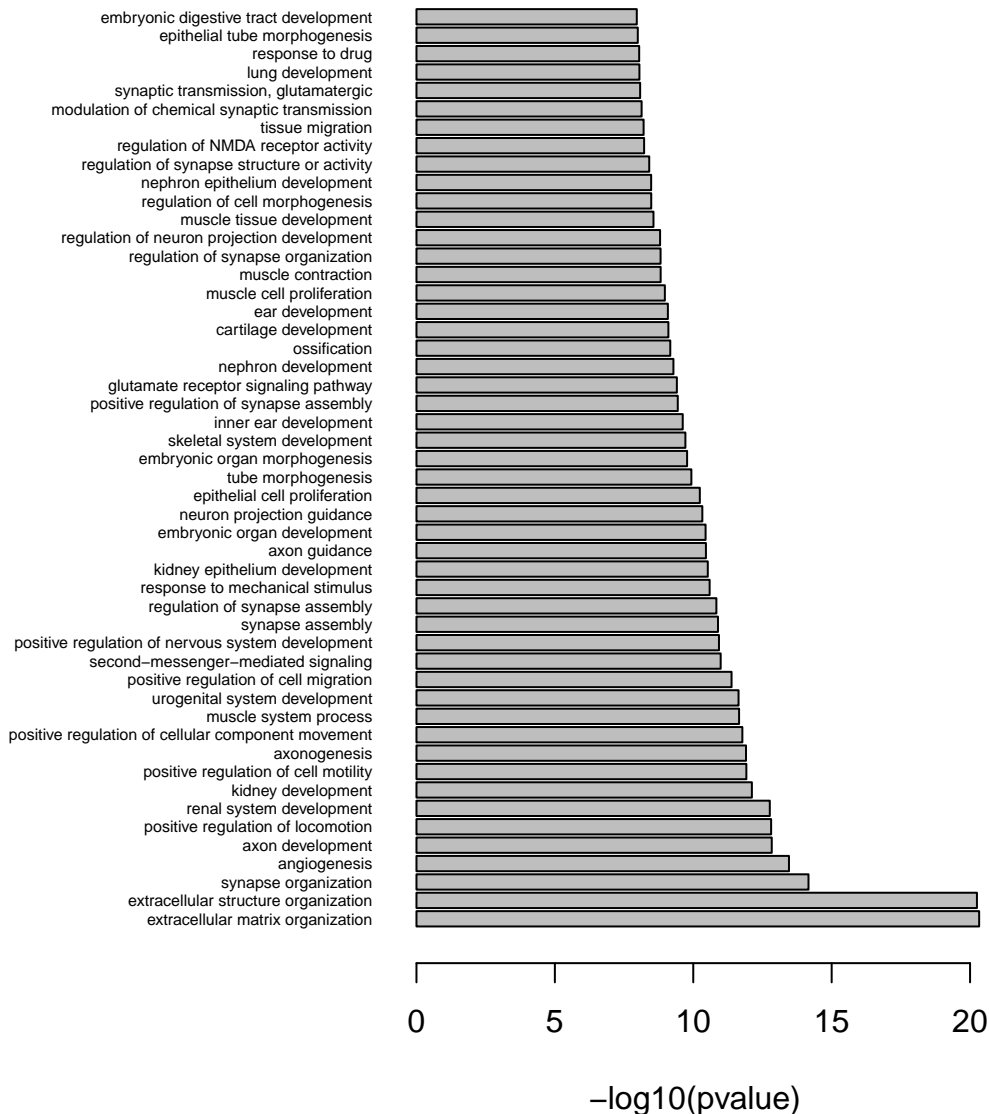

Supplement: DATASET S4 — GO-term analyses for GATA3-expressing and scratched pHAs versus EGFP-expressing and scratched pHAs in 2D cultures. [file Data_Sheet_4.ZIP › GO_term_analyses_GATA3s_vs_GFPs/clusterProfiler/pVal_CP_enrichGO_BP.pdf]

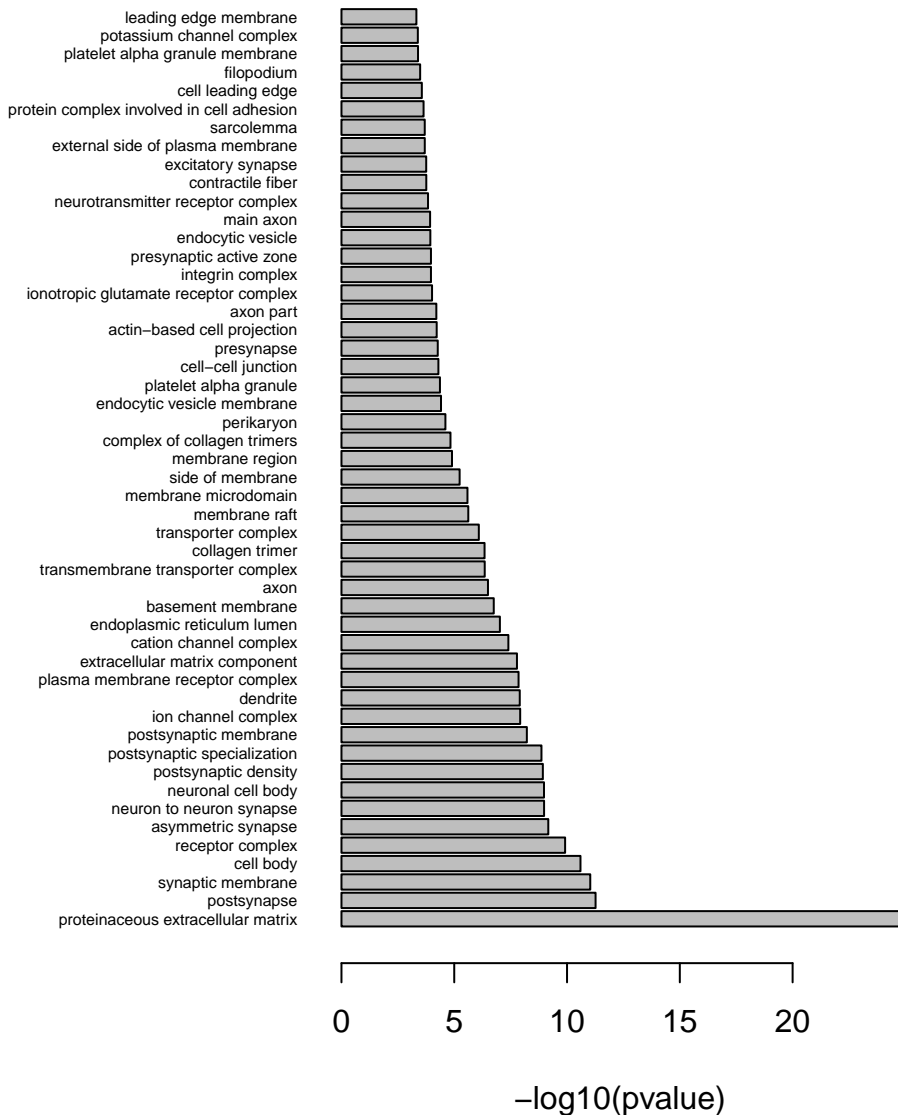

Supplement: DATASET S4 — GO-term analyses for GATA3-expressing and scratched pHAs versus EGFP-expressing and scratched pHAs in 2D cultures. [file Data_Sheet_4.ZIP › GO_term_analyses_GATA3s_vs_GFPs/clusterProfiler/pVal_CP_enrichGO_CC.pdf]

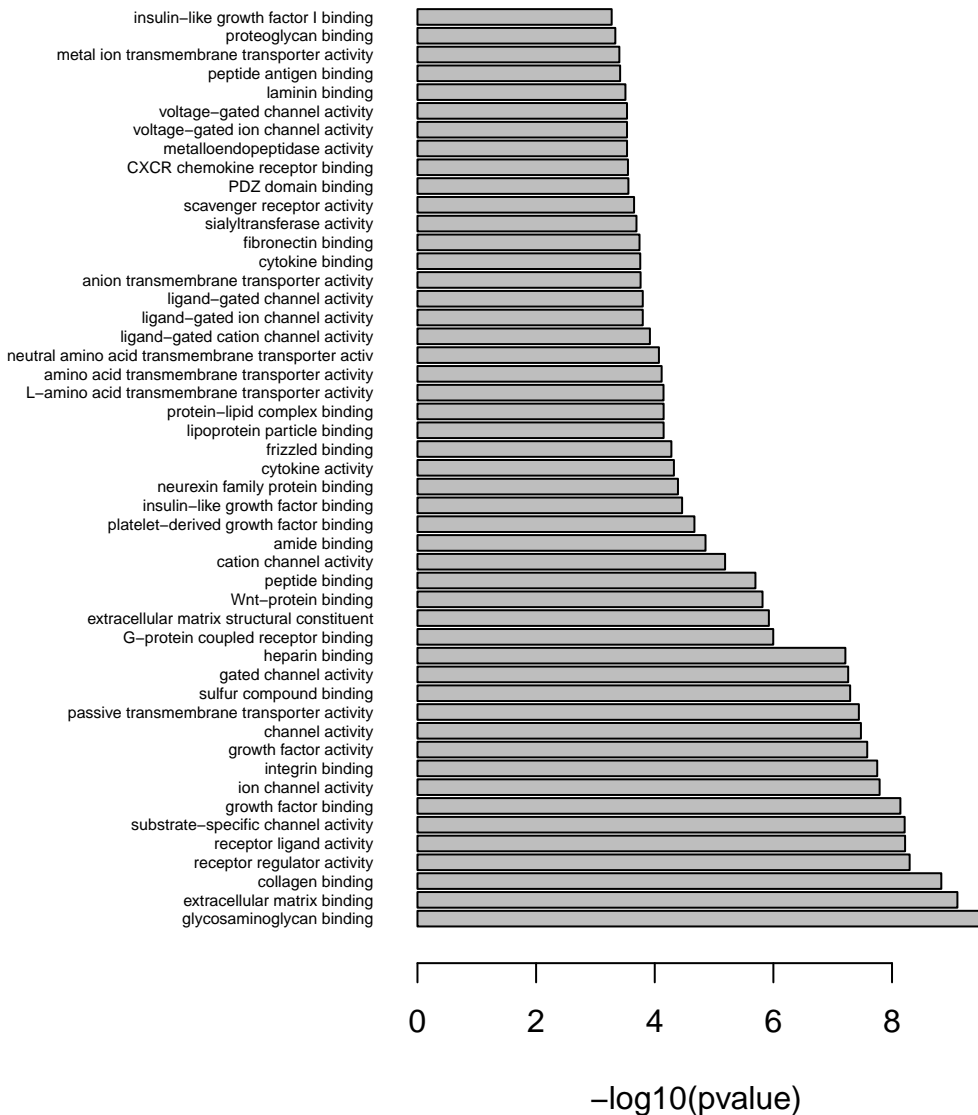

Supplement: DATASET S4 — GO-term analyses for GATA3-expressing and scratched pHAs versus EGFP-expressing and scratched pHAs in 2D cultures. [file Data_Sheet_4.ZIP › GO_term_analyses_GATA3s_vs_GFPs/clusterProfiler/pVal_CP_enrichGO_MF.pdf]

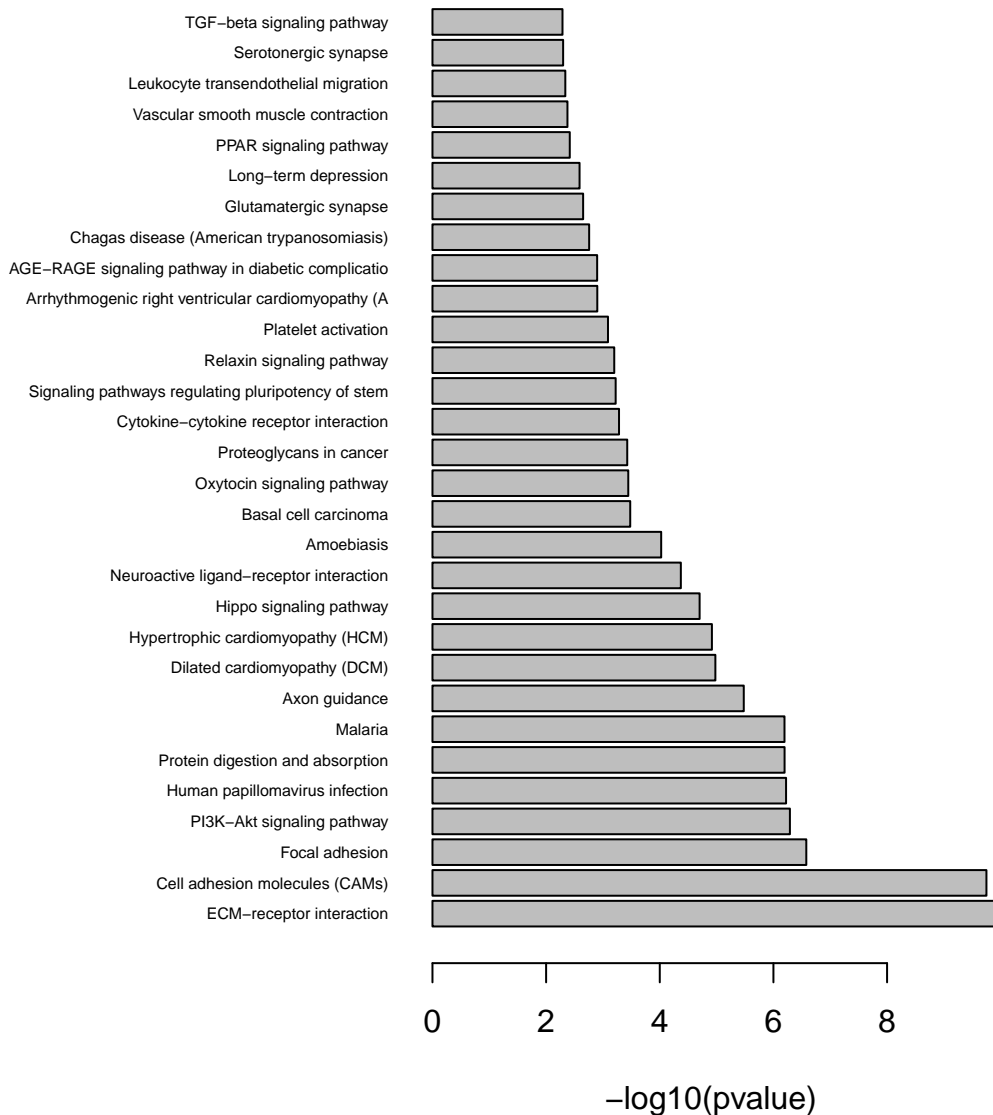

Supplement: DATASET S4 — GO-term analyses for GATA3-expressing and scratched pHAs versus EGFP-expressing and scratched pHAs in 2D cultures. [file Data_Sheet_4.ZIP › GO_term_analyses_GATA3s_vs_GFPs/clusterProfiler/pVal_CP_enrichKEGGS.pdf]

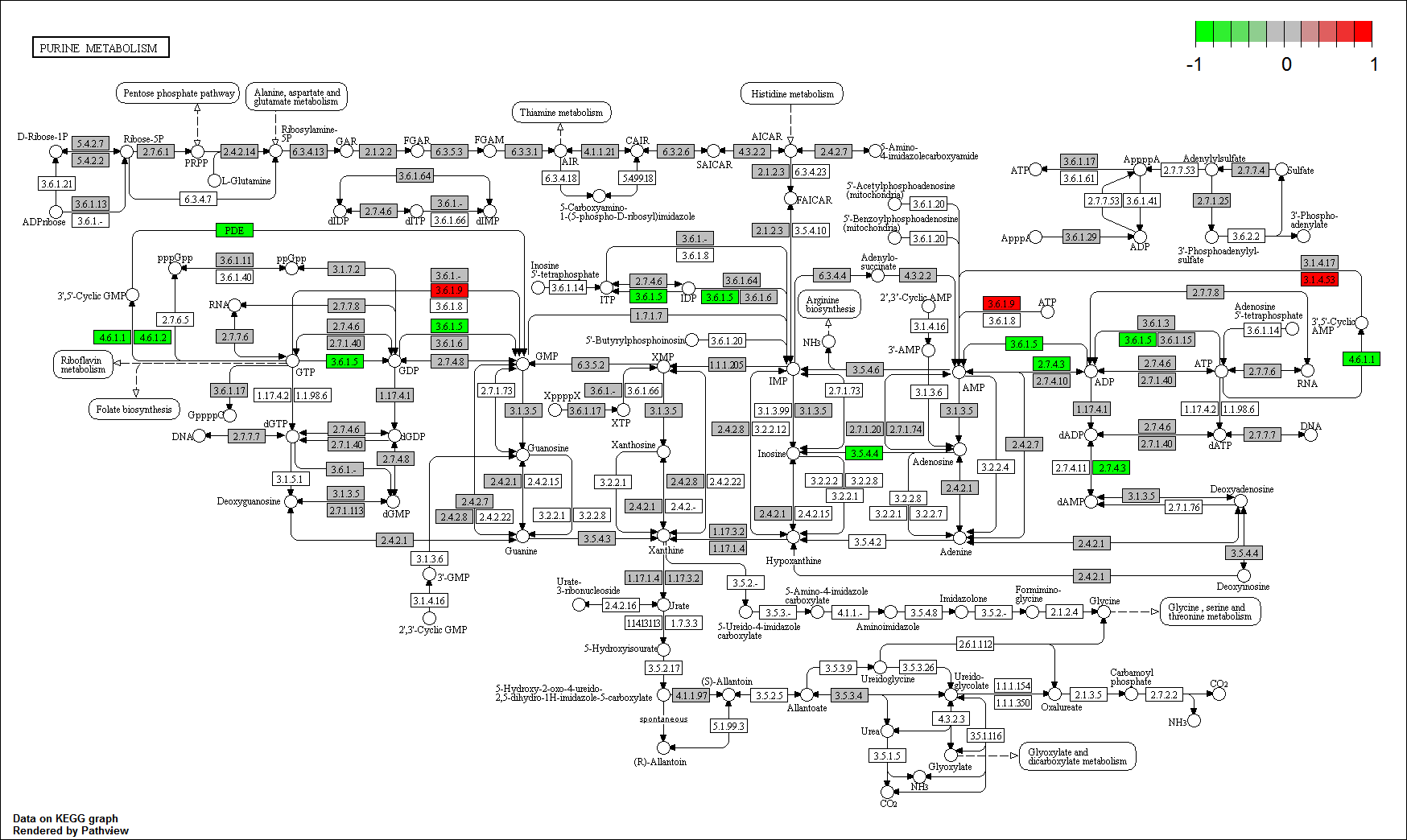

Supplement: DATASET S4 — GO-term analyses for GATA3-expressing and scratched pHAs versus EGFP-expressing and scratched pHAs in 2D cultures. [file Data_Sheet_4.ZIP › GO_term_analyses_GATA3s_vs_GFPs/GOSeq/hsa00230.Purinemetabolism.png]

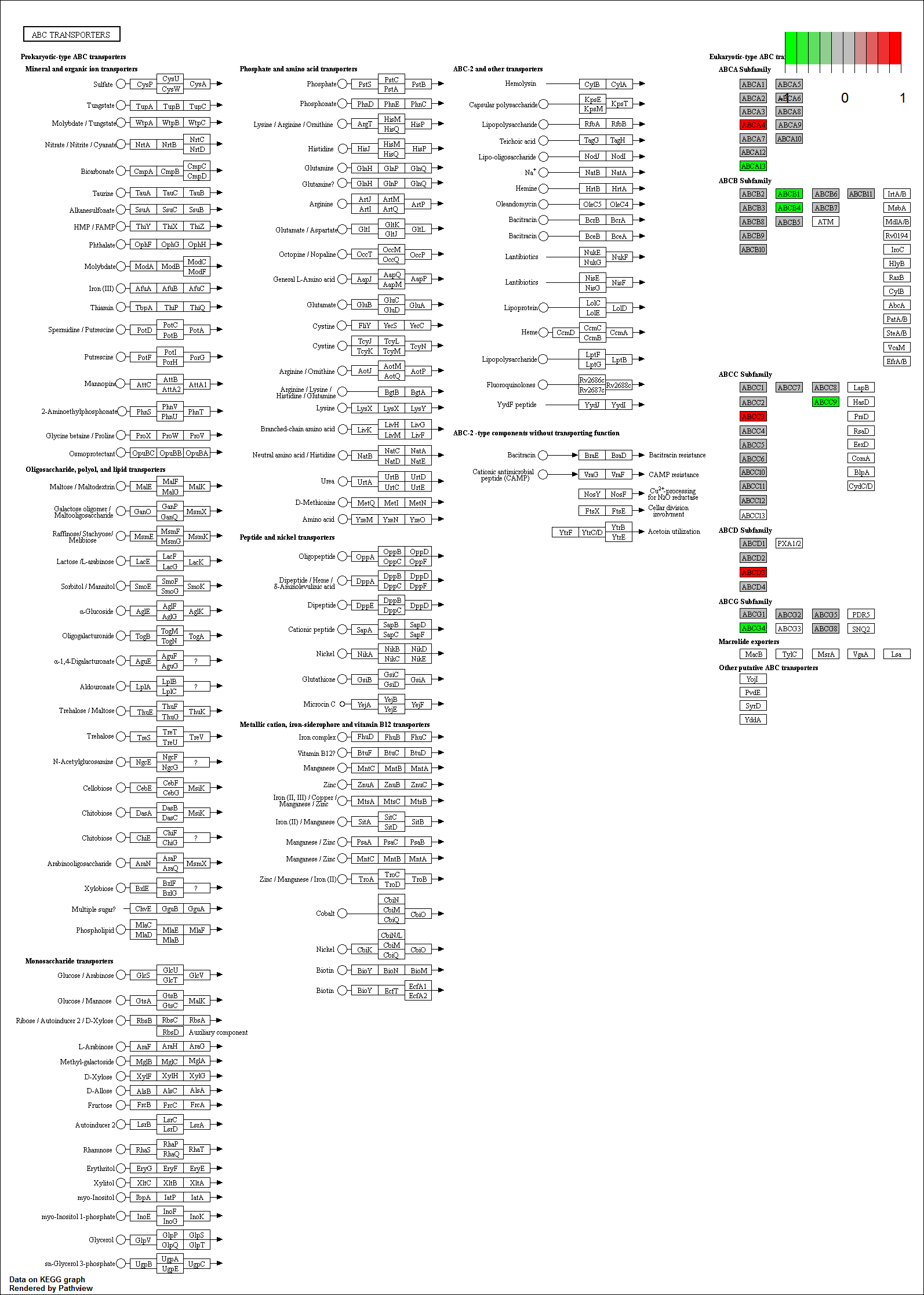

Supplement: DATASET S4 — GO-term analyses for GATA3-expressing and scratched pHAs versus EGFP-expressing and scratched pHAs in 2D cultures. [file Data_Sheet_4.ZIP › GO_term_analyses_GATA3s_vs_GFPs/GOSeq/hsa02010.ABCtransporters.png]

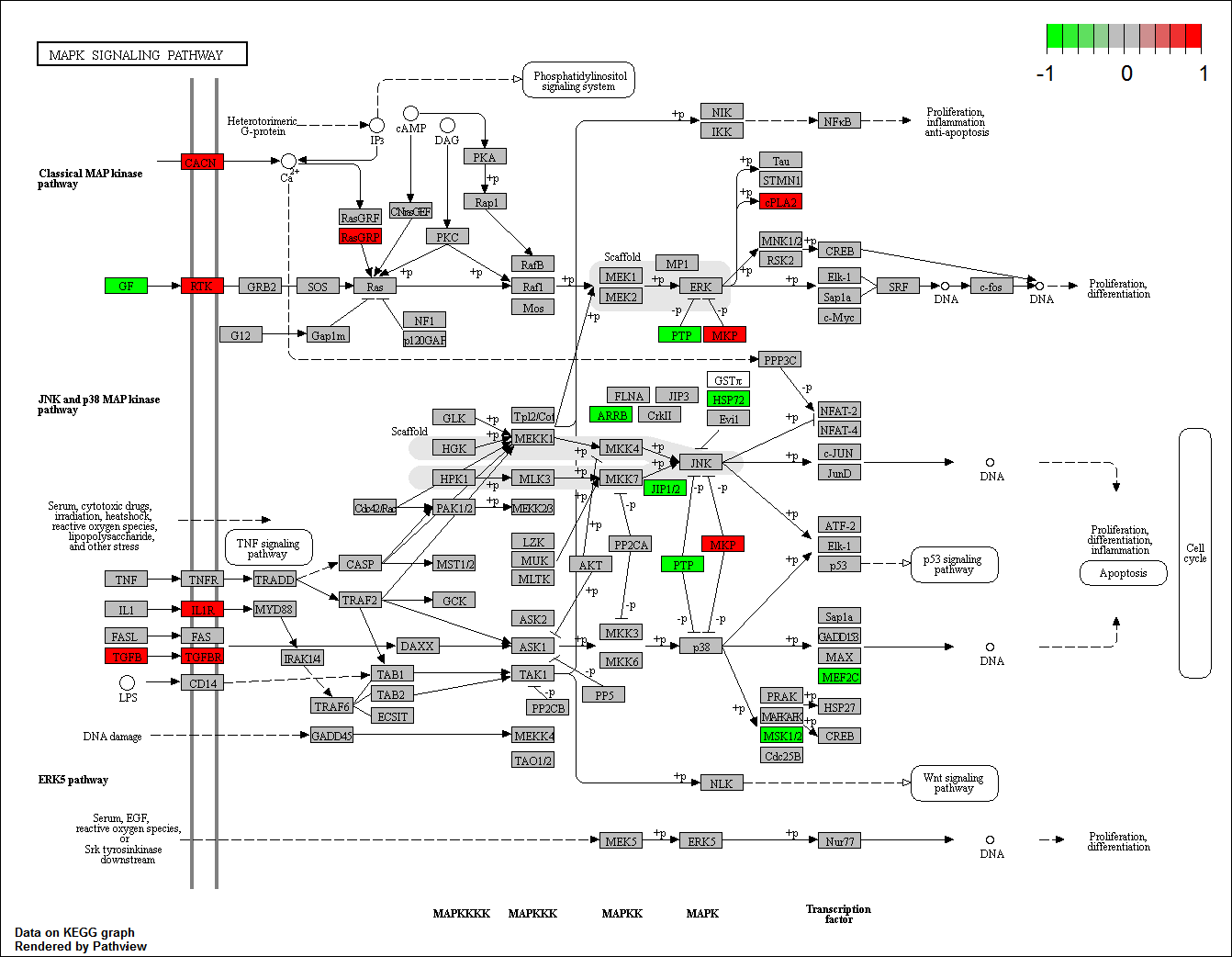

Supplement: DATASET S4 — GO-term analyses for GATA3-expressing and scratched pHAs versus EGFP-expressing and scratched pHAs in 2D cultures. [file Data_Sheet_4.ZIP › GO_term_analyses_GATA3s_vs_GFPs/GOSeq/hsa04010.MAPKsignalingpathway.png]

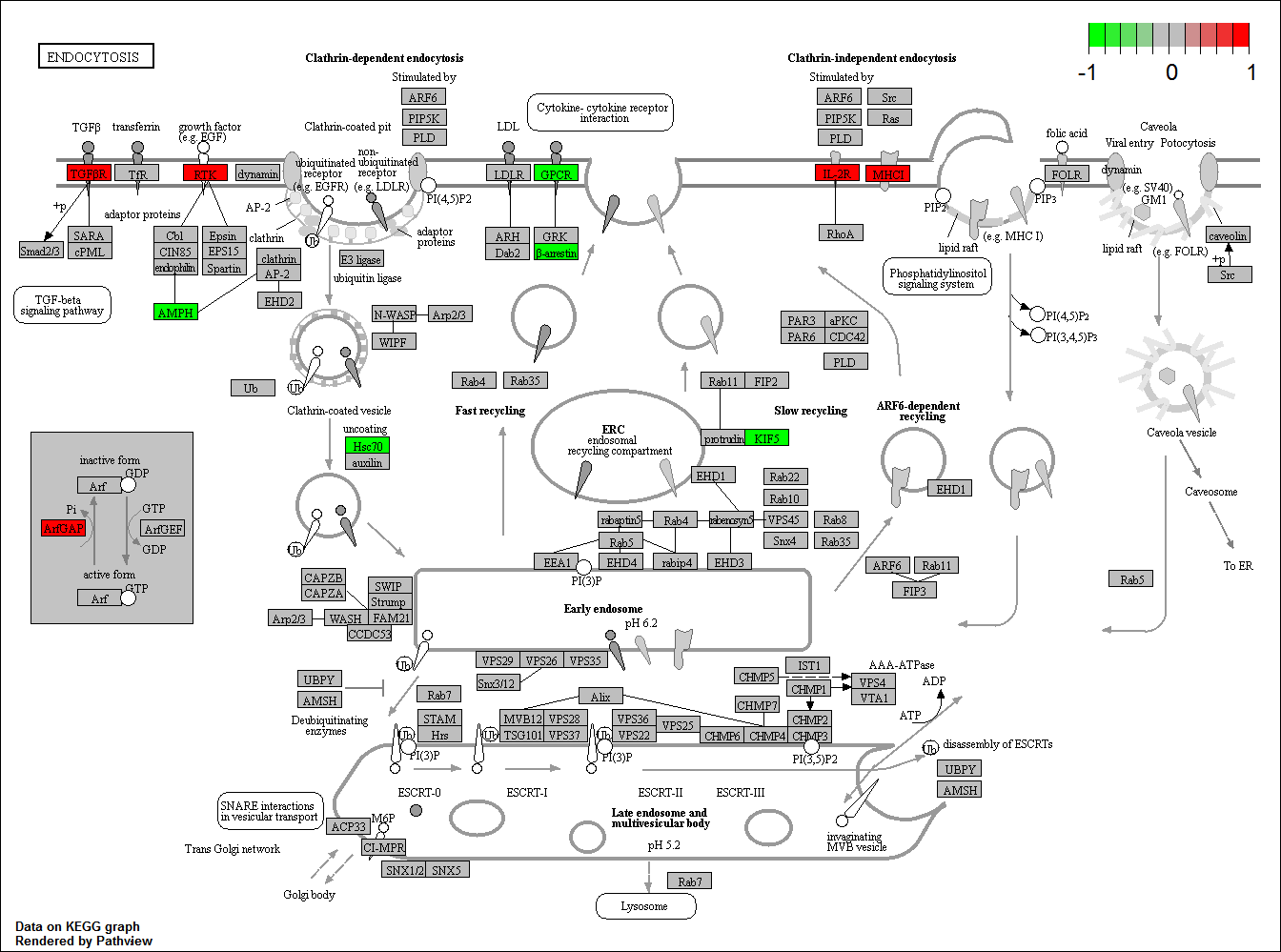

Supplement: DATASET S4 — GO-term analyses for GATA3-expressing and scratched pHAs versus EGFP-expressing and scratched pHAs in 2D cultures. [file Data_Sheet_4.ZIP › GO_term_analyses_GATA3s_vs_GFPs/GOSeq/hsa04144.Endocytosis.png]

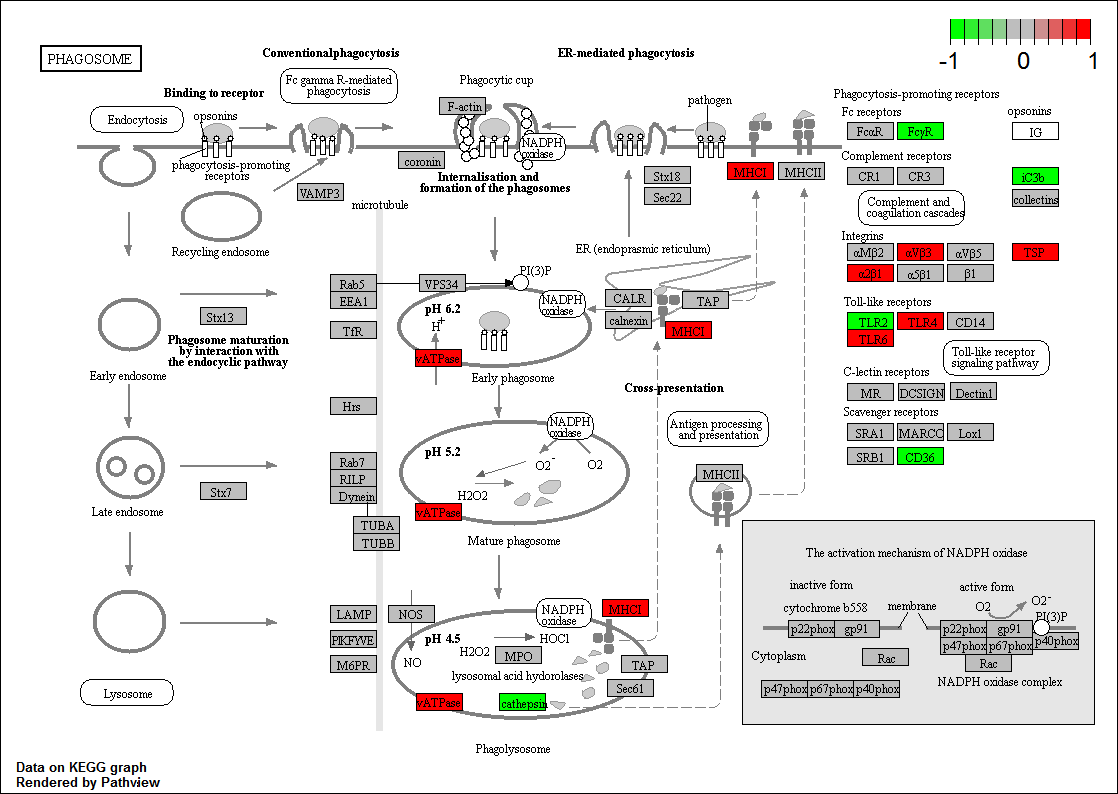

Supplement: DATASET S4 — GO-term analyses for GATA3-expressing and scratched pHAs versus EGFP-expressing and scratched pHAs in 2D cultures. [file Data_Sheet_4.ZIP › GO_term_analyses_GATA3s_vs_GFPs/GOSeq/hsa04145.Phagosome.png]

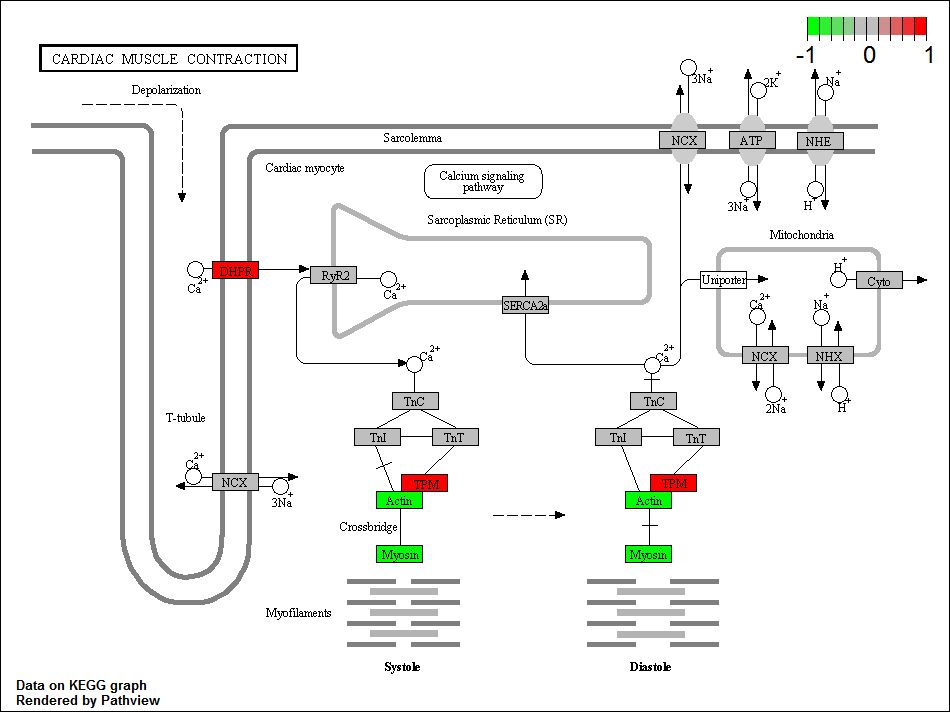

Supplement: DATASET S4 — GO-term analyses for GATA3-expressing and scratched pHAs versus EGFP-expressing and scratched pHAs in 2D cultures. [file Data_Sheet_4.ZIP › GO_term_analyses_GATA3s_vs_GFPs/GOSeq/hsa04260.Cardiacmusclecontraction.png]

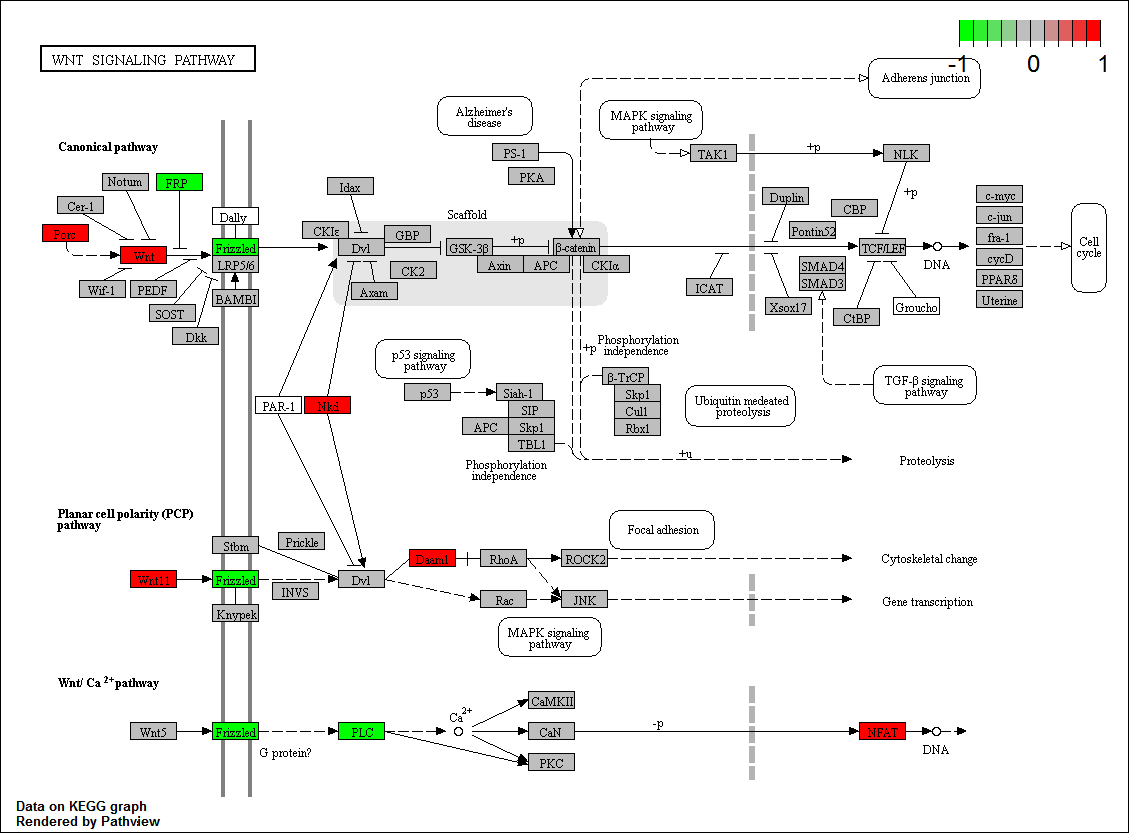

Supplement: DATASET S4 — GO-term analyses for GATA3-expressing and scratched pHAs versus EGFP-expressing and scratched pHAs in 2D cultures. [file Data_Sheet_4.ZIP › GO_term_analyses_GATA3s_vs_GFPs/GOSeq/hsa04310.Wntsignalingpathway.png]

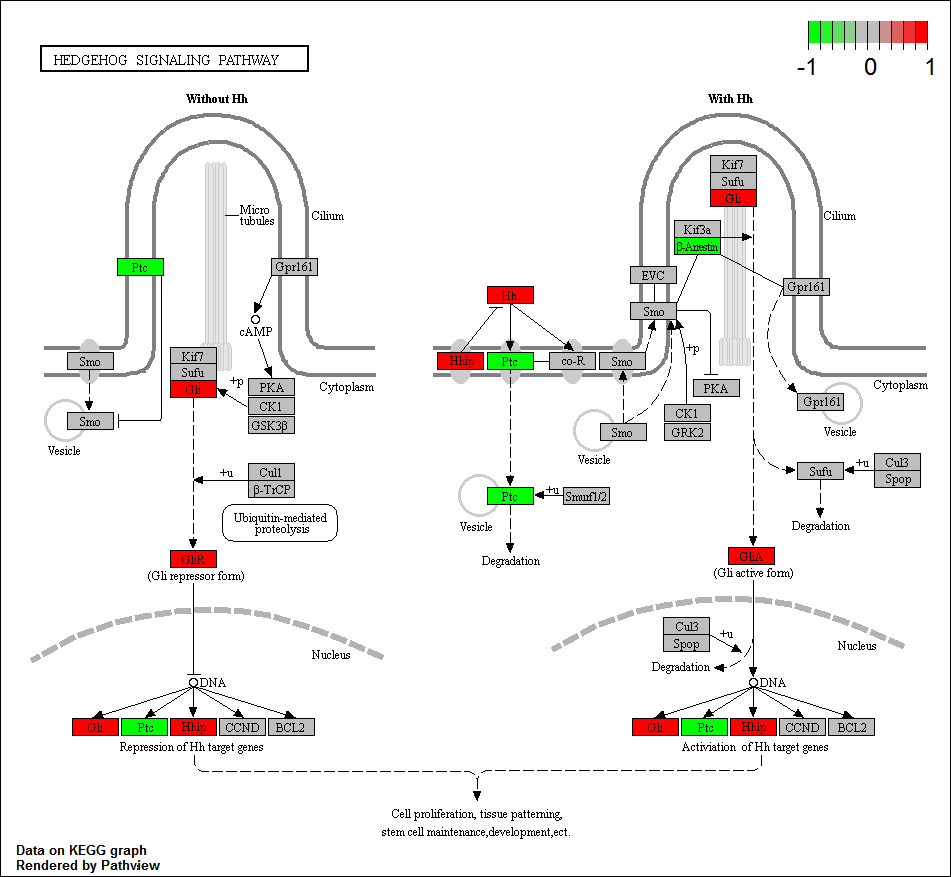

Supplement: DATASET S4 — GO-term analyses for GATA3-expressing and scratched pHAs versus EGFP-expressing and scratched pHAs in 2D cultures. [file Data_Sheet_4.ZIP › GO_term_analyses_GATA3s_vs_GFPs/GOSeq/hsa04340.Hedgehogsignalingpathway.png]

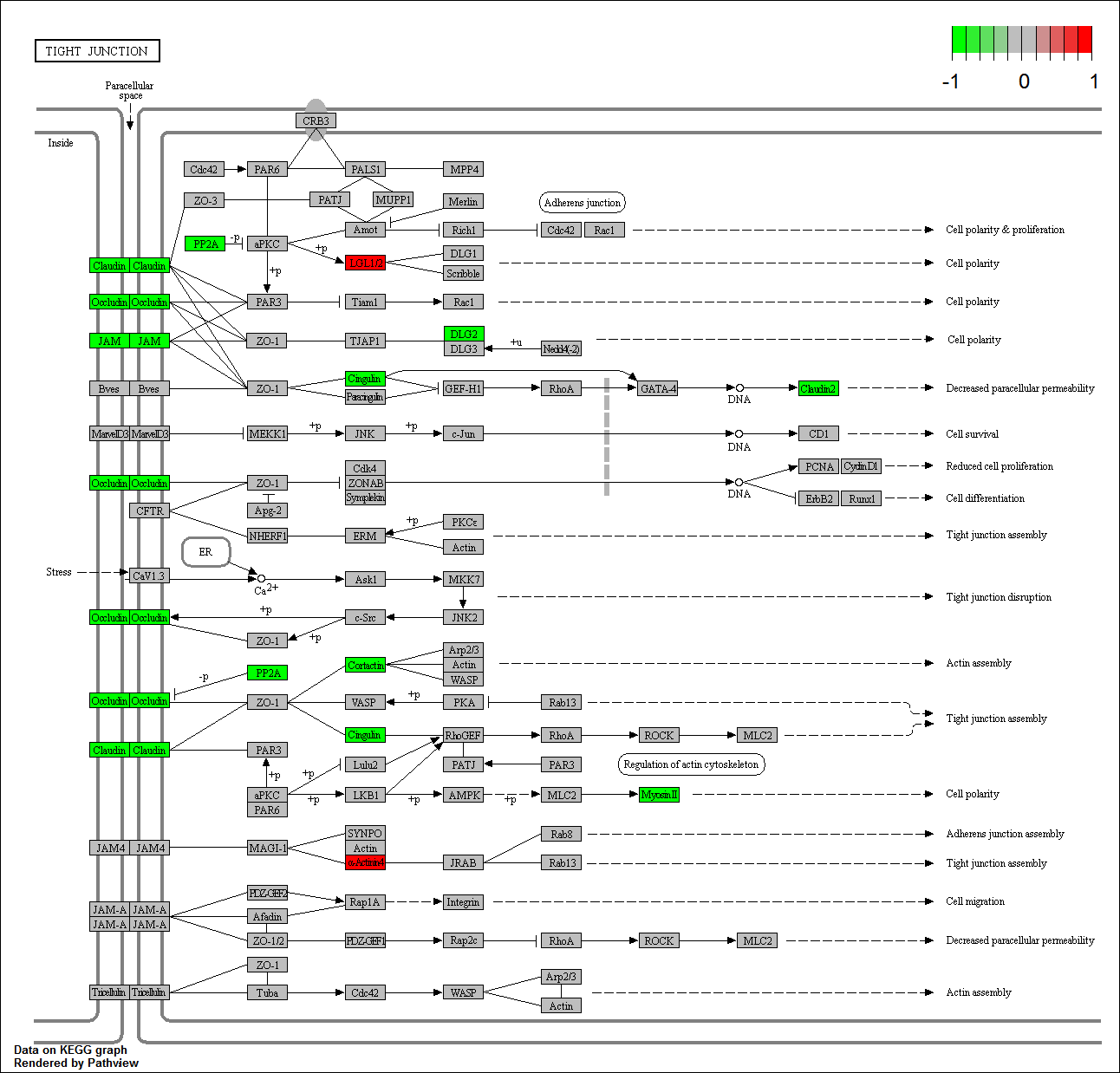

Supplement: DATASET S4 — GO-term analyses for GATA3-expressing and scratched pHAs versus EGFP-expressing and scratched pHAs in 2D cultures. [file Data_Sheet_4.ZIP › GO_term_analyses_GATA3s_vs_GFPs/GOSeq/hsa04530.Tightjunction.png]

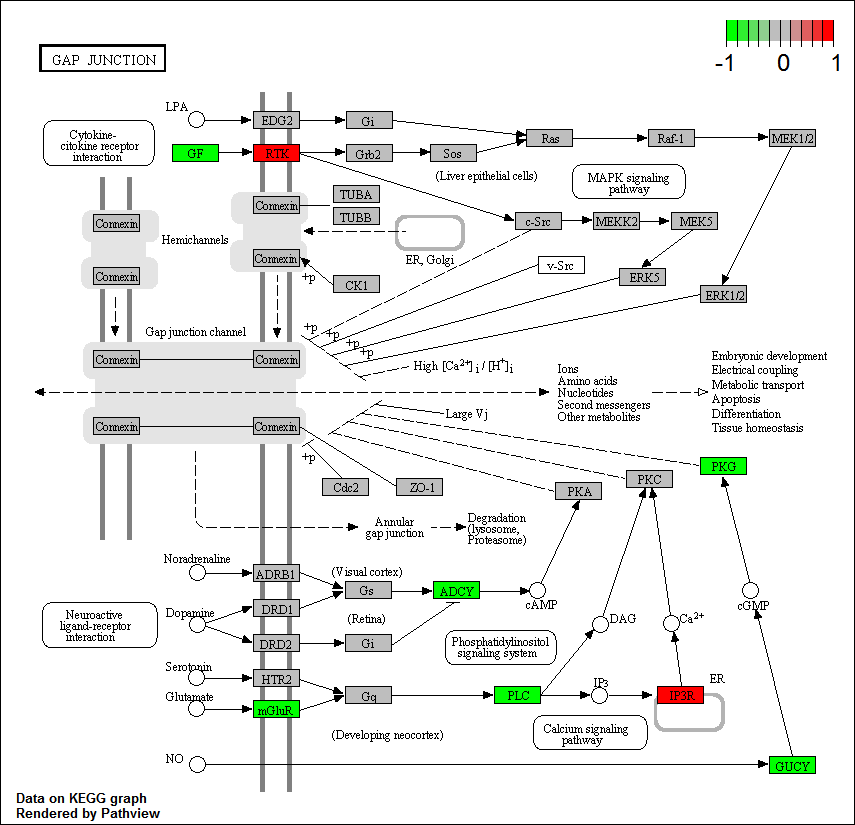

Supplement: DATASET S4 — GO-term analyses for GATA3-expressing and scratched pHAs versus EGFP-expressing and scratched pHAs in 2D cultures. [file Data_Sheet_4.ZIP › GO_term_analyses_GATA3s_vs_GFPs/GOSeq/hsa04540.Gapjunction.png]

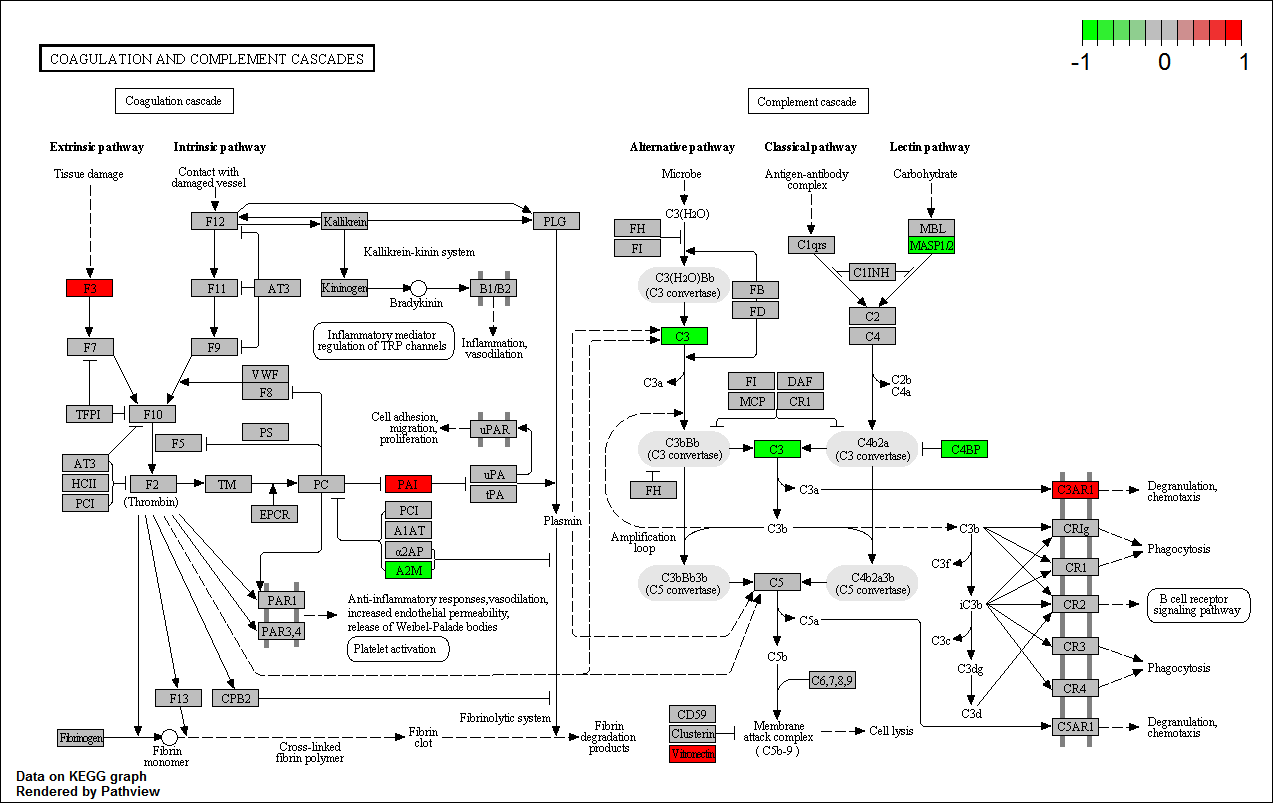

Supplement: DATASET S4 — GO-term analyses for GATA3-expressing and scratched pHAs versus EGFP-expressing and scratched pHAs in 2D cultures. [file Data_Sheet_4.ZIP › GO_term_analyses_GATA3s_vs_GFPs/GOSeq/hsa04610.Complementandcoagulationcascades.png]

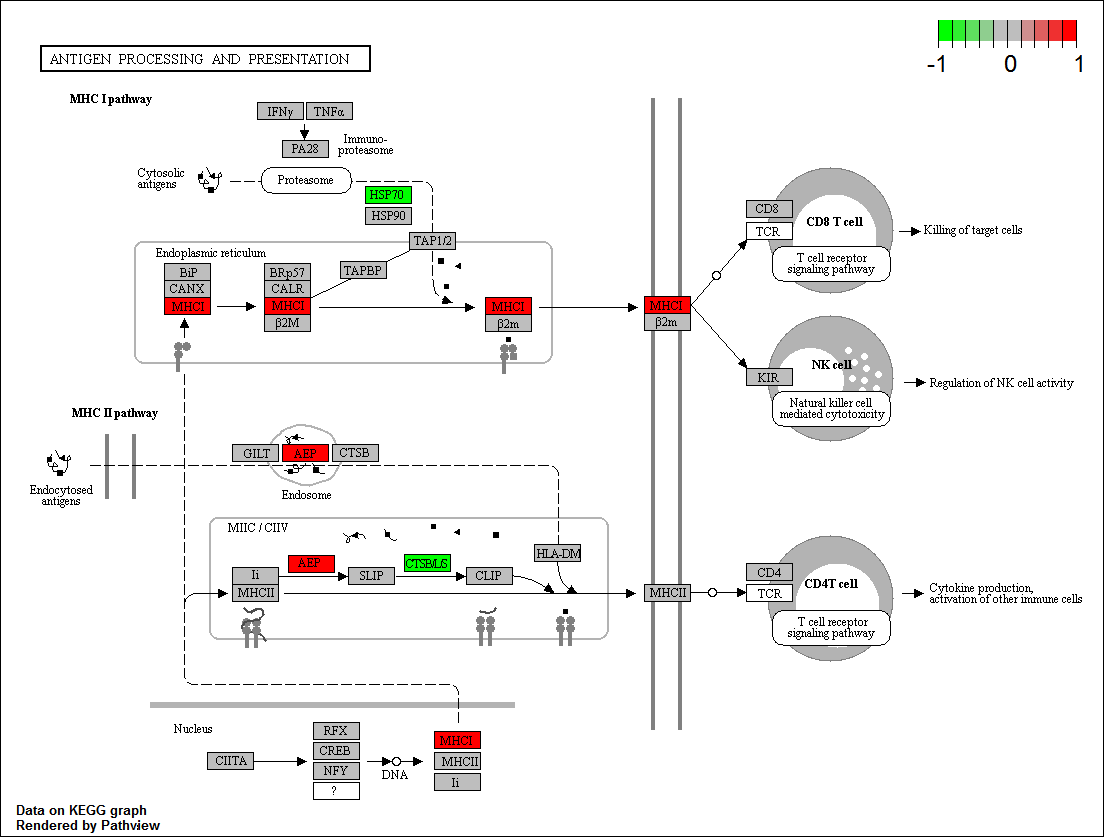

Supplement: DATASET S4 — GO-term analyses for GATA3-expressing and scratched pHAs versus EGFP-expressing and scratched pHAs in 2D cultures. [file Data_Sheet_4.ZIP › GO_term_analyses_GATA3s_vs_GFPs/GOSeq/hsa04612.Antigenprocessingandpresentation.png]

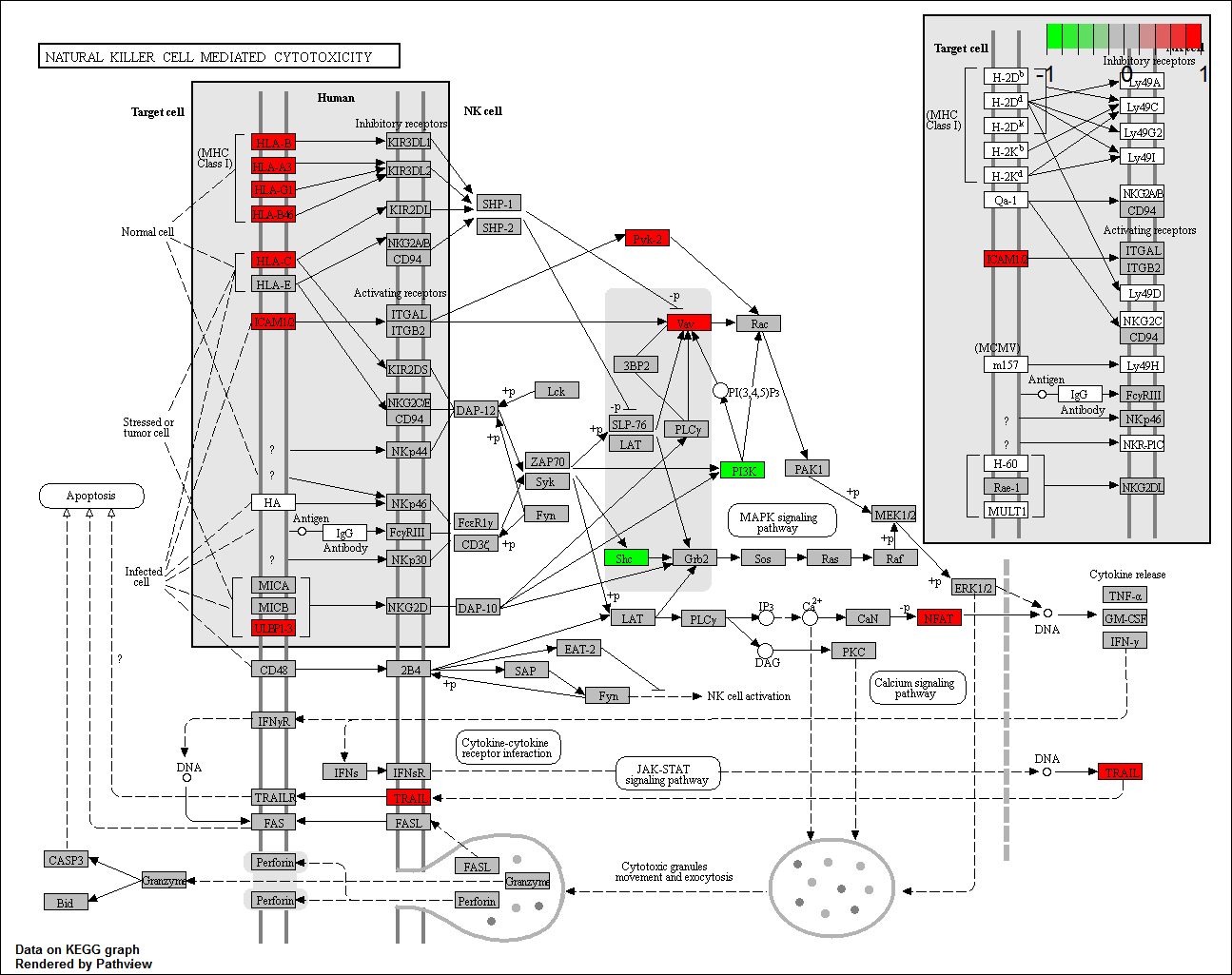

Supplement: DATASET S4 — GO-term analyses for GATA3-expressing and scratched pHAs versus EGFP-expressing and scratched pHAs in 2D cultures. [file Data_Sheet_4.ZIP › GO_term_analyses_GATA3s_vs_GFPs/GOSeq/hsa04650.Naturalkillercellmediatedcytotoxicity.png]

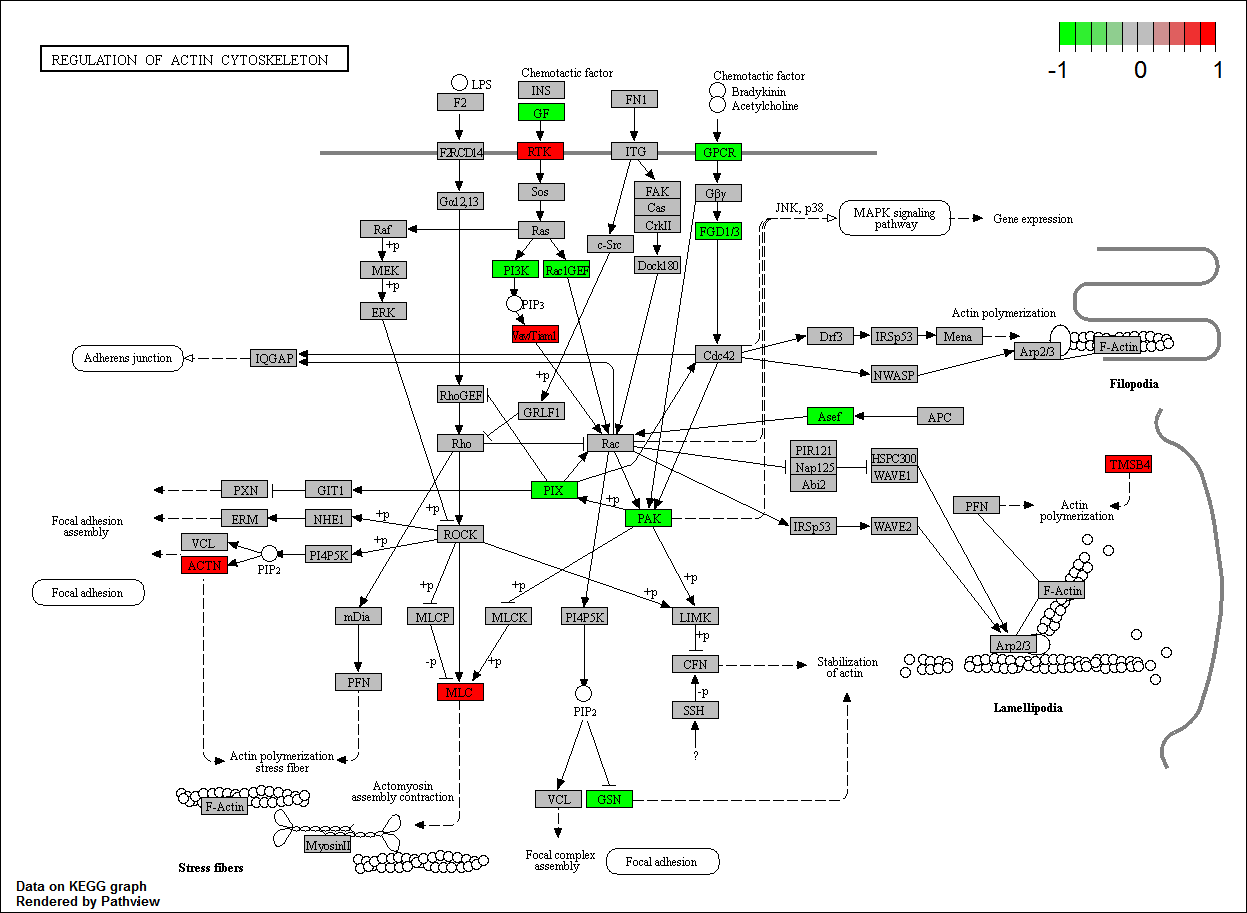

Supplement: DATASET S4 — GO-term analyses for GATA3-expressing and scratched pHAs versus EGFP-expressing and scratched pHAs in 2D cultures. [file Data_Sheet_4.ZIP › GO_term_analyses_GATA3s_vs_GFPs/GOSeq/hsa04810.Regulationofactincytoskeleton.png]

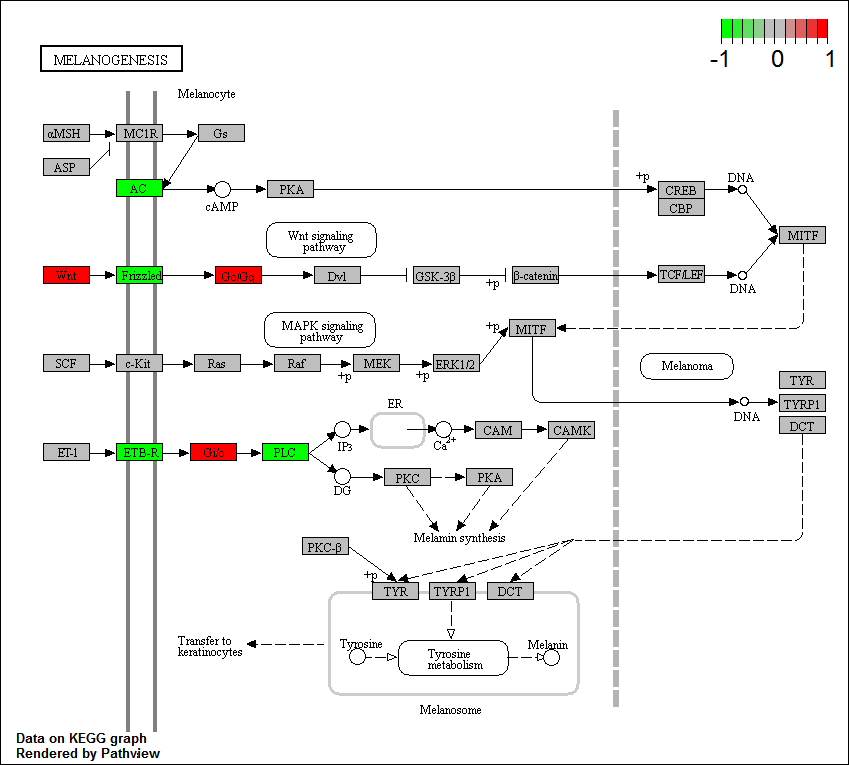

Supplement: DATASET S4 — GO-term analyses for GATA3-expressing and scratched pHAs versus EGFP-expressing and scratched pHAs in 2D cultures. [file Data_Sheet_4.ZIP › GO_term_analyses_GATA3s_vs_GFPs/GOSeq/hsa04916.Melanogenesis.png]

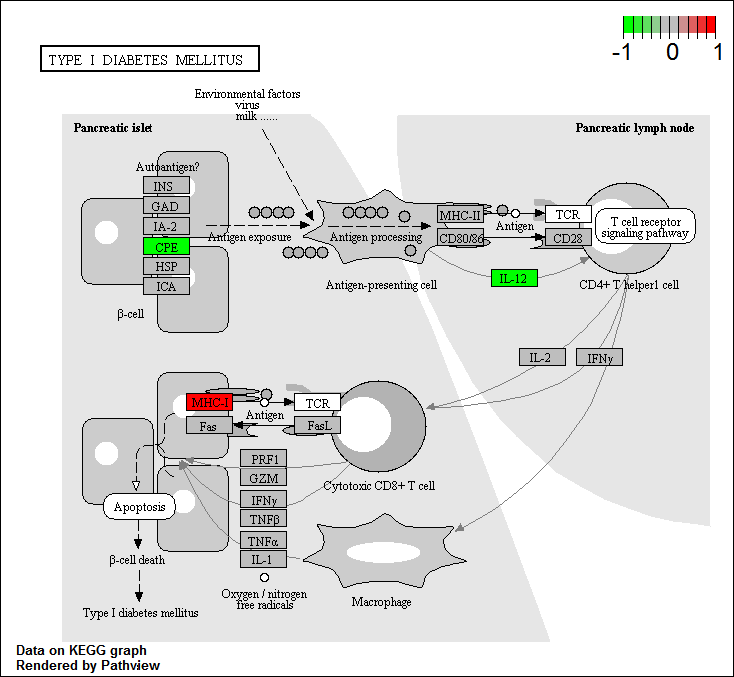

Supplement: DATASET S4 — GO-term analyses for GATA3-expressing and scratched pHAs versus EGFP-expressing and scratched pHAs in 2D cultures. [file Data_Sheet_4.ZIP › GO_term_analyses_GATA3s_vs_GFPs/GOSeq/hsa04940.TypeIdiabetesmellitus.png]

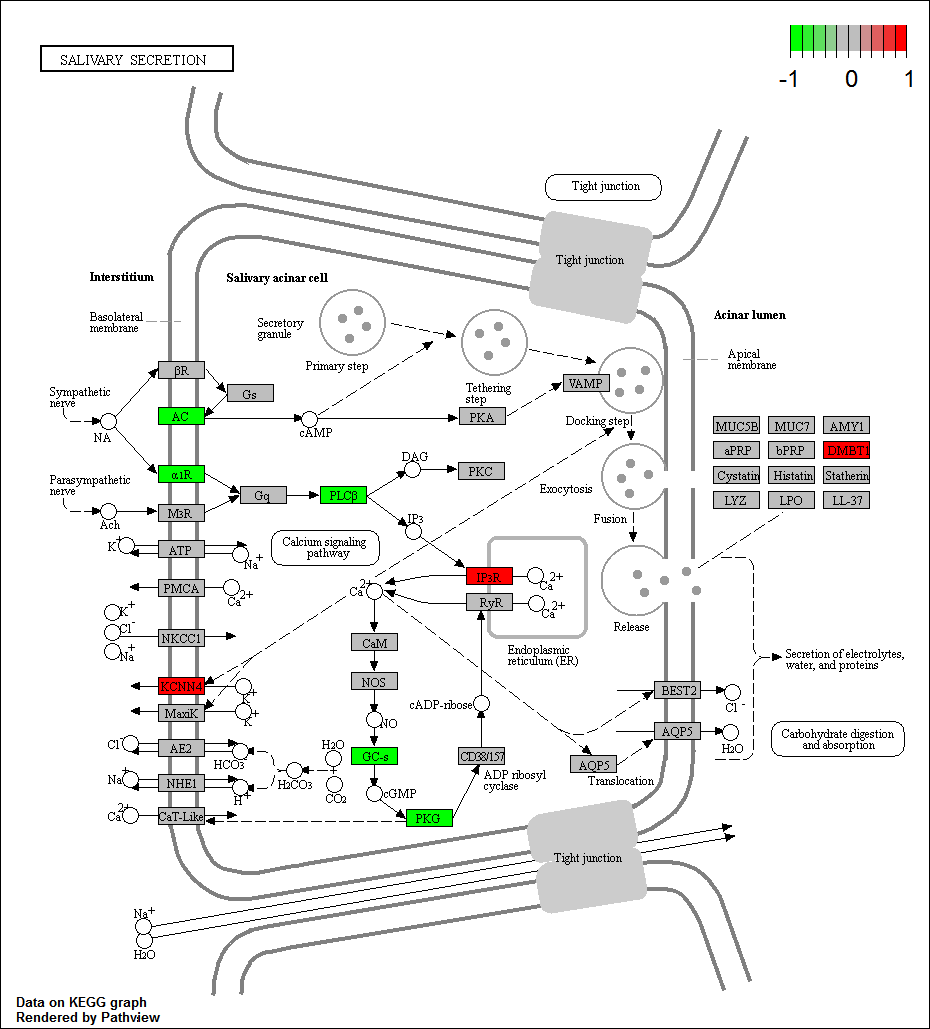

Supplement: DATASET S4 — GO-term analyses for GATA3-expressing and scratched pHAs versus EGFP-expressing and scratched pHAs in 2D cultures. [file Data_Sheet_4.ZIP › GO_term_analyses_GATA3s_vs_GFPs/GOSeq/hsa04970.Salivarysecretion.png]

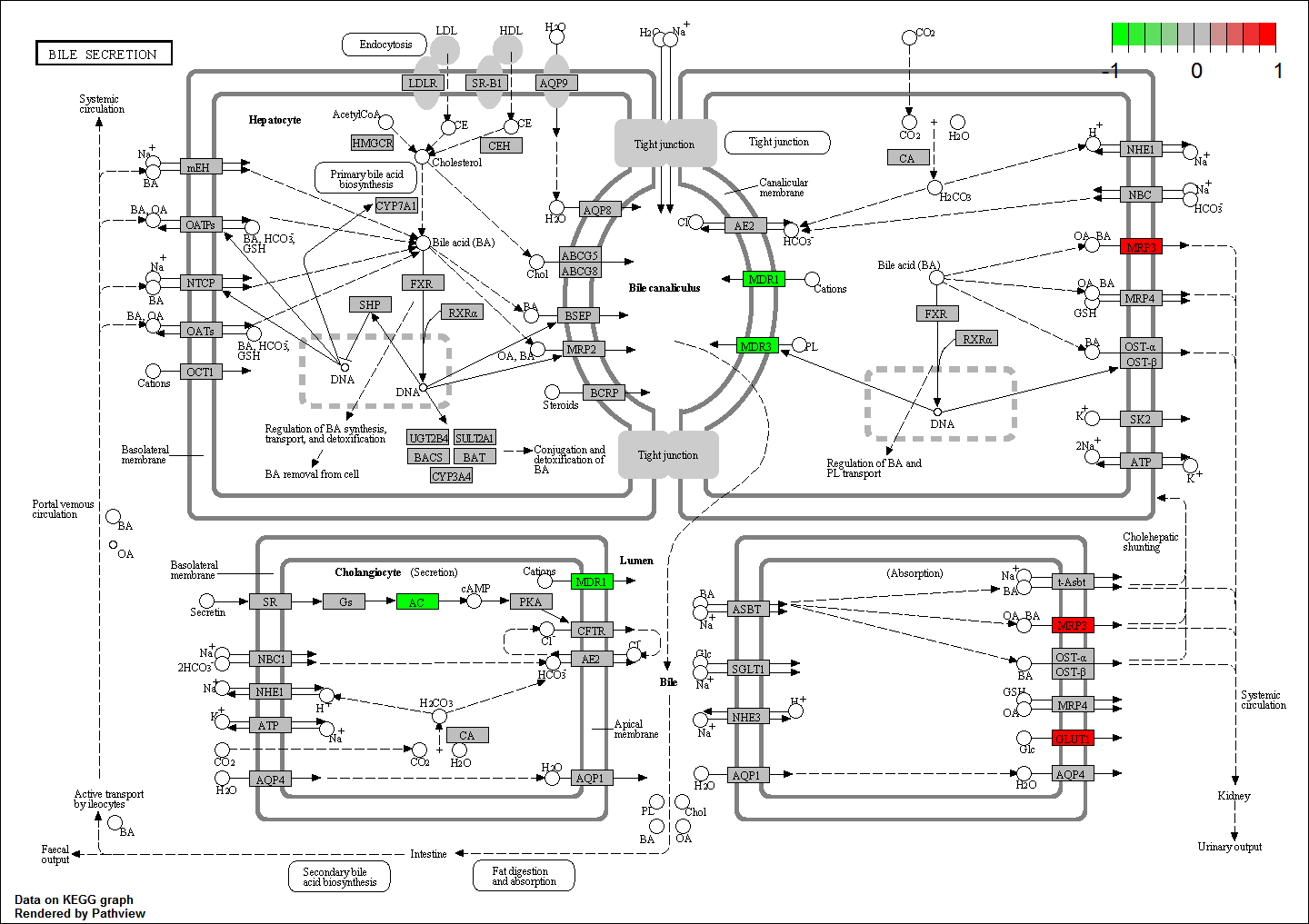

Supplement: DATASET S4 — GO-term analyses for GATA3-expressing and scratched pHAs versus EGFP-expressing and scratched pHAs in 2D cultures. [file Data_Sheet_4.ZIP › GO_term_analyses_GATA3s_vs_GFPs/GOSeq/hsa04976.Bilesecretion.png]

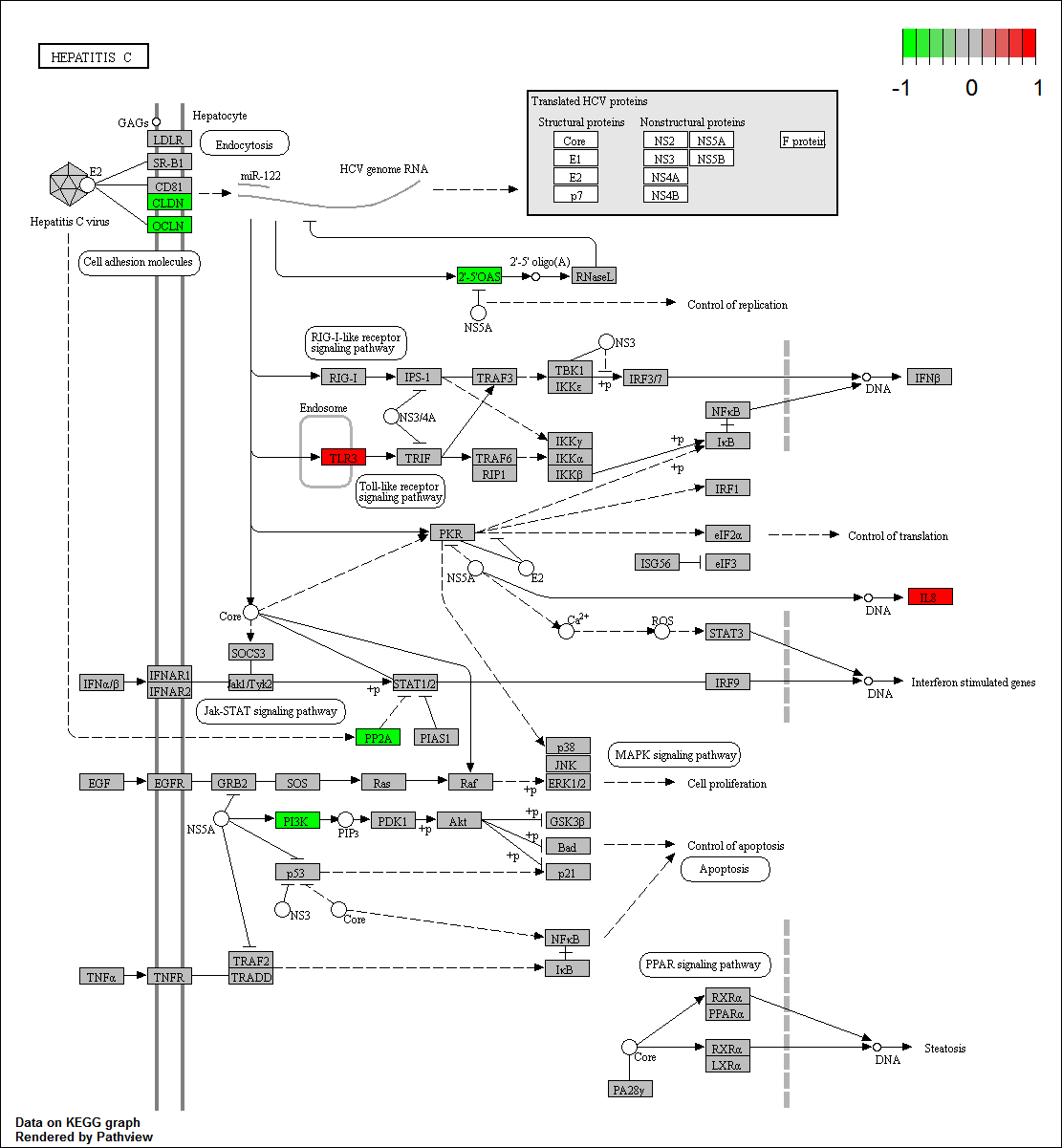

Supplement: DATASET S4 — GO-term analyses for GATA3-expressing and scratched pHAs versus EGFP-expressing and scratched pHAs in 2D cultures. [file Data_Sheet_4.ZIP › GO_term_analyses_GATA3s_vs_GFPs/GOSeq/hsa05160.HepatitisC.png]

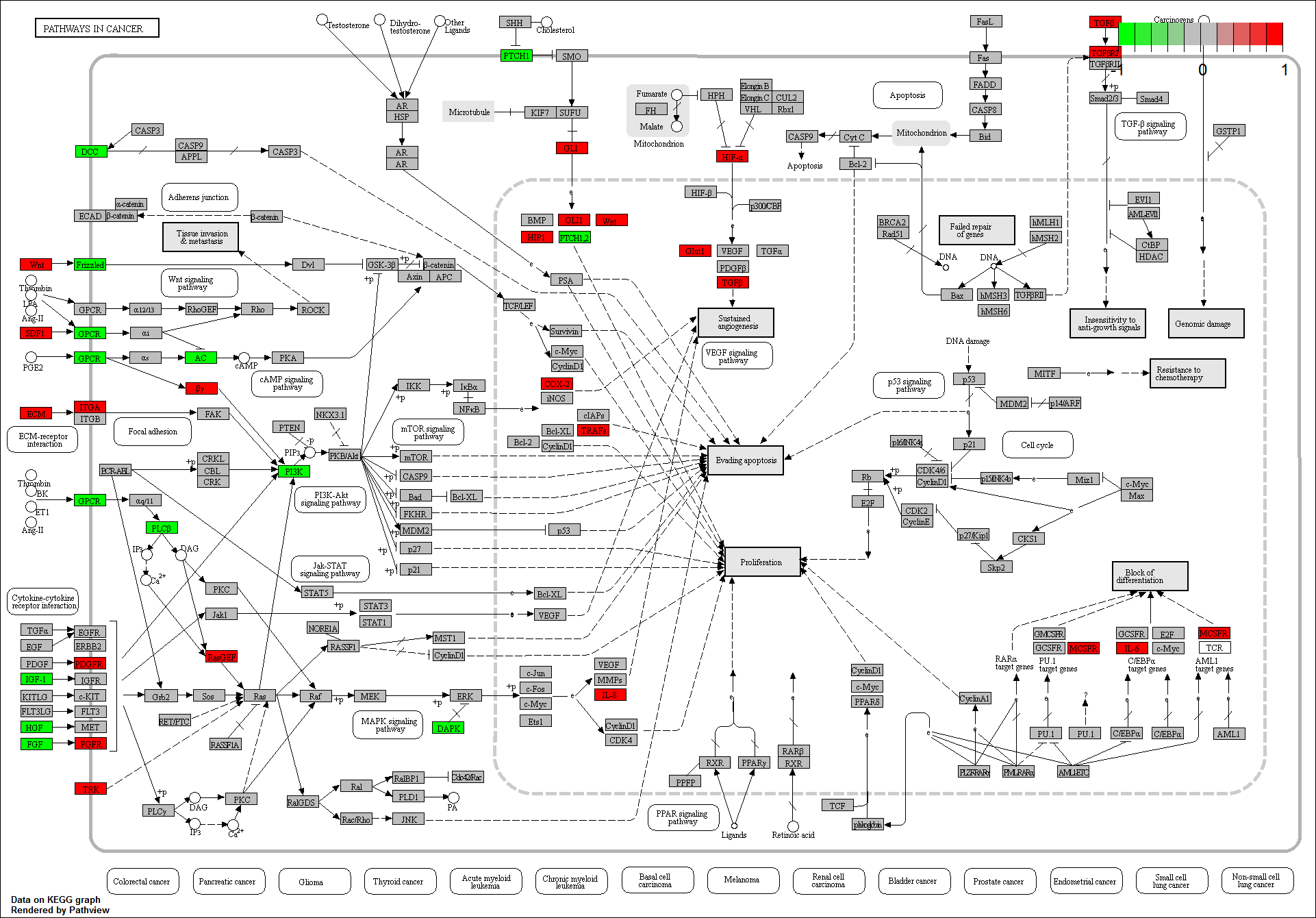

Supplement: DATASET S4 — GO-term analyses for GATA3-expressing and scratched pHAs versus EGFP-expressing and scratched pHAs in 2D cultures. [file Data_Sheet_4.ZIP › GO_term_analyses_GATA3s_vs_GFPs/GOSeq/hsa05200.Pathwaysincancer.png]

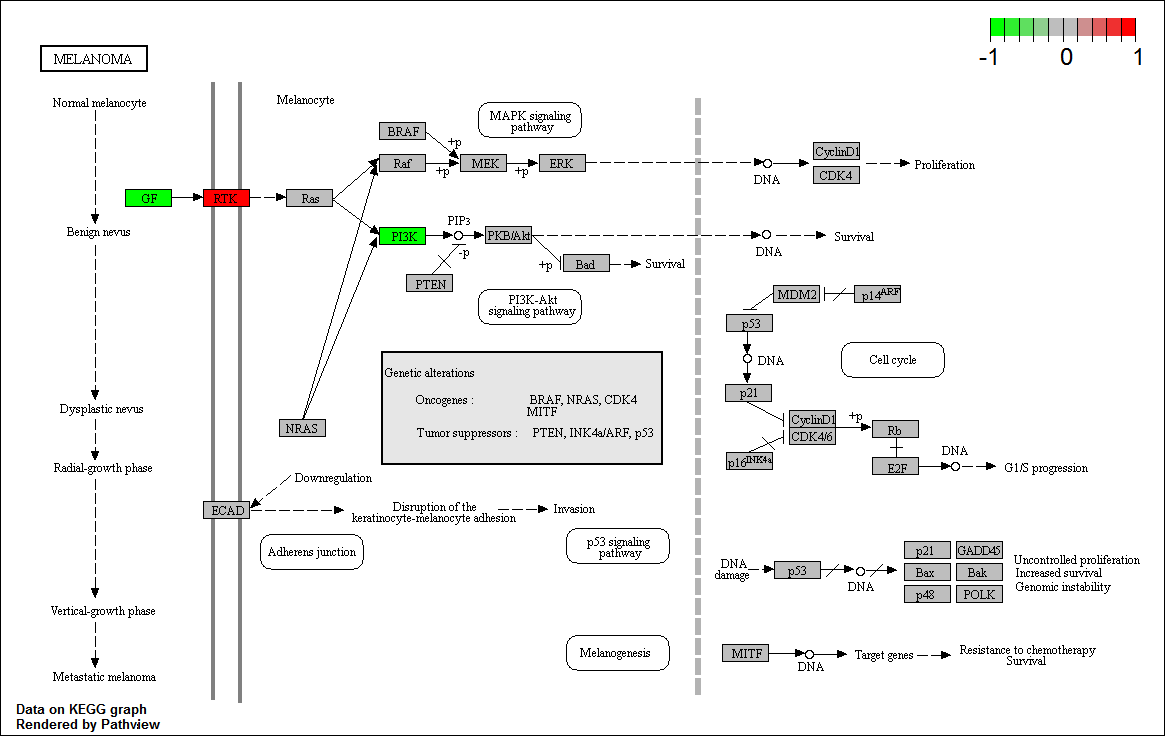

Supplement: DATASET S4 — GO-term analyses for GATA3-expressing and scratched pHAs versus EGFP-expressing and scratched pHAs in 2D cultures. [file Data_Sheet_4.ZIP › GO_term_analyses_GATA3s_vs_GFPs/GOSeq/hsa05218.Melanoma.png]

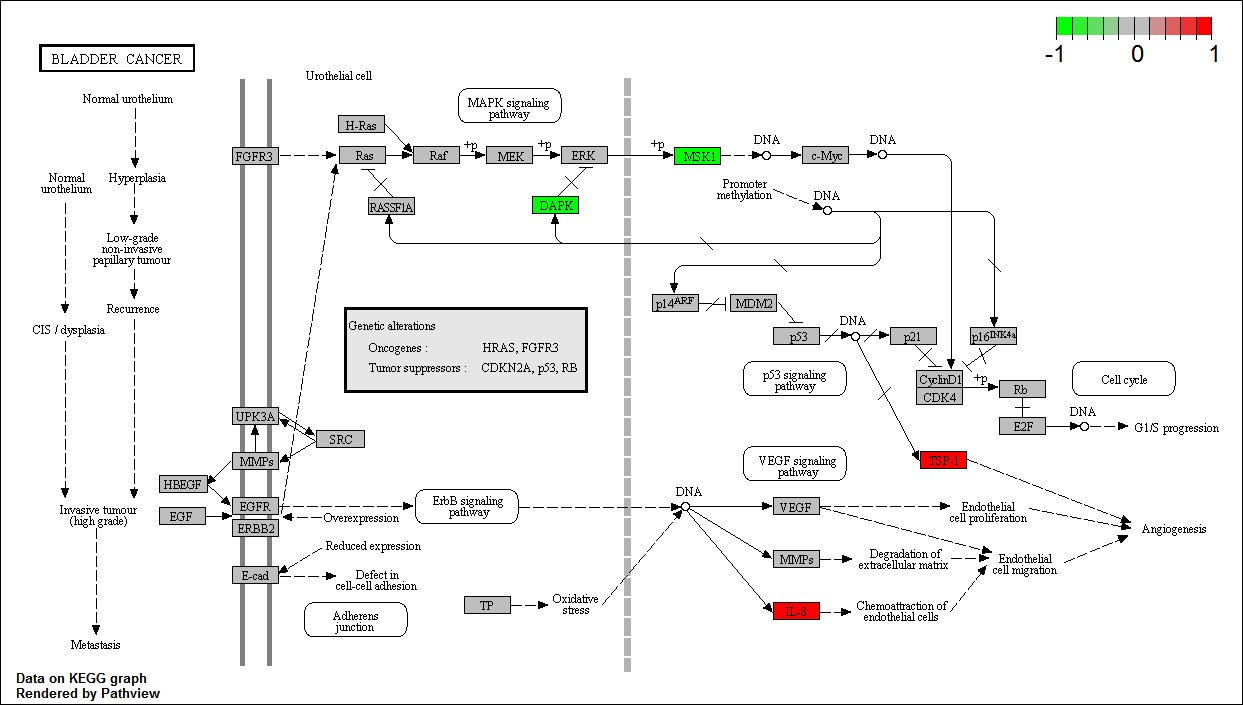

Supplement: DATASET S4 — GO-term analyses for GATA3-expressing and scratched pHAs versus EGFP-expressing and scratched pHAs in 2D cultures. [file Data_Sheet_4.ZIP › GO_term_analyses_GATA3s_vs_GFPs/GOSeq/hsa05219.Bladdercancer.png]

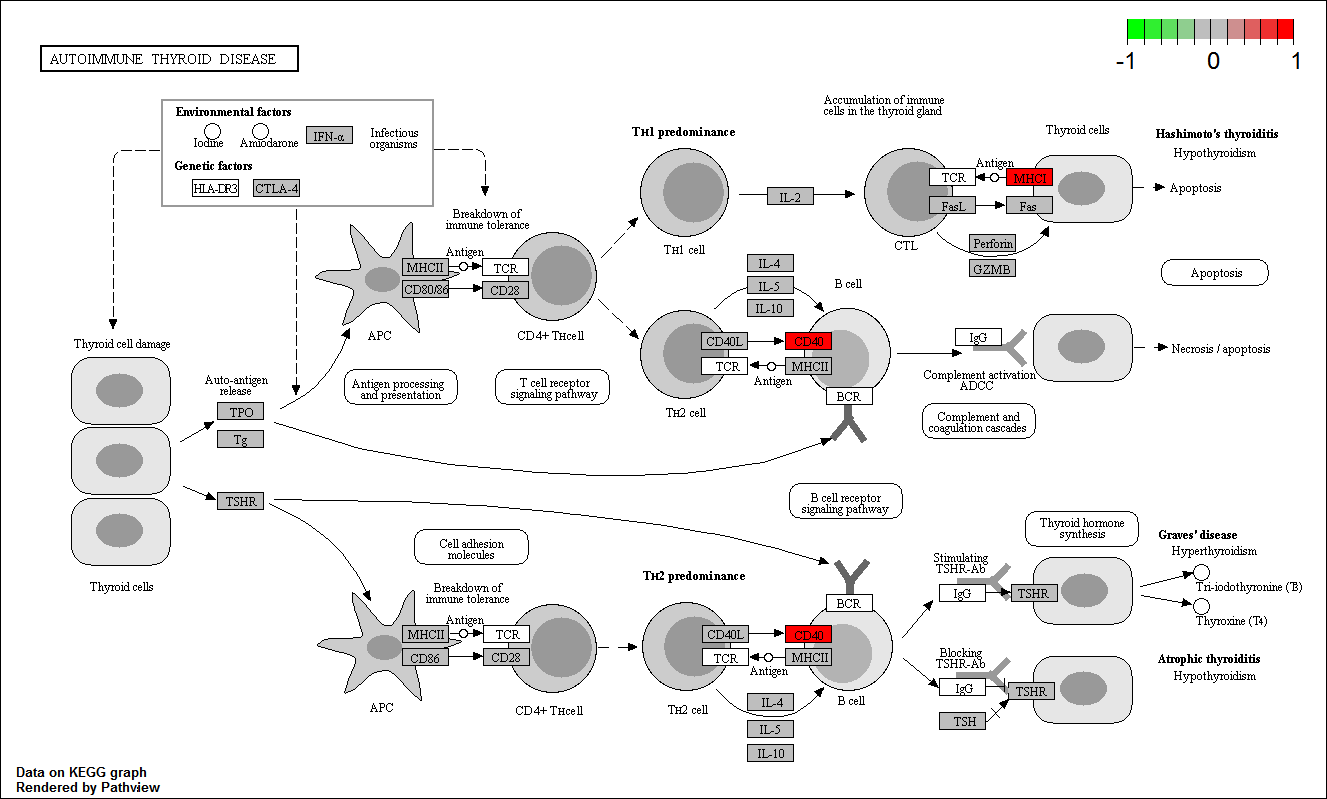

Supplement: DATASET S4 — GO-term analyses for GATA3-expressing and scratched pHAs versus EGFP-expressing and scratched pHAs in 2D cultures. [file Data_Sheet_4.ZIP › GO_term_analyses_GATA3s_vs_GFPs/GOSeq/hsa05320.Autoimmunethyroiddisease.png]

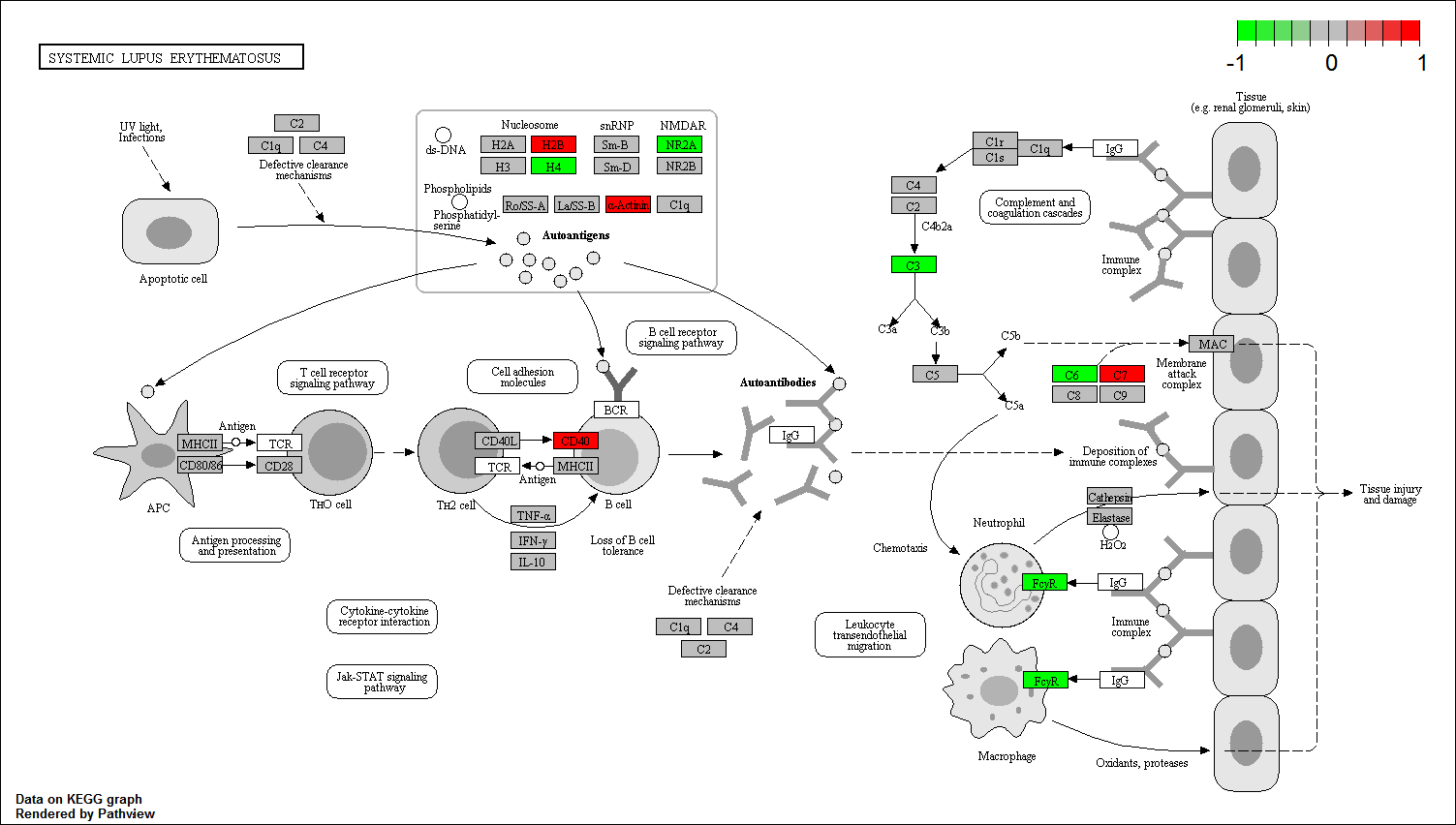

Supplement: DATASET S4 — GO-term analyses for GATA3-expressing and scratched pHAs versus EGFP-expressing and scratched pHAs in 2D cultures. [file Data_Sheet_4.ZIP › GO_term_analyses_GATA3s_vs_GFPs/GOSeq/hsa05322.Systemiclupuserythematosus.png]

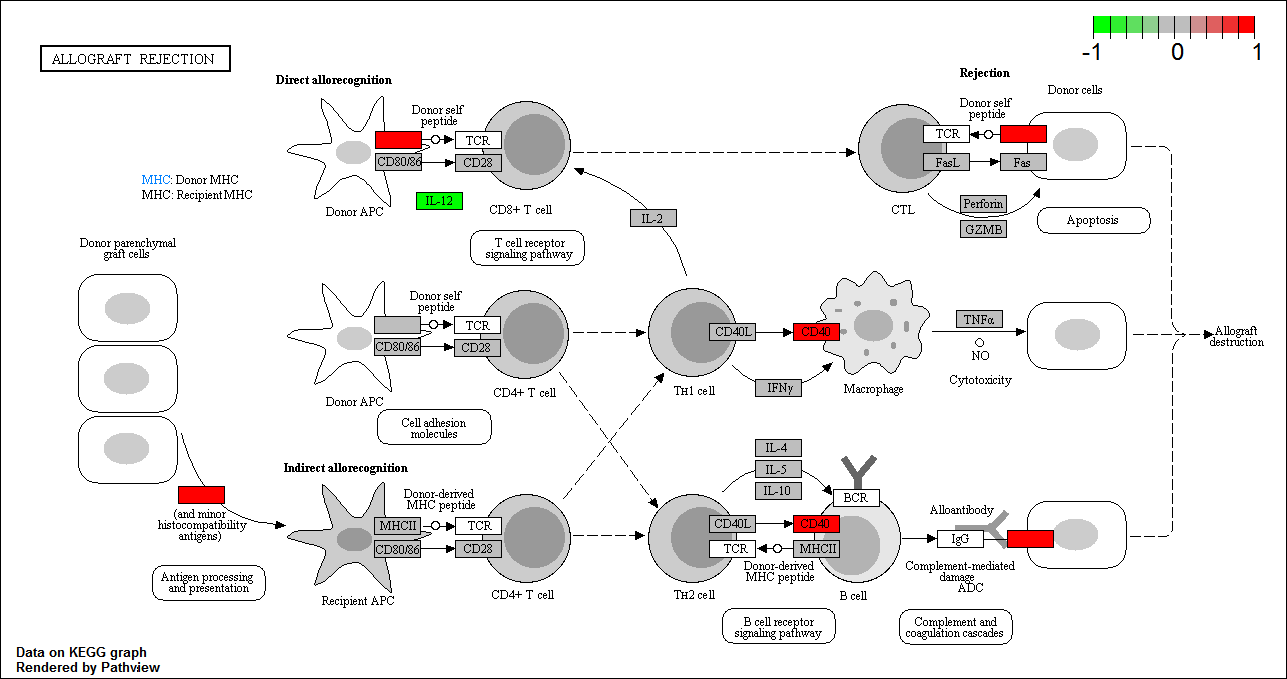

Supplement: DATASET S4 — GO-term analyses for GATA3-expressing and scratched pHAs versus EGFP-expressing and scratched pHAs in 2D cultures. [file Data_Sheet_4.ZIP › GO_term_analyses_GATA3s_vs_GFPs/GOSeq/hsa05330.Allograftrejection.png]

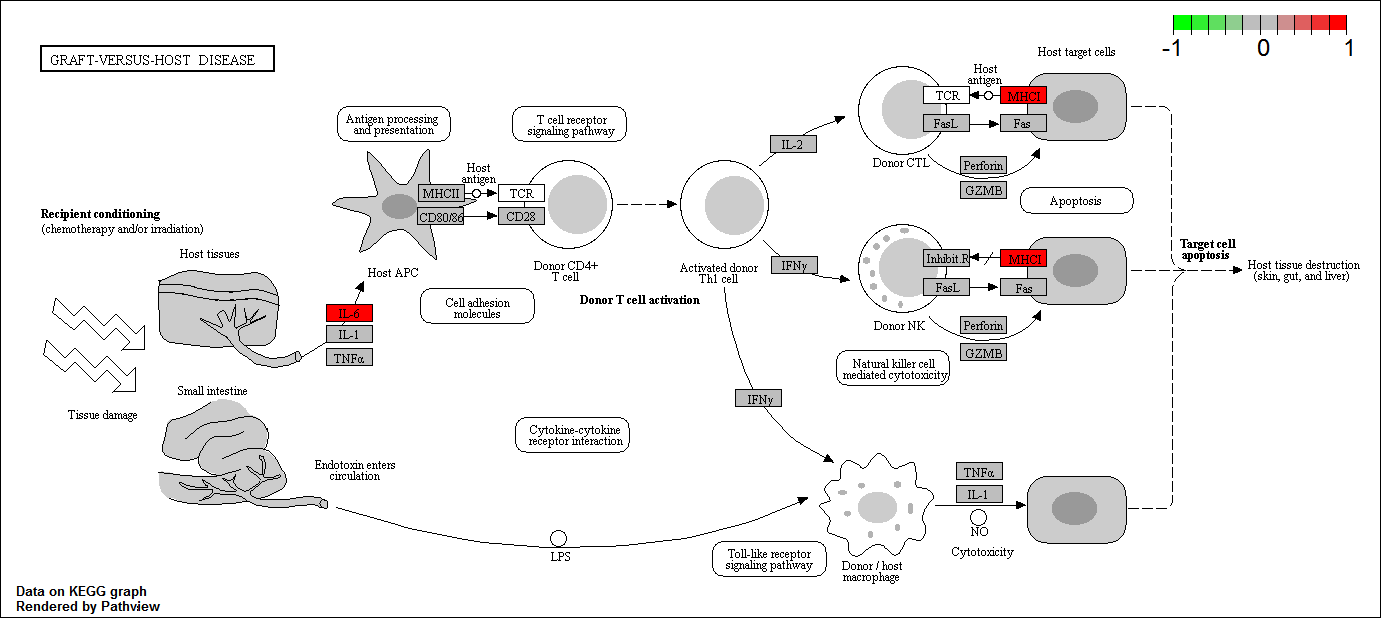

Supplement: DATASET S4 — GO-term analyses for GATA3-expressing and scratched pHAs versus EGFP-expressing and scratched pHAs in 2D cultures. [file Data_Sheet_4.ZIP › GO_term_analyses_GATA3s_vs_GFPs/GOSeq/hsa05332.Graft-versus-hostdisease.png]

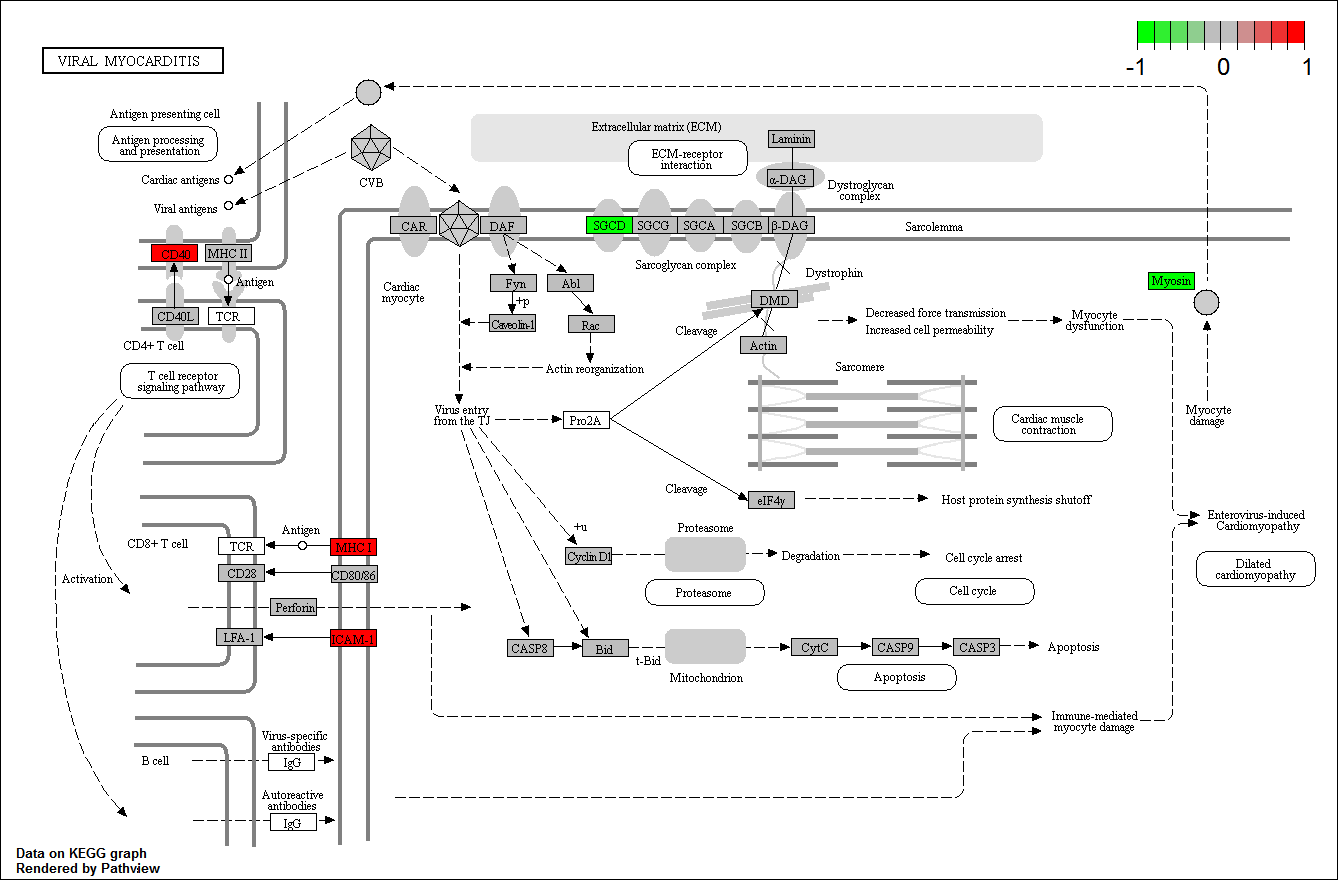

Supplement: DATASET S4 — GO-term analyses for GATA3-expressing and scratched pHAs versus EGFP-expressing and scratched pHAs in 2D cultures. [file Data_Sheet_4.ZIP › GO_term_analyses_GATA3s_vs_GFPs/GOSeq/hsa05416.Viralmyocarditis.png]

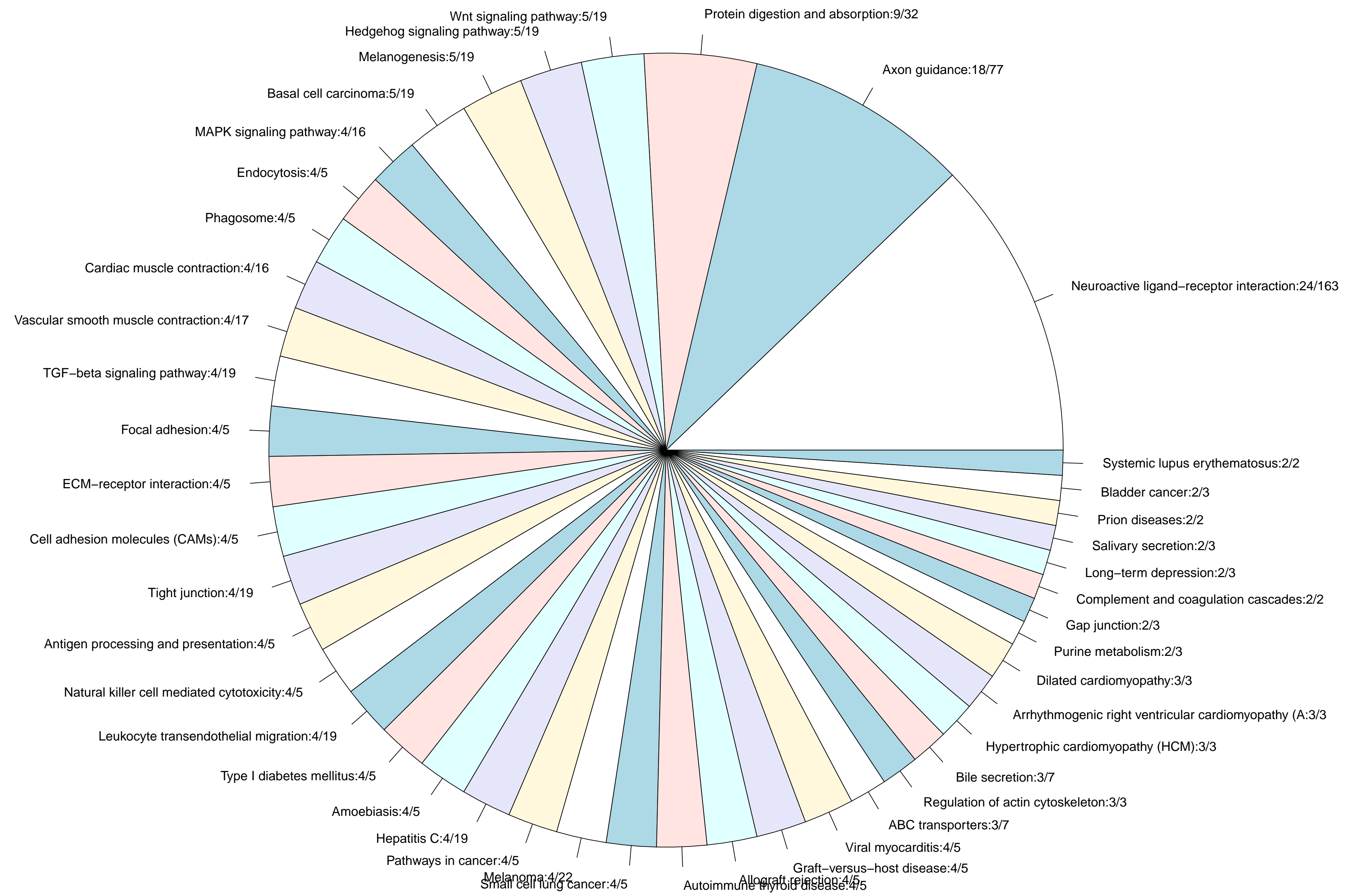

Supplement: DATASET S4 — GO-term analyses for GATA3-expressing and scratched pHAs versus EGFP-expressing and scratched pHAs in 2D cultures. [file Data_Sheet_4.ZIP › GO_term_analyses_GATA3s_vs_GFPs/GOSeq/KEGG_adjusted.GO.kegg.Over.BH.pdf]

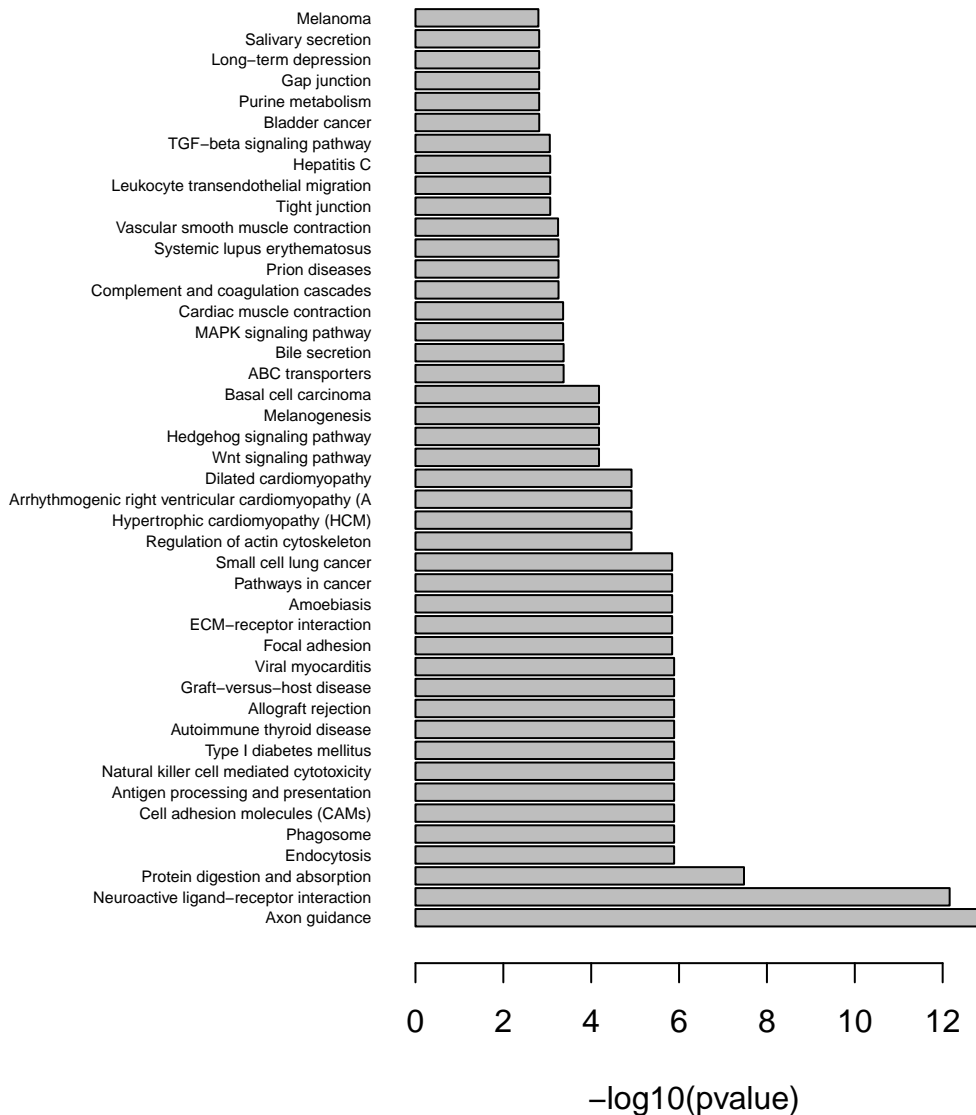

Supplement: DATASET S4 — GO-term analyses for GATA3-expressing and scratched pHAs versus EGFP-expressing and scratched pHAs in 2D cultures. [file Data_Sheet_4.ZIP › GO_term_analyses_GATA3s_vs_GFPs/GOSeq/pVal_KEGG_adjusted.GO.kegg.Over.BH.pdf]

GOstats\_BP\_Down\_pieChart

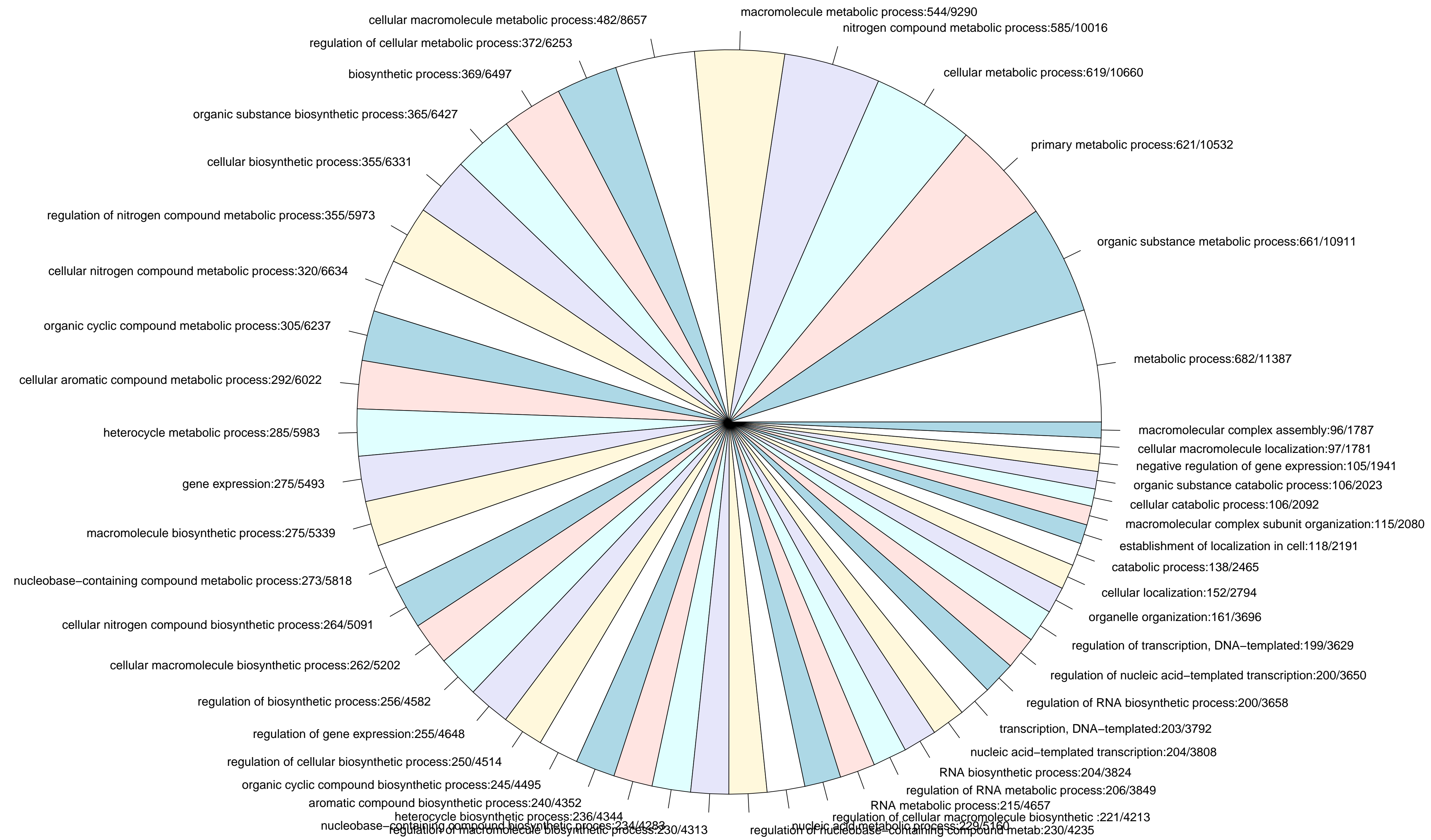

Supplement: DATASET S4 — GO-term analyses for GATA3-expressing and scratched pHAs versus EGFP-expressing and scratched pHAs in 2D cultures. [file Data_Sheet_4.ZIP › GO_term_analyses_GATA3s_vs_GFPs/GOstats/GOstats_BP_Down_pieChart.pdf]

GOstats\_BP\_Up\_pieChart

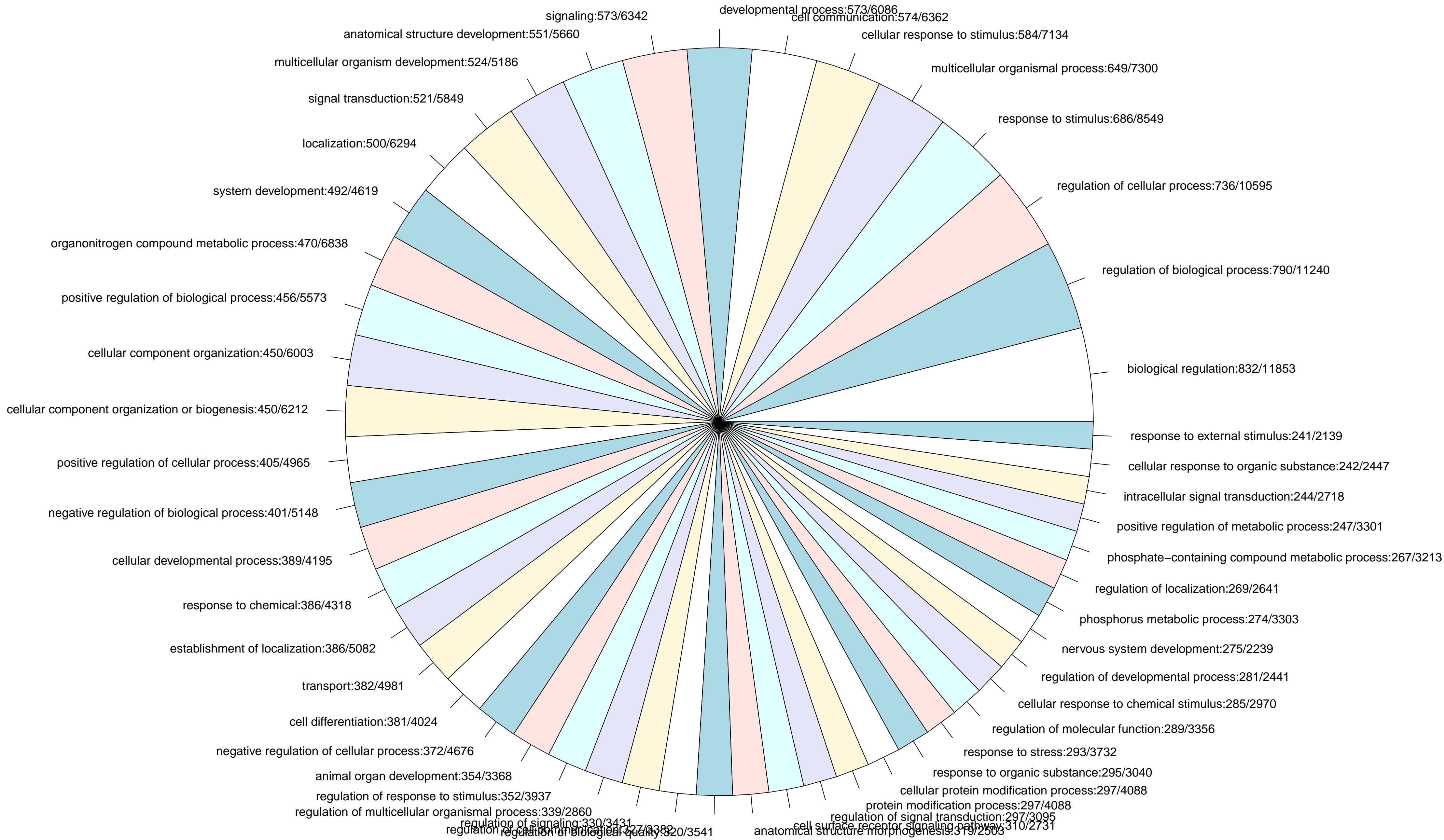

Supplement: DATASET S4 — GO-term analyses for GATA3-expressing and scratched pHAs versus EGFP-expressing and scratched pHAs in 2D cultures. [file Data_Sheet_4.ZIP › GO_term_analyses_GATA3s_vs_GFPs/GOstats/GOstats_BP_Up_pieChart.pdf]

### GOstats\_CC\_Down\_pieChart

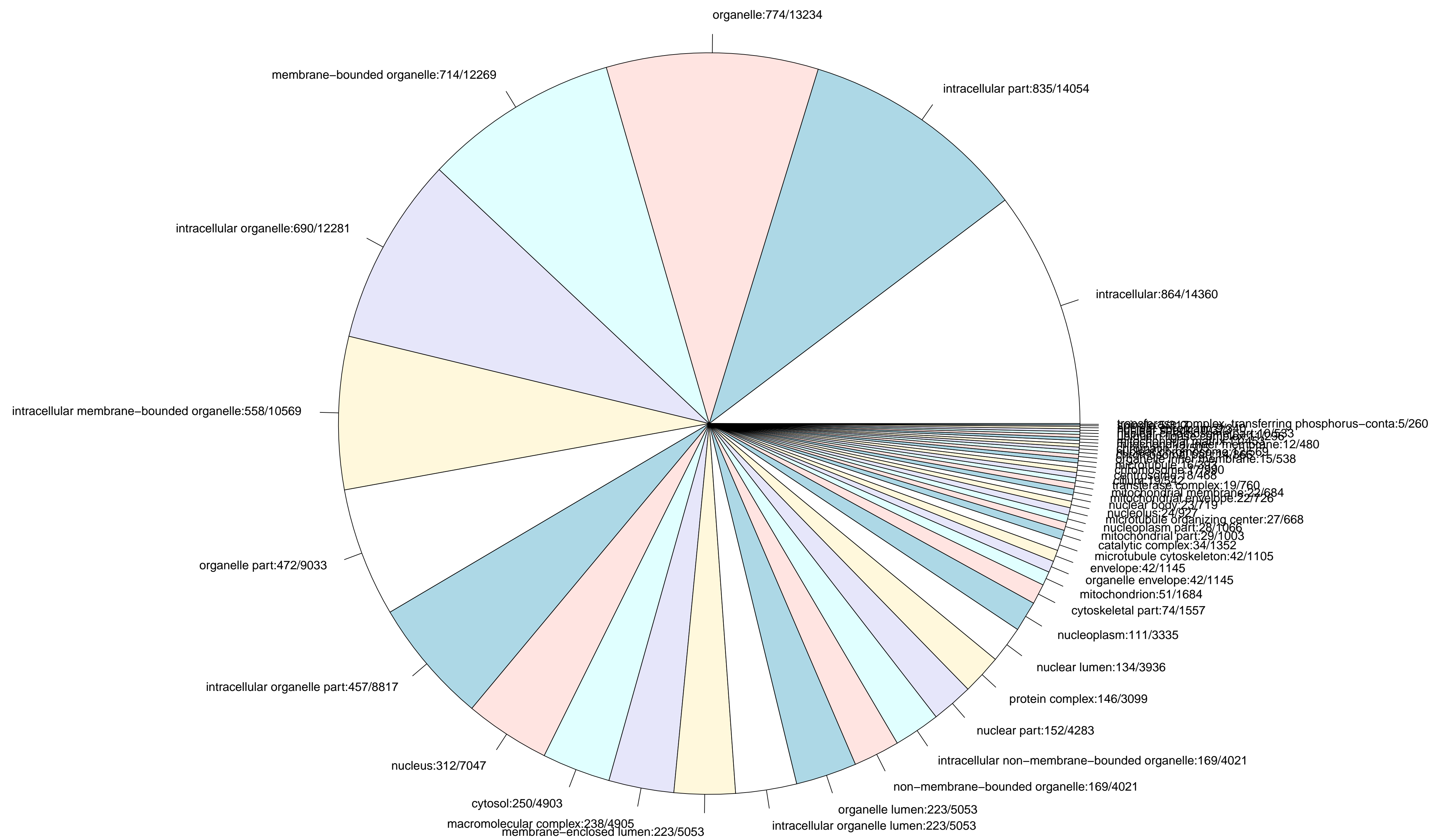

Supplement: DATASET S4 — GO-term analyses for GATA3-expressing and scratched pHAs versus EGFP-expressing and scratched pHAs in 2D cultures. [file Data_Sheet_4.ZIP › GO_term_analyses_GATA3s_vs_GFPs/GOstats/GOstats_CC_Down_pieChart.pdf]

GOstats\_CC\_Up\_pieChart

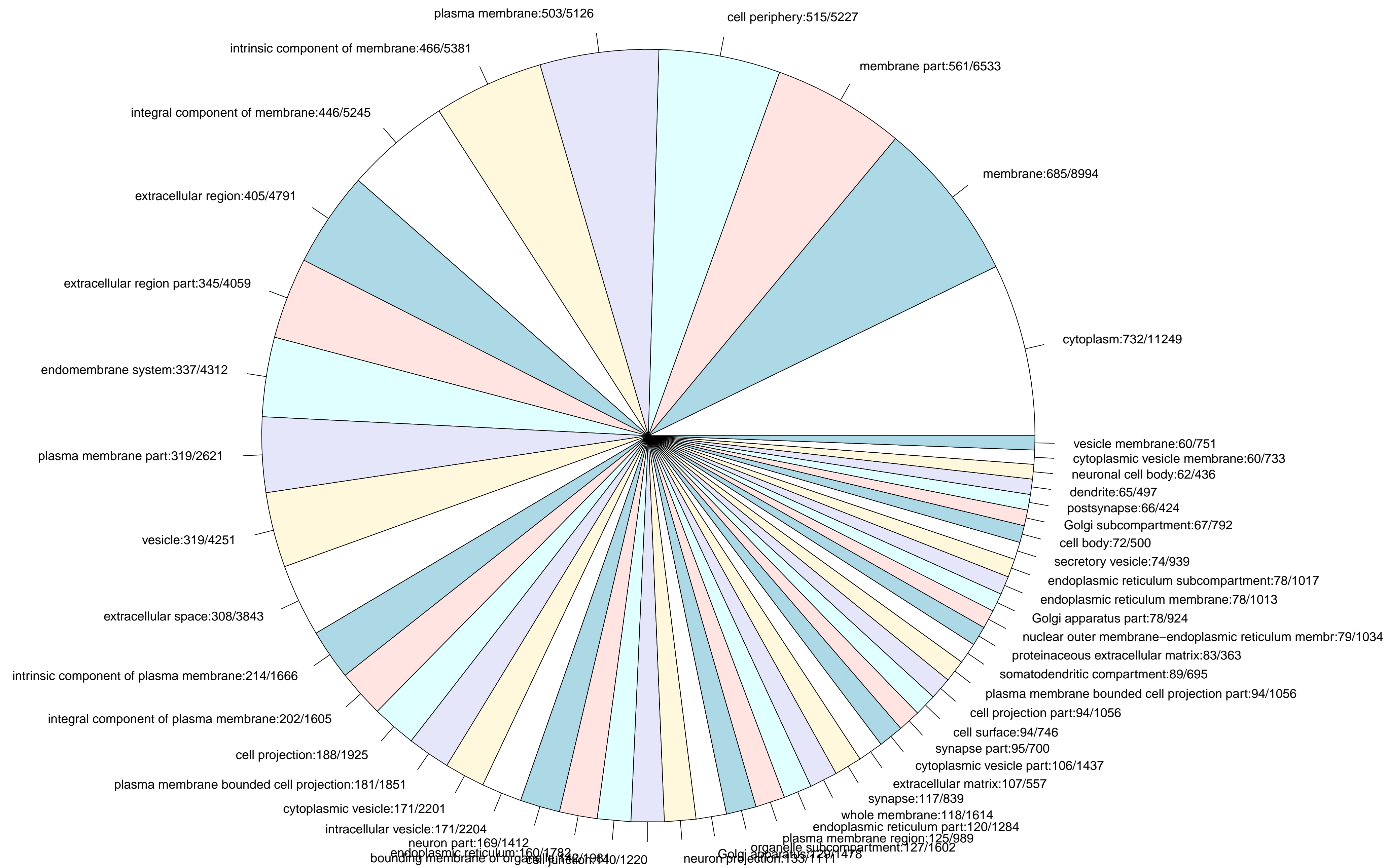

Supplement: DATASET S4 — GO-term analyses for GATA3-expressing and scratched pHAs versus EGFP-expressing and scratched pHAs in 2D cultures. [file Data_Sheet_4.ZIP › GO_term_analyses_GATA3s_vs_GFPs/GOstats/GOstats_CC_Up_pieChart.pdf]

GOstats\_kegg\_Under

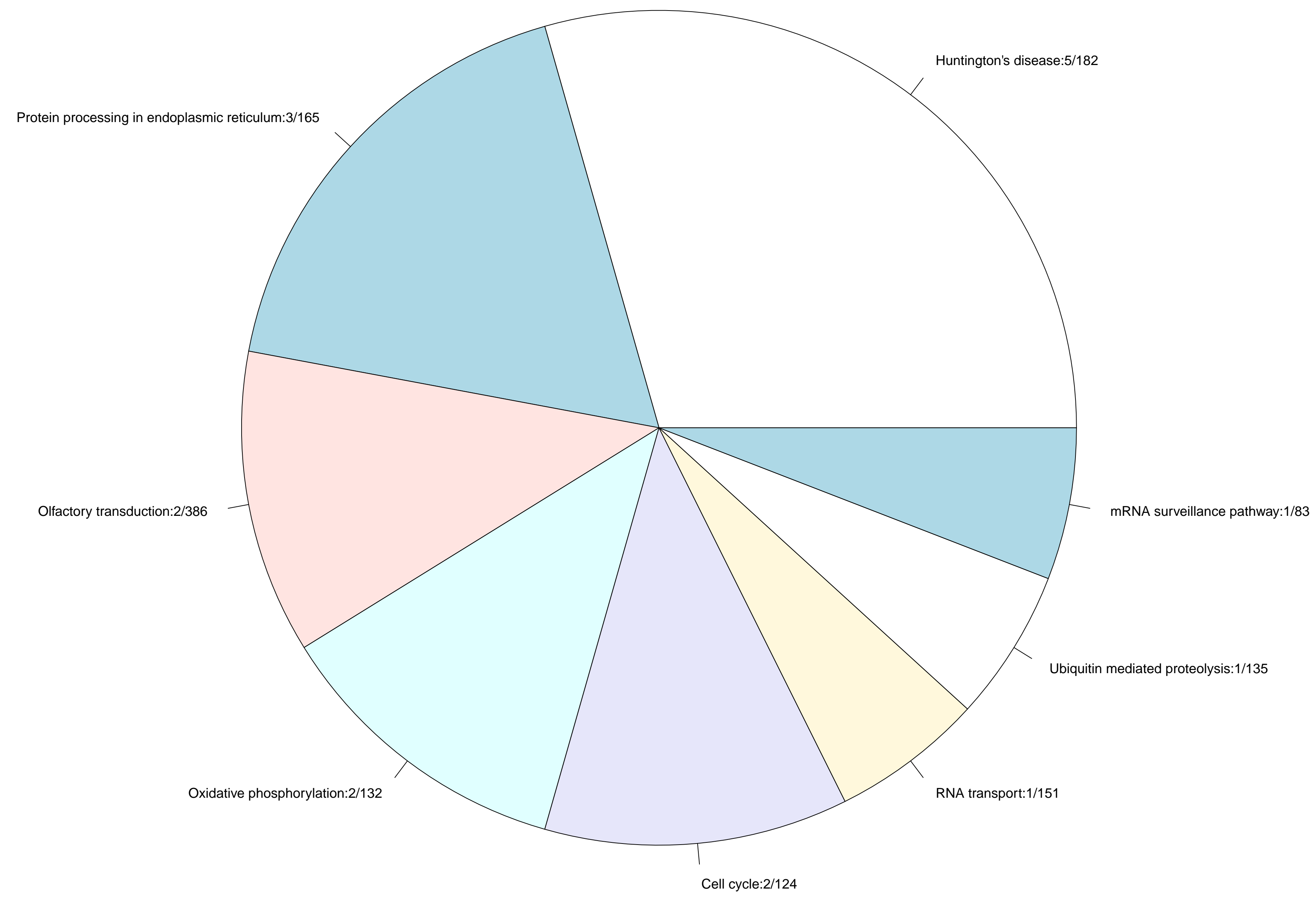

Supplement: DATASET S4 — GO-term analyses for GATA3-expressing and scratched pHAs versus EGFP-expressing and scratched pHAs in 2D cultures. [file Data_Sheet_4.ZIP › GO_term_analyses_GATA3s_vs_GFPs/GOstats/GOstats_kegg_Under.pdf]

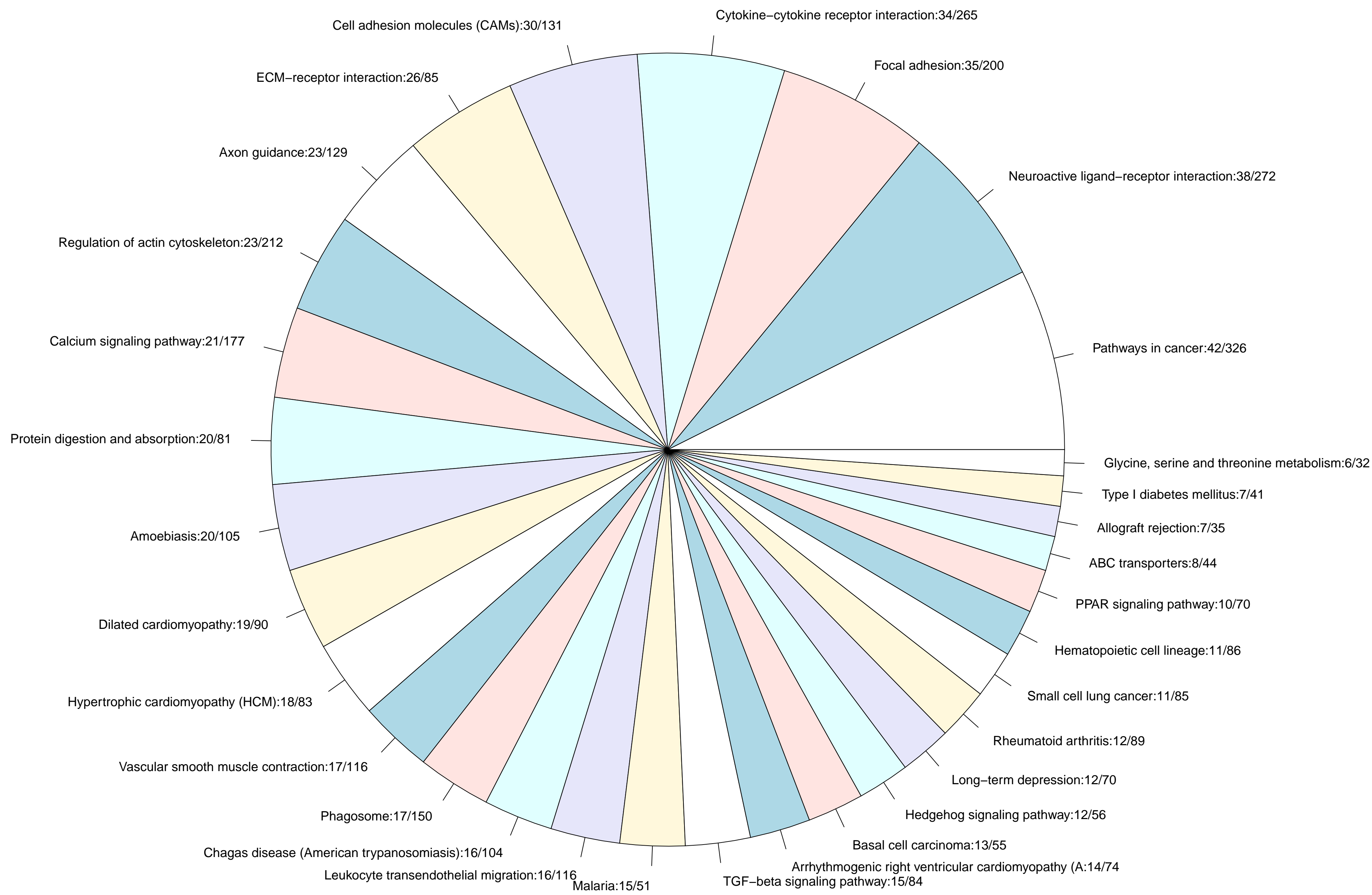

Supplement: DATASET S4 — GO-term analyses for GATA3-expressing and scratched pHAs versus EGFP-expressing and scratched pHAs in 2D cultures. [file Data_Sheet_4.ZIP › GO_term_analyses_GATA3s_vs_GFPs/GOstats/GOstats_kegg_Up.pdf]

### GOstats\_MF\_Down\_pieChart

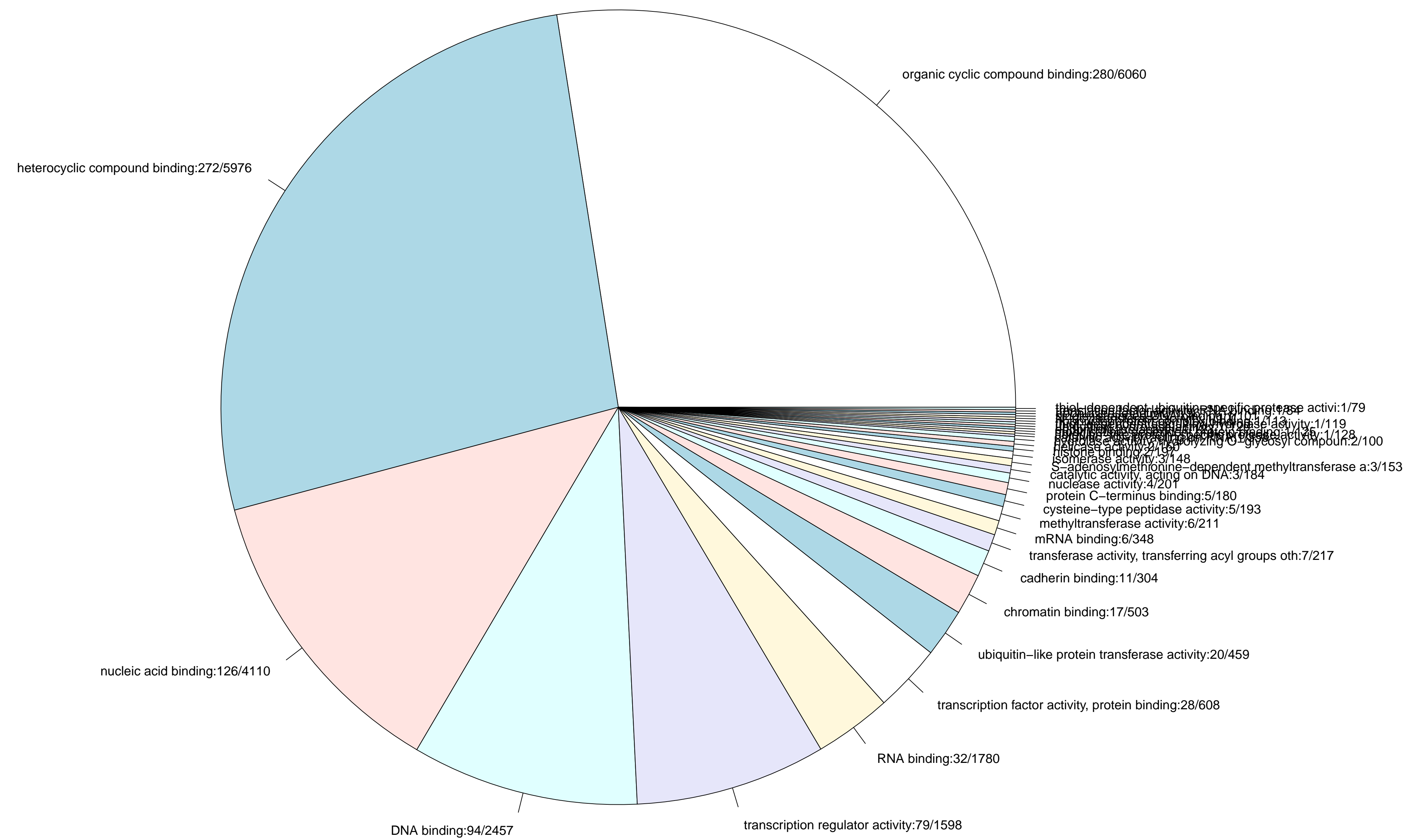

Supplement: DATASET S4 — GO-term analyses for GATA3-expressing and scratched pHAs versus EGFP-expressing and scratched pHAs in 2D cultures. [file Data_Sheet_4.ZIP › GO_term_analyses_GATA3s_vs_GFPs/GOstats/GOstats_MF_Down_pieChart.pdf]

GOstats\_MF\_Up\_pieChart

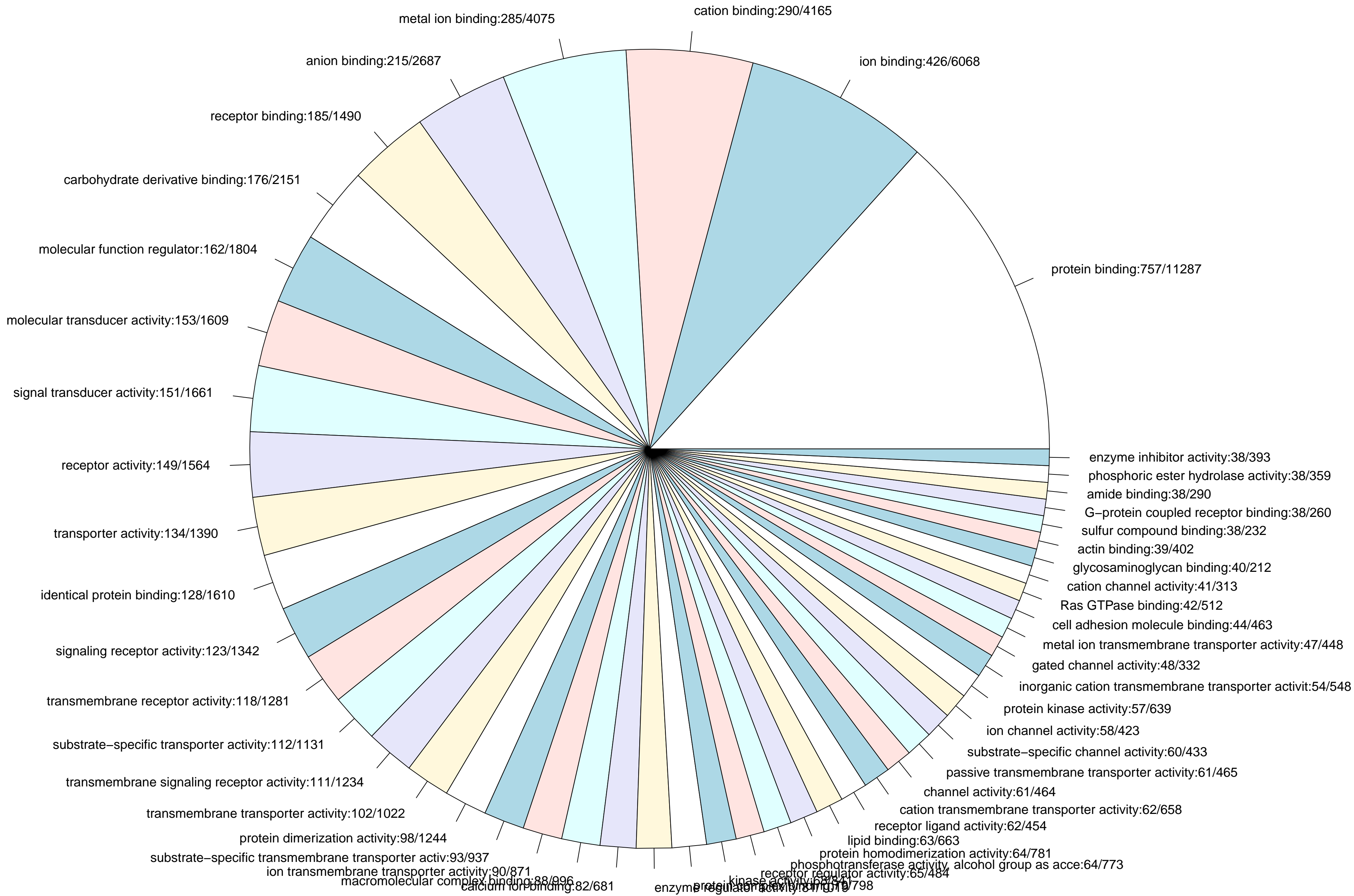

Supplement: DATASET S4 — GO-term analyses for GATA3-expressing and scratched pHAs versus EGFP-expressing and scratched pHAs in 2D cultures. [file Data_Sheet_4.ZIP › GO_term_analyses_GATA3s_vs_GFPs/GOstats/GOstats_MF_Up_pieChart.pdf]

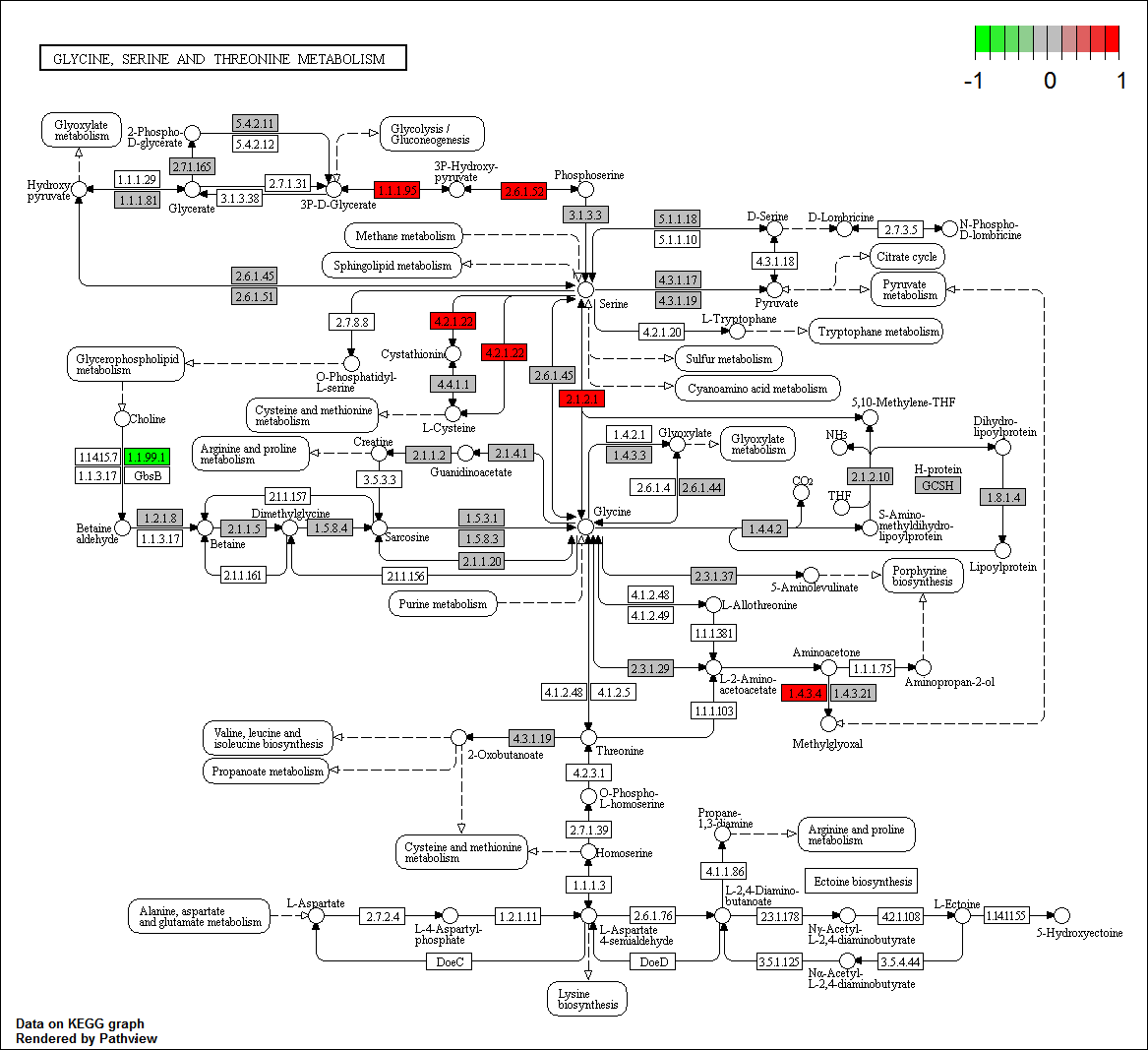

Supplement: DATASET S4 — GO-term analyses for GATA3-expressing and scratched pHAs versus EGFP-expressing and scratched pHAs in 2D cultures. [file Data_Sheet_4.ZIP › GO_term_analyses_GATA3s_vs_GFPs/GOstats/hsa00260.Glycine,serineandthreoninemetabolism.png]

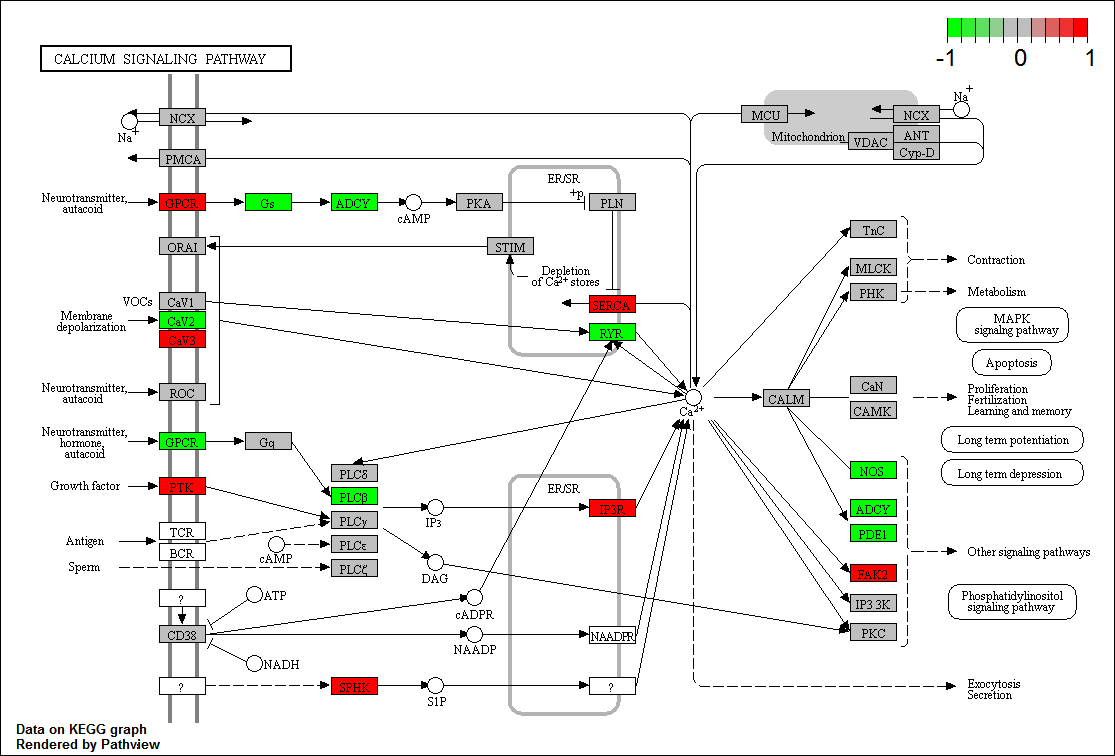

Supplement: DATASET S4 — GO-term analyses for GATA3-expressing and scratched pHAs versus EGFP-expressing and scratched pHAs in 2D cultures. [file Data_Sheet_4.ZIP › GO_term_analyses_GATA3s_vs_GFPs/GOstats/hsa04020.Calciumsignalingpathway.png]

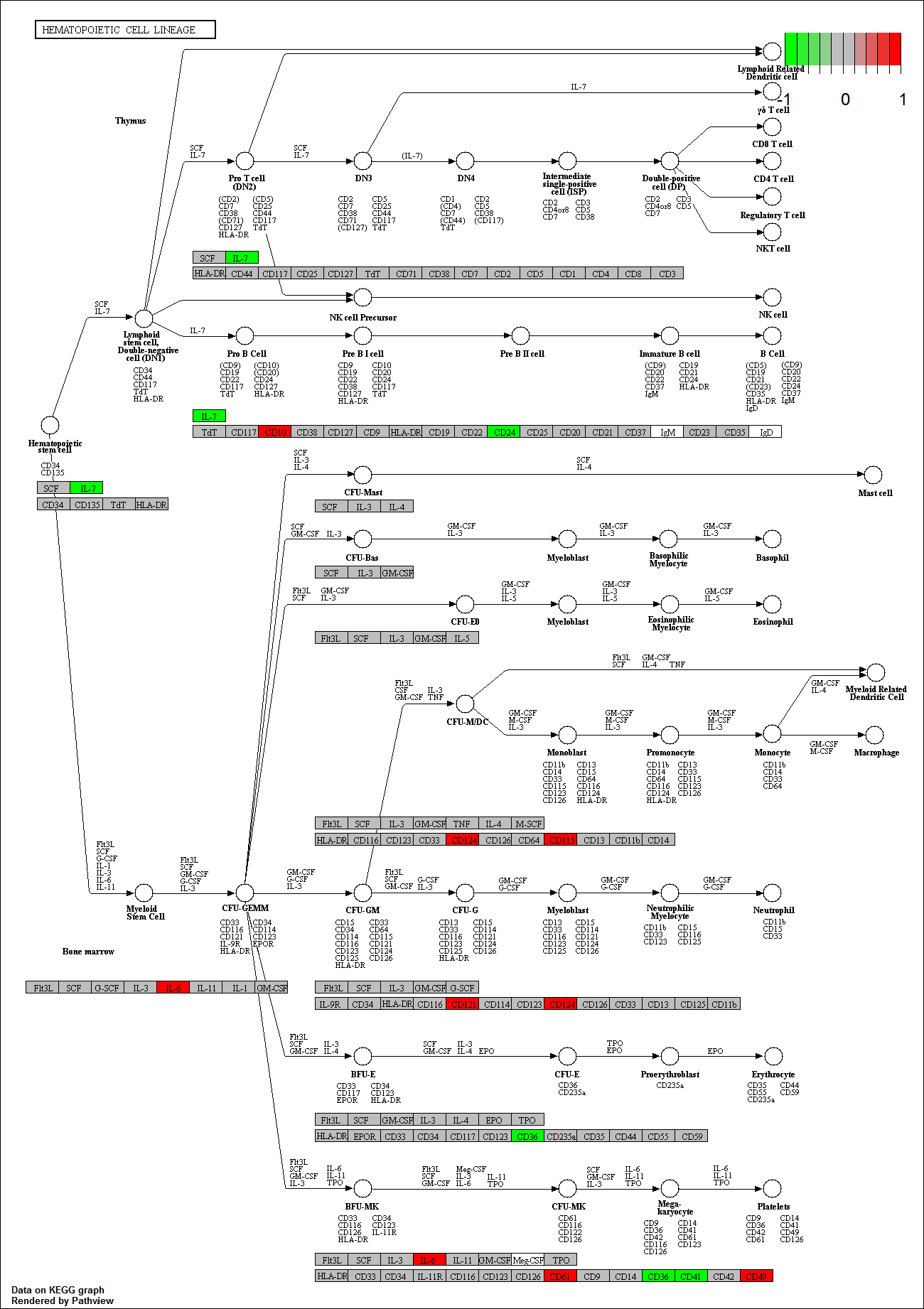

Supplement: DATASET S4 — GO-term analyses for GATA3-expressing and scratched pHAs versus EGFP-expressing and scratched pHAs in 2D cultures. [file Data_Sheet_4.ZIP › GO_term_analyses_GATA3s_vs_GFPs/GOstats/hsa04640.Hematopoieticcelllineage.png]

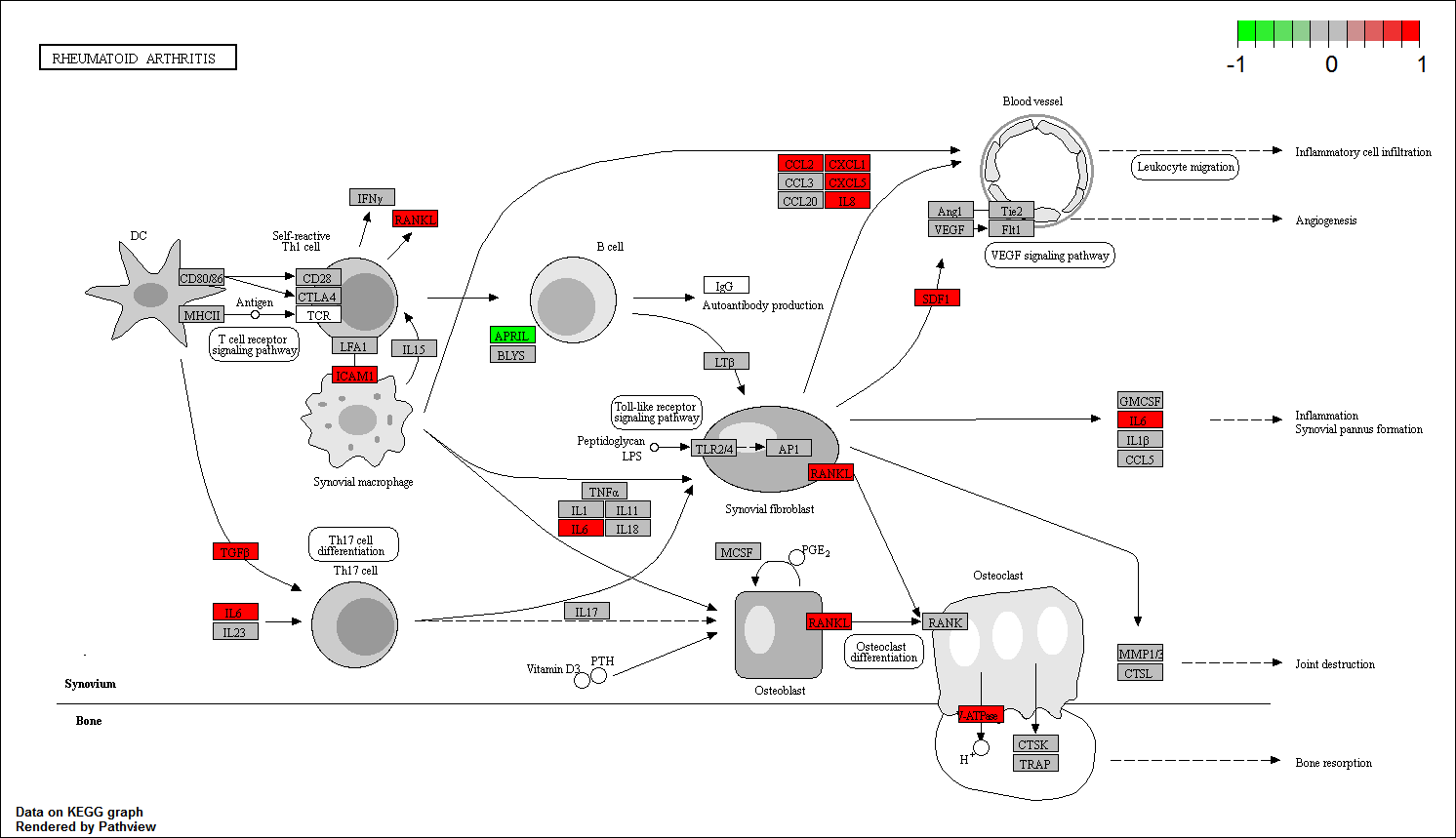

Supplement: DATASET S4 — GO-term analyses for GATA3-expressing and scratched pHAs versus EGFP-expressing and scratched pHAs in 2D cultures. [file Data_Sheet_4.ZIP › GO_term_analyses_GATA3s_vs_GFPs/GOstats/hsa05323.Rheumatoidarthritis.png]

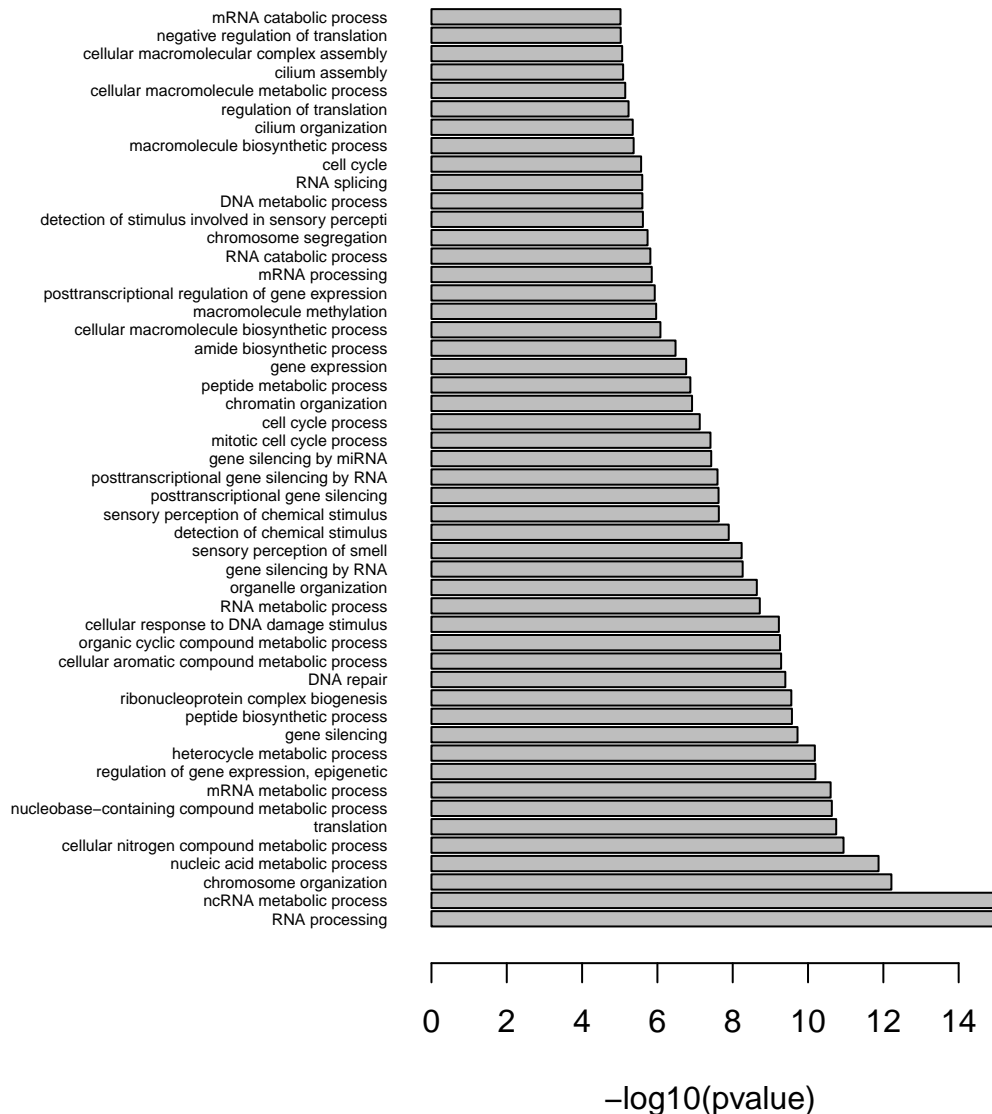

Supplement: DATASET S4 — GO-term analyses for GATA3-expressing and scratched pHAs versus EGFP-expressing and scratched pHAs in 2D cultures. [file Data_Sheet_4.ZIP › GO_term_analyses_GATA3s_vs_GFPs/GOstats/pVal_GOstats_BP_Down_pieChart.pdf]

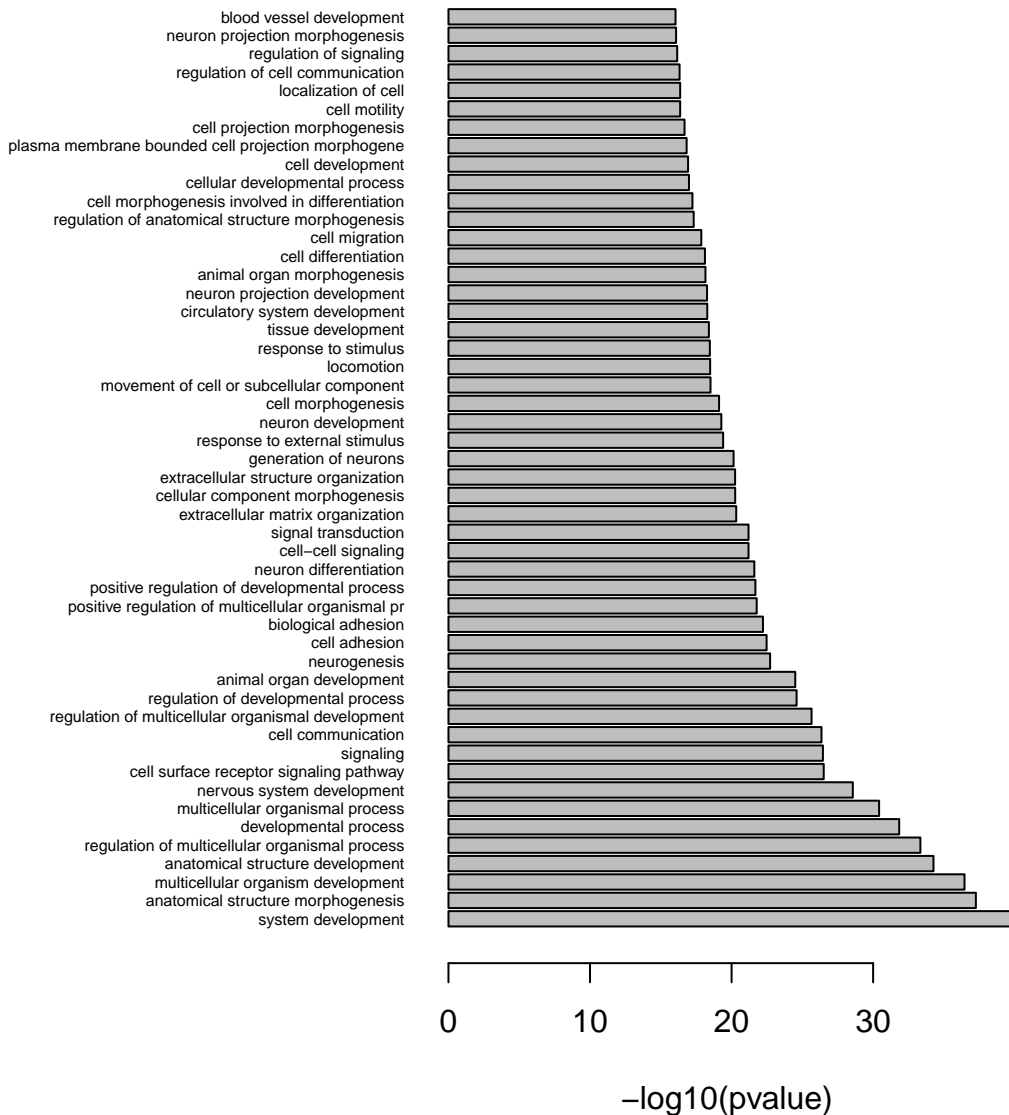

Supplement: DATASET S4 — GO-term analyses for GATA3-expressing and scratched pHAs versus EGFP-expressing and scratched pHAs in 2D cultures. [file Data_Sheet_4.ZIP › GO_term_analyses_GATA3s_vs_GFPs/GOstats/pVal_GOstats_BP_Up_pieChart.pdf]

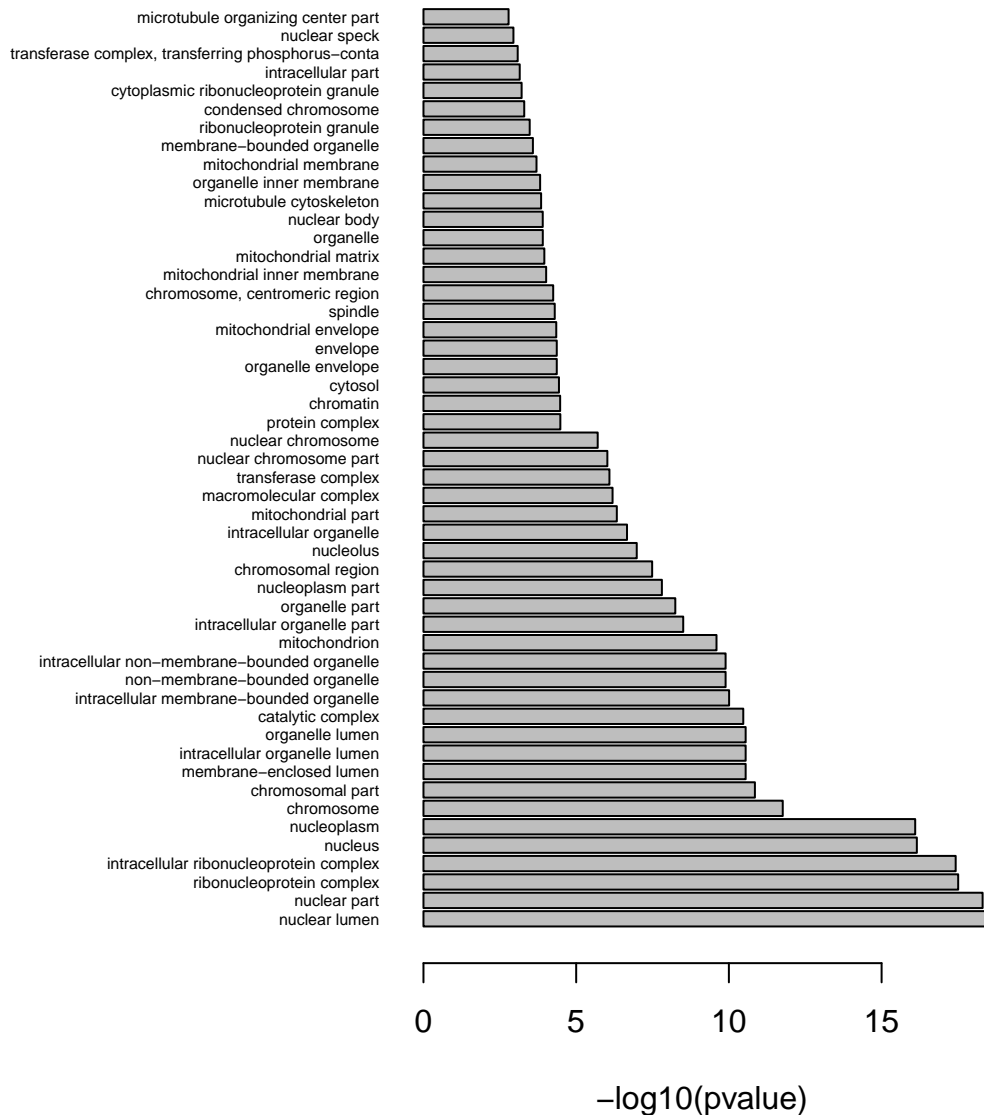

Supplement: DATASET S4 — GO-term analyses for GATA3-expressing and scratched pHAs versus EGFP-expressing and scratched pHAs in 2D cultures. [file Data_Sheet_4.ZIP › GO_term_analyses_GATA3s_vs_GFPs/GOstats/pVal_GOstats_CC_Down_pieChart.pdf]

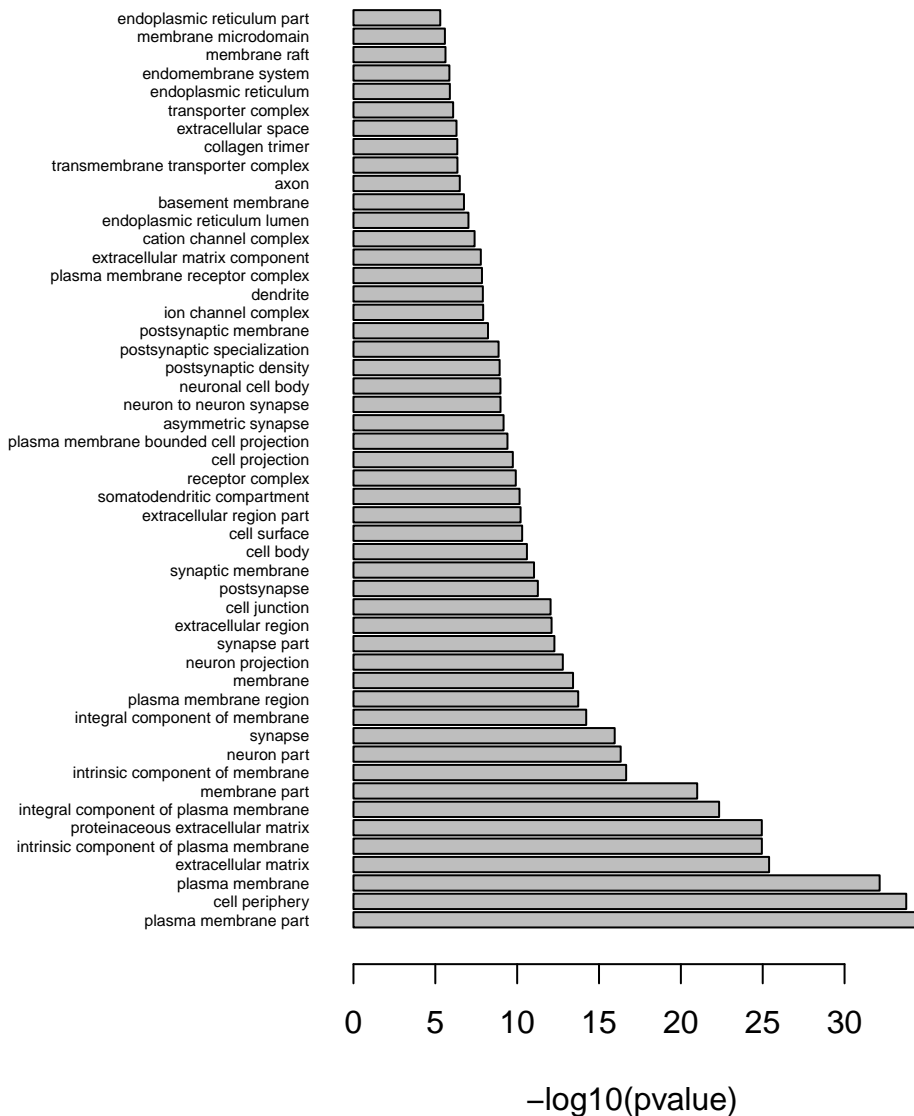

Supplement: DATASET S4 — GO-term analyses for GATA3-expressing and scratched pHAs versus EGFP-expressing and scratched pHAs in 2D cultures. [file Data_Sheet_4.ZIP › GO_term_analyses_GATA3s_vs_GFPs/GOstats/pVal_GOstats_CC_Up_pieChart.pdf]

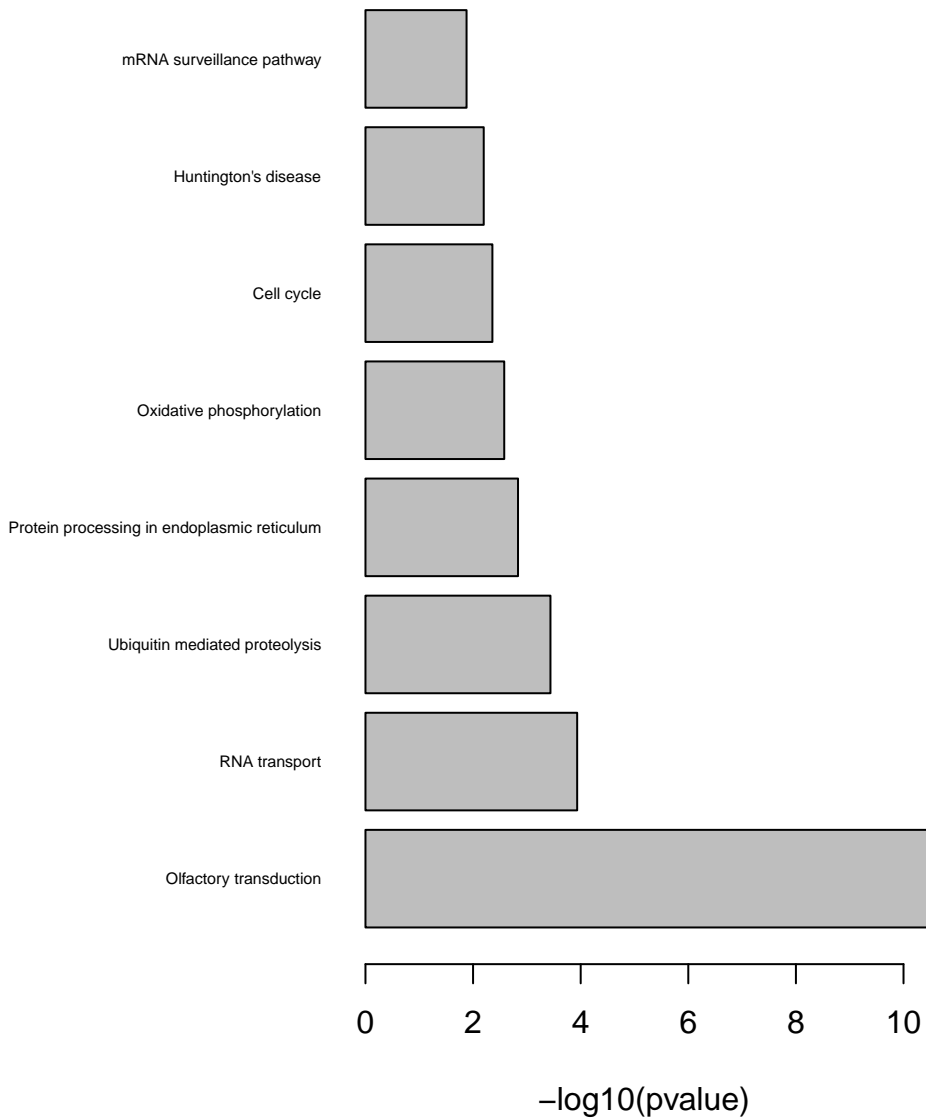

Supplement: DATASET S4 — GO-term analyses for GATA3-expressing and scratched pHAs versus EGFP-expressing and scratched pHAs in 2D cultures. [file Data_Sheet_4.ZIP › GO_term_analyses_GATA3s_vs_GFPs/GOstats/pVal_GOstats_kegg_Under.pdf]

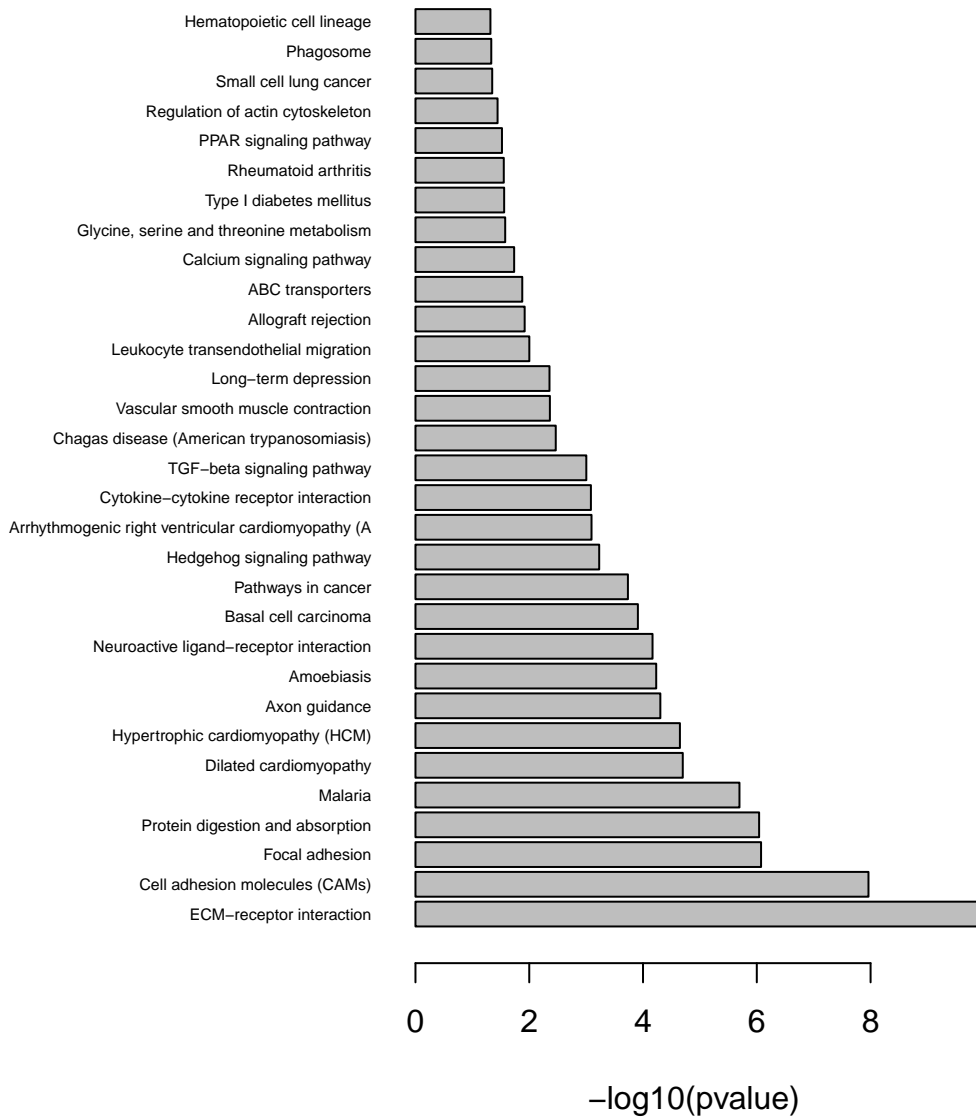

Supplement: DATASET S4 — GO-term analyses for GATA3-expressing and scratched pHAs versus EGFP-expressing and scratched pHAs in 2D cultures. [file Data_Sheet_4.ZIP › GO_term_analyses_GATA3s_vs_GFPs/GOstats/pVal_GOstats_kegg_Up.pdf]

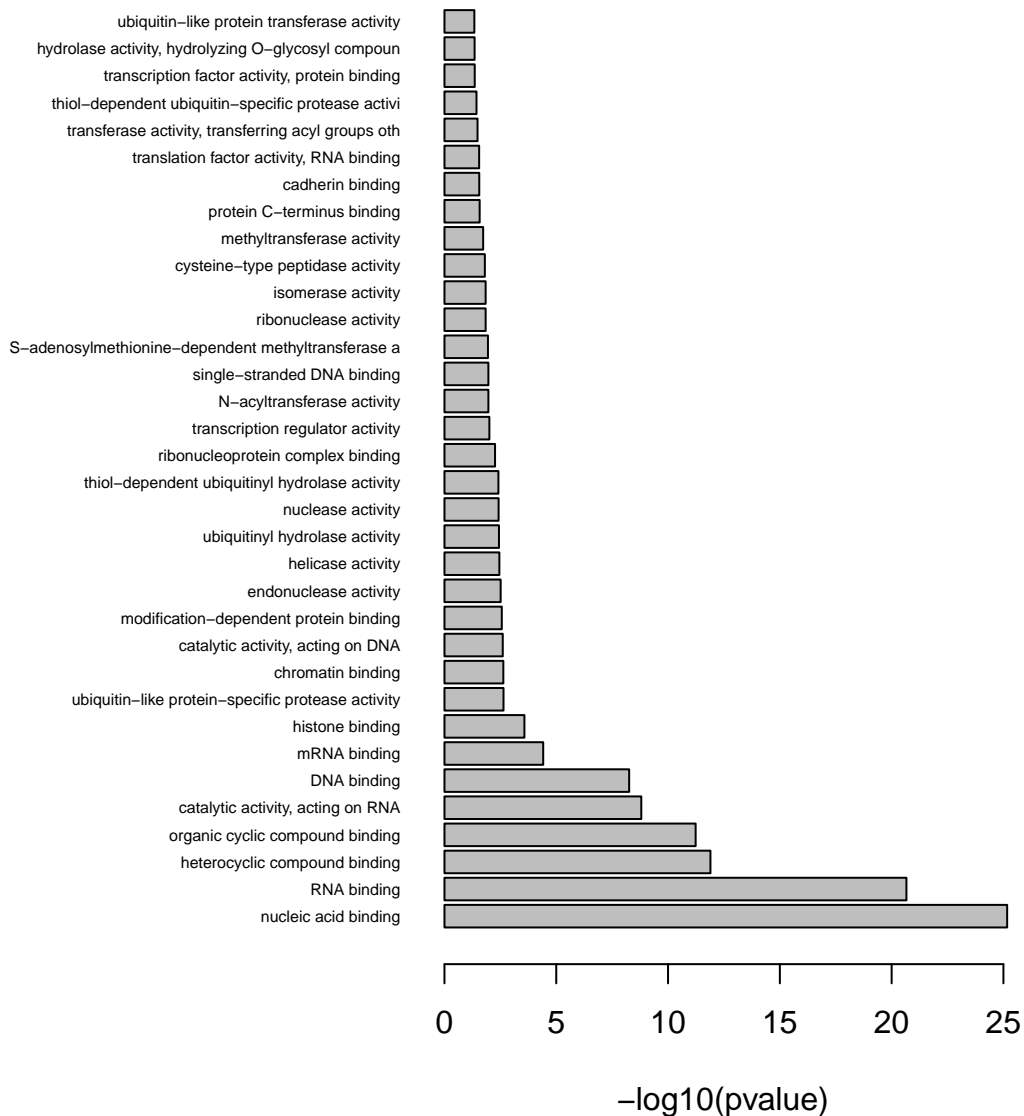

Supplement: DATASET S4 — GO-term analyses for GATA3-expressing and scratched pHAs versus EGFP-expressing and scratched pHAs in 2D cultures. [file Data_Sheet_4.ZIP › GO_term_analyses_GATA3s_vs_GFPs/GOstats/pVal_GOstats_MF_Down_pieChart.pdf]

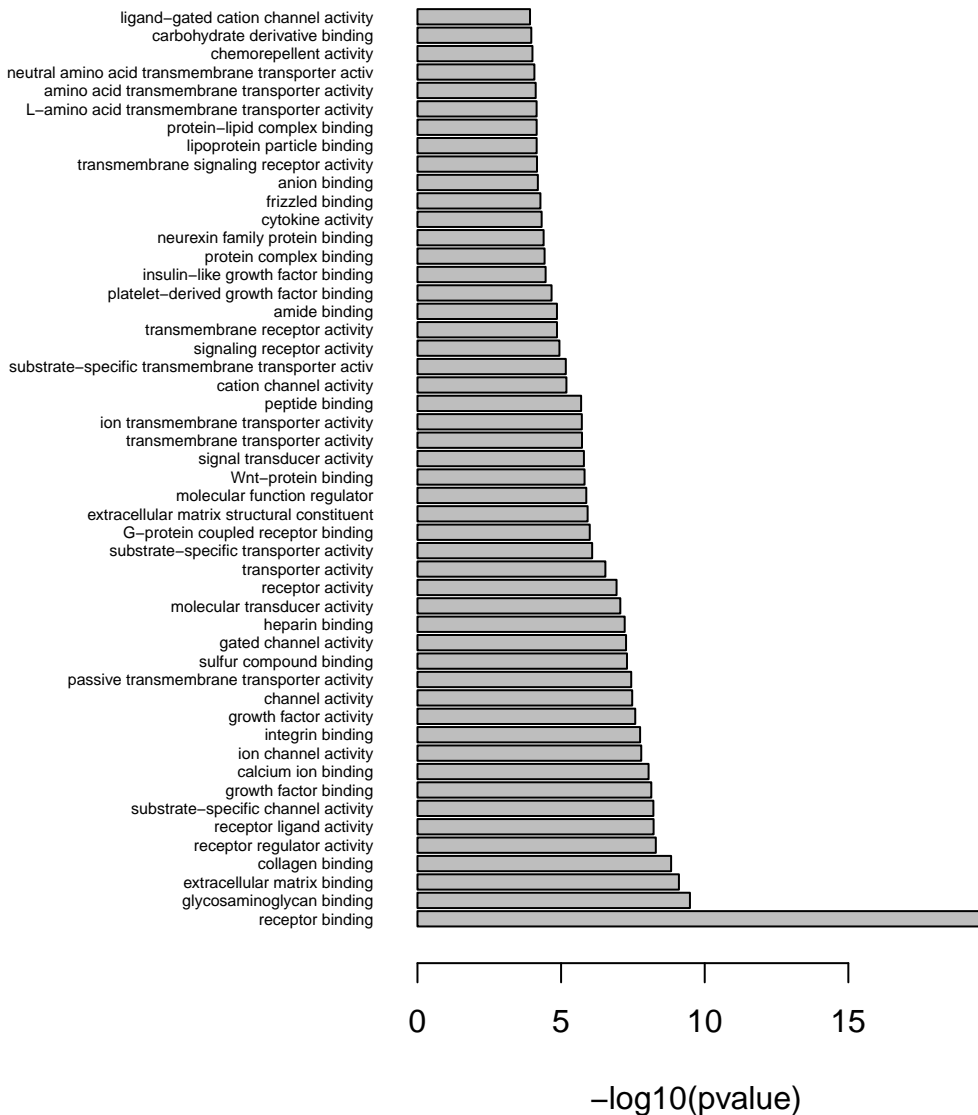

Supplement: DATASET S4 — GO-term analyses for GATA3-expressing and scratched pHAs versus EGFP-expressing and scratched pHAs in 2D cultures. [file Data_Sheet_4.ZIP › GO_term_analyses_GATA3s_vs_GFPs/GOstats/pVal_GOstats_MF_Up_pieChart.pdf]

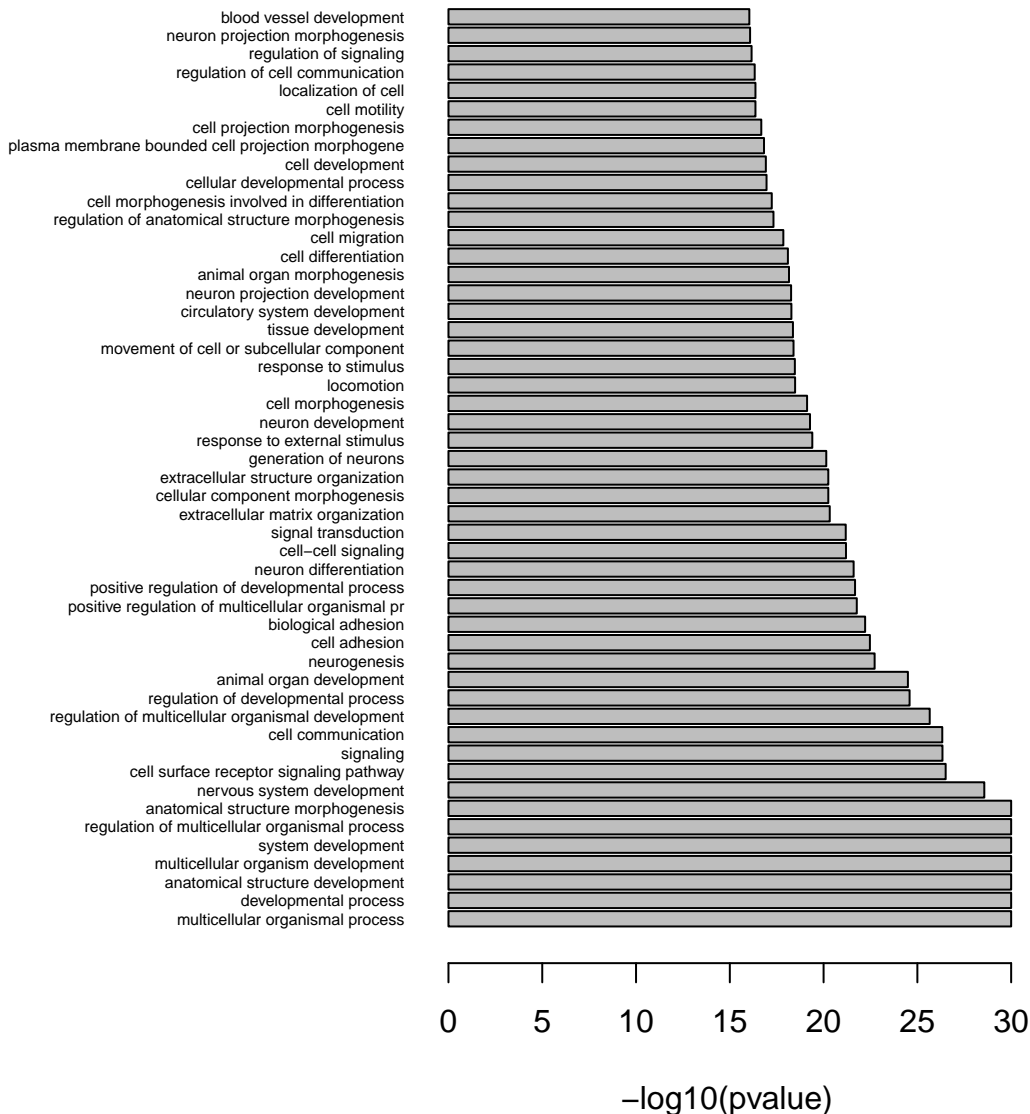

Supplement: DATASET S4 — GO-term analyses for GATA3-expressing and scratched pHAs versus EGFP-expressing and scratched pHAs in 2D cultures. [file Data_Sheet_4.ZIP › GO_term_analyses_GATA3s_vs_GFPs/topGO/pVal_topGO_BP_classicfisher_pieChart.pdf]

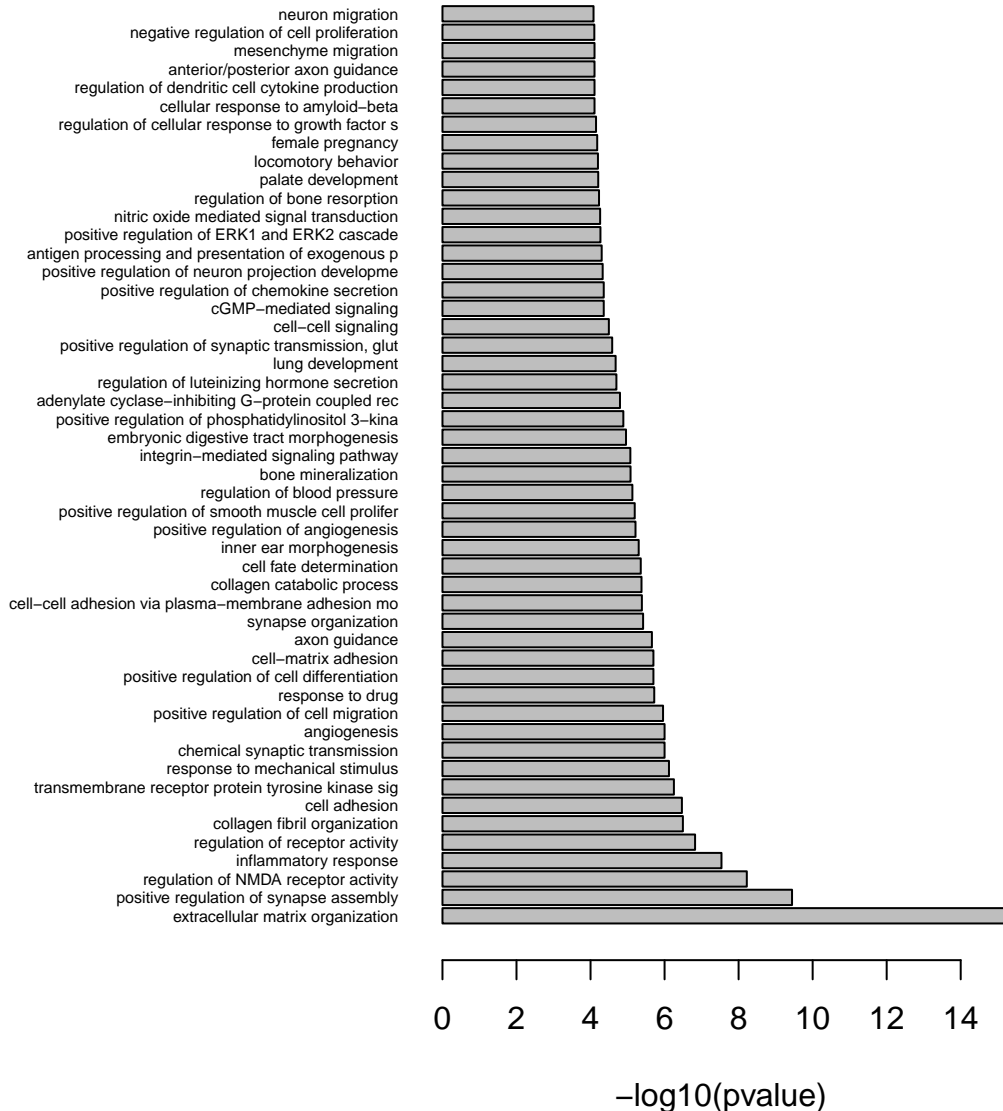

Supplement: DATASET S4 — GO-term analyses for GATA3-expressing and scratched pHAs versus EGFP-expressing and scratched pHAs in 2D cultures. [file Data_Sheet_4.ZIP › GO_term_analyses_GATA3s_vs_GFPs/topGO/pVal_topGO_BP_elimfisher_pieChart.pdf]

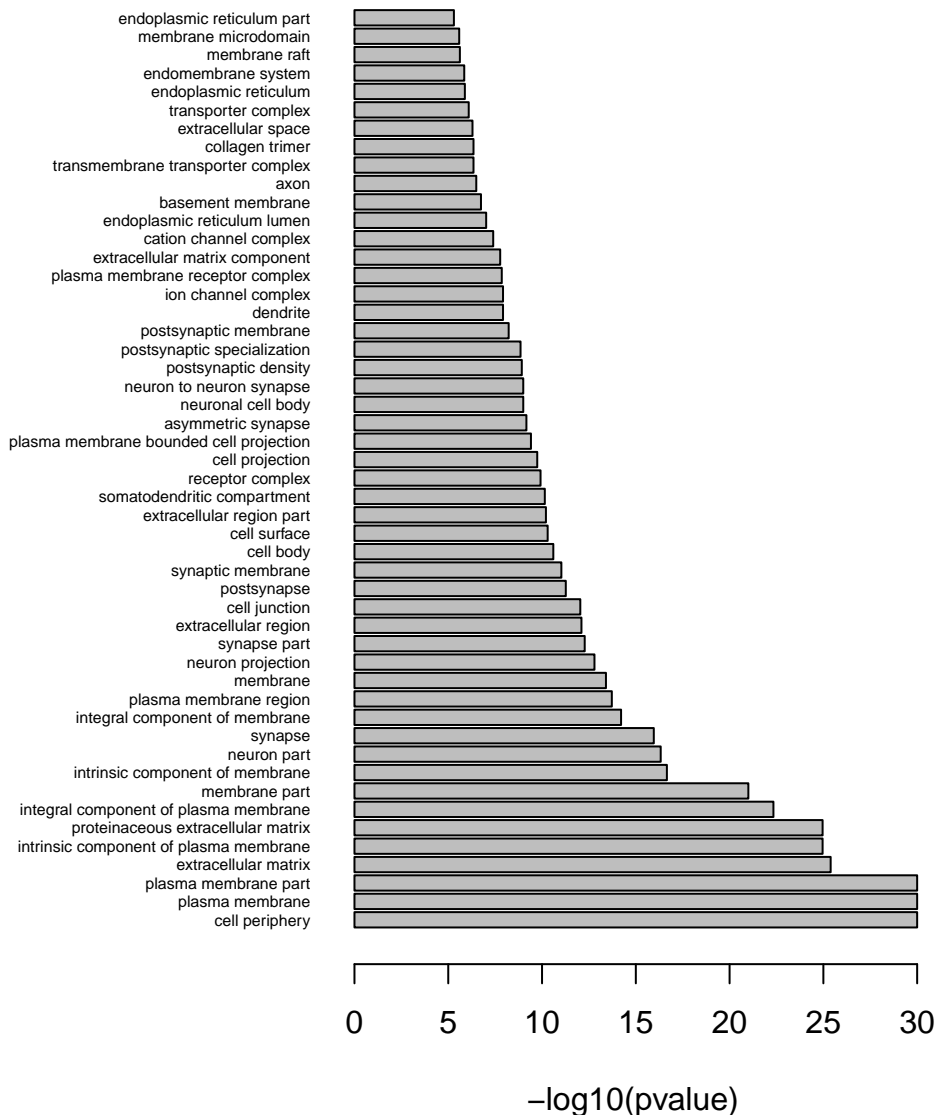

Supplement: DATASET S4 — GO-term analyses for GATA3-expressing and scratched pHAs versus EGFP-expressing and scratched pHAs in 2D cultures. [file Data_Sheet_4.ZIP › GO_term_analyses_GATA3s_vs_GFPs/topGO/pVal_topGO_CC_classicfisher_pieChart.pdf]

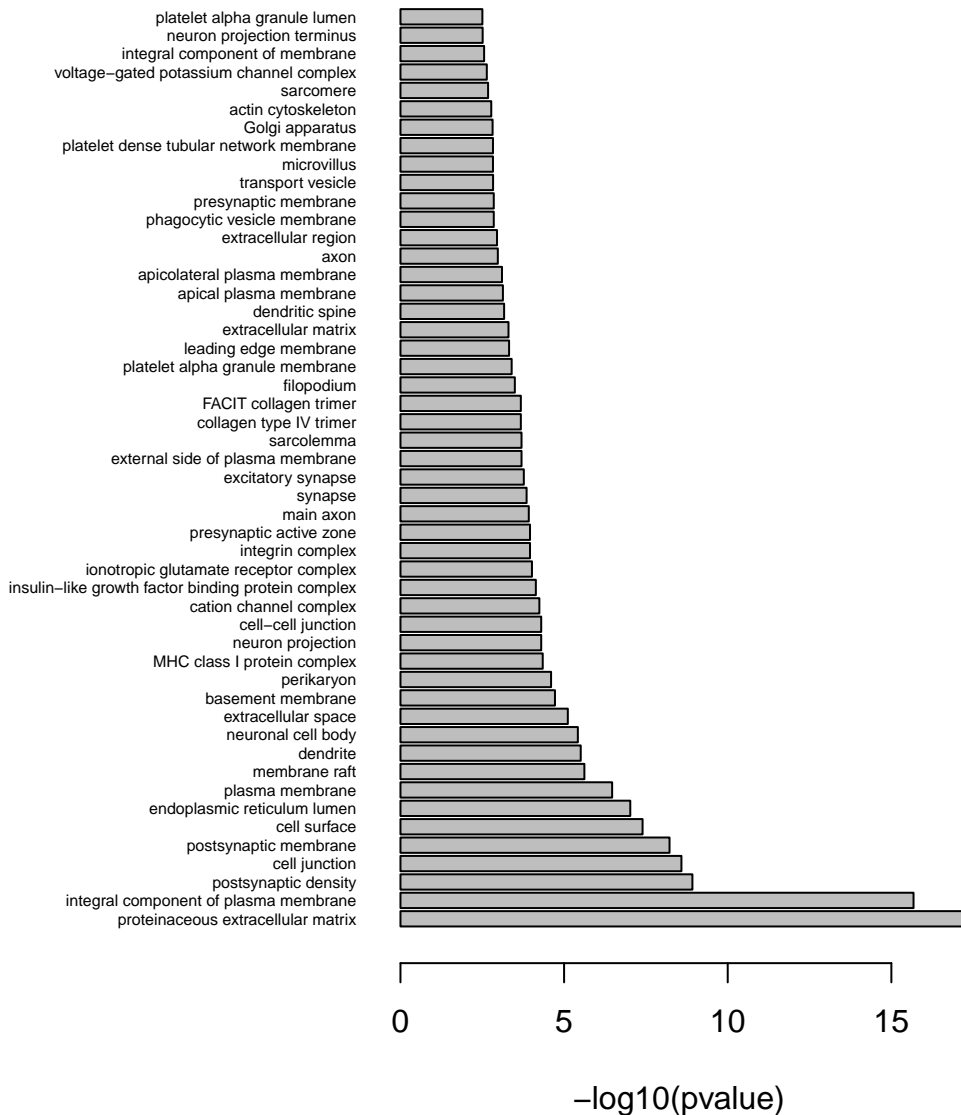

Supplement: DATASET S4 — GO-term analyses for GATA3-expressing and scratched pHAs versus EGFP-expressing and scratched pHAs in 2D cultures. [file Data_Sheet_4.ZIP › GO_term_analyses_GATA3s_vs_GFPs/topGO/pVal_topGO_CC_elimfisher_pieChart.pdf]

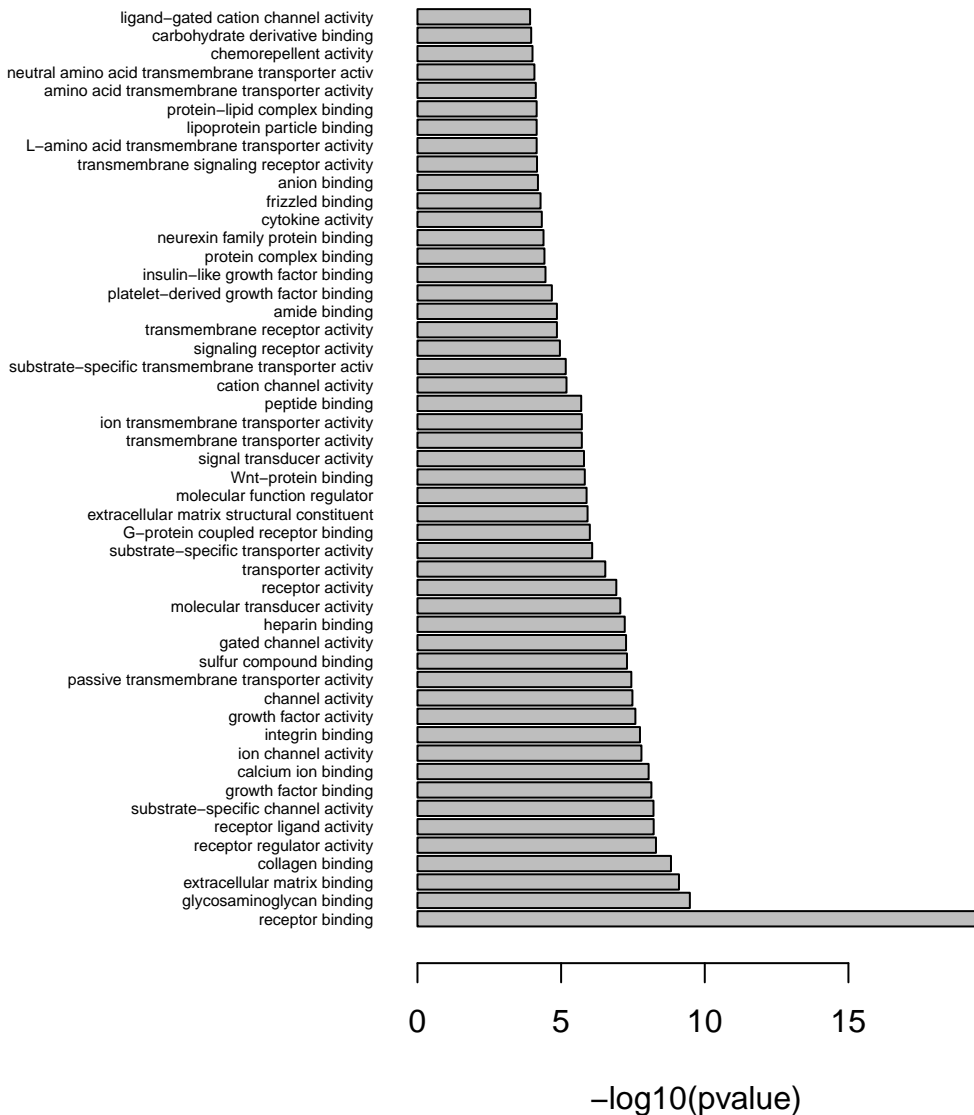

Supplement: DATASET S4 — GO-term analyses for GATA3-expressing and scratched pHAs versus EGFP-expressing and scratched pHAs in 2D cultures. [file Data_Sheet_4.ZIP › GO_term_analyses_GATA3s_vs_GFPs/topGO/pVal_topGO_MF_classicfisher_pieChart.pdf]

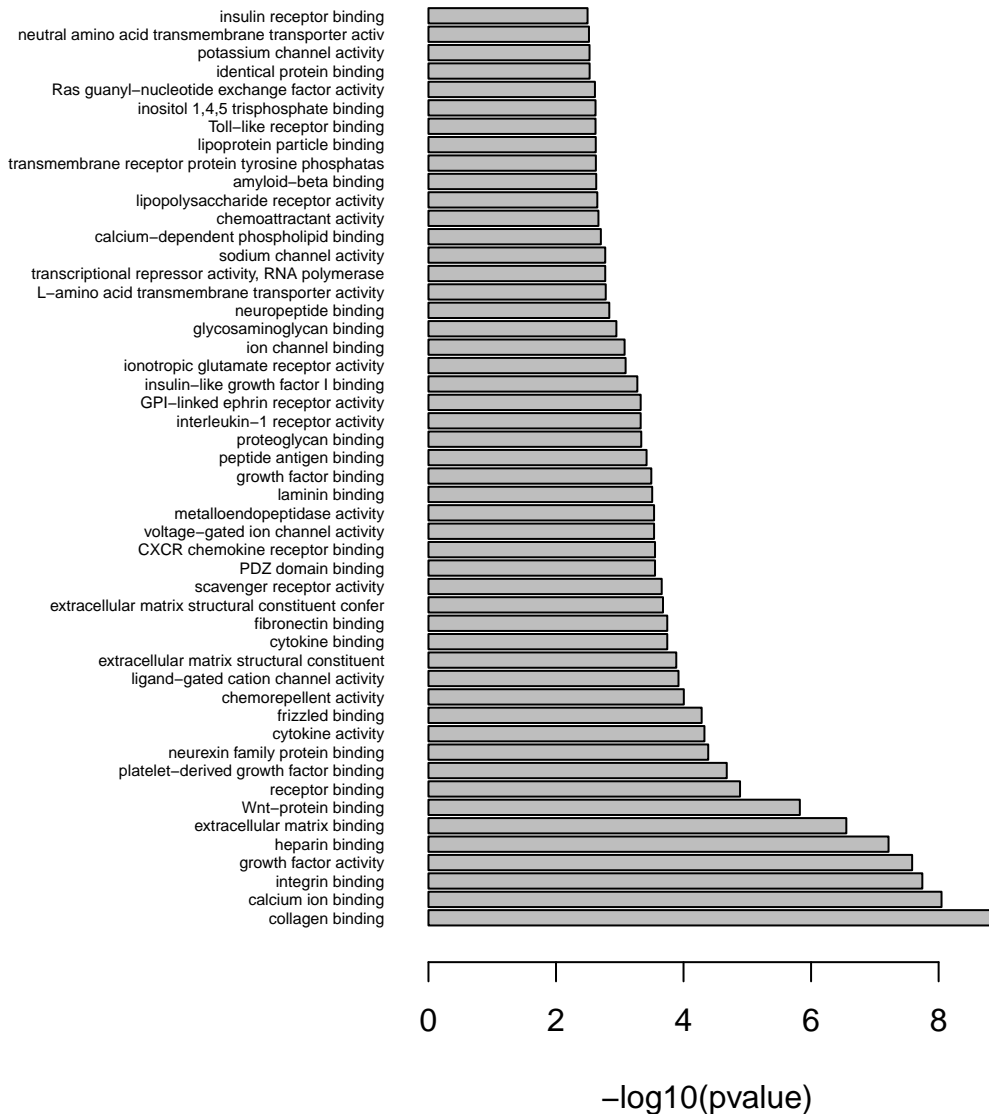

Supplement: DATASET S4 — GO-term analyses for GATA3-expressing and scratched pHAs versus EGFP-expressing and scratched pHAs in 2D cultures. [file Data_Sheet_4.ZIP › GO_term_analyses_GATA3s_vs_GFPs/topGO/pVal_topGO_MF_elimfisher_pieChart.pdf]

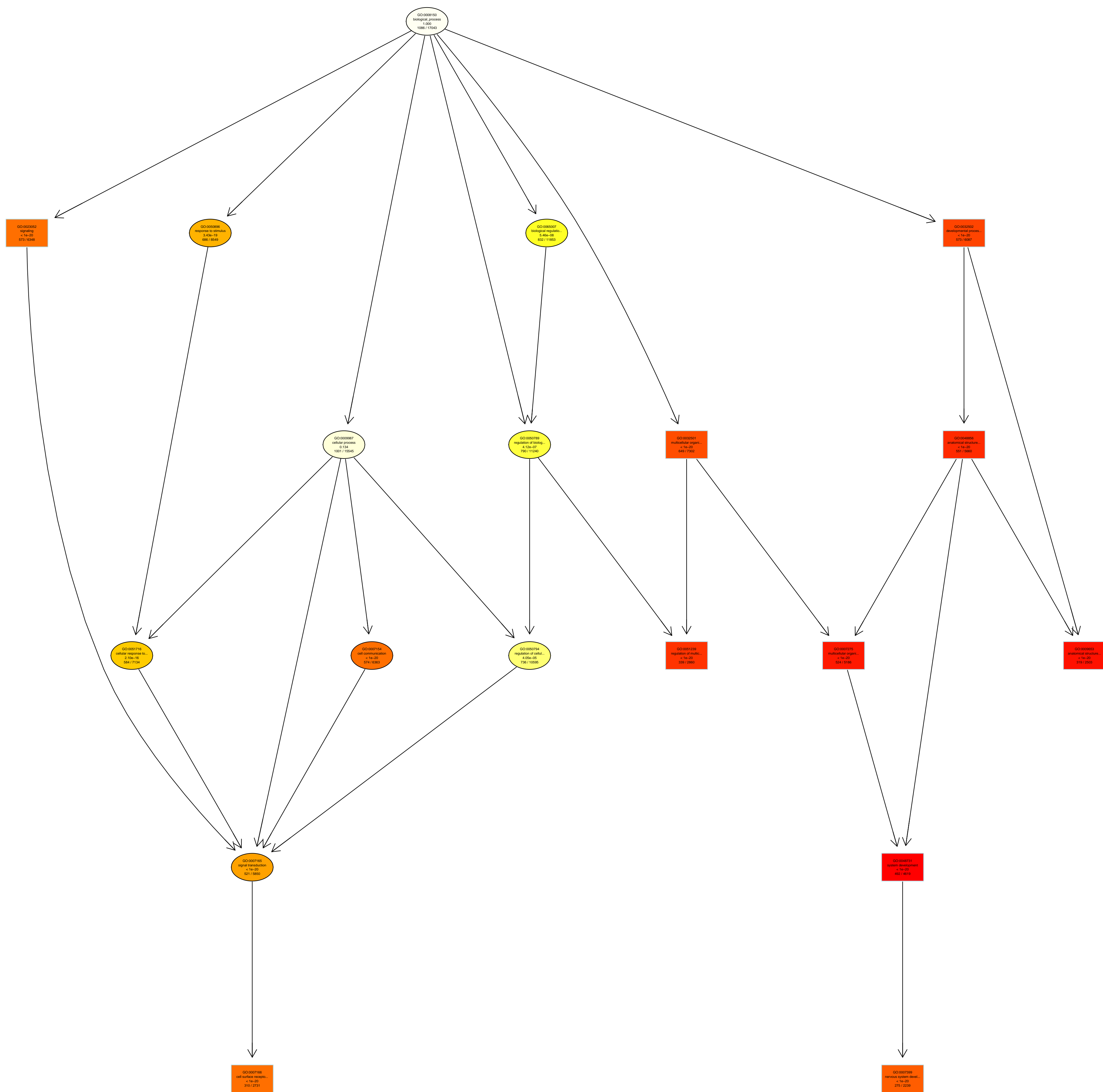

Supplement: DATASET S4 — GO-term analyses for GATA3-expressing and scratched pHAs versus EGFP-expressing and scratched pHAs in 2D cultures. [file Data_Sheet_4.ZIP › GO_term_analyses_GATA3s_vs_GFPs/topGO/topGO_BP_classicfisher_nodes.pdf]

topGO\_BP\_classicfisher\_pieChart

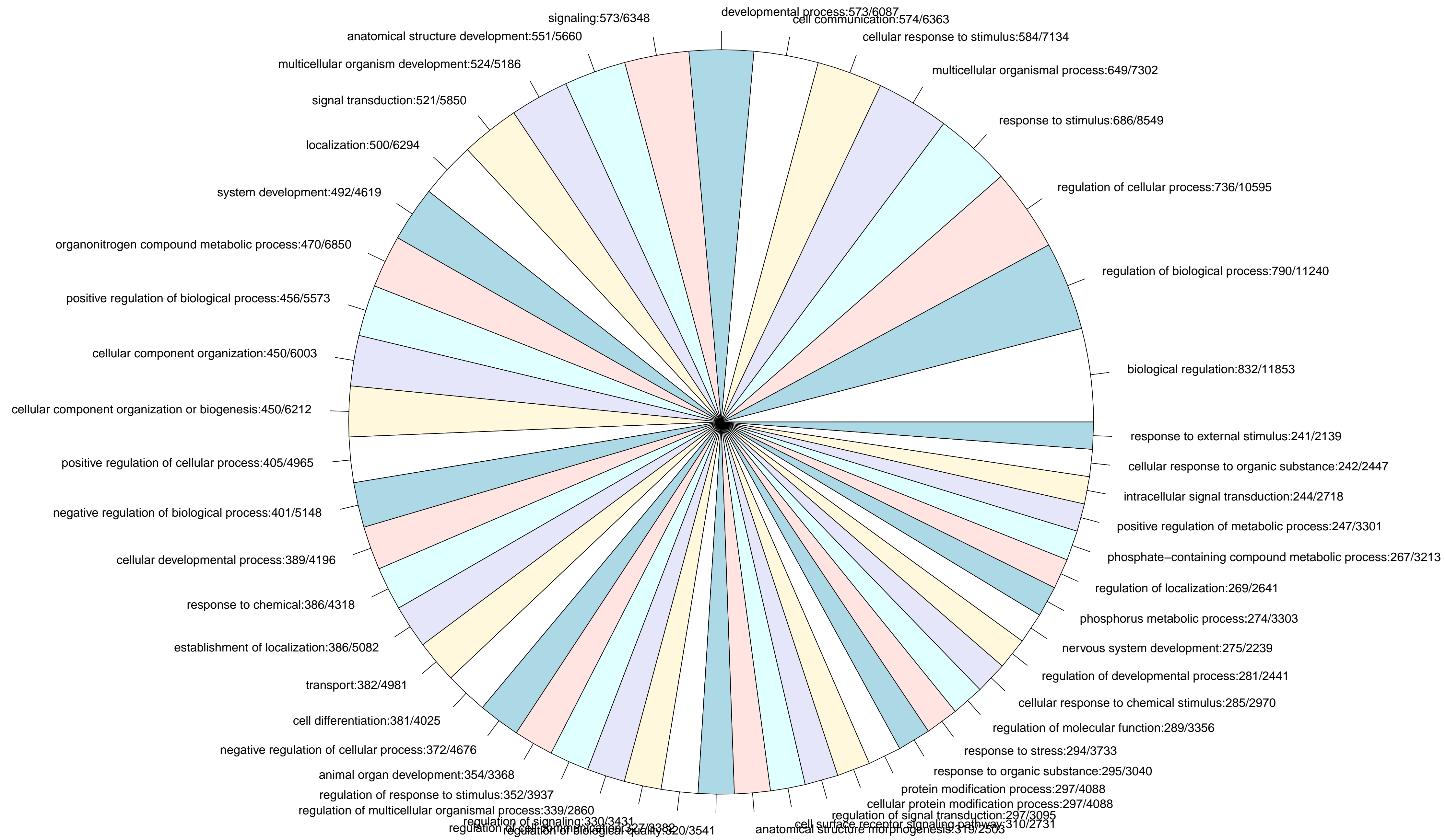

Supplement: DATASET S4 — GO-term analyses for GATA3-expressing and scratched pHAs versus EGFP-expressing and scratched pHAs in 2D cultures. [file Data_Sheet_4.ZIP › GO_term_analyses_GATA3s_vs_GFPs/topGO/topGO_BP_classicfisher_pieChart.pdf]

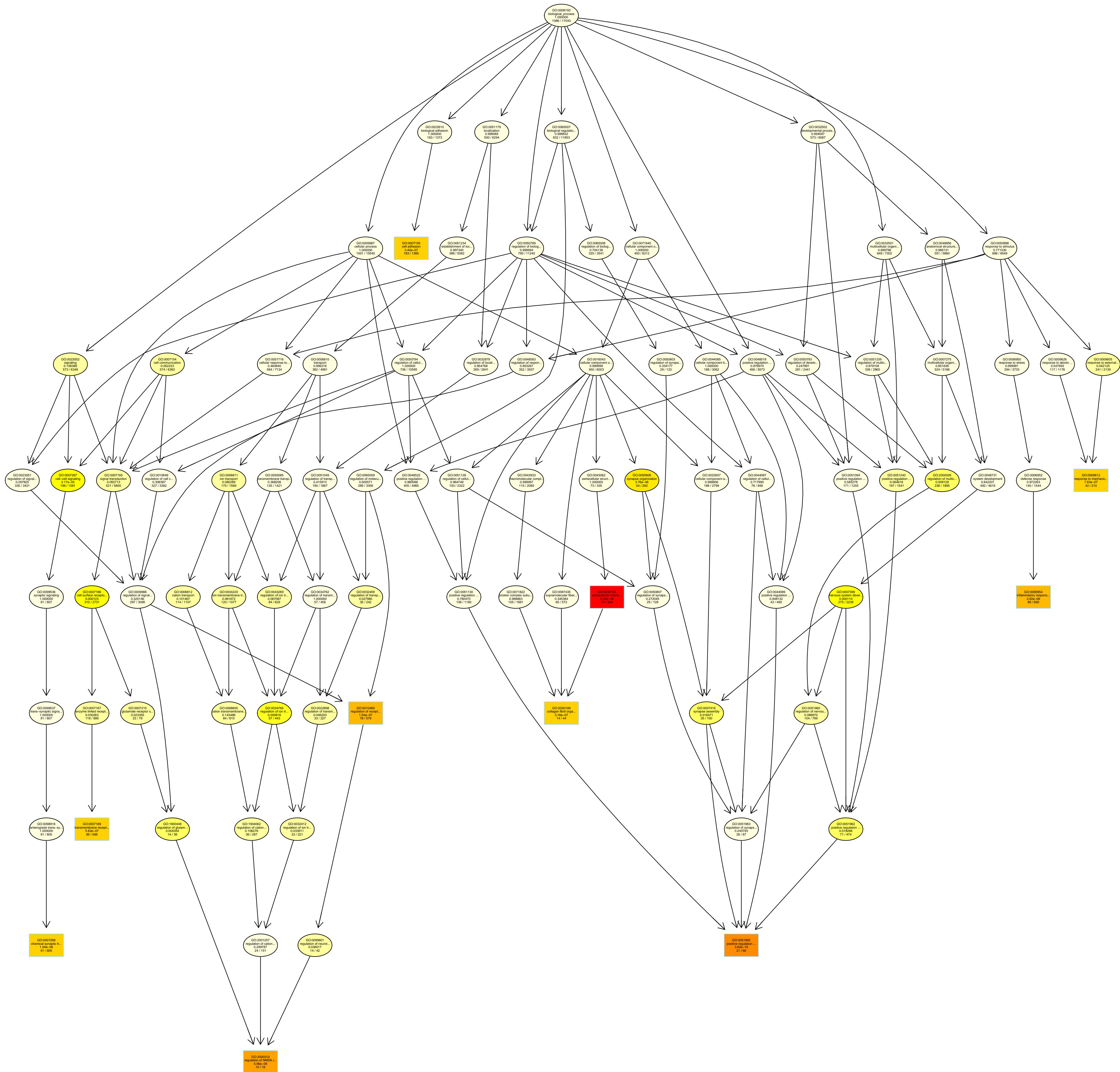

Supplement: DATASET S4 — GO-term analyses for GATA3-expressing and scratched pHAs versus EGFP-expressing and scratched pHAs in 2D cultures. [file Data_Sheet_4.ZIP › GO_term_analyses_GATA3s_vs_GFPs/topGO/topGO_BP_elimfisher_nodes.pdf]
