# Supplementary figures and images for: GATA3 Promotes the Neural Progenitor State but Not Neurogenesis in 3D Traumatic Injury Model of Primary Human Cortical Astrocytes (part 3 of 3)
Source: Front Cell Neurosci. 2019 Feb 11;13:23. doi: 10.3389/fncel.2019.00023 (PMC6380212; doi:10.3389/fncel.2019.00023)

topGO\_BP\_elimfisher\_pieChart

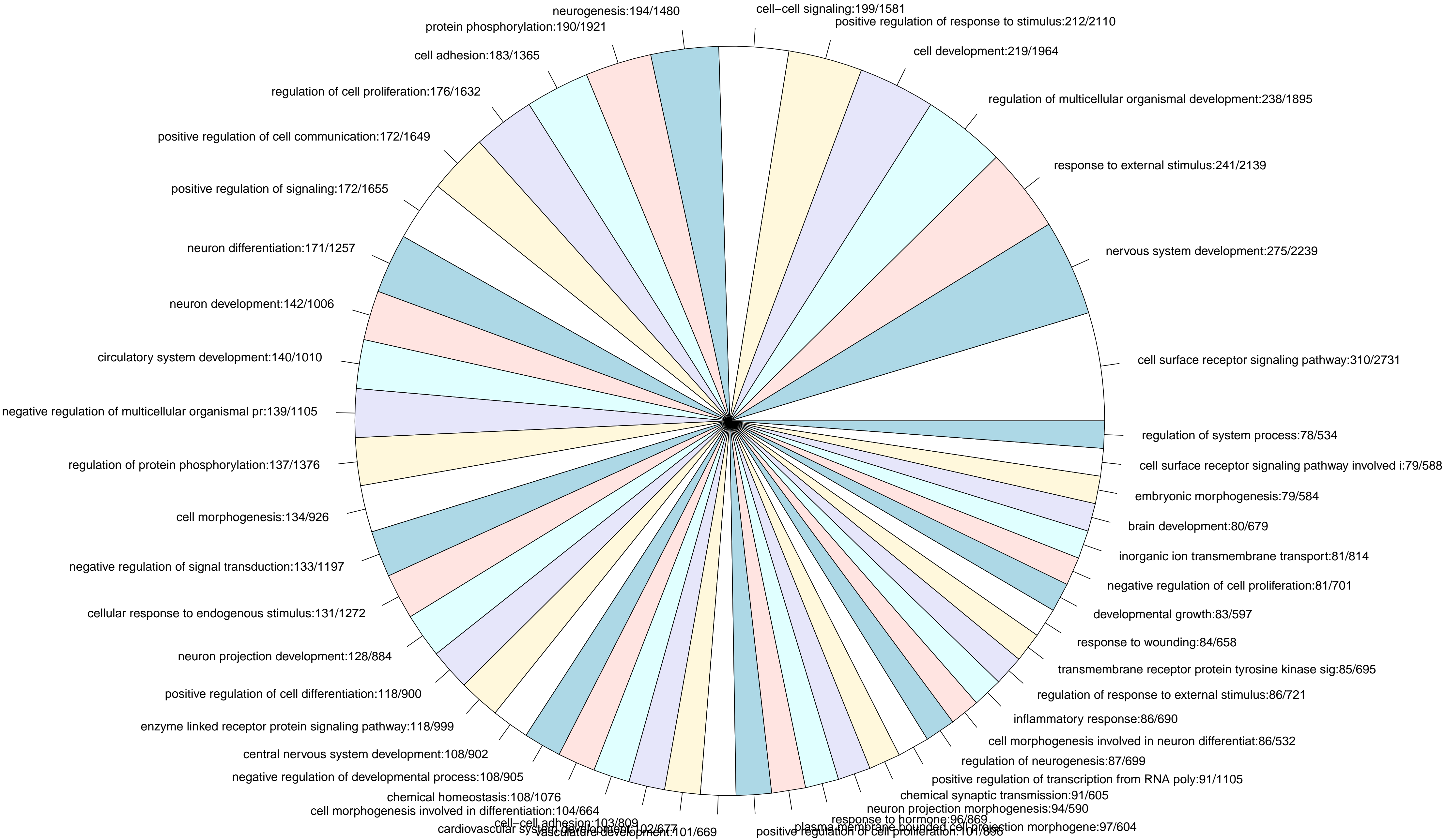

Supplement: DATASET S4 — GO-term analyses for GATA3-expressing and scratched pHAs versus EGFP-expressing and scratched pHAs in 2D cultures. [file Data_Sheet_4.ZIP › GO_term_analyses_GATA3s_vs_GFPs/topGO/topGO_BP_elimfisher_pieChart.pdf]

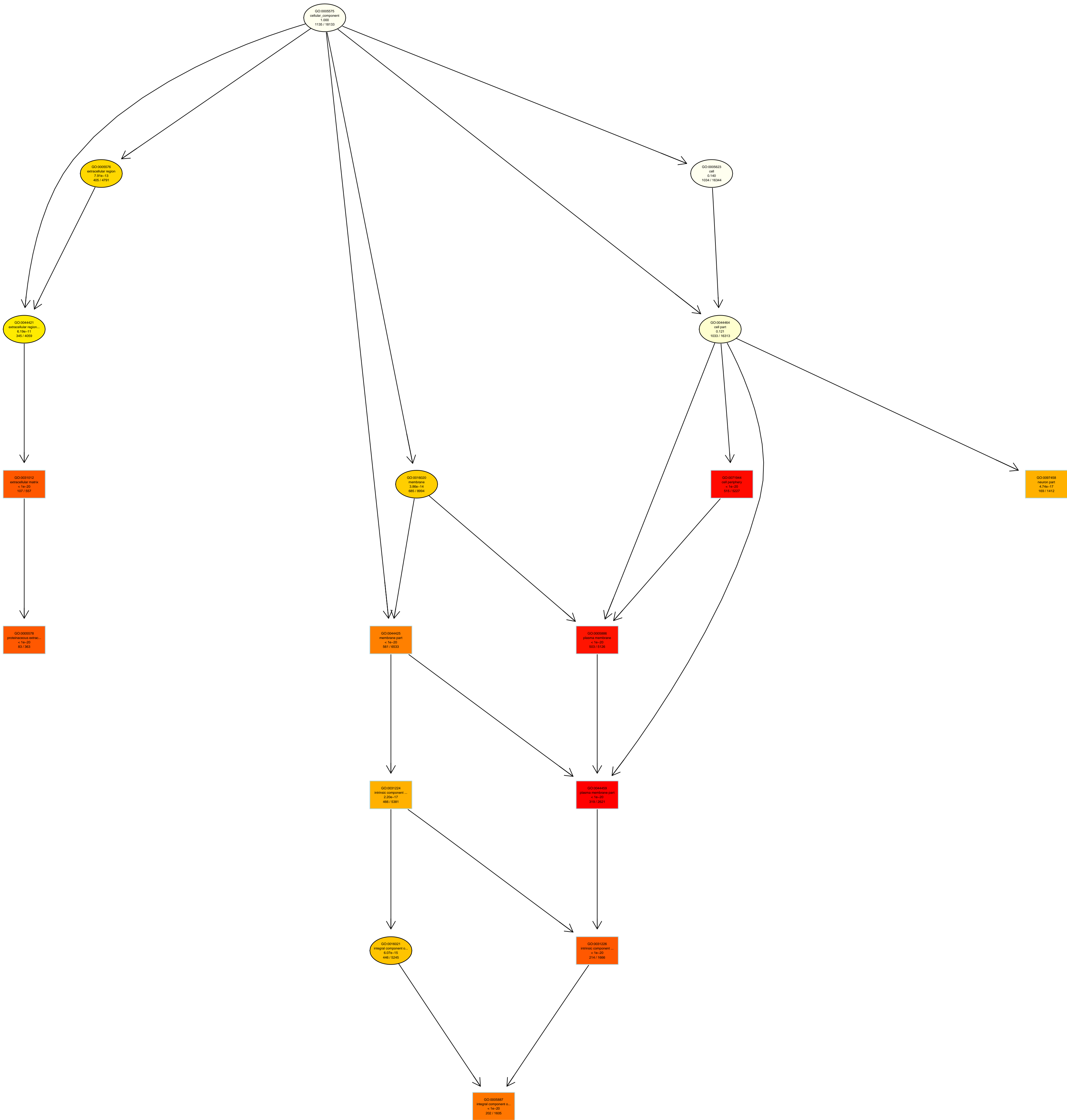

Supplement: DATASET S4 — GO-term analyses for GATA3-expressing and scratched pHAs versus EGFP-expressing and scratched pHAs in 2D cultures. [file Data_Sheet_4.ZIP › GO_term_analyses_GATA3s_vs_GFPs/topGO/topGO_CC_classicfisher_nodes.pdf]

topGO\_CC\_classicfisher\_pieChart

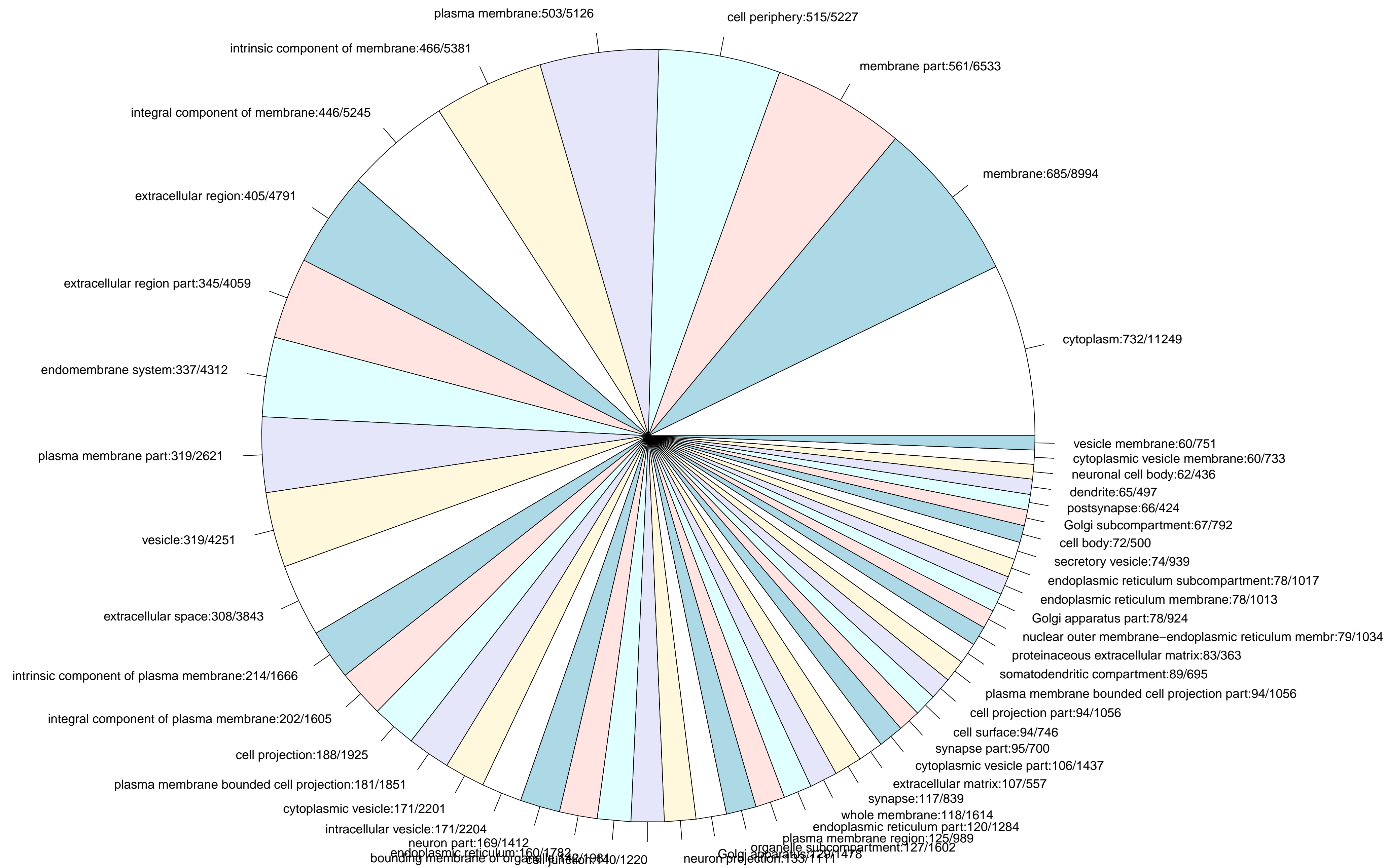

Supplement: DATASET S4 — GO-term analyses for GATA3-expressing and scratched pHAs versus EGFP-expressing and scratched pHAs in 2D cultures. [file Data_Sheet_4.ZIP › GO_term_analyses_GATA3s_vs_GFPs/topGO/topGO_CC_classicfisher_pieChart.pdf]

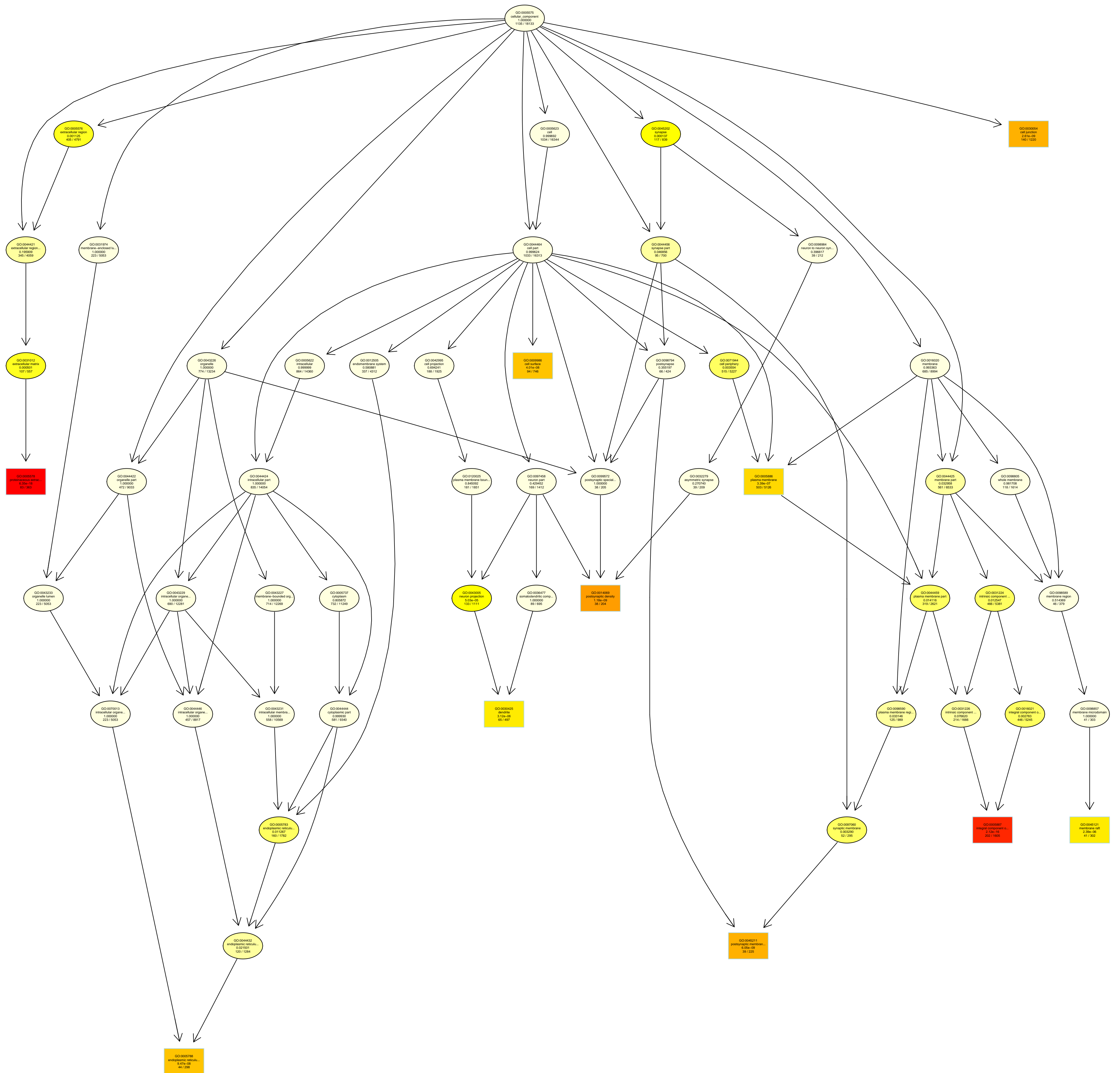

Supplement: DATASET S4 — GO-term analyses for GATA3-expressing and scratched pHAs versus EGFP-expressing and scratched pHAs in 2D cultures. [file Data_Sheet_4.ZIP › GO_term_analyses_GATA3s_vs_GFPs/topGO/topGO_CC_elimfisher_nodes.pdf]

topGO\_CC\_elimfisher\_pieChart

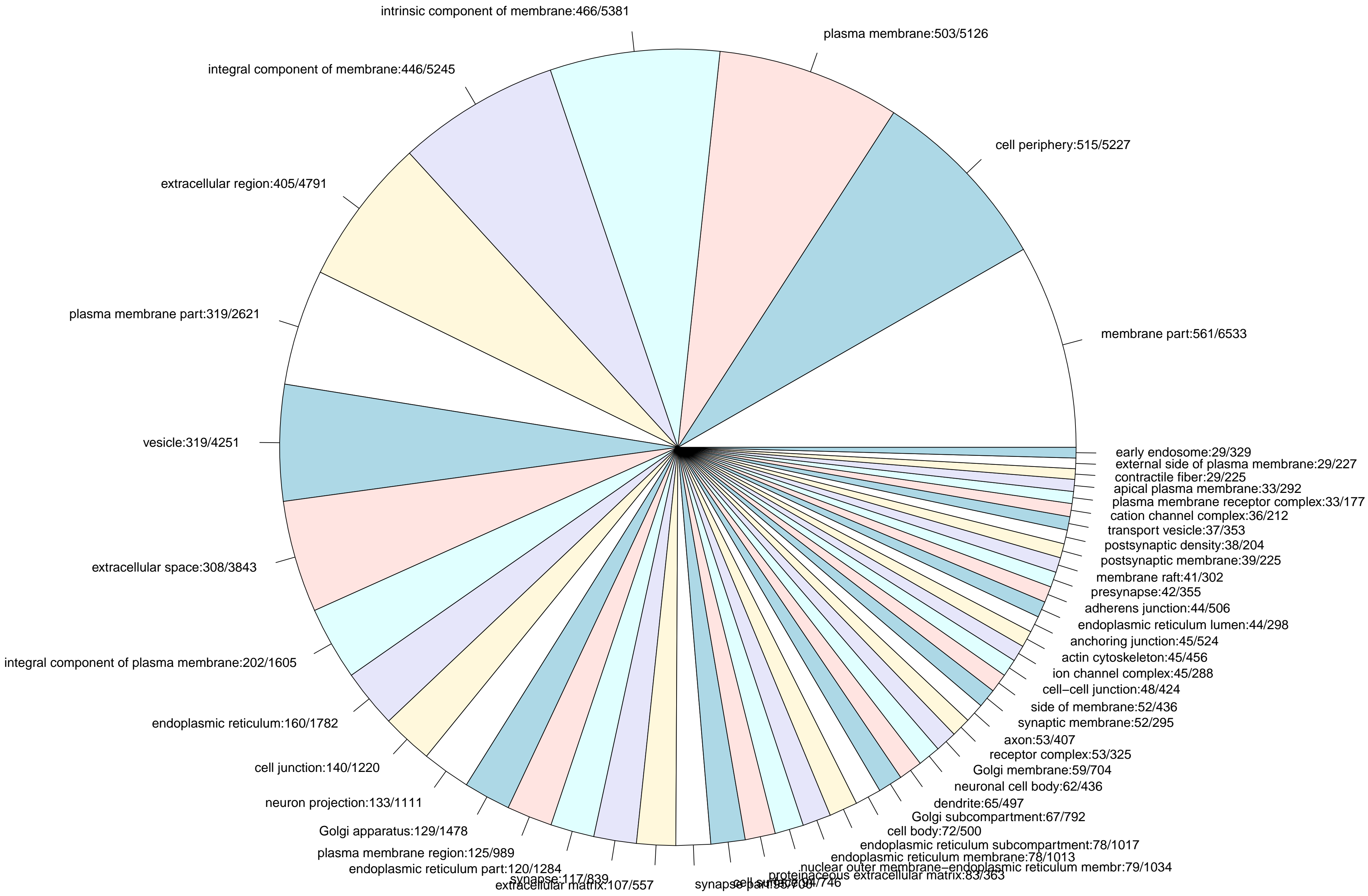

Supplement: DATASET S4 — GO-term analyses for GATA3-expressing and scratched pHAs versus EGFP-expressing and scratched pHAs in 2D cultures. [file Data_Sheet_4.ZIP › GO_term_analyses_GATA3s_vs_GFPs/topGO/topGO_CC_elimfisher_pieChart.pdf]

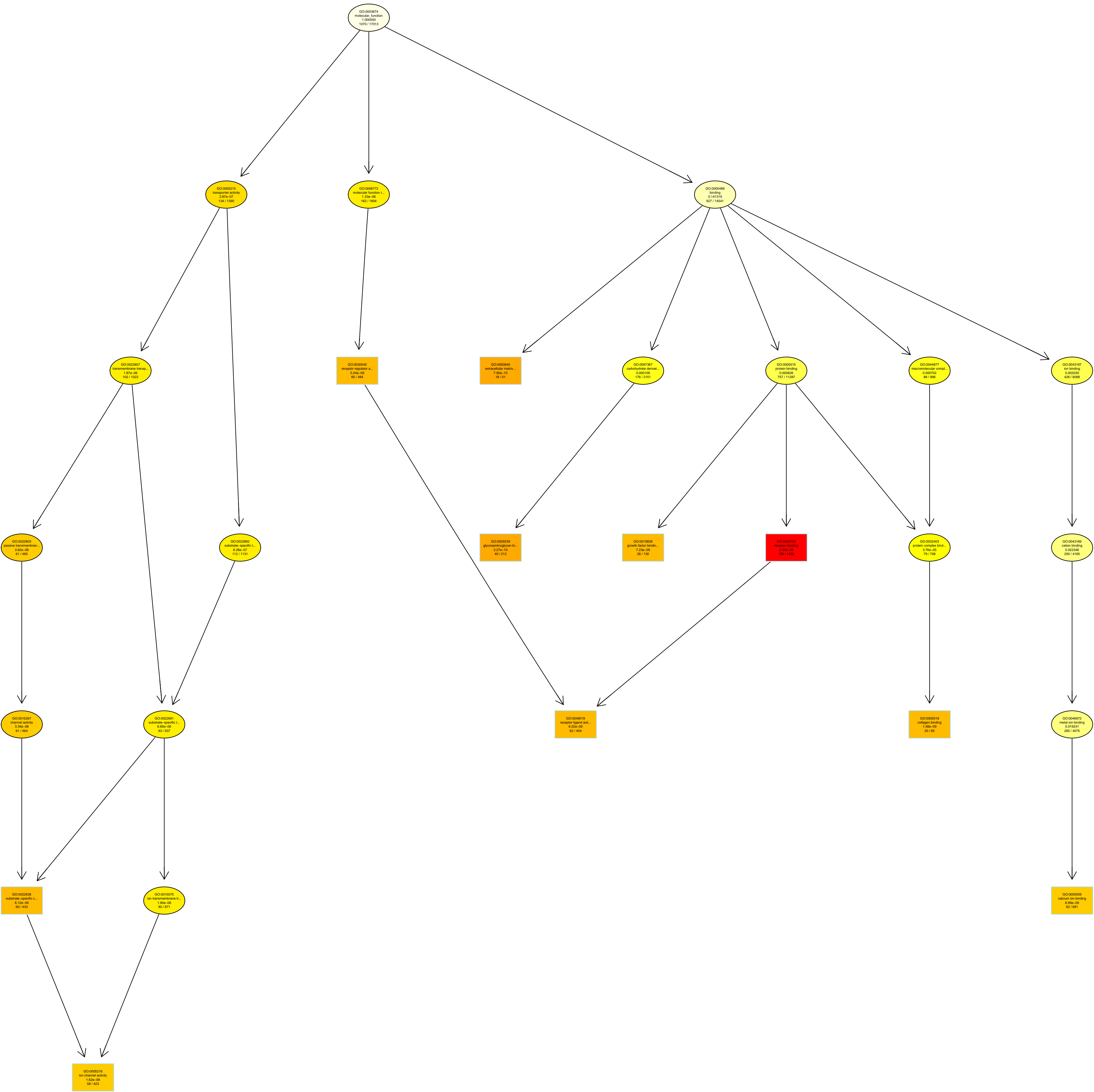

Supplement: DATASET S4 — GO-term analyses for GATA3-expressing and scratched pHAs versus EGFP-expressing and scratched pHAs in 2D cultures. [file Data_Sheet_4.ZIP › GO_term_analyses_GATA3s_vs_GFPs/topGO/topGO_MF_classicfisher_nodes.pdf]

topGO\_MF\_classicfisher\_pieChart

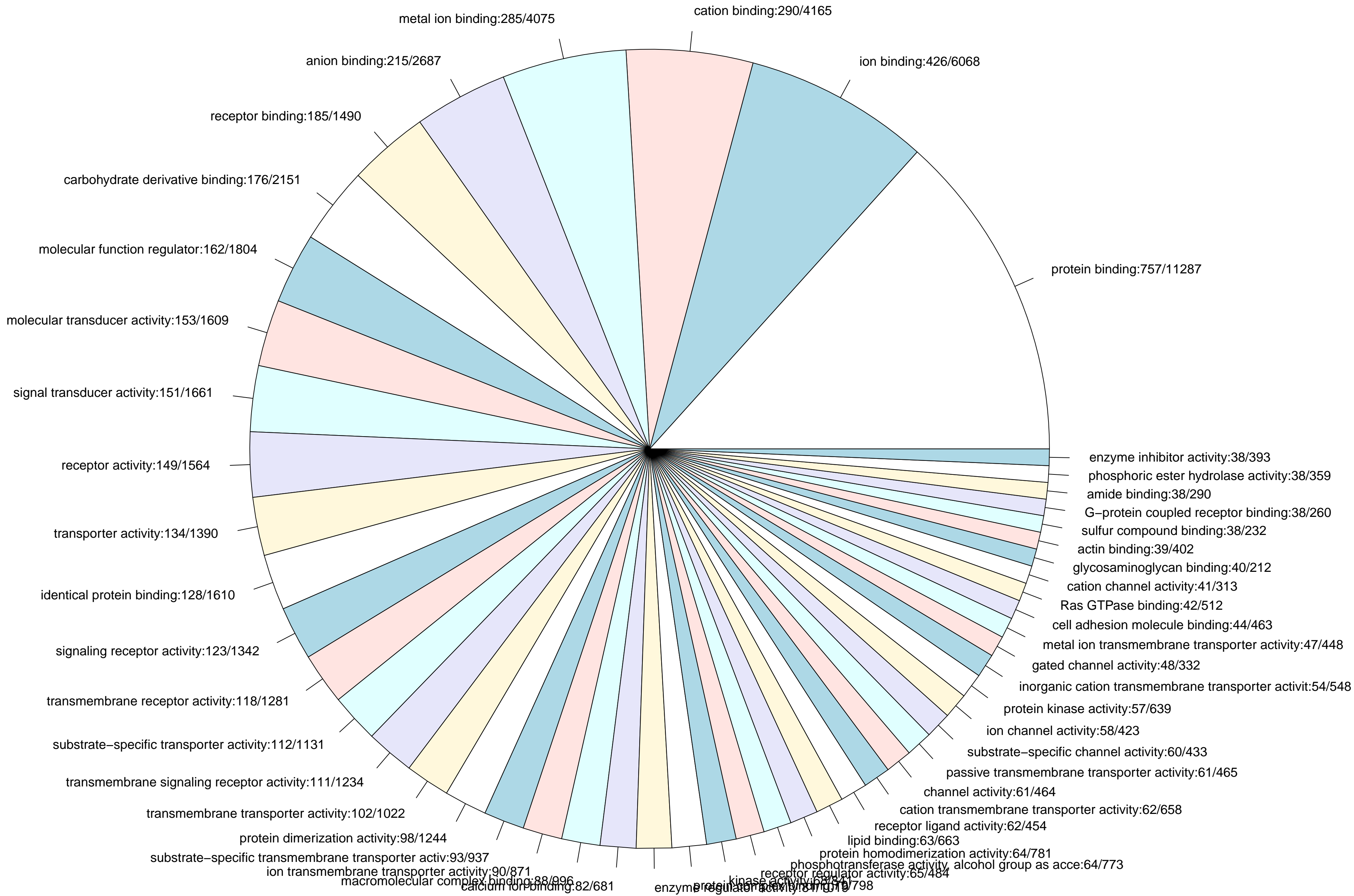

Supplement: DATASET S4 — GO-term analyses for GATA3-expressing and scratched pHAs versus EGFP-expressing and scratched pHAs in 2D cultures. [file Data_Sheet_4.ZIP › GO_term_analyses_GATA3s_vs_GFPs/topGO/topGO_MF_classicfisher_pieChart.pdf]

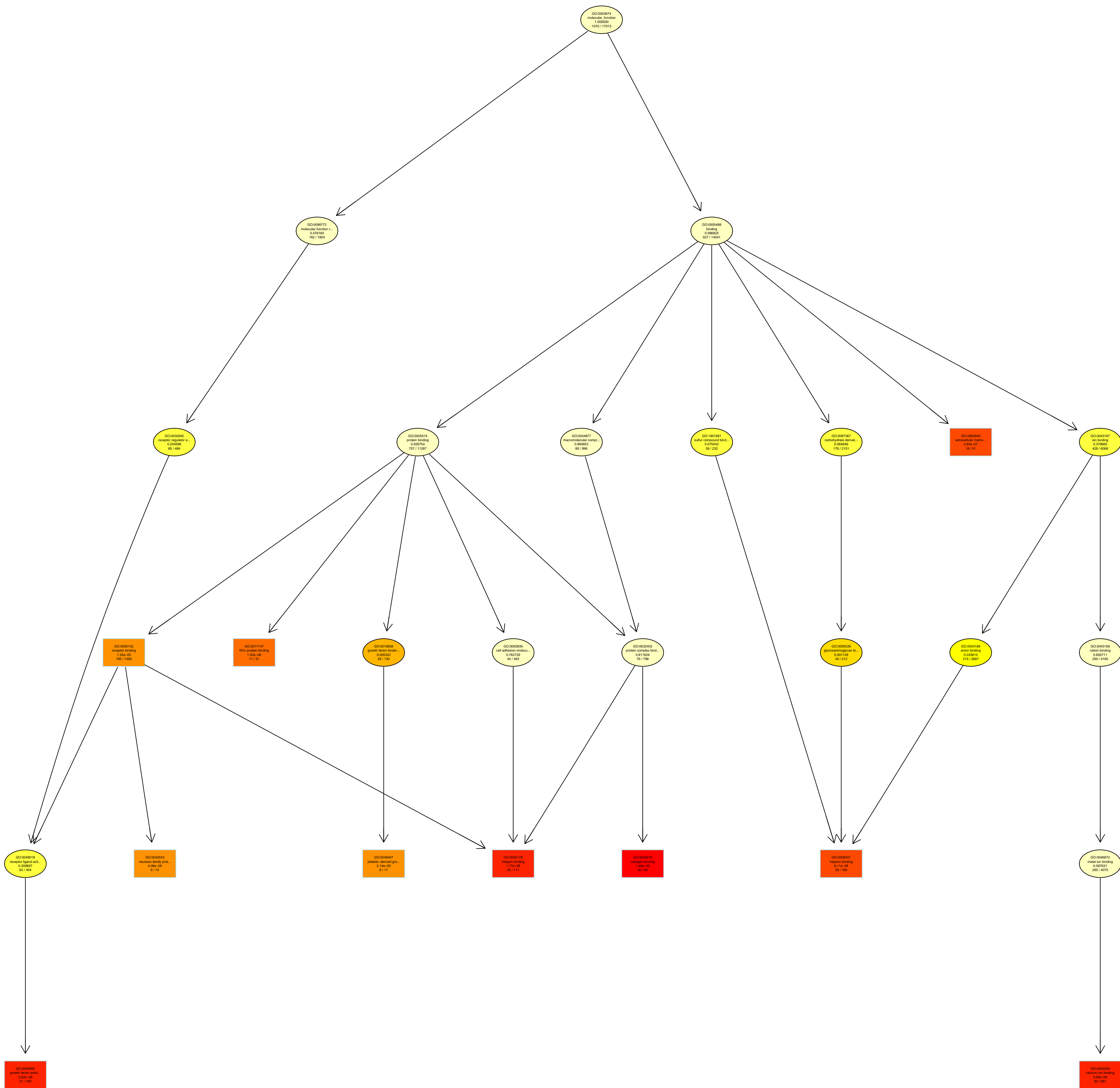

Supplement: DATASET S4 — GO-term analyses for GATA3-expressing and scratched pHAs versus EGFP-expressing and scratched pHAs in 2D cultures. [file Data_Sheet_4.ZIP › GO_term_analyses_GATA3s_vs_GFPs/topGO/topGO_MF_elimfisher_nodes.pdf]

topGO\_MF\_elimfisher\_pieChart

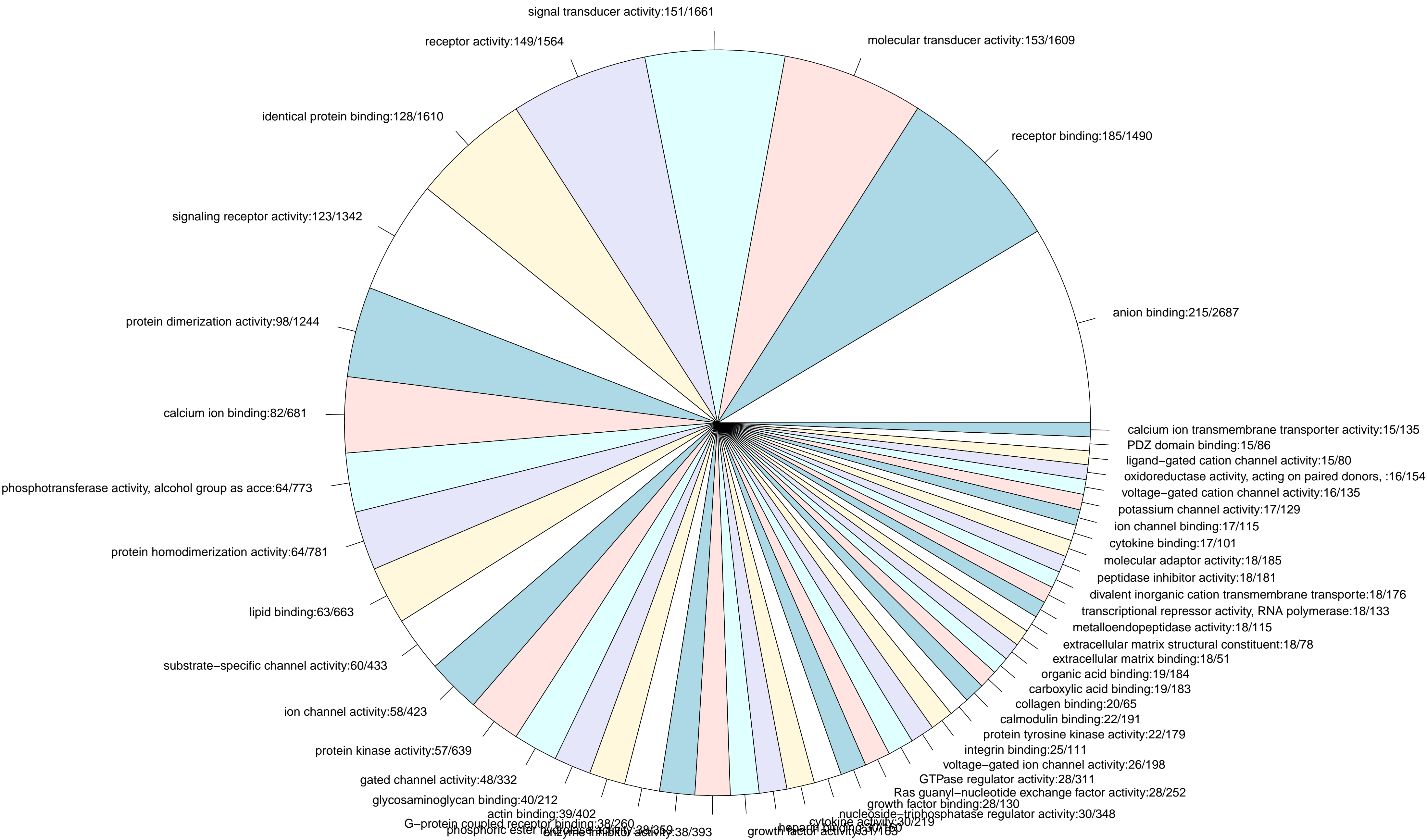

Supplement: DATASET S4 — GO-term analyses for GATA3-expressing and scratched pHAs versus EGFP-expressing and scratched pHAs in 2D cultures. [file Data_Sheet_4.ZIP › GO_term_analyses_GATA3s_vs_GFPs/topGO/topGO_MF_elimfisher_pieChart.pdf]

# Gata3\_LN\_vs\_Gata3\_NoS

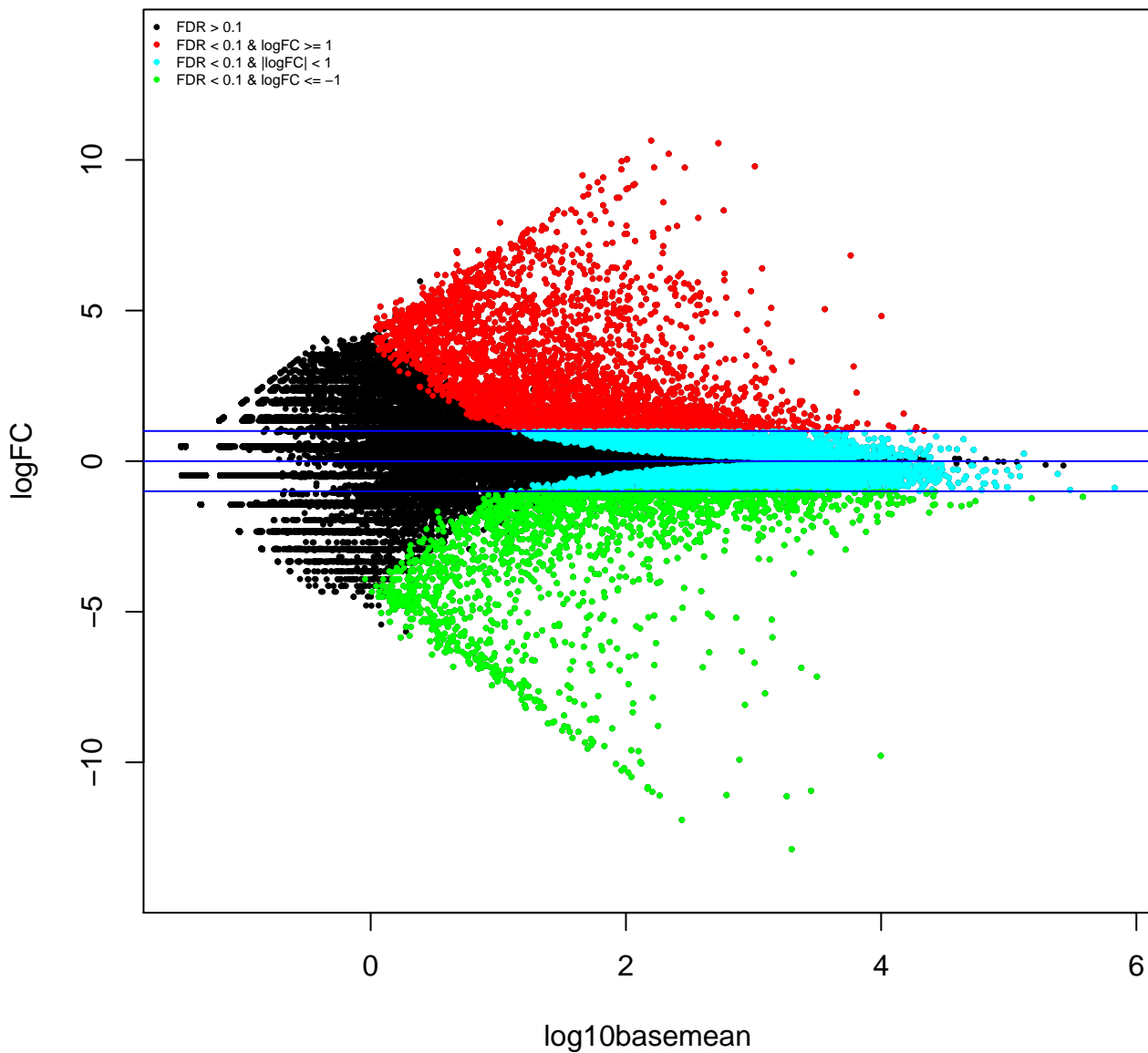

Supplement: DATASET S5 — GO-term analyses of control cultures (EGFP-expressing and no injury) in 3D versus 2D. [file Data_Sheet_5.ZIP › SD5_3D_vs_2D/Gata3_LN_vs_Gata3_NoS_MAplot.pdf]

# Gata3\_LN\_vs\_Gata3\_NoS

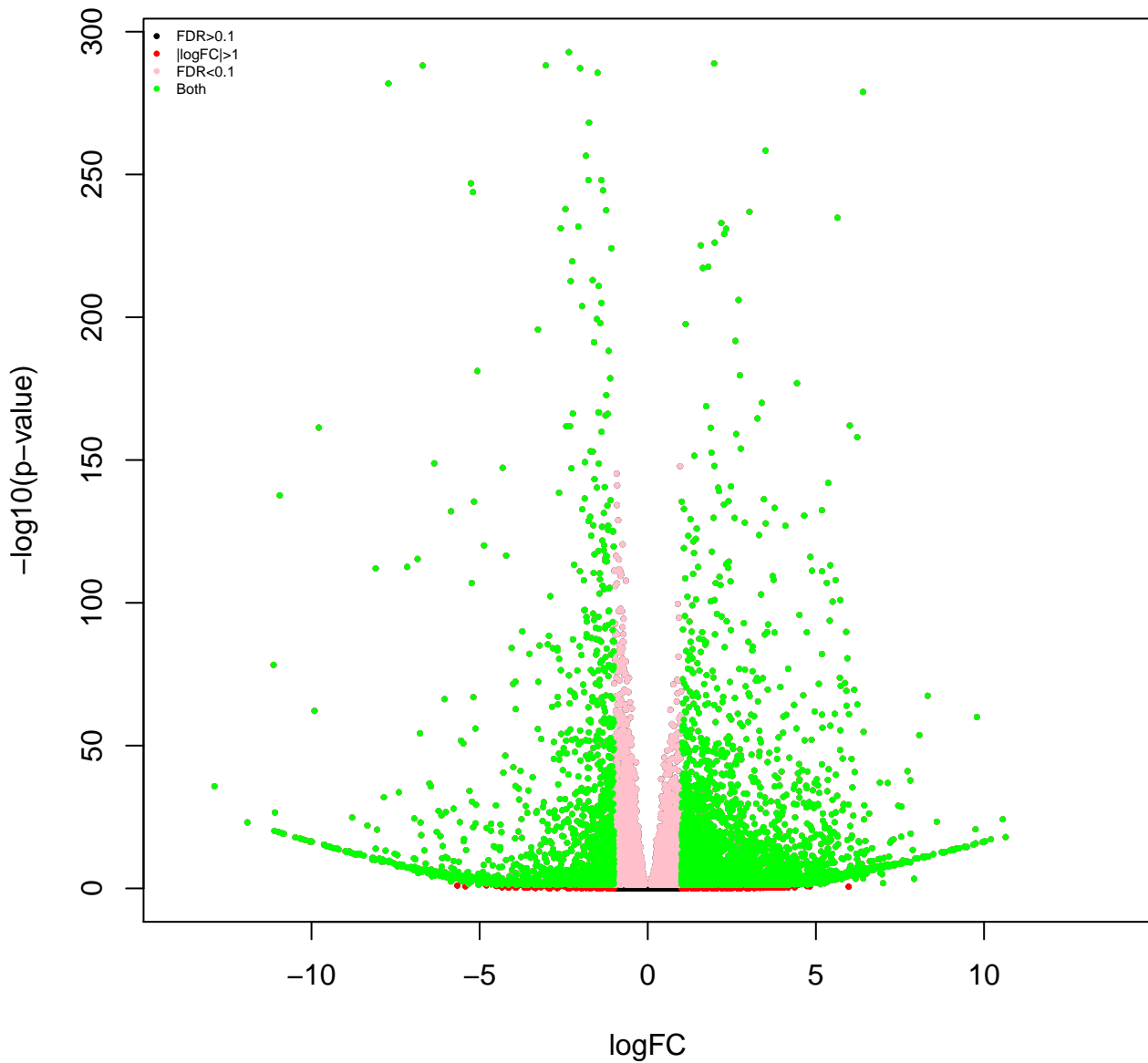

Supplement: DATASET S5 — GO-term analyses of control cultures (EGFP-expressing and no injury) in 3D versus 2D. [file Data_Sheet_5.ZIP › SD5_3D_vs_2D/Gata3_LN_vs_Gata3_NoS_Volcanoplot.pdf]

GOstats\_BP\_Down\_pieChart

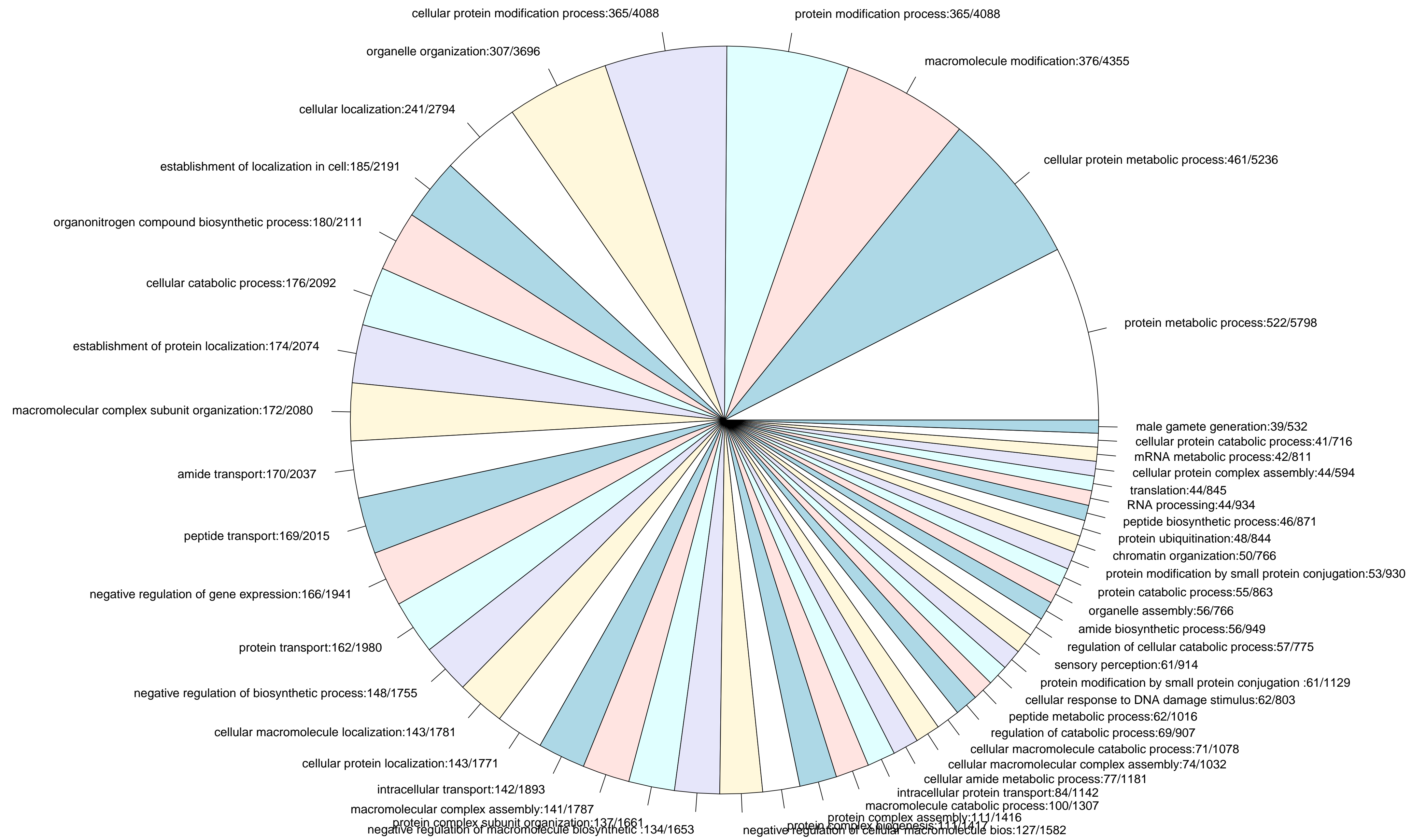

Supplement: DATASET S5 — GO-term analyses of control cultures (EGFP-expressing and no injury) in 3D versus 2D. [file Data_Sheet_5.ZIP › SD5_3D_vs_2D/GOstats/GOstats_BP_Down_pieChart.pdf]

### GOstats\_BP\_Up\_pieChart

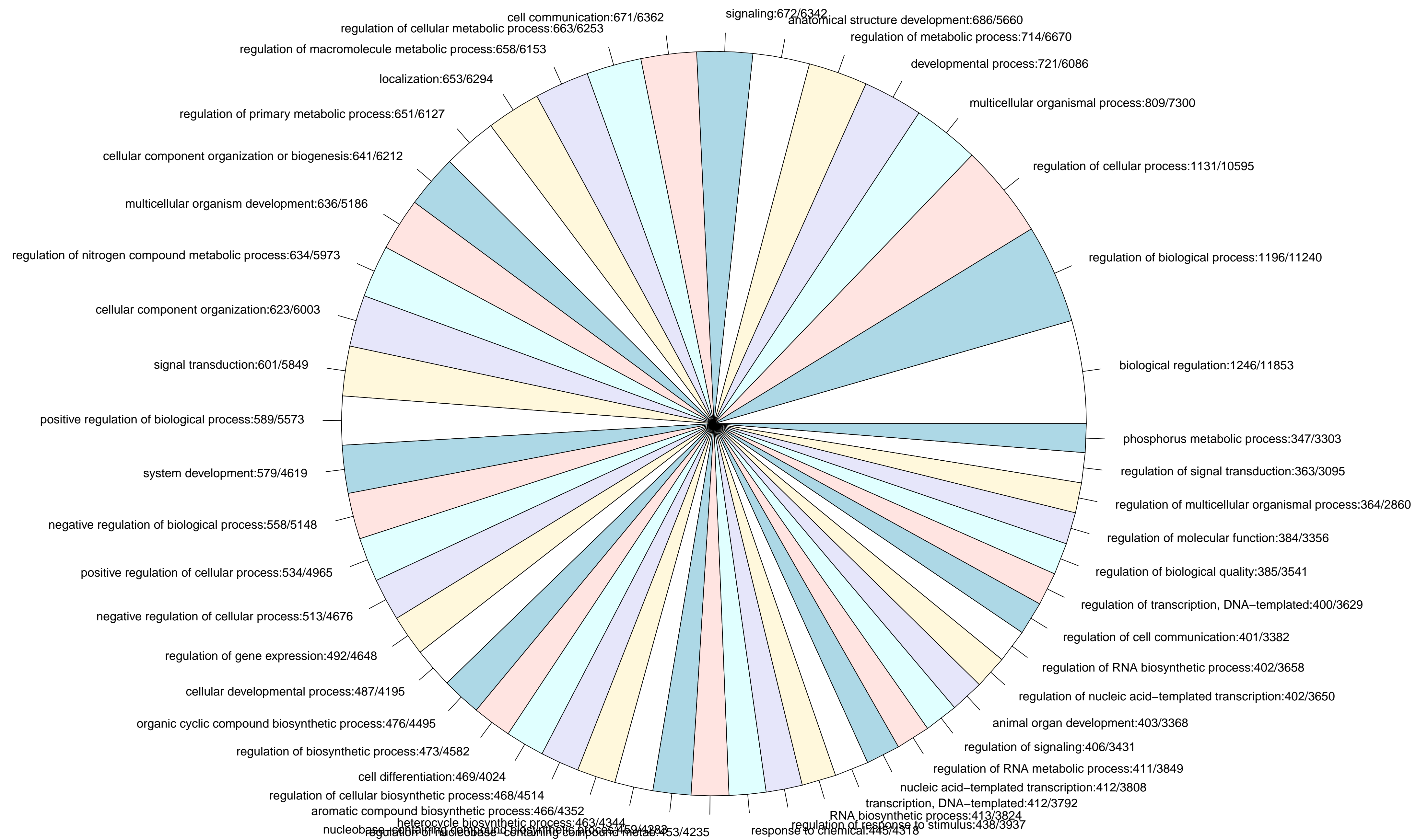

Supplement: DATASET S5 — GO-term analyses of control cultures (EGFP-expressing and no injury) in 3D versus 2D. [file Data_Sheet_5.ZIP › SD5_3D_vs_2D/GOstats/GOstats_BP_Up_pieChart.pdf]

GOstats\_CC\_Down\_pieChart

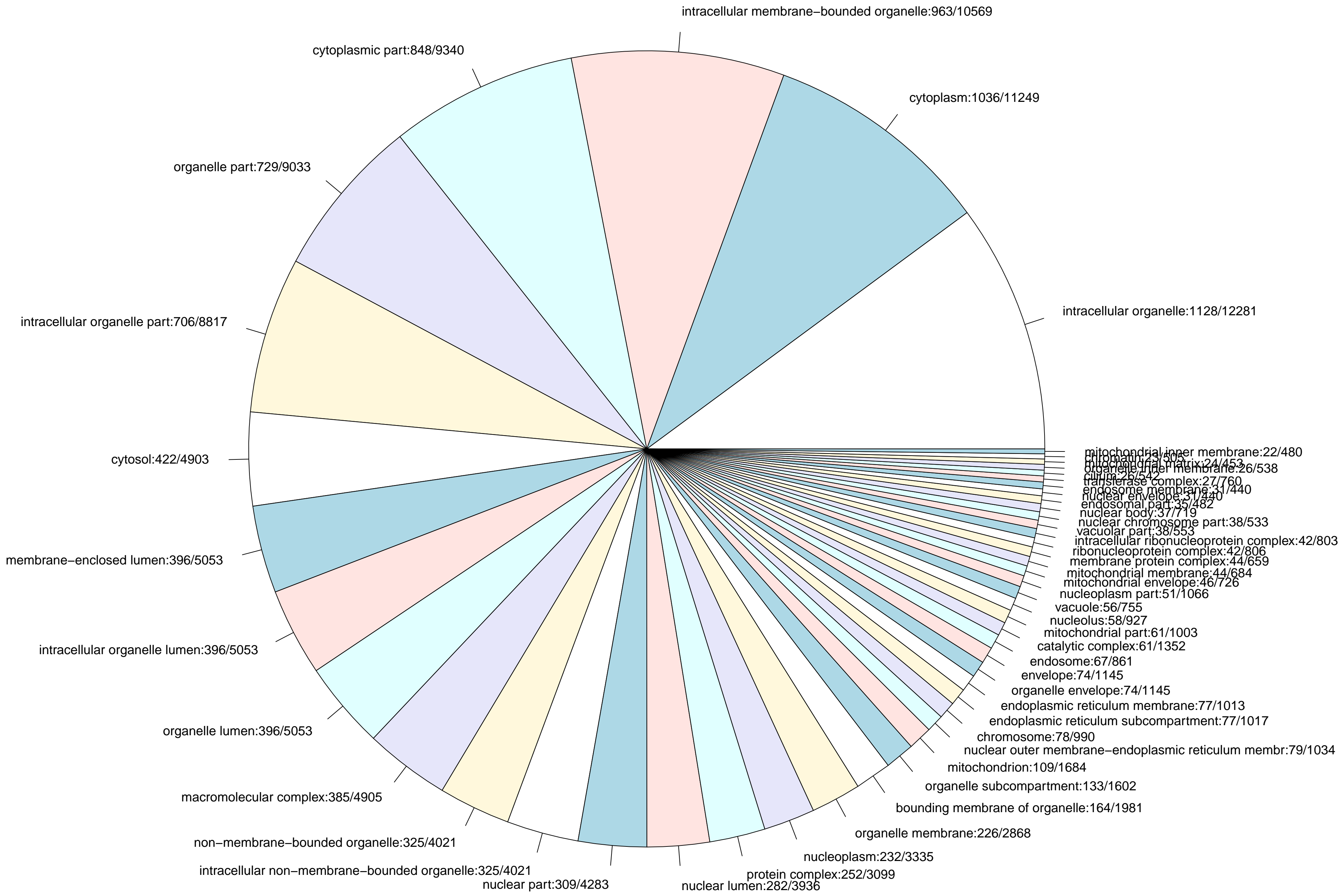

Supplement: DATASET S5 — GO-term analyses of control cultures (EGFP-expressing and no injury) in 3D versus 2D. [file Data_Sheet_5.ZIP › SD5_3D_vs_2D/GOstats/GOstats_CC_Down_pieChart.pdf]

GOstats\_CC\_Up\_pieChart

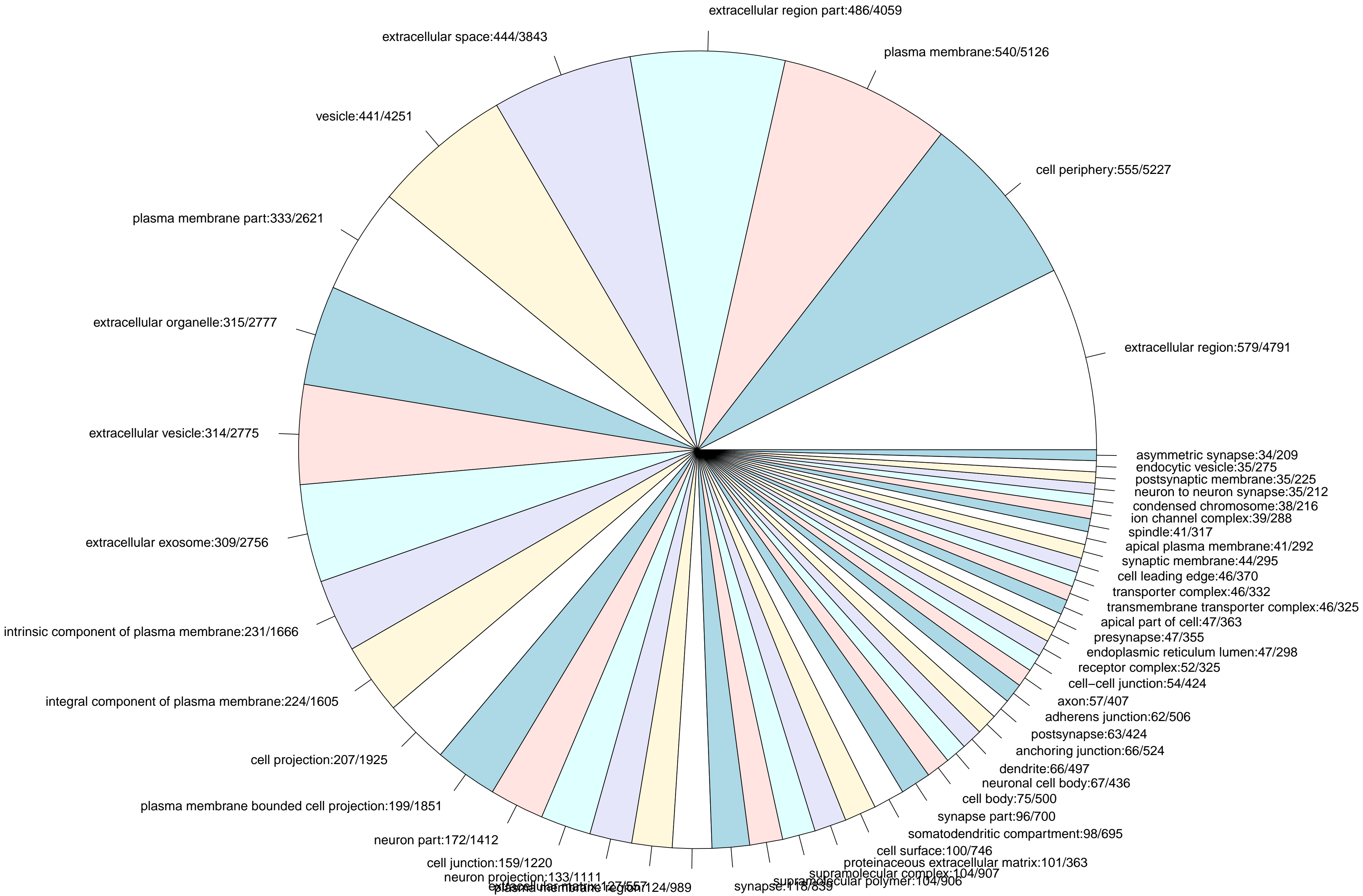

Supplement: DATASET S5 — GO-term analyses of control cultures (EGFP-expressing and no injury) in 3D versus 2D. [file Data_Sheet_5.ZIP › SD5_3D_vs_2D/GOstats/GOstats_CC_Up_pieChart.pdf]

GOstats\_kegg\_Under

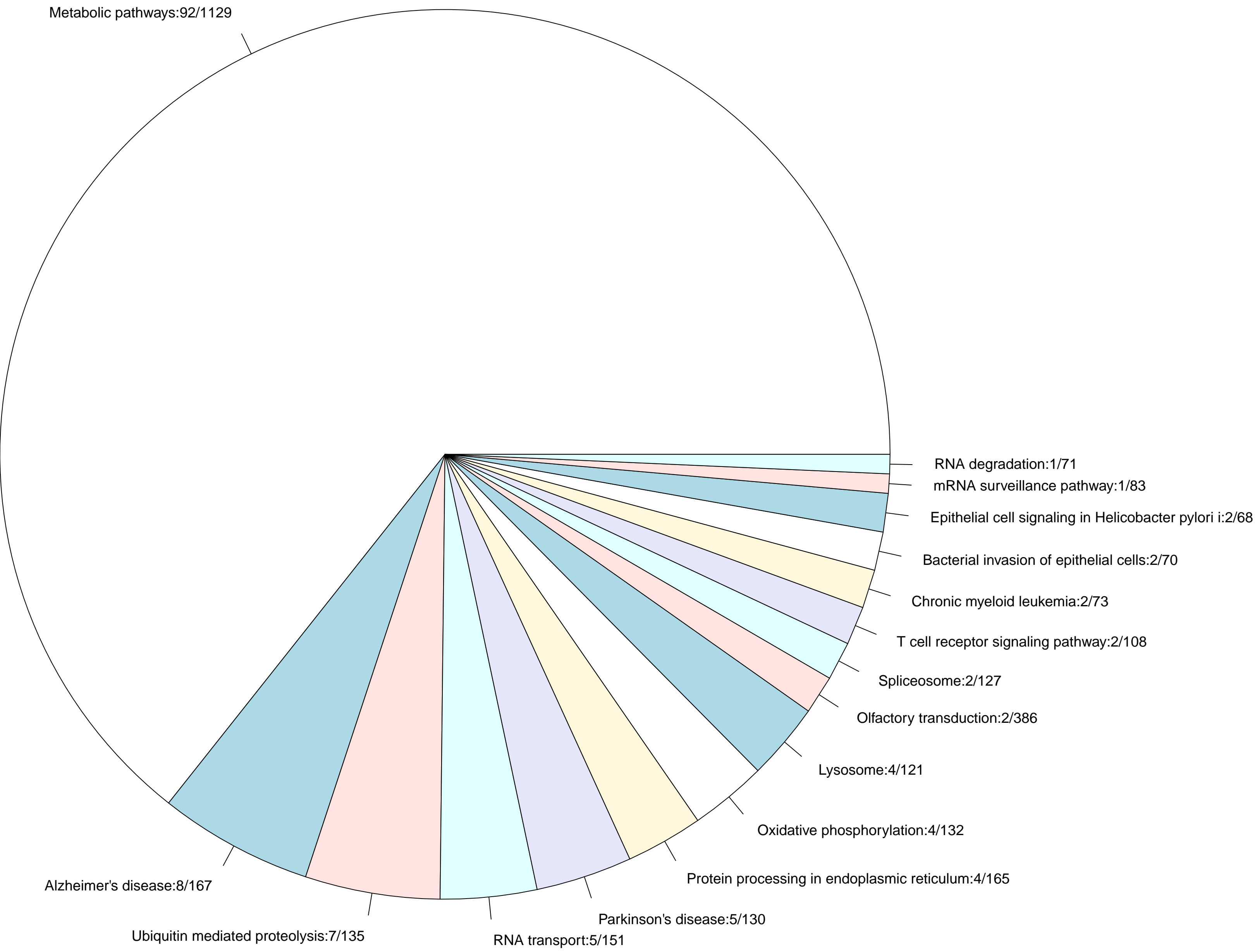

Supplement: DATASET S5 — GO-term analyses of control cultures (EGFP-expressing and no injury) in 3D versus 2D. [file Data_Sheet_5.ZIP › SD5_3D_vs_2D/GOstats/GOstats_kegg_Under.pdf]

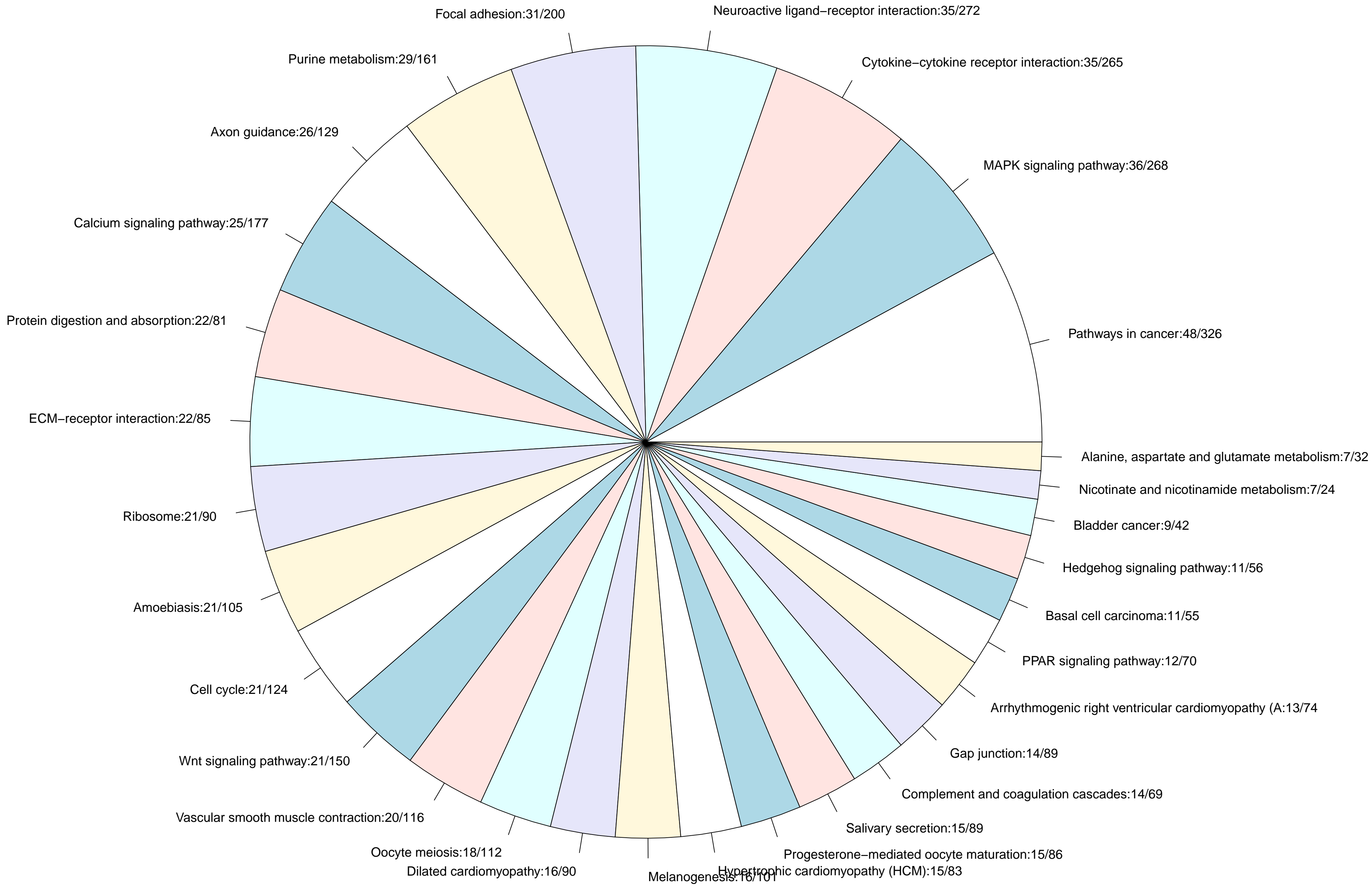

Supplement: DATASET S5 — GO-term analyses of control cultures (EGFP-expressing and no injury) in 3D versus 2D. [file Data_Sheet_5.ZIP › SD5_3D_vs_2D/GOstats/GOstats_kegg_Up.pdf]

### GOstats\_MF\_Down\_pieChart

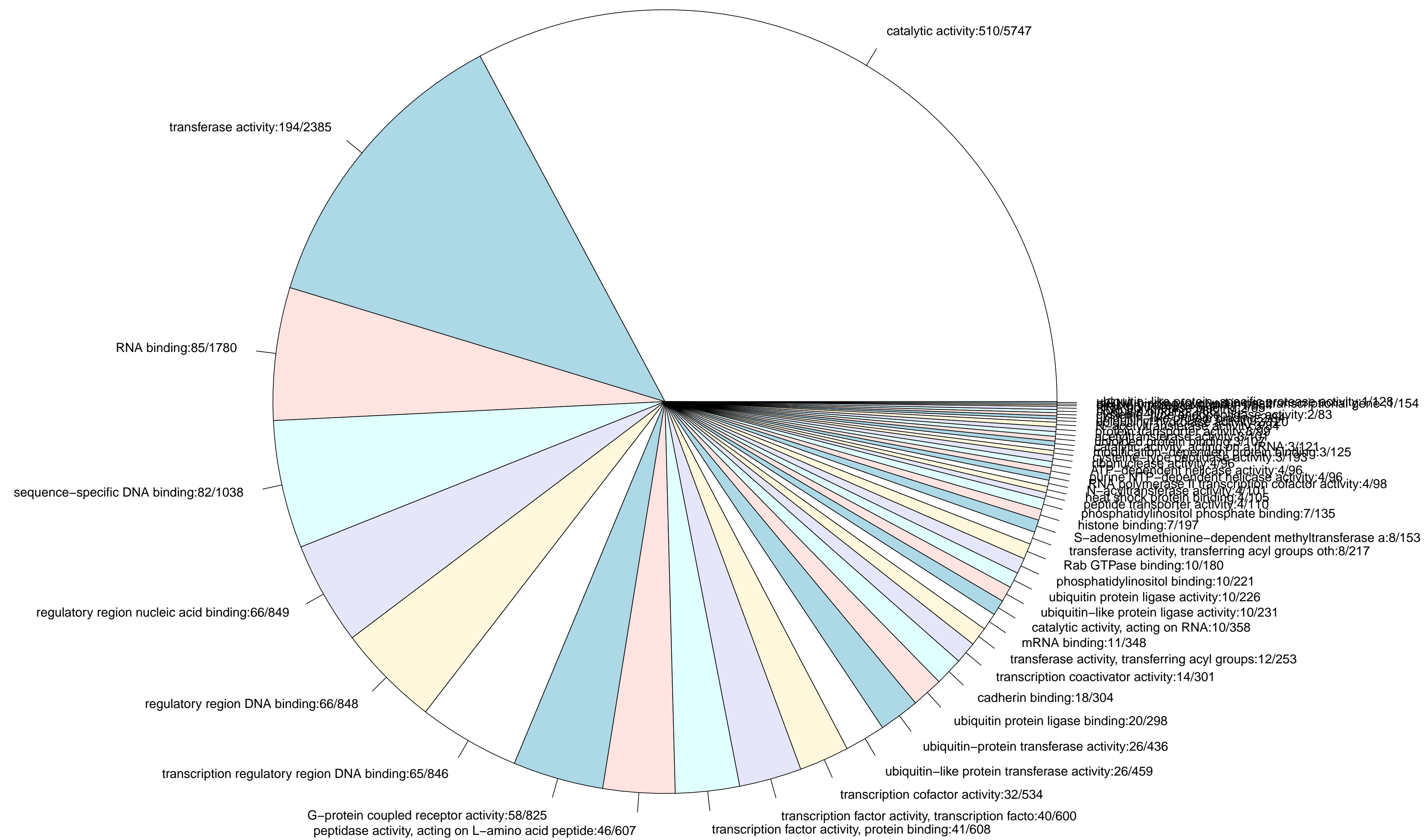

Supplement: DATASET S5 — GO-term analyses of control cultures (EGFP-expressing and no injury) in 3D versus 2D. [file Data_Sheet_5.ZIP › SD5_3D_vs_2D/GOstats/GOstats_MF_Down_pieChart.pdf]

GOstats\_MF\_Up\_pieChart

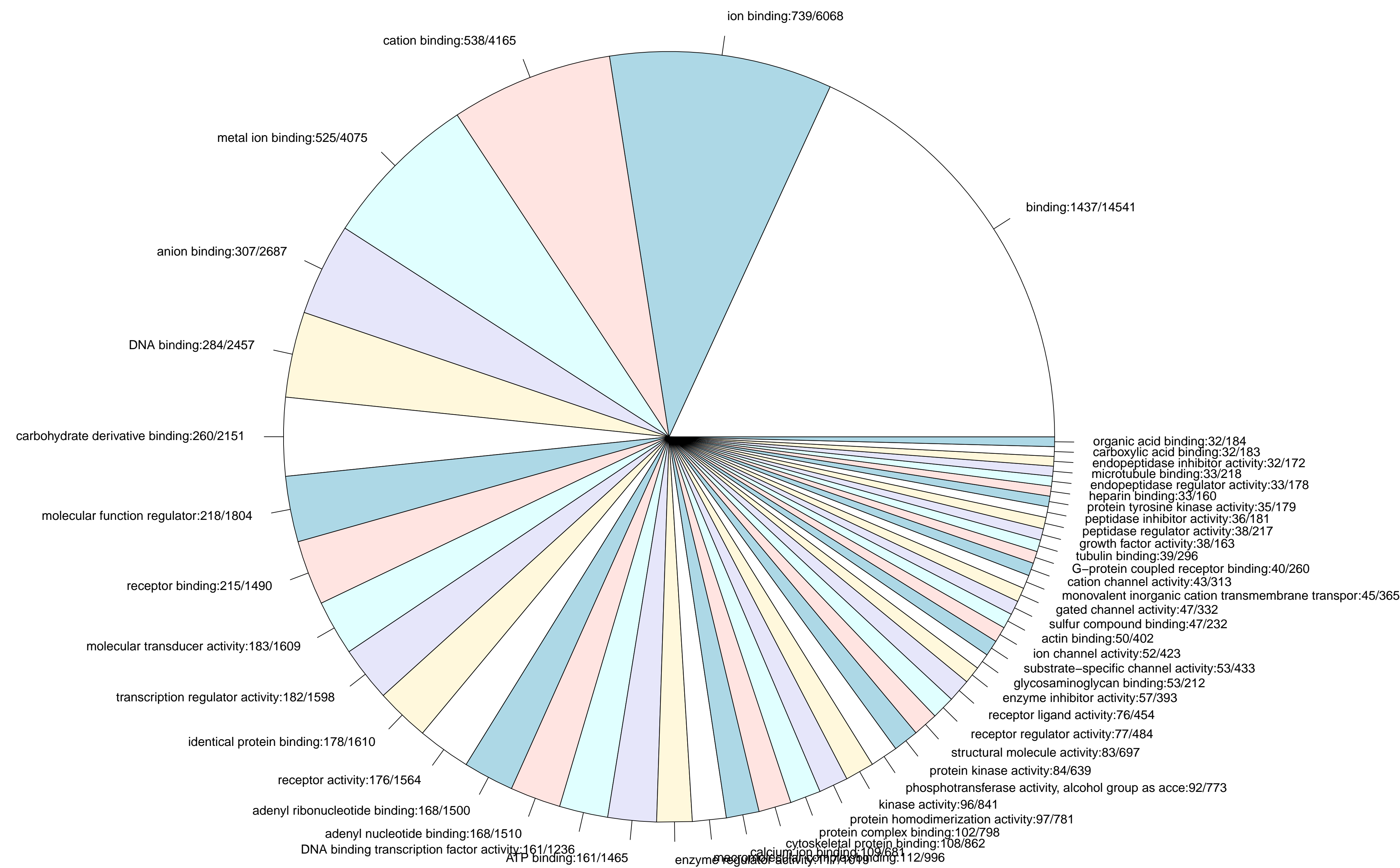

Supplement: DATASET S5 — GO-term analyses of control cultures (EGFP-expressing and no injury) in 3D versus 2D. [file Data_Sheet_5.ZIP › SD5_3D_vs_2D/GOstats/GOstats_MF_Up_pieChart.pdf]

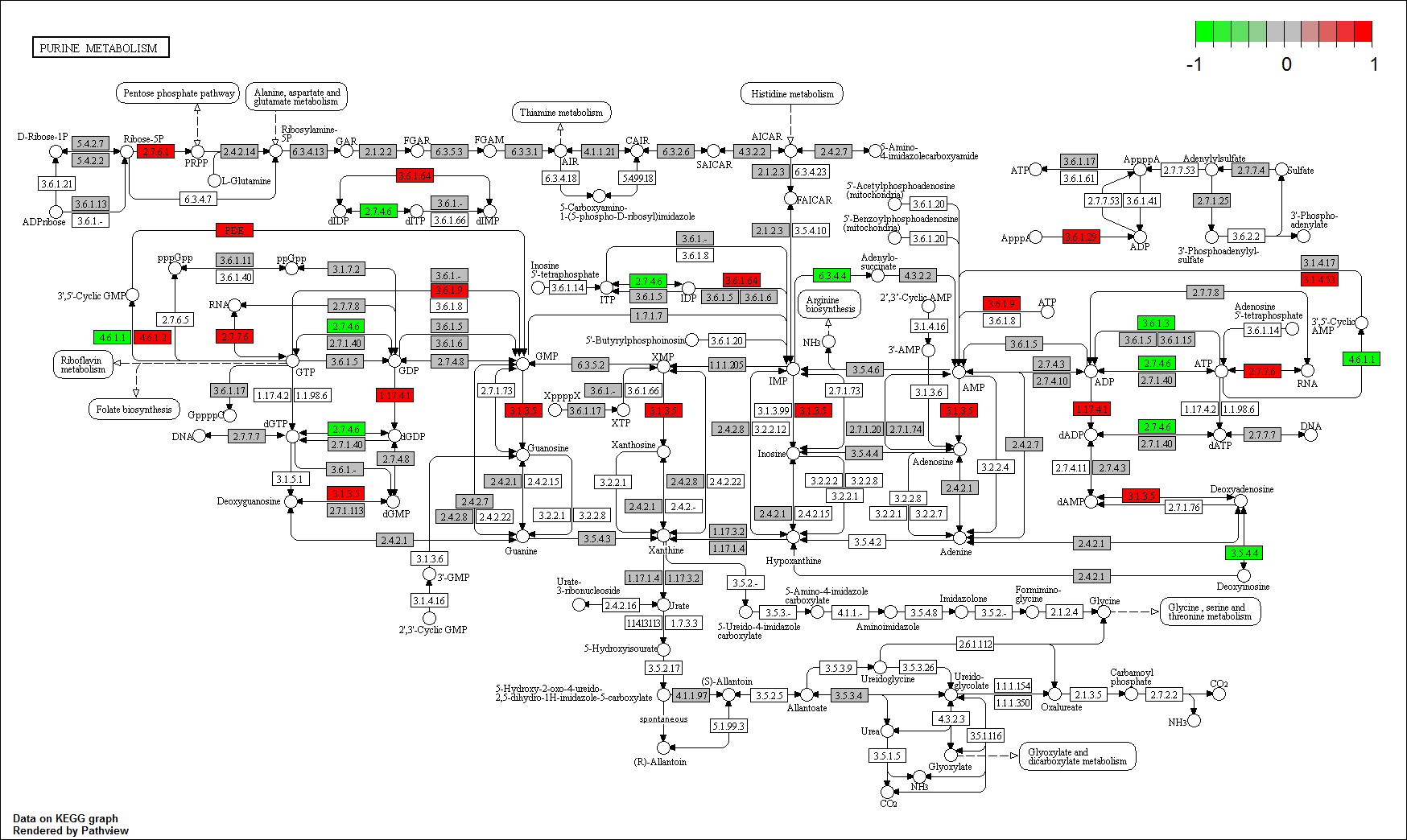

Supplement: DATASET S5 — GO-term analyses of control cultures (EGFP-expressing and no injury) in 3D versus 2D. [file Data_Sheet_5.ZIP › SD5_3D_vs_2D/GOstats/hsa00230.Purinemetabolism.png]

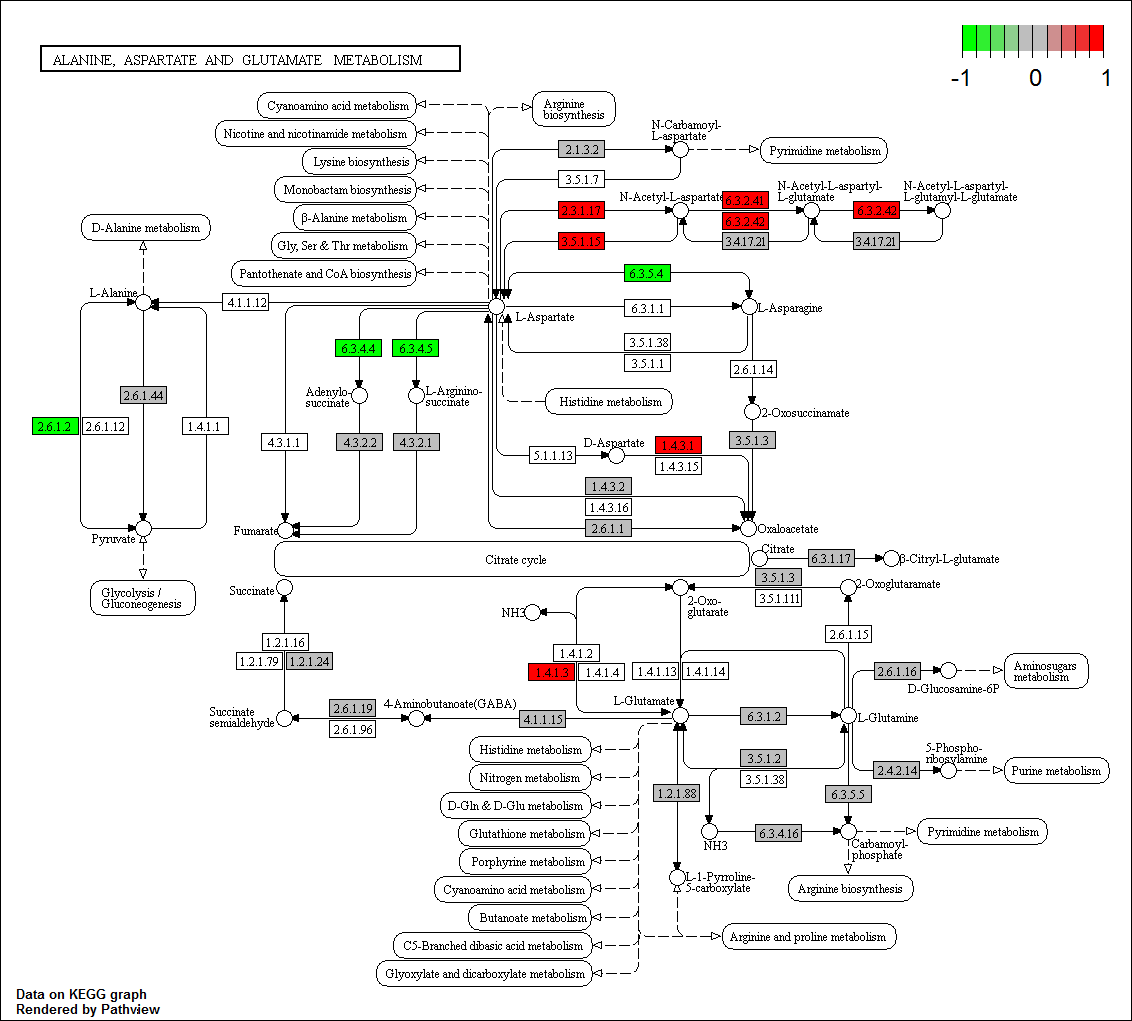

Supplement: DATASET S5 — GO-term analyses of control cultures (EGFP-expressing and no injury) in 3D versus 2D. [file Data_Sheet_5.ZIP › SD5_3D_vs_2D/GOstats/hsa00250.Alanine,aspartateandglutamatemetabolism.png]

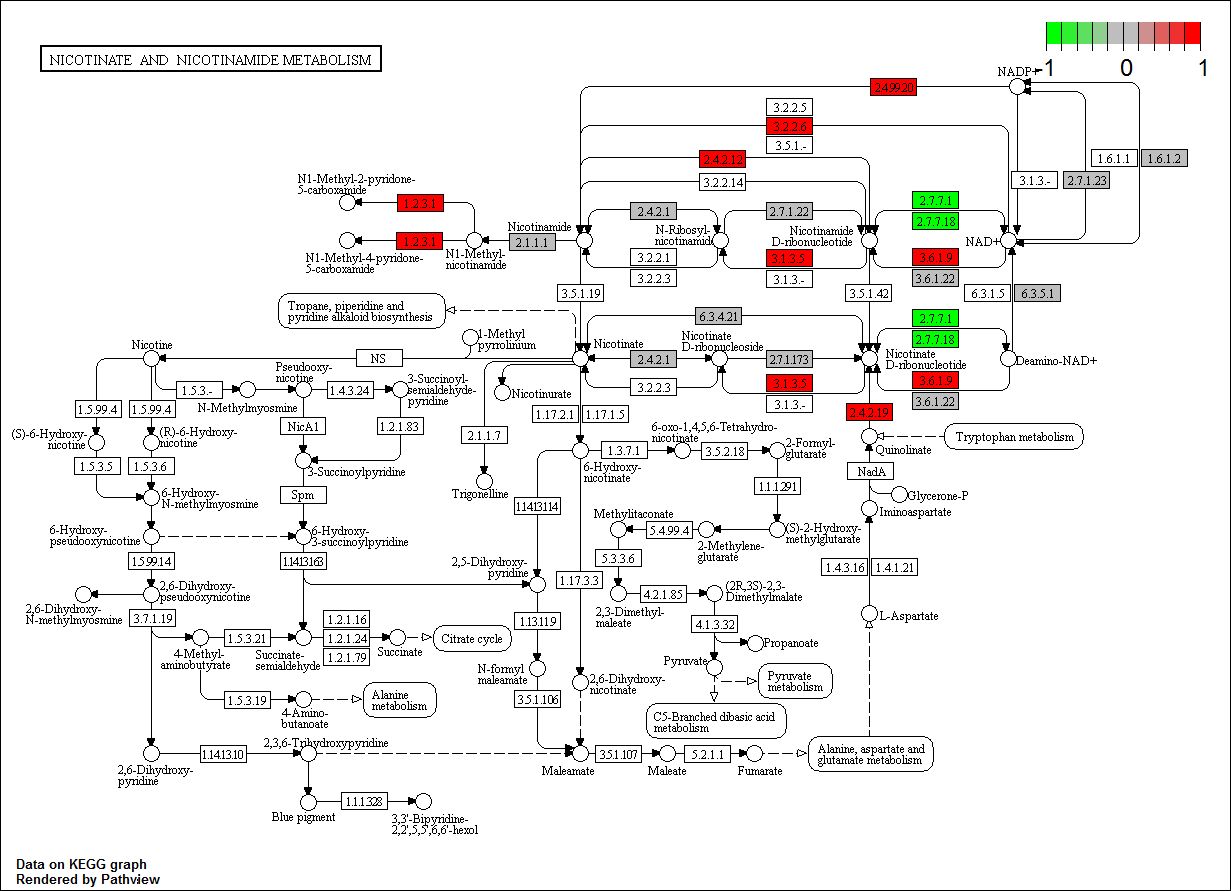

Supplement: DATASET S5 — GO-term analyses of control cultures (EGFP-expressing and no injury) in 3D versus 2D. [file Data_Sheet_5.ZIP › SD5_3D_vs_2D/GOstats/hsa00760.Nicotinateandnicotinamidemetabolism.png]

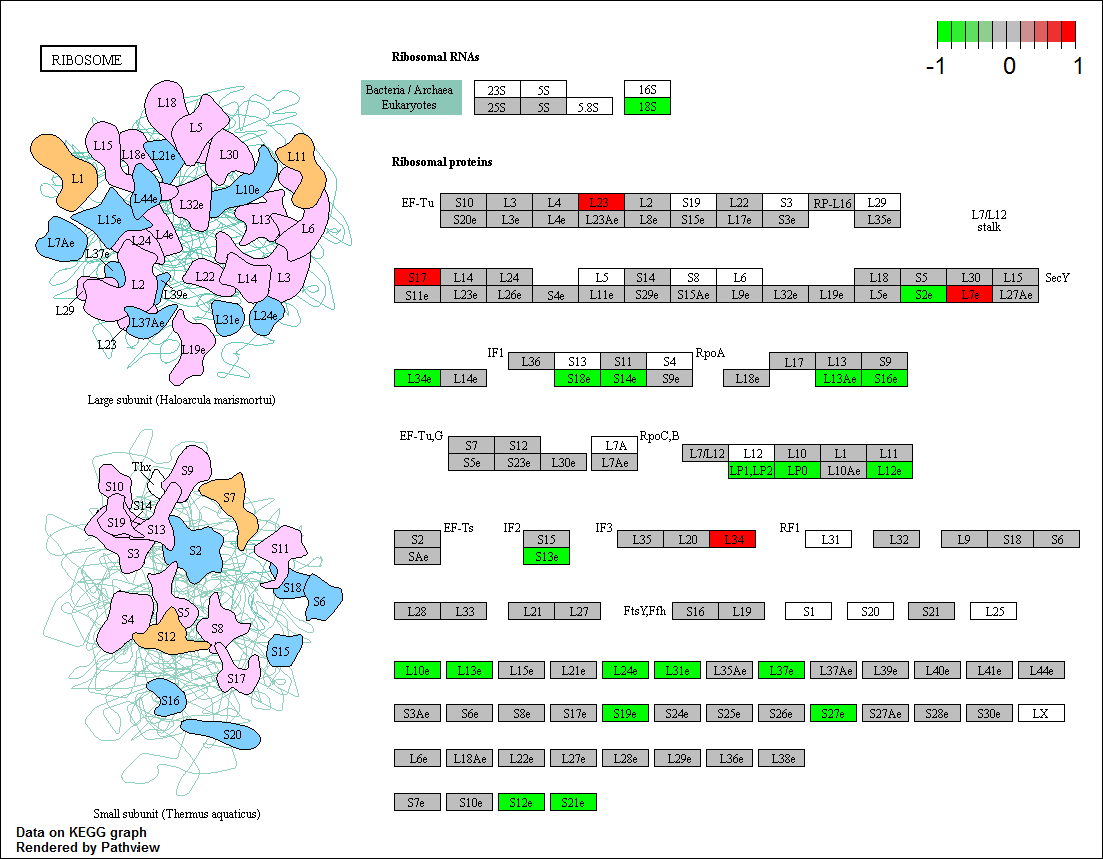

Supplement: DATASET S5 — GO-term analyses of control cultures (EGFP-expressing and no injury) in 3D versus 2D. [file Data_Sheet_5.ZIP › SD5_3D_vs_2D/GOstats/hsa03010.Ribosome.png]

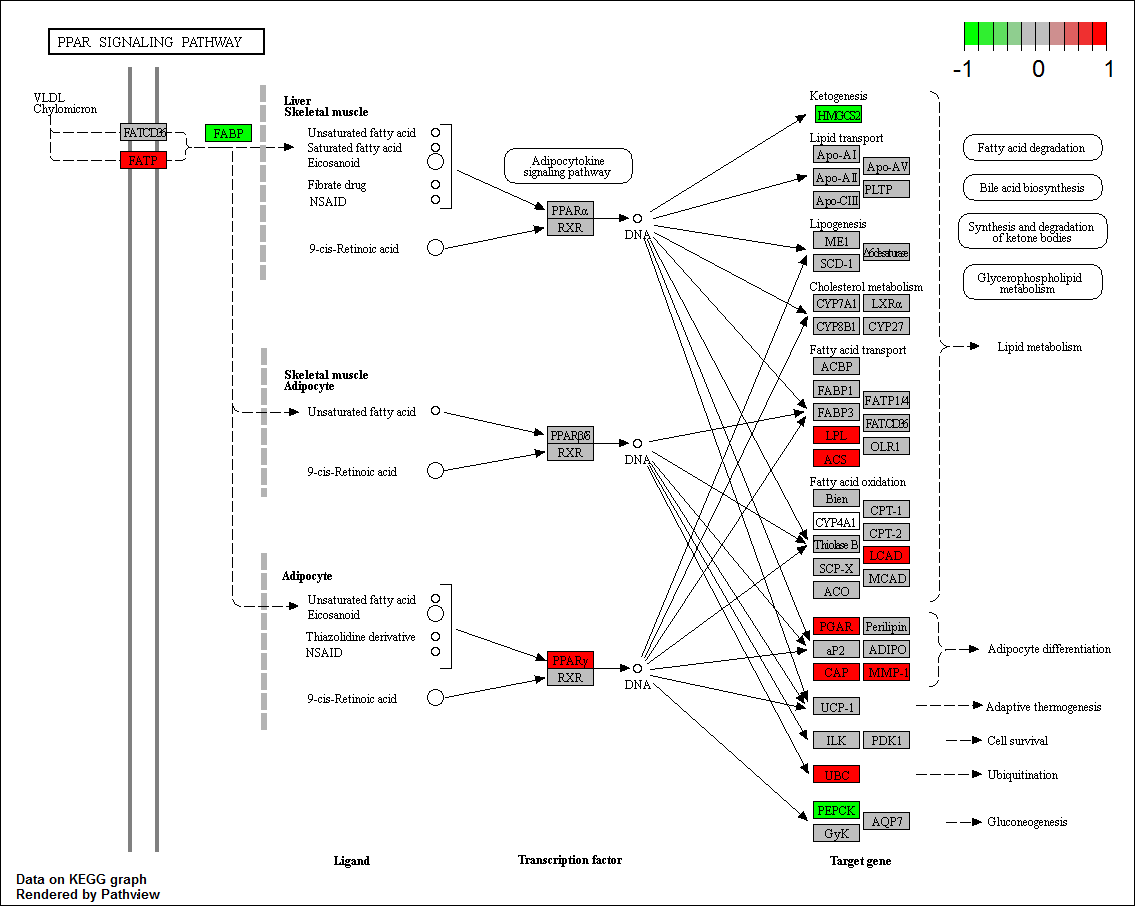

Supplement: DATASET S5 — GO-term analyses of control cultures (EGFP-expressing and no injury) in 3D versus 2D. [file Data_Sheet_5.ZIP › SD5_3D_vs_2D/GOstats/hsa03320.PPARsignalingpathway.png]

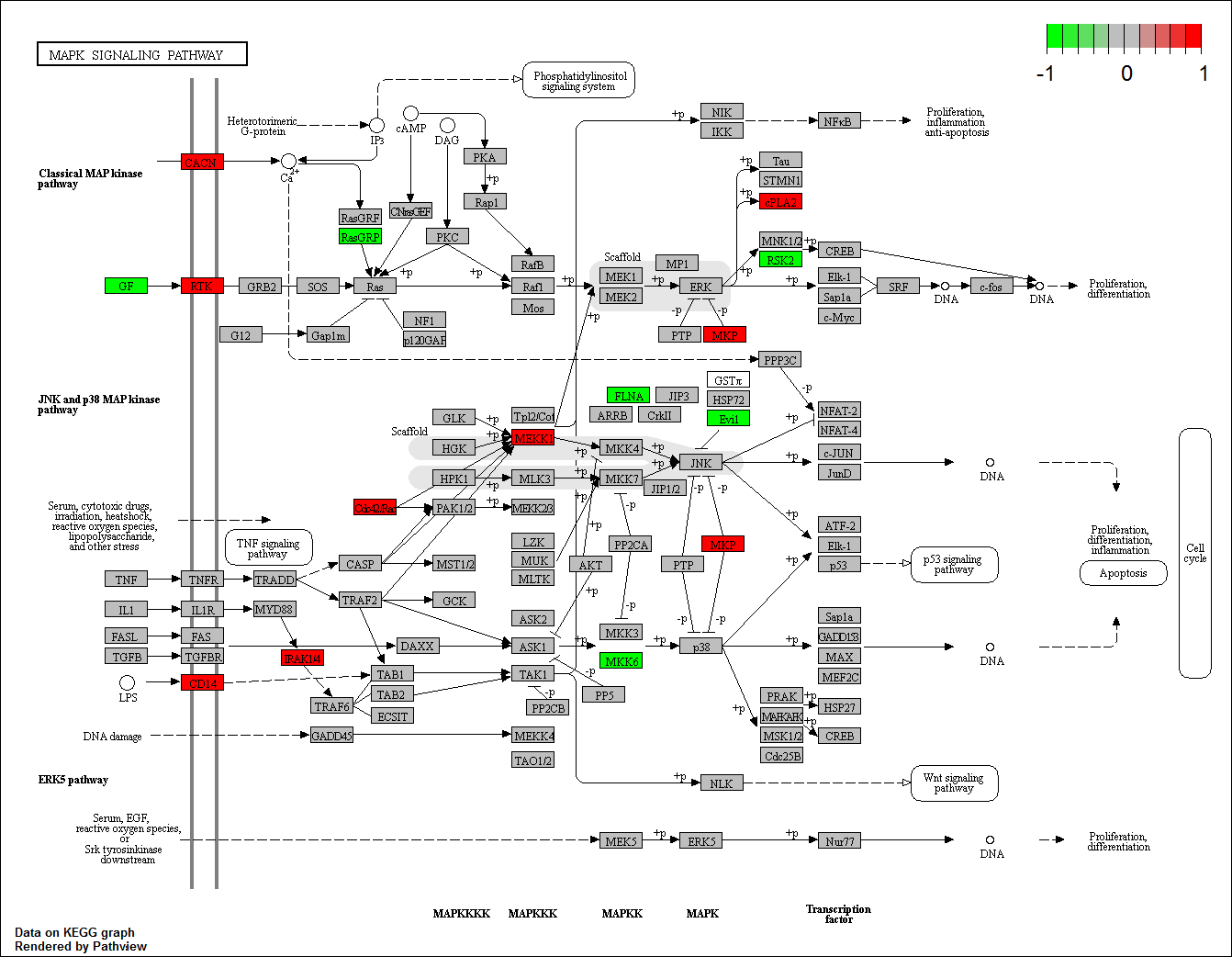

Supplement: DATASET S5 — GO-term analyses of control cultures (EGFP-expressing and no injury) in 3D versus 2D. [file Data_Sheet_5.ZIP › SD5_3D_vs_2D/GOstats/hsa04010.MAPKsignalingpathway.png]

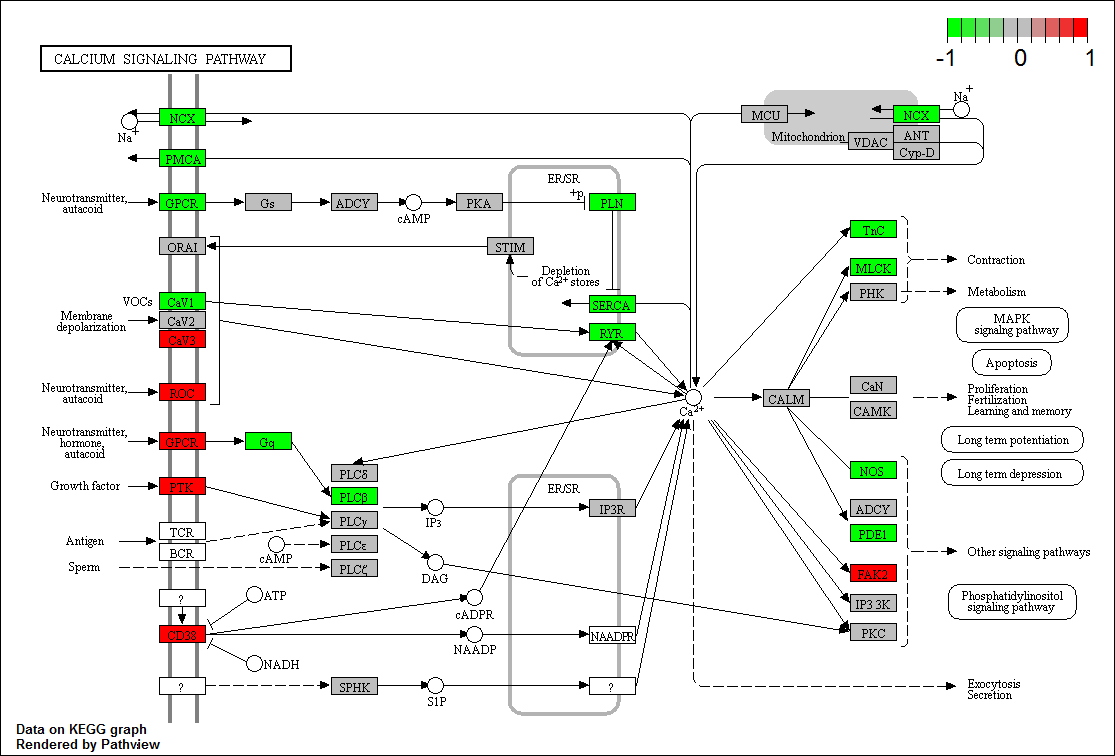

Supplement: DATASET S5 — GO-term analyses of control cultures (EGFP-expressing and no injury) in 3D versus 2D. [file Data_Sheet_5.ZIP › SD5_3D_vs_2D/GOstats/hsa04020.Calciumsignalingpathway.png]

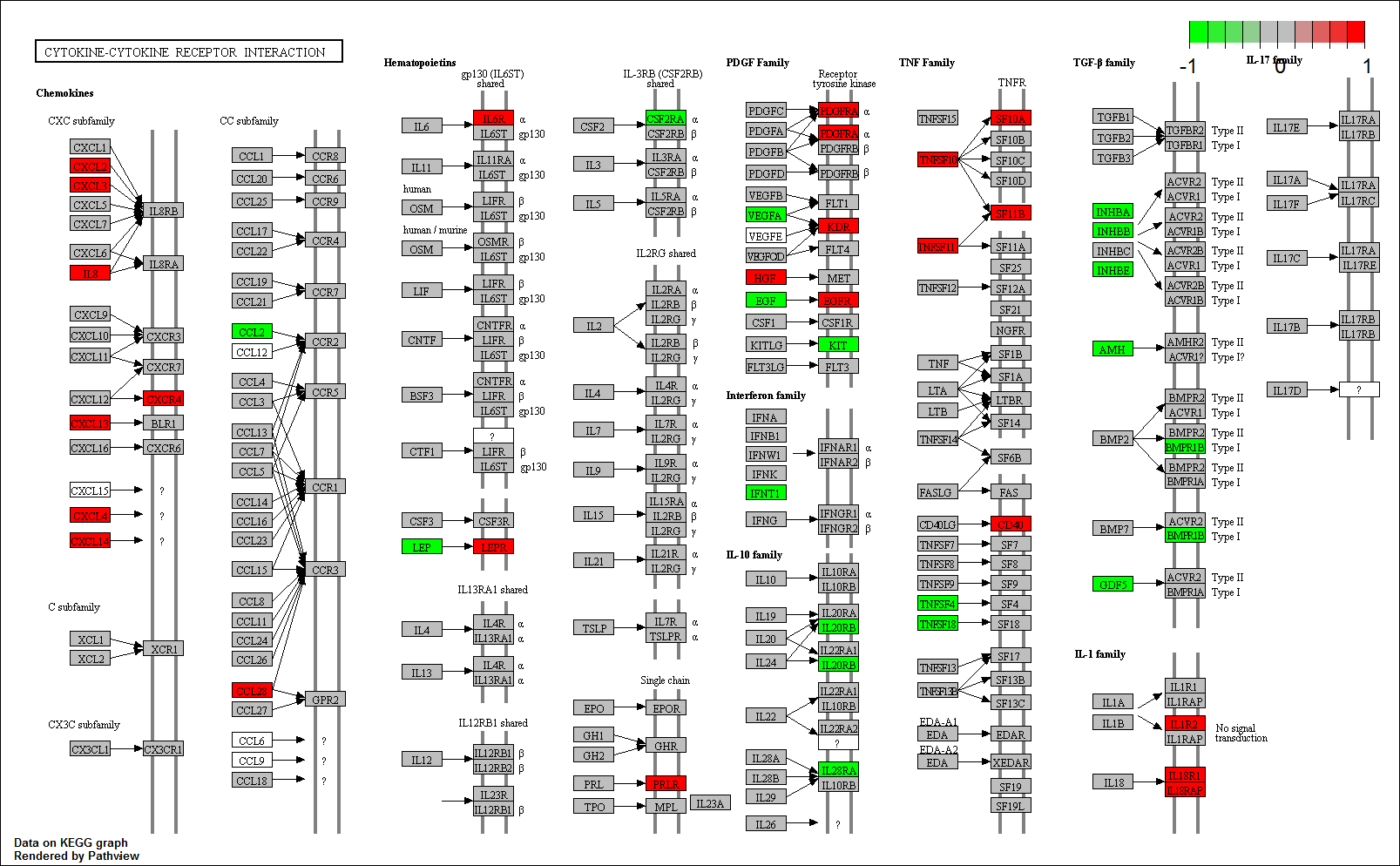

Supplement: DATASET S5 — GO-term analyses of control cultures (EGFP-expressing and no injury) in 3D versus 2D. [file Data_Sheet_5.ZIP › SD5_3D_vs_2D/GOstats/hsa04060.Cytokine-cytokinereceptorinteraction.png]

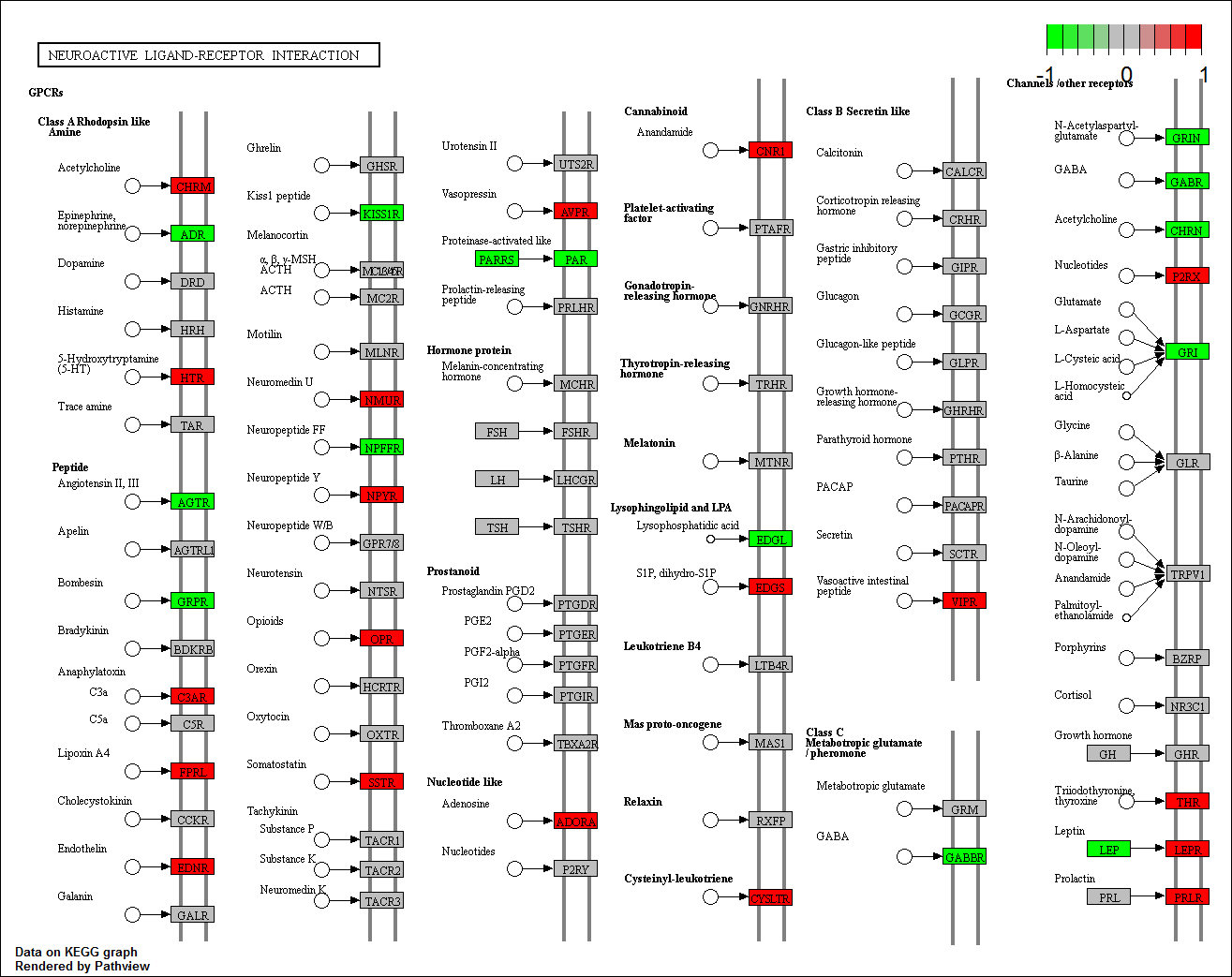

Supplement: DATASET S5 — GO-term analyses of control cultures (EGFP-expressing and no injury) in 3D versus 2D. [file Data_Sheet_5.ZIP › SD5_3D_vs_2D/GOstats/hsa04080.Neuroactiveligand-receptorinteraction.png]

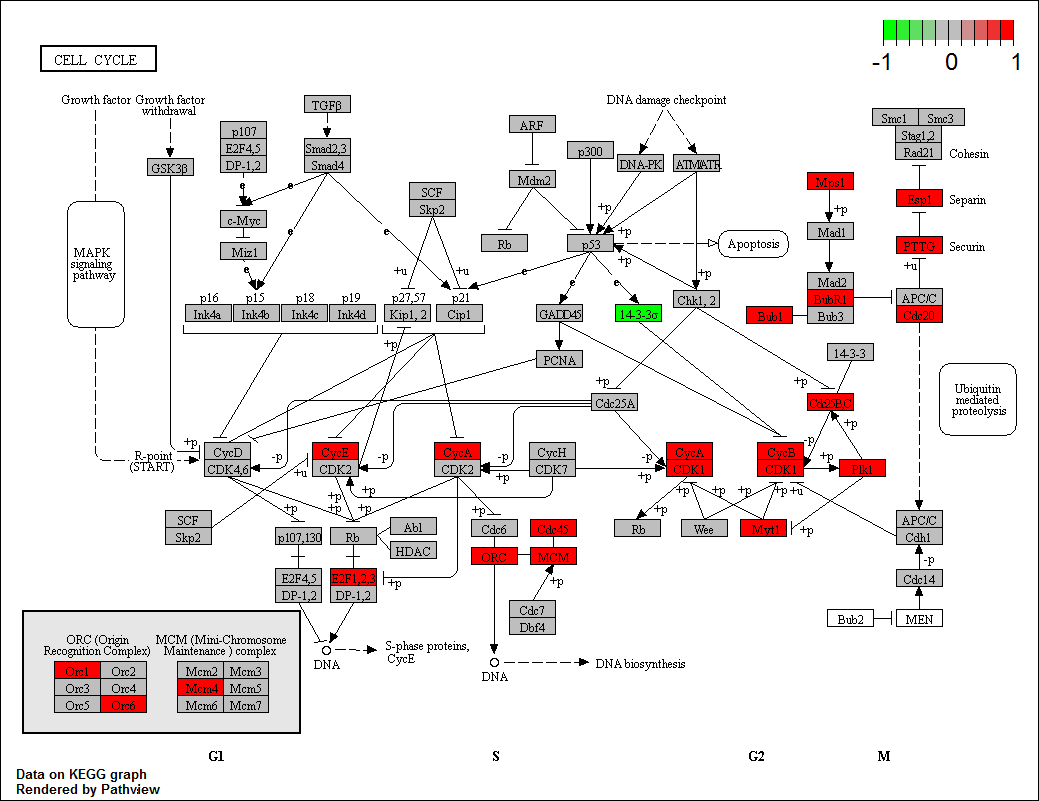

Supplement: DATASET S5 — GO-term analyses of control cultures (EGFP-expressing and no injury) in 3D versus 2D. [file Data_Sheet_5.ZIP › SD5_3D_vs_2D/GOstats/hsa04110.Cellcycle.png]

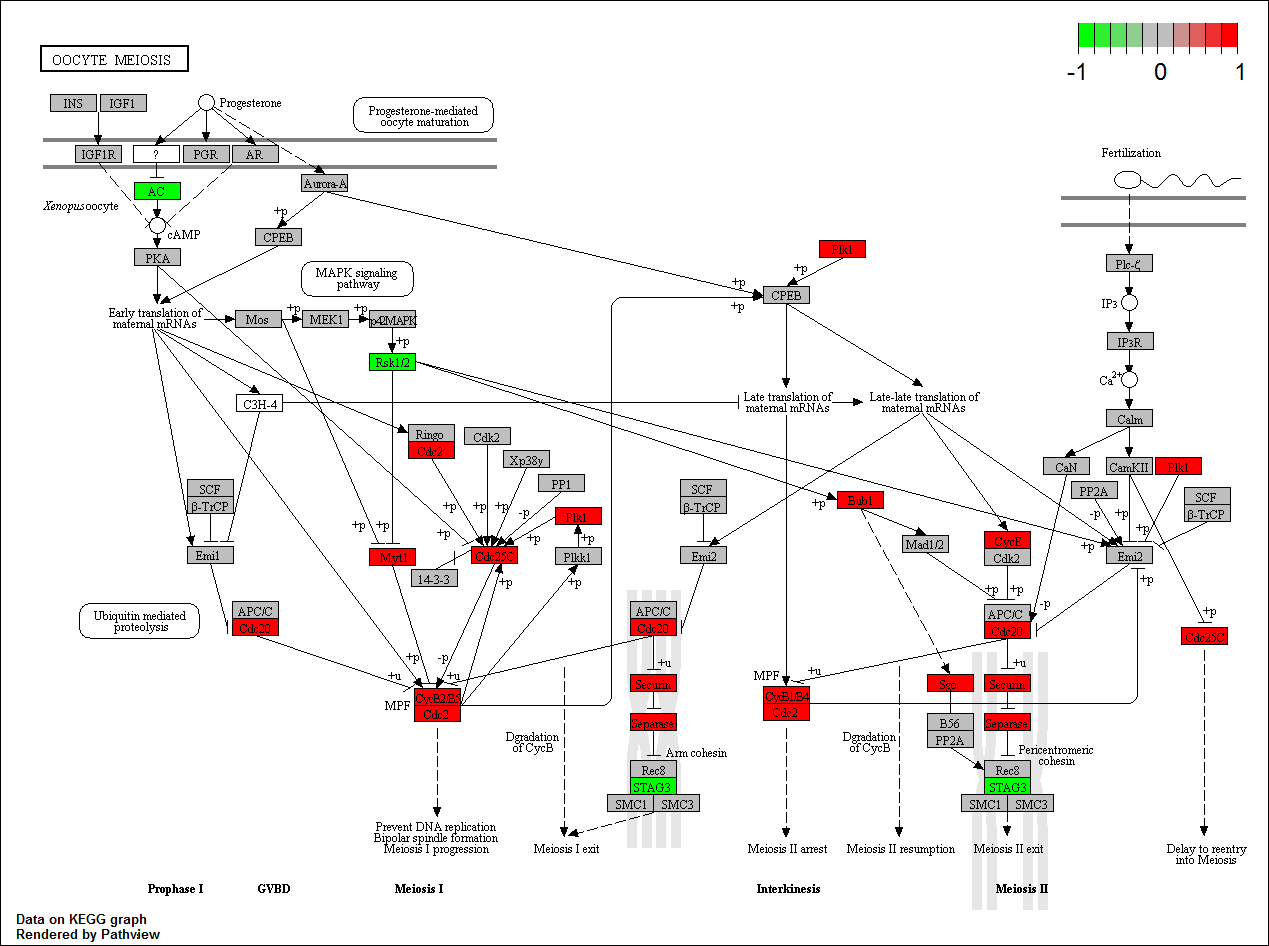

Supplement: DATASET S5 — GO-term analyses of control cultures (EGFP-expressing and no injury) in 3D versus 2D. [file Data_Sheet_5.ZIP › SD5_3D_vs_2D/GOstats/hsa04114.Oocytemeiosis.png]

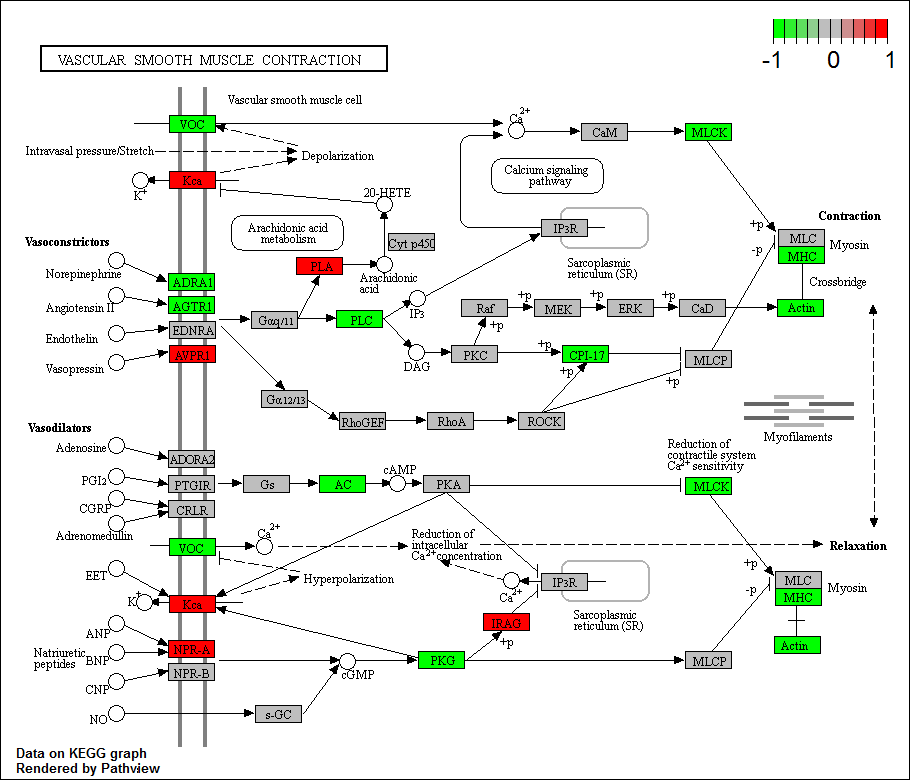

Supplement: DATASET S5 — GO-term analyses of control cultures (EGFP-expressing and no injury) in 3D versus 2D. [file Data_Sheet_5.ZIP › SD5_3D_vs_2D/GOstats/hsa04270.Vascularsmoothmusclecontraction.png]

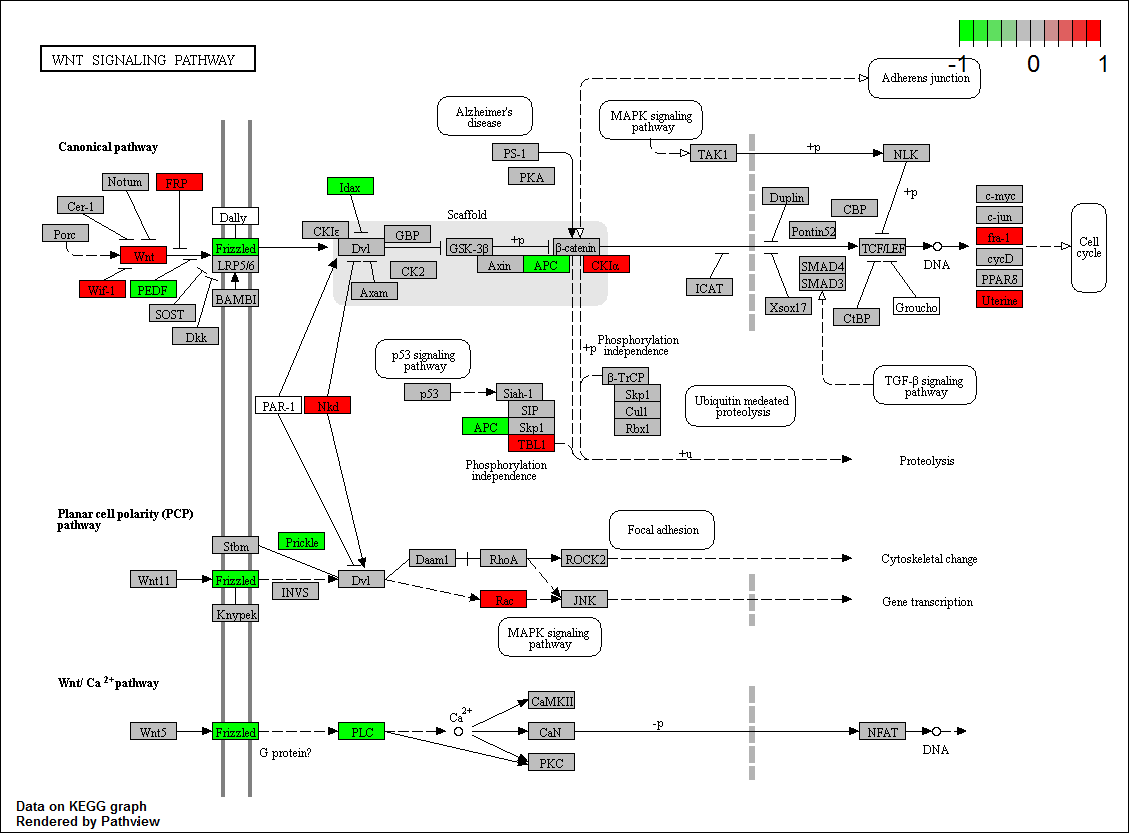

Supplement: DATASET S5 — GO-term analyses of control cultures (EGFP-expressing and no injury) in 3D versus 2D. [file Data_Sheet_5.ZIP › SD5_3D_vs_2D/GOstats/hsa04310.Wntsignalingpathway.png]

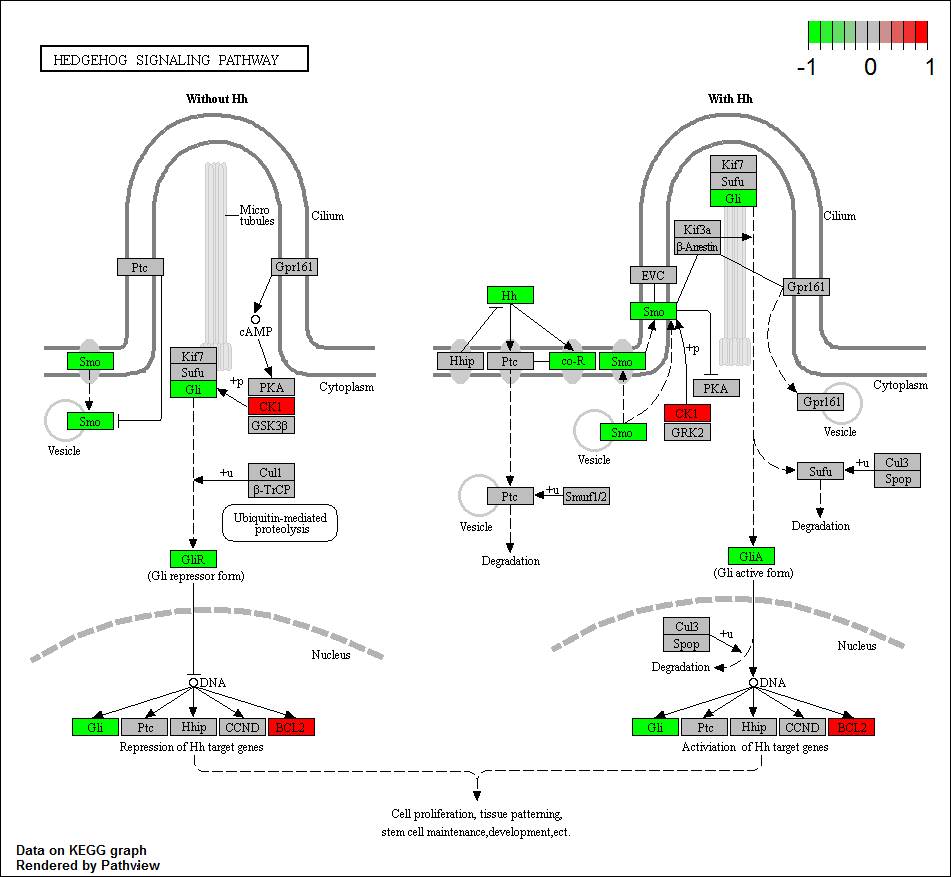

Supplement: DATASET S5 — GO-term analyses of control cultures (EGFP-expressing and no injury) in 3D versus 2D. [file Data_Sheet_5.ZIP › SD5_3D_vs_2D/GOstats/hsa04340.Hedgehogsignalingpathway.png]

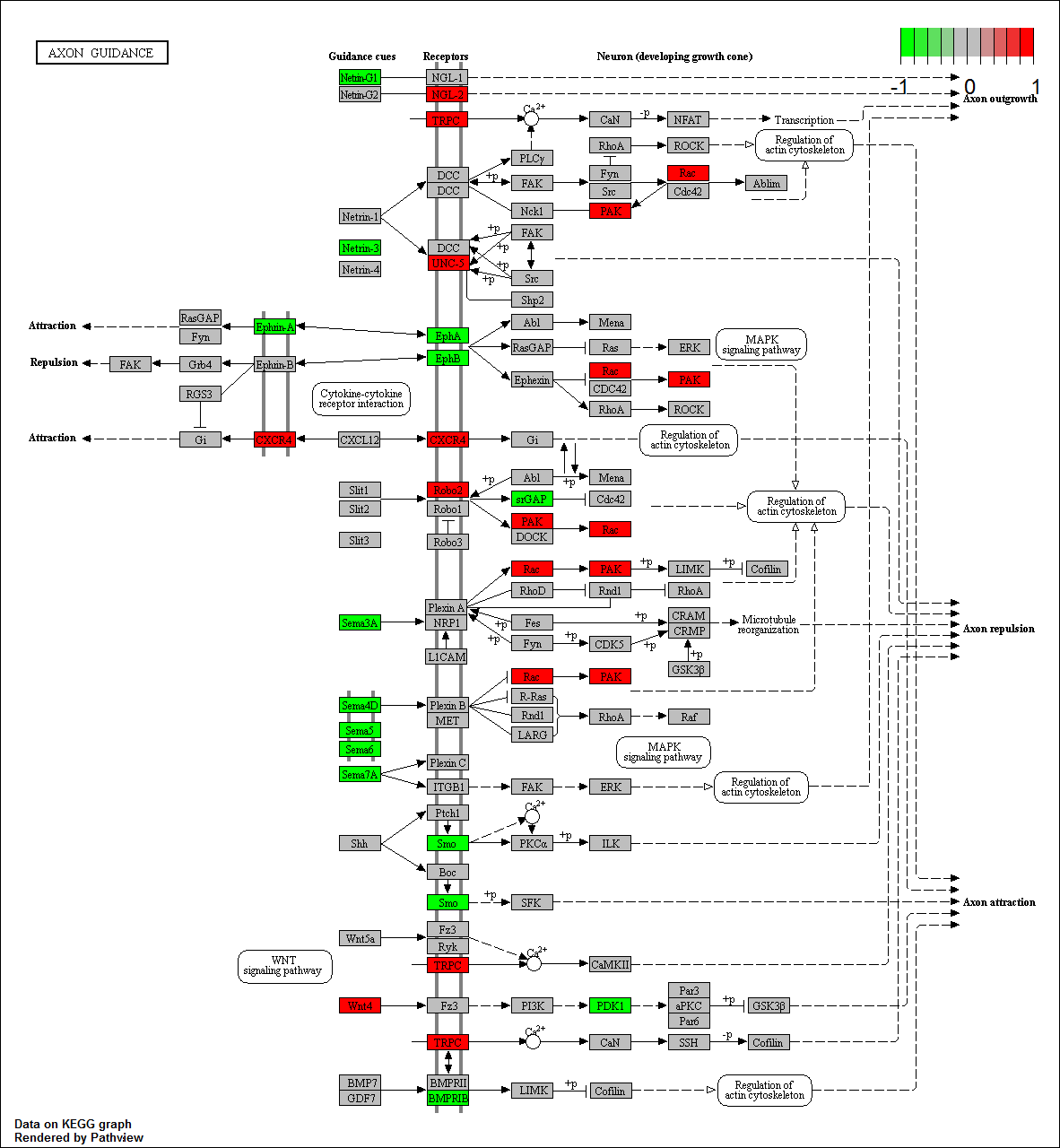

Supplement: DATASET S5 — GO-term analyses of control cultures (EGFP-expressing and no injury) in 3D versus 2D. [file Data_Sheet_5.ZIP › SD5_3D_vs_2D/GOstats/hsa04360.Axonguidance.png]

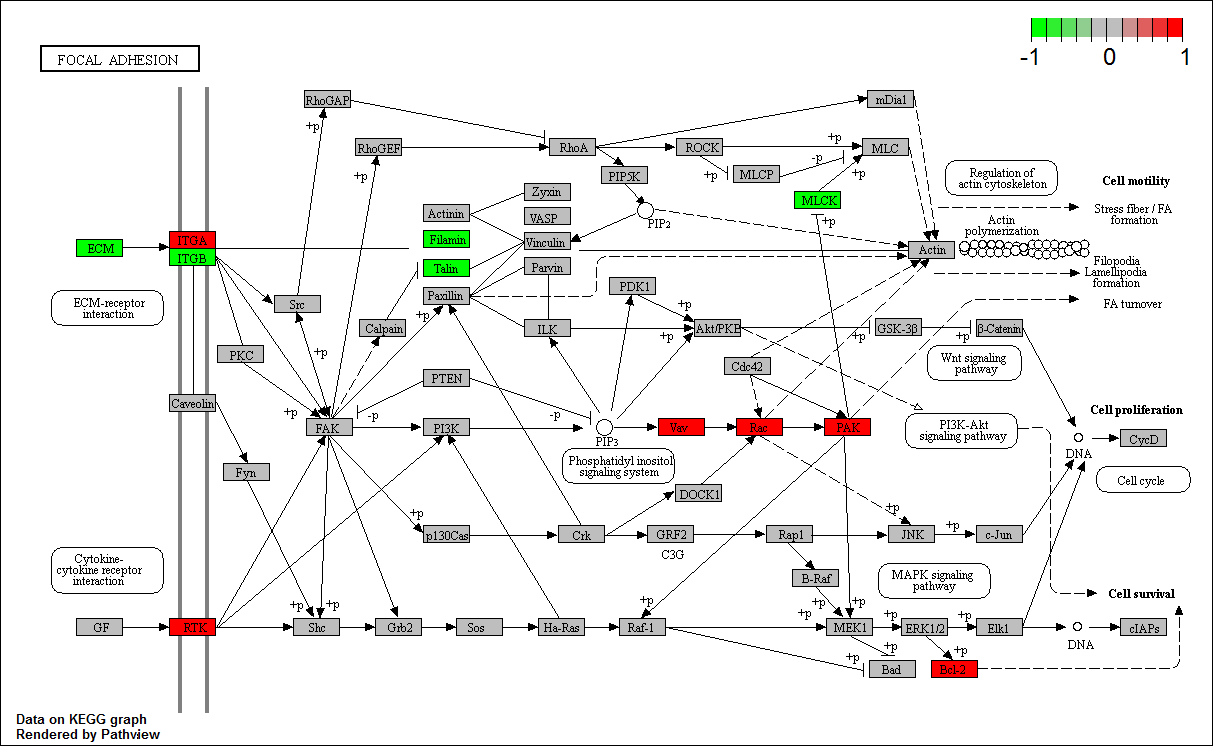

Supplement: DATASET S5 — GO-term analyses of control cultures (EGFP-expressing and no injury) in 3D versus 2D. [file Data_Sheet_5.ZIP › SD5_3D_vs_2D/GOstats/hsa04510.Focaladhesion.png]

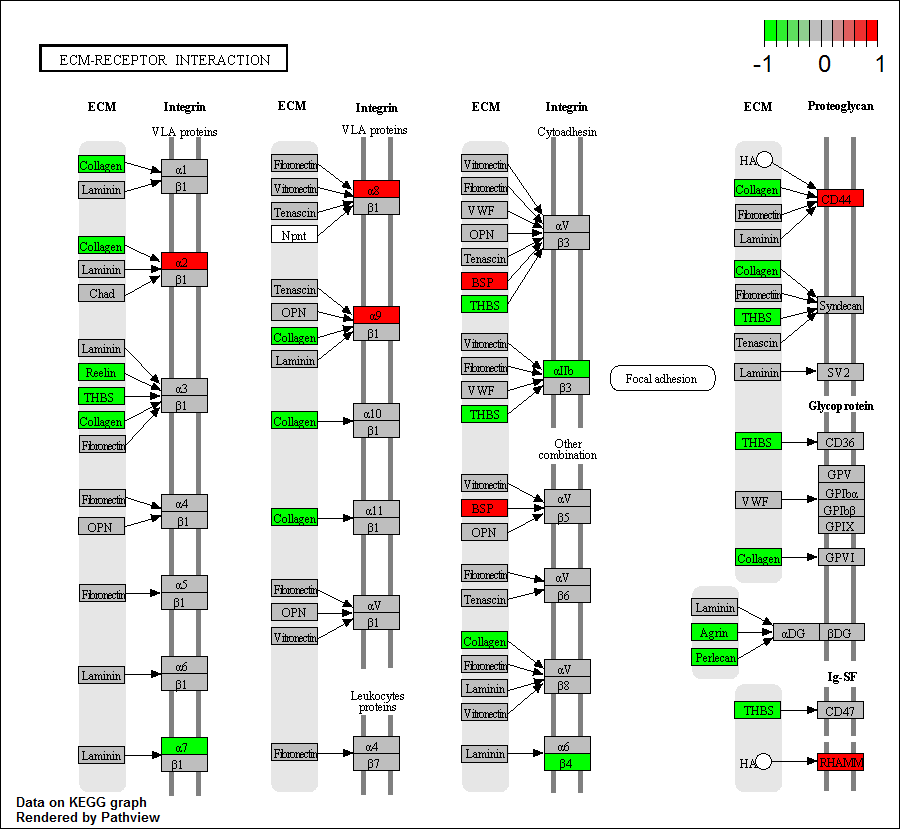

Supplement: DATASET S5 — GO-term analyses of control cultures (EGFP-expressing and no injury) in 3D versus 2D. [file Data_Sheet_5.ZIP › SD5_3D_vs_2D/GOstats/hsa04512.ECM-receptorinteraction.png]

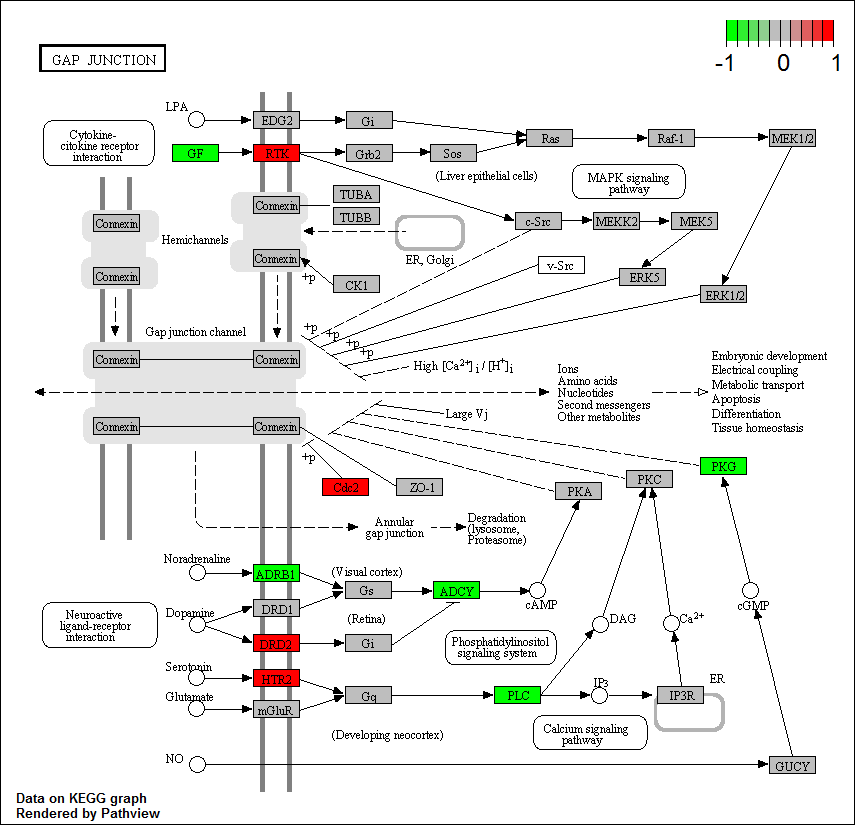

Supplement: DATASET S5 — GO-term analyses of control cultures (EGFP-expressing and no injury) in 3D versus 2D. [file Data_Sheet_5.ZIP › SD5_3D_vs_2D/GOstats/hsa04540.Gapjunction.png]

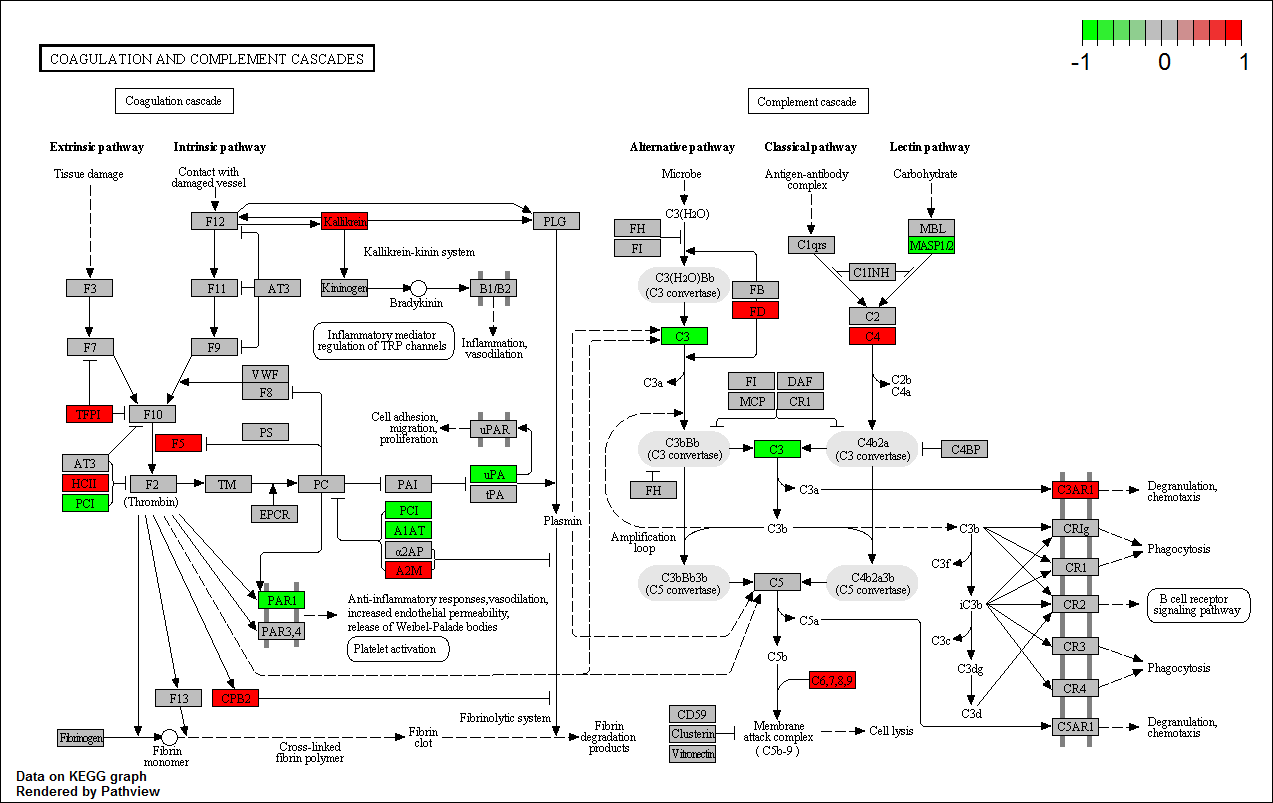

Supplement: DATASET S5 — GO-term analyses of control cultures (EGFP-expressing and no injury) in 3D versus 2D. [file Data_Sheet_5.ZIP › SD5_3D_vs_2D/GOstats/hsa04610.Complementandcoagulationcascades.png]

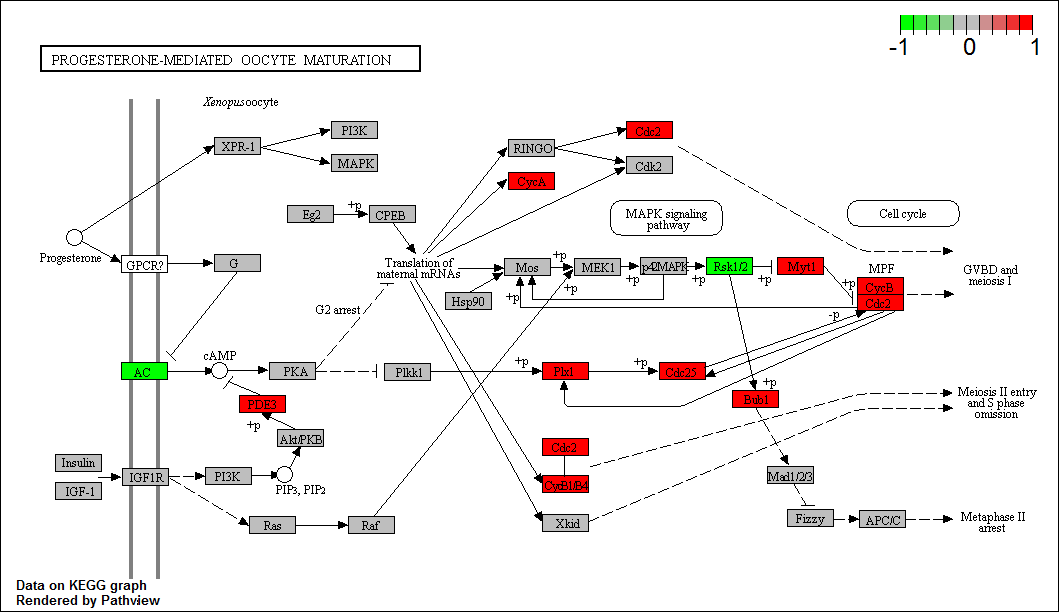

Supplement: DATASET S5 — GO-term analyses of control cultures (EGFP-expressing and no injury) in 3D versus 2D. [file Data_Sheet_5.ZIP › SD5_3D_vs_2D/GOstats/hsa04914.Progesterone-mediatedoocytematuration.png]

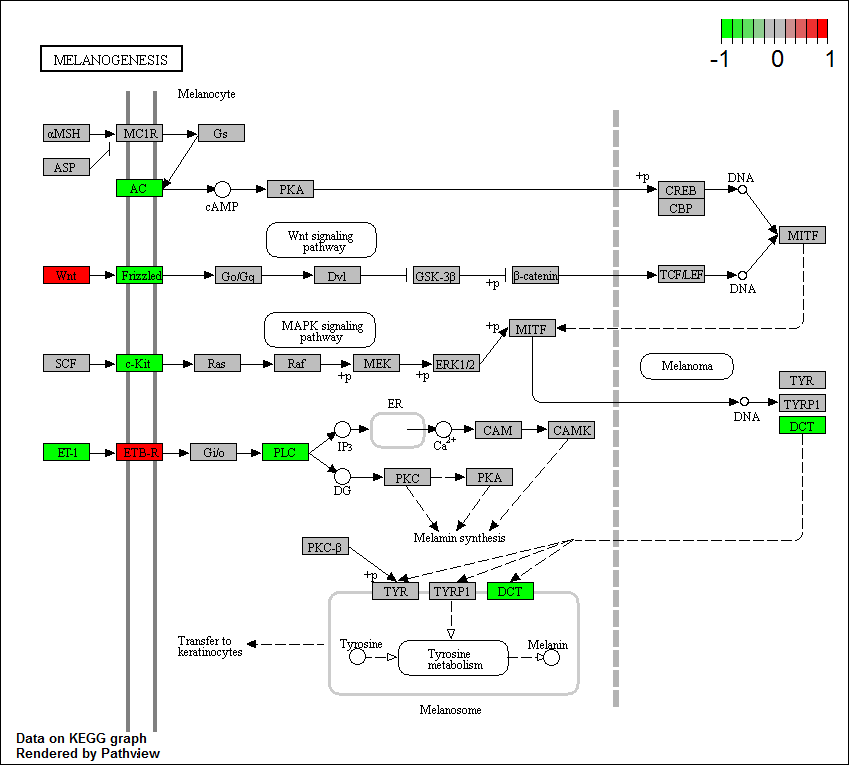

Supplement: DATASET S5 — GO-term analyses of control cultures (EGFP-expressing and no injury) in 3D versus 2D. [file Data_Sheet_5.ZIP › SD5_3D_vs_2D/GOstats/hsa04916.Melanogenesis.png]

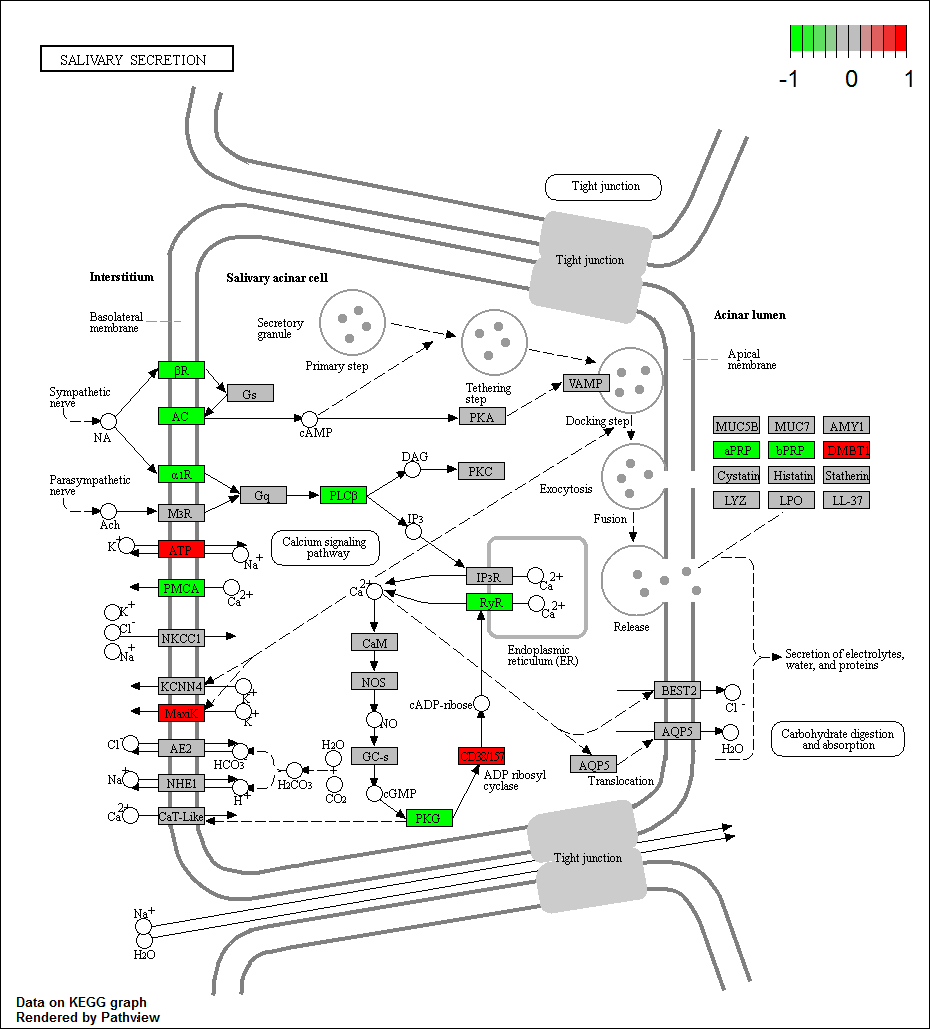

Supplement: DATASET S5 — GO-term analyses of control cultures (EGFP-expressing and no injury) in 3D versus 2D. [file Data_Sheet_5.ZIP › SD5_3D_vs_2D/GOstats/hsa04970.Salivarysecretion.png]

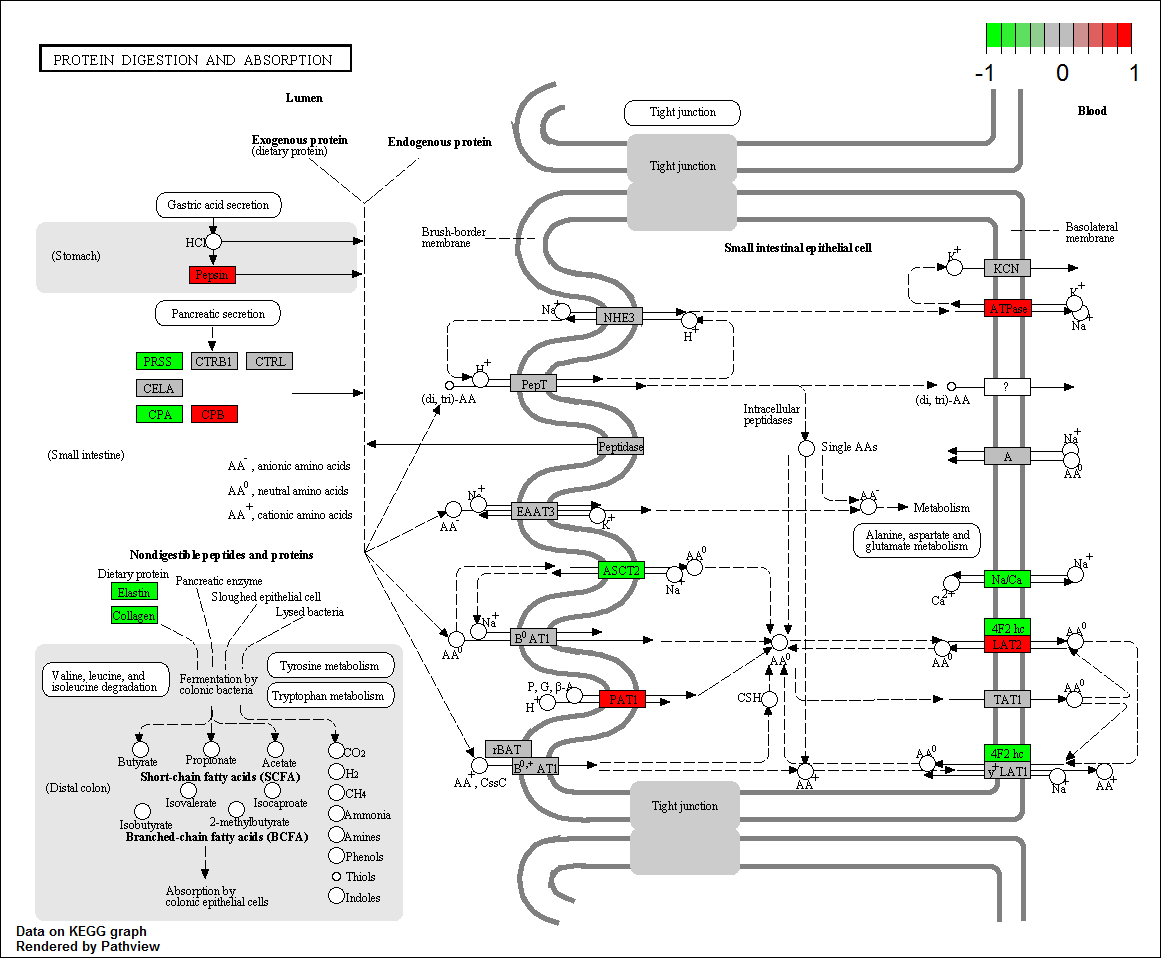

Supplement: DATASET S5 — GO-term analyses of control cultures (EGFP-expressing and no injury) in 3D versus 2D. [file Data_Sheet_5.ZIP › SD5_3D_vs_2D/GOstats/hsa04974.Proteindigestionandabsorption.png]

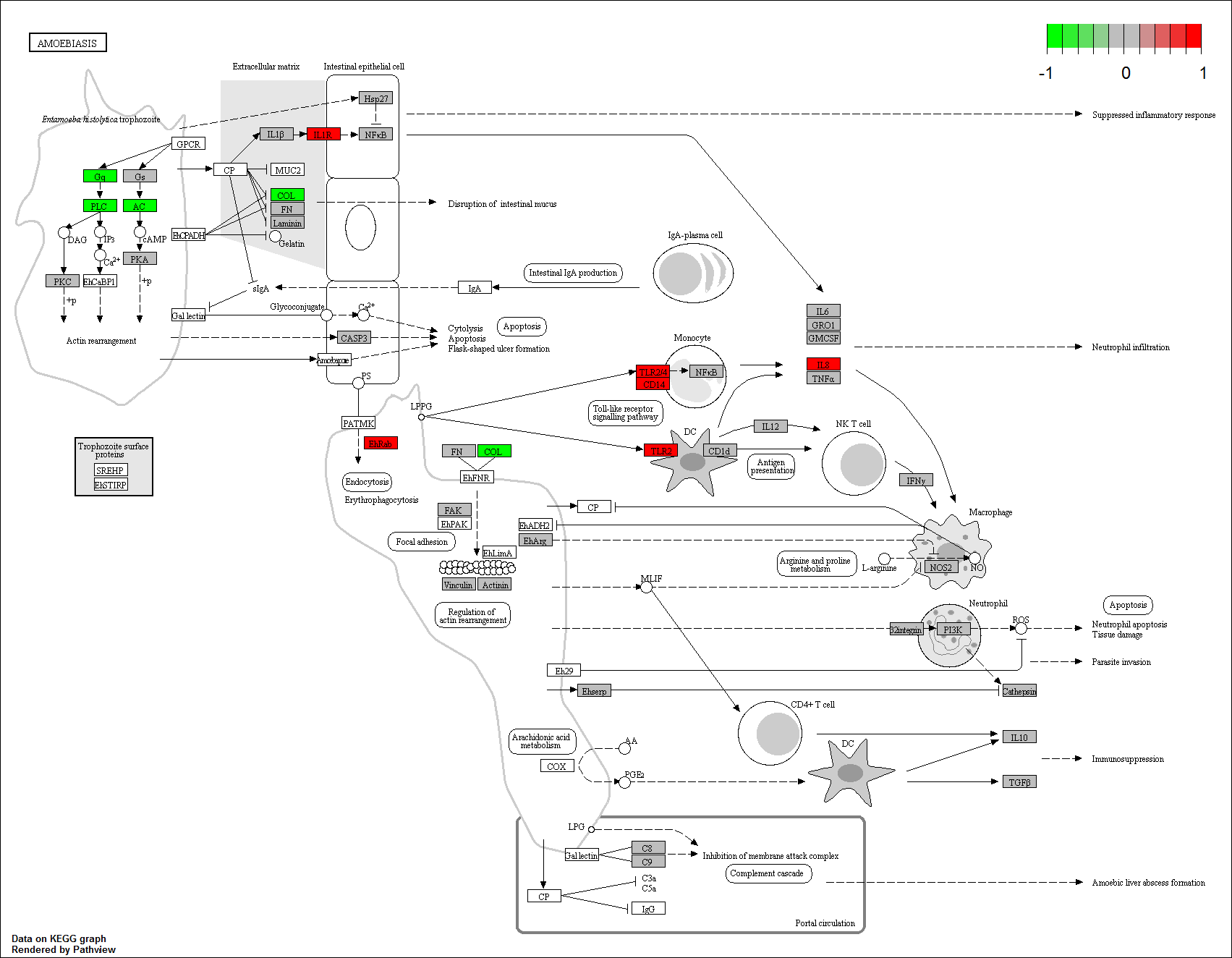

Supplement: DATASET S5 — GO-term analyses of control cultures (EGFP-expressing and no injury) in 3D versus 2D. [file Data_Sheet_5.ZIP › SD5_3D_vs_2D/GOstats/hsa05146.Amoebiasis.png]

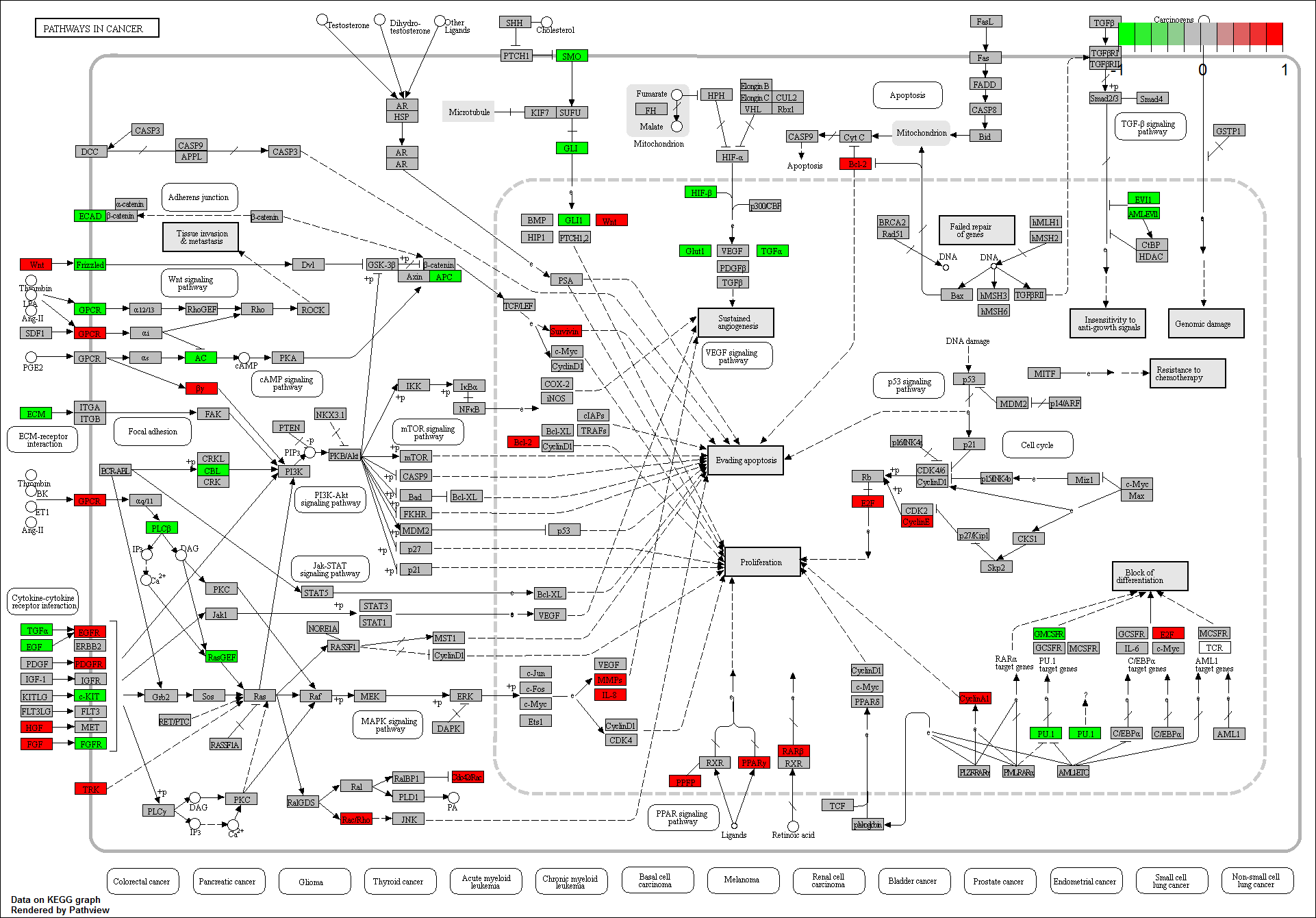

Supplement: DATASET S5 — GO-term analyses of control cultures (EGFP-expressing and no injury) in 3D versus 2D. [file Data_Sheet_5.ZIP › SD5_3D_vs_2D/GOstats/hsa05200.Pathwaysincancer.png]

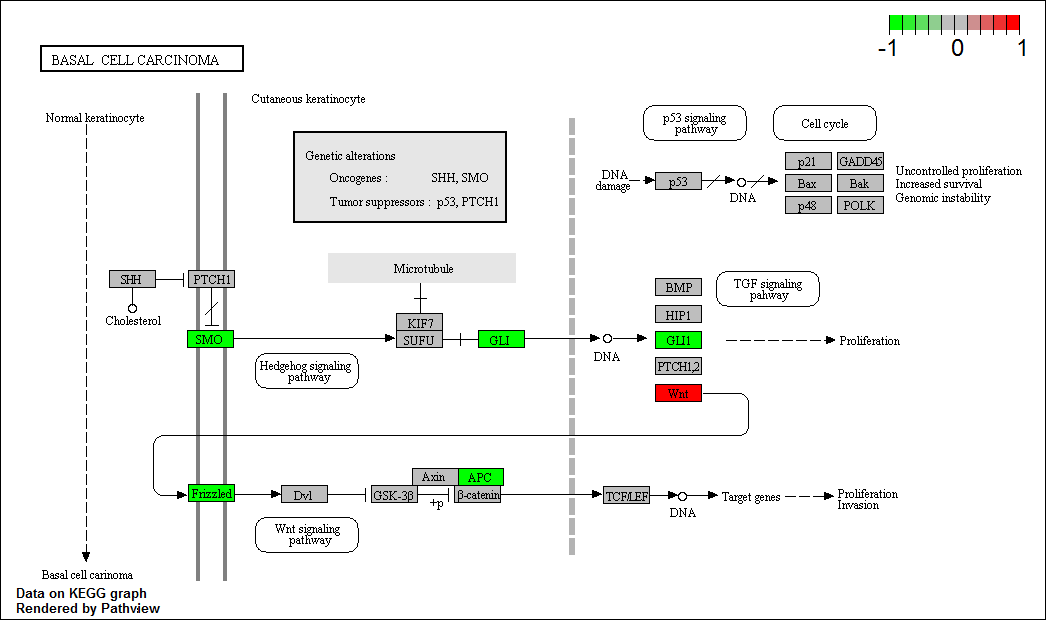

Supplement: DATASET S5 — GO-term analyses of control cultures (EGFP-expressing and no injury) in 3D versus 2D. [file Data_Sheet_5.ZIP › SD5_3D_vs_2D/GOstats/hsa05217.Basalcellcarcinoma.png]

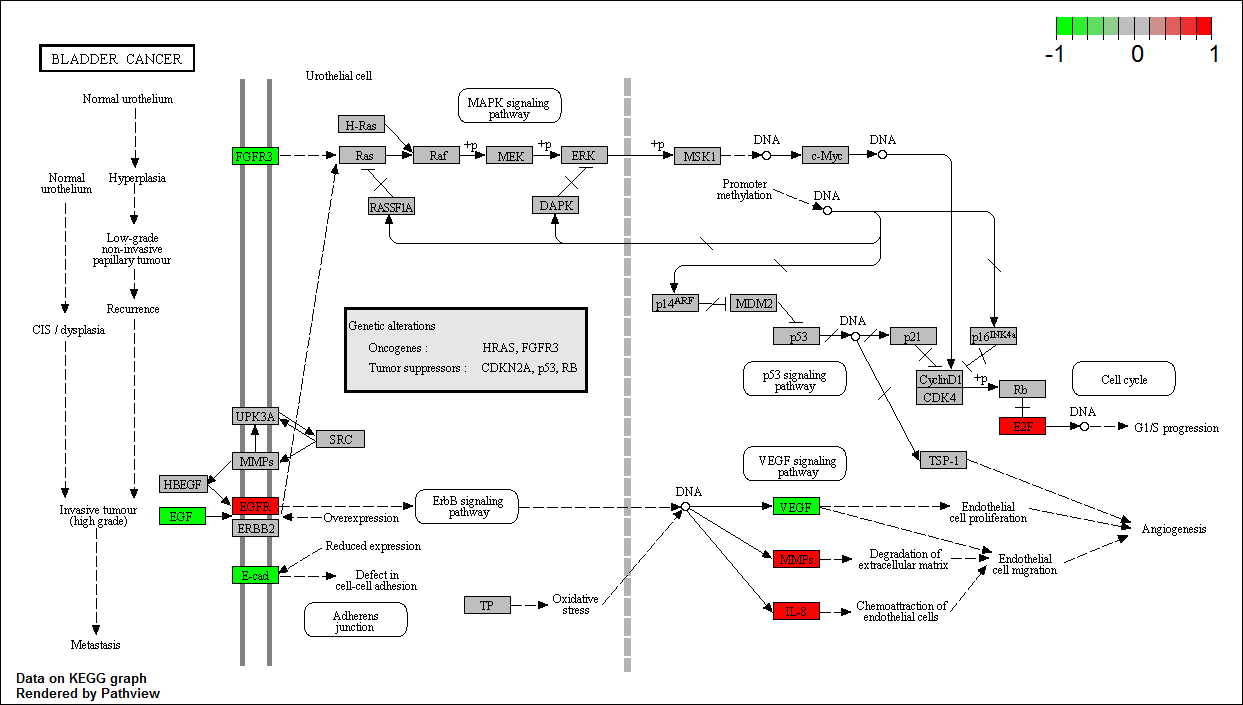

Supplement: DATASET S5 — GO-term analyses of control cultures (EGFP-expressing and no injury) in 3D versus 2D. [file Data_Sheet_5.ZIP › SD5_3D_vs_2D/GOstats/hsa05219.Bladdercancer.png]

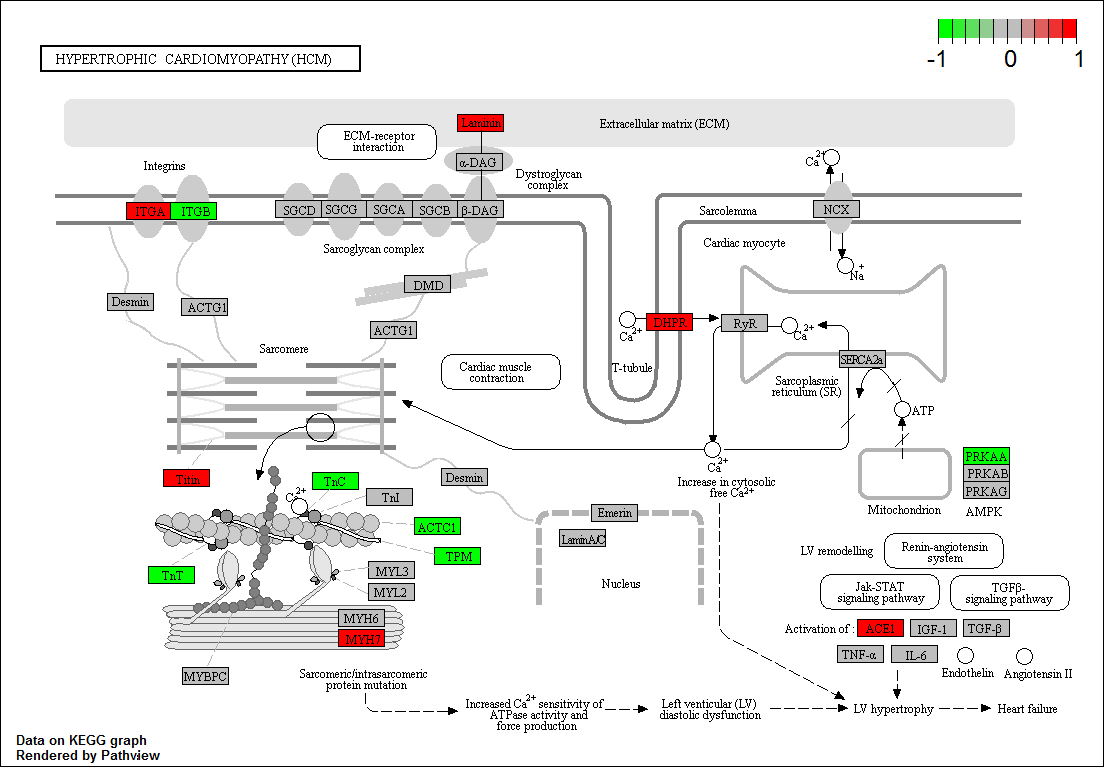

Supplement: DATASET S5 — GO-term analyses of control cultures (EGFP-expressing and no injury) in 3D versus 2D. [file Data_Sheet_5.ZIP › SD5_3D_vs_2D/GOstats/hsa05410.Hypertrophiccardiomyopathy(HCM).png]

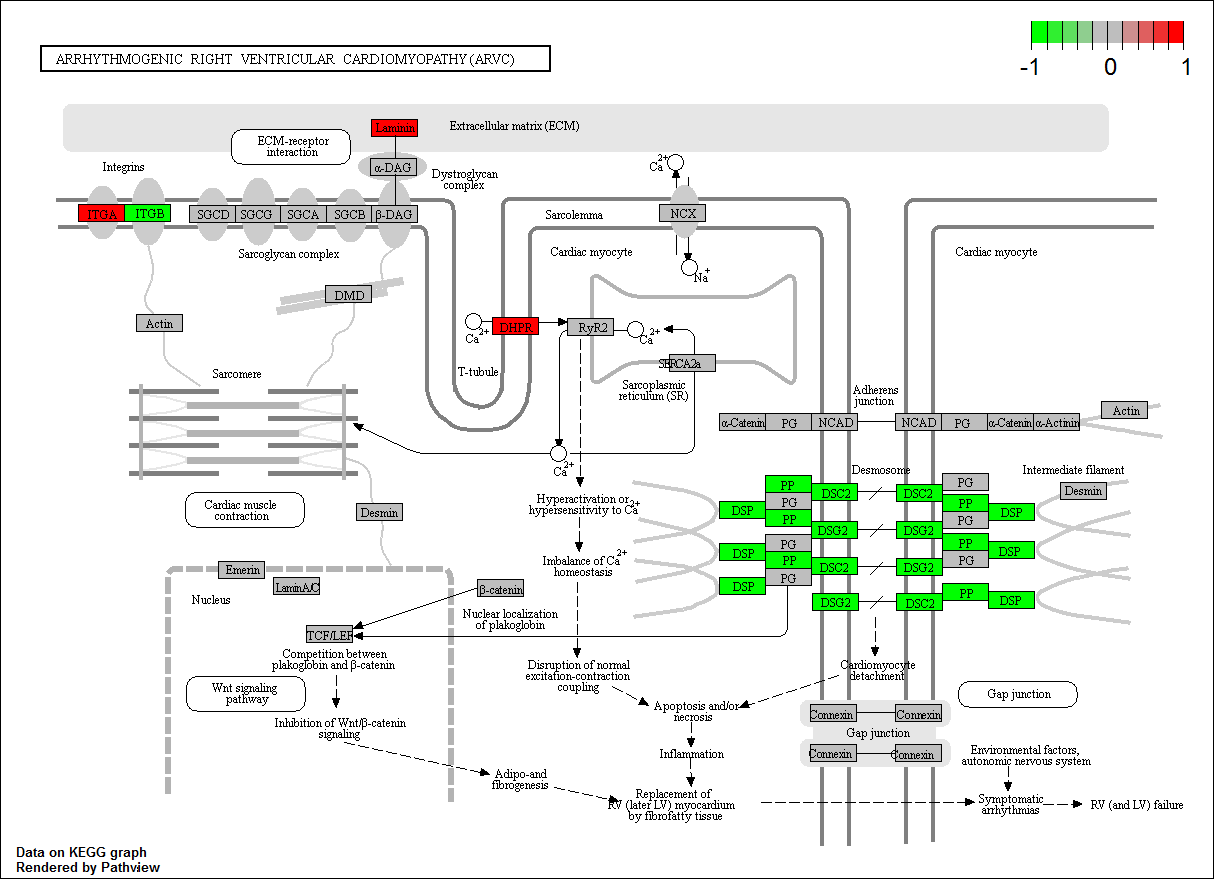

Supplement: DATASET S5 — GO-term analyses of control cultures (EGFP-expressing and no injury) in 3D versus 2D. [file Data_Sheet_5.ZIP › SD5_3D_vs_2D/GOstats/hsa05412.Arrhythmogenicrightventricularcardiomyopathy(ARVC).png]

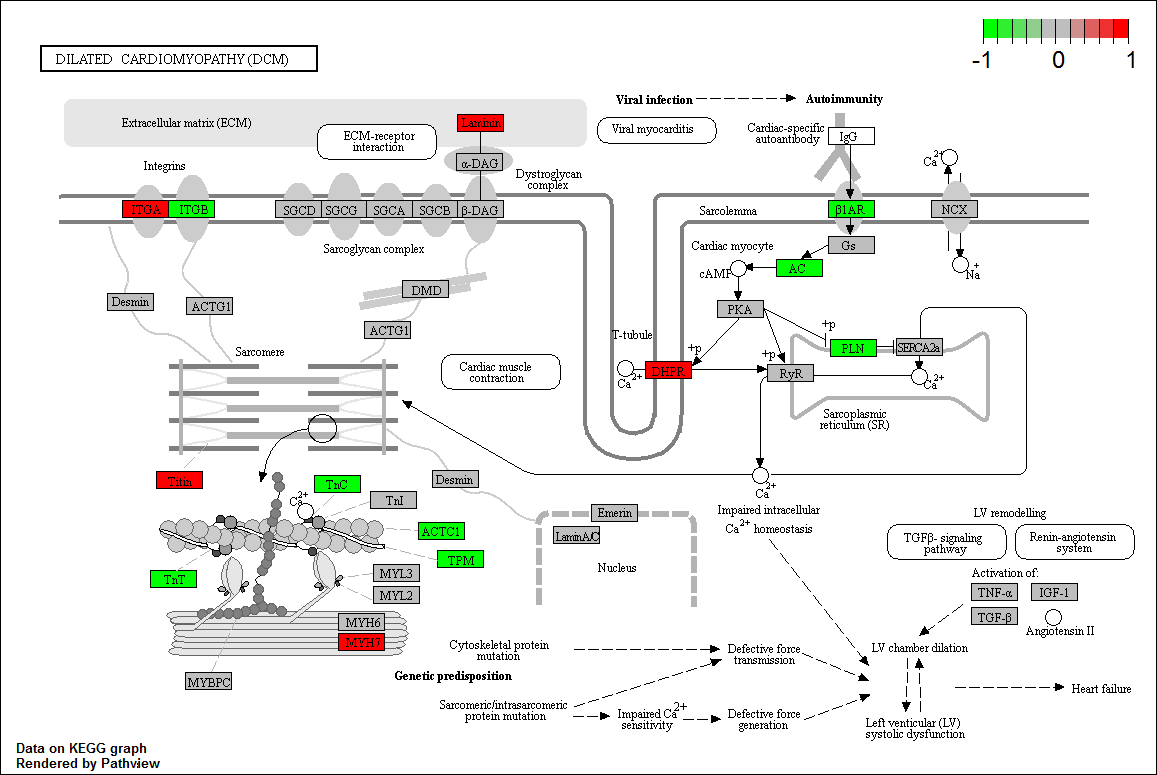

Supplement: DATASET S5 — GO-term analyses of control cultures (EGFP-expressing and no injury) in 3D versus 2D. [file Data_Sheet_5.ZIP › SD5_3D_vs_2D/GOstats/hsa05414.Dilatedcardiomyopathy.png]

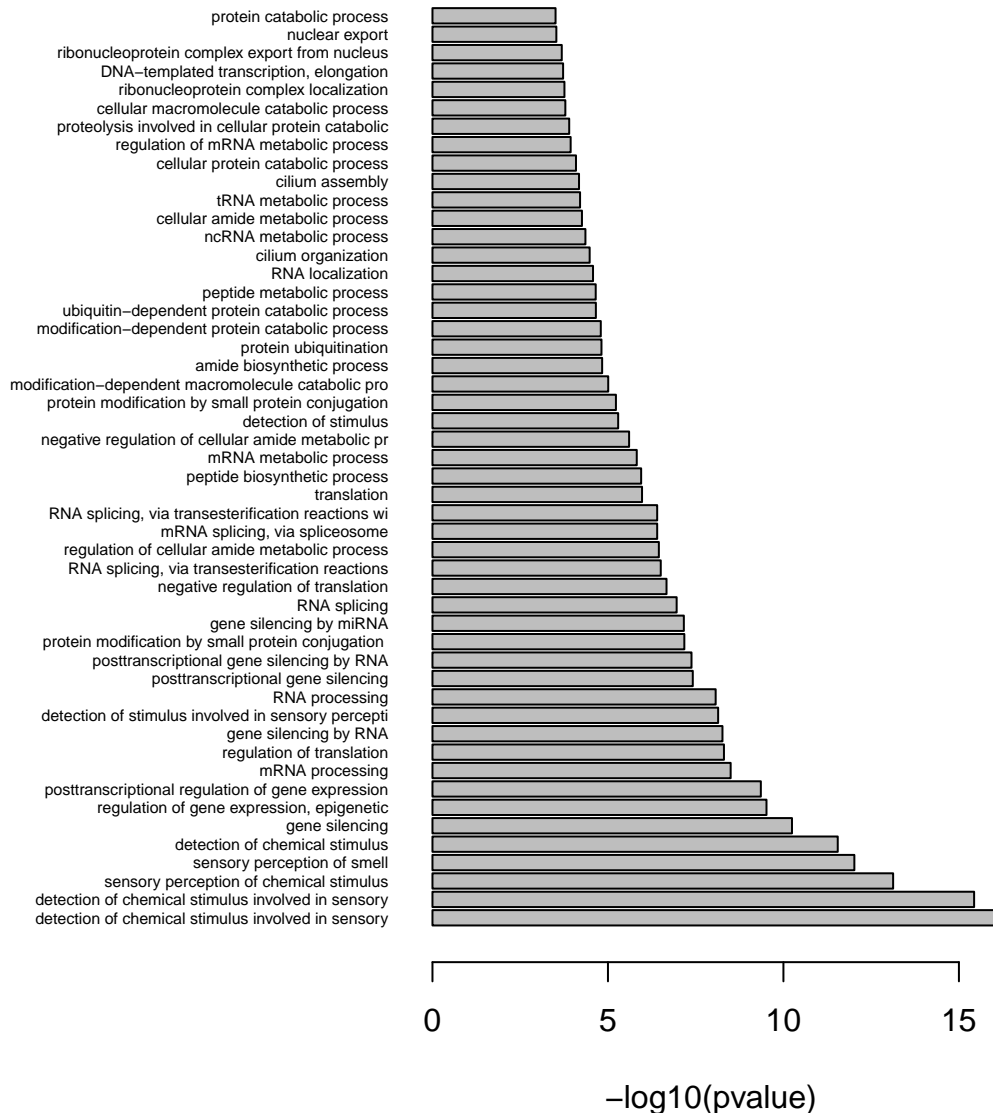

Supplement: DATASET S5 — GO-term analyses of control cultures (EGFP-expressing and no injury) in 3D versus 2D. [file Data_Sheet_5.ZIP › SD5_3D_vs_2D/GOstats/pVal_GOstats_BP_Down_pieChart.pdf]

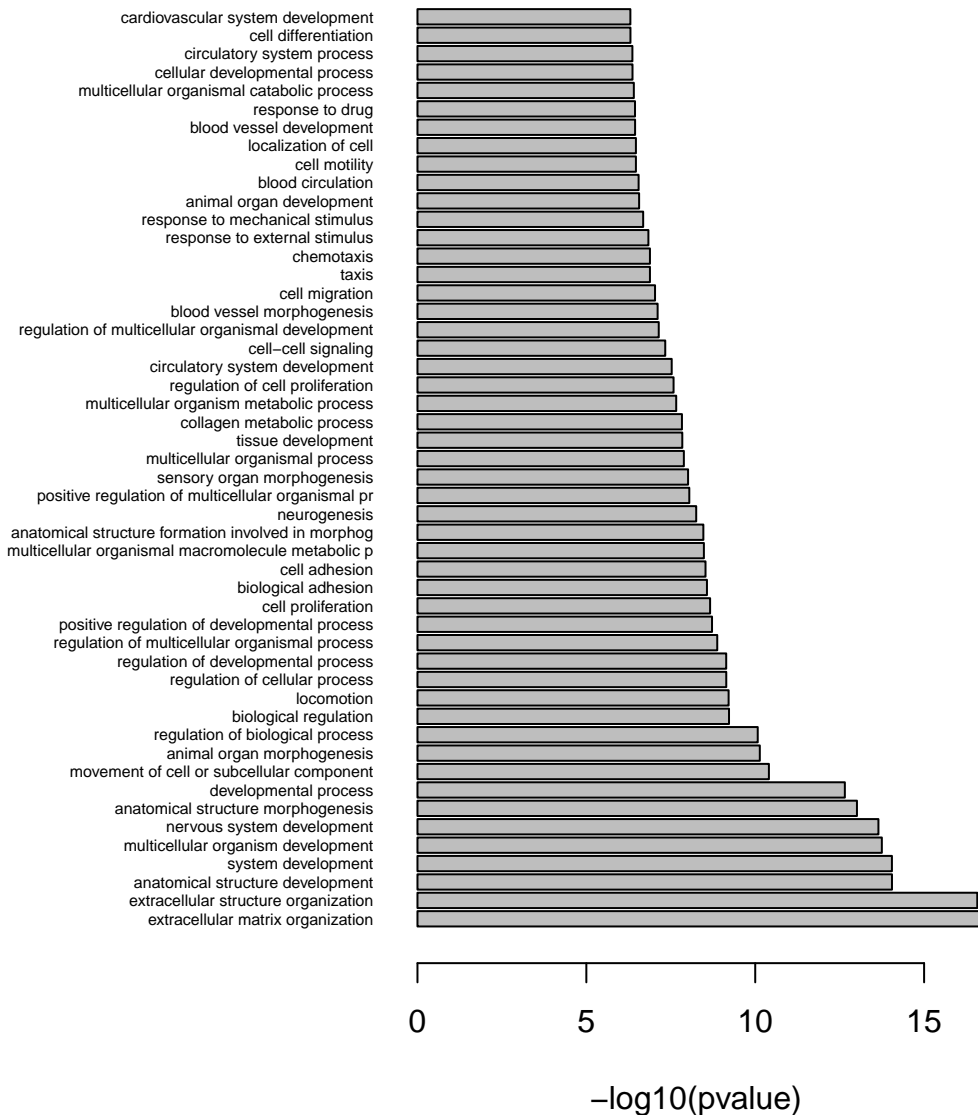

Supplement: DATASET S5 — GO-term analyses of control cultures (EGFP-expressing and no injury) in 3D versus 2D. [file Data_Sheet_5.ZIP › SD5_3D_vs_2D/GOstats/pVal_GOstats_BP_Up_pieChart.pdf]

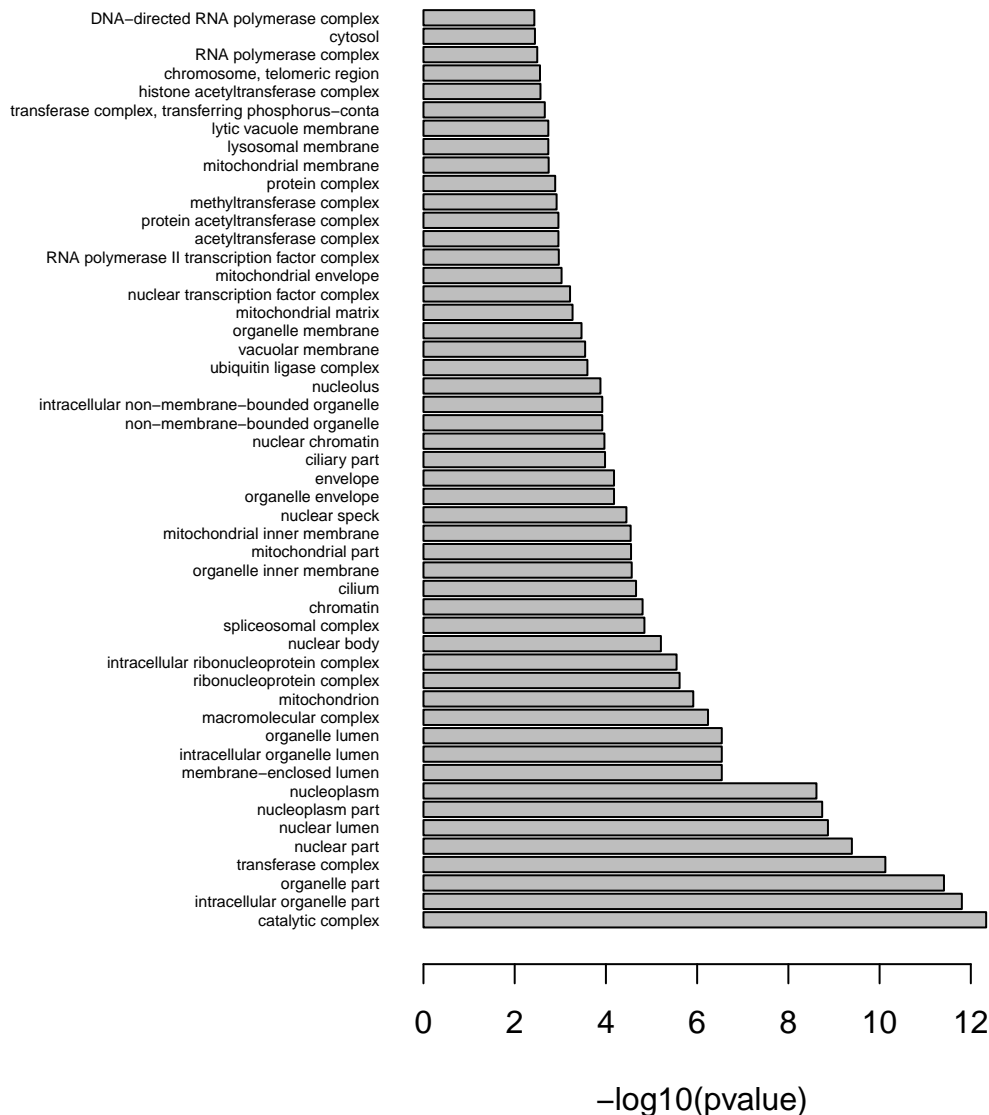

Supplement: DATASET S5 — GO-term analyses of control cultures (EGFP-expressing and no injury) in 3D versus 2D. [file Data_Sheet_5.ZIP › SD5_3D_vs_2D/GOstats/pVal_GOstats_CC_Down_pieChart.pdf]

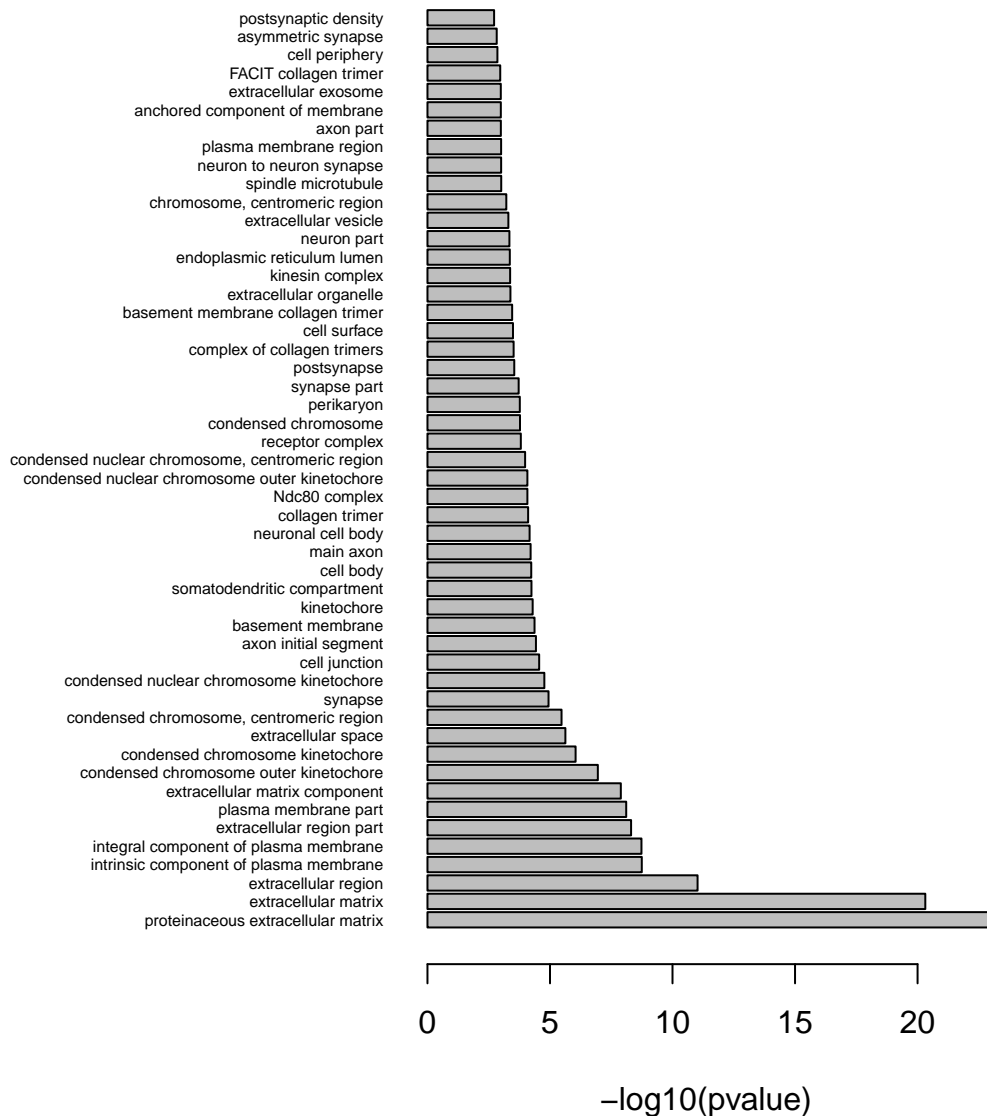

Supplement: DATASET S5 — GO-term analyses of control cultures (EGFP-expressing and no injury) in 3D versus 2D. [file Data_Sheet_5.ZIP › SD5_3D_vs_2D/GOstats/pVal_GOstats_CC_Up_pieChart.pdf]

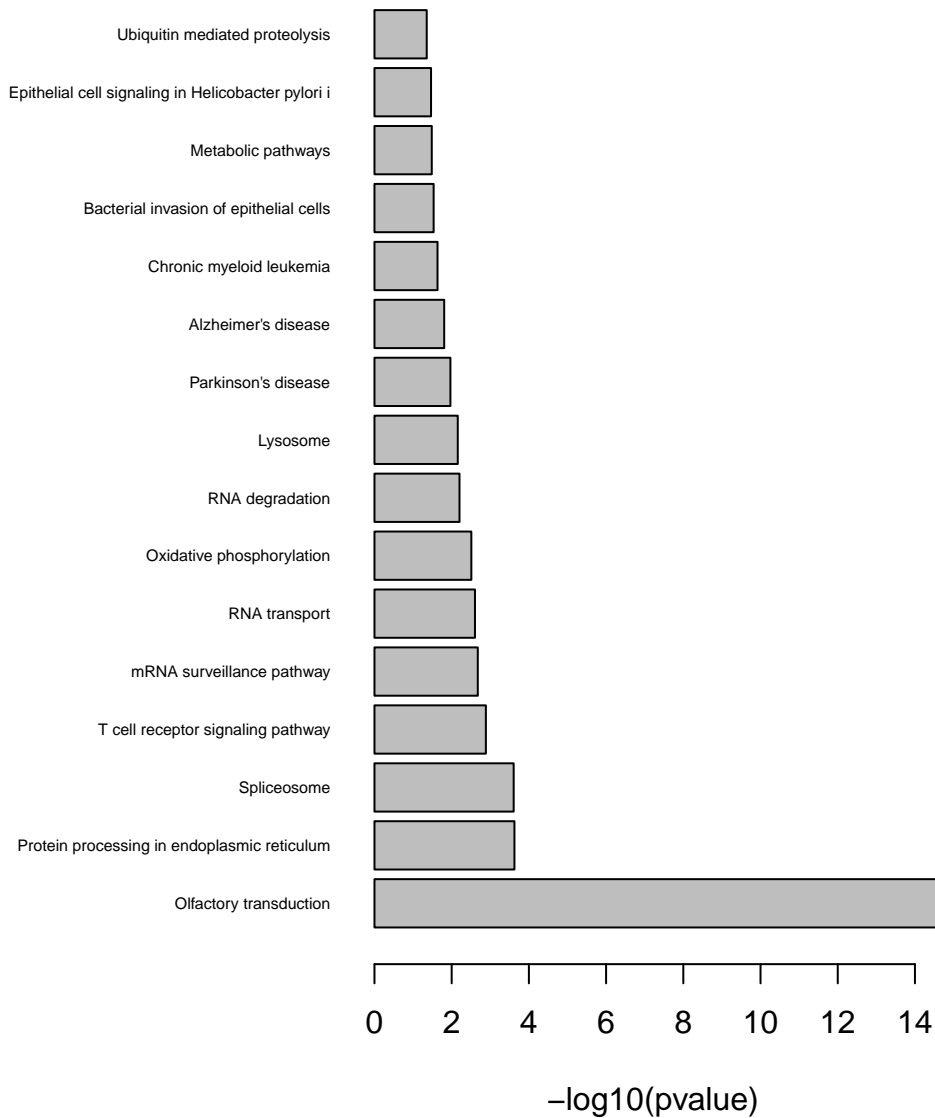

Supplement: DATASET S5 — GO-term analyses of control cultures (EGFP-expressing and no injury) in 3D versus 2D. [file Data_Sheet_5.ZIP › SD5_3D_vs_2D/GOstats/pVal_GOstats_kegg_Under.pdf]

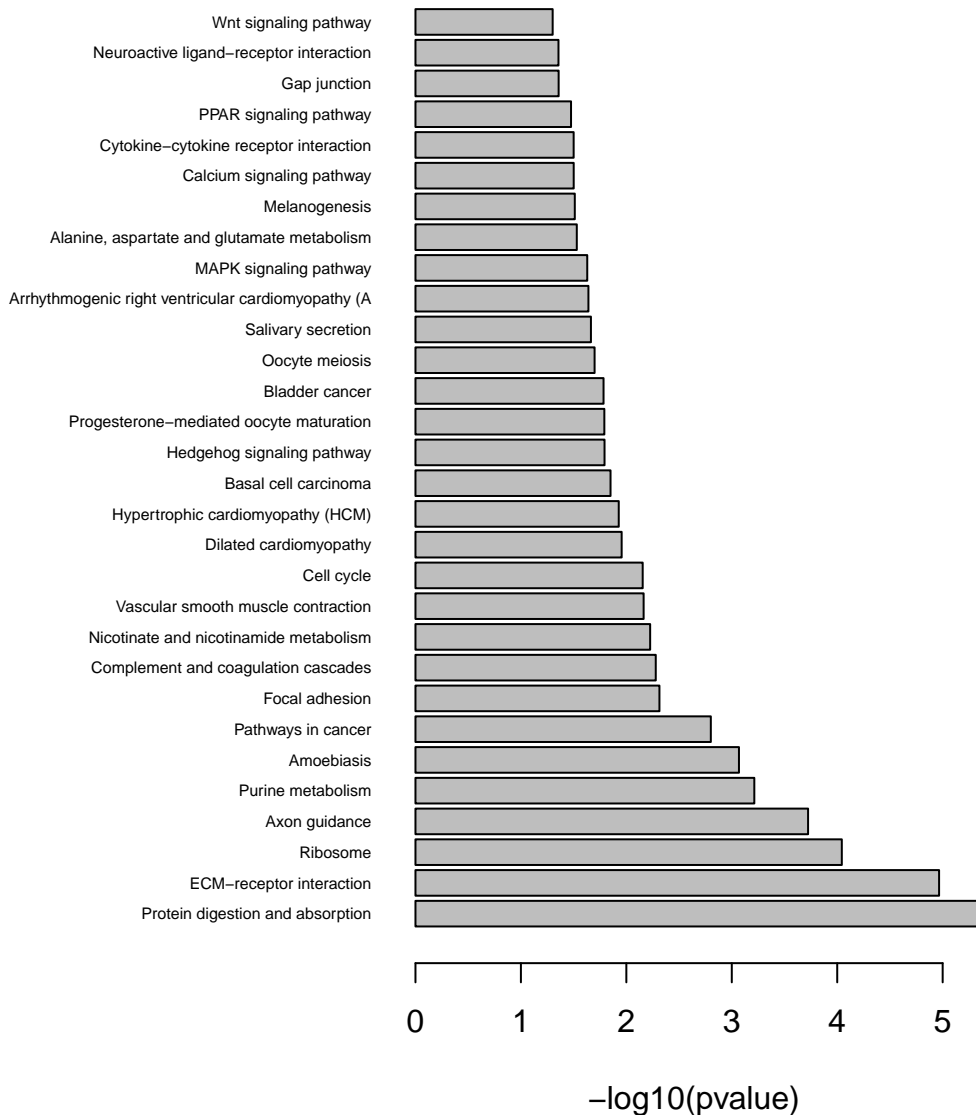

Supplement: DATASET S5 — GO-term analyses of control cultures (EGFP-expressing and no injury) in 3D versus 2D. [file Data_Sheet_5.ZIP › SD5_3D_vs_2D/GOstats/pVal_GOstats_kegg_Up.pdf]

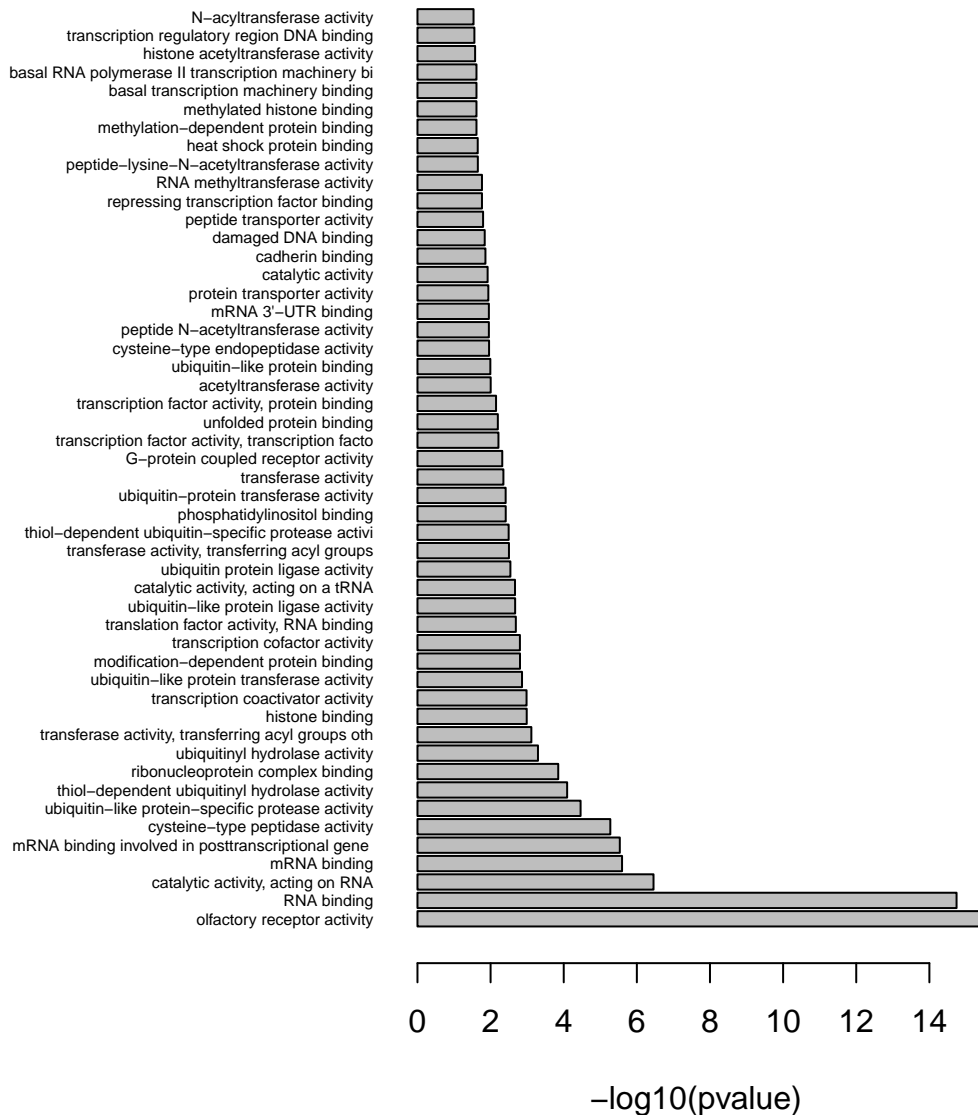

Supplement: DATASET S5 — GO-term analyses of control cultures (EGFP-expressing and no injury) in 3D versus 2D. [file Data_Sheet_5.ZIP › SD5_3D_vs_2D/GOstats/pVal_GOstats_MF_Down_pieChart.pdf]

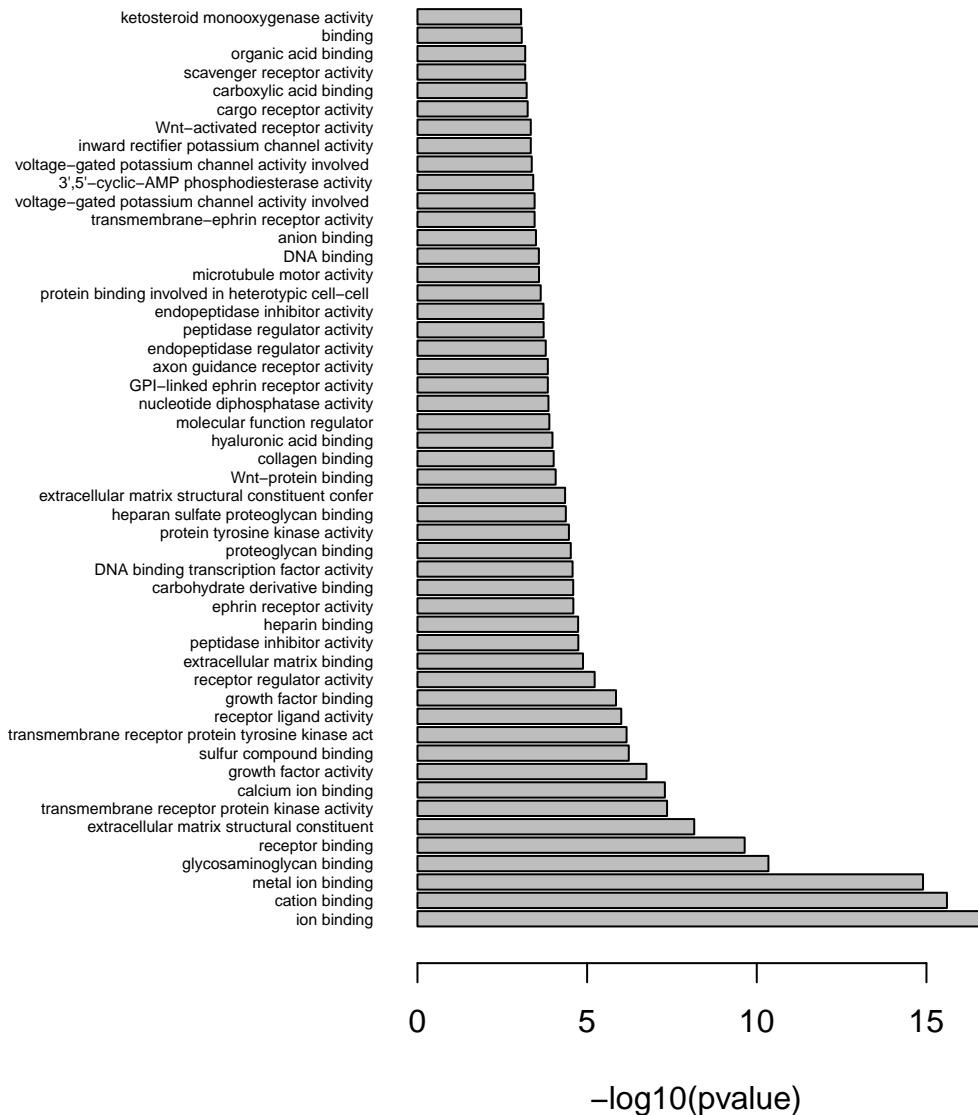

Supplement: DATASET S5 — GO-term analyses of control cultures (EGFP-expressing and no injury) in 3D versus 2D. [file Data_Sheet_5.ZIP › SD5_3D_vs_2D/GOstats/pVal_GOstats_MF_Up_pieChart.pdf]

# Gata3\_LN\_vs\_GFP\_LN

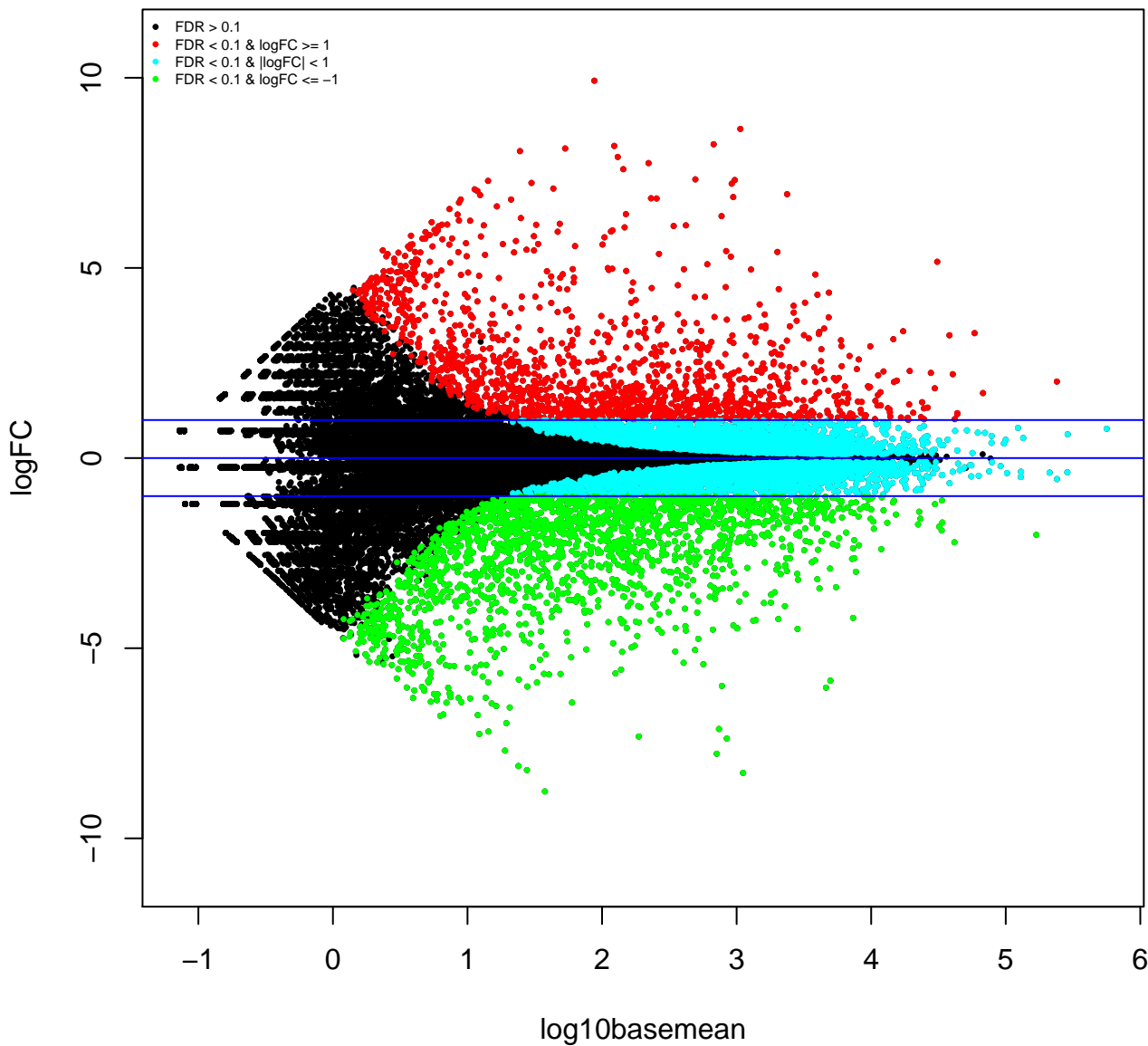

Supplement: DATASET S6 — GO-term analyses of GATA3-expressing versus EGFP-expressing unlesioned pHAs in 3D. [file Data_Sheet_6.ZIP › SD6_GATA3_vs_GFP_LN/Gata3_LN_vs_GFP_LN_MAplot.pdf]

# Gata3\_LN\_vs\_GFP\_LN

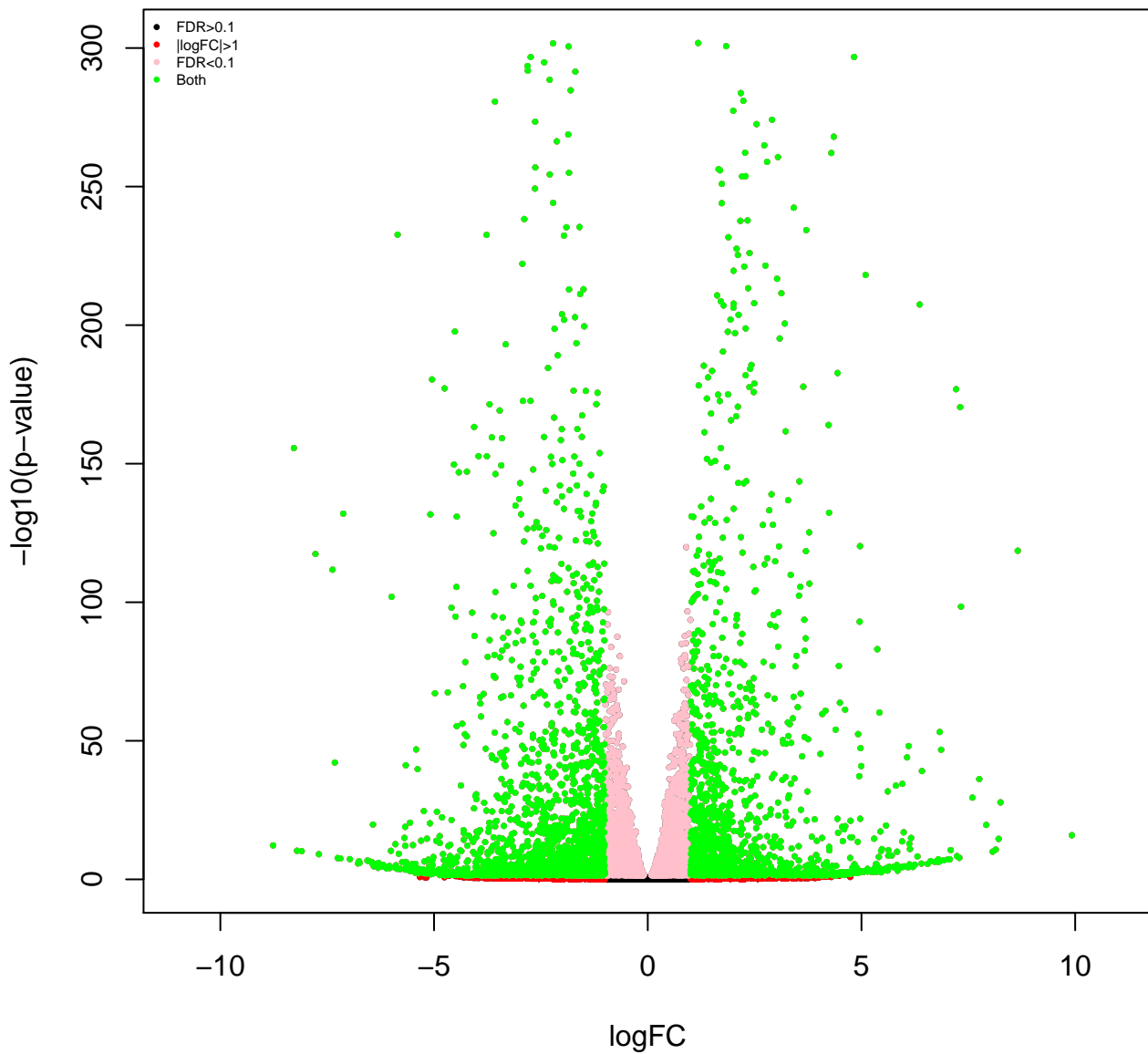

Supplement: DATASET S6 — GO-term analyses of GATA3-expressing versus EGFP-expressing unlesioned pHAs in 3D. [file Data_Sheet_6.ZIP › SD6_GATA3_vs_GFP_LN/Gata3_LN_vs_GFP_LN_Volcanoplot.pdf]

GOstats\_BP\_Down\_pieChart

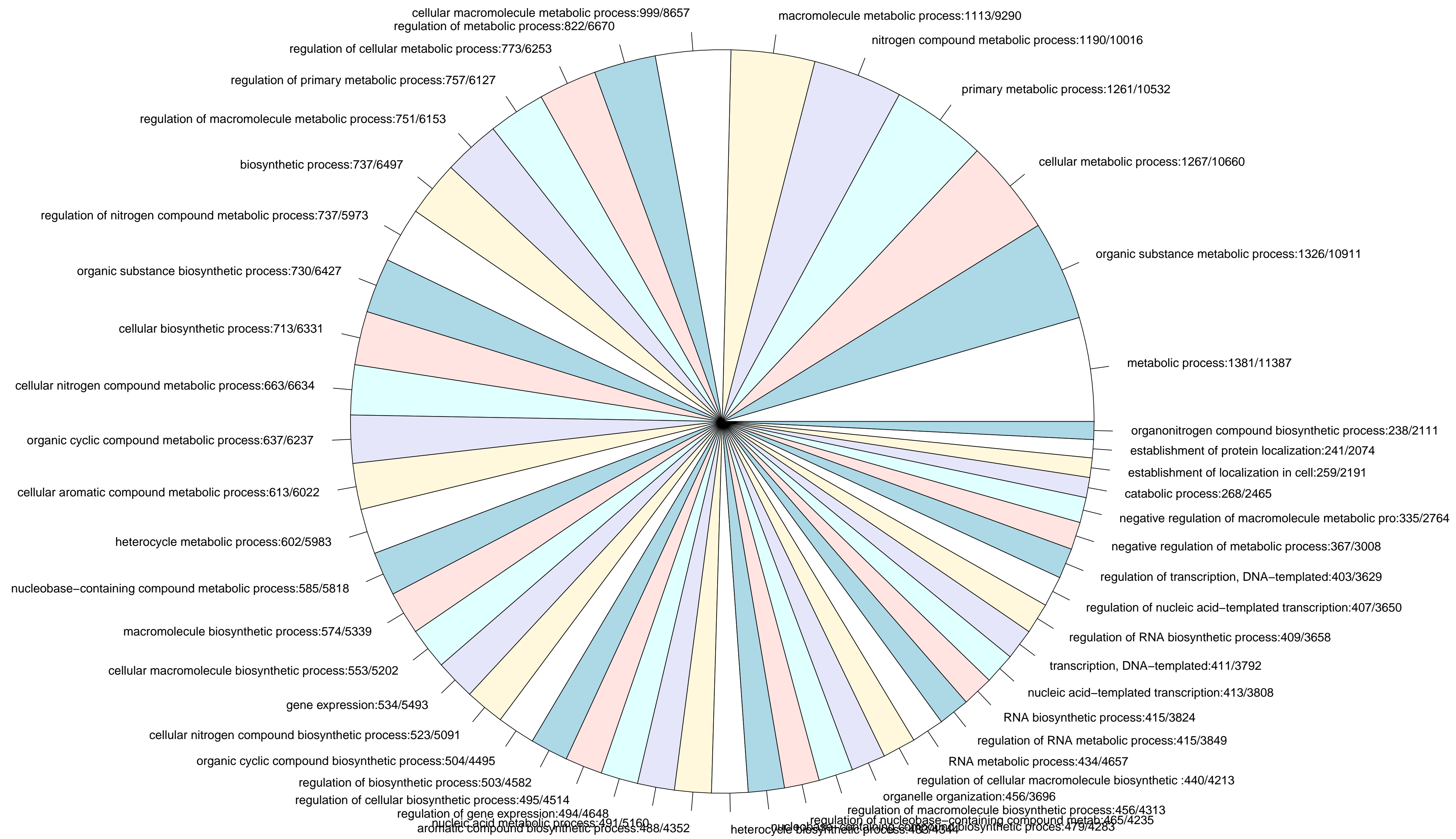

Supplement: DATASET S6 — GO-term analyses of GATA3-expressing versus EGFP-expressing unlesioned pHAs in 3D. [file Data_Sheet_6.ZIP › SD6_GATA3_vs_GFP_LN/GOstats/GOstats_BP_Down_pieChart.pdf]

GOstats\_BP\_Up\_pieChart

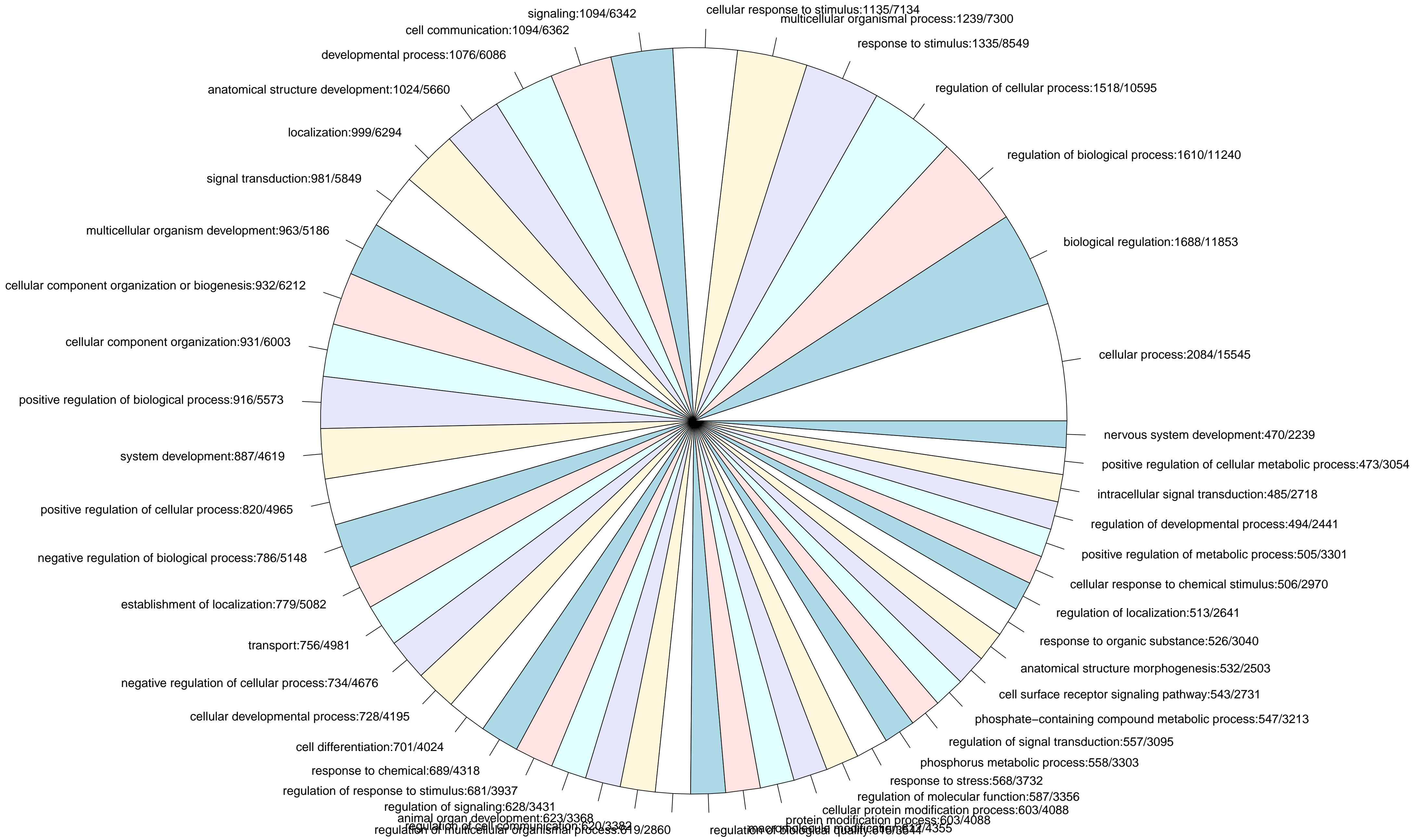

Supplement: DATASET S6 — GO-term analyses of GATA3-expressing versus EGFP-expressing unlesioned pHAs in 3D. [file Data_Sheet_6.ZIP › SD6_GATA3_vs_GFP_LN/GOstats/GOstats_BP_Up_pieChart.pdf]

GOstats\_CC\_Down\_pieChart

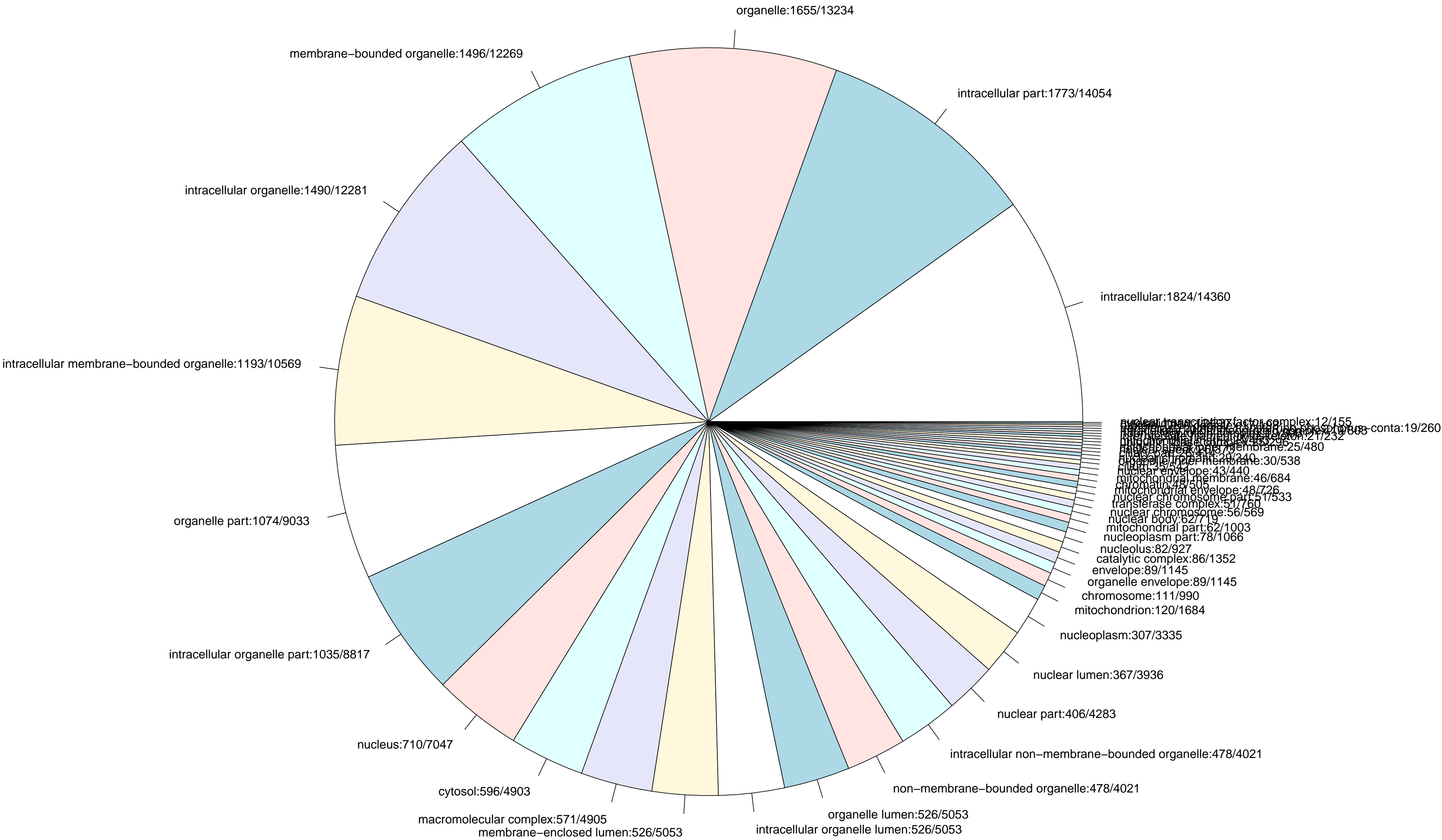

Supplement: DATASET S6 — GO-term analyses of GATA3-expressing versus EGFP-expressing unlesioned pHAs in 3D. [file Data_Sheet_6.ZIP › SD6_GATA3_vs_GFP_LN/GOstats/GOstats_CC_Down_pieChart.pdf]

### GOstats\_CC\_Up\_pieChart

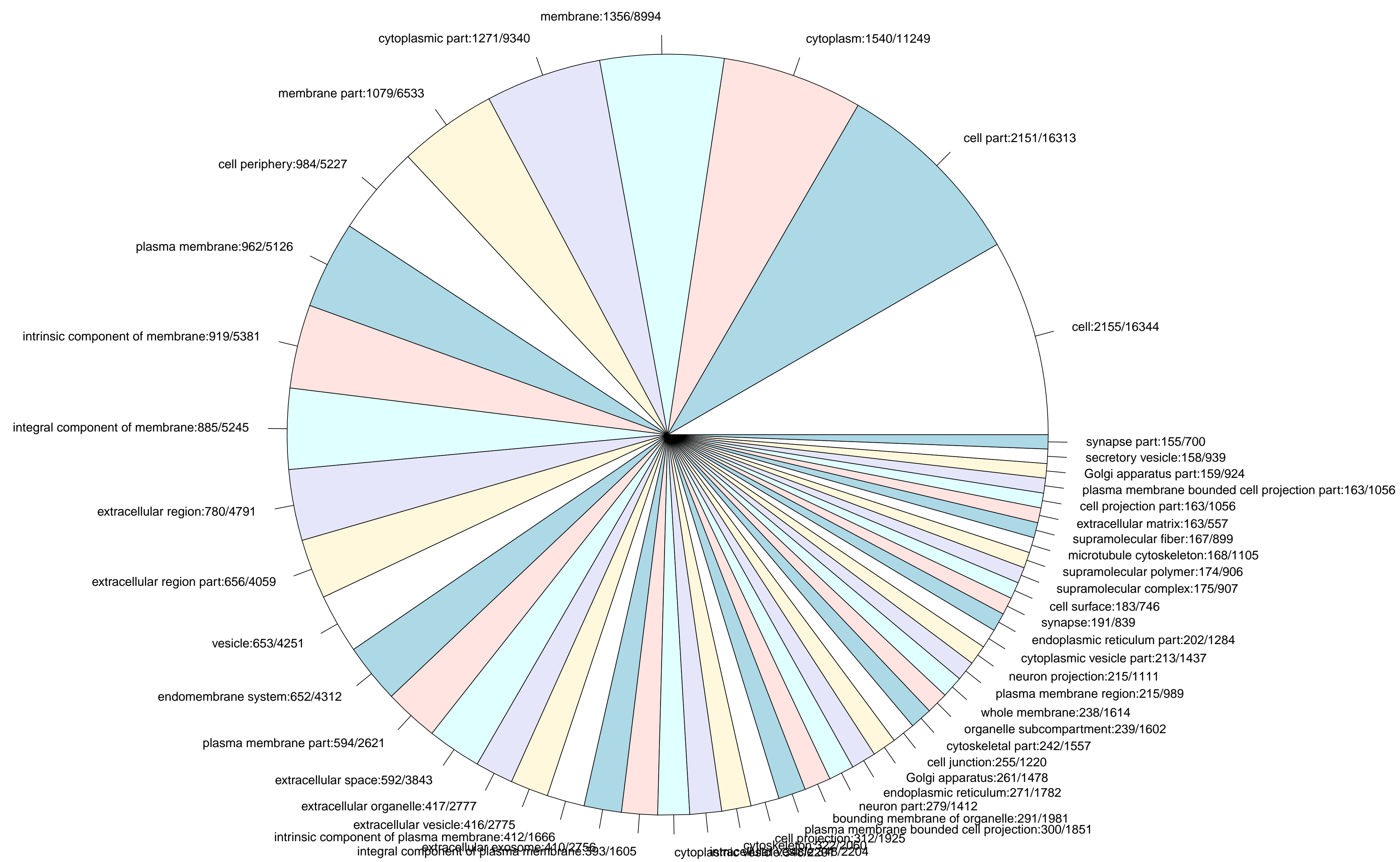

Supplement: DATASET S6 — GO-term analyses of GATA3-expressing versus EGFP-expressing unlesioned pHAs in 3D. [file Data_Sheet_6.ZIP › SD6_GATA3_vs_GFP_LN/GOstats/GOstats_CC_Up_pieChart.pdf]

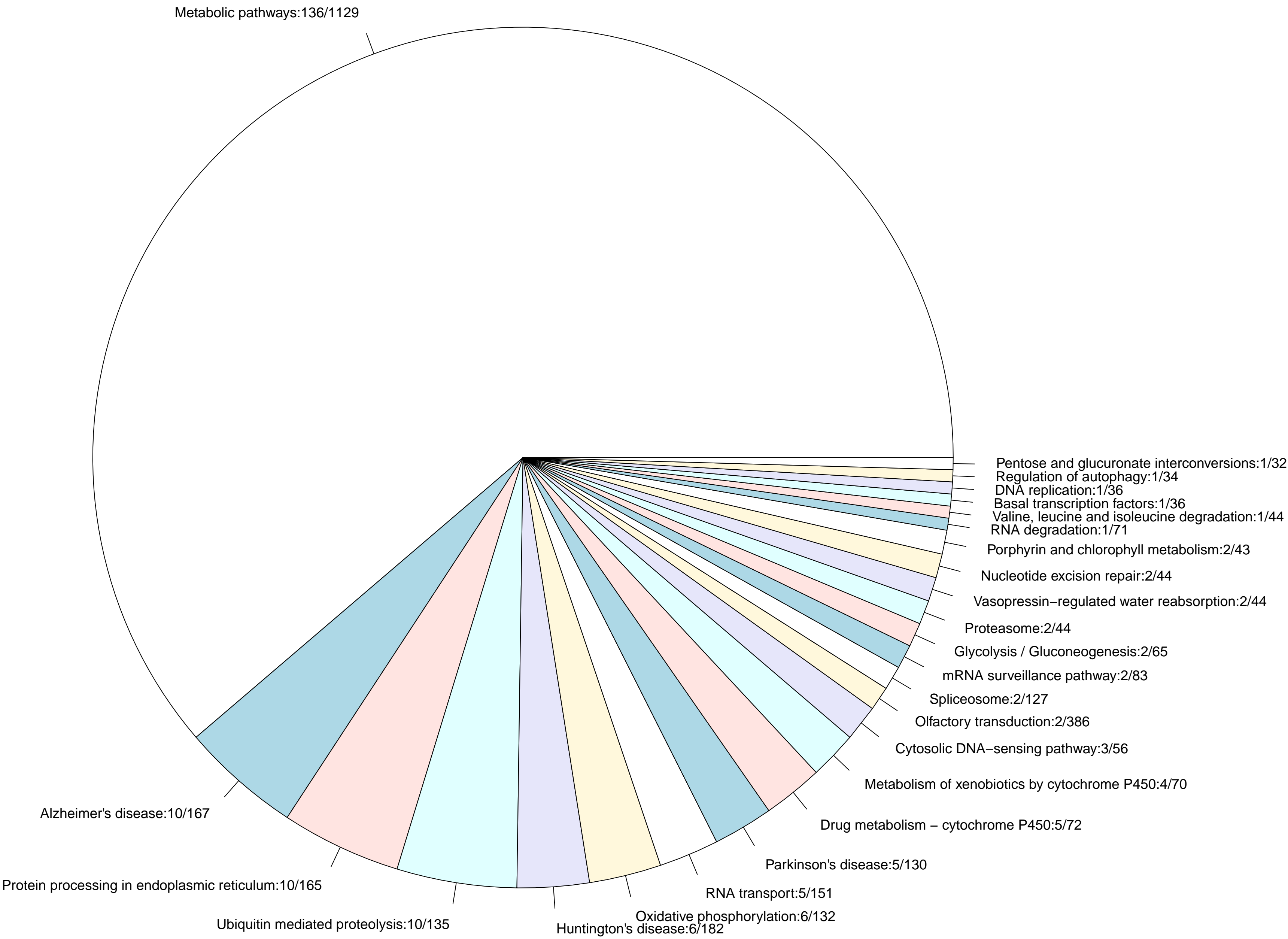

Supplement: DATASET S6 — GO-term analyses of GATA3-expressing versus EGFP-expressing unlesioned pHAs in 3D. [file Data_Sheet_6.ZIP › SD6_GATA3_vs_GFP_LN/GOstats/GOstats_kegg_Under.pdf]

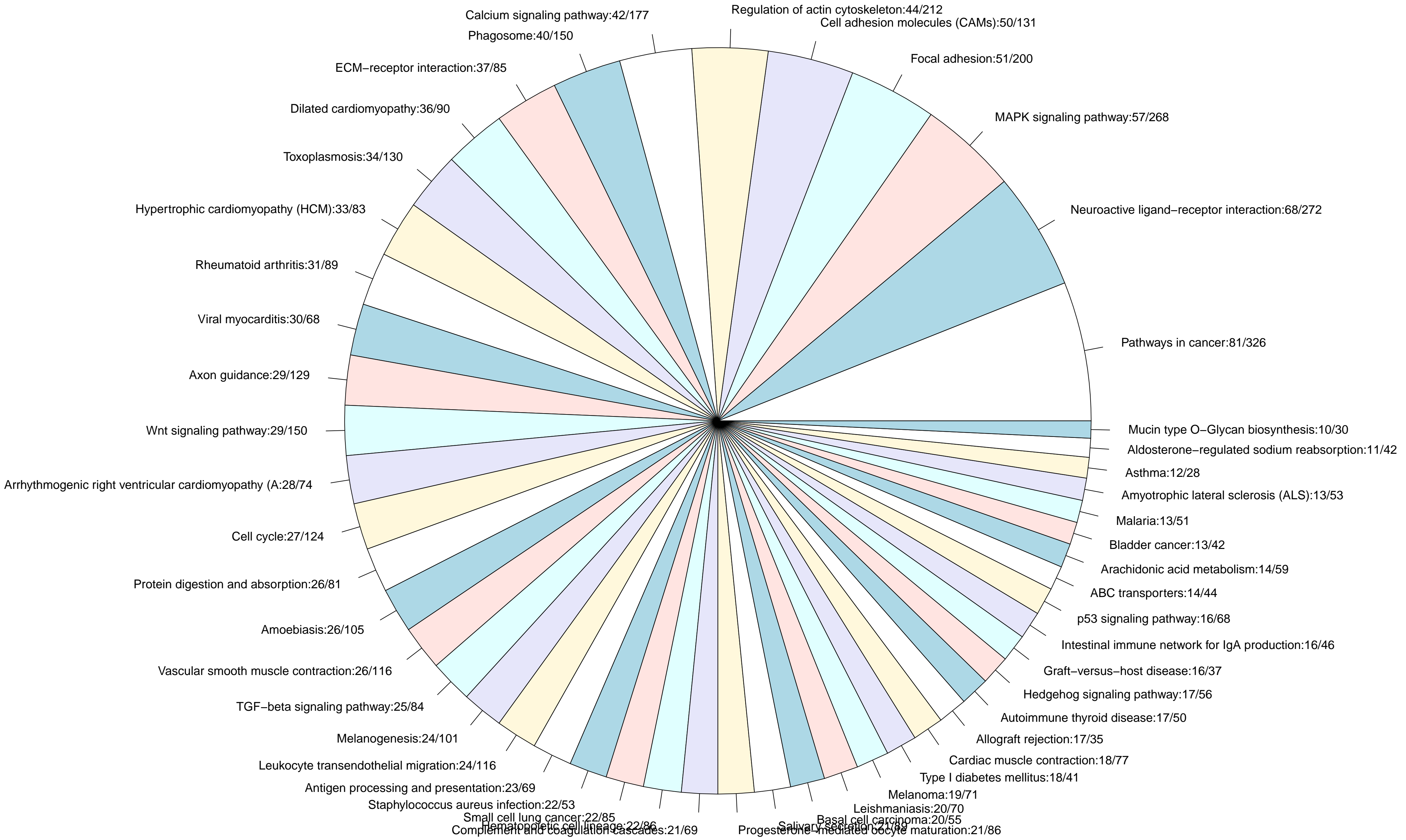

Supplement: DATASET S6 — GO-term analyses of GATA3-expressing versus EGFP-expressing unlesioned pHAs in 3D. [file Data_Sheet_6.ZIP › SD6_GATA3_vs_GFP_LN/GOstats/GOstats_kegg_Up.pdf]

### GOstats\_MF\_Down\_pieChart

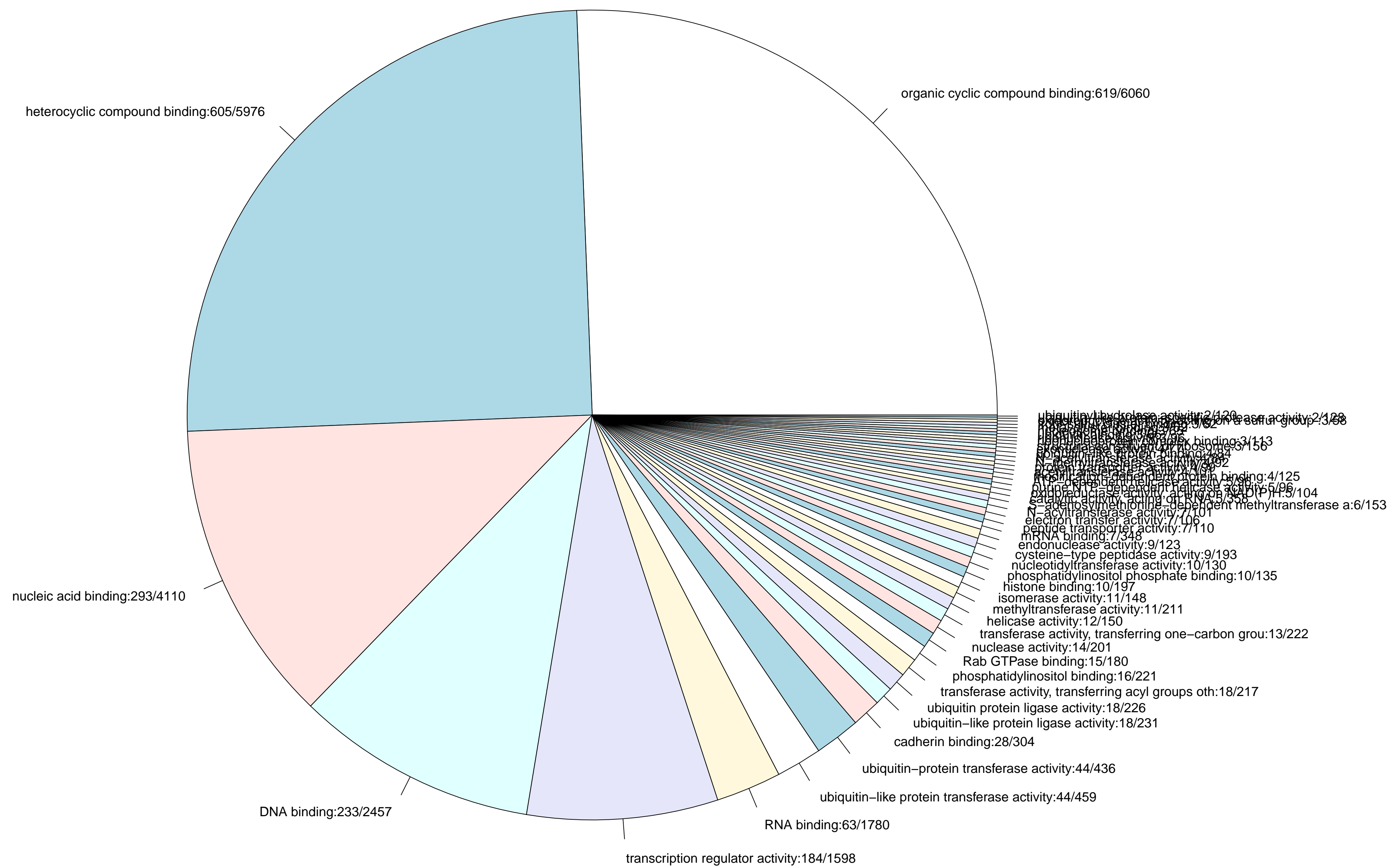

Supplement: DATASET S6 — GO-term analyses of GATA3-expressing versus EGFP-expressing unlesioned pHAs in 3D. [file Data_Sheet_6.ZIP › SD6_GATA3_vs_GFP_LN/GOstats/GOstats_MF_Down_pieChart.pdf]

GOstats\_MF\_Up\_pieChart

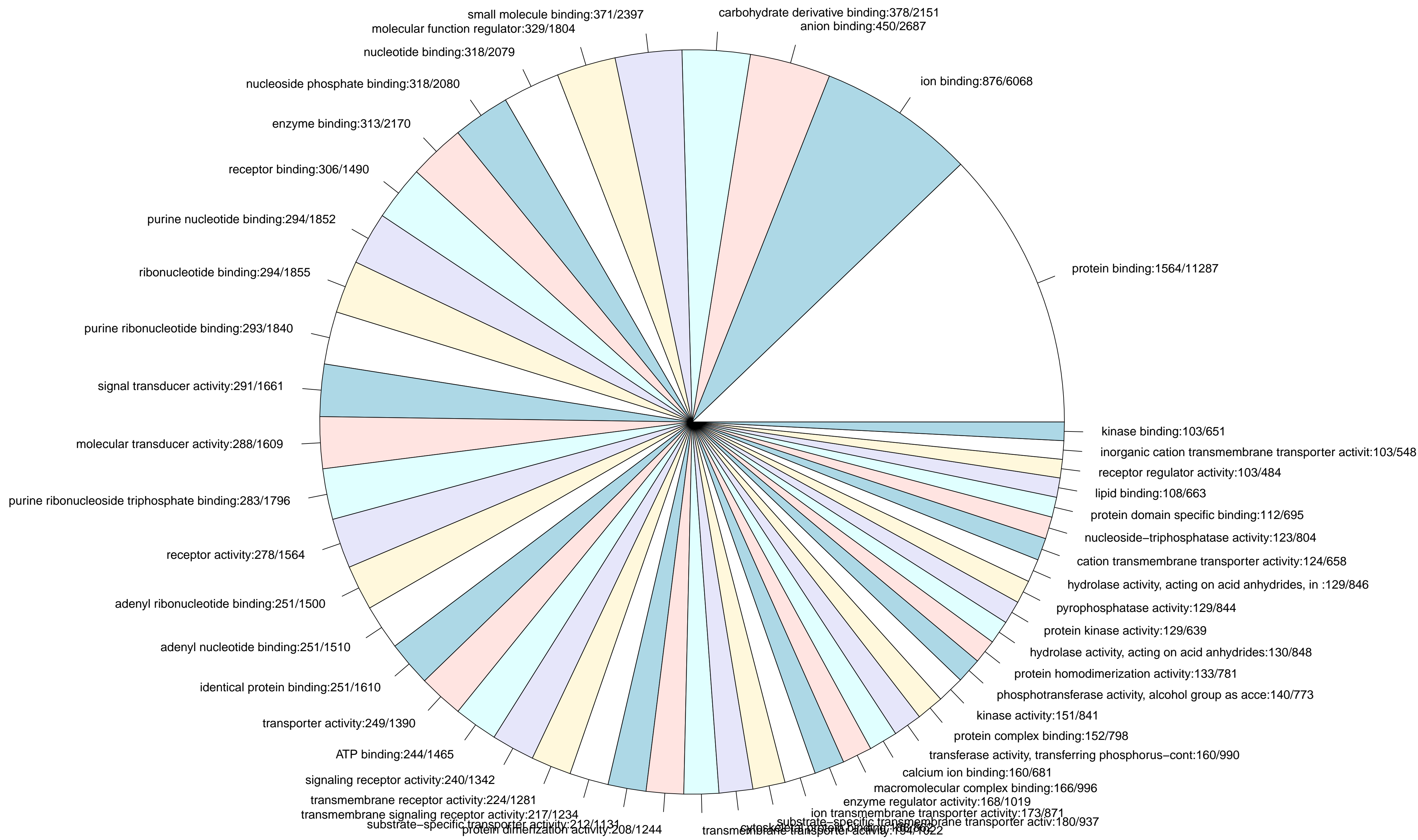

Supplement: DATASET S6 — GO-term analyses of GATA3-expressing versus EGFP-expressing unlesioned pHAs in 3D. [file Data_Sheet_6.ZIP › SD6_GATA3_vs_GFP_LN/GOstats/GOstats_MF_Up_pieChart.pdf]

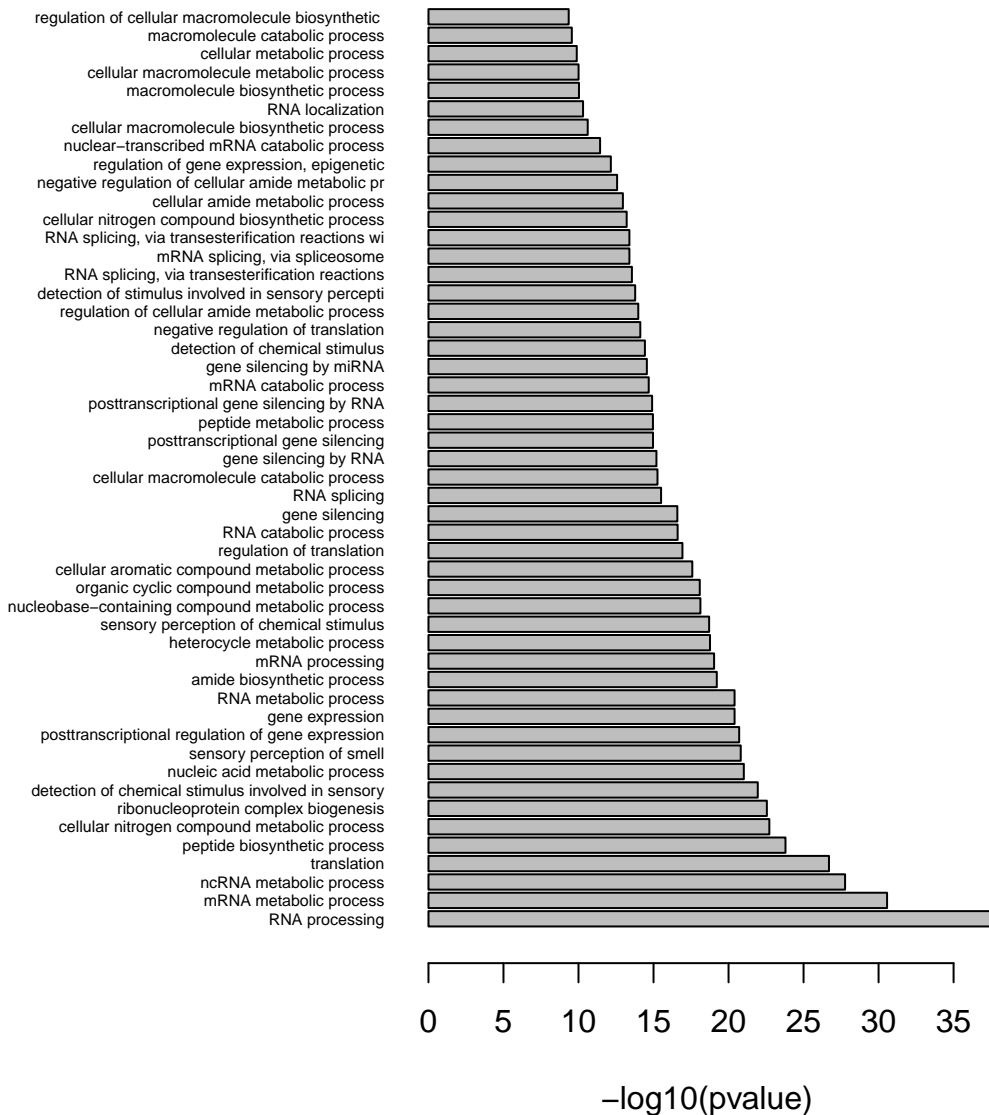

Supplement: DATASET S6 — GO-term analyses of GATA3-expressing versus EGFP-expressing unlesioned pHAs in 3D. [file Data_Sheet_6.ZIP › SD6_GATA3_vs_GFP_LN/GOstats/pVal_GOstats_BP_Down_pieChart.pdf]

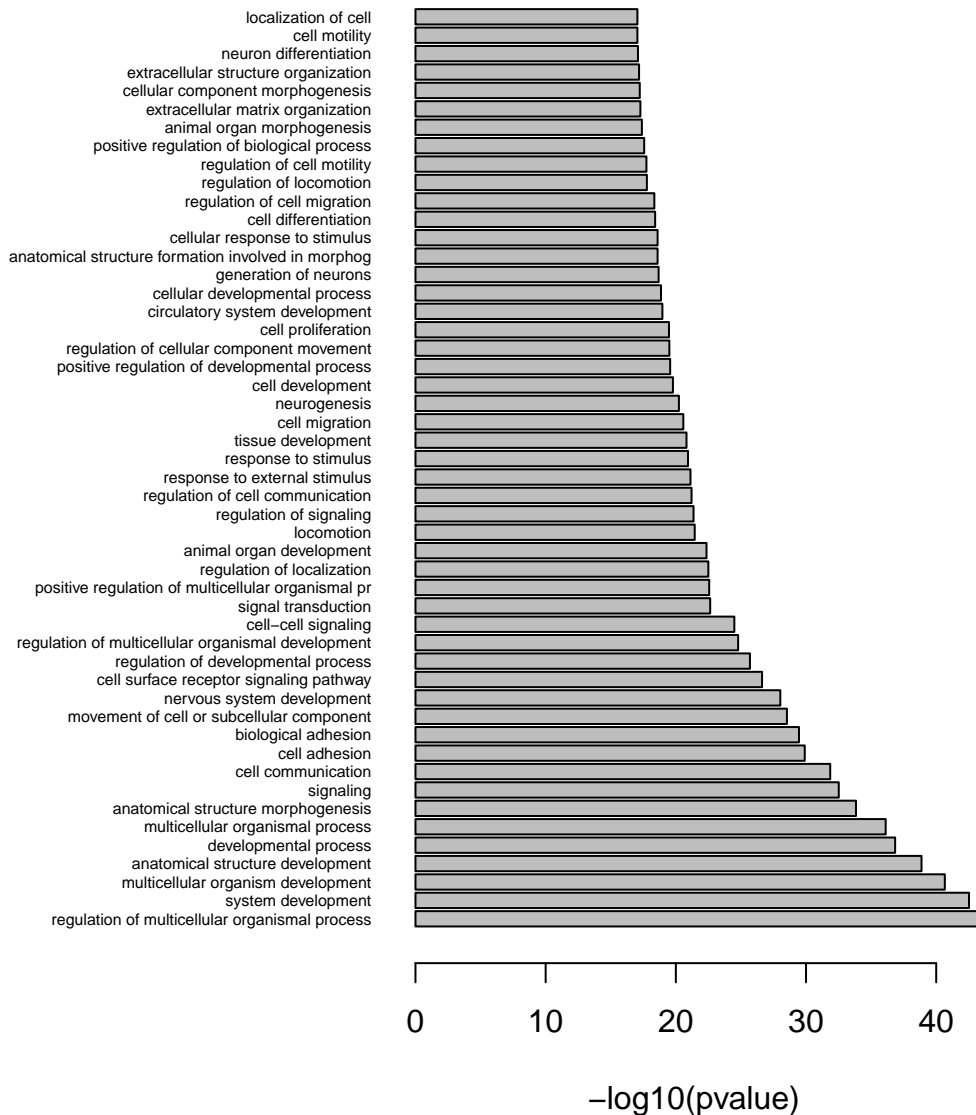

Supplement: DATASET S6 — GO-term analyses of GATA3-expressing versus EGFP-expressing unlesioned pHAs in 3D. [file Data_Sheet_6.ZIP › SD6_GATA3_vs_GFP_LN/GOstats/pVal_GOstats_BP_Up_pieChart.pdf]

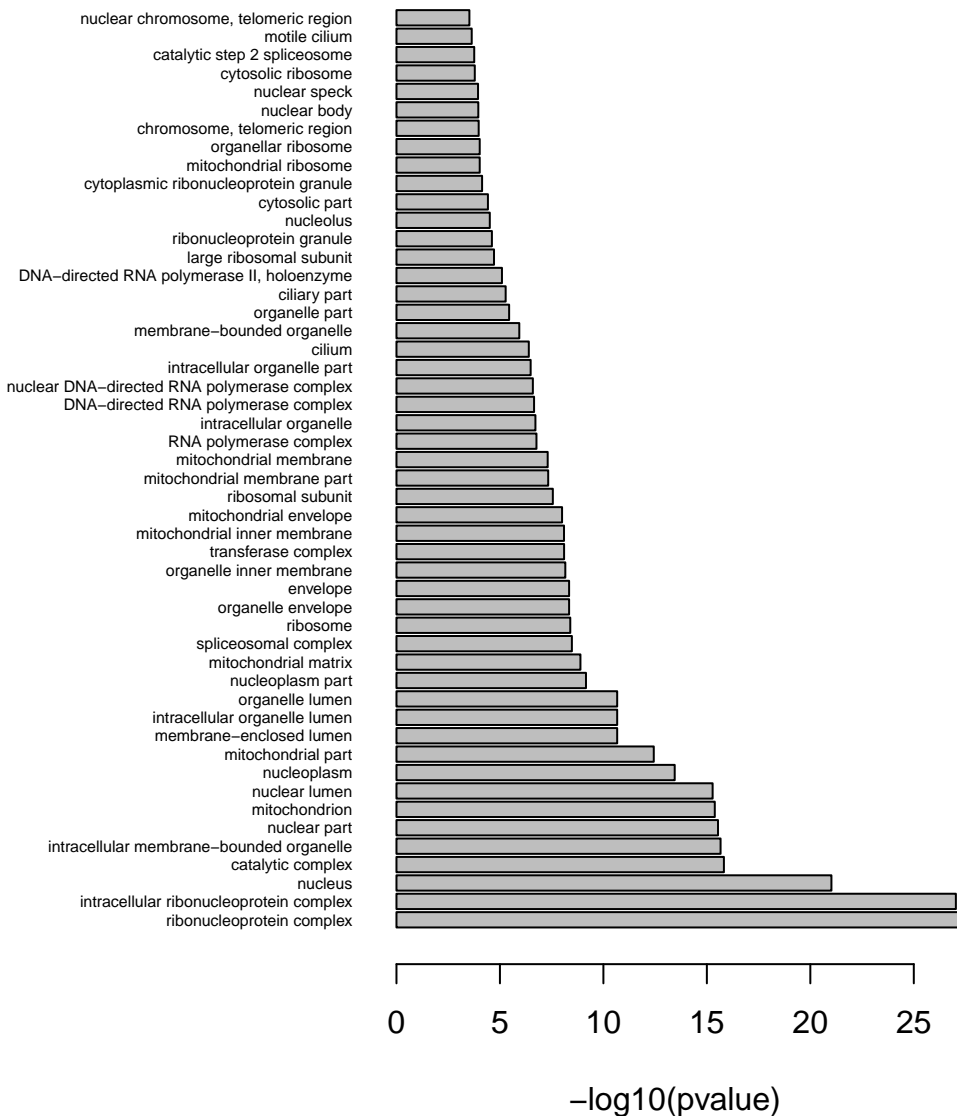

Supplement: DATASET S6 — GO-term analyses of GATA3-expressing versus EGFP-expressing unlesioned pHAs in 3D. [file Data_Sheet_6.ZIP › SD6_GATA3_vs_GFP_LN/GOstats/pVal_GOstats_CC_Down_pieChart.pdf]

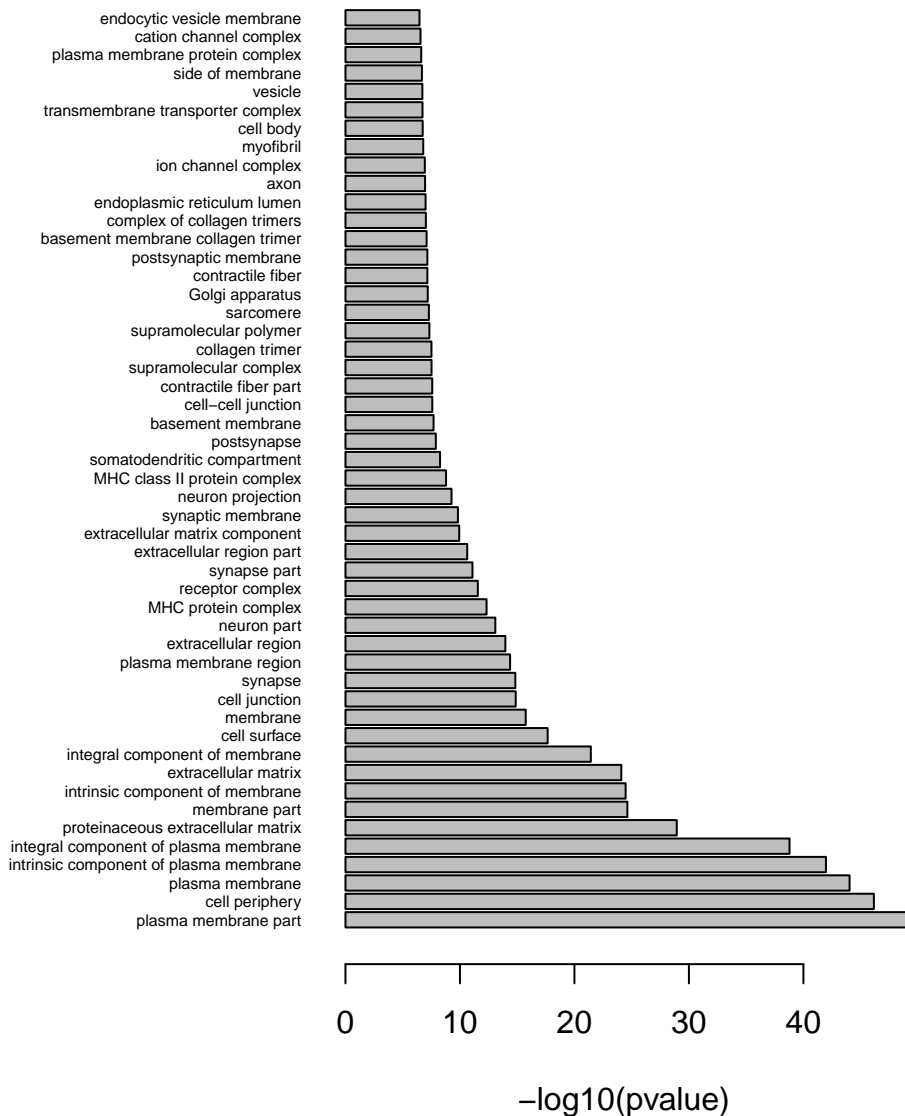

Supplement: DATASET S6 — GO-term analyses of GATA3-expressing versus EGFP-expressing unlesioned pHAs in 3D. [file Data_Sheet_6.ZIP › SD6_GATA3_vs_GFP_LN/GOstats/pVal_GOstats_CC_Up_pieChart.pdf]

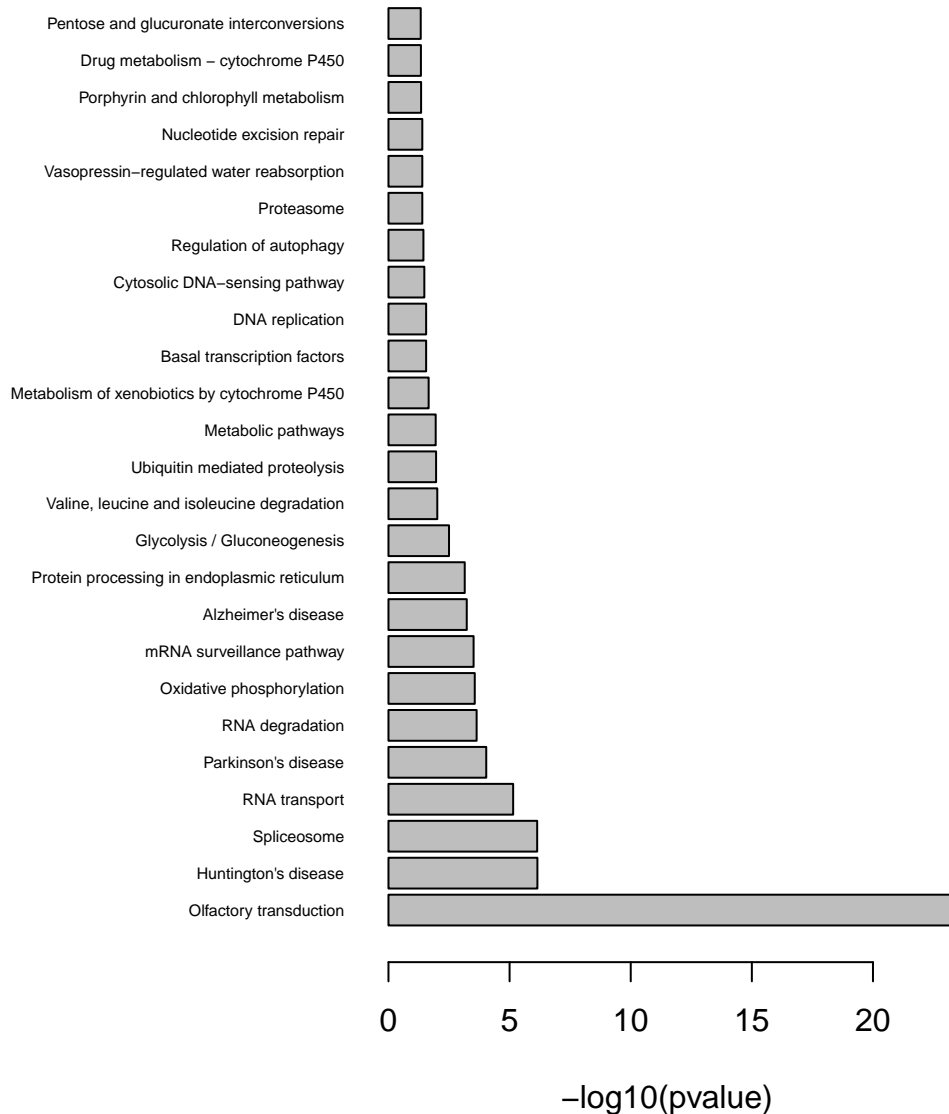

Supplement: DATASET S6 — GO-term analyses of GATA3-expressing versus EGFP-expressing unlesioned pHAs in 3D. [file Data_Sheet_6.ZIP › SD6_GATA3_vs_GFP_LN/GOstats/pVal_GOstats_kegg_Under.pdf]

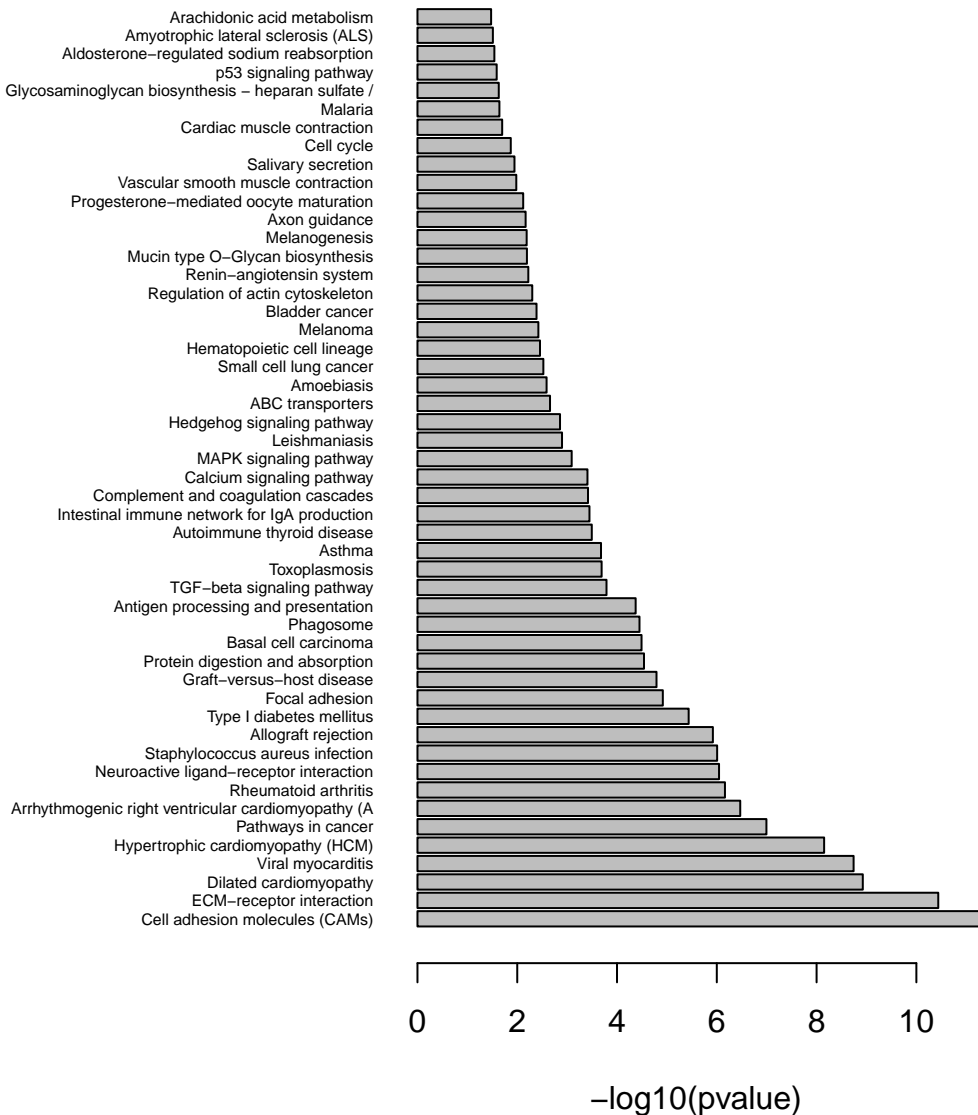

Supplement: DATASET S6 — GO-term analyses of GATA3-expressing versus EGFP-expressing unlesioned pHAs in 3D. [file Data_Sheet_6.ZIP › SD6_GATA3_vs_GFP_LN/GOstats/pVal_GOstats_kegg_Up.pdf]

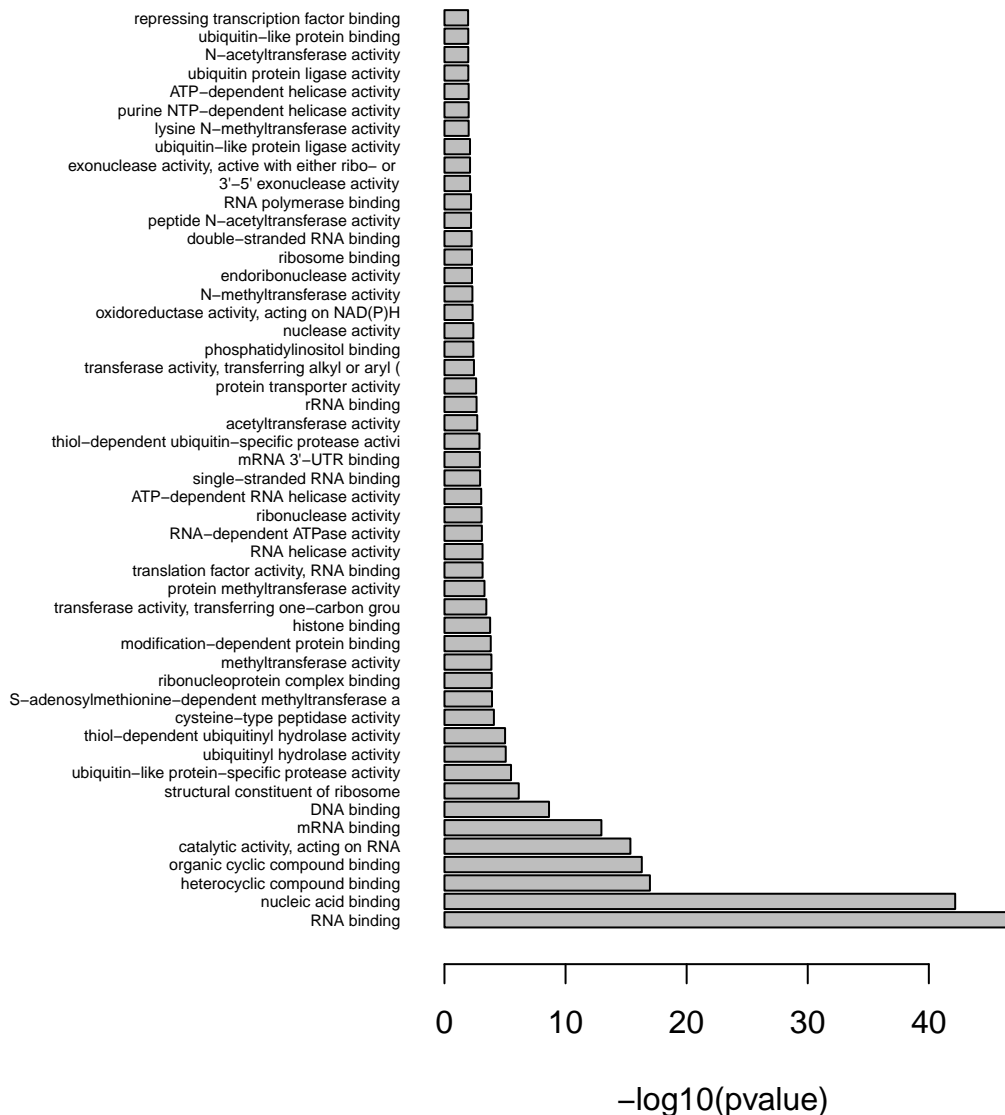

Supplement: DATASET S6 — GO-term analyses of GATA3-expressing versus EGFP-expressing unlesioned pHAs in 3D. [file Data_Sheet_6.ZIP › SD6_GATA3_vs_GFP_LN/GOstats/pVal_GOstats_MF_Down_pieChart.pdf]

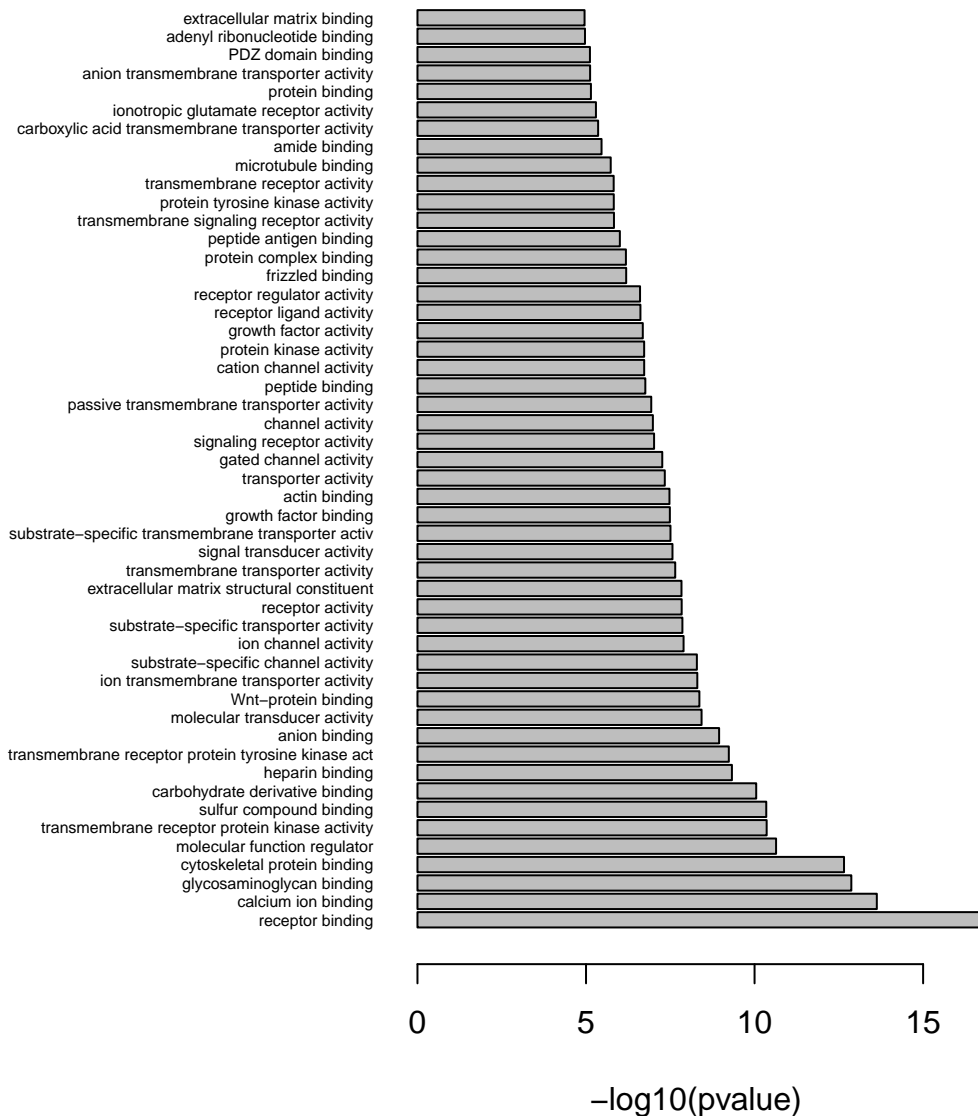

Supplement: DATASET S6 — GO-term analyses of GATA3-expressing versus EGFP-expressing unlesioned pHAs in 3D. [file Data_Sheet_6.ZIP › SD6_GATA3_vs_GFP_LN/GOstats/pVal_GOstats_MF_Up_pieChart.pdf]

# Gata3\_LP\_vs\_GFP\_LP

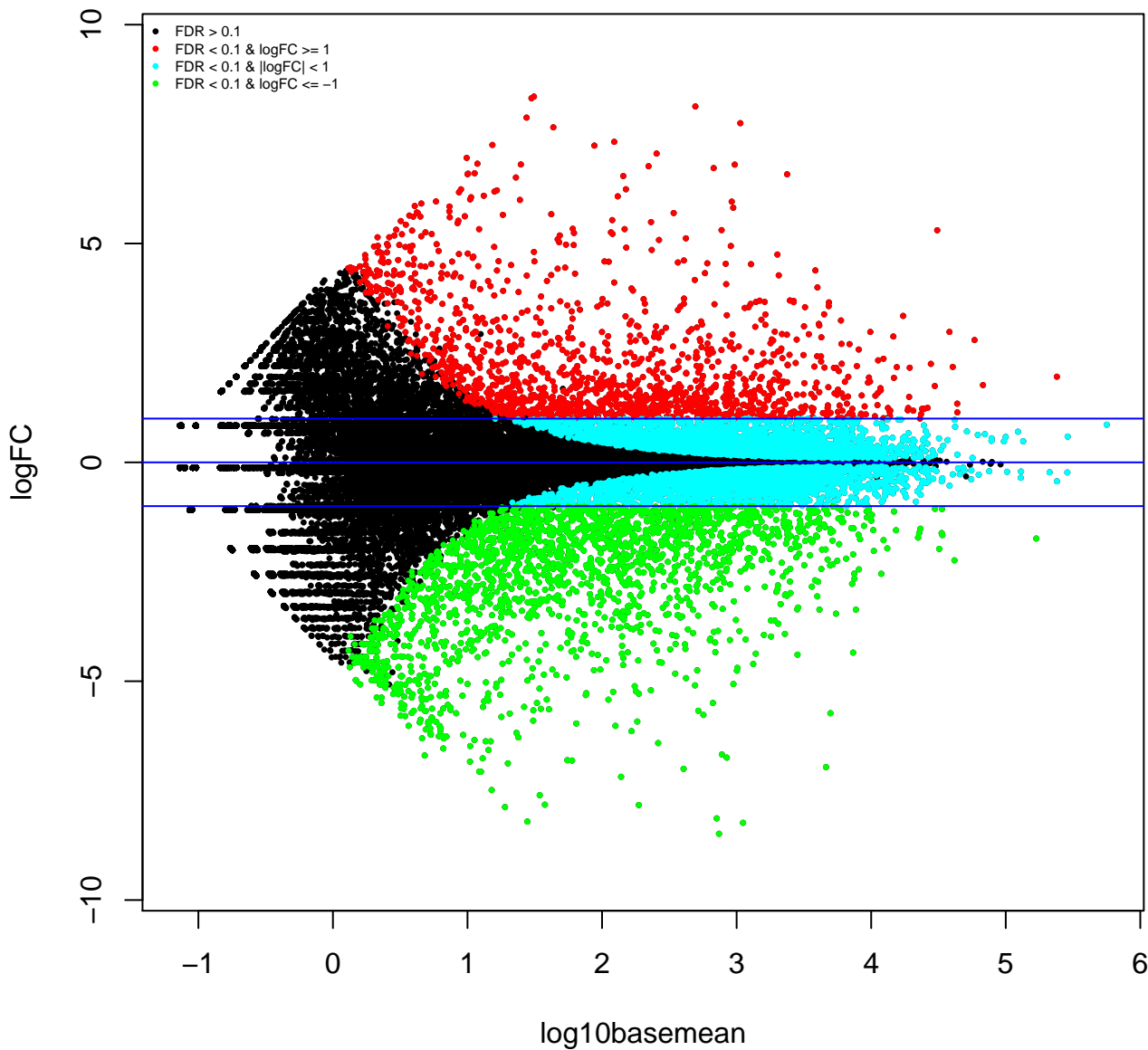

Supplement: DATASET S7 — GO-term analyses of GATA3-expressing versus EGFP-expressing lesioned pHAs in 3D. [file Data_Sheet_7.ZIP › SD7_GATA3_vs_GFP_LP/Gata3_LP_vs_GFP_LP_MAplot.pdf]

# Gata3\_LP\_vs\_GFP\_LP

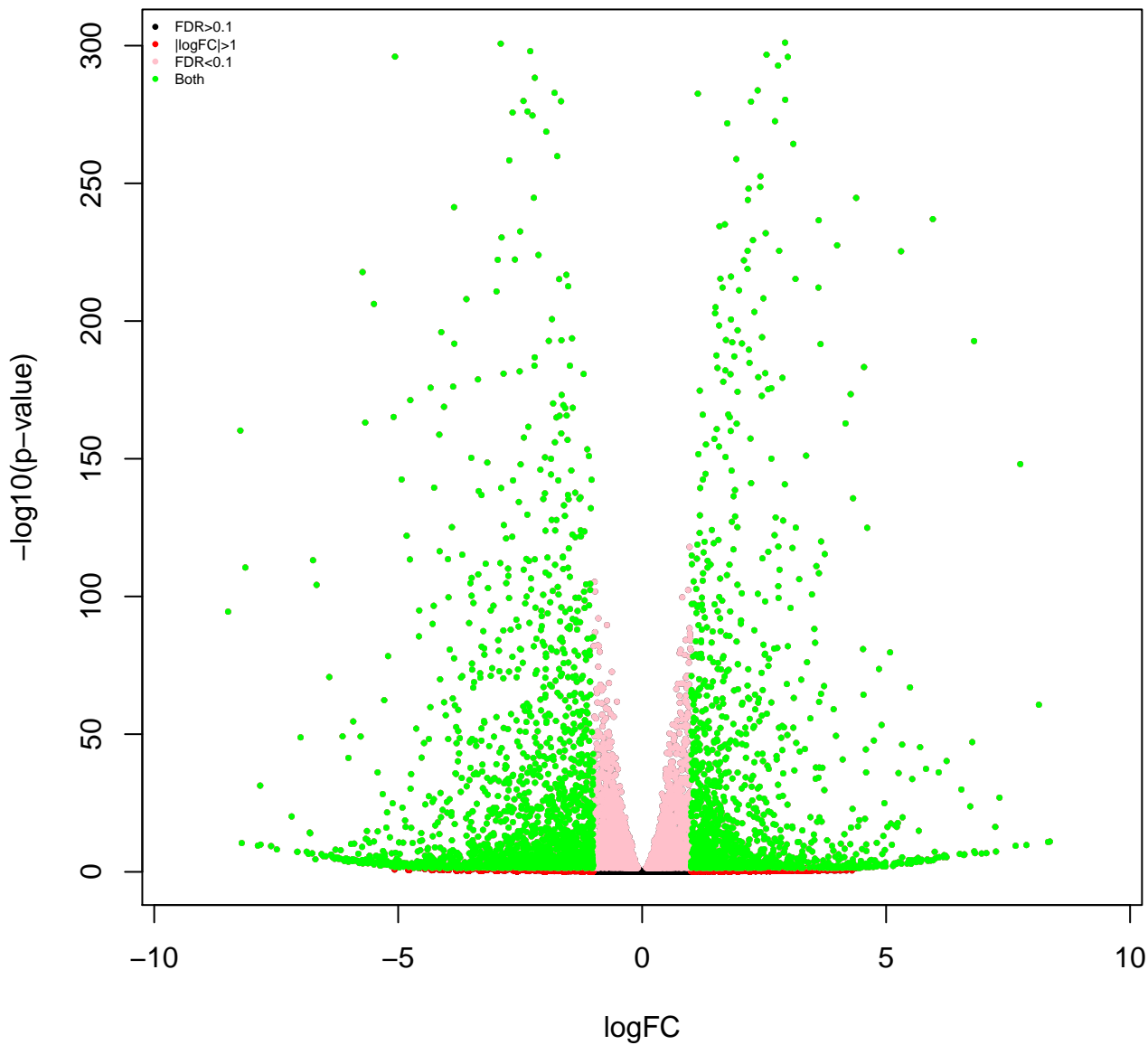

Supplement: DATASET S7 — GO-term analyses of GATA3-expressing versus EGFP-expressing lesioned pHAs in 3D. [file Data_Sheet_7.ZIP › SD7_GATA3_vs_GFP_LP/Gata3_LP_vs_GFP_LP_Volcanoplot.pdf]

GOstats\_BP\_Down\_pieChart

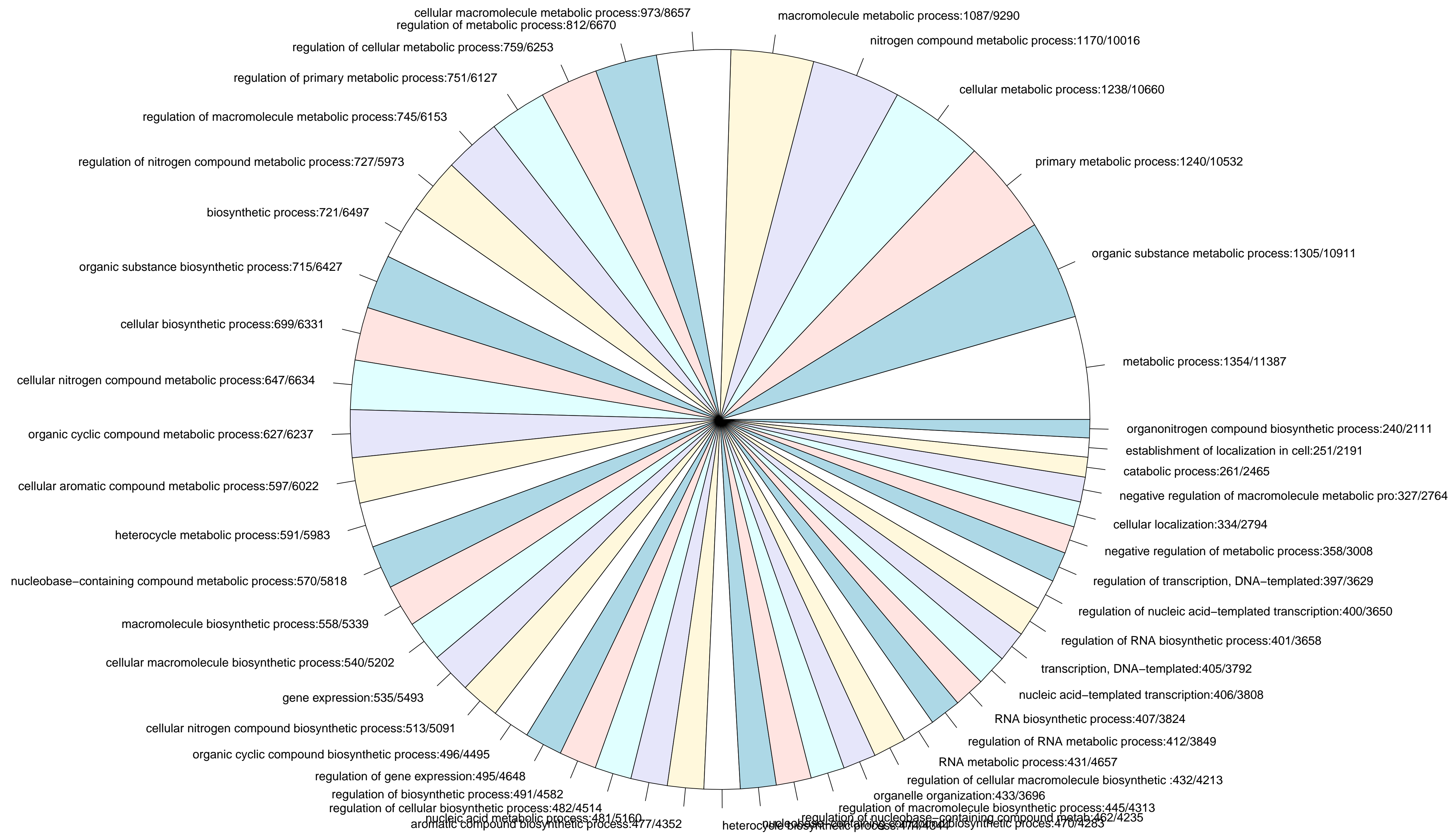

Supplement: DATASET S7 — GO-term analyses of GATA3-expressing versus EGFP-expressing lesioned pHAs in 3D. [file Data_Sheet_7.ZIP › SD7_GATA3_vs_GFP_LP/GOstats/GOstats_BP_Down_pieChart.pdf]

GOstats\_BP\_Up\_pieChart

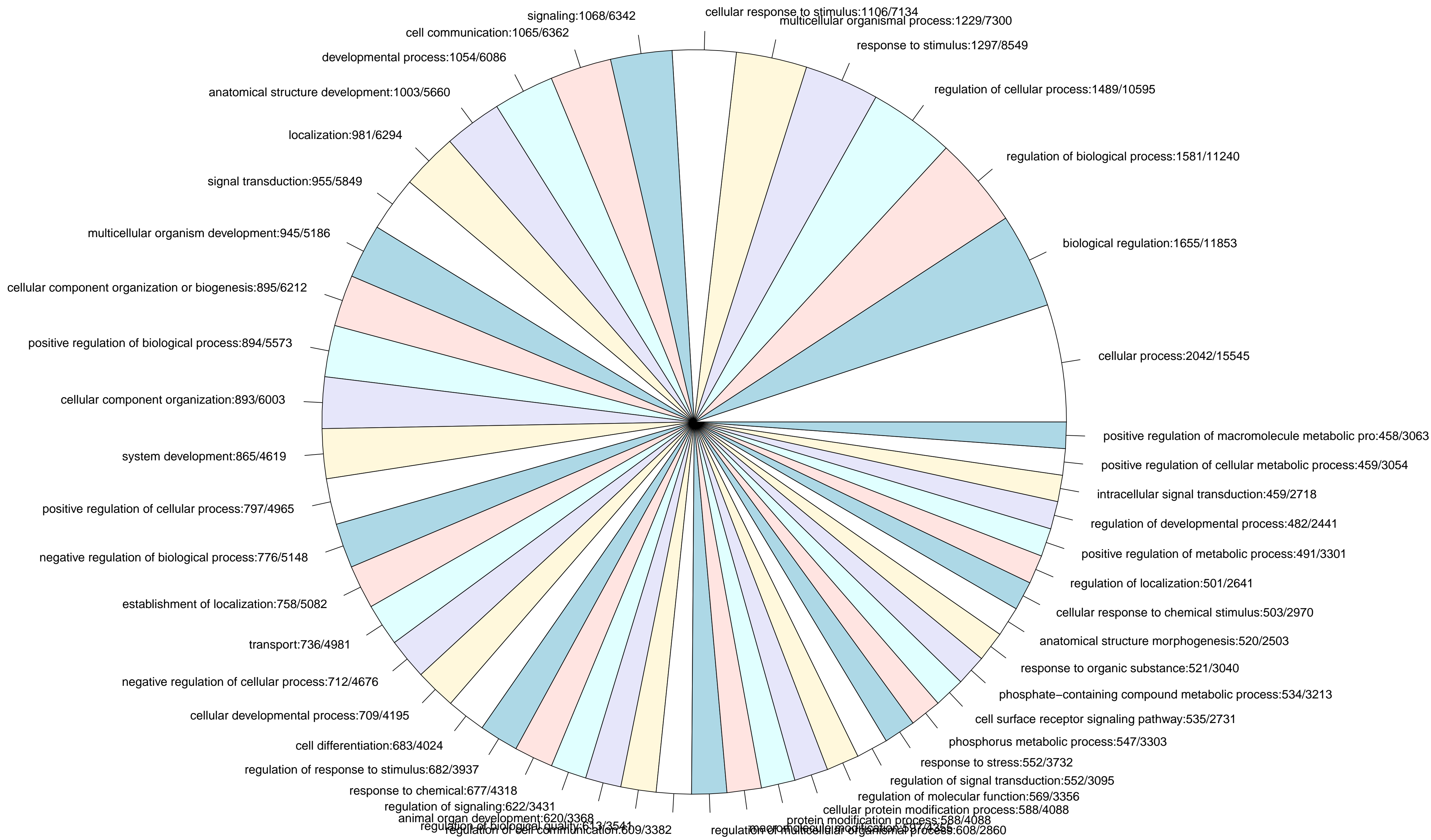

Supplement: DATASET S7 — GO-term analyses of GATA3-expressing versus EGFP-expressing lesioned pHAs in 3D. [file Data_Sheet_7.ZIP › SD7_GATA3_vs_GFP_LP/GOstats/GOstats_BP_Up_pieChart.pdf]

GOstats\_CC\_Down\_pieChart

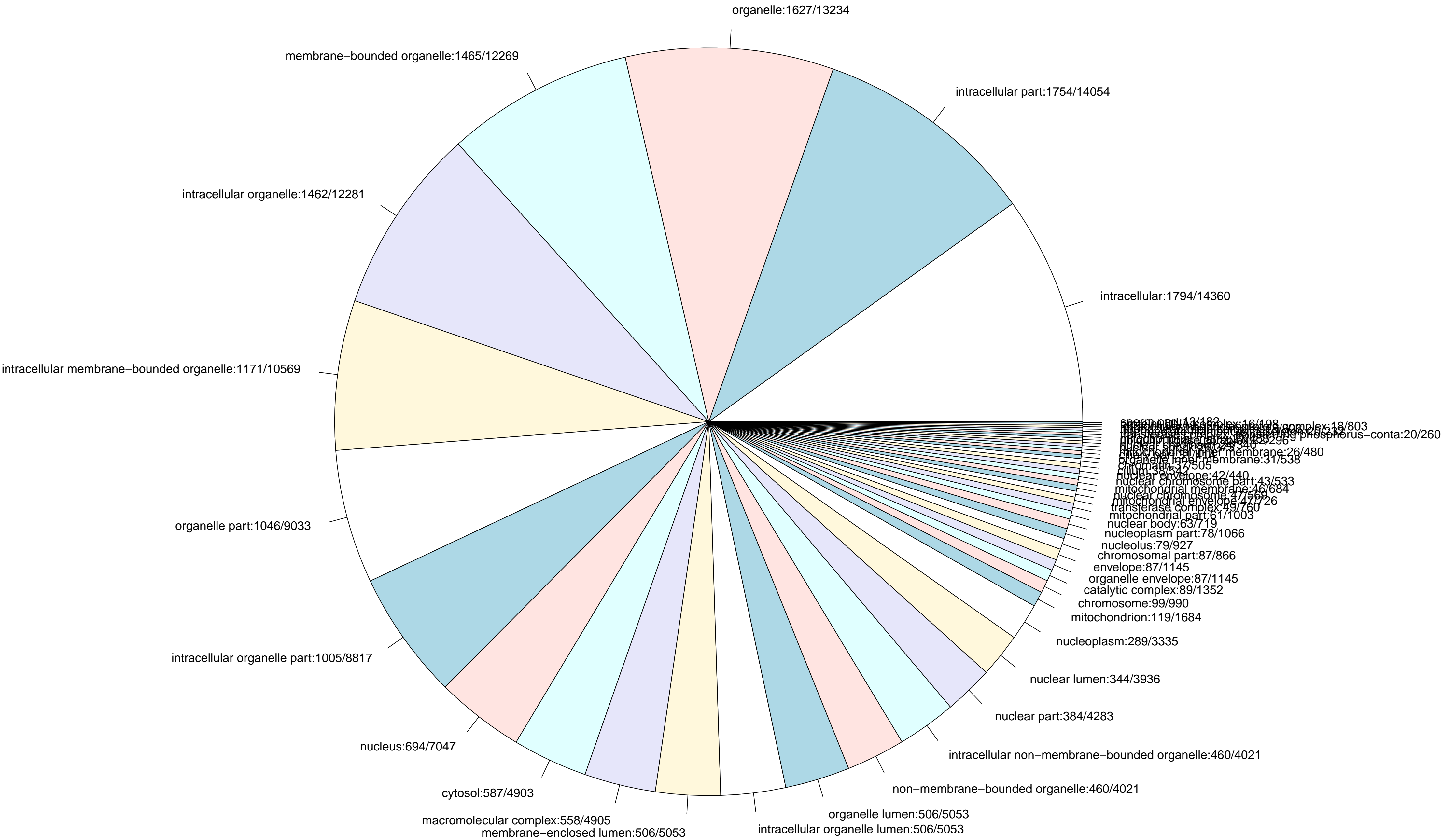

Supplement: DATASET S7 — GO-term analyses of GATA3-expressing versus EGFP-expressing lesioned pHAs in 3D. [file Data_Sheet_7.ZIP › SD7_GATA3_vs_GFP_LP/GOstats/GOstats_CC_Down_pieChart.pdf]

GOstats\_CC\_Up\_pieChart

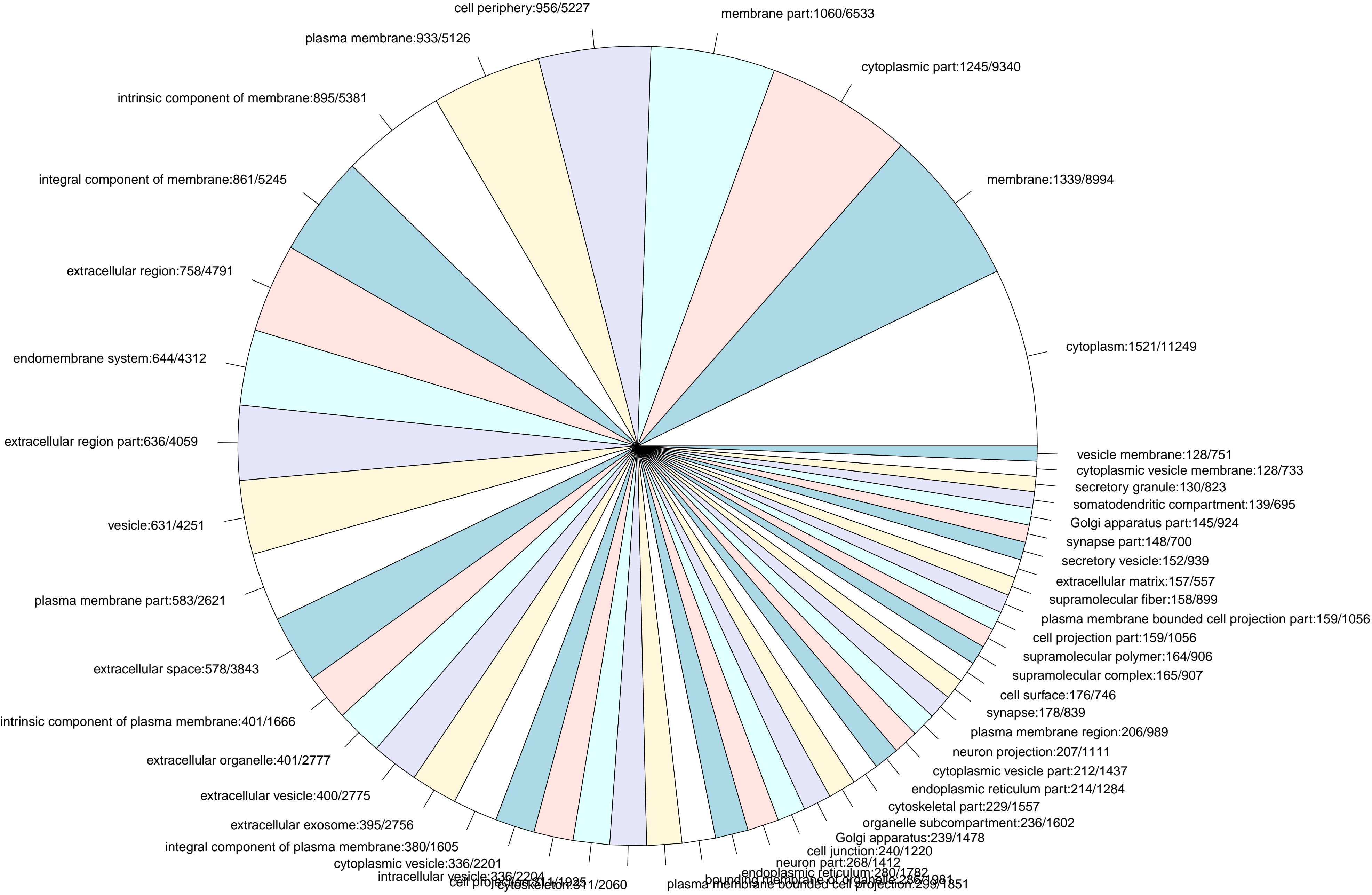

Supplement: DATASET S7 — GO-term analyses of GATA3-expressing versus EGFP-expressing lesioned pHAs in 3D. [file Data_Sheet_7.ZIP › SD7_GATA3_vs_GFP_LP/GOstats/GOstats_CC_Up_pieChart.pdf]

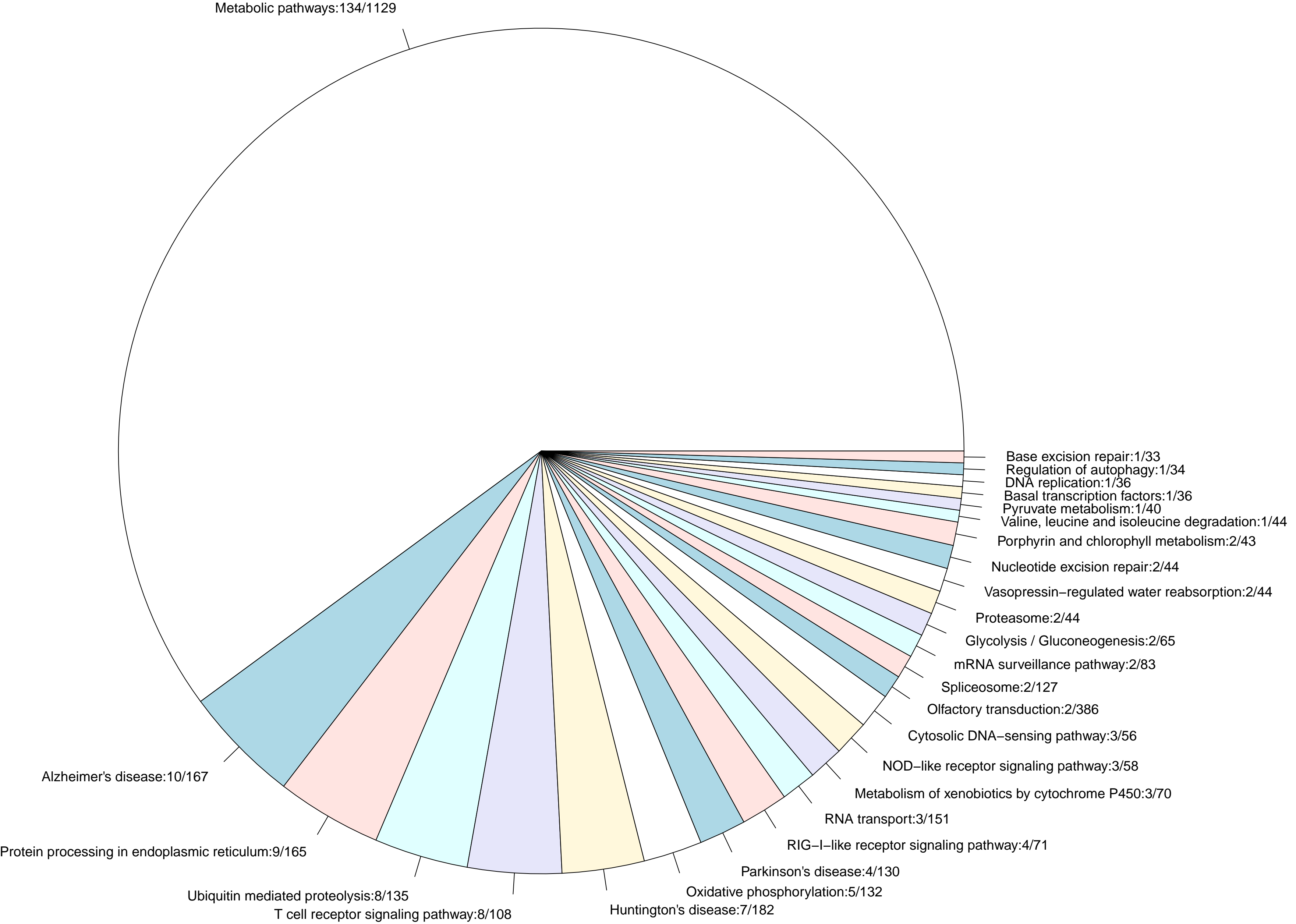

Supplement: DATASET S7 — GO-term analyses of GATA3-expressing versus EGFP-expressing lesioned pHAs in 3D. [file Data_Sheet_7.ZIP › SD7_GATA3_vs_GFP_LP/GOstats/GOstats_kegg_Under.pdf]

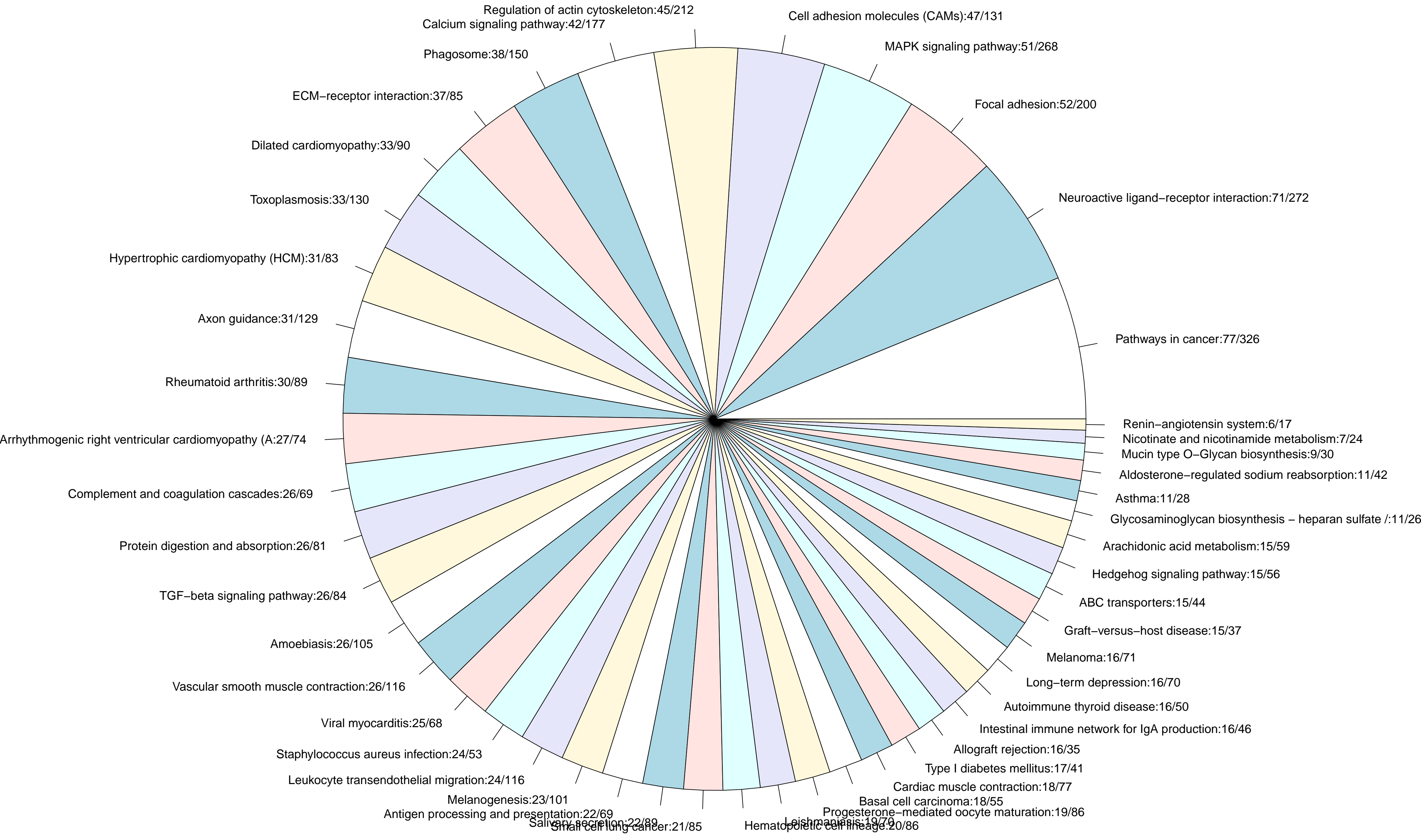

Supplement: DATASET S7 — GO-term analyses of GATA3-expressing versus EGFP-expressing lesioned pHAs in 3D. [file Data_Sheet_7.ZIP › SD7_GATA3_vs_GFP_LP/GOstats/GOstats_kegg_Up.pdf]

### GOstats\_MF\_Down\_pieChart

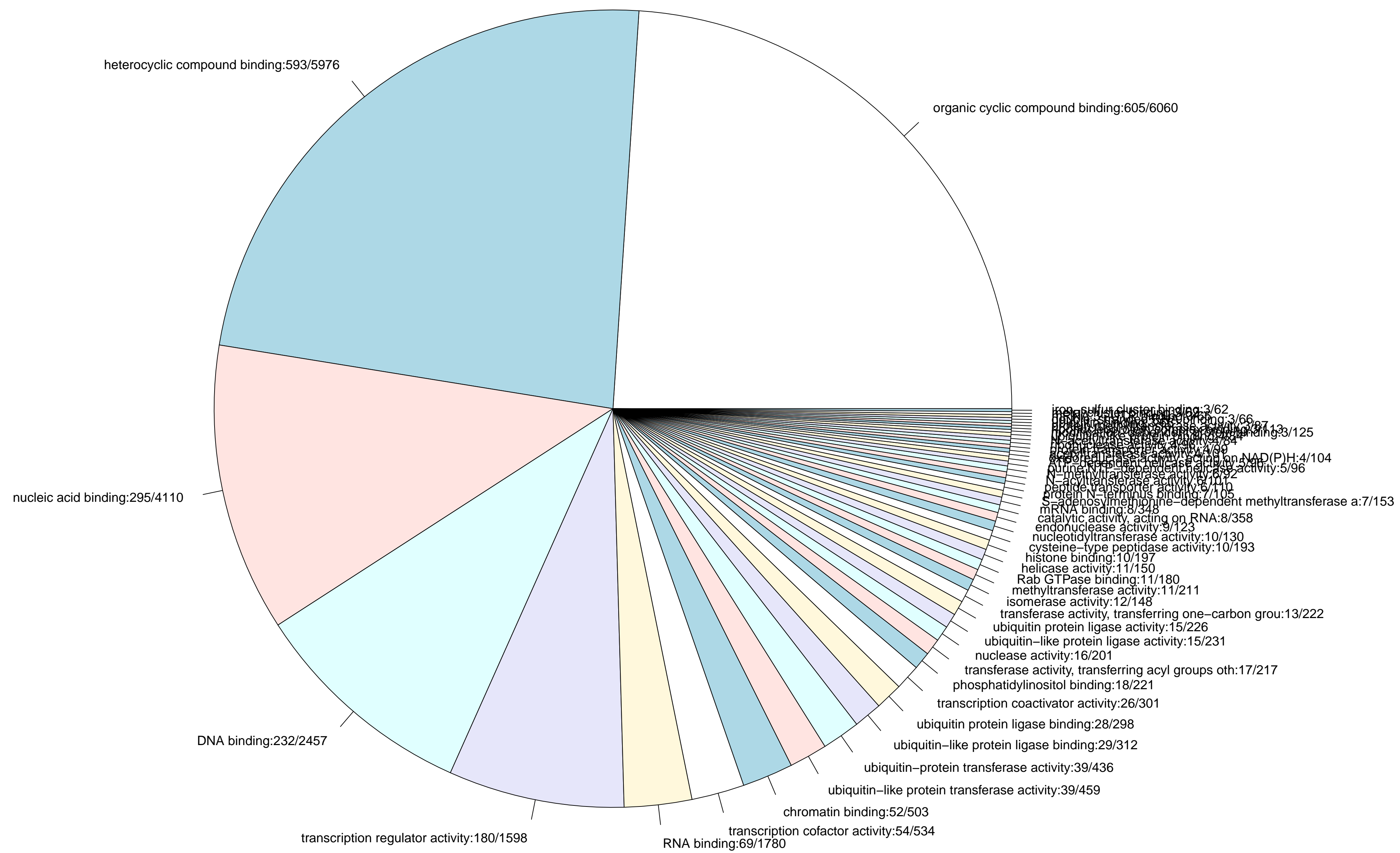

Supplement: DATASET S7 — GO-term analyses of GATA3-expressing versus EGFP-expressing lesioned pHAs in 3D. [file Data_Sheet_7.ZIP › SD7_GATA3_vs_GFP_LP/GOstats/GOstats_MF_Down_pieChart.pdf]

GOstats\_MF\_Up\_pieChart

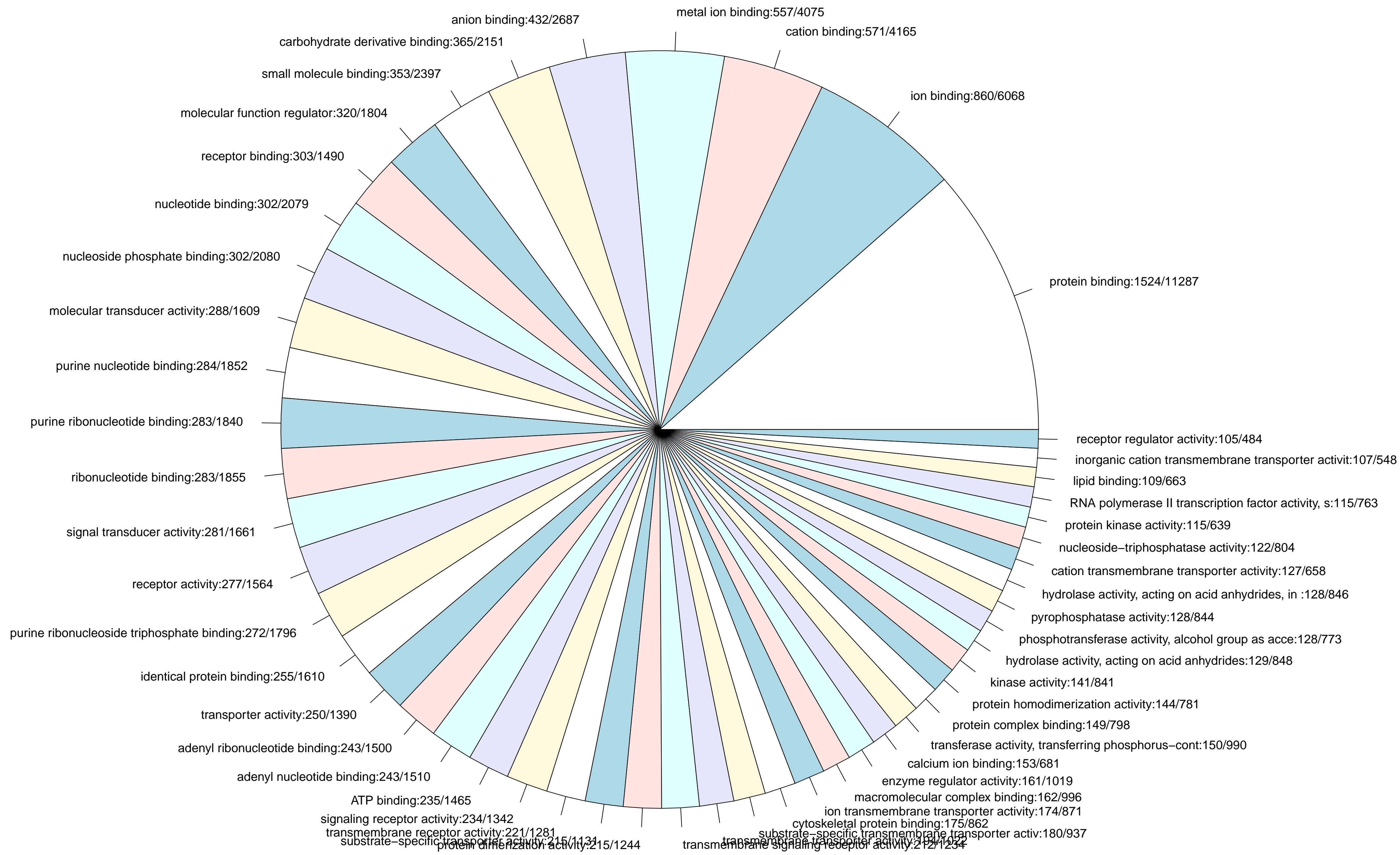

Supplement: DATASET S7 — GO-term analyses of GATA3-expressing versus EGFP-expressing lesioned pHAs in 3D. [file Data_Sheet_7.ZIP › SD7_GATA3_vs_GFP_LP/GOstats/GOstats_MF_Up_pieChart.pdf]

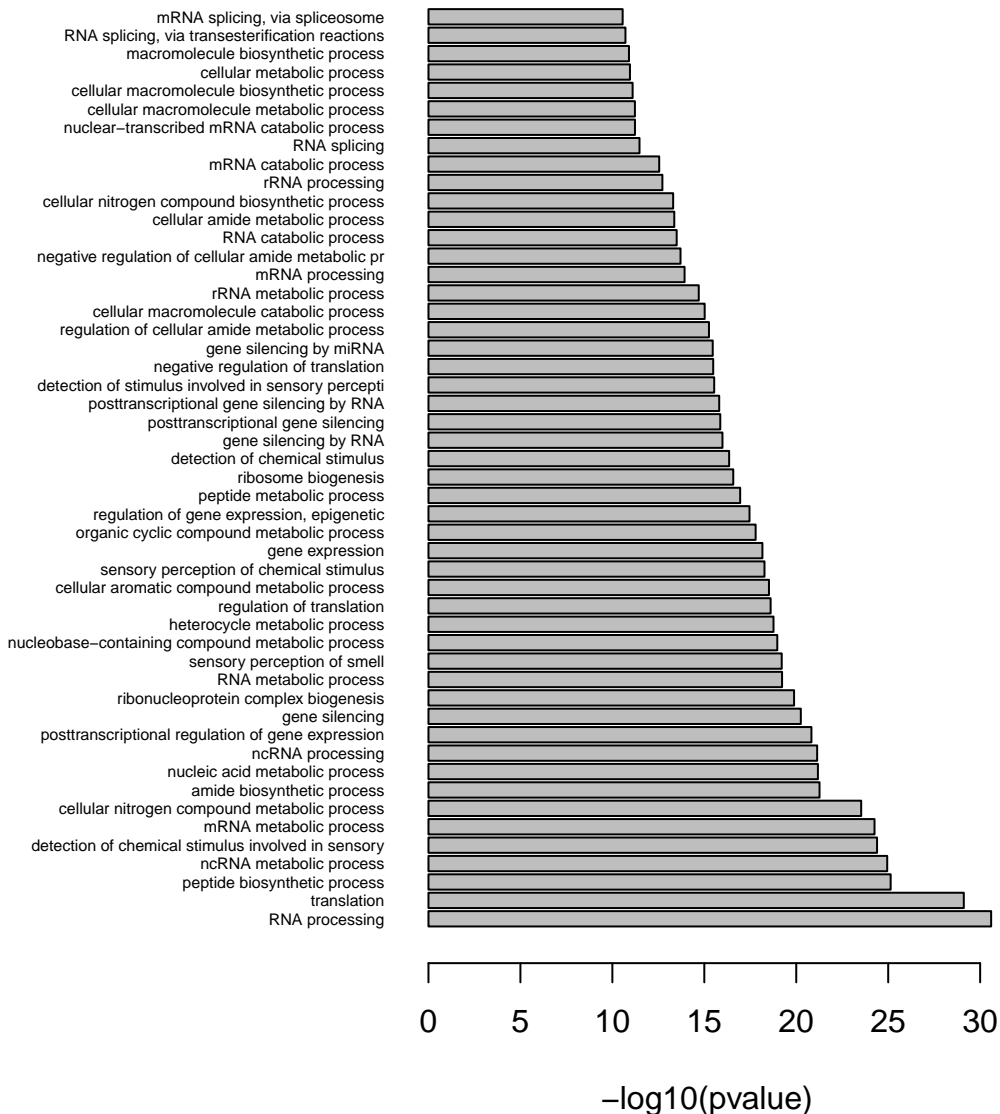

Supplement: DATASET S7 — GO-term analyses of GATA3-expressing versus EGFP-expressing lesioned pHAs in 3D. [file Data_Sheet_7.ZIP › SD7_GATA3_vs_GFP_LP/GOstats/pVal_GOstats_BP_Down_pieChart.pdf]

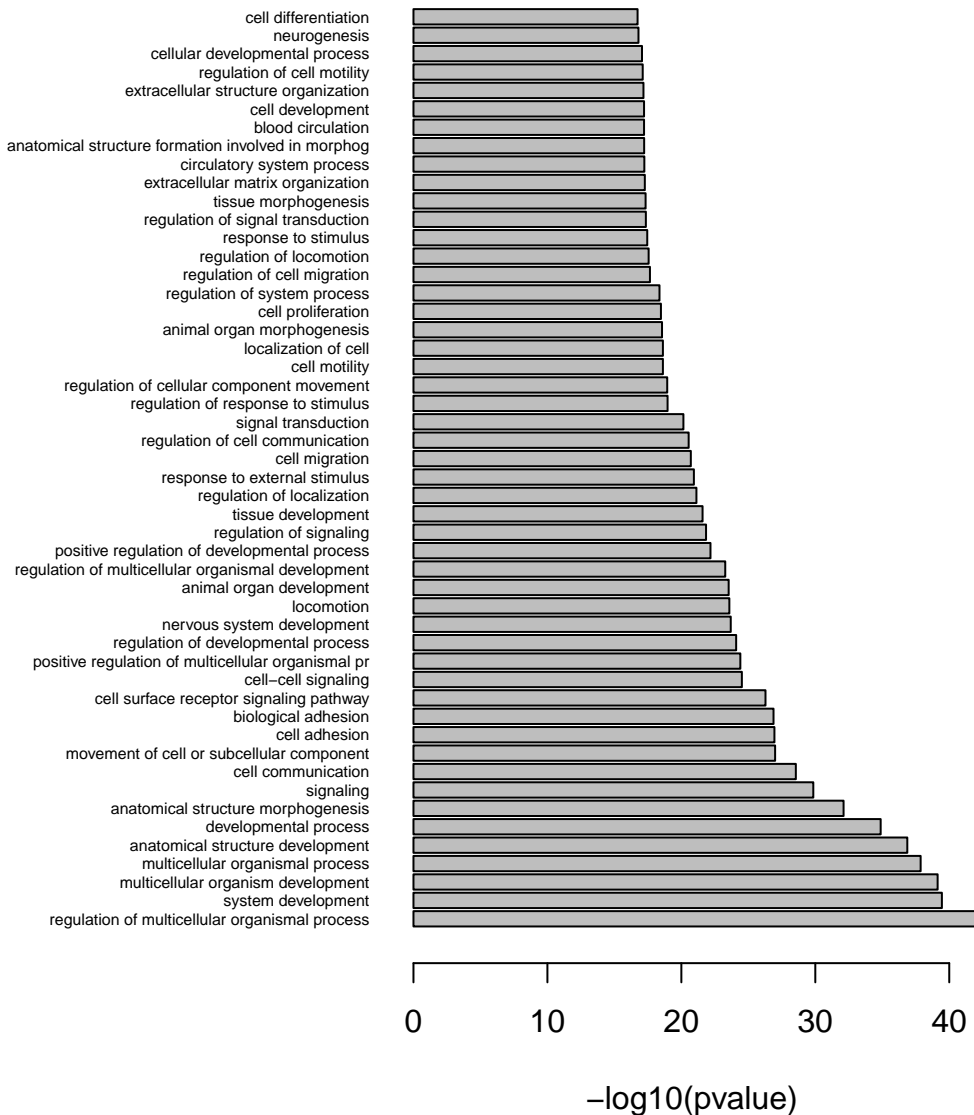

Supplement: DATASET S7 — GO-term analyses of GATA3-expressing versus EGFP-expressing lesioned pHAs in 3D. [file Data_Sheet_7.ZIP › SD7_GATA3_vs_GFP_LP/GOstats/pVal_GOstats_BP_Up_pieChart.pdf]

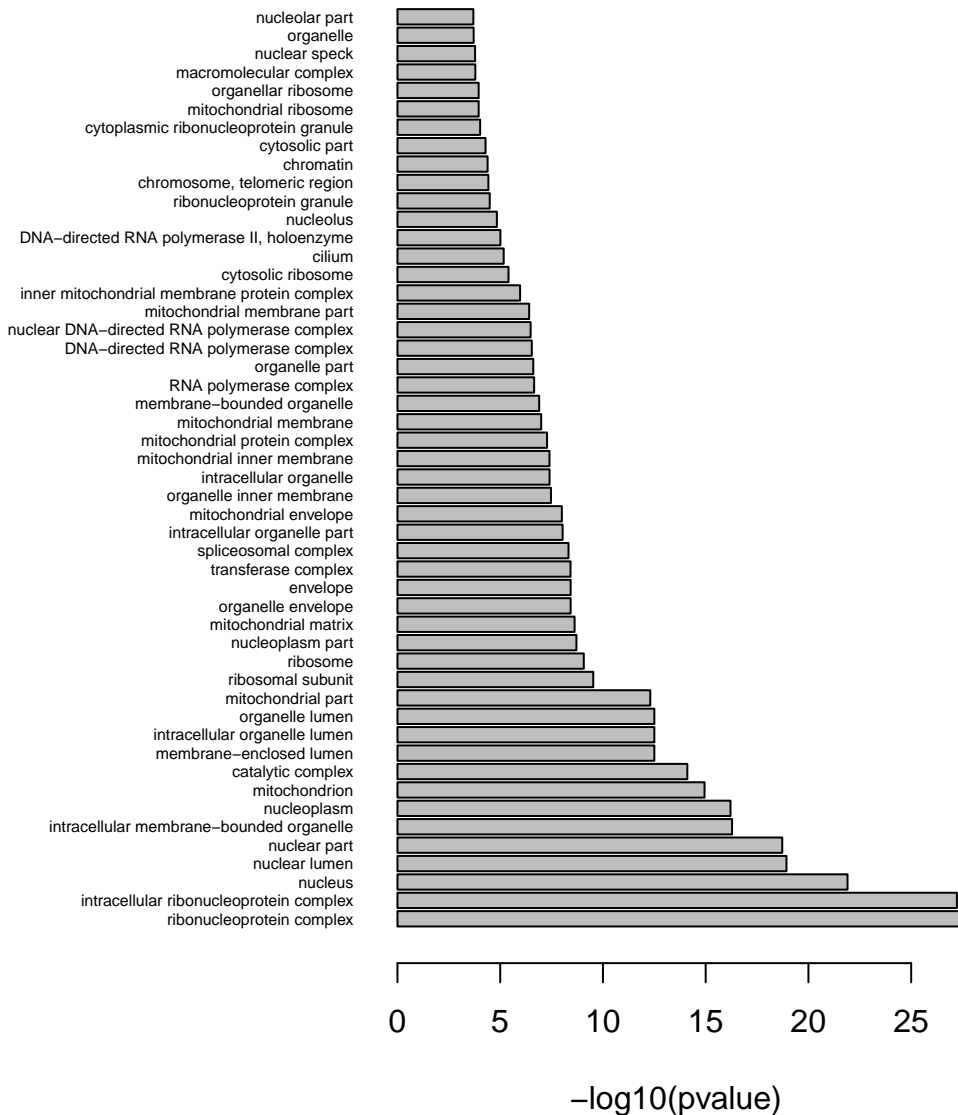

Supplement: DATASET S7 — GO-term analyses of GATA3-expressing versus EGFP-expressing lesioned pHAs in 3D. [file Data_Sheet_7.ZIP › SD7_GATA3_vs_GFP_LP/GOstats/pVal_GOstats_CC_Down_pieChart.pdf]

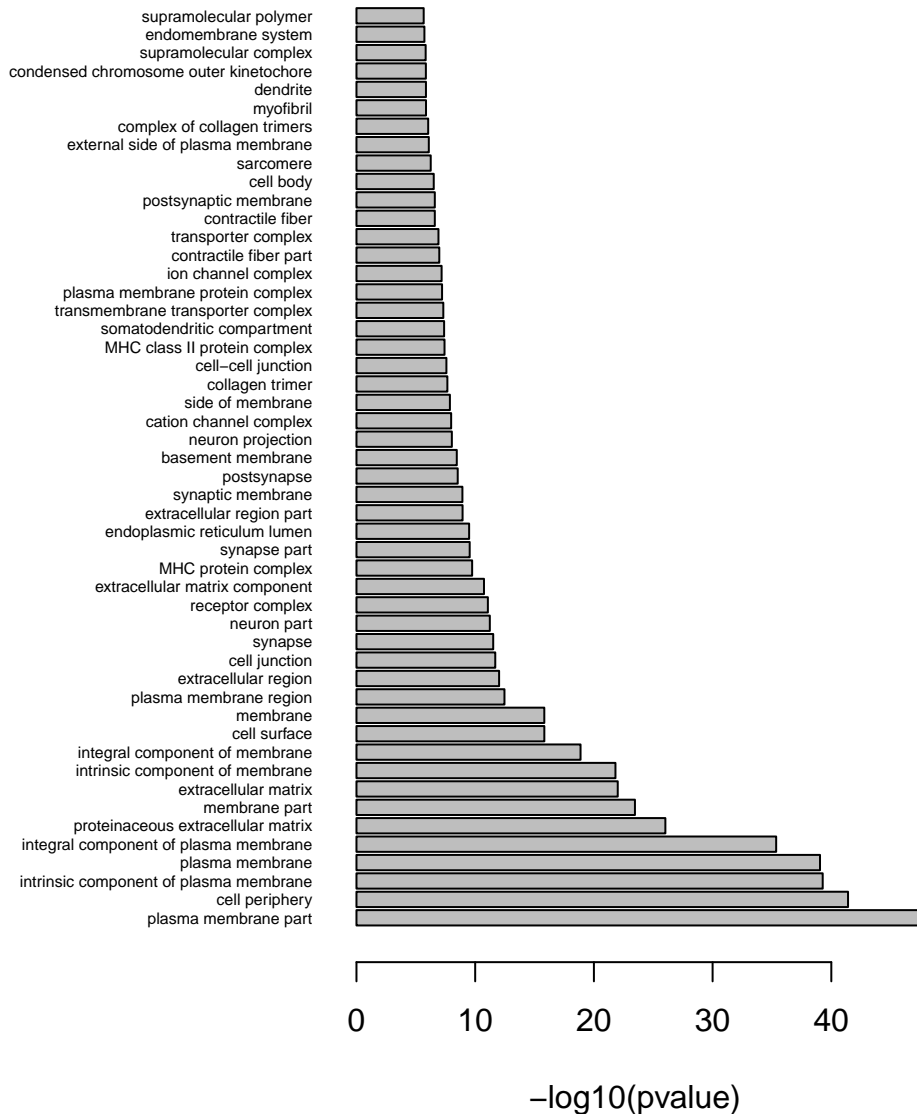

Supplement: DATASET S7 — GO-term analyses of GATA3-expressing versus EGFP-expressing lesioned pHAs in 3D. [file Data_Sheet_7.ZIP › SD7_GATA3_vs_GFP_LP/GOstats/pVal_GOstats_CC_Up_pieChart.pdf]

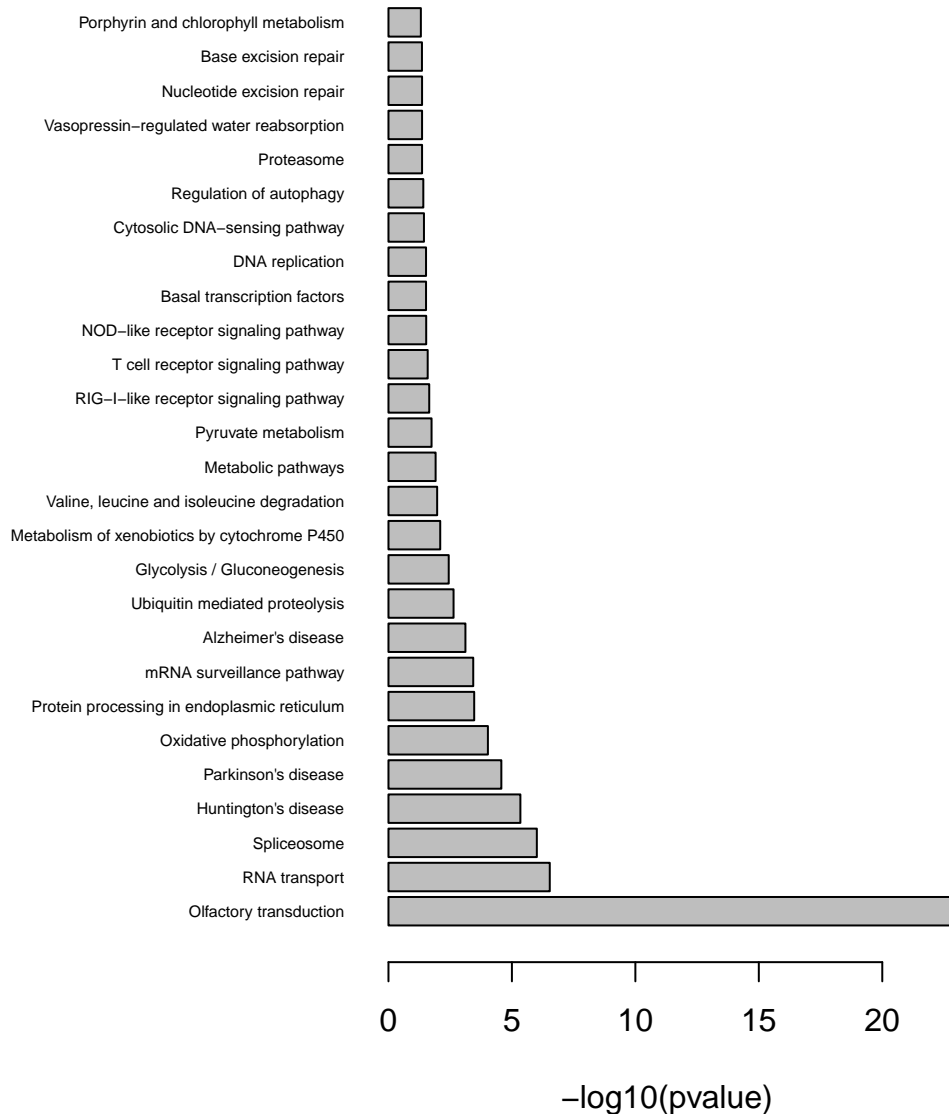

Supplement: DATASET S7 — GO-term analyses of GATA3-expressing versus EGFP-expressing lesioned pHAs in 3D. [file Data_Sheet_7.ZIP › SD7_GATA3_vs_GFP_LP/GOstats/pVal_GOstats_kegg_Under.pdf]

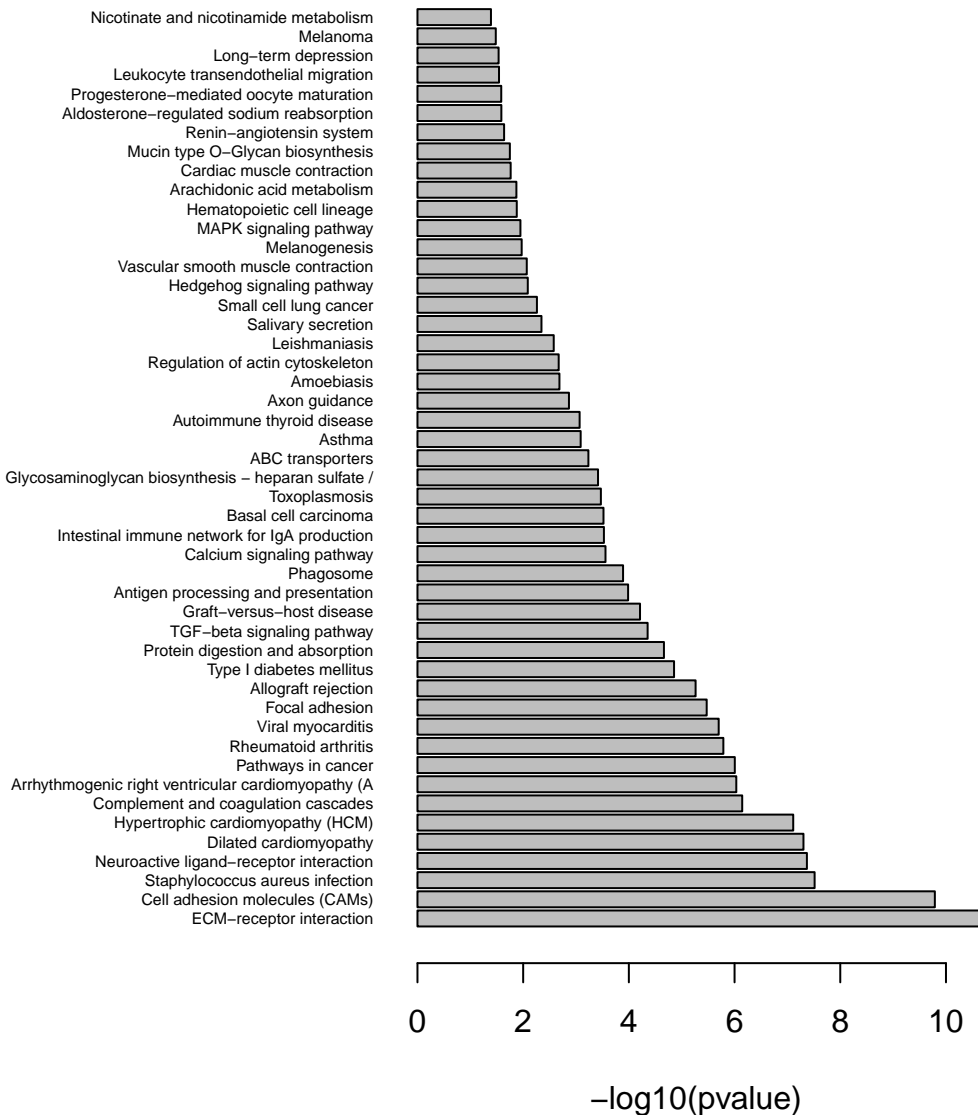

Supplement: DATASET S7 — GO-term analyses of GATA3-expressing versus EGFP-expressing lesioned pHAs in 3D. [file Data_Sheet_7.ZIP › SD7_GATA3_vs_GFP_LP/GOstats/pVal_GOstats_kegg_Up.pdf]

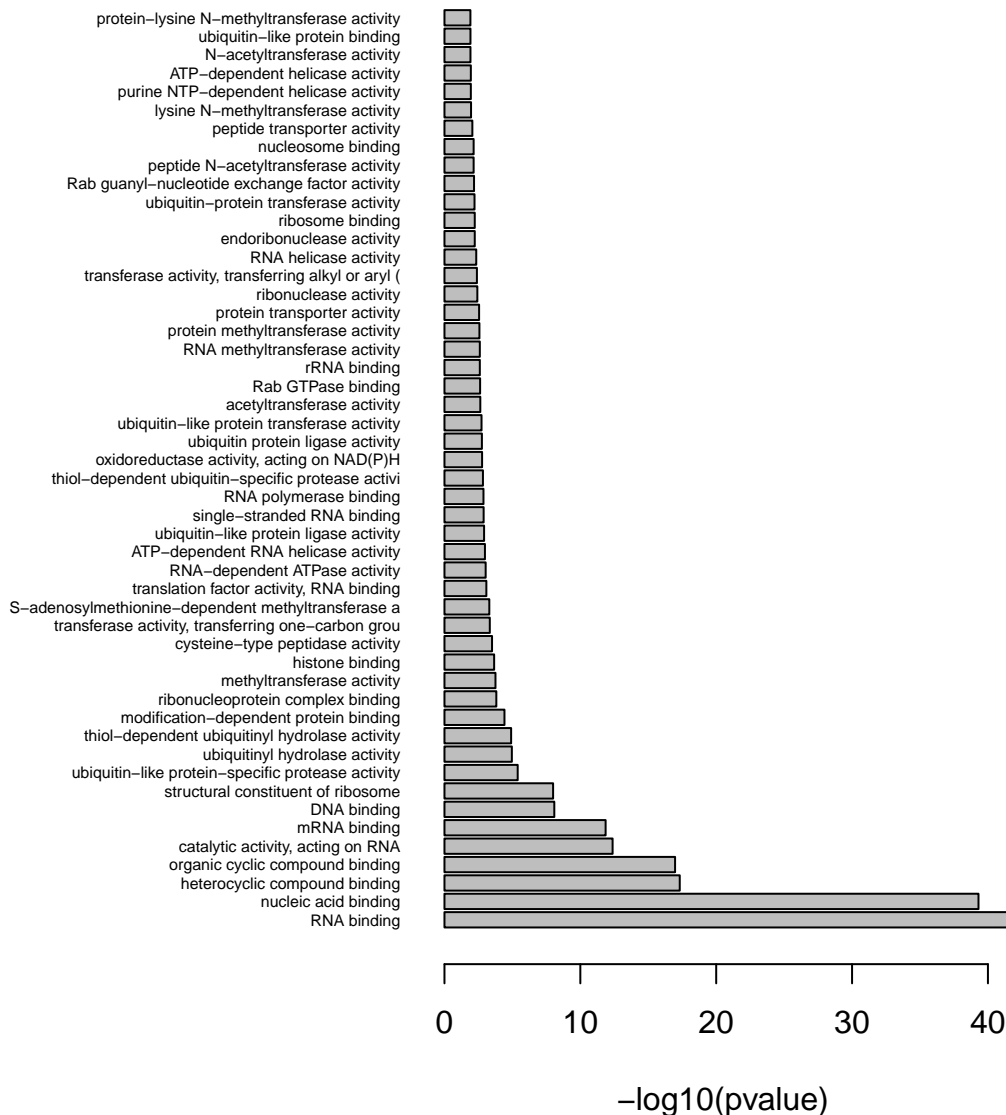

Supplement: DATASET S7 — GO-term analyses of GATA3-expressing versus EGFP-expressing lesioned pHAs in 3D. [file Data_Sheet_7.ZIP › SD7_GATA3_vs_GFP_LP/GOstats/pVal_GOstats_MF_Down_pieChart.pdf]

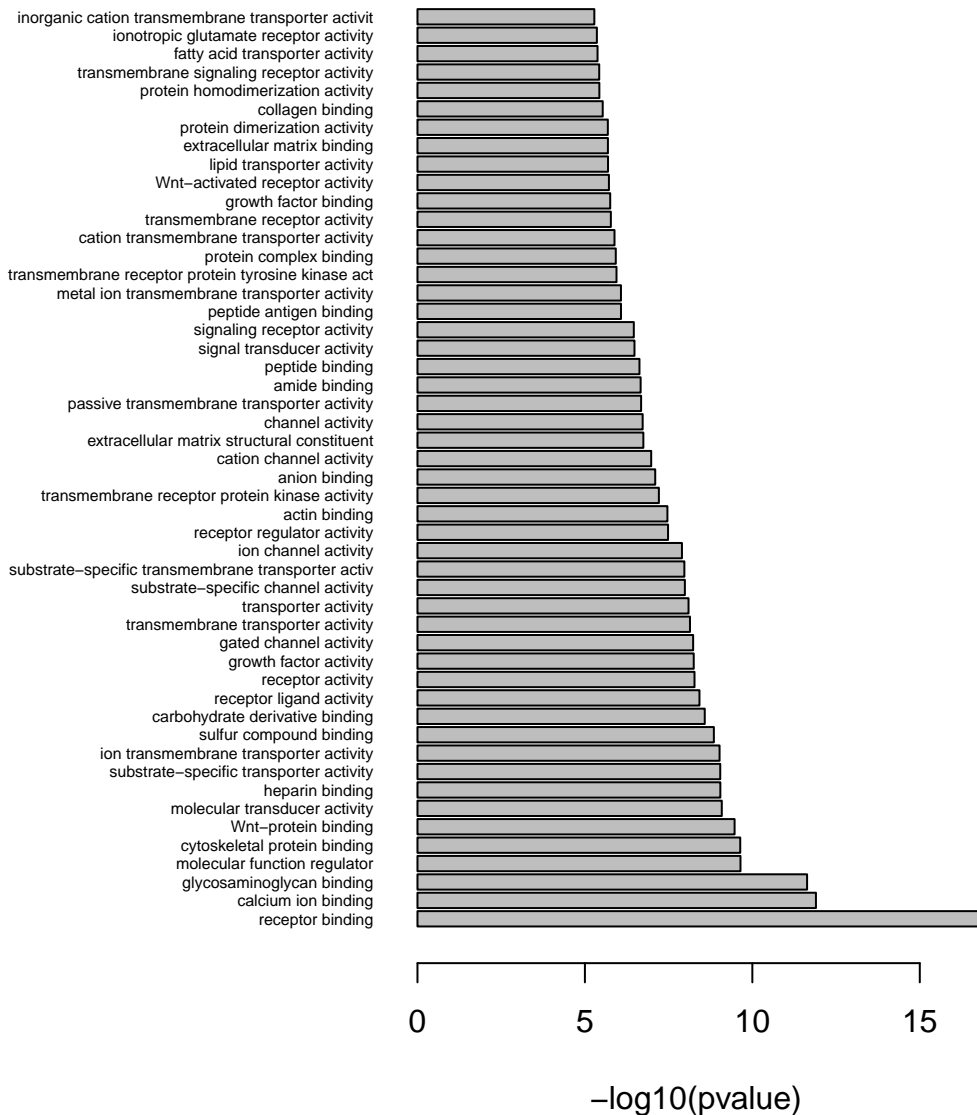

Supplement: DATASET S7 — GO-term analyses of GATA3-expressing versus EGFP-expressing lesioned pHAs in 3D. [file Data_Sheet_7.ZIP › SD7_GATA3_vs_GFP_LP/GOstats/pVal_GOstats_MF_Up_pieChart.pdf]
